# Identification of Circular RNAs from the Parental Genes Involved in Multiple Aspects of Cellular Metabolism in Barley

**DOI:** 10.3389/fpls.2016.00776

**Published:** 2016-06-03

**Authors:** Behrooz Darbani, Shahin Noeparvar, Søren Borg

**Affiliations:** ^1^Department of Molecular Biology and Genetics, Research Centre Flakkebjerg, Aarhus UniversitySlagelse, Denmark; ^2^Department of Plant and Environmental Sciences, University of CopenhagenCopenhagen, Denmark

**Keywords:** circular RNAs, coding and non-coding transcripts, leaves, seeds, transfer cells, micronutrients, mitochondria

## Abstract

RNA circularization made by head-to-tail back-splicing events is involved in the regulation of gene expression from transcriptional to post-translational levels. By exploiting RNA-Seq data and down-stream analysis, we shed light on the importance of circular RNAs in plants. The results introduce circular RNAs as novel interactors in the regulation of gene expression in plants and imply the comprehensiveness of this regulatory pathway by identifying circular RNAs for a diverse set of genes. These genes are involved in several aspects of cellular metabolism as hormonal signaling, intracellular protein sorting, carbohydrate metabolism and cell-wall biogenesis, respiration, amino acid biosynthesis, transcription and translation, and protein ubiquitination. Additionally, these parental loci of circular RNAs, from both nuclear and mitochondrial genomes, encode for different transcript classes including protein coding transcripts, microRNA, rRNA, and long non-coding/microprotein coding RNAs. The results shed light on the mitochondrial exonic circular RNAs and imply the importance of circular RNAs for regulation of mitochondrial genes. Importantly, we introduce circular RNAs in barley and elucidate their cellular-level alterations across tissues and in response to micronutrients iron and zinc. In further support of circular RNAs' functional roles in plants, we report several cases where fluctuations of circRNAs do not correlate with the levels of their parental-loci encoded linear transcripts.

## Introduction

Although the discovery of circular RNAs goes back to the late 70's (Hsu and Coca-Prados, 1979; Arnberg et al., 1980), there has been a surprising lack of attention paid to their function until recent years. Most recent contributions have revealed the regulated levels of circular RNAs implying the on-purpose production of circular RNAs in animal cells (Conn et al., 2015; You et al., 2015). In mammals, circular RNAs are involved in the positive regulation of gene expression at the transcriptional and post-transcriptional levels (Hansen et al., 2013; Memczak et al., 2013; Andreeva and Cooper, 2015; Li et al., 2015). Additionally, circular RNAs can suppress the expression of genes (Ashwal-Fluss et al., 2014). Based on these observations, circular RNAs can accomplish regulatory roles through different mechanisms by acting on miRNAs or proteins. As expected, the existence of circular RNAs is a general phenomenon across the eukaryotic tree of life (Lasda and Parker, 2014; Wang et al., 2014). Unfortunately, there is not much known about circular RNAs, their expressional profile, and annotated functions in plants. Very recent reports highlight the presence of circular RNAs in model plants rice and *Arabidopsis* (Wang et al., 2014; Lu et al., 2015; Ye et al., 2015). It has also been shown that circular RNAs can regulate the expression of their parental genes in rice (Lu et al., 2015). Here, we report circular RNAs from barley, present in leaves, grains and grain transfer cells. The study reveals their cellular-level variations across tissues and in response to micronutrients. The parental genes of identified circular RNAs are involved in a wide range of metabolic pathways and encode for different classes of transcripts. The results imply that the contribution of circular RNAs to the regulation of gene expression in plants is not confined to nuclear genes but also take place in organelles.

## Materials and methods

### Plant materials

To identify circular RNAs, we used our comprehensive barley RNA-Seq data with the Genbank accession number SRA297575. Field-grown barley plants at the growth stage 18 ± 2 days after anthesis were treated with a foliar application of iron (15 mM FeSO_4_.7H_2_O) or zinc (0.5% ZnSO_4_.7H_2_O) solution. Leaves and immature seeds were collected from three biological replicates of untreated plants and treated plants 6 and 24 h after the treatments.

### RNA sequencing

Laser capture microdissection was used to collect transfer cells for RNA isolation. Paired end (2 × 101 bases) sequencing was performed using HiSeq 2000 Illumina platform and Truseq technology. Due to the low-quantity sample (3.6–18 ng), linear amplification of RNAs was performed using NuGEN Ovation® RNA-Seq kit by which samples were enriched against ribosomal RNA using a mixture of semi-random and random primers. This gave us the chance to hunt for different RNA classes including non-poly(A) circular RNAs.

### Read mapping and circular RNA identification

The sequencing reads were trimmed for the first 20 and the last 6 nucleotides using Trimmomatic-0.33 (Bolger et al., 2014). In addition, a quality filtering was applied to both ends of the reads for nucleotides with quality scores lower than 22 as well as sliding trimming with a window size of 4 and an average quality score of 15 in Phred-33 scoring system. Employing a split mapping algorithm, BWA-MEM (bwa-0.7.12; Li, 2013) was used to map more than 514 million trimmed paired reads (2 × 76 bases) onto the barley genome release 26 available at EnsemblPlants (http://plants.ensembl.org/index.html). To avoid the process of read-trimming during mapping, we applied a large clipping penalty (-L 20000). The SAM file of alignment was inspected by the software CIRI v1.2 (Gao et al., 2015) to identify the paired reads supporting the junctions in circular RNA. The identified circular RNAs were further filtered out manually. Due to the possibility of contig mis-assembly, we first excluded the circular RNA candidates if the flanking regions of their junctions were mapped onto two different genomic contigs. Second, circular RNAs were not further considered if their junction regions were evidenced, i.e., the occurrence of back-splicing was rejected, by any publicly-available linear transcript or genomic sequence at NCBI. Recalling the functioning mechanisms of circular RNAs (Lasda and Parker, 2014; Andreeva and Cooper, 2015), repeated nucleotide motives can be of the utmost importance. We, therefore, inspected the identified circular RNAs for a maximum of two different nucleotide patterns with highest scores. We used the CLC software which creates a new Hidden Markov Model based on the selected sequence to find repeated nucleotide motives. We adjusted the pattern length varying from 4 to 25 bases.

### Real-time PCR

Three biological replicates from leaf and whole-seed samples were pooled and used for RNA extraction and subsequent cDNA preparation. The RNA samples were extracted using the FastRNA^TM^ Pro Green Kit. After DNase I (QIAGEN) treatment, RNA samples were again washed and precipitated. Additionally, RNaseR treated RNA samples were prepared to validate the circular RNAs through sample enrichment for circular RNAs vs. linear RNAs. Partial digestion of 25–50 μg DNase I-treated RNA samples, extracted from five different whole-seed and five different leaf samples, were performed by 20 units of RNaseR per reaction in 40 min. The random-primed first-strand cDNAs were synthesized using Superscript II (Invitrogen Life Technologies, Carlsbad, CA, USA) according to the manufacturer instruction with some modifications. Briefly, reactions containing 1–2 μg RNA and 2 μl reverse transcriptase II were carried out at 42°C for 50 min, followed at 46°C for 10 min. The reactions were terminated at 75°C for 15 min. To remove the RNA, RNaseH was added and incubated at 37°C for 20 min. Inactivation of RNaseH was performed at 65°C for 10 min followed by an on-ice cooling step. The ABI Applied Biosystems 7900 HT Thermal Cycler was used for real-time PCR (see Supplementary File 1 for primers). All cDNA samples were diluted three times to be used in real-time PCR. The cDNA sample (1 μl), primer pairs (5 μM each, 2 μl), SYBR® Green PCR Master Mix from ABI Applied Biosystems (5 μl), and nuclease-free water (1 μl) in combination were used in real-time PCR reactions. Real-time PCR reactions were performed in three technical replicates. The annealing temperature of PCR cycles was 64°C in all reactions and for different primer pairs. Convergent primers were used to target the reference genes *V-ATPase* and *Gadph* as well as the linear transcripts of the genes of interest. Divergent primers were also applied to target the circular RNAs in all undigested and digested RNA samples. The geometric average (Vandesompele et al., 2002) of two reference genes, barley *Gadph* and V-*ATPase* (MLOC_59475 and MLOC_18233; Darbani et al., 2015), was used to correct the expression data. To quantify the reference genes in RNaseR enrichment analysis, each reference gene was used to correct the other. The real-time PCR reactions were further analyzed for amplicon size using Agilent Bioanalyzer.

## Results and discussion

### Grain transfer cell-specific RNA sequencing and circular RNA identification

**Figure 1:**
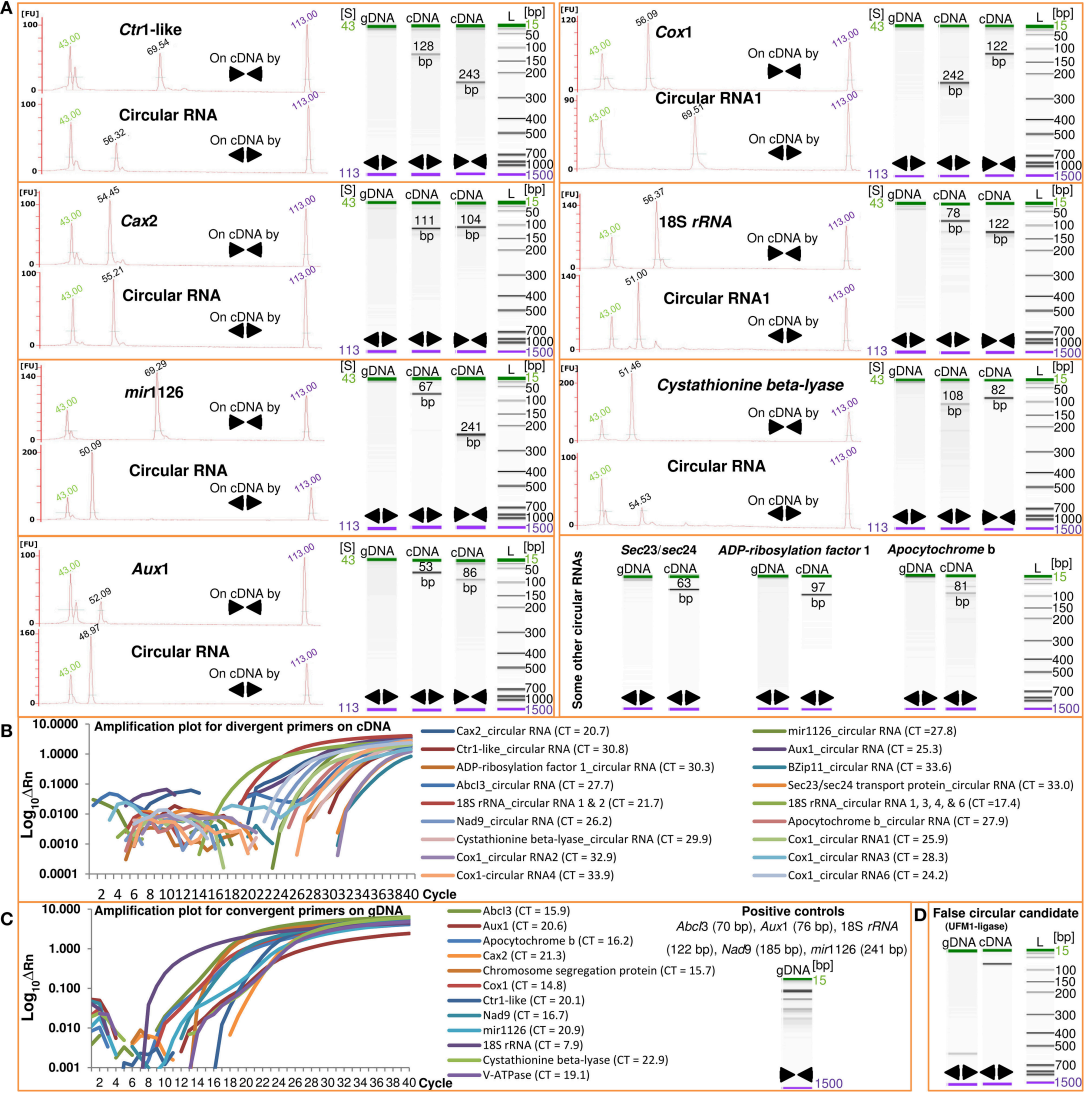
**Analysis of barley circular RNAs**. Real-time PCR using divergent (◄►) and convergent (►◄) primers and subsequent amplicon-size analysis on Agilent Bioanalyzer were used to confirm the identified barley circular RNAs. The absence of genomic DNA fragments corresponding to the junctions of 26 selected circular RNAs (**A** and Supplementary File 1) as well as the detection of 21 examined circular RNAs at transcript level (**B** and Supplementary File 1) validate the existence of circular RNAs in both barley leaves and seeds. The real-time PCR reactions were further analyzed by Agilent Bioanalyzer which confirmed the amplicons at the expected sizes shown as electropherograms and gel electrophoresis runs **(A)**. We also used the convergent primers of parental genes as positive controls for the genomic DNA **(C)**. The false positive circular RNA of the gene UFM1-ligase was included in the analysis as a negative control **(D)**. FU and S represent fluorescence absorbance and time of electrophoresis of the PCR reactions in Agilent Bioanalyzer. The electrophoresis time (in seconds) are shown on top of the electropherogram peaks as well as the lower and upper marker peaks. The labels of cDNA and gDNA indicate the type of template used in the real-time PCR reactions. Amplification plots for the divergent primers on gDNA indicating no amplification and for the divergent and convergent primers across all cDNA samples are shown in Supplementary File 1. See Table 1 for full name of the genes. Bp, base pair; CT, the cycle threshold of amplified fragments; ΔRn, the normalized fluorescence of the reporter dye minus baseline in real-time PCR; L, Ladder.

For the first time, we report fluctuations of circular RNAs across plant tissues and in response to foliar application of micronutrients. The software CIRI (Gao et al., 2015) was used to analyze RNA-Seq reads for existence of circular RNAs in barley. To exclude false positive candidates, we manually filtered out the circular RNA candidates with junction regions spanning over two genomic contigs or publicly-available linear nucleotide sequences. By analyzing 514.2 million paired-end reads (2 × 101 bases) representing the transcriptome of barley seed transfer cells, we identified 62 transfer cell-specific circular RNAs originated from 48 parental genes (Table 1). The sequence and isoforms of the circular RNAs and the structural relationship with their parental genes are shown in Supplementary File 1. To further validate the identified circular RNAs, we used divergent primers to amplify the junction regions of circular RNAs. Real-time PCR was applied to ensure the absence of genomic fragments that can encode for the circular RNA candidates. Divergent primer pairs designed for 26 selected circular RNAs from 18 different parental genes did not amplify any genomic fragment (Figure 1A and Supplementary File 1). As a negative control, divergent primers for the false positive circular RNA candidate of the gene UFM1- ligase worked as well as the convergent primers for the parental genes on genomic DNA (Figures 1C,D). The circular RNA of UFM1-ligase was not a true candidate revealed by finding of a linear transcript (GenBank: AK356330) with the junction region during the manual filtering process. This additionally highlights the importance of our manual filtering step to exclude linear transcripts that can be identified as circular RNA candidates due to possible mis-assemblies or repeated long nucleotide motives in the genome. In the same manner, close inspection of 262 identified circular RNA candidates through our manual filtering step (see the Methods) introduced 62 circular RNAs (Table 1).

**Table 1:** **Barley transfer cell-specific circular RNAs**.

**Circular RNAs^a^**	**Genomic location/parental gene^b^**	**Arabidopsis homolog^c^**
Kinesin-related protein 11-like_circular RNA	Ch1:128008822-128014221/MLOC_10504	AT4G39050
Internal spacer 2 of 18S,5.8S,26S rRNA_circular RNA1-2	Ch1:177434203-177434347; 1:429512475-429512569	–
Cytochrome c oxidase *Cox*1_circular RNA1-6	Ch1:23864318-238666057/MLOC_370	ATMG01360
Probable long non-coding RNA_circular RNA	Ch1:363958561-363959513	–
ADP-ribosylation factor 1_circular RNA	Ch1:389129363-389130325/MLOC_71884	AT1G10630
18S rRNA_circular RNA1-7	Ch1:429513196-429515439	AT3G41768
SAD1/UNC-84 domain protein 2_circular RNA	Ch1:60689841-60690191/MLOC_74926	AT3G10730
MicroRNA1126_circular RNA	Ch2:467982888-467983386	–
Unknown_circular RNA	Ch2:476367671-476368169	–
*Sec*23/*Sec*24 transport protein_circular RNA	Ch2:482080734-482081397/MLOC_37573	AT2G27460
Inositol transporter 2_circular RNA	Ch2:483514445-483514888/MLOC_38368	AT1G30220
ARID/BRIGHT DNA-binding domain protein_circular RNA	Ch2:532048153-532048410	AT2G17410
Alpha-mannosidase 1_circular RNA	Ch2:566491430-566492027/MLOC_75116	AT1G51590
Probable long non-coding RNA_circular RNA	Ch2:605441109-605441443	–
Unknown_circular RNA	Ch3:100501939-100502294	–
Mitogen-activated protein kinase (*Ctr*1-like)_circular RNA	Ch3:351423944-351425007/MLOC_56360	AT3G58640
Ubiquitin-conjugating enzyme 11_circular RNA	Ch3:473869516-473869731	AT3G08690
Auxin influx transporter *Aux*1_circular RNA	Ch3:486789367-486789848/MLOC_54960	AT2G38120
Laccase 12_circular RNA	Ch3:499962355-499962651/MLOC_19559	AT5G05390
Fumarase 2_circular RNA	Ch4:243159573-243159671/MLOC_36687	AT5G50950
Probable long non-coding RNA_circular RNA	Ch4:247058951-247059280	–
RNA-binding (RRM/RBD/RNP motifs) protein_circular RNA	Ch4:252670767-252671078/MLOC_74552	AT3G07810
Probable microtubule-stabilizing protein_circular RNA	Ch4:330511991-330512323/MLOC_11124	–
Vacuolar cation/proton exchanger *Cax*2_circular RNA	Ch4:356281355-356282249/MLOC_37140	AT3G13320
Formin-like protein 20_circular RNA1-2	Ch4:491530198-491530553/MLOC_81990	–
Probable beta-1-4-glucosyltransferase_circular RNA	Ch4:71095325-71097097/MLOC_44675	AT4G37420
Probable aminopeptidase_circular RNA	Ch5:286201637-286202872	AT3G19340
Probable chromosome segregation protein_circular RNA	Ch5:361207403-361207861	–
Transducin/WD40 repeat-like protein_circular RNA	Ch5:484562574-484563329/MLOC_58976	AT2G40360
Ribosomal protein L30/L7_circular RNA	Ch5:524054971-524055497/MLOC_17150	AT3G13580
Probable long non-coding RNA_circular RNA	Ch5:53926495-53926935	–
Unknown_circular RNA	Ch5:6888869-6889034	–
Abscisic acid-responsive protein_circular RNA	Ch6:20988426-20990992/MLOC_15028	AT5G42560
Ribosomal protein L6_circular RNA	Ch6:245896672-245897042/MLOC_63134	AT1G74050
Far upstream element-binding protein 2_circular RNA	Ch6:245908278-245908734/MLOC_60294	AT2G25970
RNA-binding (RRM/RBD/RNP motifs) protein_circular RNA	Ch6:260427394-260427518/MLOC_7493	AT3G13224
Glycyl-tRNA synthetase 2_circular RNA1-2	Ch6:268555508-268556362/MLOC_63502	AT3G48110
RNA-binding (RRM/RBD/RNP motifs) protein_circular RNA	Ch6:308455217-308455370/MLOC_68712	AT3G04500
*BZip*11_circular RNA	Ch6:52088926-52089401/MLOC_63436	AT4G34590
Ubiquitin-specific protease 17_circular RNA	Ch7:307483097-307485269	AT5G65450
Unknown_circular RNA	Ch7:4237959-4238849	–
Probable KH domain-containing splicing factor_circular RNA	Ch7:457516955-457517152/MLOC_10055	AT3G32940
Cystathionine beta-lyase_circular RNA	Ch7:53946083-53946582/MLOC_71910	AT3G57050
Sec-independent protein translocase_circular RNA	Morex_contig_106453:2137-3175	ATMG00570
ATP-binding cassette *Abc*I3_circular RNA	Morex_contig_1661226:956-1173/MLOC_24918	ATMG00900
Apocytochrome b_circular RNA	Morex_contig_42365:12844-13477MLOC_58118	ATMG00220
NADH Dehydrogenase *Nad*9_circular RNA	Morex_contig_70567:2809-3204/MLOC_76215	ATMG00070

a See Supplementary File 1 for sequences;

b Available online at http://plants.ensembl.org/index.html;

c Available online at https://www.arabidopsis.org/

### Occurrence of the identified transfer-cell circular events in leaves and grains

**Figure 2:**
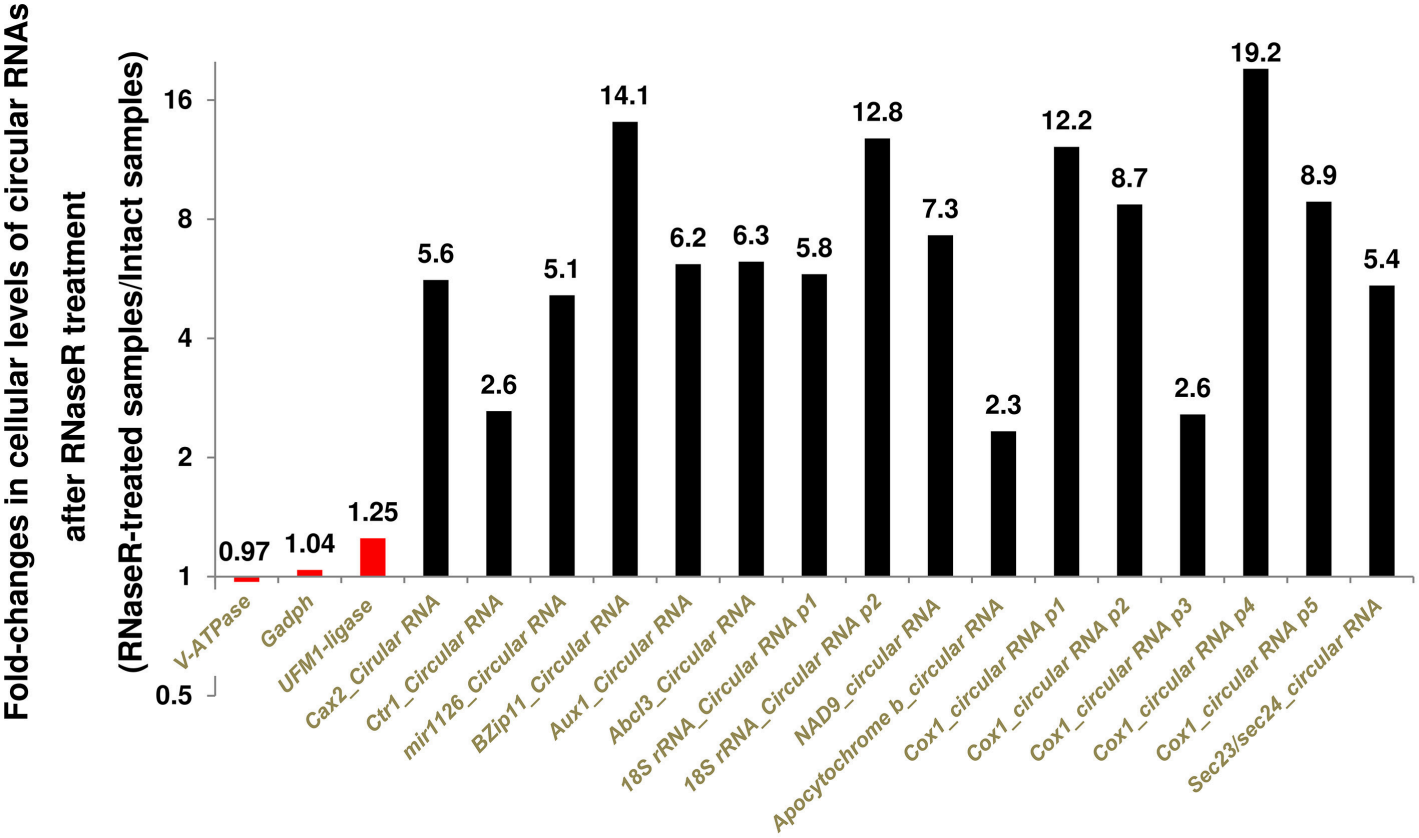
**Digestion of Linear RNAs by RNaseR enriched the circular RNAs up to 18 times**. The averages of 30 corrected measurements (10 samples with 3 technical replicates) both before and after digestion were applied to examine the RNaseR-resistance of amplicons. In addition to the reference genes, we used UFM1-protein ligase as a negative control. It was a false positive circular RNA candidate supported by a linear transcript with Genbank accession number AK356330. Different divergent primers targeting circular RNA isoforms are shown as p1 to p5. See Table 1 for the gene names.

We were also able to detect the identified seed transfer cell circular RNAs in RNA samples from whole seeds as well as leaves (Figure 1B and Supplementary File 1). Further validation came from partial digestion of linear RNAs. Because circular RNAs are RNaseR resistant, treatment with RNaseR enriches for circular RNAs in contrast to linear RNAs. Targeting different circular RNAs using divergent primers revealed a 130–1820% increase in the levels of circular RNAs after partial digestion with RNaseR (Figure 2). As the negative controls, convergent primers of *Gadph* (corrected by the reference gene V-*ATPase*) and convergent primers of V-*ATPase* (corrected by the reference gene *Gadph*) showed only 4% increase and reduction at transcript levels, respectively (Figure 2). Analyzing the false positive circular RNA candidate of UFM1-ligase also revealed no more than 25% increase after digestion with RNaseR (Figure 2).

### Cellular-level variations of circular RNAs across tissues and in response to micronutrients

**Figure 3:**
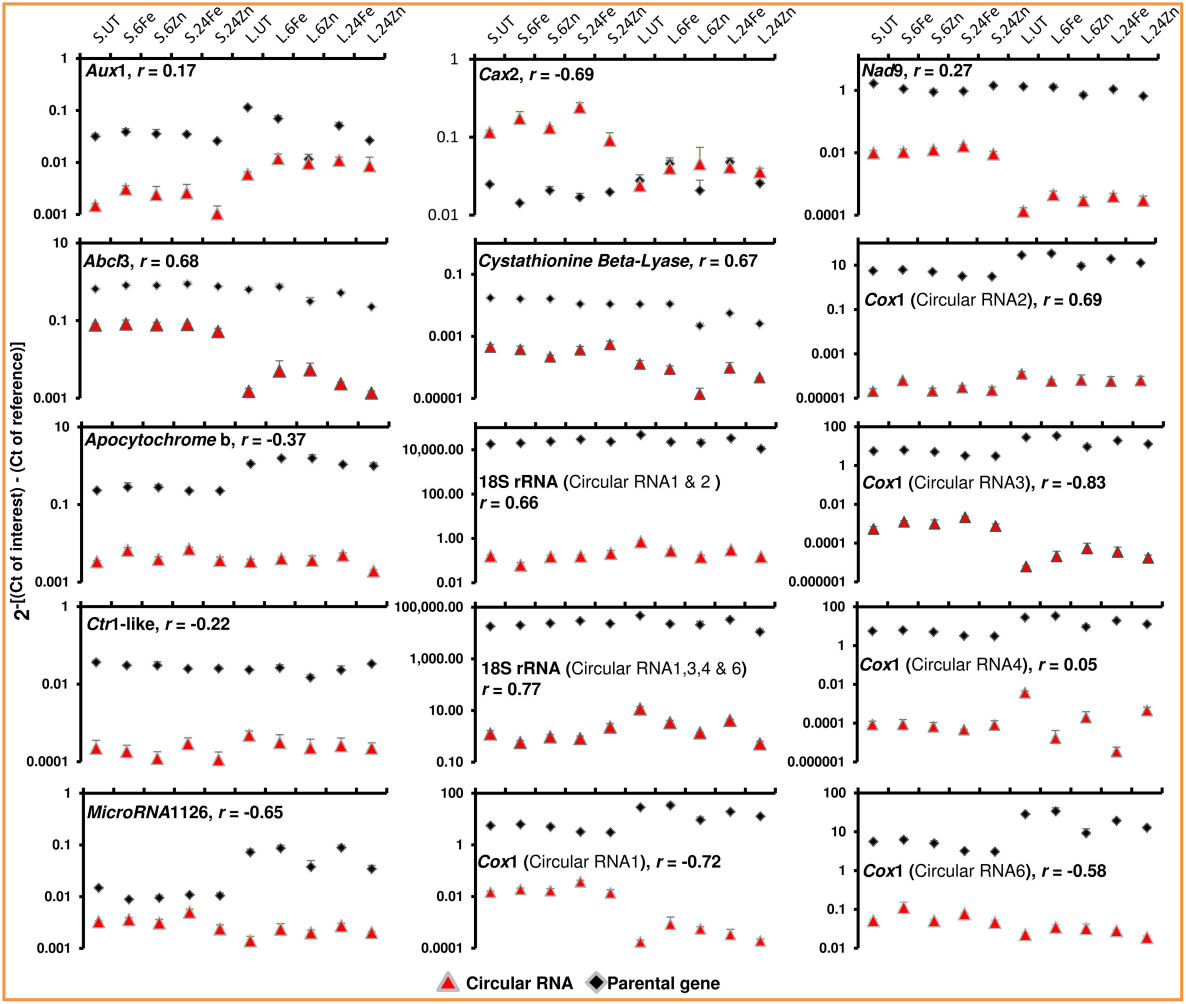
**The cellular levels of circular RNAs differ across tissues and in response to micronutrients and show in general no strong correlation with the linear-transcript expression of their parental genes**. Real-time PCR was applied to target the circular RNA junctions and the parental genes using divergent and convergent primer pairs, respectively. Error bars represent the standard deviations for three technical replicates. Three biological replicates for each sample were pooled in the experiment. Seed and leaf samples are shown as S and L. UT, Fe, and Zn are for untreated, iron-treated, and zinc-treated plants. The samples of 6 and 24 h after treatments are represented by 6 and 24. The Pearson correlations (*r*) between circular RNAs and their parental genes were measured on log_2_ transformed data. See Table 1 for full name of the genes. CT, the cycle threshold of amplified fragments.

Here, we demonstrate that the levels of circular RNAs vary across tissues and treatments (Figure 3). The divergent primers, designed for the junction regions, were used in real-time PCR to examine the fluctuations in cellular levels of circular RNAs across leaves and seeds both before and after foliar application of the micronutrients iron and zinc. We found opposite expression trends between *Cax*2 circular RNA and *Cax*2 mRNA when comparing seed samples with leaf samples (Figure 3). CAX2 is an H^+^/cation antiporter and sequesters calcium and other cations like zinc and iron within vacuoles (Darbani et al., 2013). The opposite expression trends may indicate a difference in calcium and heavy metal vacuolar-storage behavior between leaves and seeds. In agreement, the expression of *Cax*2 was repressed in seeds by the foliar application of micronutrients and in particular by iron which in leaves triggered the expression of *Cax*2 (Figure 3). In general, we found weak and negative correlations between cellular levels of circular RNAs and the linear-transcript levels of their parental genes (Figure 3). The weak and negative correlations rule out any speculation about recognizing the circular RNAs as transcriptional or post-transcriptional artifacts. Furthermore, the different and opposite cellular level variations among circular RNAs as well as among their isoforms disagree with the possible contribution of linear RNA-specific and circular RNA-specific degradation mechanisms to the observed absence of correlation. Recalling the regulatory actions of circular RNAs on gene expression as well as their regulated cellular levels in animals, we found the barley circular RNAs responding to foliar application of micronutrients in both leaves and seeds (Figure 3). They not only behaved differently when comparing the treatments with micronutrients iron and zinc, but also when comparing leaf and seed tissues (Figure 3). We additionally found different cellular levels among the circular RNA isoforms of *Cox*1 across tissues and treatments (Figure 3). Whether they play different roles or not, has yet to be revealed. We noticed circular RNAs for the genes that are known to be involved in zinc and iron homeostasis; the circular RNAs of calcium/zinc/iron homeostasis components including ATP-binding cassette *AbcI*3 and *Cax*2 (Darbani et al., 2013) responded to the foliar applications of iron and zinc (Figure 3). We have previously reported the interaction between micronutrient homeostasis and respiration and the involvement of auxin and ethylene signaling as well as intracellular protein sorting mechanisms in iron and zinc homeostasis (Darbani et al., 2013, 2015). Involved in the mentioned cellular functions, we accordingly found genes with circular RNAs regulated by the iron and zinc foliar applications (Figure 3 and Supplementary File 1). This includes the genes coding for the respiratory components cytochrome c oxidase subunit COX1, apocytochrome b, and NADH dehydrogenase NAD9, the auxin and ethylene signaling factors of auxin influx transporter AUX1 and CTR1-like serine/threonine-protein kinase, and the intracellular trafficking components of SEC23/SEC24 and ADP-ribosylation factor 1. Additionally, we found the parental genes responding to the foliar applications (Figure 3). Taken together, the results suggest that circular RNAs may have a regulatory role in micronutrient homeostasis which can be exploited in crop biotechnology for iron and zinc biofortification.

### The possible function of circular RNAs and their involvement in mitochondrial functionality

As already mentioned, the inter-macromolecular interactions are the basis for function of circular RNAs. The repeated nucleotide motives can therefore play critical roles. Although we could not find highly pronounced microRNA target sites as reported by Hansen et al. (2013), patterns with 8–22 nucleotides in length were however identified 2 to 13 times along the circular RNAs (Supplementary File 1). Further experiments are still needed to unravel their contribution. A fascinating discovery in our study was the identification of microRNA and putative long non-coding/microprotein coding RNAs as the transcripts of origin for circular RNAs (Table 1). We examined the microRNA1126 and it's identified circular RNA and unraveled their regulated cellular levels (Figure 3 and Supplementary File 1). It is worth mentioning that plant microRNAs encode microproteins to regulate their own transcription (Lauressergues et al., 2015). Hence, there might be a general self-induced regulatory network where microproteins and circular RNAs contribute spatiotemporally to fine-tune the action of microRNAs. The results also provide primary insights into the mitochondrion dependency on circular RNAs in plants. Most interestingly, we found exonic circular RNAs for mitochondrial genes encoding COX1, apocytochrome b, NAD9, ABCI3, and sec-independent protein translocase (Table 1 and Supplementary File 1). Therefore, the functioning of circular RNAs is most likely a common mechanism exploited also by the plant mitochondrion. In agreement, the circular RNAs of the genes *Cox*1, *Nad*9, apocytochrome b, and *Abc*I3 showed regulated cellular levels across tissues and in response to the micronutrients (Figure 3 and Supplementary File 1). As respiratory engines, mitochondria are very dependent on micronutrients (Darbani et al., 2013, 2015).

Considering the known regulatory roles of circular RNAs in gene expression and the functional diversity of the parental genes for the identified plant circular RNAs (see Table 1), our results argue for the existence of an additional regulatory mechanism by which plant cells govern their nuclear and organellar gene expression through exploiting circular RNAs. By this report we finally open a new research area to further expand the plant circular RNA archive and study their role in detail as well as their potential use in applied crop biotechnology.

## Author contributions

BD conceived and designed the study. BD and SN performed the experiments, analyzed the data, and wrote the manuscript. SB supervised the study, discussed the results, and wrote the manuscript. All authors approved the final manuscript.

## Funding

The study was financially supported by HarvestPlus Programme, agreement number: 2014H6315.AAR and PhD stipend from Aarhus University GSST PhD school.

### Conflict of interest statement

The authors declare that the research was conducted in the absence of any commercial or financial relationships that could be construed as a potential conflict of interest.
