# Supplementary material for: Identification of Circular RNAs from the Parental Genes Involved in Multiple Aspects of Cellular Metabolism in Barley
Source: Front Plant Sci. 2016 Jun 3;7:776. doi: 10.3389/fpls.2016.00776 (PMC4891351; doi:10.3389/fpls.2016.00776)
Supplement: Supplementary file 1 [file DataSheet1.PDF]

Suppl. File 1

Identification of Circular RNAs From the Parental Genes Involved in Multiple Aspects of Cellular Metabolism in Barley

Behrooz Darbani[1,2§]*, Shahin Noeparvar[1§], Søren Borg[1]*

[1] Department of Molecular Biology and Genetics, Research Centre Flakkebjerg, Aarhus University, Denmark

[2] Department of Plant and Environmental Sciences, University of Copenhagen, Denmark

This supplementary file contains the following information:

I. Expression profile of the circular RNAs

II. Circular RNA sequences, their structural relationship with the parental genes, and the amplification plots of real-time PCR reactions

III. The identified nucleotide patterns in circular RNAs

IV. Primer sequences

Part I

Expression profile of the circular RNAs. Divergent primers were used to target the junction region of circular RNAs by real-time PCR. The geometric average of barley *Gadph* and V-*ATPase* (MLOC_59475 and MLOC_18233; see Darbani et al., 2015) was used to correct the expression data. Error bars represent the standard deviations for three technical replicates. Three biological replicates for each sample were pooled in the experiment. Seed and leaf samples are shown as S and L. UT, Fe, and Zn are for untreated, iron-treated, and zinc-treated plants. The samples of 6 h and 24 h after treatments are represented by 6 and 24. See Table 1 for full name of the genes.

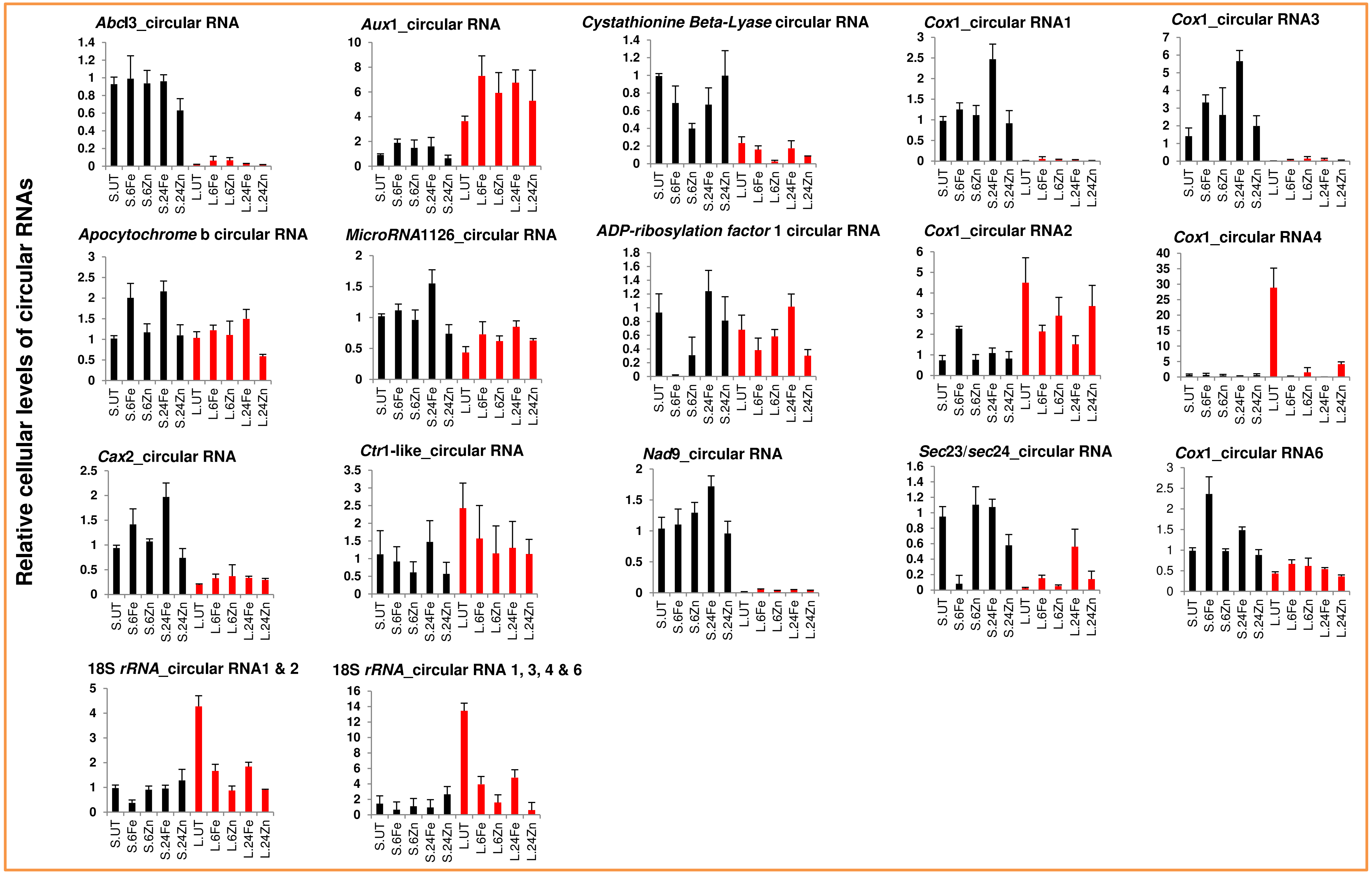

Part II

Vacuolar cation/proton exchanger Cax2_circular RNA (ID: Ch4:356281355-356282249**)**

GAAGAAAGCACCGAGGATGAGGAGGATGAAAAGGAGATAACACAGGGCGAAGCTATCTCCT
GGCTTTTTGTATTGACTATTTGGATTTCAATTCTCTCTGGGTATCTGGTAGATGCCATACAGGTAA
CGAACTGTTTACATCAGGATACAAATATAATCGCTATTTTACATCAATTCATTATCTCCTGCTCGA
ACATGCCACTGGCCTTTGTAACAGGGGGCCTCTGAATCATTAAACATGCCACTGGCCTTTATTAG
TGTTATTCTGCTTCCTATCGTGGGGAATGCTGCTGAGCATGCAAGCGCCATTATGTTTGCCATGA
GAAACAAACTTGTAAGCAGCCCCTGCTTTCTGCAACTCAATTGTGTGTTTCTGTTCTCATCTTTAA
TTTCTTGCATGCCATAAGGACATTACATTGGGTGTCGCCATAGGGTCGTCAACACAGATATCTAT
GTTTGTANNNNNNNNNNNNNNNNNNNNNNNNNNNNNNNNNNNNNNNNNNNNNNNNNNNNN
NNNNNNNNNNNNNNNNNNNNNNNNNNNNNNNNNNNNNNNNNNNNNNNNNNATTCCATTCTGTGT
GGTAATTGGCTGGATGATGGGGGAAGAGATGGACTTGAACTTTCAATTGTTTGAGACAGCAACT
CTTTTTATAACAGTACTTGTGGTGGCATTTATGCTACAGGAAGGCACGTCAAACTATTTTAAAGG
CCTTATGCTCATATTATGTTATCTCATAG**TTGCCGCAAGCTTTTTTGTGCACGTTGATCCCAAAT
CAA**

The nucleotides of junction-region are underlined. The nucleotides of junction-region which are supported by the junction-spaning sequencing reads are shown in red. Introns are not shown if the absence is supported by sequencing reads. In the absence of supporting sequencing reads, the intronic nucletides are shown as N.

Structural relationship between the circular RNA and its parental gene

```
                                        20                  40                  60                  80
Genomic(MLOC_37140)  AAAACGCTTTCCAAACAGTTCAGGTTCTTTTAACTAGTCATGTAACCATTTGCTGTTTCTCAAGTTCACGTGAGGCACAA 80
cDNA1(MLOC_37140)    AAAACGCTTTCCAAACAGTTCAGGTTCTTTTAACTAGTCATGTAACCATTTGCTGTTTCTCAAGTTCACGTGAGGCACAA 80
cDNA2(MLOC_37140     -------------------------------------------------------------------------------- -
cDNA3(MLOC_37140     -------------------------------------------------------------------------------- -
CircularRNA          -------------------------------------------------------------------------------- -
Cir_Forward.Primer   -------------------------------------------------------------------------------- -
Cir_Rreverse.Primer  -------------------------------------------------------------------------------- -

                                       100                 120                 140                 160
Genomic(MLOC_37140)  AGGAACAATTGCCTATCAAAACAAACCAGAGAAAATCTGGACTTAAATTTGTGCACAGTTCCTTCGAATCCTAGTTTACC 160
cDNA1(MLOC_37140)    AGGAACAATTGCCTATCAAAACAAACCAGAGAAAATCTGGACTTAAATTTGTGCACAGTTCCTTCGAATCCTAGTTTACC 160
cDNA2(MLOC_37140     -------------------------------------------------------------------------------- -
cDNA3(MLOC_37140     -------------------------------------------------------------------------------- -
CircularRNA          -------------------------------------------------------------------------------- -
Cir_Forward.Primer   -------------------------------------------------------------------------------- -
Cir_Rreverse.Primer  -------------------------------------------------------------------------------- -

                                       180                 200                 220                 240
Genomic(MLOC_37140)  TACTTGCCATAACAGGAAGAAAGCACCGAGGATGAGGAGGATGAAAAGGAGATAACACAGGGCGAAGCTATCTCCTGGCT 240
cDNA1(MLOC_37140)    TACTTGCCATAACAGGAAGAAAGCACCGAGGATGAGGAGGATGAAAAGGAGATAACACAGGGCGAAGCTATCTCCTGGCT 240
cDNA2(MLOC_37140     ------------CAGGAAGAAAGCACCGAGGATGAGGAGGATGAAAAGGAGATAACACAGGGCGAAGCTATCTCCTGGCT 68
cDNA3(MLOC_37140     -----------------------------------GGAGGATGAAAAGGAGATAACACAGGGCGAAGCTATCTCCTGGCT 45
CircularRNA          ---------------GAAGAAAGCACCGAGGATGAGGAGGATGAAAAGGAGATAACACAGGGCGAAGCTATCTCCTGGCT 65
Cir_Forward.Primer   -------------------------------------------------------------------------------- -
Cir_Rreverse.Primer  -------------------------------ATGAGGAGGATGAAAAGGAGATAACA----------------------- 26

                                       260                 280                 300                 320
Genomic(MLOC_37140)  TTTTGTATTGACTATTTGGATTTCAATTCTCTCTGGGTATCTGGTAGATGCCATACAGGTAACGAACTGTTTACATCAGG 320
cDNA1(MLOC_37140)    TTTTGTATTGACTATTTGGATTTCAATTCTCTCTGGGTATCTGGTAGATGCCATACAGG--------------------- 299
cDNA2(MLOC_37140     TTTTGTATTGACTATTTGGATTTCAATTCTCTCTGGGTATCTGGTAGATGCCATACAGG--------------------- 127
cDNA3(MLOC_37140     TTTTGTATTGACTATTTGGATTTCAATTCTCTCTGGGTATCTGGTAGATGCCATACAGGTAACGAACTGTTTACATCAGG 125
CircularRNA          TTTTGTATTGACTATTTGGATTTCAATTCTCTCTGGGTATCTGGTAGATGCCATACAGGTAACGAACTGTTTACATCAGG 145
Cir_Forward.Primer   -------------------------------------------------------------------------------- -
Cir_Rreverse.Primer  -------------------------------------------------------------------------------- 26

                                       340                 360                 380                 400
Genomic(MLOC_37140)  ATACAAATATAATCGCTATTTTACATCAATTCATTATCTCCTGCTCGAACATGCCACTGGCCTTTGTAACAGGGGGCCTC 400
cDNA1(MLOC_37140)    -------------------------------------------------------------------------GGGCCTC 306
cDNA2(MLOC_37140     -------------------------------------------------------------------------GGGCCTC 134
cDNA3(MLOC_37140     ATACAAATATAATCGCTATTTTACATCAATTCATTATCTCCTGCTCGAACATGCCACTGGCCTTTGTAACAGGGGGCCTC 205
CircularRNA          ATACAAATATAATCGCTATTTTACATCAATTCATTATCTCCTGCTCGAACATGCCACTGGCCTTTGTAACAGGGGGCCTC 225
Cir_Forward.Primer   -------------------------------------------------------------------------------- -
Cir_Rreverse.Primer  -------------------------------------------------------------------------------- 26

                                       420                 440                 460                 480
Genomic(MLOC_37140)  TGAATCATTAAACATGCCACTGGCCTTTATTAGTGTTATTCTGCTTCCTATCGTGGGGAATGCTGCTGAGCATGCAAGCG 480
cDNA1(MLOC_37140)    TGAATCATTAAACATGCCACTGGCCTTTATTAGTGTTATTCTGCTTCCTATCGTGGGGAATGCTGCTGAGCATGCAAGCG 386
cDNA2(MLOC_37140     TGAATCATTAAACATGCCACTGGCCTTTATTAGTGTTATTCTGCTTCCTATCGTGGGGAATGCTGCTGAGCATGCAAGCG 214
cDNA3(MLOC_37140     TGAATCATTAAACATGCCACTGGCCTTTATTAGTGTTATTCTGCTTCCTATCGTGGGGAATGCTGCTGAGCATGCAAGCG 285
CircularRNA          TGAATCATTAAACATGCCACTGGCCTTTATTAGTGTTATTCTGCTTCCTATCGTGGGGAATGCTGCTGAGCATGCAAGCG 305
Cir_Forward.Primer   -------------------------------------------------------------------------------- -
Cir_Rreverse.Primer  -------------------------------------------------------------------------------- 26

                                       500                 520                 540                 560
Genomic(MLOC_37140)  CCATTATGTTTGCCATGAGAAACAAACTTGTAAGCAGCCCCTGCTTTCTGCAACTCAATTGTGTGTTTCTGTTCTCATCT 560
cDNA1(MLOC_37140)    CCATTATGTTTGCCATGAGAAACAAACTTG-------------------------------------------------- 416
cDNA2(MLOC_37140     CCATTATGTTTGCCATGAGAAACAAACTTGTAAGCAGCCCCTGCTTTCTGCAACTCAATTGTGTGTTTCTGTTCTCATCT 294
cDNA3(MLOC_37140     CCATTATGTTTGCCATGAGAAACAAACTTG-------------------------------------------------- 315
CircularRNA          CCATTATGTTTGCCATGAGAAACAAACTTGTAAGCAGCCCCTGCTTTCTGCAACTCAATTGTGTGTTTCTGTTCTCATCT 385
Cir_Forward.Primer   -------------------------------------------------------------------------------- -
Cir_Rreverse.Primer  -------------------------------------------------------------------------------- 26

                                       580                 600                 620                 640
Genomic(MLOC_37140)  TTAATTTCTTGCATGCCATAAGGACATTACATTGGGTGTCGCCATAGGGTCGTCAACACAGATATCTATGTTTGTAGTAA 640
cDNA1(MLOC_37140)    -----------------------ACATTACATTGGGTGTCGCCATAGGGTCGTCAACACAGATATCTATGTTTGTA---- 469
cDNA2(MLOC_37140     TTAATTTCTTGCATGCCATAAGGACATTACATTGGGTGTCGCCATAGGGTCGTCAACACAGATATCTATGTTTGTA---- 370
cDNA3(MLOC_37140     -----------------------ACATTACATTGGGTGTCGCCATAGGGTCGTCAACACAGATATCTATGTTTGTA---- 368
CircularRNA          TTAATTTCTTGCATGCCATAAGGACATTACATTGGGTGTCGCCATAGGGTCGTCAACACAGATATCTATGTTTGTANNNN 465
Cir_Forward.Primer   -------------------------------------------------------------------------------- -
Cir_Rreverse.Primer  -------------------------------------------------------------------------------- 26

                                       660                 680                 700                 720
Genomic(MLOC_37140)  GTTCATTTACTCAACGAGATTCAACTTTCGTCCTGTACATTTTCATTTTTTTGCAAGGTTAAACAATTGCAACCAACACC 720
cDNA1(MLOC_37140)    -------------------------------------------------------------------------------- 469
cDNA2(MLOC_37140     -------------------------------------------------------------------------------- 370
cDNA3(MLOC_37140     -------------------------------------------------------------------------------- 368
CircularRNA          NNNNNNNNNNNNNNNNNNNNNNNNNNNNNNNNNNNNNNNNNNNNNNNNNNNNNNNNNNNNNNNNNNNNNNNNNNNNNNNN 545
Cir_Forward.Primer   -------------------------------------------------------------------------------- -
Cir_Rreverse.Primer  -------------------------------------------------------------------------------- 26

                                       740                 760                 780                 800
Genomic(MLOC_37140)  CACGTTGCATGATTTGCAGATTCCATTCTGTGTGGTAATTGGCTGGATGATGGGGGAAGAGATGGACTTGAACTTTCAAT 800
cDNA1(MLOC_37140)    -------------------ATTCCATTCTGTGTGGTAATTGGCTGGATGATGGGGGAAGAGATGGACTTGAACTTTCAAT 530
cDNA2(MLOC_37140     -------------------ATTCCATTCTGTGTGGTAATTGGCTGGATGATGGGGGAAGAGATGGACTTGAACTTTCAAT 431
cDNA3(MLOC_37140     -------------------ATTCCATTCTGTGTGGTAATTGGCTGGATGATGGGGGAAGAGATGGACTTGAACTTTCAAT 429
CircularRNA          NNNNNNNNNNNNNNNNNNNATTCCATTCTGTGTGGTAATTGGCTGGATGATGGGGGAAGAGATGGACTTGAACTTTCAAT 625
Cir_Forward.Primer   -------------------------------------------------------------------------------- -
Cir_Rreverse.Primer  -------------------------------------------------------------------------------- 26
```

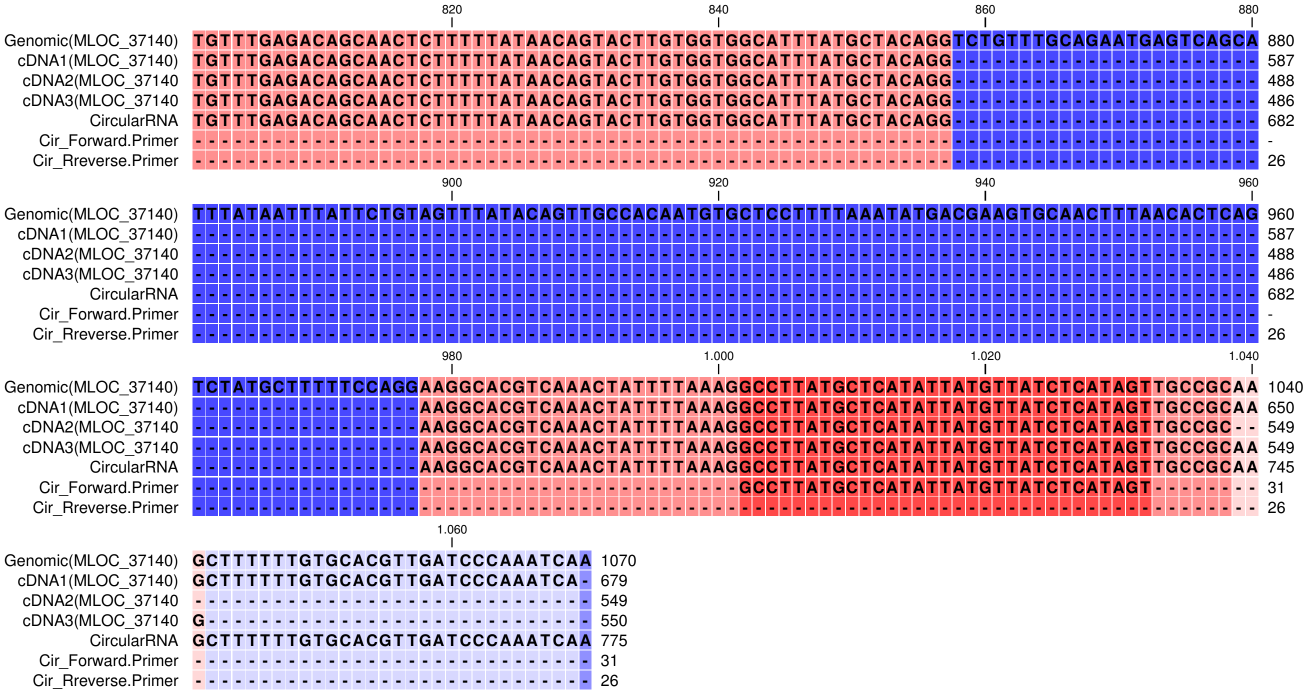

820 840 860 880
Genomic(MLOC_37140) TGTTTGAGACAGCAACTCTTTTTATAACAGTACTTGTGGTGGCATTTATGCTACAGGTCTGTTTGCAGAATGAGTCAGCA 880
cDNA1(MLOC_37140) TGTTTGAGACAGCAACTCTTTTTATAACAGTACTTGTGGTGGCATTTATGCTACAGG----------------------- 587
cDNA2(MLOC_37140 TGTTTGAGACAGCAACTCTTTTTATAACAGTACTTGTGGTGGCATTTATGCTACAGG----------------------- 488
cDNA3(MLOC_37140 TGTTTGAGACAGCAACTCTTTTTATAACAGTACTTGTGGTGGCATTTATGCTACAGG----------------------- 486
CircularRNA TGTTTGAGACAGCAACTCTTTTTATAACAGTACTTGTGGTGGCATTTATGCTACAGG----------------------- 682
Cir_Forward.Primer -- -
Cir_Rreverse.Primer -- 26
900 920 940 960
Genomic(MLOC_37140) TTTATAATTTATTCTGTAGTTTATACAGTTGCCACAATGTGCTCCTTTTAAATATGACGAAGTGCAACTTTAACACTCAG 960
cDNA1(MLOC_37140) -- 587
cDNA2(MLOC_37140 -- 488
cDNA3(MLOC_37140 -- 486
CircularRNA -- 682
Cir_Forward.Primer -- -
Cir_Rreverse.Primer -- 26
980 1.000 1.020 1.040
Genomic(MLOC_37140) TCTATGCTTTTTCCAGGAAGGCACGTCAAACTATTTTAAAGGCCTTATGCTCATATTATGTTATCTCATAGTTGCCGCAA 1040
cDNA1(MLOC_37140) -----------------AAGGCACGTCAAACTATTTTAAAGGCCTTATGCTCATATTATGTTATCTCATAGTTGCCGCAA 650
cDNA2(MLOC_37140 -----------------AAGGCACGTCAAACTATTTTAAAGGCCTTATGCTCATATTATGTTATCTCATAGTTGCCGC-- 549
cDNA3(MLOC_37140 -----------------AAGGCACGTCAAACTATTTTAAAGGCCTTATGCTCATATTATGTTATCTCATAGTTGCCGCAA 549
CircularRNA -----------------AAGGCACGTCAAACTATTTTAAAGGCCTTATGCTCATATTATGTTATCTCATAGTTGCCGCAA 745
Cir_Forward.Primer ---GCCTTATGCTCATATTATGTTATCTCATAGT-------- 31
Cir_Rreverse.Primer -- 26
1.060
Genomic(MLOC_37140) GCTTTTTTGTGCACGTTGATCCCAAATCAA 1070
cDNA1(MLOC_37140) GCTTTTTTGTGCACGTTGATCCCAAATCA- 679
cDNA2(MLOC_37140 ------------------------------ 549
cDNA3(MLOC_37140 G----------------------------- 550
CircularRNA GCTTTTTTGTGCACGTTGATCCCAAATCAA 775
Cir_Forward.Primer ------------------------------ 31
Cir_Rreverse.Primer ------------------------------ 26

Real-Time PCR for the junction region of vacuolar cation/proton exchanger Cax2_circular RNA (ID: Ch4:356281355-356282249)

By divergent primers on genomic DNA

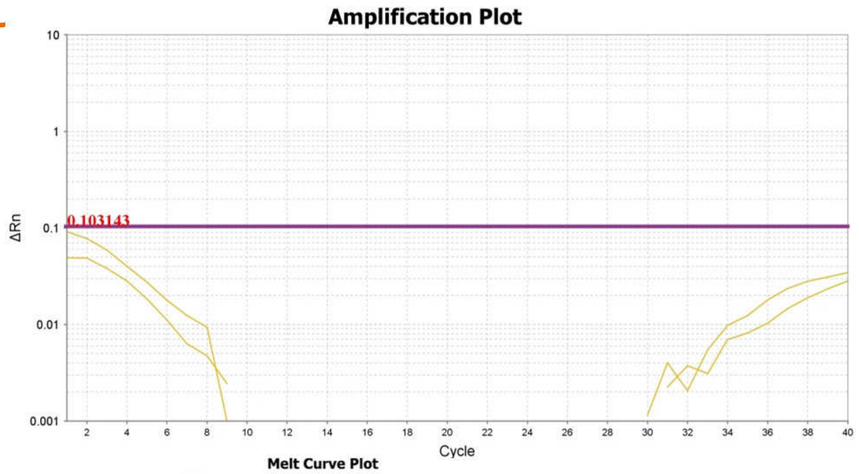

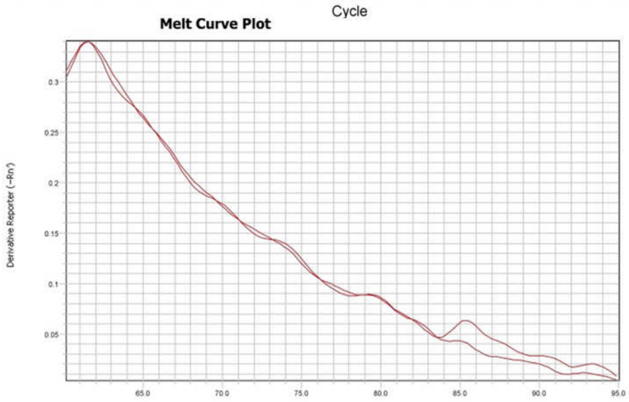

By divergent primers on cDNAs

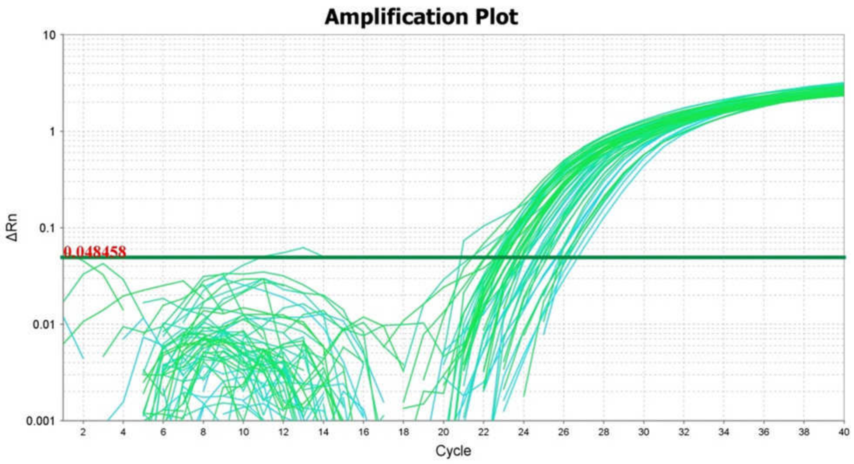

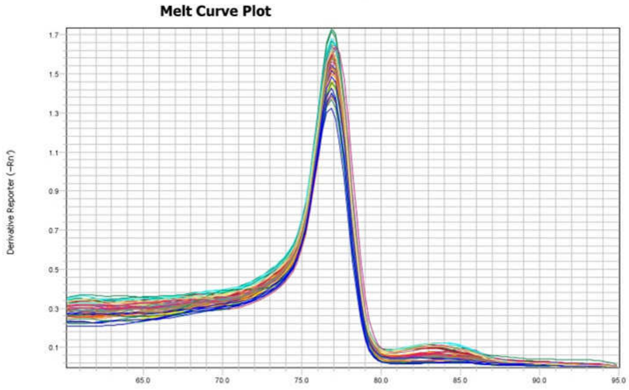

Real-Time PCR for the junction region of vacuolar cation/proton exchanger Cax2_circular RNA (ID: Ch4:356281355-356282249**)**

By divergent primers ◀▶ & with no template

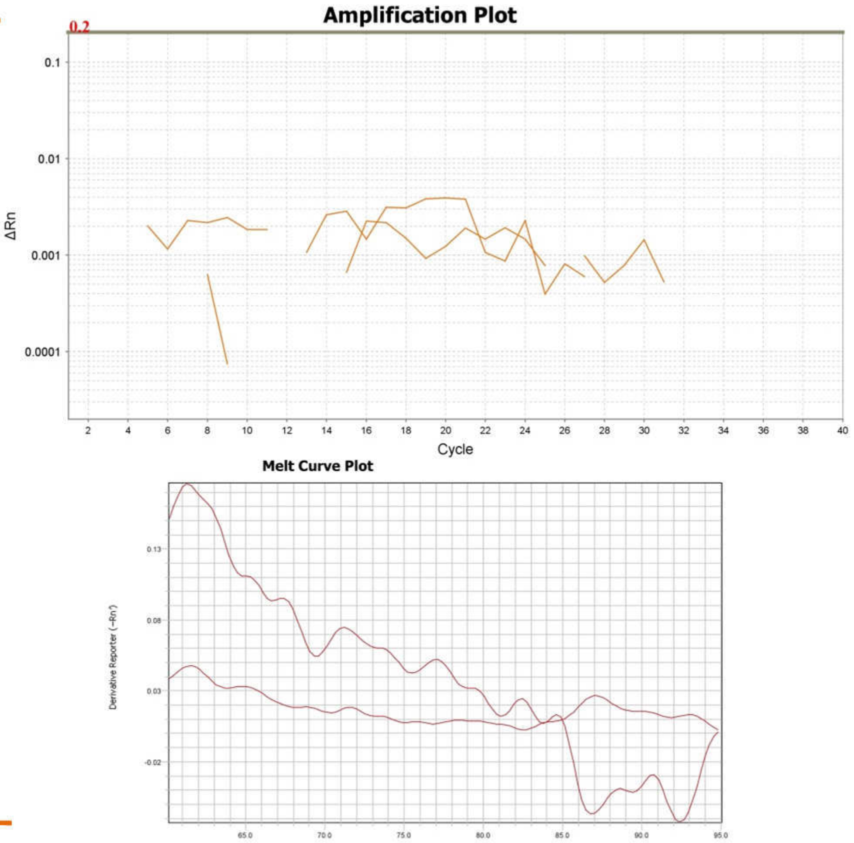

Real-Time PCR for the vacuolar cation/proton exchanger Cax2

By convergent primers ▶◀ on cDNAs

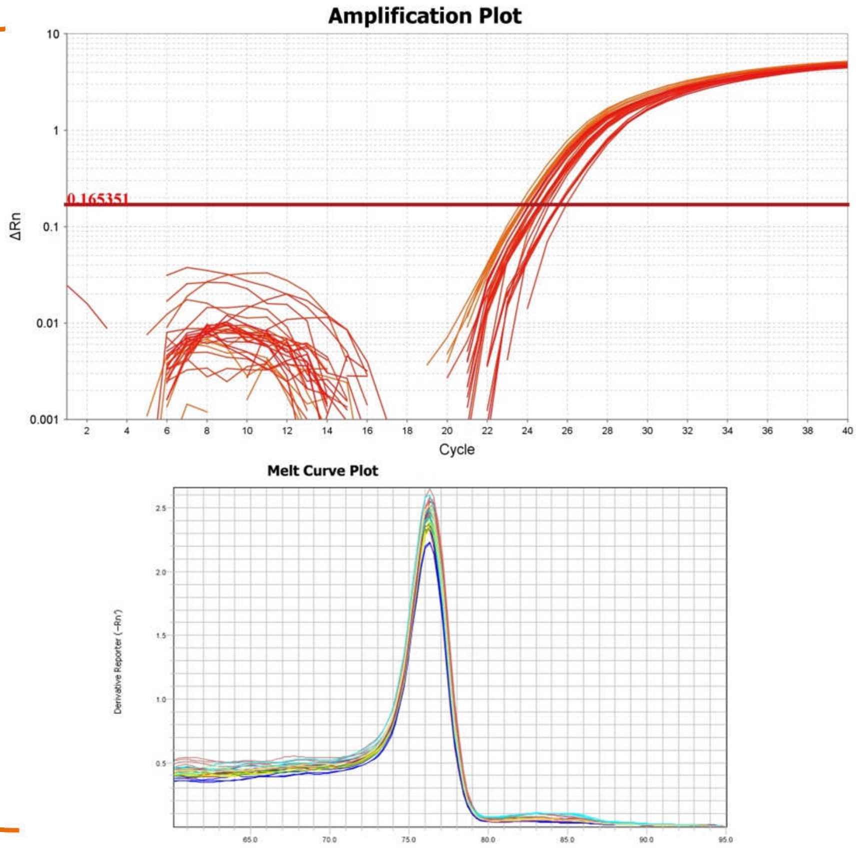

By convergent primers ▶◀ & with no template

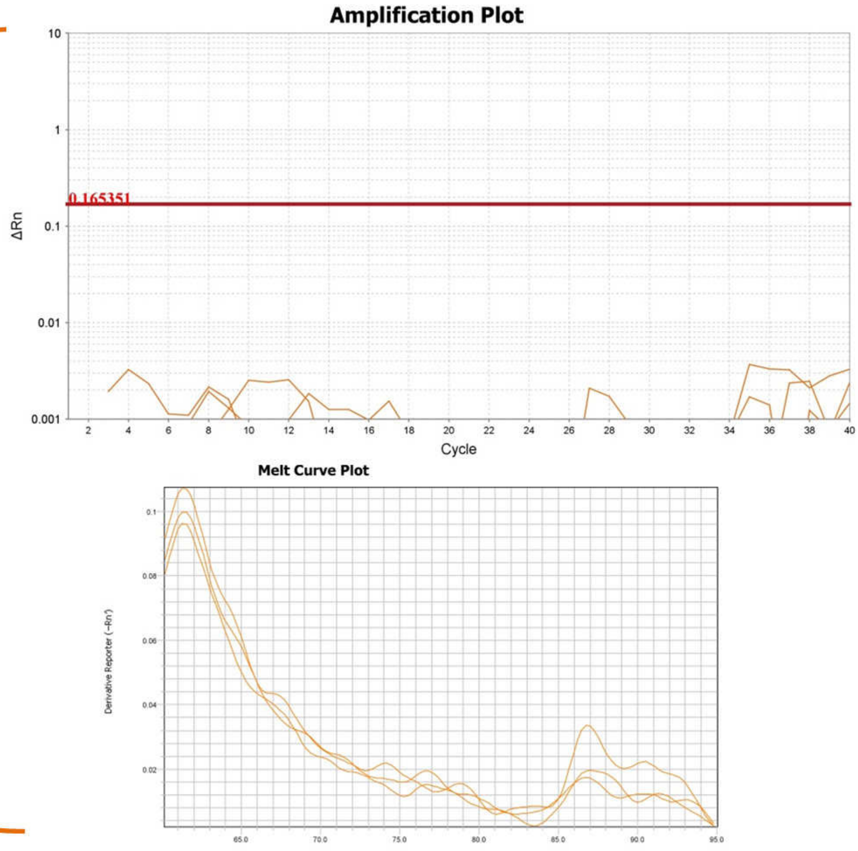

Mitogen-activated serine/threonine-protein kinase (Ctr1-like)_circular RNA (ID: Ch3:351423944-351425007)

GGTACACGGGTTGGAATAGGATTCTTTGGAGAGGTTTTCCGTGGTATATGGAATGGCACTGAT
GTTGCCATCAAAGTATTTCTGGAGCAGGATCTCACGACTGAAAACATGGAAGATTTTTGCAATGA
GATATACATCCTGA**GCCGGCTGCGGCATCCAAATGTAATATTGTTTCTTGGGGCATGCAT**

The nucleotides of junction-region are underlined. The nucleotides of junction-region which are supported by the junction-spaning sequencing reads are shown in red. Introns are not shown if the absence is supported by sequencing reads. In the absence of supporting sequencing reads, the intronic nucletides are shown as N.

Structural relationship between the circular RNA and its parental gene

```
                     20        40        60        80
Genomic(MLOC_56360)  AAAGAACGAGCCAATATTAATGTTCGAAGCAAATGCGCATAATAAAAACGGTACGGGCCGGCCGGCCGGGCACACAAGAG 80
cDNA(MLOC_56360)     AAAGAACGAGCCAATATTAATGTTCGAAGCAAATGCGCATAATAAAAACGGTACGGGCCGGCCGGCCGGGCACACAAGAG 80
CircularRNA          -------------------------------------------------------------------------------- -
Cir_Forward.Primer   -------------------------------------------------------------------------------- -
Cir_Rreverse.Primer  -------------------------------------------------------------------------------- -

                     100       120       140       160
Genomic(MLOC_56360)  AGCAAAGTGCTGCTACGAAACCCCCGTCACCAGGGCTCTCCCTCCTGGCCGCCCTGCCATTCCACAGTCCCCGCACAAAG 160
cDNA(MLOC_56360)     AGCAAAGTGCTGCTACGAAACCCCCGTCACCAGGGCTCTCCCTCCTGGCCGCCCTGCCATTCCACAGTCCCCGCACAAAG 160
CircularRNA          -------------------------------------------------------------------------------- -
Cir_Forward.Primer   -------------------------------------------------------------------------------- -
Cir_Rreverse.Primer  -------------------------------------------------------------------------------- -

                     180       200       220       240
Genomic(MLOC_56360)  CCAGCCAGTGCGTACCGACGCTTCCCCCACAGCCTGGACTGGAGCCGCCGTACTTTTCACTCTCTCGTCGGCTCCCTCCC 240
cDNA(MLOC_56360)     CCAGCCAGTGCGTACCGACGCTTCCCCCACAGCCTGGACTGGAGCCGCCGTACTTTTCACTCTCTCGTCGGCTCCCTCCC 240
CircularRNA          -------------------------------------------------------------------------------- -
Cir_Forward.Primer   -------------------------------------------------------------------------------- -
Cir_Rreverse.Primer  -------------------------------------------------------------------------------- -

                     260       280       300       320
Genomic(MLOC_56360)  TCGTTCCCGGGTCTCAGCTCTCAAGTCTCAACTCCCAGTTCTCCTCCTCCTCCCCCTGGCCCTCGCCCGCACGCTGCTCG 320
cDNA(MLOC_56360)     TCGTTCCCGGGTCTCAGCTCTCAAGTCTCAACTCCCAGTTCTCCTCCTCCTCCCCCTGGCCCTCGCCCGCACGCTGCTCG 320
CircularRNA          -------------------------------------------------------------------------------- -
Cir_Forward.Primer   -------------------------------------------------------------------------------- -
Cir_Rreverse.Primer  -------------------------------------------------------------------------------- -

                     340       360       380       400
Genomic(MLOC_56360)  CTCGATCGTCGCCTACCTAGTACCTACCATACCATCACACCTACCGCCCCTCTCTCCCACTGTCTCTCTCGTCCGCACCG 400
cDNA(MLOC_56360)     CTCGATCGTCGCCTACCTAGTACCTACCATACCATCACACCTACCGCCCCTCTCTCCCACTGTCTCTCTCGTCCGCACCG 400
CircularRNA          -------------------------------------------------------------------------------- -
Cir_Forward.Primer   -------------------------------------------------------------------------------- -
Cir_Rreverse.Primer  -------------------------------------------------------------------------------- -

                     420       440       460       480
Genomic(MLOC_56360)  TCAGACCCCCCCGCGCCCGCGATCGACCCCGCGCCGCCCGCACGCTCCCGAATCCTCAGCCGCCGGACGCCTCCTTCCGA 480
cDNA(MLOC_56360)     TCAGACCCCCCCGCGCCCGCGATCGACCCCGCGCCGCCCGCACGCTCCCGAATCCTCAGCCGCCGGACGCCTCCTTCCGA 480
CircularRNA          -------------------------------------------------------------------------------- -
Cir_Forward.Primer   -------------------------------------------------------------------------------- -
Cir_Rreverse.Primer  -------------------------------------------------------------------------------- -

                     500       520       540       560
Genomic(MLOC_56360)  CGAAGCGAAGCAGGCCGCCTCGGGCTCTCCGCCACCGCGTCCTCCTCCCTCCGCACTGCGCCGGCCGCTGCTGGATTGGC 560
cDNA(MLOC_56360)     CGAAGCGAAGCAGGCCGCCTCGGGCTCTCCGCCACCGCGTCCTCCTCCCTCCGCACTGCGCCGGCCGCTGCTGGATTGGC 560
CircularRNA          -------------------------------------------------------------------------------- -
Cir_Forward.Primer   -------------------------------------------------------------------------------- -
Cir_Rreverse.Primer  -------------------------------------------------------------------------------- -

                     580       600       620       640
Genomic(MLOC_56360)  GTGGAGATCCACCTGGGCCTGTTTCTCCTGCTGCCTGGTAAGCCGGCCCTCCCTCACCTGCCTGGATTCGTTTCGCTCTG 640
cDNA(MLOC_56360)     GTGGAGATCCACCTGGGCCTGTTTCTCCTGCTGCCTG------------------------------------------- 597
CircularRNA          -------------------------------------------------------------------------------- -
Cir_Forward.Primer   -------------------------------------------------------------------------------- -
Cir_Rreverse.Primer  -------------------------------------------------------------------------------- -

                     660       680       700       720
Genomic(MLOC_56360)  ATCTAGCCGGTCTGCCCTGGCCTGCGCCGCGCTGCGAGAAATCGAATCCGAATATATGGTCCACGCCCTGTGCAATCTGT 720
cDNA(MLOC_56360)     -------------------------------------------------------------------------------- 597
CircularRNA          -------------------------------------------------------------------------------- -
Cir_Forward.Primer   -------------------------------------------------------------------------------- -
Cir_Rreverse.Primer  -------------------------------------------------------------------------------- -

                     740       760       780       800
Genomic(MLOC_56360)  GTAAATGCGTCCGTACCGGGGAAATATACTCGCTGCAGGTGCGGATTCGCTCGCTGGTACGCCGCCAGTGCACGCCGCCC 800
cDNA(MLOC_56360)     -------------------------------------------------------------------------------- 597
CircularRNA          -------------------------------------------------------------------------------- -
Cir_Forward.Primer   -------------------------------------------------------------------------------- -
Cir_Rreverse.Primer  -------------------------------------------------------------------------------- -

                     820       840       860       880
Genomic(MLOC_56360)  ATCCGTATGATTCGGTTCCTGTTGCTCAGCGCGCGCGGCATCTCAGTCTCAGGGCCGTTATTGTCCTGCGACCCCAGAGA 880
cDNA(MLOC_56360)     -------------------------------------------------------------------------------- 597
CircularRNA          -------------------------------------------------------------------------------- -
Cir_Forward.Primer   -------------------------------------------------------------------------------- -
Cir_Rreverse.Primer  -------------------------------------------------------------------------------- -

                     900       920       940       960
Genomic(MLOC_56360)  ATCGGACTCGAGGTGCAGGCGCGCATGATTCCTCCCATTTCGGTCATAAATAGCCGCCGGAGGAAAGGCCTGAGCTACAG 960
cDNA(MLOC_56360)     -------------------------------------------------------------------------------- 597
CircularRNA          -------------------------------------------------------------------------------- -
Cir_Forward.Primer   -------------------------------------------------------------------------------- -
Cir_Rreverse.Primer  -------------------------------------------------------------------------------- -

                     980       1.000     1.020     1.040
Genomic(MLOC_56360)  CCCGACGCCGTTACATGGTCGTTTATGGAATTGTGGGATGTACTATGGTTACTGTATGTTGACGCGTAGAGTACTGGTCT 1040
cDNA(MLOC_56360)     -------------------------------------------------------------------------------- 597
CircularRNA          -------------------------------------------------------------------------------- -
Cir_Forward.Primer   -------------------------------------------------------------------------------- -
Cir_Rreverse.Primer  -------------------------------------------------------------------------------- -

                     1.060     1.080     1.100     1.120
Genomic(MLOC_56360)  GTTTTACCTTTTTTCCATTTTTACCCAAGGTTTTTACCAGAGTTGCTATTTTTTTGGTAAATAAGAGCCTGGATCTACAC 1120
cDNA(MLOC_56360)     -------------------------------------------------------------------------------- 597
CircularRNA          -------------------------------------------------------------------------------- -
Cir_Forward.Primer   -------------------------------------------------------------------------------- -
Cir_Rreverse.Primer  -------------------------------------------------------------------------------- -
```

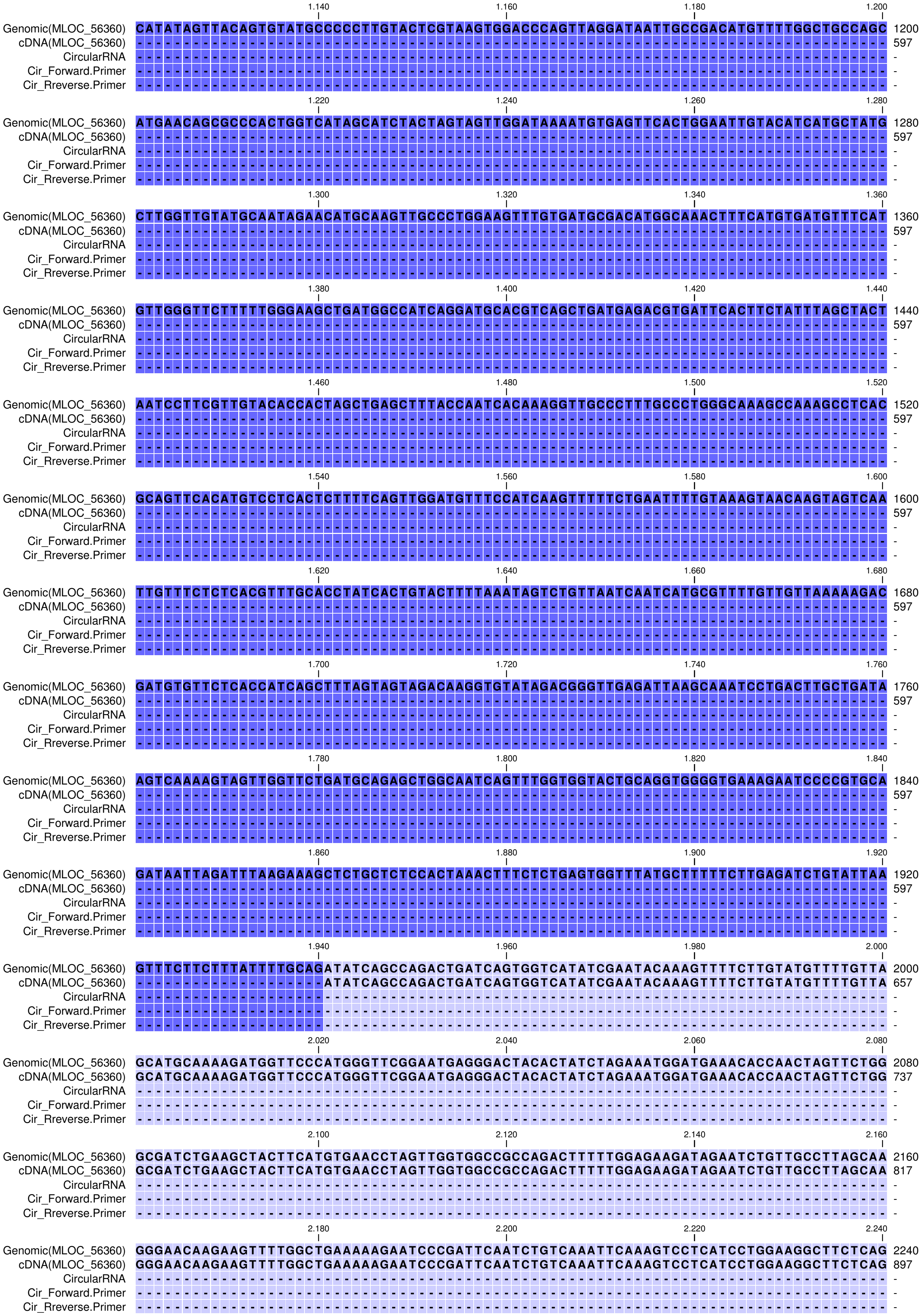
1.140 1.160 1.180 1.200
Genomic(MLOC_56360) CATATAGTTACAGTGTATGCCCCCTTGTACTCGTAAGTGGACCCAGTTAGGATAATTGCCGACATGTTTTGGCTGCCAGC 1200
cDNA(MLOC_56360) - 597
CircularRNA - -
Cir_Forward.Primer - -
Cir_Rreverse.Primer - -
1.220 1.240 1.260 1.280
Genomic(MLOC_56360) ATGAACAGCGCCCACTGGTCATAGCATCTACTAGTAGTTGGATAAAATGTGAGTTCACTGGAATTGTACATCATGCTATG 1280
cDNA(MLOC_56360) - 597
CircularRNA - -
Cir_Forward.Primer - -
Cir_Rreverse.Primer - -
1.300 1.320 1.340 1.360
Genomic(MLOC_56360) CTTGGTTGTATGCAATAGAACATGCAAGTTGCCCTGGAAGTTTGTGATGCGACATGGCAAACTTTCATGTGATGTTTCAT 1360
cDNA(MLOC_56360) - 597
CircularRNA - -
Cir_Forward.Primer - -
Cir_Rreverse.Primer - -
1.380 1.400 1.420 1.440
Genomic(MLOC_56360) GTTGGGTTCTTTTTGGGAAGCTGATGGCCATCAGGATGCACGTCAGCTGATGAGACGTGATTCACTTCTATTTAGCTACT 1440
cDNA(MLOC_56360) - 597
CircularRNA - -
Cir_Forward.Primer - -
Cir_Rreverse.Primer - -
1.460 1.480 1.500 1.520
Genomic(MLOC_56360) AATCCTTCGTTGTACACCACTAGCTGAGCTTTACCAATCACAAAGGTTGCCCTTTGCCCTGGGCAAAGCCAAAGCCTCAC 1520
cDNA(MLOC_56360) - 597
CircularRNA - -
Cir_Forward.Primer - -
Cir_Rreverse.Primer - -
1.540 1.560 1.580 1.600
Genomic(MLOC_56360) GCAGTTCACATGTCCTCACTCTTTTCAGTTGGATGTTTCCATCAAGTTTTTCTGAATTTTGTAAAGTAACAAGTAGTCAA 1600
cDNA(MLOC_56360) - 597
CircularRNA - -
Cir_Forward.Primer - -
Cir_Rreverse.Primer - -
1.620 1.640 1.660 1.680
Genomic(MLOC_56360) TTGTTTCTCTCACGTTTGCACCTATCACTGTACTTTTAAATAGTCTGTTAATCAATCATGCGTTTTGTTGTTAAAAAGAC 1680
cDNA(MLOC_56360) - 597
CircularRNA - -
Cir_Forward.Primer - -
Cir_Rreverse.Primer - -
1.700 1.720 1.740 1.760
Genomic(MLOC_56360) GATGTGTTCTCACCATCAGCTTTAGTAGTAGACAAGGTGTATAGACGGGTTGAGATTAAGCAAATCCTGACTTGCTGATA 1760
cDNA(MLOC_56360) - 597
CircularRNA - -
Cir_Forward.Primer - -
Cir_Rreverse.Primer - -
1.780 1.800 1.820 1.840
Genomic(MLOC_56360) AGTCAAAAGTAGTTGGTTCTGATGCAGAGCTGGCAATCAGTTTGGTGGTACTGCAGGTGGGGTGAAAGAATCCCCGTGCA 1840
cDNA(MLOC_56360) - 597
CircularRNA - -
Cir_Forward.Primer - -
Cir_Rreverse.Primer - -
1.860 1.880 1.900 1.920
Genomic(MLOC_56360) GATAATTAGATTTAAGAAAGCTCTGCTCTCCACTAAACTTTCTCTGAGTGGTTTATGCTTTTTCTTGAGATCTGTATTAA 1920
cDNA(MLOC_56360) - 597
CircularRNA - -
Cir_Forward.Primer - -
Cir_Rreverse.Primer - -
1.940 1.960 1.980 2.000
Genomic(MLOC_56360) GTTTCTTCTTTATTTTGCAGATATCAGCCAGACTGATCAGTGGTCATATCGAATACAAAGTTTTCTTGTATGTTTTGTTA 2000
cDNA(MLOC_56360) --------------------ATATCAGCCAGACTGATCAGTGGTCATATCGAATACAAAGTTTTCTTGTATGTTTTGTTA 657
CircularRNA - -
Cir_Forward.Primer - -
Cir_Rreverse.Primer - -
2.020 2.040 2.060 2.080
Genomic(MLOC_56360) GCATGCAAAAGATGGTTCCCATGGGTTCGGAATGAGGGACTACACTATCTAGAAATGGATGAAACACCAACTAGTTCTGG 2080
cDNA(MLOC_56360) GCATGCAAAAGATGGTTCCCATGGGTTCGGAATGAGGGACTACACTATCTAGAAATGGATGAAACACCAACTAGTTCTGG 737
CircularRNA - -
Cir_Forward.Primer - -
Cir_Rreverse.Primer - -
2.100 2.120 2.140 2.160
Genomic(MLOC_56360) GCGATCTGAAGCTACTTCATGTGAACCTAGTTGGTGGCCGCCAGACTTTTTGGAGAAGATAGAATCTGTTGCCTTAGCAA 2160
cDNA(MLOC_56360) GCGATCTGAAGCTACTTCATGTGAACCTAGTTGGTGGCCGCCAGACTTTTTGGAGAAGATAGAATCTGTTGCCTTAGCAA 817
CircularRNA - -
Cir_Forward.Primer - -
Cir_Rreverse.Primer - -
2.180 2.200 2.220 2.240
Genomic(MLOC_56360) GGGAACAAGAAGTTTTGGCTGAAAAAGAATCCCGATTCAATCTGTCAAATTCAAAGTCCTCATCCTGGAAGGCTTCTCAG 2240
cDNA(MLOC_56360) GGGAACAAGAAGTTTTGGCTGAAAAAGAATCCCGATTCAATCTGTCAAATTCAAAGTCCTCATCCTGGAAGGCTTCTCAG 897
CircularRNA - -
Cir_Forward.Primer - -
Cir_Rreverse.Primer - -

```
                                      2.260               2.280               2.300               2.320
Genomic(MLOC_56360) TTACTGTGGTCAACTGGAACTTATTCAGGGTTTATTCCAAATGGTTTCTATTCGATCATCCCGGTGAGGATCTCCCCCTC 2320
   cDNA(MLOC_56360) TTACTGTGGTCAACTGGAACTTATTCAGGGTTTATTCCAAATGGTTTCTATTCGATCATCCCGG---------------- 961
        CircularRNA -------------------------------------------------------------------------------- -
   Cir_Forward.Primer -------------------------------------------------------------------------------- -
  Cir_Rreverse.Primer -------------------------------------------------------------------------------- -

                                      2.340               2.360               2.380               2.400
Genomic(MLOC_56360) CCTCAGTAAACATGTTCTGTTCTTTGGAGACATGGTCCTTGTAGAAGAACTACACTAGATTATTTGTGCACGTCATGTGG 2400
   cDNA(MLOC_56360) -------------------------------------------------------------------------------- 961
        CircularRNA -------------------------------------------------------------------------------- -
   Cir_Forward.Primer -------------------------------------------------------------------------------- -
  Cir_Rreverse.Primer -------------------------------------------------------------------------------- -

                                      2.420               2.440               2.460               2.480
Genomic(MLOC_56360) CTTTTGAGTTAGATGCTCCCAAATTCATGTCAAGCACTCACTTTCTGAAGAAGATGCTTAATGCTCCATCTAAACTGGTA 2480
   cDNA(MLOC_56360) -------------------------------------------------------------------------------- 961
        CircularRNA -------------------------------------------------------------------------------- -
   Cir_Forward.Primer -------------------------------------------------------------------------------- -
  Cir_Rreverse.Primer -------------------------------------------------------------------------------- -

                                      2.500               2.520               2.540               2.560
Genomic(MLOC_56360) ACCATGGACGTGGACTTAATTTGATTTTTTTTGGTTGTCCTTGGCCTGCTGCTGTTTCCAGGACTCTATGTAAGACAACA 2560
   cDNA(MLOC_56360) -------------------------------------------------------------------------------- 961
        CircularRNA -------------------------------------------------------------------------------- -
   Cir_Forward.Primer -------------------------------------------------------------------------------- -
  Cir_Rreverse.Primer -------------------------------------------------------------------------------- -

                                      2.580               2.600               2.620               2.640
Genomic(MLOC_56360) CATGTTATCCTGCCCATATCTTCCTTCTTGTGTTGTGCGGGTCTTGATGGATAGTTCTTTTGGTGGCTTTGTATTATTTC 2640
   cDNA(MLOC_56360) -------------------------------------------------------------------------------- 961
        CircularRNA -------------------------------------------------------------------------------- -
   Cir_Forward.Primer -------------------------------------------------------------------------------- -
  Cir_Rreverse.Primer -------------------------------------------------------------------------------- -

                                      2.660               2.680               2.700               2.720
Genomic(MLOC_56360) AGTGTAGTAACGATAAATCTGAAAAATGGAACAACCTATATATTTGAAGGGCAGCATTGTTGTAGACAATTACATTTCAT 2720
   cDNA(MLOC_56360) -------------------------------------------------------------------------------- 961
        CircularRNA -------------------------------------------------------------------------------- -
   Cir_Forward.Primer -------------------------------------------------------------------------------- -
  Cir_Rreverse.Primer -------------------------------------------------------------------------------- -

                                      2.740               2.760               2.780               2.800
Genomic(MLOC_56360) ATGGTTCAGAAAGTGTCACAGTTTTGTAGCACATGAAGCATGTCAACCTTCCCAATGTGTTTTTTTCAATGAGGAATGAA 2800
   cDNA(MLOC_56360) -------------------------------------------------------------------------------- 961
        CircularRNA -------------------------------------------------------------------------------- -
   Cir_Forward.Primer -------------------------------------------------------------------------------- -
  Cir_Rreverse.Primer -------------------------------------------------------------------------------- -

                                      2.820               2.840               2.860               2.880
Genomic(MLOC_56360) GTTGTTTTCTCGGAAAATTAGGAATCAAGTTCCTAGGTCACAAAAATTATGGATCATTGGAAAAGTACTAGAATTTGGAT 2880
   cDNA(MLOC_56360) -------------------------------------------------------------------------------- 961
        CircularRNA -------------------------------------------------------------------------------- -
   Cir_Forward.Primer -------------------------------------------------------------------------------- -
  Cir_Rreverse.Primer -------------------------------------------------------------------------------- -

                                      2.900               2.920               2.940               2.960
Genomic(MLOC_56360) TTATTATTTCGCAACAATTTCTACTTCTCTTCTCGAACTGCTTATTAAGTCCCGAATGATTTAGAGGATGCTCTGCTTTT 2960
   cDNA(MLOC_56360) -------------------------------------------------------------------------------- 961
        CircularRNA -------------------------------------------------------------------------------- -
   Cir_Forward.Primer -------------------------------------------------------------------------------- -
  Cir_Rreverse.Primer -------------------------------------------------------------------------------- -

                                      2.980               3.000               3.020               3.040
Genomic(MLOC_56360) GTTTCAGTTCTTTGTGGTTATGTTTTTTCCACCTGTGACGCTCTCATACAGGATAAAAAGCTGAAGGAGATTTTCCCAAC 3040
   cDNA(MLOC_56360) ----------------------------------------------------ATAAAAAGCTGAAGGAGATTTTCCCAAC 989
        CircularRNA -------------------------------------------------------------------------------- -
   Cir_Forward.Primer -------------------------------------------------------------------------------- -
  Cir_Rreverse.Primer -------------------------------------------------------------------------------- -

                                      3.060               3.080               3.100               3.120
Genomic(MLOC_56360) AATACCATCACTAAACGACCTTCAAAGTCTTGAAGCAGATGGGCTTAAGCCTGAAATAATTGTCGTAGATGCTGAAAAGG 3120
   cDNA(MLOC_56360) AATACCATCACTAAACGACCTTCAAAGTCTTGAAGCAGATGGGCTTAAGCCTGAAATAATTGTCGTAGATGCTGAAAAGG 1069
        CircularRNA -------------------------------------------------------------------------------- -
   Cir_Forward.Primer -------------------------------------------------------------------------------- -
  Cir_Rreverse.Primer -------------------------------------------------------------------------------- -

                                      3.140               3.160               3.180               3.200
Genomic(MLOC_56360) ATAAGAAGATTTTCATGTTGAAGCAGCTTAGTGGCGCACTTGTGAAAGGATTGAACAATCCAGCTCTAGTGATAAAGAAG 3200
   cDNA(MLOC_56360) ATAAGAAGATTTTCATGTTGAAGCAGCTTAGTGGCGCACTTGTGAAAGGATTGAACAATCCAGCTCTAGTGATAAAGAAG 1149
        CircularRNA -------------------------------------------------------------------------------- -
   Cir_Forward.Primer -------------------------------------------------------------------------------- -
  Cir_Rreverse.Primer -------------------------------------------------------------------------------- -

                                      3.220               3.240               3.260               3.280
Genomic(MLOC_56360) ATAGCAGGTTTGGTAAGTTCTAGAAATTATAACCGGGTACAACTGTTTATTTTTCACATGTGCAATTCCTCATCGGGGGC 3280
   cDNA(MLOC_56360) ATAGCAGGTTTGGT------------------------------------------------------------------ 1163
        CircularRNA -------------------------------------------------------------------------------- -
   Cir_Forward.Primer -------------------------------------------------------------------------------- -
  Cir_Rreverse.Primer -------------------------------------------------------------------------------- -

                                      3.300               3.320               3.340               3.360
Genomic(MLOC_56360) TTGGTTGATCACTGCTATGGTATGATCTTAGGGTACTTTTGATGCTTAATAAATGTAAAACCAAATTTGGTTAAAAAAAC 3360
   cDNA(MLOC_56360) -------------------------------------------------------------------------------- 1163
        CircularRNA -------------------------------------------------------------------------------- -
   Cir_Forward.Primer -------------------------------------------------------------------------------- -
  Cir_Rreverse.Primer -------------------------------------------------------------------------------- -
```

```
                      3.380               3.400               3.420               3.440
Genomic(MLOC_56360)   TTTCGGTTAGAGTAGAACTGGCGACTCTGATATTTTAGTTAGTCCTTTCTTTGGTTACATCAAATTGTTCTTGTTGAATT 3440
cDNA(MLOC_56360)      -------------------------------------------------------------------------------- 1163
CircularRNA           -------------------------------------------------------------------------------- -
Cir_Forward.Primer    -------------------------------------------------------------------------------- -
Cir_Rreverse.Primer   -------------------------------------------------------------------------------- -

                      3.460               3.480               3.500               3.520
Genomic(MLOC_56360)   AAGTAAGAAAAAATTAACTTGTTGCTTGATGTATCAAATAAAACTTGGCAAGTTATTCTCGAACAGGTTTTTGACTGCTT 3520
cDNA(MLOC_56360)      --------------------------------------------------------------------TTTTGACTGCTT 1175
CircularRNA           -------------------------------------------------------------------------------- -
Cir_Forward.Primer    -------------------------------------------------------------------------------- -
Cir_Rreverse.Primer   -------------------------------------------------------------------------------- -

                      3.540               3.560               3.580               3.600
Genomic(MLOC_56360)   CAAGGGCCAAAACTCTGATGCAAGCCCAGGAAGAGCTTCAACCGAAGATATTCACTTCTTCGGAAATAGAGGACCACAAC 3600
cDNA(MLOC_56360)      CAAGGGCCAAAACTCTGATGCAAGCCCAGGAAGAGCTTCAACCGAAGATATTCACTTCTTCGGAAATAGAGGACCACAAC 1255
CircularRNA           -------------------------------------------------------------------------------- -
Cir_Forward.Primer    -------------------------------------------------------------------------------- -
Cir_Rreverse.Primer   -------------------------------------------------------------------------------- -

                      3.620               3.640               3.660               3.680
Genomic(MLOC_56360)   TTCTTGGGCAGATAAGGCATGGATCATGCCGACCCCGAGCTATCCTATTTAAAGTTCTTGCCGATGCTGTTGGCCTTGAG 3680
cDNA(MLOC_56360)      TTCTTGGGCAGATAAGGCATGGATCATGCCGACCCCGAGCTATCCTATTTAAAGTTCTTGCCGATGCTGTTGGCCTTGAG 1335
CircularRNA           -------------------------------------------------------------------------------- -
Cir_Forward.Primer    -------------------------------------------------------------------------------- -
Cir_Rreverse.Primer   -------------------------------------------------------------------------------- -

                      3.700               3.720               3.740               3.760
Genomic(MLOC_56360)   AGTAAACTCGTTGTGGTATGTTGACATCCCTTTTCAATCTTTTGTATATTTCAGATATTTCTTTGTGCATCGAATAATAT 3760
cDNA(MLOC_56360)      AGTAAACTCGTTGTGG---------------------------------------------------------------- 1351
CircularRNA           -------------------------------------------------------------------------------- -
Cir_Forward.Primer    -------------------------------------------------------------------------------- -
Cir_Rreverse.Primer   -------------------------------------------------------------------------------- -

                      3.780               3.800               3.820               3.840
Genomic(MLOC_56360)   TCTGTCCTTCATAGGGTCTACCTGATGACGGTGCAGTTGGATTTGTGGACTCCTACAAACATATGTCTGTGGTAGTTCCG 3840
cDNA(MLOC_56360)      ---------------GTCTACCTGATGACGGTGCAGTTGGATTTGTGGACTCCTACAAACATATGTCTGTGGTAGTTCCG 1416
CircularRNA           -------------------------------------------------------------------------------- -
Cir_Forward.Primer    -------------------------------------------------------------------------------- -
Cir_Rreverse.Primer   -------------------------------------------------------------------------------- -

                      3.860               3.880               3.900               3.920
Genomic(MLOC_56360)   CTGAATTCTATGGAGCTACTTGTTGACCTTATGCGATTTCCCGGCCAGTTGATTCCTTTTTCAGCCAAGGCCATCTTTAT 3920
cDNA(MLOC_56360)      CTGAATTCTATGGAGCTACTTGTTGACCTTATGCGATTTCCCGGCCAGTTGATTCCTTTTTCAGCCAAGGCCATCTTTAT 1496
CircularRNA           -------------------------------------------------------------------------------- -
Cir_Forward.Primer    -------------------------------------------------------------------------------- -
Cir_Rreverse.Primer   -------------------------------------------------------------------------------- -

                      3.940               3.960               3.980               4.000
Genomic(MLOC_56360)   ATCACATATCTCTGCTGCGGGTGAGAGCGATTCAGCTGAAAATGACTCGTGTGATTCTCCCCTGGAGCCCAACAGCCCTC 4000
cDNA(MLOC_56360)      ATCACATATCTCTGCTGCGGGTGAGAGCGATTCAGCTGAAAATGACTCGTGTGATTCTCCCCTGGAGCCCAACAGCCCTC 1576
CircularRNA           -------------------------------------------------------------------------------- -
Cir_Forward.Primer    -------------------------------------------------------------------------------- -
Cir_Rreverse.Primer   -------------------------------------------------------------------------------- -

                      4.020               4.040               4.060               4.080
Genomic(MLOC_56360)   TATATGGATTATCTGATAAAGTTGAAGCTGAAGGGTATGCTTATGCATGAAATTTCTCCTCTTAGTTATGCAATTCAGGC 4080
cDNA(MLOC_56360)      TATATGGATTATCTGATAAAGTTGAAGCTGAAGG---------------------------------------------- 1610
CircularRNA           -------------------------------------------------------------------------------- -
Cir_Forward.Primer    -------------------------------------------------------------------------------- -
Cir_Rreverse.Primer   -------------------------------------------------------------------------------- -

                      4.100               4.120               4.140               4.160
Genomic(MLOC_56360)   CTTTGTTATTTTTTTCATATCACCATGAGTATCGTCATAGAGAGTTCATAGTTCACTCACTCGACAAATTTAGTTGGCAT 4160
cDNA(MLOC_56360)      -------------------------------------------------------------------------------- 1610
CircularRNA           -------------------------------------------------------------------------------- -
Cir_Forward.Primer    -------------------------------------------------------------------------------- -
Cir_Rreverse.Primer   -------------------------------------------------------------------------------- -

                      4.180               4.200               4.220               4.240
Genomic(MLOC_56360)   ATTGAGTGGCTCACACCCTTTATGGATGAACTTATACCGTGAACCTTTGAACATATAGCATGGCCTTATGATTATTTATA 4240
cDNA(MLOC_56360)      -------------------------------------------------------------------------------- 1610
CircularRNA           -------------------------------------------------------------------------------- -
Cir_Forward.Primer    -------------------------------------------------------------------------------- -
Cir_Rreverse.Primer   -------------------------------------------------------------------------------- -

                      4.260               4.280               4.300               4.320
Genomic(MLOC_56360)   GATAAAAAAATCATAGCTTGTGTTGTTGGGCAGGGTATTAGTGACTTTTAAACATAATCATGTAGTAGAACTGTAAGATT 4320
cDNA(MLOC_56360)      -------------------------------------------------------------------------------- 1610
CircularRNA           -------------------------------------------------------------------------------- -
Cir_Forward.Primer    -------------------------------------------------------------------------------- -
Cir_Rreverse.Primer   -------------------------------------------------------------------------------- -

                      4.340               4.360               4.380               4.400
Genomic(MLOC_56360)   GATAATTATTCTAGCATATGATTGTTGAAACCAATAATTTTGTAAGACAGATGGTGATGTATGCCCCTTGCTGGTTTAGG 4400
cDNA(MLOC_56360)      -------------------------------------------------------------------------------- 1610
CircularRNA           -------------------------------------------------------------------------------- -
Cir_Forward.Primer    -------------------------------------------------------------------------------- -
Cir_Rreverse.Primer   -------------------------------------------------------------------------------- -

                      4.420               4.440               4.460               4.480
Genomic(MLOC_56360)   TTGGTTGGTTGGAACATTATTGATTGTATGGTGGCTACAACAGTGAATCTTCACACTGGCCGCAATAGAAGCTGAGATAG 4480
cDNA(MLOC_56360)      -------------------------------------------------------------------------------- 1610
CircularRNA           -------------------------------------------------------------------------------- -
Cir_Forward.Primer    -------------------------------------------------------------------------------- -
Cir_Rreverse.Primer   -------------------------------------------------------------------------------- -
```

4.500 4.520 4.540 4.560

Genomic(MLOC_56360) GTTATTTGCATGAAAAAAATACAGATATTTGTGAAATTGGCATCGGAAGTTTATATCTGACAACATTAACCAAATAAAGA 4560
cDNA(MLOC_56360) 1610
CircularRNA -
Cir_Forward.Primer -
Cir_Rreverse.Primer -

4.580 4.600 4.620 4.640

Genomic(MLOC_56360) AATTGTACAGAAGAAAATCAGTCATGGATATTTTAATTGTATAGTTGTTGTGCTATAGTTCTAAATAGCGTCTGTATTGG 4640
cDNA(MLOC_56360) 1610
CircularRNA -
Cir_Forward.Primer -
Cir_Rreverse.Primer -

4.660 4.680 4.700 4.720

Genomic(MLOC_56360) CTGATATAAATATTGCGGTTTTGGCTGCCTGCTTATATCATGTCTACGAGCAGGATATCCAATACTTTTAATCTTGAAAT 4720
cDNA(MLOC_56360) 1610
CircularRNA -
Cir_Forward.Primer -
Cir_Rreverse.Primer -

4.740 4.760 4.780 4.800

Genomic(MLOC_56360) TACCATTAGAAAGGCAAAGAGATCTAGTAGAAGAATAGGTTACATGCTCAGGTATATCATATATGGAGAAAAAGGCACCT 4800
cDNA(MLOC_56360) 1610
CircularRNA -
Cir_Forward.Primer -
Cir_Rreverse.Primer -

4.820 4.840 4.860 4.880

Genomic(MLOC_56360) ACCAGCTGTATCGGTTCTTTCCTTTTTTTTGTGTGTCCAATCCTTCTATTATTATTTTCTAGCTTTCTAGAGTTCATATT 4880
cDNA(MLOC_56360) 1610
CircularRNA -
Cir_Forward.Primer -
Cir_Rreverse.Primer -

4.900 4.920 4.940 4.960

Genomic(MLOC_56360) TGAGCAAGATAAAAACATTCCATCTCTATCTCAGTACAGAACCGAAGCTTCATCAAATCTATCTGGACGTTCATTGCGTA 4960
cDNA(MLOC_56360) AACCGAAGCTTCATCAAATCTATCTGGACGTTCATTGCGTA 1651
CircularRNA -
Cir_Forward.Primer -
Cir_Rreverse.Primer -

4.980 5.000 5.020 5.040

Genomic(MLOC_56360) ATACAATGTTGAGATCGAGAACATTTTCAGAGGGGAAATTAAGGTATCACCTAATATAATATTAGTCACTATCCGTTGAT 5040
cDNA(MLOC_56360) ATACAATGTTGAGATCGAGAACATTTTCAGAGGGGAAATTA 1692
CircularRNA -
Cir_Forward.Primer -
Cir_Rreverse.Primer -

5.060 5.080 5.100 5.120

Genomic(MLOC_56360) TGGTAAATTTTGCTTTAGTGTACACCCCTTTGGGGATATGGCTTTCAAATTGCAGTCACTTACAATTACAAAGCATAGTT 5120
cDNA(MLOC_56360) 1692
CircularRNA -
Cir_Forward.Primer -
Cir_Rreverse.Primer -

5.140 5.160 5.180 5.200

Genomic(MLOC_56360) CCATATTTTTCTCTGGTTATCAGTATTGTTAAGCTTCACAACCAAAAGTCTGAACTGATGGAAAAGGCTAGGTAATCCAC 5200
cDNA(MLOC_56360) 1692
CircularRNA -
Cir_Forward.Primer -
Cir_Rreverse.Primer -

5.220 5.240 5.260 5.280

Genomic(MLOC_56360) ATATACACTTCAACACCCCCTCTCATGTGTGATGCGGAAGTCAACACGTGAATAGACTCAGAGGTATGGCTCAAGAGGGC 5280
cDNA(MLOC_56360) 1692
CircularRNA -
Cir_Forward.Primer -
Cir_Rreverse.Primer -

5.300 5.320 5.340 5.360

Genomic(MLOC_56360) TATACGTGGATACAGAGGGGGTTAGCAGCATTTTTTGGATTAATTGCGAAGTCAGGACTTGAACTCAAGACCTTAGGCAT 5360
cDNA(MLOC_56360) 1692
CircularRNA -
Cir_Forward.Primer -
Cir_Rreverse.Primer -

5.380 5.400 5.420 5.440

Genomic(MLOC_56360) TGATACCATGTTAAGCTTCATGCACTAGCCAACACAACCAAAAGTACGAACTGATGGAAAAAGCTAGGTACTACACATGT 5440
cDNA(MLOC_56360) 1692
CircularRNA -
Cir_Forward.Primer -
Cir_Rreverse.Primer -

5.460 5.480 5.500 5.520

Genomic(MLOC_56360) ACACTTCAACAAGTATTATTGCAAGGATAATAATCATTGTTATTGCAAAGTACGTACTATGCTACACAAATCAATGTACA 5520
cDNA(MLOC_56360) 1692
CircularRNA -
Cir_Forward.Primer -
Cir_Rreverse.Primer -

5.540 5.560 5.580 5.600

Genomic(MLOC_56360) CATGCATGCAGTGGATTTTTCCATATCTGCATGCAGAATTTTAAAGTAAGTTAGCATTCCAACCCCATACTCACATCATA 5600
cDNA(MLOC_56360) 1692
CircularRNA -
Cir_Forward.Primer -
Cir_Rreverse.Primer -

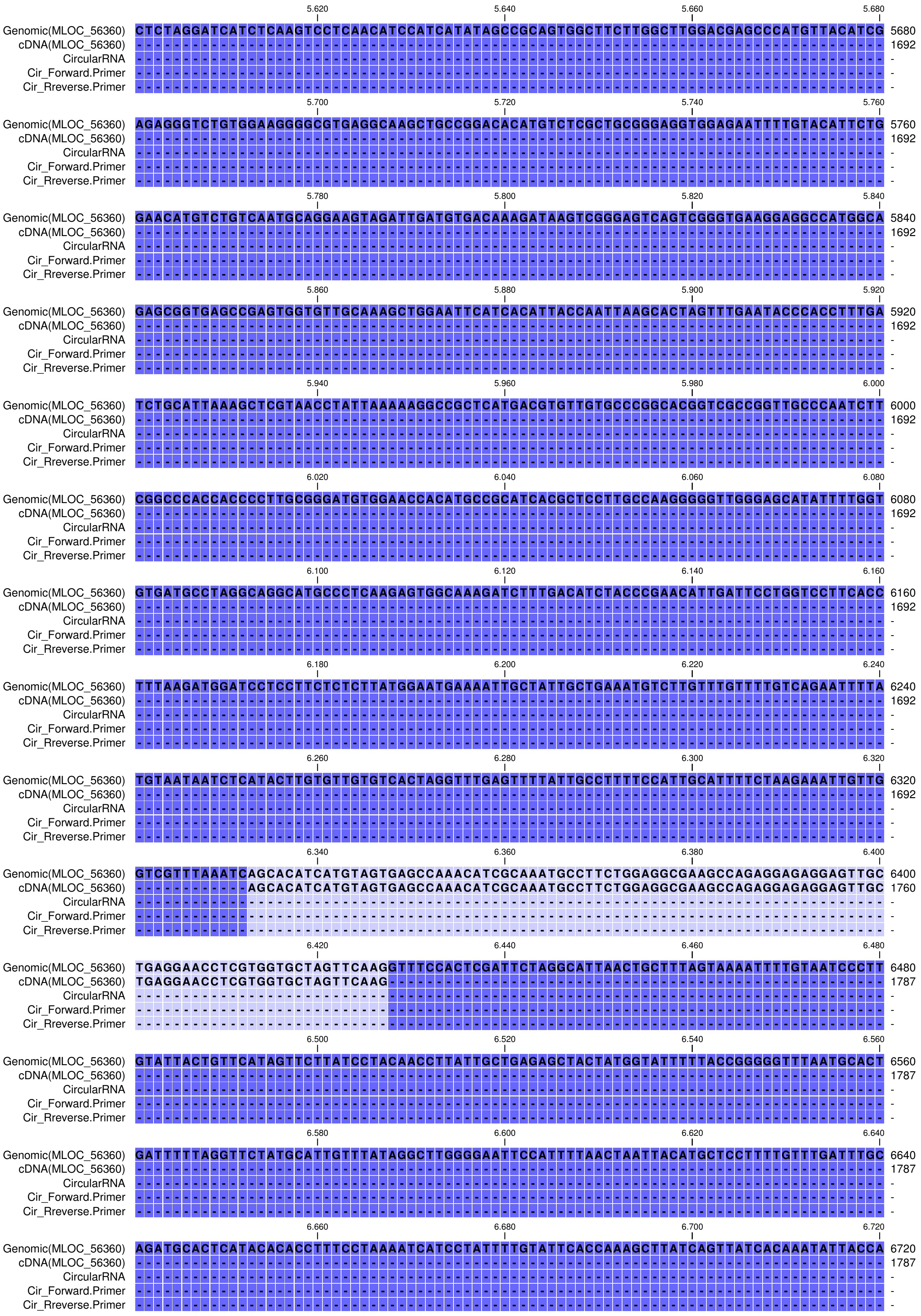
Genomic(MLOC_56360)
cDNA(MLOC_56360)
CircularRNA
Cir_Forward.Primer
Cir_Rreverse.Primer

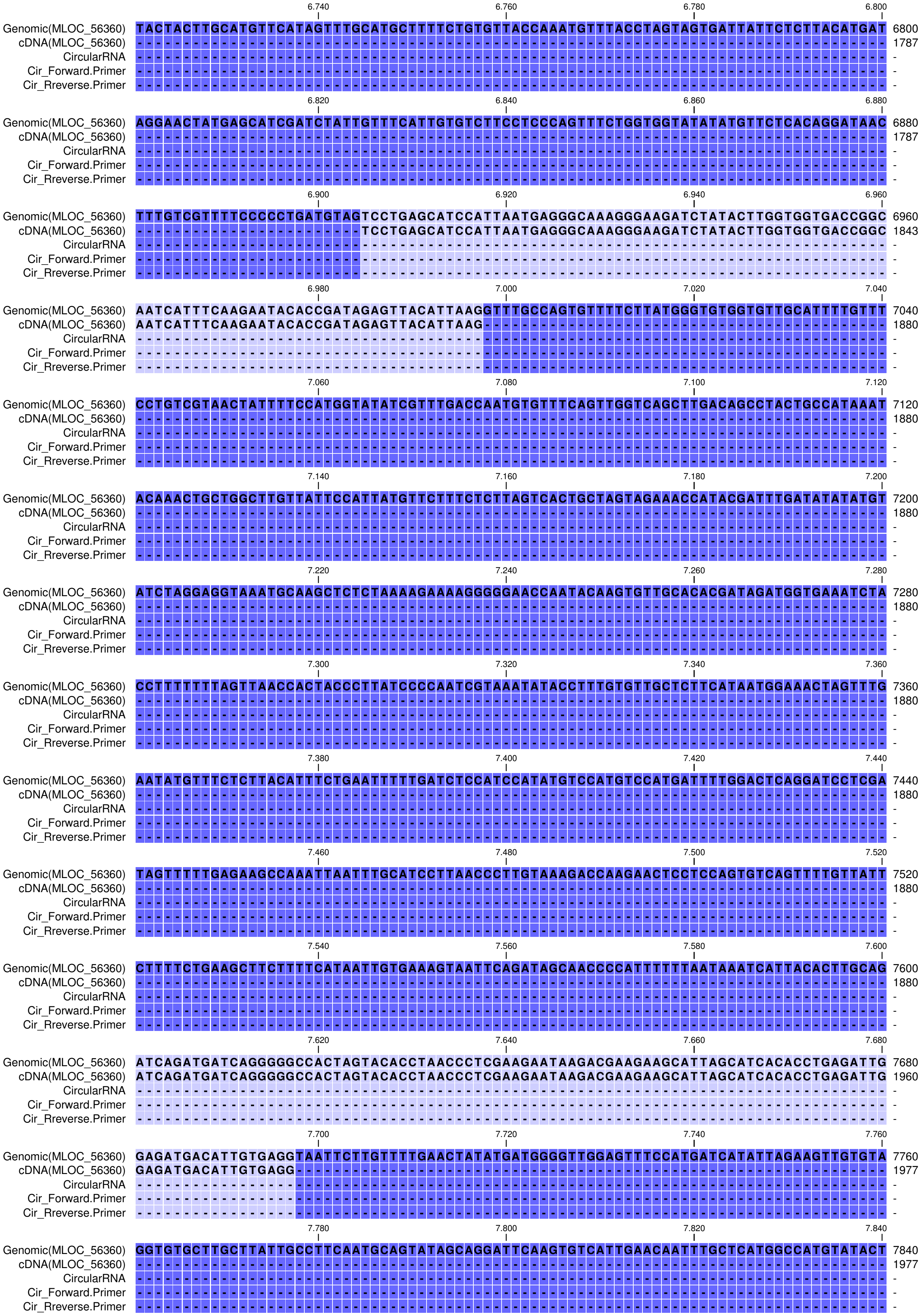
Genomic(MLOC_56360)
cDNA(MLOC_56360)
CircularRNA
Cir_Forward.Primer
Cir_Rreverse.Primer

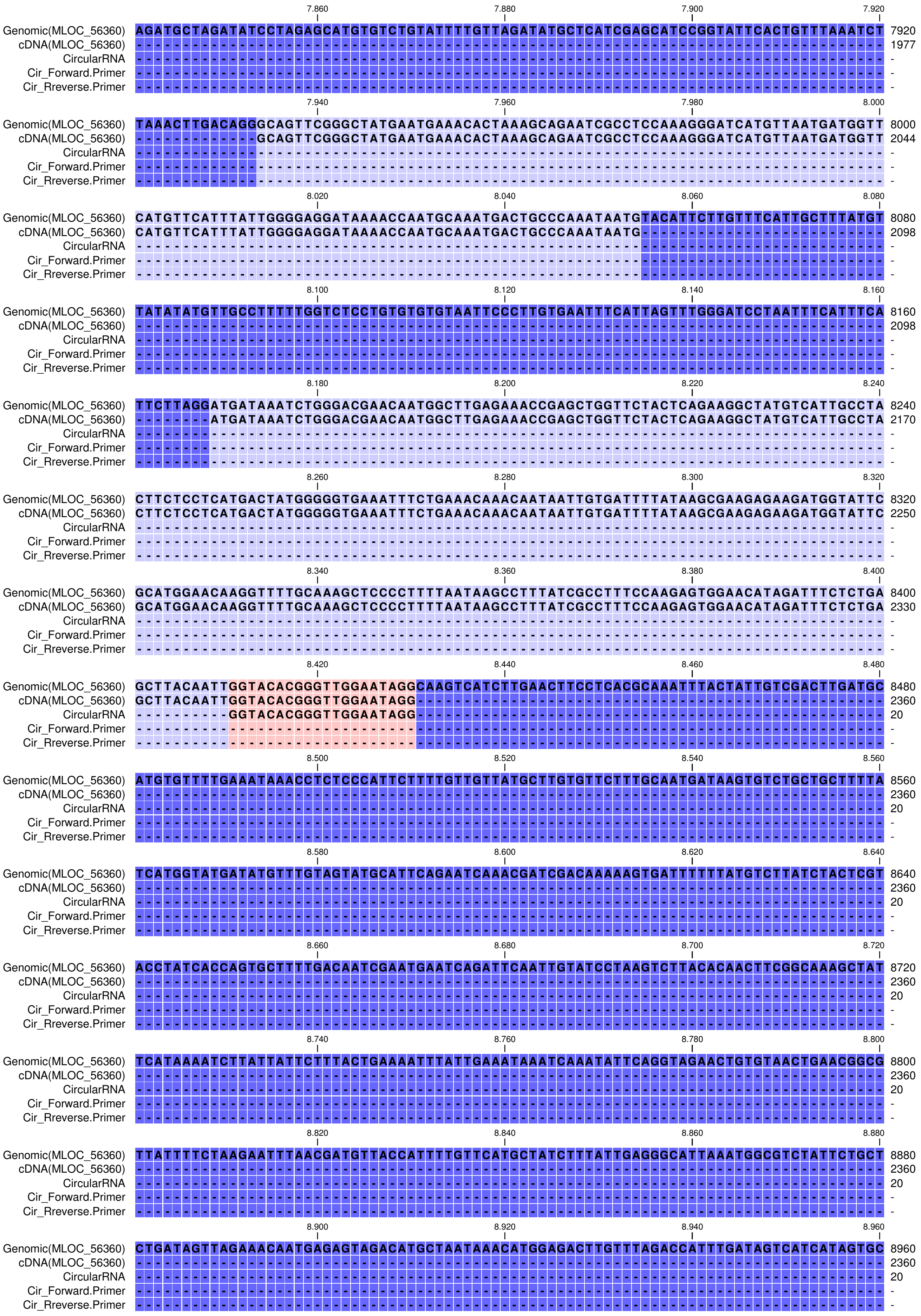
Genomic(MLOC_56360)
cDNA(MLOC_56360)
CircularRNA
Cir_Forward.Primer
Cir_Rreverse.Primer

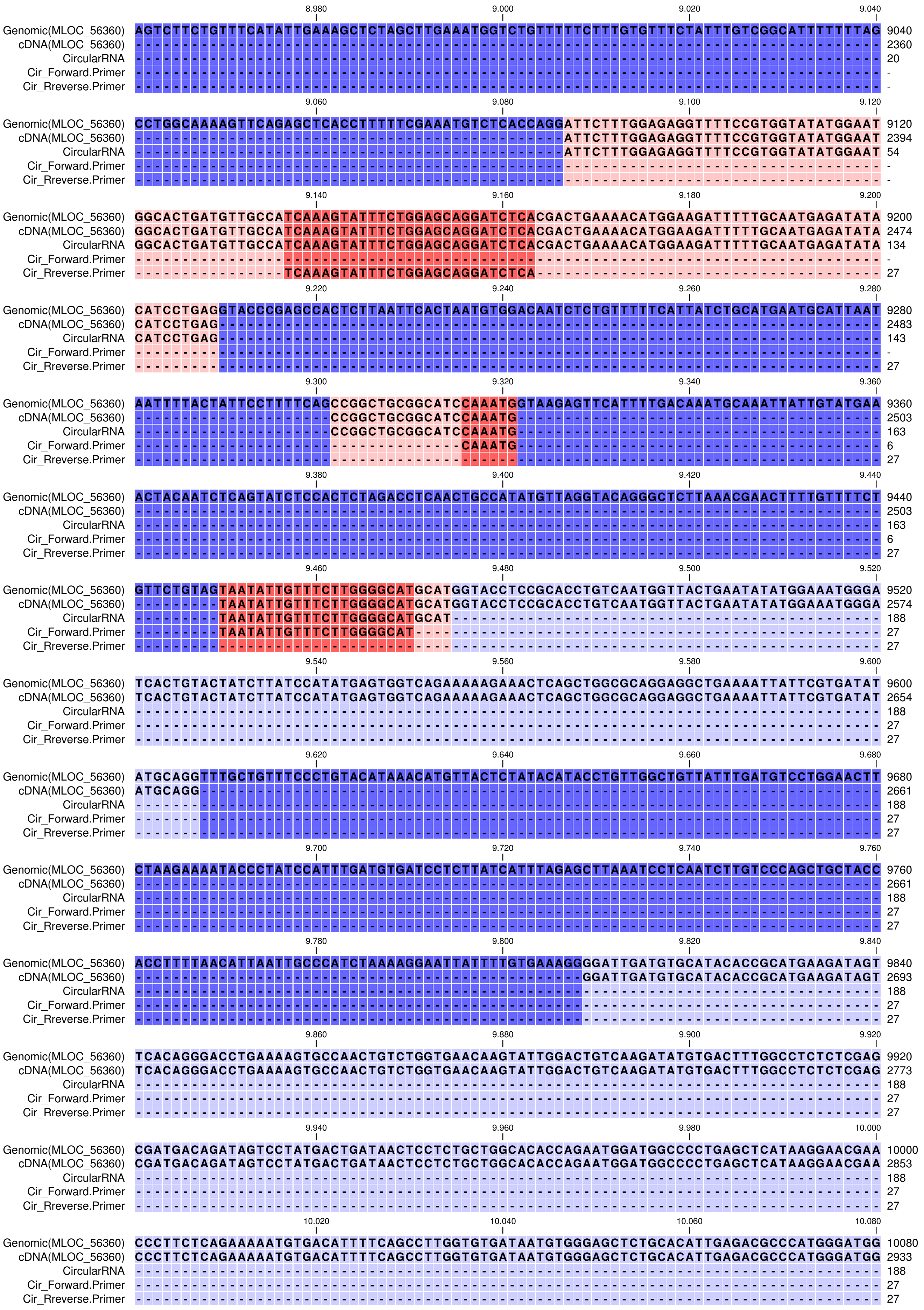
Genomic(MLOC_56360)
cDNA(MLOC_56360)
CircularRNA
Cir_Forward.Primer
Cir_Rreverse.Primer

```
                    10.100              10.120              10.140              10.160
Genomic(MLOC_56360) GATCGCCCCAGTTCAAGTAAGCATTCATCCACTTTCATCAAAACGTCTGCAATATAACCGAATCATCTAGAGATTCTCTT 10160
cDNA(MLOC_56360)    GATCGCCCCAGTTCAAGT-------------------------------------------------------------- 2951
CircularRNA         -------------------------------------------------------------------------------- 188
Cir_Forward.Primer  -------------------------------------------------------------------------------- 27
Cir_Rreverse.Primer -------------------------------------------------------------------------------- 27

                    10.180              10.200              10.220              10.240
Genomic(MLOC_56360) AACTGAAGCATACGCGGTATTCTGATCTCTACGGTTTTGATCATTAATCCGTCTGGTGCATCCCTATTTCTTGATGGTTC 10240
cDNA(MLOC_56360)    -------------------------------------------------------------------------------- 2951
CircularRNA         -------------------------------------------------------------------------------- 188
Cir_Forward.Primer  -------------------------------------------------------------------------------- 27
Cir_Rreverse.Primer -------------------------------------------------------------------------------- 27

                    10.260              10.280              10.300              10.320
Genomic(MLOC_56360) TTATACATGTTAGTAGGCAAAGCAGTTATTTTGCCCCAAGTGTTGTTGGCTGAACTTGAGAATTGGCGTTTTACAGGTGG 10320
cDNA(MLOC_56360)    ------------------------------------------------------------------------------GG 2953
CircularRNA         -------------------------------------------------------------------------------- 188
Cir_Forward.Primer  -------------------------------------------------------------------------------- 27
Cir_Rreverse.Primer -------------------------------------------------------------------------------- 27

                    10.340              10.360              10.380              10.400
Genomic(MLOC_56360) TGTATGCAGTTACTGAAGGGTCGGGGCTTGAAATTCCAGAAGGGCCTCTTGGCAAGTTAATTGCAGGTAATGATGCTTTC 10400
cDNA(MLOC_56360)    TGTATGCAGTTACTGAAGGGTCGGGGCTTGAAATTCCAGAAGGGCCTCTTGGCAAGTTAATTGCAG-------------- 3019
CircularRNA         -------------------------------------------------------------------------------- 188
Cir_Forward.Primer  -------------------------------------------------------------------------------- 27
Cir_Rreverse.Primer -------------------------------------------------------------------------------- 27

                    10.420              10.440              10.460              10.480
Genomic(MLOC_56360) TGAAGCGATGTTTTTATATTCCAGGTTTCCGGTGGAACAAAGTCTGGAATCACTTTGAGTCAATGTTTTCAGCTTATTC 10480
cDNA(MLOC_56360)    -------------------------------------------------------------------------------- 3019
CircularRNA         -------------------------------------------------------------------------------- 188
Cir_Forward.Primer  -------------------------------------------------------------------------------- 27
Cir_Rreverse.Primer -------------------------------------------------------------------------------- 27

                    10.500              10.520              10.540              10.560
Genomic(MLOC_56360) TGGTCTTTAGTTCCAGCTTAGGGACAATTGTTTTTGTTTACTGCAGATTGCTGGGCAGAGCCCCAAGATCGGCCGAGCTG 10560
cDNA(MLOC_56360)    ----------------------------------------------ATTGCTGGGCAGAGCCCCAAGATCGGCCGAGCTG 3053
CircularRNA         -------------------------------------------------------------------------------- 188
Cir_Forward.Primer  -------------------------------------------------------------------------------- 27
Cir_Rreverse.Primer -------------------------------------------------------------------------------- 27

                    10.580              10.600              10.620              10.640
Genomic(MLOC_56360) CCAGGAGATCCTCACTCGCTTGCTCGACTGCGAGTACGCTGAGAGCTGAGAGCCTGAAACGAACCTGGTGAATGCTTCAC 10640
cDNA(MLOC_56360)    CCAGGAGATCCTCACTCGCTTGCTCGACTGCGAGTACGCTGAGAGCTGAGAGCCTGAAACGAACCTGGTGAATGCTTCAC 3133
CircularRNA         -------------------------------------------------------------------------------- 188
Cir_Forward.Primer  -------------------------------------------------------------------------------- 27
Cir_Rreverse.Primer -------------------------------------------------------------------------------- 27

                    10.660              10.680              10.700              10.720
Genomic(MLOC_56360) TCCCAACCTCCCAGTGCCTTCAGAGGTGGAACCGTTTCTCTGTATAAAACTTGTAAAAATCTAGCCCGTTGATCCTGCAG 10720
cDNA(MLOC_56360)    TCCCAACCTCCCAGTGCCTTCAGAGGTGGAACCGTTTCTCTGTATAAAACTTGTAAAAATCTAGCCCGTTGATCCTGCAG 3213
CircularRNA         -------------------------------------------------------------------------------- 188
Cir_Forward.Primer  -------------------------------------------------------------------------------- 27
Cir_Rreverse.Primer -------------------------------------------------------------------------------- 27

                    10.740              10.760              10.780              10.800
Genomic(MLOC_56360) ACCGGCAAGGATATACATATATTAAACTCTTGTATTTCGGCTGTATATCGGCGACCCTTTCTTTCAAATTTGTAAAGGAA 10800
cDNA(MLOC_56360)    ACCGGCAAGGATATACATATATTAAACTCTTGTATTTCGGCTGTATATCGGCGACCCTTTCTTTCAAATTTGTAAAGGAA 3293
CircularRNA         -------------------------------------------------------------------------------- 188
Cir_Forward.Primer  -------------------------------------------------------------------------------- 27
Cir_Rreverse.Primer -------------------------------------------------------------------------------- 27

                    10.820              10.840              10.860
Genomic(MLOC_56360) AAATTCTCTCCTTTTAATAGAAGAATGTTGCAGGGCTAAGGCTAACTCCAACGCAGACGATCCATTTTGTCCGTCCG 10877
cDNA(MLOC_56360)    AAATTCTCTCCTTTTAATAGAAGAATGTTGCAGGGCT---------------------------------------- 3330
CircularRNA         ----------------------------------------------------------------------------- 188
Cir_Forward.Primer  ----------------------------------------------------------------------------- 27
Cir_Rreverse.Primer ----------------------------------------------------------------------------- 27
```

Real-Time PCR for the junction region of mitogen-activated serine/threonine-protein kinase (Ctr1-like)_circular RNA (ID: Ch3:351423944-351425007**)**

By divergent primers ◀▶ on genomic DNA

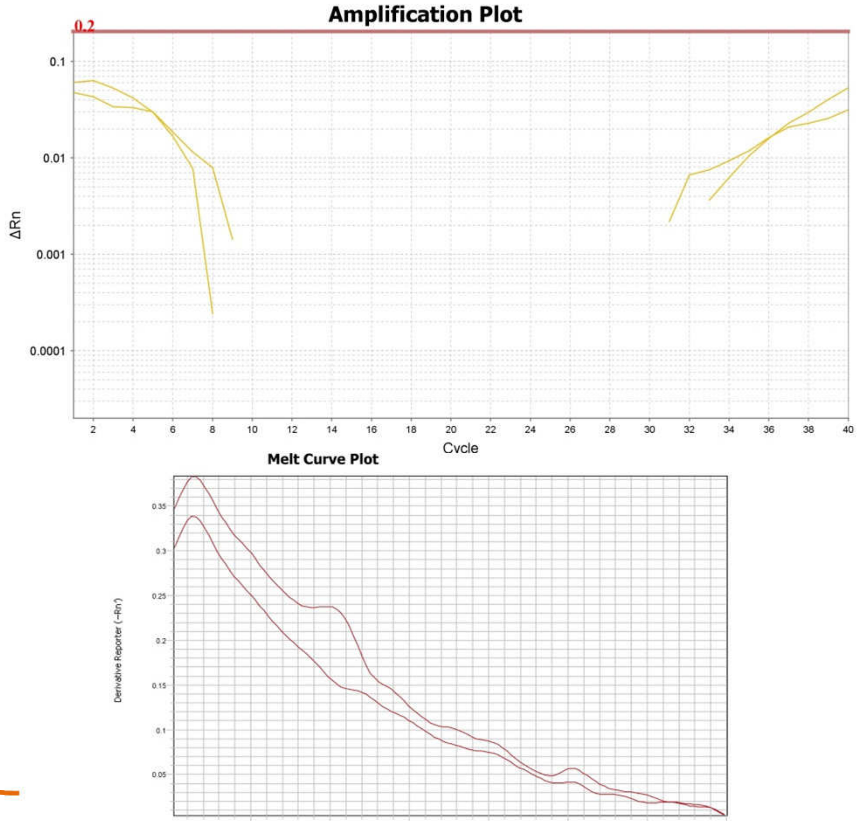

By divergent primers ◀▶ on cDNAs

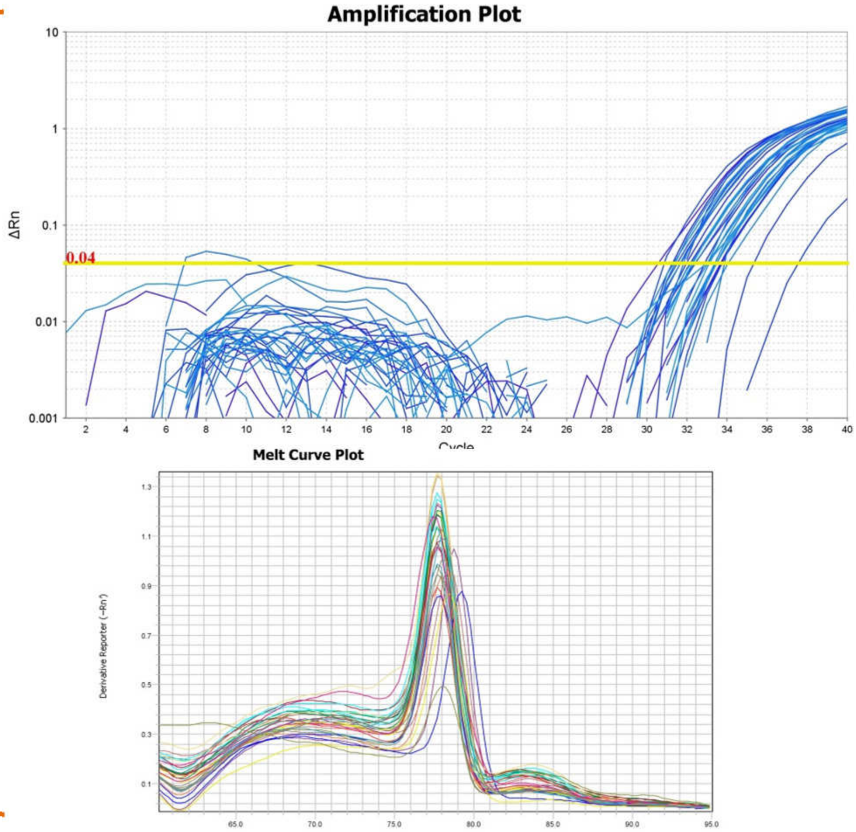

Real-Time PCR for the junction region of mitogen-activated serine/threonine-protein kinase (Ctr1-like)_circular RNA (ID: Ch3:351423944-351425007**)**

By divergent primers ◀▶ & with no template

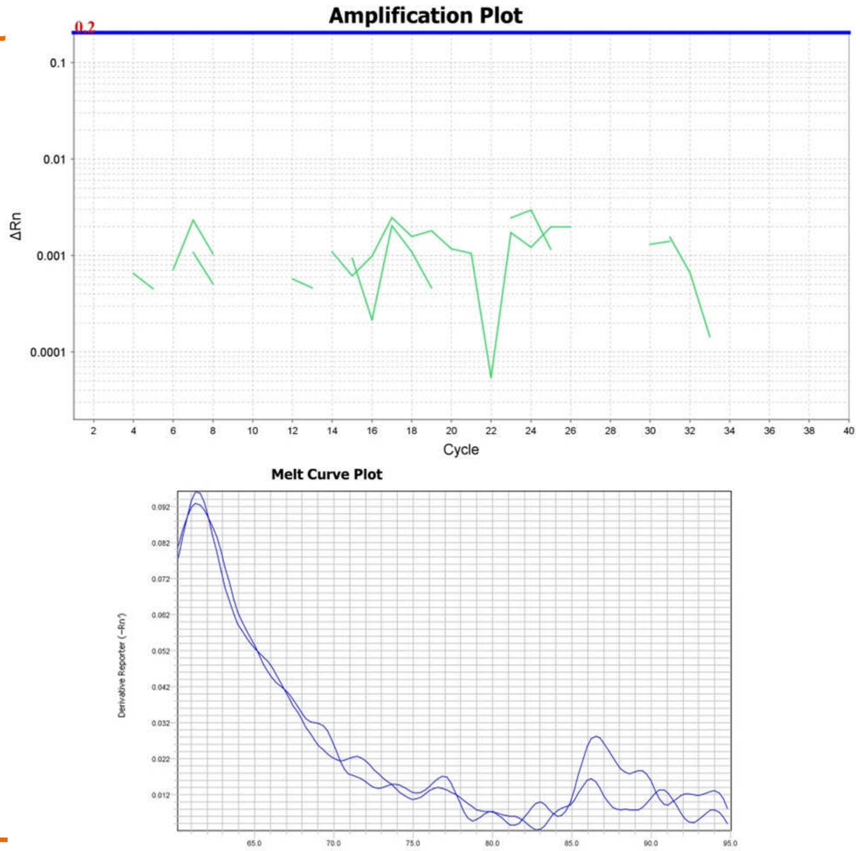

Real-Time PCR for the Mitogen-activated serine/threonine-protein kinase (Ctr1-like)

By convergent primers ▶◀ on cDNAs

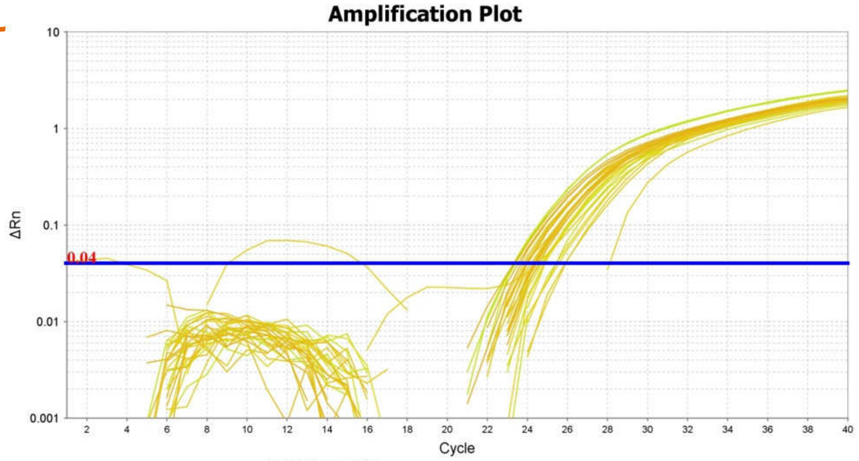

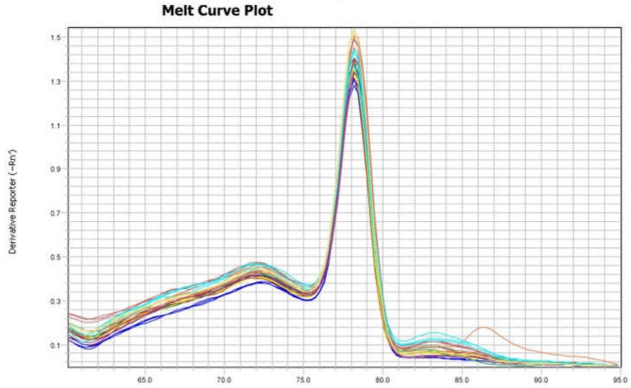

By convergent primers ▶◀ & with no template

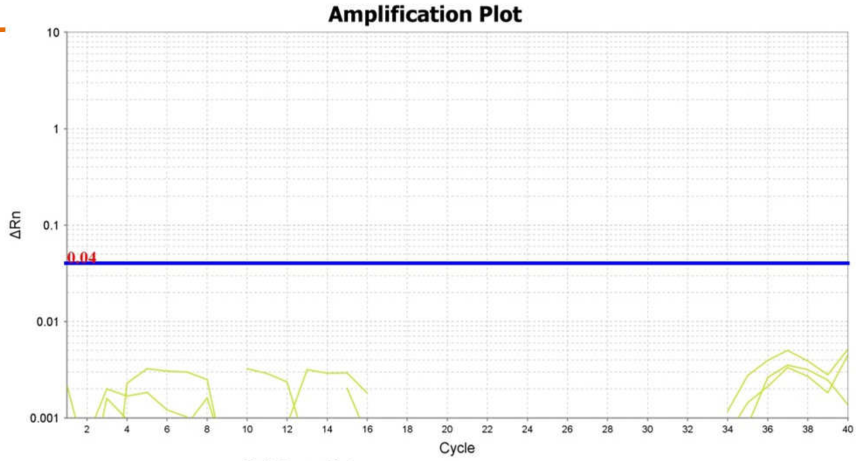

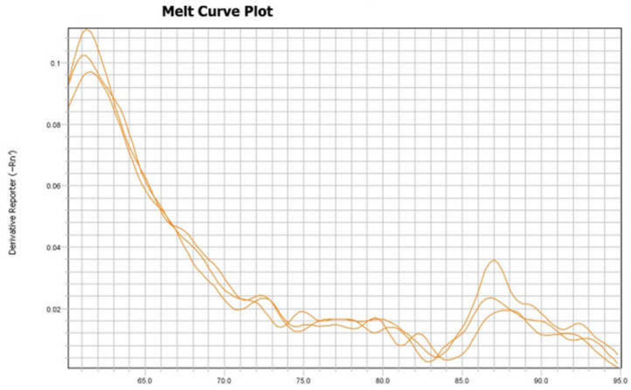

MicroRNA1126_circular RNA (ID: Ch2:467982888-467983386)

GCAACAAAATGAAGCTCTCCTTTCCATTAGCATATTCCAGAAGGATTTATTGCACATAAAAGT
GATGGCTTGGGCCTTCGGCACCATGCCGGCATCAGTTTGCATGTTGTCGCTCCATTTGTATTGC
TGTTGCAAAGAAAAGTGCCAACAGTGCATTGCCGTATGTTAGGAGGTGACACTTAAGCACTATN
NNNNNNNNNNNNNNNNNNNNNNNNNNNNNNNNNNNNNNNNNNNNNNNNNNNNNNNNNNNNN
NNNNNNNNNNNNNNNNNNNNNNNNNNNNNATAGCGTCAATTTCCCTGGCGTTGTGGGAGCTGC
TGCTGTTGCATGTAAATTGGTTTGTCCAAATCGACAGTCCGACGCTCCAGCCGATGCAGCTGCG
ACCCCGCCCTCTGGATCAGCTCACCACCCAACCAGTTACACTCAGTTATGTGACAGTTTTGATTC
ATTCTACAGAATTATTGGAACTCTGGAATAACCAAATCCCAATTCTGCTTCAAAG

The nucleotides of junction-region are underlined. The nucleotides of junction-region which are supported by the junction-spaning sequencing reads are shown in red. Introns are not shown if the absence is supported by sequencing reads. In the absence of supporting sequencing reads, the intronic nucleotides are shown as N.

Structural relationship between the circular RNA and its parental gene

```
                    20 40 60 80
Genomic(mir1126)    GCTGATGACGATCCATGCGAACAAGTGTATTATTAGGTGCAACATGGACTTATAAGTCTCTGTAAACAAACAGGTAGGGA 80
cDNA1(mir1126)      -------------------------------------------------------------------------------- -
cDNA2(mir1126)      -------------------------------------------------------------------------------- -
CircularRNA         -------------------------------------------------------------------------------- -
Cir_Forward.Primer  -------------------------------------------------------------------------------- -
Cir_Rreverse.Primer -------------------------------------------------------------------------------- -

                    100 120 140 160
Genomic(mir1126)    CTCGGGACTTATAAGTCCGTGTAAACAAACAGGTAGGCACTTATGACTTATAAGTTGAGACTTAAAAAAGTCCCAGGACT 160
cDNA1(mir1126)      -------------------------------------------------------------------------------- -
cDNA2(mir1126)      -------------------------------------------------------------------------------- -
CircularRNA         -------------------------------------------------------------------------------- -
Cir_Forward.Primer  -------------------------------------------------------------------------------- -
Cir_Rreverse.Primer -------------------------------------------------------------------------------- -

                    180 200 220 240
Genomic(mir1126)    TATAAAACAAACACGGCCTAAGTCCAACTATCTTATCAATCATTGTACGTGACCTAAGCGTAGATCGCCGAGCCATGCCA 240
cDNA1(mir1126)      -------------------------------------------------------------------------------- -
cDNA2(mir1126)      -------------------------------------------------------------------------------- -
CircularRNA         -------------------------------------------------------------------------------- -
Cir_Forward.Primer  -------------------------------------------------------------------------------- -
Cir_Rreverse.Primer -------------------------------------------------------------------------------- -

                    260 280 300 320
Genomic(mir1126)    CGTCATACATGCCATCGCTGAGCCATGCCGCGTCGAATCTACAGTGACGTTGGATTTTTTTATTTTTAAAAAAGGTAGAA 320
cDNA1(mir1126)      -------------------------------------------------------------------------------- -
cDNA2(mir1126)      -------------------------------------------------------------------------------- -
CircularRNA         -------------------------------------------------------------------------------- -
Cir_Forward.Primer  -------------------------------------------------------------------------------- -
Cir_Rreverse.Primer -------------------------------------------------------------------------------- -

                    340 360 380 400
Genomic(mir1126)    TTAATTTCTCTCCTAGAAAATGGCATGACGCAACGGCCCCTTTTGTTCCTCCCTTCCTCAGGCGGGCTAGGGTTGCCGCC 400
cDNA1(mir1126)      -------------TAGAAAATGGCATGACGCAACGGCCCCTTTTGTTCCTCCCTTCCTCAGGCGGGCTAGGGTTGCCGCC 67
cDNA2(mir1126)      ---------------------------------------------TTCCTCCCTTCCTCAGGCGGGCTAGGGTTGCCGCC 35
CircularRNA         -------------------------------------------------------------------------------- -
Cir_Forward.Primer  -------------------------------------------------------------------------------- -
Cir_Rreverse.Primer -------------------------------------------------------------------------------- -

                    420 440 460 480
Genomic(mir1126)    GCCGTCGCCCGAGCCCCATCGGCGCATCCCAGCTCTTCTGGCGCCCTTCACCAGAGAAGGCATCCACCTCCGATCTTCCA 480
cDNA1(mir1126)      GCCGTCGCCCGAGCCCCATCGGCGCATCCCAGCTCTTCTGGCGCCCTTCACCAGAGAAGGCATCCACCTCCGATCTTCCA 147
cDNA2(mir1126)      GCCGTCGCCCGAGCCCCATCGGCGCATCCCAGCTCTTCTGGCGCCCTTCACCAGAGAAGGCATCCACCTCCGATCTTCCA 115
CircularRNA         -------------------------------------------------------------------------------- -
Cir_Forward.Primer  -------------------------------------------------------------------------------- -
Cir_Rreverse.Primer -------------------------------------------------------------------------------- -

                    500 520 540 560
Genomic(mir1126)    TCCCCAAGCCTTCTCGTCTTCGCGCTCCACCCAGCCTTCATGATGGTAATAGAGGAGGGGGATGAAGGGGATGCCACAAC 560
cDNA1(mir1126)      TCCCCAAGCCTTCTCGTCTTCGCGCTCCACCCAGCCTTCATGATGGTAATAGAGGAGGGGGATGAAGGGGATGCCACAAC 227
cDNA2(mir1126)      TCCCCAAGCCTTCTCGTCTTCGCGCTCCACCCAGCCTTCATGATGGTAATAGAGGAGGGGGATGAAGGGGATGCCACAAC 195
CircularRNA         -------------------------------------------------------------------------------- -
Cir_Forward.Primer  -------------------------------------------------------------------------------- -
Cir_Rreverse.Primer -------------------------------------------------------------------------------- -

                    580 600 620 640
Genomic(mir1126)    GACCTCGCGCCCCCGGTGCTGCGCCCACGAGCGGAGGAGCGGGTAGTGGTGGCGGTGGAGGCGTGGTGATGTCGGGACGA 640
cDNA1(mir1126)      GACCTCGCGCCCCCGGTGCTGCGCCCACGAGCGGAGGAGCGGGTAGTGGTGGCGGTGGAGGCGTGGTGATGTCGGGACGA 307
cDNA2(mir1126)      GACCTCGCGCCCCCGGTGCTGCGCCCACGAGCGGAGGAGCGGGTAGTGGTGGCGGTGGAGGCGTGGTGATGTCGGGACGA 275
CircularRNA         -------------------------------------------------------------------------------- -
Cir_Forward.Primer  -------------------------------------------------------------------------------- -
Cir_Rreverse.Primer -------------------------------------------------------------------------------- -

                    660 680 700 720
Genomic(mir1126)    TGGTGTGGTCGGAGGCGCTATGTCGGCATGATAGCCGAGATCATGCATCAACTGTGCCACAAGGTGCGTCGCCTTCCCTT 720
cDNA1(mir1126)      TGGTGTGGTCGGAGGCGCTATGTCGGCATGATAGCCGAGATCATGCATCAACTGTGCCACAAGG---------------- 371
cDNA2(mir1126)      TGGTGTGGTCGGAGGCGCTATGTCGGCATGATAGCCGAGATCATGCATCAACTGTGCCACAAGGTGCGTCGCCTTCCCTT 355
CircularRNA         -------------------------------------------------------------------------------- -
Cir_Forward.Primer  -------------------------------------------------------------------------------- -
Cir_Rreverse.Primer -------------------------------------------------------------------------------- -

                    740 760 780 800
Genomic(mir1126)    CTCCCGTCGCGCCAACAAGCGTCGATGCGTGCAACCTTCTCACCGCCGGGCTCAACGGCCTGCTCACCCTCCCCTCCCCA 800
cDNA1(mir1126)      -------------------------------------------------------------------------------- 371
cDNA2(mir1126)      CTCCCGTCGCGCCAACAAGCGTCGATGCGTGCAACCTTCTCACCGCCGGGCTCAACGGCCTGCTCACCCTCCCCTCCCCA 435
CircularRNA         -------------------------------------------------------------------------------- -
Cir_Forward.Primer  -------------------------------------------------------------------------------- -
Cir_Rreverse.Primer -------------------------------------------------------------------------------- -

                    820 840 860 880
Genomic(mir1126)    GGCTCTCCGAGTGGCTCCTCTGTGCCGCCCCTGACTAAAAAACAAGTCTTTGATTGTGTTTTCGTTTGAAGCTGTGGAGC 880
cDNA1(mir1126)      --CTCTCCGAGTGGCTCCTCTGTGCCGCCCCTGACTAAAAAACAAGTCTTTGATTGTGTTTTCGTTTGAAGCTGTGGAGC 449
cDNA2(mir1126)      GGCTCTCCGAGTGGCTCCTCTGTGCCGCCCCTGACTAAAAAACAAGTCTTTGATTGTGTTTTCGTTTGAAGCTGTGGAGC 515
CircularRNA         -------------------------------------------------------------------------------- -
Cir_Forward.Primer  -------------------------------------------------------------------------------- -
Cir_Rreverse.Primer -------------------------------------------------------------------------------- -

                    900 920 940 960
Genomic(mir1126)    ACGCGCGAGGTGCTCGACTGATTGATTGTGTCCTTGCAAGATTCATGGACCACGATGGTGGGTCAGACCCTTTGCACGTG 960
cDNA1(mir1126)      ACGCGCGAGGTGCTCGACTGATTGATTGTGTCCTTGCAAGATTCATGGACCACGATGGTGGGTCAGACCCTTTGCACGTG 529
cDNA2(mir1126)      ACGCGCGAGGTGCTCGACTGATTGATTGTGTCCTTGCAAGATTCATGGACCACGATGGTGGGTCAGACCCTTTGCACGTG 595
CircularRNA         -------------------------------------------------------------------------------- -
Cir_Forward.Primer  -------------------------------------------------------------------------------- -
Cir_Rreverse.Primer -------------------------------------------------------------------------------- -
```

```
                                  980                 1.000               1.020               1.040
Genomic(mir1126)    TGCTCCCAGGATGGTGCTCCTGTTGTTCTTGTTTTCGCTCCGGTTGATCTTTCCCGTGGAGGTAGTTCATACGACCGGAG 1040
cDNA1(mir1126)      TGCTCCCAGGATGGTGCTCCTGTTGTTCTTGTTTTCGCTCCGGTTGATCTTTCCCGTGGAGGT----------------- 592
cDNA2(mir1126)      TGCTCCCAGGATGGTGCTCCTGTTGTTCTTGTTTTCGCTCCGGTTGATCTTTCCCGTGGAGGT----------------- 658
CircularRNA         -------------------------------------------------------------------------------- -
Cir_Forward.Primer  -------------------------------------------------------------------------------- -
Cir_Rreverse.Primer -------------------------------------------------------------------------------- -

                                  1.060               1.080               1.100               1.120
Genomic(mir1126)    TTGTTGCTTCTCTTTGTGTTTTGACTCATGGTGCTGTCACCTGTGTATTTGCTGATGTGCGTTTTGTGTTACCCAGGTTC 1120
cDNA1(mir1126)      ------------------------------------------------------------------------------TC 594
cDNA2(mir1126)      ------------------------------------------------------------------------------TC 660
CircularRNA         -------------------------------------------------------------------------------- -
Cir_Forward.Primer  -------------------------------------------------------------------------------- -
Cir_Rreverse.Primer -------------------------------------------------------------------------------- -

                                  1.140               1.160               1.180               1.200
Genomic(mir1126)    CTGTGATGTGAGCTGGCTGTTTGTTCTCATTCCCGTTTCAGGCAACGGGTGTTCCTTATAGTTTCATCCAAGTTGAGCTT 1200
cDNA1(mir1126)      CTGTGATGTGAGCTGGCTGTTTGTTCTCATTCCCGTTTCAGGCAACGGGTGTTCCTTATAGTTTCATCCAAGTTGAGCTT 674
cDNA2(mir1126)      CTGTGATGTGAGCTGGCTGTTTGTTCTCATTCCCGTTTCAGGCAACGGGTGTTCCTTATAGTTTCATCCAAGTTGAGCTT 740
CircularRNA         -------------------------------------------------------------------------------- -
Cir_Forward.Primer  -------------------------------------------------------------------------------- -
Cir_Rreverse.Primer -------------------------------------------------------------------------------- -

                                  1.220               1.240               1.260               1.280
Genomic(mir1126)    CGCTCTGCCATTGAGATGCGGTTCATCCTCTCCATGTATGCATGCGCTGCTTCGGTGCTGTTTTATTAGTTATCTTGTGG 1280
cDNA1(mir1126)      CGCTCTGCCATTGAGATGCGGTTCATCCTCTCCATG-------------------------------------------- 710
cDNA2(mir1126)      CGCTCTGCCATTGAGATGCGGTTCATCCTCTCCATG-------------------------------------------- 776
CircularRNA         -------------------------------------------------------------------------------- -
Cir_Forward.Primer  -------------------------------------------------------------------------------- -
Cir_Rreverse.Primer -------------------------------------------------------------------------------- -

                                  1.300               1.320               1.340               1.360
Genomic(mir1126)    TGGGATCGTGCTCGTCAATCATCACCTATTCCTTCTTATGTTGCACGATCGTGTTGCTCTGTTTGTGATGTAGAGCAGCG 1360
cDNA1(mir1126)      -------------------------------------------------------------------------------- 710
cDNA2(mir1126)      -------------------------------------------------------------------------------- 776
CircularRNA         -------------------------------------------------------------------------------- -
Cir_Forward.Primer  -------------------------------------------------------------------------------- -
Cir_Rreverse.Primer -------------------------------------------------------------------------------- -

                                  1.380               1.400               1.420               1.440
Genomic(mir1126)    TTGTAGGTTTGATTCTGGCGTGGCATGTTGATGGATCAGTGCTCTAAAATTTTGCATTTCTTTCTCAGGATTCCTGAGAT 1440
cDNA1(mir1126)      ---------------------------------------------------------------------ATTCCTGAGAT 721
cDNA2(mir1126)      ---------------------------------------------------------------------ATTCCTGAGAT 787
CircularRNA         -------------------------------------------------------------------------------- -
Cir_Forward.Primer  -------------------------------------------------------------------------------- -
Cir_Rreverse.Primer -------------------------------------------------------------------------------- -

                                  1.460               1.480               1.500               1.520
Genomic(mir1126)    TTGCAGCGGCGTTCATGTTCAGACTGTGACACGATAACGACATGTGAGCTCATCCTGGTTGCCAAGCAGTGGCTCTTCTT 1520
cDNA1(mir1126)      TTGCAGCGGCGTTCATGTTCAGACTGTGACACGATAACGACATGTGAGCTCATCCTGGTTGCCAAGCAGTGGCTCTTCTT 801
cDNA2(mir1126)      TTGCAGCGGCGTTCATGTTCAGACTGTGACACGATAACGACATGTGAGCTCATCCTGGTTGCCAAGCAGTGGCTCTTCTT 867
CircularRNA         -------------------------------------------------------------------------------- -
Cir_Forward.Primer  -------------------------------------------------------------------------------- -
Cir_Rreverse.Primer -------------------------------------------------------------------------------- -

                                  1.540               1.560               1.580               1.600
Genomic(mir1126)    CGTTGGTTGTCTGCTATGGCGGCGACCATTTGCTCGCCCACTATGGCGTCCGACATGATTTGGAGGTTAGAAGGTTGTAG 1600
cDNA1(mir1126)      CGTTGGTTGTCTGCTATGGCGGCGACCATTTGCTCGCCCACTATGGCGTCCGACATGATTTGGAGGTTAGAAGGTTGTAG 881
cDNA2(mir1126)      CGTTGGTTGTCTGCTATGGCGGCGACCATTTGCTCGCCCACTATGGCGTCCGACATGATTTGGAGGTTAGAAGGTTGTAG 947
CircularRNA         -------------------------------------------------------------------------------- -
Cir_Forward.Primer  -------------------------------------------------------------------------------- -
Cir_Rreverse.Primer -------------------------------------------------------------------------------- -

                                  1.620               1.640               1.660               1.680
Genomic(mir1126)    ATCATAGCACCCAGGAGACAACTGTTATTCATCACACATTTGGTGGTCGTCTGCAATCTCAGTCCCCTTTGTTTTCACAT 1680
cDNA1(mir1126)      ATCATAGCACCCAGGAGACAACTGTTATTCATCACACATTTGGTGGT--------------------------------- 928
cDNA2(mir1126)      ATCATAGCACCCAGGAGACAACTGTTATTCATCACACATTTGGTGGT--------------------------------- 994
CircularRNA         -------------------------------------------------------------------------------- -
Cir_Forward.Primer  -------------------------------------------------------------------------------- -
Cir_Rreverse.Primer -------------------------------------------------------------------------------- -

                                  1.700               1.720               1.740               1.760
Genomic(mir1126)    TTGATTTAATATTTCAATTTATAATCAGTGACCCTGCCTTTCTGATTGCTCTATTTCTAATCAGTTATAAATTCTCATGG 1760
cDNA1(mir1126)      -------------------------------------------------------------------------------- 928
cDNA2(mir1126)      -------------------------------------------------------------------------------- 994
CircularRNA         -------------------------------------------------------------------------------- -
Cir_Forward.Primer  -------------------------------------------------------------------------------- -
Cir_Rreverse.Primer -------------------------------------------------------------------------------- -

                                  1.780               1.800               1.820               1.840
Genomic(mir1126)    GATCTGTCTTTCAGTATGCATATGTTACAATTCATCAAATGCCTAACTTTTGAATGGCATGTGCAGGTTCAGTGTACTGC 1840
cDNA1(mir1126)      --------------------------------------------------------------------TCAGTGTACTGC 940
cDNA2(mir1126)      --------------------------------------------------------------------TCAGTGTACTGC 1006
CircularRNA         -------------------------------------------------------------------------------- -
Cir_Forward.Primer  -------------------------------------------------------------------------------- -
Cir_Rreverse.Primer -------------------------------------------------------------------------------- -

                                  1.860               1.880               1.900               1.920
Genomic(mir1126)    ATGTGGAATGGTCTCAAATTGCTATAAGAATATGATGGATGGACTTAGACTGTTGCAATTCTGTTCATTGTTGTAGAGTG 1920
cDNA1(mir1126)      ATGTGGAATGGTCTCAAATTGCTATAAGAATATGATGGATGGACTTAGACTGTTGCAATTCTGTTCATTGTTGTAGAGTG 1020
cDNA2(mir1126)      ATGTGGAATGGTCTCAAATTGCTATAAGAATATGATGGATGGACTTAGACTGTTGCAATTCTGTTCATTGTTGTAGAGTG 1086
CircularRNA         -------------------------------------------------------------------------------- -
Cir_Forward.Primer  -------------------------------------------------------------------------------- -
Cir_Rreverse.Primer -------------------------------------------------------------------------------- -
```

```
                                                        1.940               1.960               1.980               2.000
Genomic(mir1126)   GCTTGATGGGGACAATAAGTCCAAATGAGATGGGTAAGTCCTATTTACCGCATCTATACACTTCTGTTACTTAGCAACAA 2000
cDNA1(mir1126)     GCTTGATGGGGACAATAAGTCCAAATGAGATGGG---------------------------------------------- 1054
cDNA2(mir1126)     GCTTGATGGGGACAATAAGTCCAAATGAGATGGG---------------------------------------------- 1120
CircularRNA        -------------------------------------------------------------------------------- -
Cir_Forward.Primer -------------------------------------------------------------------------------- -
Cir_Rreverse.Primer -------------------------------------------------------------------------------- -

                                                        2.020               2.040               2.060               2.080
Genomic(mir1126)   ATGTTACAGGTTCATATTTTGATATAAGATTTGGAGATTATCTTTCTTTGCCATACATGTTATGCTTAAGTACTTAGAAA 2080
cDNA1(mir1126)     -------------------------------------------------------------------------------- 1054
cDNA2(mir1126)     -------------------------------------------------------------------------------- 1120
CircularRNA        -------------------------------------------------------------------------------- -
Cir_Forward.Primer -------------------------------------------------------------------------------- -
Cir_Rreverse.Primer -------------------------------------------------------------------------------- -

                                                        2.100               2.120               2.140               2.160
Genomic(mir1126)   TAACCAAAAGGAGAAGAATTCTTACATGATCCCTGTTACTGCTCGTTTTGCTGGTGTCTTCGCTCTATGTTTTCAGGTTG 2160
cDNA1(mir1126)     -------------------------------------------------------------------------------- 1054
cDNA2(mir1126)     -------------------------------------------------------------------------------- 1120
CircularRNA        -------------------------------------------------------------------------------- -
Cir_Forward.Primer -------------------------------------------------------------------------------- -
Cir_Rreverse.Primer -------------------------------------------------------------------------------- -

                                                        2.180               2.200               2.220               2.240
Genomic(mir1126)   AACTTCAGACCTTCACAATGCAGTTATTATTTTGCAGGATCTAAGACGGGGCATATAACACTTCTTATAATTCAGACCAC 2240
cDNA1(mir1126)     --------------------------------------ATCTAAGACGGGGCATATAACACTTCTTATAATTCAGACCAC 1096
cDNA2(mir1126)     --------------------------------------ATCTAAGACGGGGCATATAACACTTCTTATAATTCAGACCAC 1162
CircularRNA        -------------------------------------------------------------------------------- -
Cir_Forward.Primer -------------------------------------------------------------------------------- -
Cir_Rreverse.Primer -------------------------------------------------------------------------------- -

                                                        2.260               2.280               2.300               2.320
Genomic(mir1126)   ACTTGCCAATAAAGTTGAATGGGTTGGAGCAATAGTTCTAAAACTTCATTAAGTCGAATTAGGCAACCTTTTTTCTGAAT 2320
cDNA1(mir1126)     ACTTGCCAATAAAGTTGAATGGGTTGGAGCAATAGTTCTAAAACTTCATTAAGTCGAATTAGGCAACCTTTTTTCTGAAT 1176
cDNA2(mir1126)     ACTTGCCAATAAAGTTGAATGGGTTGGAGCAATAGTTCTAAAACTTCATTAAGTCGAATTAGGCAACCTTTTTTCTGAAT 1242
CircularRNA        -------------------------------------------------------------------------------- -
Cir_Forward.Primer -------------------------------------------------------------------------------- -
Cir_Rreverse.Primer -------------------------------------------------------------------------------- -

                                                        2.340               2.360               2.380               2.400
Genomic(mir1126)   GGCCTTCTTTCTCCATTGCATGTCAAATGCAATCAAGCATGCCTCTGCTAAGGGTTAGAAGAATCCAAGGAGTATGTGTC 2400
cDNA1(mir1126)     GGCCTTCTTTCTCCATTGCATGTCAAATGCAATCAAGCATGCCTCTGCTAAGGGTTAGAAGAATCCAAGGA--------- 1247
cDNA2(mir1126)     GGCCTTCTTTCTCCATTGCATGTCAAATGCAATCAAGCATGCCTCTGCTAAGGGTTAGAAGAATCCAAGGA--------- 1313
CircularRNA        -------------------------------------------------------------------------------- -
Cir_Forward.Primer -------------------------------------------------------------------------------- -
Cir_Rreverse.Primer -------------------------------------------------------------------------------- -

                                                        2.420               2.440               2.460               2.480
Genomic(mir1126)   CACGATCTCATTTATATTACTAAGAAATACCTTTTTTTCTTTCTAAATGCACACGATAGACTGTCTGATACTGAAAAATT 2480
cDNA1(mir1126)     -------------------------------------------------------------------------------- 1247
cDNA2(mir1126)     -------------------------------------------------------------------------------- 1313
CircularRNA        -------------------------------------------------------------------------------- -
Cir_Forward.Primer -------------------------------------------------------------------------------- -
Cir_Rreverse.Primer -------------------------------------------------------------------------------- -

                                                        2.500               2.520               2.540               2.560
Genomic(mir1126)   CTGGTTGCTTAGGTTTCTTCTCACCTAGCAGCTTCCATAGAGTATATTGCAACTTTAGAAATTTATTGAAAATGATAGCT 2560
cDNA1(mir1126)     -------------------------------------------------------------------------------- 1247
cDNA2(mir1126)     -------------------------------------------------------------------------------- 1313
CircularRNA        -------------------------------------------------------------------------------- -
Cir_Forward.Primer -------------------------------------------------------------------------------- -
Cir_Rreverse.Primer -------------------------------------------------------------------------------- -

                                                        2.580               2.600               2.620               2.640
Genomic(mir1126)   CTGATGTTAATGGCCAAGATGGTCCGATAGGCTTTTTTGATAATTTATCAGGAGAACGTGCAAAAGAAAATGGAGAGGGT 2640
cDNA1(mir1126)     -------------------------------------------------------------------------------- 1247
cDNA2(mir1126)     -------------------------------------------------------------------------------- 1313
CircularRNA        -------------------------------------------------------------------------------- -
Cir_Forward.Primer -------------------------------------------------------------------------------- -
Cir_Rreverse.Primer -------------------------------------------------------------------------------- -

                                                        2.660               2.680               2.700               2.720
Genomic(mir1126)   GCTCAAATGACTCAAATAGAACTACGAAATATCCGCATTAGCAAGCTAGGTATATTCAAGGAAATAATACGATGCATTGT 2720
cDNA1(mir1126)     -------------------------------------------------------------------------------- 1247
cDNA2(mir1126)     -------------------------------------------------------------------------------- 1313
CircularRNA        -------------------------------------------------------------------------------- -
Cir_Forward.Primer -------------------------------------------------------------------------------- -
Cir_Rreverse.Primer -------------------------------------------------------------------------------- -

                                                        2.740               2.760               2.780               2.800
Genomic(mir1126)   ATTTTGTTTATATGCTTATGTAGACTGACCAACTAAATTTATCCATGAAGGCCTTGGTAGATAGACATCATACATGATAG 2800
cDNA1(mir1126)     -------------------------------------------------------------------------------- 1247
cDNA2(mir1126)     -------------------------------------------------------------------------------- 1313
CircularRNA        -------------------------------------------------------------------------------- -
Cir_Forward.Primer -------------------------------------------------------------------------------- -
Cir_Rreverse.Primer -------------------------------------------------------------------------------- -

                                                        2.820               2.840               2.860               2.880
Genomic(mir1126)   AAATGTGTGGATAACATCTCAACACGGTTGTATTTATCTTAACTATGAGCTGCAATTCTTTTGGTTCCCAATTCTTCCAG 2880
cDNA1(mir1126)     -------------------------------------------------------------------------------- 1247
cDNA2(mir1126)     -------------------------------------------------------------------------------- 1313
CircularRNA        -------------------------------------------------------------------------------- -
Cir_Forward.Primer -------------------------------------------------------------------------------- -
Cir_Rreverse.Primer -------------------------------------------------------------------------------- -
```

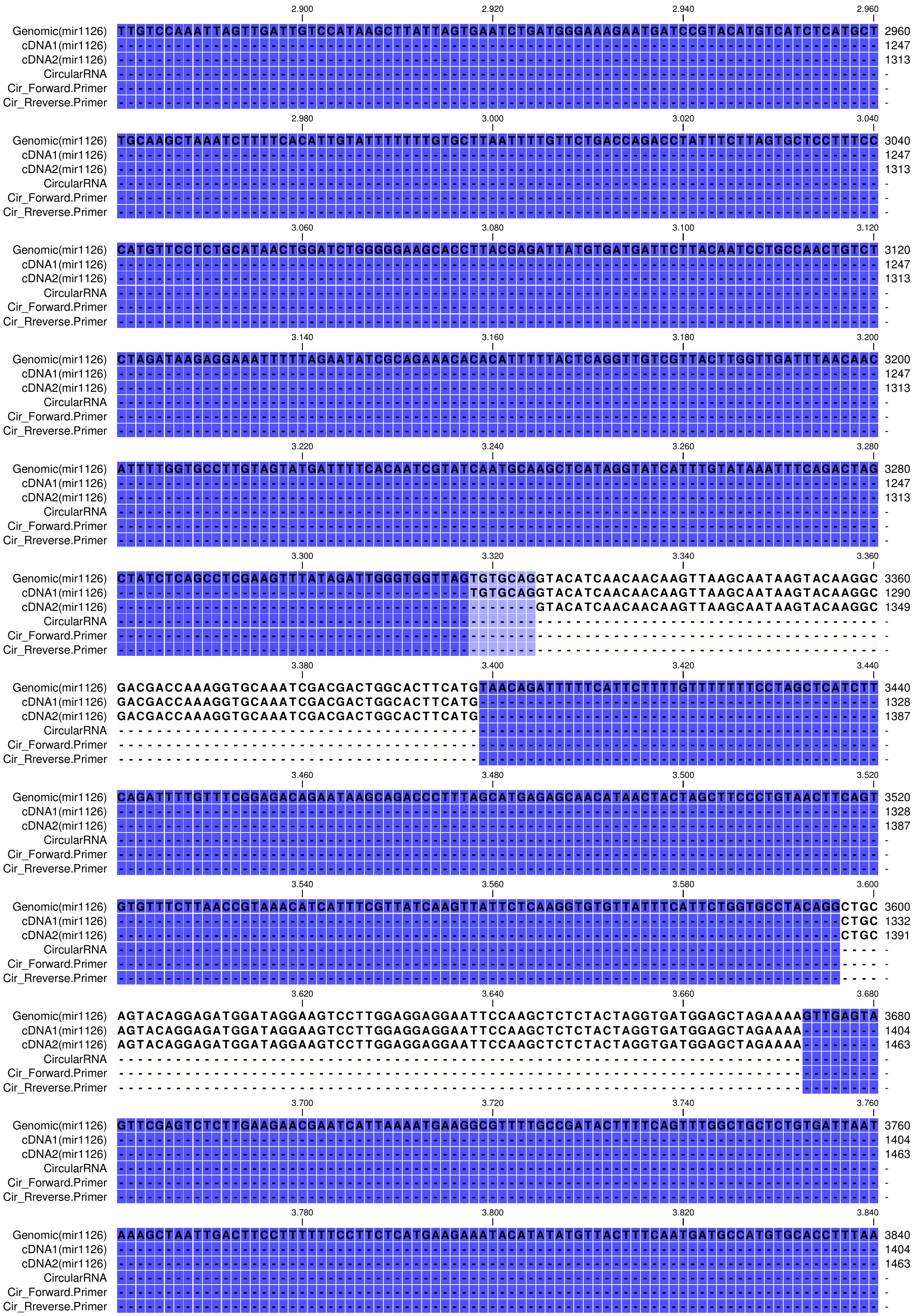

2.900 2.920 2.940 2.960
Genomic(mir1126) TTGTCCAAATTAGTTGATTGTCCATAAGCTTATTAGTGAATCTGATGGGAAAGAATGATCCGTACATGTCATCTCATGCT 2960
cDNA1(mir1126) 1247
cDNA2(mir1126) 1313
CircularRNA -
Cir_Forward.Primer -
Cir_Rreverse.Primer -
2.980 3.000 3.020 3.040
Genomic(mir1126) TGCAAGCTAAATCTTTTCACATTGTATTTTTTTGTGCTTAATTTTGTTCTGACCAGACCTATTTCTTAGTGCTCCTTTCC 3040
cDNA1(mir1126) 1247
cDNA2(mir1126) 1313
CircularRNA -
Cir_Forward.Primer -
Cir_Rreverse.Primer -
3.060 3.080 3.100 3.120
Genomic(mir1126) CATGTTCCTCTGCATAACTGGATCTGGGGGAAGCACCTTACGAGATTATGTGATGATTCTTACAATCCTGCCAACTGTCT 3120
cDNA1(mir1126) 1247
cDNA2(mir1126) 1313
CircularRNA -
Cir_Forward.Primer -
Cir_Rreverse.Primer -
3.140 3.160 3.180 3.200
Genomic(mir1126) CTAGATAAGAGGAAATTTTTAGAATATCGCAGAAACACACATTTTTACTCAGGTTGTCGTTACTTGGTTGATTTAACAAC 3200
cDNA1(mir1126) 1247
cDNA2(mir1126) 1313
CircularRNA -
Cir_Forward.Primer -
Cir_Rreverse.Primer -
3.220 3.240 3.260 3.280
Genomic(mir1126) ATTTTGGTGCCTTGTAGTATGATTTTCACAATCGTATCAATGCAAGCTCATAGGTATCATTTGTATAAATTTCAGACTAG 3280
cDNA1(mir1126) 1247
cDNA2(mir1126) 1313
CircularRNA -
Cir_Forward.Primer -
Cir_Rreverse.Primer -
3.300 3.320 3.340 3.360
Genomic(mir1126) CTATCTCAGCCTCGAAGTTTATAGATTGGGTGGTTAGTGTGCAGGTACATCAACAACAAGTTAAGCAATAAGTACAAGGC 3360
cDNA1(mir1126) TGTGCAGGTACATCAACAACAAGTTAAGCAATAAGTACAAGGC 1290
cDNA2(mir1126) GTACATCAACAACAAGTTAAGCAATAAGTACAAGGC 1349
CircularRNA -
Cir_Forward.Primer -
Cir_Rreverse.Primer -
3.380 3.400 3.420 3.440
Genomic(mir1126) GACGACCAAAGGTGCAAATCGACGACTGGCACTTCATGTAACAGATTTTTCATTCTTTTGTTTTTTTCCTAGCTCATCTT 3440
cDNA1(mir1126) GACGACCAAAGGTGCAAATCGACGACTGGCACTTCATG 1328
cDNA2(mir1126) GACGACCAAAGGTGCAAATCGACGACTGGCACTTCATG 1387
CircularRNA -
Cir_Forward.Primer -
Cir_Rreverse.Primer -
3.460 3.480 3.500 3.520
Genomic(mir1126) CAGATTTTGTTTCGGAGACAGAATAAGCAGACCCTTTAGCATGAGAGCAACATAACTACTAGCTTCCCTGTAACTTCAGT 3520
cDNA1(mir1126) 1328
cDNA2(mir1126) 1387
CircularRNA -
Cir_Forward.Primer -
Cir_Rreverse.Primer -
3.540 3.560 3.580 3.600
Genomic(mir1126) GTGTTTCTTAACCGTAAACATCATTTCGTTATCAAGTTATTCTCAAGGTGTGTTATTTCATTCTGGTGCCTACAGGCTGC 3600
cDNA1(mir1126) CTGC 1332
cDNA2(mir1126) CTGC 1391
CircularRNA -
Cir_Forward.Primer -
Cir_Rreverse.Primer -
3.620 3.640 3.660 3.680
Genomic(mir1126) AGTACAGGAGATGGATAGGAAGTCCTTGGAGGAGGAATTCCAAGCTCTCTACTAGGTGATGGAGCTAGAAAAGTTGAGTA 3680
cDNA1(mir1126) AGTACAGGAGATGGATAGGAAGTCCTTGGAGGAGGAATTCCAAGCTCTCTACTAGGTGATGGAGCTAGAAAA 1404
cDNA2(mir1126) AGTACAGGAGATGGATAGGAAGTCCTTGGAGGAGGAATTCCAAGCTCTCTACTAGGTGATGGAGCTAGAAAA 1463
CircularRNA -
Cir_Forward.Primer -
Cir_Rreverse.Primer -
3.700 3.720 3.740 3.760
Genomic(mir1126) GTTCGAGTCTCTTGAAGAACGAATCATTAAAATGAAGGCGTTTTGCCGATACTTTTCAGTTTGGCTGCTCTGTGATTAAT 3760
cDNA1(mir1126) 1404
cDNA2(mir1126) 1463
CircularRNA -
Cir_Forward.Primer -
Cir_Rreverse.Primer -
3.780 3.800 3.820 3.840
Genomic(mir1126) AAAGCTAATTGACTTCCTTTTTTCCTTCTCATGAAGAAATACATATATGTTACTTTCAATGATGCCATGTGCACCTTTAA 3840
cDNA1(mir1126) 1404
cDNA2(mir1126) 1463
CircularRNA -
Cir_Forward.Primer -
Cir_Rreverse.Primer -

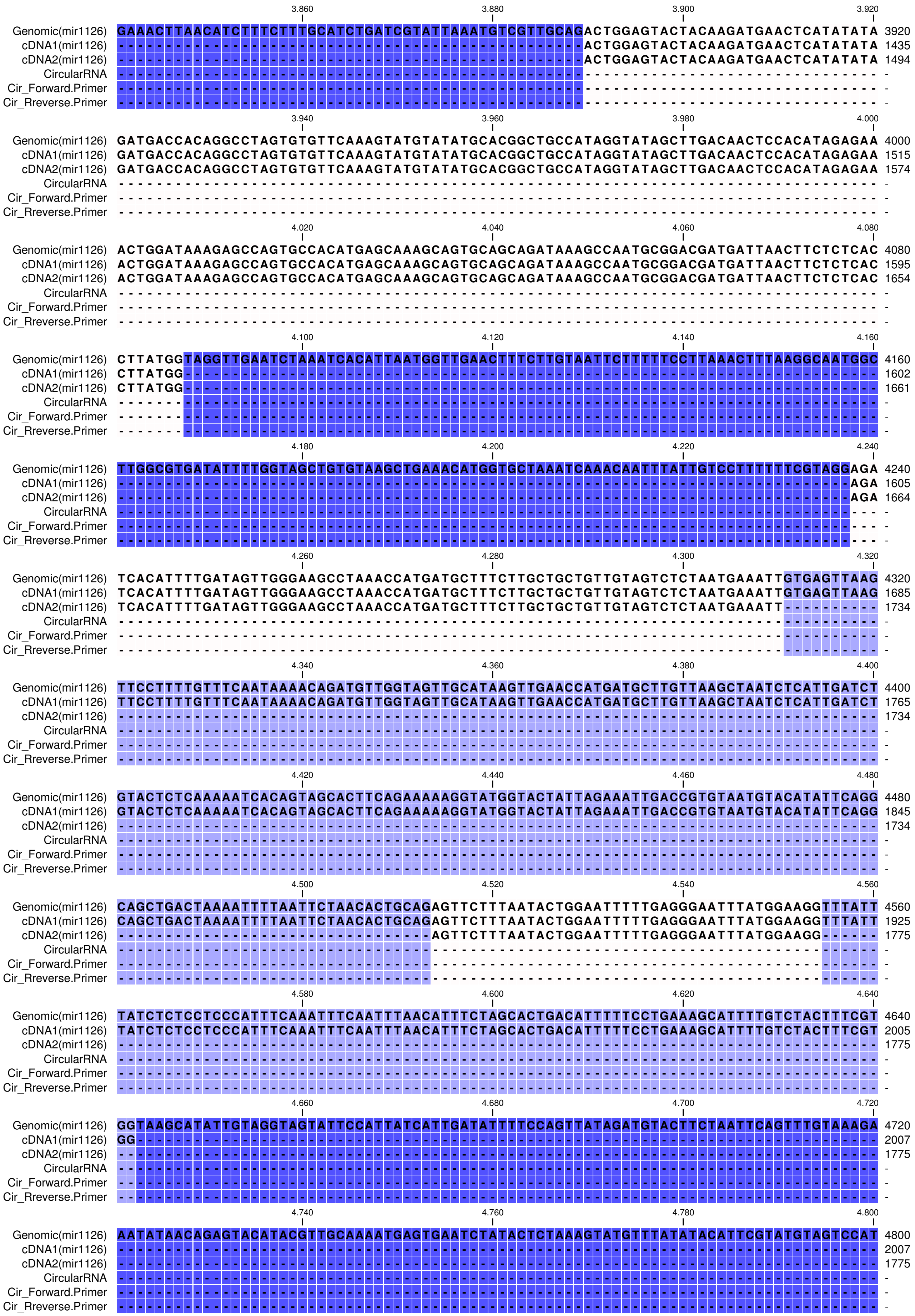
Genomic(mir1126)
cDNA1(mir1126)
cDNA2(mir1126)
CircularRNA
Cir_Forward.Primer
Cir_Rreverse.Primer

```
                          4.820               4.840               4.860               4.880
Genomic(mir1126)   AGTGAAATTCTAAAAAGACTTATATTTAGGAACGGAGGGAGTACTTATGTTCCCACTGAATCGACTGAATACTAAGGGCA 4880
cDNA1(mir1126)     -------------------------------------------------------------------------------- 2007
cDNA2(mir1126)     -------------------------------------------------------------------------------- 1775
CircularRNA        -------------------------------------------------------------------------------- -
Cir_Forward.Primer -------------------------------------------------------------------------------- -
Cir_Rreverse.Primer -------------------------------------------------------------------------------- -

                          4.900               4.920               4.940               4.960
Genomic(mir1126)   TCTTTGATTCACAGGCTCGAACACTTTCTCCAGTATGGAAATGAAGCATGCTGTGGGGAGGATCTAATAAAAATTGATCT 4960
cDNA1(mir1126)     -------------------------------------------------------------------------------- 2007
cDNA2(mir1126)     -------------------------------------------------------------------------------- 1775
CircularRNA        -------------------------------------------------------------------------------- -
Cir_Forward.Primer -------------------------------------------------------------------------------- -
Cir_Rreverse.Primer -------------------------------------------------------------------------------- -

                          4.980               5.000               5.020               5.040
Genomic(mir1126)   AAAATAAAGAAAGAGCAATCTTTGGATGACAATGAGATTCATGATGGAGACTCAATGCAGATTGTCTTTCCTTTCCATTT 5040
cDNA1(mir1126)     -------------------------------------------------------------------------------- 2007
cDNA2(mir1126)     -------------------------------------------------------------------------------- 1775
CircularRNA        -------------------------------------------------------------------------------- -
Cir_Forward.Primer -------------------------------------------------------------------------------- -
Cir_Rreverse.Primer -------------------------------------------------------------------------------- -

                          5.060               5.080               5.100               5.120
Genomic(mir1126)   GTTTCTTATTCTTTTCATCTAACTCCCTCTGTCCCAAAATAAGTGTCGCTGATCTATTATAAATTTTGTACTAATTTAAT 5120
cDNA1(mir1126)     -------------------------------------------------------------------------------- 2007
cDNA2(mir1126)     -------------------------------------------------------------------------------- 1775
CircularRNA        -------------------------------------------------------------------------------- -
Cir_Forward.Primer -------------------------------------------------------------------------------- -
Cir_Rreverse.Primer -------------------------------------------------------------------------------- -

                          5.140               5.160               5.180               5.200
Genomic(mir1126)   ACTAAATCTGCGACACTTATTCTGGGACGAAGAGAGTATAATGTTTTGTGCTGAGTTAGGTGGAAGGTCGAGACGACCTC 5200
cDNA1(mir1126)     -------------------------------------------------------------------------------- 2007
cDNA2(mir1126)     -------------------------------------------------------------------------------- 1775
CircularRNA        -------------------------------------------------------------------------------- -
Cir_Forward.Primer -------------------------------------------------------------------------------- -
Cir_Rreverse.Primer -------------------------------------------------------------------------------- -

                          5.220               5.240               5.260               5.280
Genomic(mir1126)   ACCTACATAAATGTTCCTTTGTTCTACTCCCTCTGTTCCTAAATATTTGTCTTTTTAAAGATTCAACTACGGACTACATA 5280
cDNA1(mir1126)     -------------------------------------------------------------------------------- 2007
cDNA2(mir1126)     -------------------------------------------------------------------------------- 1775
CircularRNA        -------------------------------------------------------------------------------- -
Cir_Forward.Primer -------------------------------------------------------------------------------- -
Cir_Rreverse.Primer -------------------------------------------------------------------------------- -

                          5.300               5.320               5.340               5.360
Genomic(mir1126)   CGGAACAAAATGAGTGAACTTACACTCTGAATTATGTCTATATATATATCCATATGTAGCCCATAGGGAAATCTCTAAAA 5360
cDNA1(mir1126)     -------------------------------------------------------------------------------- 2007
cDNA2(mir1126)     -------------------------------------------------------------------------------- 1775
CircularRNA        -------------------------------------------------------------------------------- -
Cir_Forward.Primer -------------------------------------------------------------------------------- -
Cir_Rreverse.Primer -------------------------------------------------------------------------------- -

                          5.380               5.400               5.420               5.440
Genomic(mir1126)   AAACAAATATTTAGGAACGGAGGGAGTATTTATTAATGTATGGATGCTTACCGACCCTAAATATATTTGGTTAAAATGTT 5440
cDNA1(mir1126)     -------------------------------------------------------------------------------- 2007
cDNA2(mir1126)     -------------------------------------------------------------------------------- 1775
CircularRNA        -------------------------------------------------------------------------------- -
Cir_Forward.Primer -------------------------------------------------------------------------------- -
Cir_Rreverse.Primer -------------------------------------------------------------------------------- -

                          5.460               5.480               5.500               5.520
Genomic(mir1126)   TCCCTATGAAGTTTCTATACTATATAAGAAAGTTGGTTTGAAATATTTGAGGTCATATTCTATTTTACAGGTATAGTTGC 5520
cDNA1(mir1126)     -------------------------------------------------------------------------------- 2007
cDNA2(mir1126)     -------------------------------------------------------------------------------- 1775
CircularRNA        -------------------------------------------------------------------------------- -
Cir_Forward.Primer -------------------------------------------------------------------------------- -
Cir_Rreverse.Primer -------------------------------------------------------------------------------- -

                          5.540               5.560               5.580               5.600
Genomic(mir1126)   ATAGCCGATTCAGTTGCGTTAGTTCAGAACAAGTGTTTGTATGTACATGAAAGATAGCATATTCATTTATACATTCAAAT 5600
cDNA1(mir1126)     -------------------------------------------------------------------------------- 2007
cDNA2(mir1126)     -------------------------------------------------------------------------------- 1775
CircularRNA        -------------------------------------------------------------------------------- -
Cir_Forward.Primer -------------------------------------------------------------------------------- -
Cir_Rreverse.Primer -------------------------------------------------------------------------------- -

                          5.620               5.640               5.660               5.680
Genomic(mir1126)   AATTGTACATAAGCGCCATTAAGGATTATAAGCAAGATAGGTGCATTCCCTCCGTTCACATATATAAGATTTTCCACCTT 5680
cDNA1(mir1126)     -------------------------------------------------------------------------------- 2007
cDNA2(mir1126)     -------------------------------------------------------------------------------- 1775
CircularRNA        -------------------------------------------------------------------------------- -
Cir_Forward.Primer -------------------------------------------------------------------------------- -
Cir_Rreverse.Primer -------------------------------------------------------------------------------- -

                          5.700               5.720               5.740               5.760
Genomic(mir1126)   TTTCCTGATTCGGATGTATATAGATGCATTTAGTGTGTTTGTTCATCCATTTCAGTCTGTATGTTGTTCATATTGAAATA 5760
cDNA1(mir1126)     -------------------------------------------------------------------------------- 2007
cDNA2(mir1126)     -------------------------------------------------------------------------------- 1775
CircularRNA        -------------------------------------------------------------------------------- -
Cir_Forward.Primer -------------------------------------------------------------------------------- -
Cir_Rreverse.Primer -------------------------------------------------------------------------------- -
```

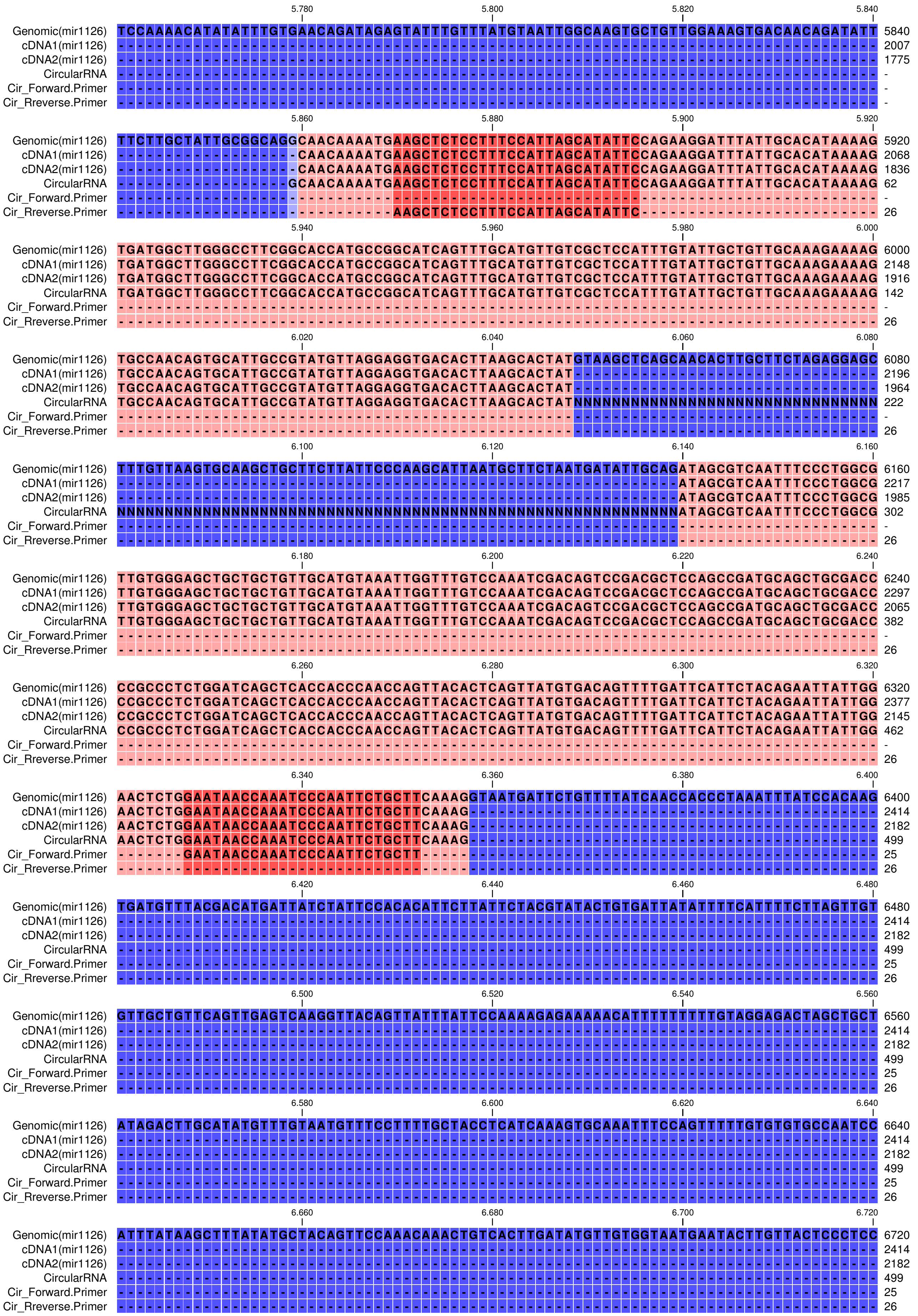

Genomic(mir1126)
cDNA1(mir1126)
cDNA2(mir1126)
CircularRNA
Cir_Forward.Primer
Cir_Rreverse.Primer
TCCAAAACATATATTTGTGAACAGATAGAGTATTTGTTTATGTAATTGGCAAGTGCTGTTGGAAAGTGACAACAGATATT 5840
TTCTTGCTATTGCGGCAGGCAACAAAATGAAGCTCTCCTTTCCATTAGCATATTCCAGAAGGATTTATTGCACATAAAAG 5920
AAGCTCTCCTTTCCATTAGCATATTC 26
TGATGGCTTGGGCCTTCGGCACCATGCCGGCATCAGTTTGCATGTTGTCGCTCCATTTGTATTGCTGTTGCAAAGAAAAG 6000
TGCCAACAGTGCATTGCCGTATGTTAGGAGGTGACACTTAAGCACTATGTAAGCTCAGCAACACTTGCTTCTAGAGGAGC 6080
TTTGTTAAGTGCAAGCTGCTTCTTATTCCCAAGCATTAATGCTTCTAATGATATTGCAGATAGCGTCAATTTCCCTGGCG 6160
TTGTGGGAGCTGCTGCTGTTGCATGTAAATTGGTTTGTCCAAATCGACAGTCCGACGCTCCAGCCGATGCAGCTGCGACC 6240
CCGCCCTCTGGATCAGCTCACCACCCAACCAGTTACACTCAGTTATGTGACAGTTTTGATTCATTCTACAGAATTATTGG 6320
AACTCTGGAATAACCAAATCCCAATTCTGCTTCAAAGGTAATGATTCTGTTTTATCAACCACCCTAAATTTATCCACAAG 6400
GAATAACCAAATCCCAATTCTGCTT 25
TGATGTTTACGACATGATTATCTATTCCACACATTCTTATTCTACGTATACTGTGATTATATTTTCATTTTCTTAGTTGT 6480
GTTGCTGTTCAGTTGAGTCAAGGTTACAGTTATTTATTCCAAAAGAGAAAAACATTTTTTTTTTGTAGGAGACTAGCTGCT 6560
ATAGACTTGCATATGTTTGTAATGTTTCCTTTTGCTACCTCATCAAAGTGCAAATTTCCAGTTTTTGTGTGTGCCAATCC 6640
ATTTATAAGCTTTATATGCTACAGTTCCAAACAAACTGTCACTTGATATGTTGTGGTAATGAATACTTGTTACTCCCTCC 6720

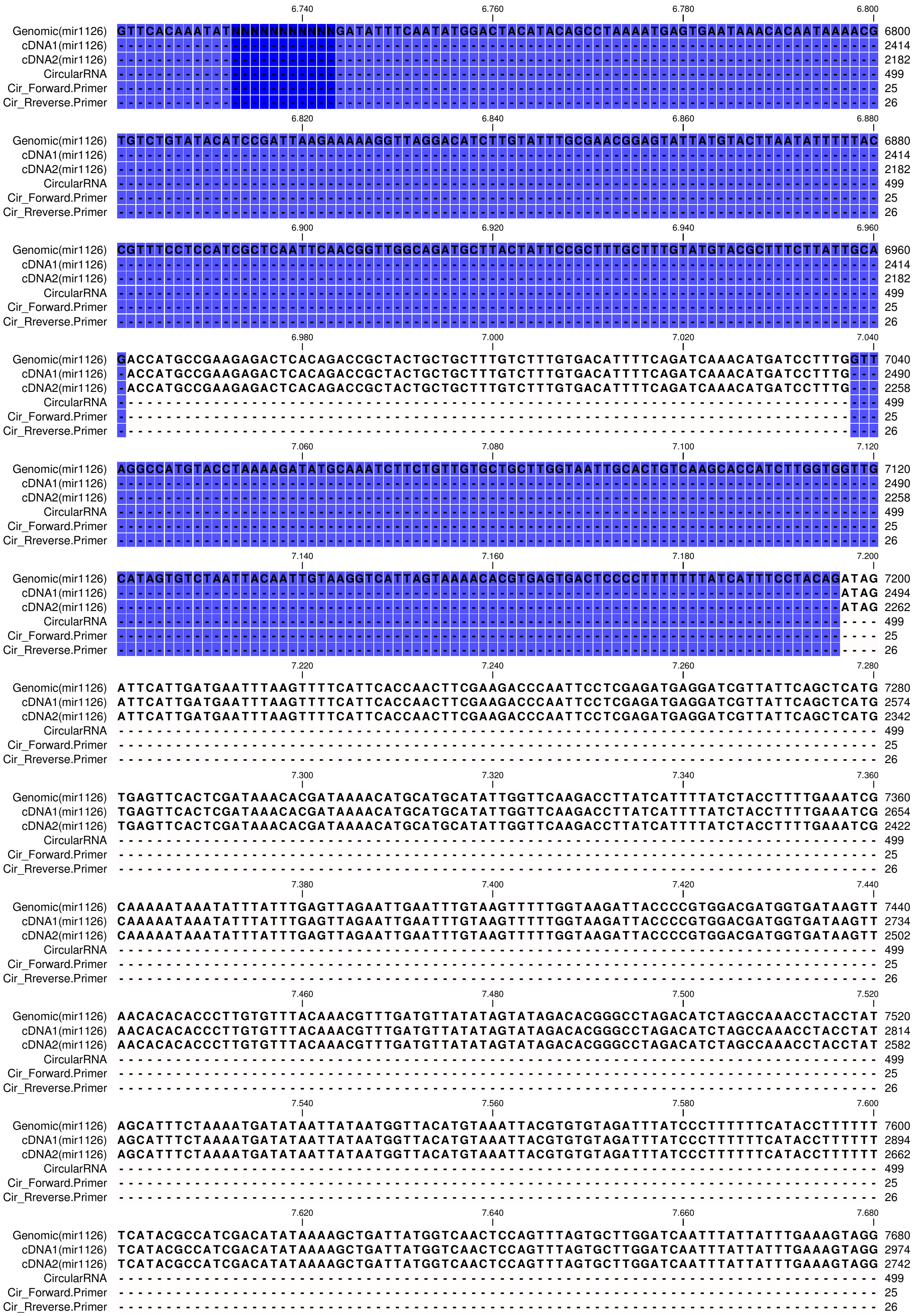

6.740 6.760 6.780 6.800
Genomic(mir1126) GTTCACAAATATNNNNNNNNNNNGATATTTCAATATGGACTACATACAGCCTAAAATGAGTGAATAAACACAATAAAACG 6800
cDNA1(mir1126) -- 2414
cDNA2(mir1126) -- 2182
CircularRNA -- 499
Cir_Forward.Primer -- 25
Cir_Rreverse.Primer -- 26
6.820 6.840 6.860 6.880
Genomic(mir1126) TGTCTGTATACATCCGATTAAGAAAAAGGTTAGGACATCTTGTATTTGCGAACGGAGTATTATGTACTTAATATTTTTAC 6880
cDNA1(mir1126) -- 2414
cDNA2(mir1126) -- 2182
CircularRNA -- 499
Cir_Forward.Primer -- 25
Cir_Rreverse.Primer -- 26
6.900 6.920 6.940 6.960
Genomic(mir1126) CGTTTCCTCCATCGCTCAATTCAACGGTTGGCAGATGCTTACTATTCCGCTTTGCTTTGTATGTACGCTTTCTTATTGCA 6960
cDNA1(mir1126) -- 2414
cDNA2(mir1126) -- 2182
CircularRNA -- 499
Cir_Forward.Primer -- 25
Cir_Rreverse.Primer -- 26
6.980 7.000 7.020 7.040
Genomic(mir1126) GACCATGCCGAAGAGACTCACAGACCGCTACTGCTGCTTTGTCTTTGTGACATTTTCAGATCAAACATGATCCTTTGGTT 7040
cDNA1(mir1126) -ACCATGCCGAAGAGACTCACAGACCGCTACTGCTGCTTTGTCTTTGTGACATTTTCAGATCAAACATGATCCTTTG--- 2490
cDNA2(mir1126) -ACCATGCCGAAGAGACTCACAGACCGCTACTGCTGCTTTGTCTTTGTGACATTTTCAGATCAAACATGATCCTTTG--- 2258
CircularRNA -- 499
Cir_Forward.Primer -- 25
Cir_Rreverse.Primer -- 26
7.060 7.080 7.100 7.120
Genomic(mir1126) AGGCCATGTACCTAAAAGATATGCAAATCTTCTGTTGTGCTGCTTGGTAATTGCACTGTCAAGCACCATCTTGGTGGTTG 7120
cDNA1(mir1126) -- 2490
cDNA2(mir1126) -- 2258
CircularRNA -- 499
Cir_Forward.Primer -- 25
Cir_Rreverse.Primer -- 26
7.140 7.160 7.180 7.200
Genomic(mir1126) CATAGTGTCTAATTACAATTGTAAGGTCATTAGTAAAACACGTGAGTGACTCCCCTTTTTTTATCATTTCCTACAGATAG 7200
cDNA1(mir1126) --ATAG 2494
cDNA2(mir1126) --ATAG 2262
CircularRNA -- 499
Cir_Forward.Primer -- 25
Cir_Rreverse.Primer -- 26
7.220 7.240 7.260 7.280
Genomic(mir1126) ATTCATTGATGAATTTAAGTTTTCATTCACCAACTTCGAAGACCCAATTCCTCGAGATGAGGATCGTTATTCAGCTCATG 7280
cDNA1(mir1126) ATTCATTGATGAATTTAAGTTTTCATTCACCAACTTCGAAGACCCAATTCCTCGAGATGAGGATCGTTATTCAGCTCATG 2574
cDNA2(mir1126) ATTCATTGATGAATTTAAGTTTTCATTCACCAACTTCGAAGACCCAATTCCTCGAGATGAGGATCGTTATTCAGCTCATG 2342
CircularRNA -- 499
Cir_Forward.Primer -- 25
Cir_Rreverse.Primer -- 26
7.300 7.320 7.340 7.360
Genomic(mir1126) TGAGTTCACTCGATAAACACGATAAAACATGCATGCATATTGGTTCAAGACCTTATCATTTTATCTACCTTTTGAAATCG 7360
cDNA1(mir1126) TGAGTTCACTCGATAAACACGATAAAACATGCATGCATATTGGTTCAAGACCTTATCATTTTATCTACCTTTTGAAATCG 2654
cDNA2(mir1126) TGAGTTCACTCGATAAACACGATAAAACATGCATGCATATTGGTTCAAGACCTTATCATTTTATCTACCTTTTGAAATCG 2422
CircularRNA -- 499
Cir_Forward.Primer -- 25
Cir_Rreverse.Primer -- 26
7.380 7.400 7.420 7.440
Genomic(mir1126) CAAAAATAAATATTTATTTGAGTTAGAATTGAATTTGTAAGTTTTTGGTAAGATTACCCCGTGGACGATGGTGATAAGTT 7440
cDNA1(mir1126) CAAAAATAAATATTTATTTGAGTTAGAATTGAATTTGTAAGTTTTTGGTAAGATTACCCCGTGGACGATGGTGATAAGTT 2734
cDNA2(mir1126) CAAAAATAAATATTTATTTGAGTTAGAATTGAATTTGTAAGTTTTTGGTAAGATTACCCCGTGGACGATGGTGATAAGTT 2502
CircularRNA -- 499
Cir_Forward.Primer -- 25
Cir_Rreverse.Primer -- 26
7.460 7.480 7.500 7.520
Genomic(mir1126) AACACACACCCTTGTGTTTACAAACGTTTGATGTTATATAGTATAGACACGGGCCTAGACATCTAGCCAAACCTACCTAT 7520
cDNA1(mir1126) AACACACACCCTTGTGTTTACAAACGTTTGATGTTATATAGTATAGACACGGGCCTAGACATCTAGCCAAACCTACCTAT 2814
cDNA2(mir1126) AACACACACCCTTGTGTTTACAAACGTTTGATGTTATATAGTATAGACACGGGCCTAGACATCTAGCCAAACCTACCTAT 2582
CircularRNA -- 499
Cir_Forward.Primer -- 25
Cir_Rreverse.Primer -- 26
7.540 7.560 7.580 7.600
Genomic(mir1126) AGCATTTCTAAAATGATATAATTATAATGGTTACATGTAAATTACGTGTGTAGATTTATCCCTTTTTTCATACCTTTTTT 7600
cDNA1(mir1126) AGCATTTCTAAAATGATATAATTATAATGGTTACATGTAAATTACGTGTGTAGATTTATCCCTTTTTTCATACCTTTTTT 2894
cDNA2(mir1126) AGCATTTCTAAAATGATATAATTATAATGGTTACATGTAAATTACGTGTGTAGATTTATCCCTTTTTTCATACCTTTTTT 2662
CircularRNA -- 499
Cir_Forward.Primer -- 25
Cir_Rreverse.Primer -- 26
7.620 7.640 7.660 7.680
Genomic(mir1126) TCATACGCCATCGACATATAAAAGCTGATTATGGTCAACTCCAGTTTAGTGCTTGGATCAATTTATTATTTGAAAGTAGG 7680
cDNA1(mir1126) TCATACGCCATCGACATATAAAAGCTGATTATGGTCAACTCCAGTTTAGTGCTTGGATCAATTTATTATTTGAAAGTAGG 2974
cDNA2(mir1126) TCATACGCCATCGACATATAAAAGCTGATTATGGTCAACTCCAGTTTAGTGCTTGGATCAATTTATTATTTGAAAGTAGG 2742
CircularRNA -- 499
Cir_Forward.Primer -- 25
Cir_Rreverse.Primer -- 26

```
                    7.700               7.720               7.740               7.760
Genomic(mir1126)    GGTTAAAGTTTGCCAAGAAAAAAGCTGATTATTACTTGAGATTGGTGGAGTAGACTATATCCCACCCAGTCCTGTTTCCA 7760
cDNA1(mir1126)      GGTTAAAGTTTGCCAAGAAAAAAGCTGATTATTACTTGAGATTGGTGGAGTAGACTATATCCCACCCAGTCCTGTTTCCA 3054
cDNA2(mir1126)      GGTTAAAGTTTGCCAAGAAAAAAGCTGATTATTACTTGAGATTGGTGGAGTAGACTATATCCCACCCAGTCCTGTTTCCA 2822
CircularRNA         -------------------------------------------------------------------------------- 499
Cir_Forward.Primer  -------------------------------------------------------------------------------- 25
Cir_Rreverse.Primer -------------------------------------------------------------------------------- 26

                    7.780               7.800               7.820               7.840
Genomic(mir1126)    TCAAAATACACAAACCATTTTGGTTGTGGGCCTATCTCATCTTAGCGGTTTTGTTTGGGCCTCATTCCACTAGTACACTA 7840
cDNA1(mir1126)      TCAAAATACACAAACCATTTTGGTTGTGGGCCTATCTCATCTTAGCGGTTTTGTTTGGGCCTCATTCCACTAGTACACTA 3134
cDNA2(mir1126)      TCAAAATACACAAACCATTTTGGTTGTGGGCCTATCTCATCTTAGCGGTTTTGTTTGGGCCTCATTCCACTAGTACACTA 2902
CircularRNA         -------------------------------------------------------------------------------- 499
Cir_Forward.Primer  -------------------------------------------------------------------------------- 25
Cir_Rreverse.Primer -------------------------------------------------------------------------------- 26

                    7.860               7.880               7.900               7.920
Genomic(mir1126)    GTCATCGTCTCATATCCCAAATTTCCCTTAGTCTCACTCCCAATCACTTCACGATCCGAGGAAGGGAATGCTAGCAGCCA 7920
cDNA1(mir1126)      GTCATCGTCTCATATCCCAAATTTCCCTTAGTCTCACTCCCAATCACTTCACGATCCGAGGAAGGGAATGCTAGCAGCCA 3214
cDNA2(mir1126)      GTCATCGTCTCATATCCCAAATTTCCCTTAGTCTCACTCCCAATCACTTCACGATCCGAGGAAGGGAATGCTAGCAGCCA 2982
CircularRNA         -------------------------------------------------------------------------------- 499
Cir_Forward.Primer  -------------------------------------------------------------------------------- 25
Cir_Rreverse.Primer -------------------------------------------------------------------------------- 26

                    7.940               7.960               7.980               8.000
Genomic(mir1126)    TGGCGATAGTGTCTCCTTCGTCCGTTCATGCTACTTGAGAGGATGATGACTCTGGCGACACCGCGTGAGAGACATCTATG 8000
cDNA1(mir1126)      TGGCGATAGTGTCTCCTTCGTCCGTTCATGCTACTTGAGAGGATGATGACTCTGGCGACACCGCGTGAGAGACATCTATG 3294
cDNA2(mir1126)      TGGCGATAGTGTCTCCTTCGTCCGTTCATGCTACTTGAGAGGATGATGACTCTGGCGACACCGCGTGAGAGACATCTATG 3062
CircularRNA         -------------------------------------------------------------------------------- 499
Cir_Forward.Primer  -------------------------------------------------------------------------------- 25
Cir_Rreverse.Primer -------------------------------------------------------------------------------- 26

                    8.020               8.040               8.060               8.080
Genomic(mir1126)    GAAGCGAGGTTGCCGCCATCCTTGTTGCGACGACAATGCCACGAAGCATATATAGTGGAGGGTCGAGCGGGATGGTGGTG 8080
cDNA1(mir1126)      GAAGCGAGGTTGCCGCCATCCTTGTTGCGACGACAATGCCACGAAGCATATATAGTGGAGGGTCGAGCGGGATGGTGGTG 3374
cDNA2(mir1126)      GAAGCGAGGTTGCCGCCATCCTTGTTGCGACGACAATGCCACGAAGCATATATAGTGGAGGGTCGAGCGGGATGGTGGTG 3142
CircularRNA         -------------------------------------------------------------------------------- 499
Cir_Forward.Primer  -------------------------------------------------------------------------------- 25
Cir_Rreverse.Primer -------------------------------------------------------------------------------- 26

                    8.100               8.120               8.140               8.160
Genomic(mir1126)    TGGCAGCATCGCGGAAGGAGGTGCCGGCCATTCATAAGGATCTTAGCTGATTTATAAGTGTGAGACTCAACAATGTACAT 8160
cDNA1(mir1126)      TGGCAGCATCGCGGAAGGAGGTGCCGGCCATTCATAAGGATCTTAGCTGATTTATAAGTGTGAGACTCAACAATGTACAT 3454
cDNA2(mir1126)      TGGCAGCATCGCGGAAGGAGGTGCCGGCCATTCATAAGGATCTTAGCTGATTTATAAGTGTGAGACTCAACAATGTACAT 3222
CircularRNA         -------------------------------------------------------------------------------- 499
Cir_Forward.Primer  -------------------------------------------------------------------------------- 25
Cir_Rreverse.Primer -------------------------------------------------------------------------------- 26

                    8.180               8.200               8.220               8.240
Genomic(mir1126)    ACTAACGAGAAATTAAGTGTGAGACTCAACAAGGTACATACTAACGAGAAATTATTTAATCCTGCACATTGGAGCATACG 8240
cDNA1(mir1126)      ACTAACGAGAAATTAAGTGTGAGACTCAACAAGGTACATACTAACGAGAAATTATTTAATCCTGCACATTGGAGCATACG 3534
cDNA2(mir1126)      ACTAACGAGAAATTAAGTGTGAGACTCAACAAGGTACATACTAACGAGAAATTATTTAATCCTGCACATTGGAGCATACG 3302
CircularRNA         -------------------------------------------------------------------------------- 499
Cir_Forward.Primer  -------------------------------------------------------------------------------- 25
Cir_Rreverse.Primer -------------------------------------------------------------------------------- 26

                    8.260               8.280               8.300               8.320
Genomic(mir1126)    ACACCAATATGCTTGGTGAGCTATGCTTCACAAGGCCATGCGCAATACTGATAGCACGTGTACTCTCATAACAAAGGAGT 8320
cDNA1(mir1126)      ACACCAATATGCTTGGTGAGCTATGCTTCACAAGGCCATGCGCAATACTGATAGCACGTGTACTCTCATAACAAAGGAGT 3614
cDNA2(mir1126)      ACACCAATATGCTTGGTGAGCTATGCTTCACAAGGCCATGCGCAATACTGATAGCACGTGTACTCTCATAACAAAGGAGT 3382
CircularRNA         -------------------------------------------------------------------------------- 499
Cir_Forward.Primer  -------------------------------------------------------------------------------- 25
Cir_Rreverse.Primer -------------------------------------------------------------------------------- 26

                    8.340               8.360               8.380               8.400
Genomic(mir1126)    TGGTGTAGTGACGAAGACATAAAAAAATGCTTAATCAACCATCGTATCCTTTTGTTGTCAAACGGGCCATAGTTTGCAAC 8400
cDNA1(mir1126)      TGGTGTAGTGACGAAGACATAAAAAAATGCTTAATCAACCATCGTATCCTTTTGTTGTCAAACGGGCCATAGTTTGCAAC 3694
cDNA2(mir1126)      TGGTGTAGTGACGAAGACATAAAAAAATGCTTAATCAACCATCGTATCCTTTTGTTGTCAAACGGGCCATAGTTTGCAAC 3462
CircularRNA         -------------------------------------------------------------------------------- 499
Cir_Forward.Primer  -------------------------------------------------------------------------------- 25
Cir_Rreverse.Primer -------------------------------------------------------------------------------- 26

                    8.420               8.440               8.460               8.480
Genomic(mir1126)    AAAGCCATGTTTCTTTGTCTTTTAGGCAATGAGAAAACCATCAAGAAAAACACAGTAAATAGAAAGGGACAAGCGAACTT 8480
cDNA1(mir1126)      AAAGCCATGTTTCTTTGTCTTTTA-------------------------------------------------------- 3718
cDNA2(mir1126)      AAAGCCATGTTTCTTTGTCTTTTA-------------------------------------------------------- 3486
CircularRNA         -------------------------------------------------------------------------------- 499
Cir_Forward.Primer  -------------------------------------------------------------------------------- 25
Cir_Rreverse.Primer -------------------------------------------------------------------------------- 26

                    8.500               8.520               8.540               8.560
Genomic(mir1126)    GAGCATAGGAAGAATAGCGAGATGGTGAGAGATAATTCCATGAAGATGTCATAGAACACGGGGGAGGTGACTCAAGTGGA 8560
cDNA1(mir1126)      -------------------------------------------------------------------------------- 3718
cDNA2(mir1126)      -------------------------------------------------------------------------------- 3486
CircularRNA         -------------------------------------------------------------------------------- 499
Cir_Forward.Primer  -------------------------------------------------------------------------------- 25
Cir_Rreverse.Primer -------------------------------------------------------------------------------- 26

                    8.580               8.600               8.620               8.640
Genomic(mir1126)    TCGAAGTGGGAGCAAAGACAAATTGACTTGGAACGAGAACGCGACATGAAATGCCTGGATGACATCTAAATACACAAGAC 8640
cDNA1(mir1126)      -------------------------------------------------------------------------------- 3718
cDNA2(mir1126)      -------------------------------------------------------------------------------- 3486
CircularRNA         -------------------------------------------------------------------------------- 499
Cir_Forward.Primer  -------------------------------------------------------------------------------- 25
Cir_Rreverse.Primer -------------------------------------------------------------------------------- 26
```

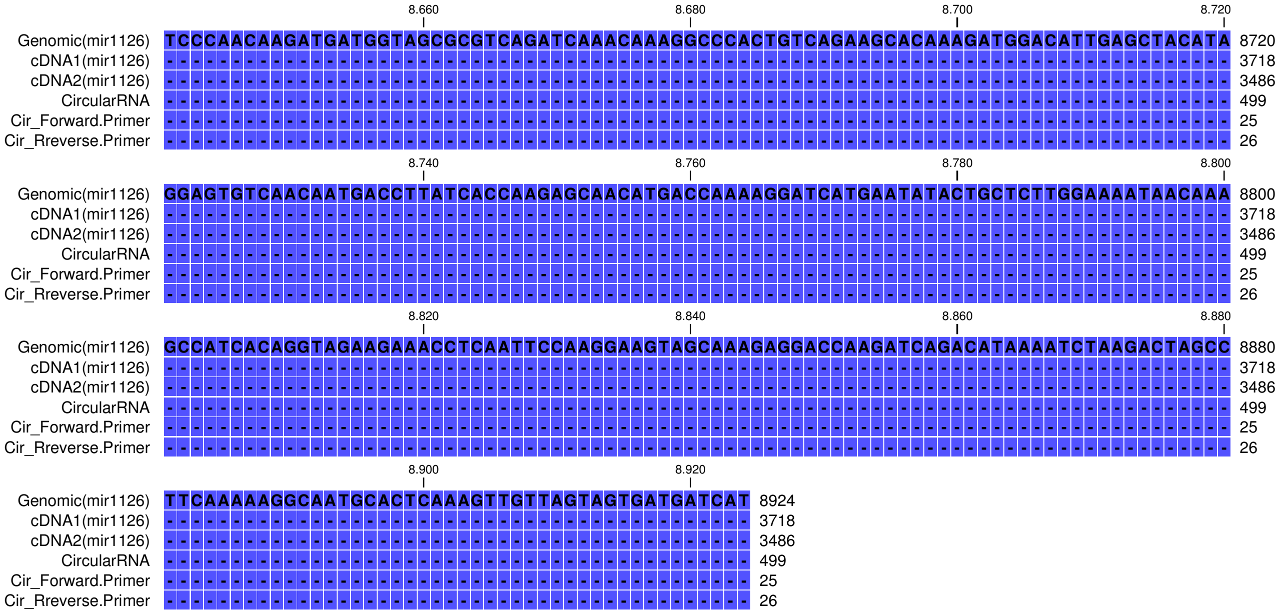
8.660 8.680 8.700 8.720
Genomic(mir1126) TCCCAACAAGATGATGGTAGCGCGTCAGATCAAACAAAGGCCCACTGTCAGAAGCACAAAGATGGACATTGAGCTACATA 8720
cDNA1(mir1126) -- 3718
cDNA2(mir1126) -- 3486
CircularRNA -- 499
Cir_Forward.Primer -- 25
Cir_Rreverse.Primer -- 26
8.740 8.760 8.780 8.800
Genomic(mir1126) GGAGTGTCAACAATGACCTTATCACCAAGAGCAACATGACCAAAAGGATCATGAATATACTGCTCTTGGAAAATAACAAA 8800
cDNA1(mir1126) -- 3718
cDNA2(mir1126) -- 3486
CircularRNA -- 499
Cir_Forward.Primer -- 25
Cir_Rreverse.Primer -- 26
8.820 8.840 8.860 8.880
Genomic(mir1126) GCCATCACAGGTAGAAGAAACCTCAATTCCAAGGAAGTAGCAAAGAGGACCAAGATCAGACATAAAATCTAAGACTAGCC 8880
cDNA1(mir1126) -- 3718
cDNA2(mir1126) -- 3486
CircularRNA -- 499
Cir_Forward.Primer -- 25
Cir_Rreverse.Primer -- 26
8.900 8.920
Genomic(mir1126) TTCAAAAAGGCAATGCACTCAAAGTTGTTAGTAGTGATGATCAT 8924
cDNA1(mir1126) -- 3718
cDNA2(mir1126) -- 3486
CircularRNA -- 499
Cir_Forward.Primer -- 25
Cir_Rreverse.Primer -- 26

Real-Time PCR for the junction region of microRNA1126_circular RNA

(ID: Ch2:467982888-467983386)

By divergent primers on genomic DNA

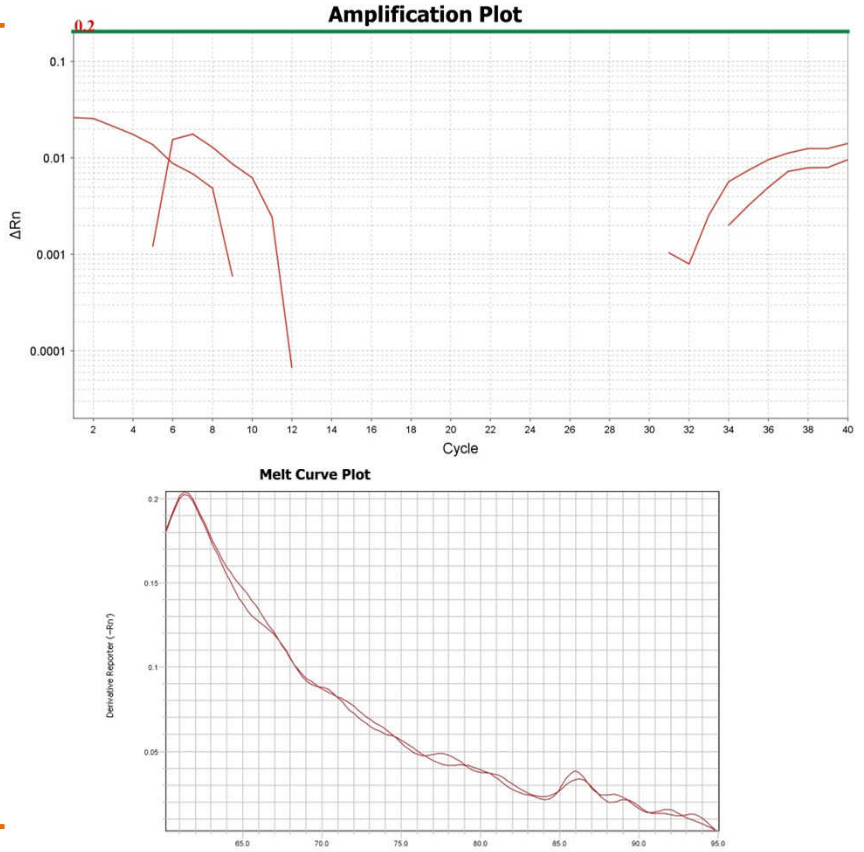

By divergent primers on cDNAs

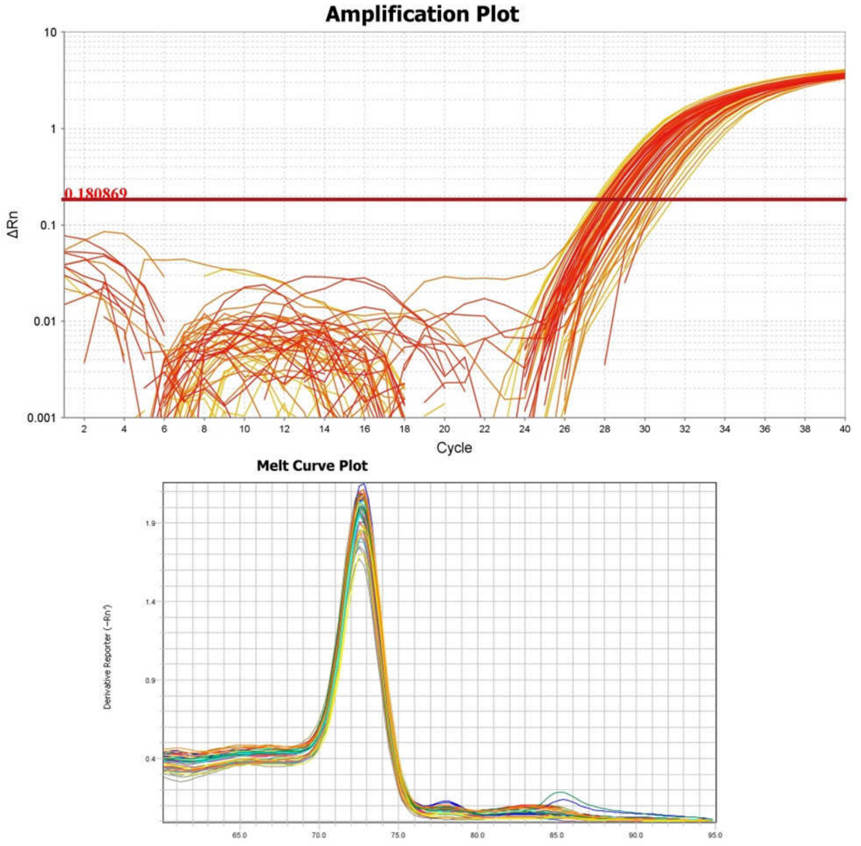

Real-Time PCR for the junction region of microRNA1126_circular RNA

(ID: Ch2:467982888-467983386)

By divergent primers ◀▶ &
with no template

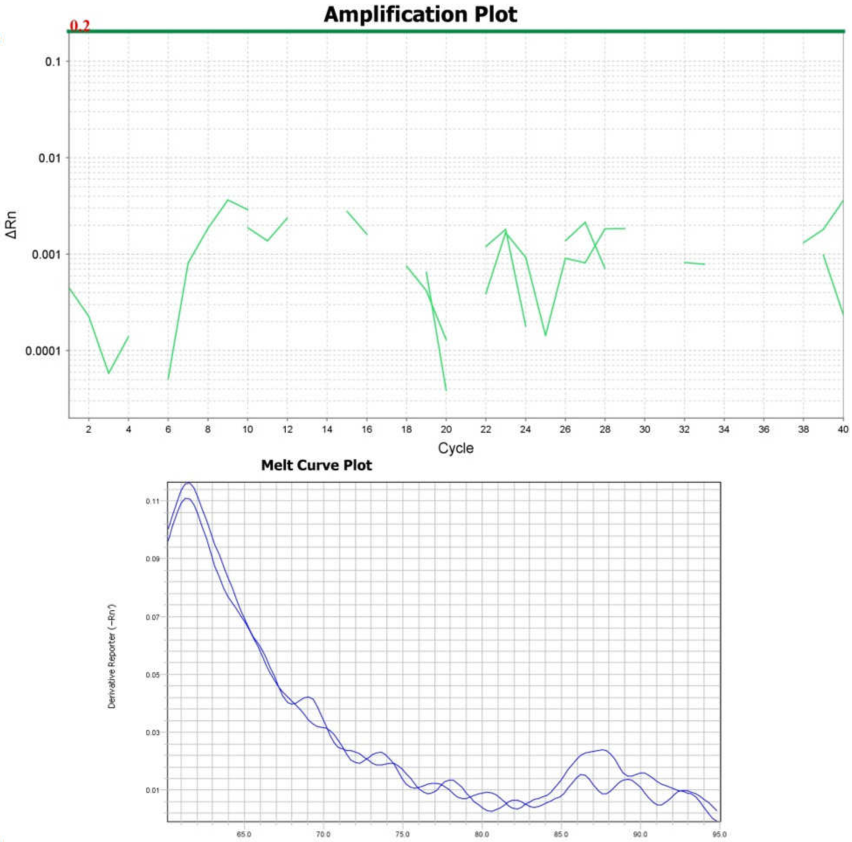

Real-Time PCR for the microRNA1126

By convergent primers ▶◀ on cDNAs

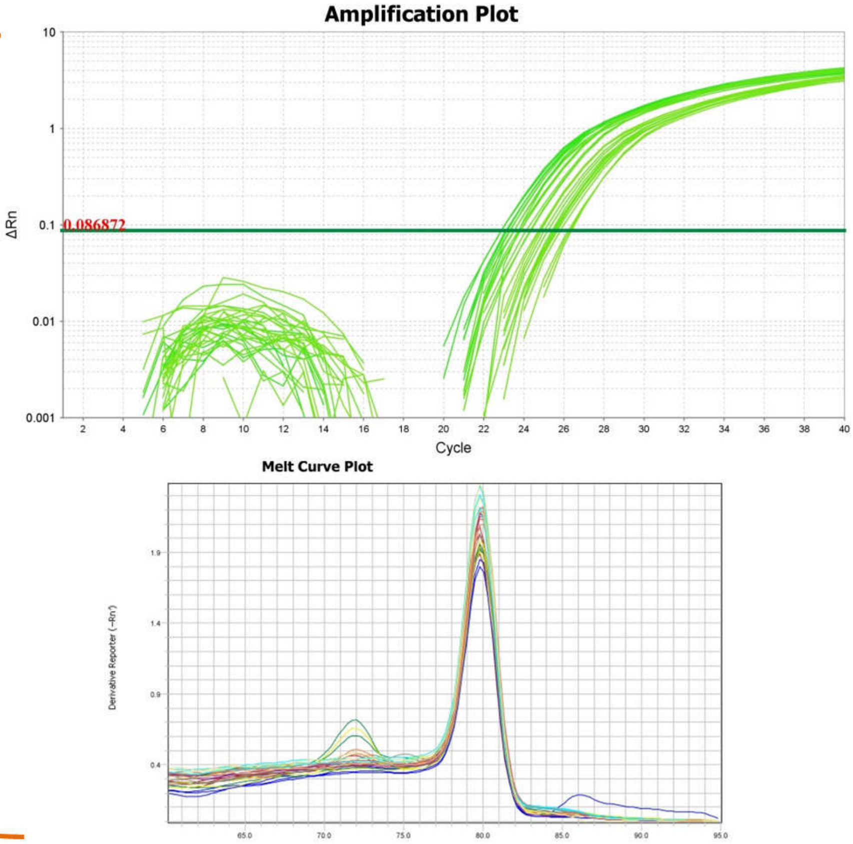

By convergent primers ▶◀ & with no template

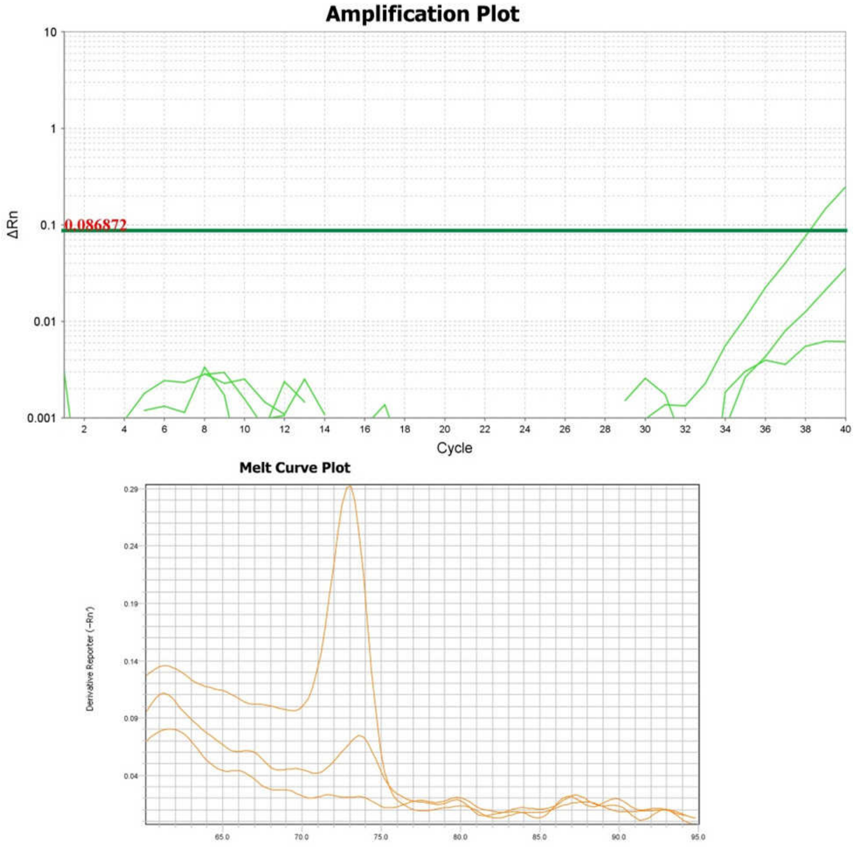

Aux1_circular RNA (ID: Ch3:486789367-486789848**)**

GGAGATCATGCACGCGATGTGGAAGCCGGCCAAGTTCAAGTACATCTACCTGCTGGCGACGC
TGTACGTGTTCACGCTGACGCTGCCGTCGGCGTCGGCCATGTACTGGGCGTACGGGGACGAG
CTGCTGGCGCACGCCAACGCCTTCTCGCTGCTGCCCAAGACGGCGTGGCGGGACGCCGCCGT
GATCCTCATGCTCATCCACCAGTTCATCACCTTCGGCTTCGCCTGCACGCCGCTCTACTTCGTG
TGGGAGAAGGTGATCGGCATGCACGACACCAAGAGCGTCTGCCTCCGCGCCCTCGCCAGGCT
CCCCATCGTCGTCCCCATCTGGTTCCTCGCCATCATCTTCCCCTTCTTCGGCCCCATCAACTCC
GCCGTCGGCGCCCTCCTCGTCAGCTTCACCGTCTACATCATCCCCGCCTCCCCGCCCTCGCCC
ACATC**CCCACATCCTCACCTACCGATCCGCATCCGCACGCGCC**

The nucleotides of junction-region are underlined. The nucleotides of junction-region which are supported by the junction-spaning sequencing reads are shown in red. Introns are not shown if the absence is supported by sequencing reads. In the absence of supporting sequencing reads, the intronic nucletides are shown as N.

Structural relationship between the circular RNA and its parental gene

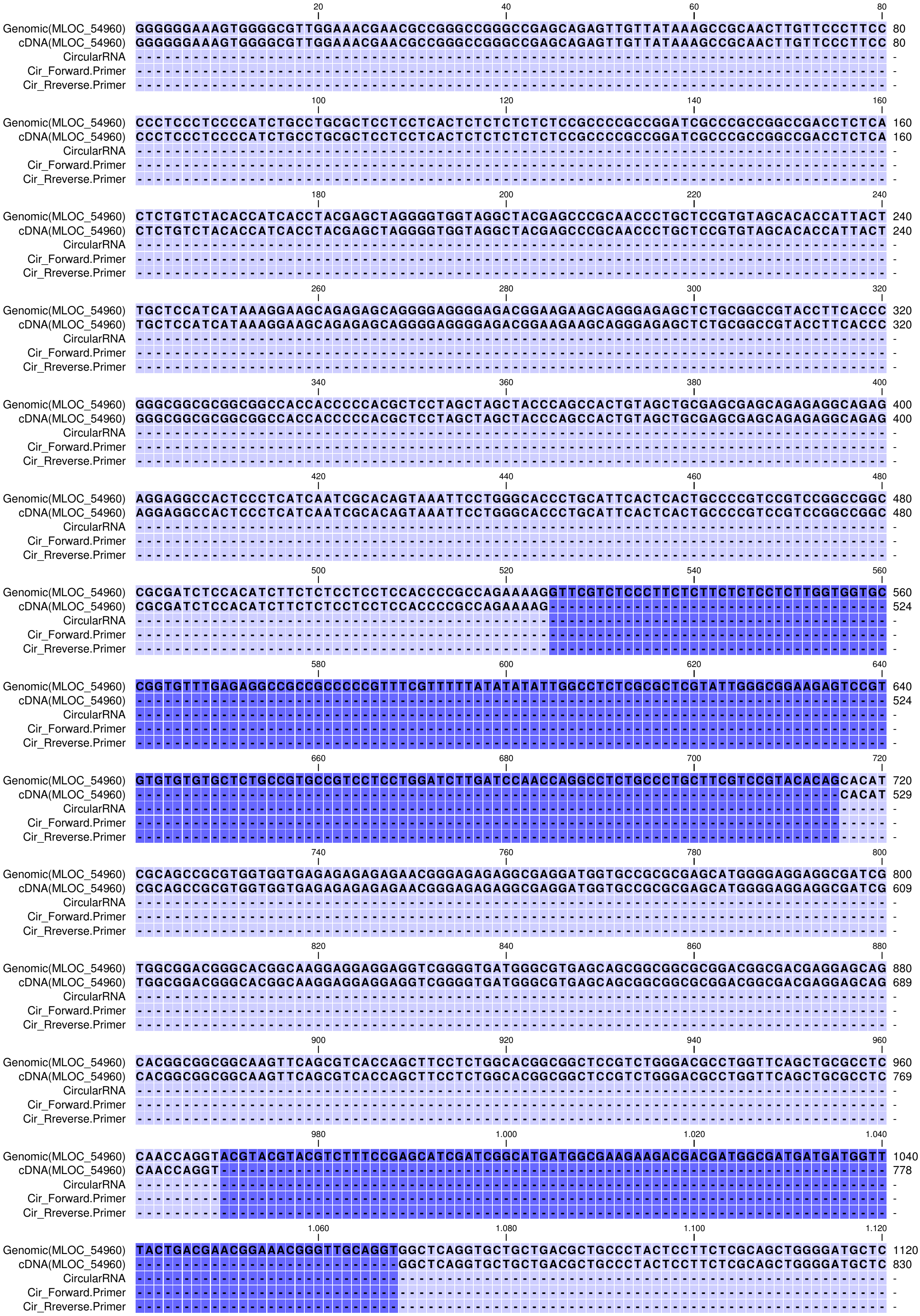
Genomic(MLOC_54960)
cDNA(MLOC_54960)
CircularRNA
Cir_Forward.Primer
Cir_Rreverse.Primer
20 40 60 80 100 120 140 160 180 200 220 240 260 280 300 320 340 360 380 400 420 440 460 480 500 520 540 560 580 600 620 640 660 680 700 720 740 760 780 800 820 840 860 880 900 920 940 960 980 1.000 1.020 1.040 1.060 1.080 1.100 1.120

```
                      1.140               1.160               1.180               1.200
Genomic(MLOC_54960)   TCGGGCATCCTGCTGCAGCTCTTCTACGGCTTCCTGGGCAGCTGGACCGCCTACCTCATCAGCGTCCTCTACGTCGAGTA 1200
cDNA(MLOC_54960)      TCGGGCATCCTGCTGCAGCTCTTCTACGGCTTCCTGGGCAGCTGGACCGCCTACCTCATCAGCGTCCTCTACGTCGAGTA 910
CircularRNA           -------------------------------------------------------------------------------- -
Cir_Forward.Primer    -------------------------------------------------------------------------------- -
Cir_Rreverse.Primer   -------------------------------------------------------------------------------- -

                      1.220               1.240               1.260               1.280
Genomic(MLOC_54960)   CCGCTCCCGCAAGGAGAAGGAGGGCGTCAGCTTCAAGAACCACGTCATCCAGGTACGCACCCCTCTTCCTTCATCCCTAA 1280
cDNA(MLOC_54960)      CCGCTCCCGCAAGGAGAAGGAGGGCGTCAGCTTCAAGAACCACGTCATCCAG---------------------------- 962
CircularRNA           -------------------------------------------------------------------------------- -
Cir_Forward.Primer    -------------------------------------------------------------------------------- -
Cir_Rreverse.Primer   -------------------------------------------------------------------------------- -

                      1.300               1.320               1.340               1.360
Genomic(MLOC_54960)   TAACGGATTGCAGTTTCCGGCTTCACCAAGCGGCCACGCCTTTCACTTCTCTTCTCTTCTCTTCTCTTCTCTTCTGTCGG 1360
cDNA(MLOC_54960)      -------------------------------------------------------------------------------- 962
CircularRNA           -------------------------------------------------------------------------------- -
Cir_Forward.Primer    -------------------------------------------------------------------------------- -
Cir_Rreverse.Primer   -------------------------------------------------------------------------------- -

                      1.380               1.400               1.420               1.440
Genomic(MLOC_54960)   CCCTGGCTGCAAAAACACGAAAAAAATGCTCTCCTTTCCCTTTTCCCCGAATGAGAAAACAAAGCAGAAAACCTCCTCGG 1440
cDNA(MLOC_54960)      -------------------------------------------------------------------------------- 962
CircularRNA           -------------------------------------------------------------------------------- -
Cir_Forward.Primer    -------------------------------------------------------------------------------- -
Cir_Rreverse.Primer   -------------------------------------------------------------------------------- -

                      1.460               1.480               1.500               1.520
Genomic(MLOC_54960)   GTTGCTTGCGGAACGGAAAGTGTGAACCGCATCCTTAATCCTTCCCTTTTAGTCGCAAAACCGGAACCGCATTTGCTCCC 1520
cDNA(MLOC_54960)      -------------------------------------------------------------------------------- 962
CircularRNA           -------------------------------------------------------------------------------- -
Cir_Forward.Primer    -------------------------------------------------------------------------------- -
Cir_Rreverse.Primer   -------------------------------------------------------------------------------- -

                      1.540               1.560               1.580               1.600
Genomic(MLOC_54960)   AACTAAACTAATTATGCTTAATGAATGATGTATATACAGTACAGTACATGTACATGTACTAGTAGAAACATACGTACATG 1600
cDNA(MLOC_54960)      -------------------------------------------------------------------------------- 962
CircularRNA           -------------------------------------------------------------------------------- -
Cir_Forward.Primer    -------------------------------------------------------------------------------- -
Cir_Rreverse.Primer   -------------------------------------------------------------------------------- -

                      1.620               1.640               1.660               1.680
Genomic(MLOC_54960)   ATGGATGGAGTGAGTGAGTGAGTAGCAAGTAGCAACTGTGGGCGGTTGATTAATGGCCAAGAAAGAGAGAGAGAGATAGA 1680
cDNA(MLOC_54960)      -------------------------------------------------------------------------------- 962
CircularRNA           -------------------------------------------------------------------------------- -
Cir_Forward.Primer    -------------------------------------------------------------------------------- -
Cir_Rreverse.Primer   -------------------------------------------------------------------------------- -

                      1.700               1.720               1.740               1.760
Genomic(MLOC_54960)   TACTAGGAAGAAGAATCACTACTACCGGACTGGAGTAGTAGTACTAACGAAATGAAGCTGCGGTGTTGATTGCGCAGTGG 1760
cDNA(MLOC_54960)      -----------------------------------------------------------------------------TGG 965
CircularRNA           -------------------------------------------------------------------------------- -
Cir_Forward.Primer    -------------------------------------------------------------------------------- -
Cir_Rreverse.Primer   -------------------------------------------------------------------------------- -

                      1.780               1.800               1.820               1.840
Genomic(MLOC_54960)   TTCGAGGTGCTCGACGGGCTGCTGGGCCCGTACTGGAAGGCGGCCGGGCTGGCCTTCAACTGCACGTTCCTCCTCTTCGG 1840
cDNA(MLOC_54960)      TTCGAGGTGCTCGACGGGCTGCTGGGCCCGTACTGGAAGGCGGCCGGGCTGGCCTTCAACTGCACGTTCCTCCTCTTCGG 1045
CircularRNA           -------------------------------------------------------------------------------- -
Cir_Forward.Primer    -------------------------------------------------------------------------------- -
Cir_Rreverse.Primer   -------------------------------------------------------------------------------- -

                      1.860               1.880               1.900               1.920
Genomic(MLOC_54960)   CACCGTCATCCAGCTGATCGCCTGCGCCAGGTCAGTCAGCCGCTCCATCCATCCATCCATCCATCACCCTCATCGTCCTA 1920
cDNA(MLOC_54960)      CACCGTCATCCAGCTGATCGCCTGCGCCAG-------------------------------------------------- 1075
CircularRNA           -------------------------------------------------------------------------------- -
Cir_Forward.Primer    -------------------------------------------------------------------------------- -
Cir_Rreverse.Primer   -------------------------------------------------------------------------------- -

                      1.940               1.960               1.980               2.000
Genomic(MLOC_54960)   CTACTCACTGTGCCAGCACCACCAGTCCAGCCGCTGAGATGAGATGAAAAATGAATTTATTTTTACATGTACATGCAGCA 2000
cDNA(MLOC_54960)      ------------------------------------------------------------------------------CA 1077
CircularRNA           -------------------------------------------------------------------------------- -
Cir_Forward.Primer    -------------------------------------------------------------------------------- -
Cir_Rreverse.Primer   -------------------------------------------------------------------------------- -

                      2.020               2.040               2.060               2.080
Genomic(MLOC_54960)   ACATCTACTACATCAACGACCGGCTGGACAAGCGGACGTGGACATACATCTTCGGCGCGTGCTGCGCGACGACGGTGTTC 2080
cDNA(MLOC_54960)      ACATCTACTACATCAACGACCGGCTGGACAAGCGGACGTGGACATACATCTTCGGCGCGTGCTGCGCGACGACGGTGTTC 1157
CircularRNA           -------------------------------------------------------------------------------- -
Cir_Forward.Primer    -------------------------------------------------------------------------------- -
Cir_Rreverse.Primer   -------------------------------------------------------------------------------- -

                      2.100               2.120               2.140               2.160
Genomic(MLOC_54960)   ATCCCGTCGTTCCACAACTACCGGATCTGGTCCTTCCTGGGGCTGGGCATGACCACCTACACCGCCTGGTACCTCGCCAT 2160
cDNA(MLOC_54960)      ATCCCGTCGTTCCACAACTACCGGATCTGGTCCTTCCTGGGGCTGGGCATGACCACCTACACCGCCTGGTACCTCGCCAT 1237
CircularRNA           -------------------------------------------------------------------------------- -
Cir_Forward.Primer    -------------------------------------------------------------------------------- -
Cir_Rreverse.Primer   -------------------------------------------------------------------------------- -

                      2.180               2.200               2.220               2.240
Genomic(MLOC_54960)   CGCCGCGCTCATCAACGGCCAGGTCGAGGGCGTCACCCACACCGGACCAAACAAGCTCGTCCTCTACTTCACCGGCGCCA 2240
cDNA(MLOC_54960)      CGCCGCGCTCATCAACGGCCAGGTCGAGGGCGTCACCCACACCGGACCAAACAAGCTCGTCCTCTACTTCACCGGCGCCA 1317
CircularRNA           -------------------------------------------------------------------------------- -
Cir_Forward.Primer    -------------------------------------------------------------------------------- -
Cir_Rreverse.Primer   -------------------------------------------------------------------------------- -
```

```
                                    2.260               2.280               2.300               2.320
Genomic(MLOC_54960)  CCAACATCCTCTACACCTTCGGCGGCCACGCCGTCACAGTGTAAGCACCCACACACTATTCTCTTACTGTTTACCTCCAC  2320
cDNA(MLOC_54960)     CCAACATCCTCTACACCTTCGGCGGCCACGCCGTCACAGT----------------------------------------  1357
CircularRNA          --------------------------------------------------------------------------------  -
Cir_Forward.Primer   --------------------------------------------------------------------------------  -
Cir_Rreverse.Primer  --------------------------------------------------------------------------------  -

                                    2.340               2.360               2.380               2.400
Genomic(MLOC_54960)  TTATCTCTTCAGCAAGTAAATTACTACTCTAGTAAGACACGTCTGGATGAAGAAGGGTGGTGAACTTTCCGCCGGGCTGC  2400
cDNA(MLOC_54960)     --------------------------------------------------------------------------------  1357
CircularRNA          --------------------------------------------------------------------------------  -
Cir_Forward.Primer   --------------------------------------------------------------------------------  -
Cir_Rreverse.Primer  --------------------------------------------------------------------------------  -

                                    2.420               2.440               2.460               2.480
Genomic(MLOC_54960)  AGCTTTTGCCGGCCGATCGGGCCGGAGGACATGTCTGGATTGGAAACCCGGTTCTAGTTTCCATTCCAATGGTCCCCCGG  2480
cDNA(MLOC_54960)     --------------------------------------------------------------------------------  1357
CircularRNA          --------------------------------------------------------------------------------  -
Cir_Forward.Primer   --------------------------------------------------------------------------------  -
Cir_Rreverse.Primer  --------------------------------------------------------------------------------  -

                                    2.500               2.520               2.540               2.560
Genomic(MLOC_54960)  CCATGGCCCTCTGCCCAGACCGGTCCATGGTACTTGCGGCAGCAGCAGCAGTACCACACTAACAGTAGCACTGTATGCAG  2560
cDNA(MLOC_54960)     --------------------------------------------------------------------------------  1357
CircularRNA          --------------------------------------------------------------------------------  -
Cir_Forward.Primer   --------------------------------------------------------------------------------  -
Cir_Rreverse.Primer  --------------------------------------------------------------------------------  -

                                    2.580               2.600               2.620               2.640
Genomic(MLOC_54960)  TACCAGTACGAGACTATTAGTTAATCTTGATTAGTAGTAGTAACTAAACAAAGATTGTAGCAGCATGTAGTACGTAGCTT  2640
cDNA(MLOC_54960)     --------------------------------------------------------------------------------  1357
CircularRNA          --------------------------------------------------------------------------------  -
Cir_Forward.Primer   --------------------------------------------------------------------------------  -
Cir_Rreverse.Primer  --------------------------------------------------------------------------------  -

                                    2.660               2.680               2.700               2.720
Genomic(MLOC_54960)  CCGCTTTACATGTAGCTAGGCTAGCCCCATCCAACCCATGGGGCACAGATCCCAGCGCACAGTTAGGGGCACTTATAAAA  2720
cDNA(MLOC_54960)     --------------------------------------------------------------------------------  1357
CircularRNA          --------------------------------------------------------------------------------  -
Cir_Forward.Primer   --------------------------------------------------------------------------------  -
Cir_Rreverse.Primer  --------------------------------------------------------------------------------  -

                                    2.740               2.760               2.780               2.800
Genomic(MLOC_54960)  ACTTACACATAGCCCCCTTCTGGTTTAGCTTAGCTTAGCTTAGGTTAGCTAGCTTGCGGCCATTAGTCAGTCCAGTCCGA  2800
cDNA(MLOC_54960)     --------------------------------------------------------------------------------  1357
CircularRNA          --------------------------------------------------------------------------------  -
Cir_Forward.Primer   --------------------------------------------------------------------------------  -
Cir_Rreverse.Primer  --------------------------------------------------------------------------------  -

                                    2.820               2.840               2.860               2.880
Genomic(MLOC_54960)  TGGAAGCTCGGTGCGCTCATGTGCGGTGATGCATCCAAAAGGTCCTCCTCCCTACCAGTACTAGCACCCACACACACACA  2880
cDNA(MLOC_54960)     --------------------------------------------------------------------------------  1357
CircularRNA          --------------------------------------------------------------------------------  -
Cir_Forward.Primer   --------------------------------------------------------------------------------  -
Cir_Rreverse.Primer  --------------------------------------------------------------------------------  -

                                    2.900               2.920               2.940               2.960
Genomic(MLOC_54960)  CACACACACACACACATGCATGATACAGTCTAGTCTAGAGGAGAAAAGAATCGAAGGCGCGGTATATTTTACATCACCGG  2960
cDNA(MLOC_54960)     --------------------------------------------------------------------------------  1357
CircularRNA          --------------------------------------------------------------------------------  -
Cir_Forward.Primer   --------------------------------------------------------------------------------  -
Cir_Rreverse.Primer  --------------------------------------------------------------------------------  -

                                    2.980               3.000               3.020               3.040
Genomic(MLOC_54960)  CCACCATGCATGCATTAGACTAGAGCCCTTTATTCCCATTGCTCATCTGCCGCGATTAGTTATTAACTAGTAATCCAGTG  3040
cDNA(MLOC_54960)     --------------------------------------------------------------------------------  1357
CircularRNA          --------------------------------------------------------------------------------  -
Cir_Forward.Primer   --------------------------------------------------------------------------------  -
Cir_Rreverse.Primer  --------------------------------------------------------------------------------  -

                                    3.060               3.080               3.100               3.120
Genomic(MLOC_54960)  CTCGGGTCACCGGTGTCTCCTGCTAGCCAGCTAGCTTAGCCATGATTTCAGGCCATGCGGATGCGGGGGGCGAATAATTA  3120
cDNA(MLOC_54960)     --------------------------------------------------------------------------------  1357
CircularRNA          --------------------------------------------------------------------------------  -
Cir_Forward.Primer   --------------------------------------------------------------------------------  -
Cir_Rreverse.Primer  --------------------------------------------------------------------------------  -

                                    3.140               3.160               3.180               3.200
Genomic(MLOC_54960)  GCTTCGTCCCCTTCAGTGAGTGAGTTAGTGCGGTGTTCAAACACGCTGGTCGCTCGCTAGTGGCCGCCGTTGGGCCGGAG  3200
cDNA(MLOC_54960)     --------------------------------------------------------------------------------  1357
CircularRNA          --------------------------------------------------------------------------------  -
Cir_Forward.Primer   --------------------------------------------------------------------------------  -
Cir_Rreverse.Primer  --------------------------------------------------------------------------------  -

                                    3.220               3.240               3.260               3.280
Genomic(MLOC_54960)  ATATTTGAACCATTTTTATACCTTGTAAAATAAGATCTCATATCGTAACCTCGGCGACCGGAGCACTACTACTGTTACTA  3280
cDNA(MLOC_54960)     --------------------------------------------------------------------------------  1357
CircularRNA          --------------------------------------------------------------------------------  -
Cir_Forward.Primer   --------------------------------------------------------------------------------  -
Cir_Rreverse.Primer  --------------------------------------------------------------------------------  -

                                    3.300               3.320               3.340               3.360
Genomic(MLOC_54960)  TCAGTACAGTACTCCATTATTAAGTAGCAGCACAGTGGTAGTTGCTAATGCATGCTGCAGCCAGTGGTGGCGTGAAACGA  3360
cDNA(MLOC_54960)     --------------------------------------------------------------------------------  1357
CircularRNA          --------------------------------------------------------------------------------  -
Cir_Forward.Primer   --------------------------------------------------------------------------------  -
Cir_Rreverse.Primer  --------------------------------------------------------------------------------  -
```

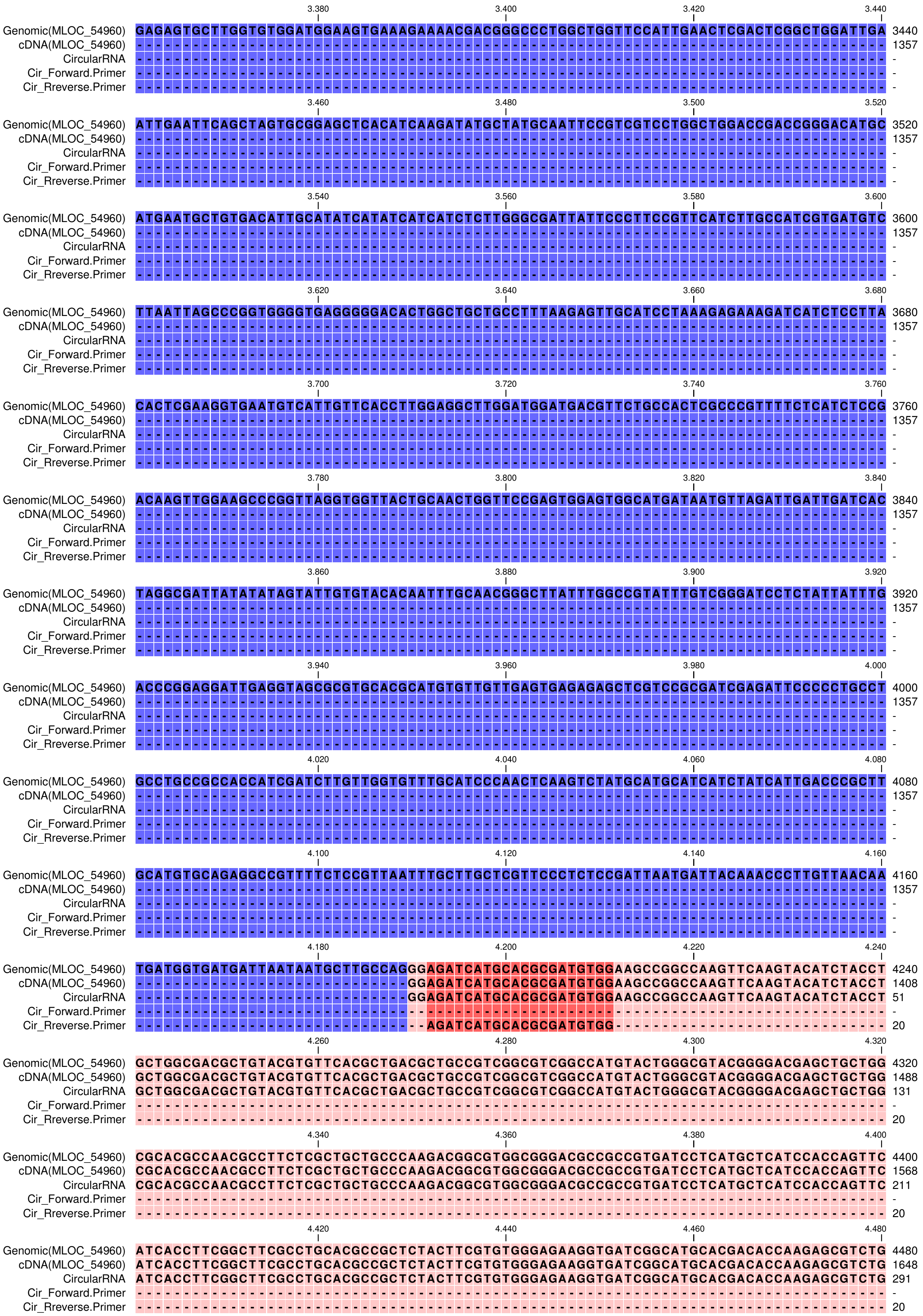

3.380 3.400 3.420 3.440
Genomic(MLOC_54960) GAGAGTGCTTGGTGTGGATGGAAGTGAAAGAAAACGACGGGCCCTGGCTGGTTCCATTGAACTCGACTCGGCTGGATTGA 3440
cDNA(MLOC_54960) -- 1357
CircularRNA -- -
Cir_Forward.Primer -- -
Cir_Rreverse.Primer -- -
3.460 3.480 3.500 3.520
Genomic(MLOC_54960) ATTGAATTCAGCTAGTGCGGAGCTCACATCAAGATATGCTATGCAATTCCGTCGTCCTGGCTGGACCGACCGGGACATGC 3520
cDNA(MLOC_54960) -- 1357
CircularRNA -- -
Cir_Forward.Primer -- -
Cir_Rreverse.Primer -- -
3.540 3.560 3.580 3.600
Genomic(MLOC_54960) ATGAATGCTGTGACATTGCATATCATATCATCATCTCTTGGGCGATTATTCCCTTCCGTTCATCTTGCCATCGTGATGTC 3600
cDNA(MLOC_54960) -- 1357
CircularRNA -- -
Cir_Forward.Primer -- -
Cir_Rreverse.Primer -- -
3.620 3.640 3.660 3.680
Genomic(MLOC_54960) TTAATTAGCCCGGTGGGGTGAGGGGGACACTGGCTGCTGCCTTTAAGAGTTGCATCCTAAAGAGAAAGATCATCTCCTTA 3680
cDNA(MLOC_54960) -- 1357
CircularRNA -- -
Cir_Forward.Primer -- -
Cir_Rreverse.Primer -- -
3.700 3.720 3.740 3.760
Genomic(MLOC_54960) CACTCGAAGGTGAATGTCATTGTTCACCTTGGAGGCTTGGATGGATGACGTTCTGCCACTCGCCCGTTTTCTCATCTCCG 3760
cDNA(MLOC_54960) -- 1357
CircularRNA -- -
Cir_Forward.Primer -- -
Cir_Rreverse.Primer -- -
3.780 3.800 3.820 3.840
Genomic(MLOC_54960) ACAAGTTGGAAGCCCGGTTAGGTGGTTACTGCAACTGGTTCCGAGTGGAGTGGCATGATAATGTTAGATTGATTGATCAC 3840
cDNA(MLOC_54960) -- 1357
CircularRNA -- -
Cir_Forward.Primer -- -
Cir_Rreverse.Primer -- -
3.860 3.880 3.900 3.920
Genomic(MLOC_54960) TAGGCGATTATATATAGTATTGTGTACACAATTTGCAACGGGCTTATTTGGCCGTATTTGTCGGGATCCTCTATTATTTG 3920
cDNA(MLOC_54960) -- 1357
CircularRNA -- -
Cir_Forward.Primer -- -
Cir_Rreverse.Primer -- -
3.940 3.960 3.980 4.000
Genomic(MLOC_54960) ACCCGGAGGATTGAGGTAGCGCGTGCACGCATGTGTTGTTGAGTGAGAGAGCTCGTCCGCGATCGAGATTCCCCCTGCCT 4000
cDNA(MLOC_54960) -- 1357
CircularRNA -- -
Cir_Forward.Primer -- -
Cir_Rreverse.Primer -- -
4.020 4.040 4.060 4.080
Genomic(MLOC_54960) GCCTGCCGCCACCATCGATCTTGTTGGTGTTTGCATCCCAACTCAAGTCTATGCATGCATCATCTATCATTGACCCGCTT 4080
cDNA(MLOC_54960) -- 1357
CircularRNA -- -
Cir_Forward.Primer -- -
Cir_Rreverse.Primer -- -
4.100 4.120 4.140 4.160
Genomic(MLOC_54960) GCATGTGCAGAGGCCGTTTTCTCCGTTAATTTGCTTGCTCGTTCCCTCTCCGATTAATGATTACAAACCCTTGTTAACAA 4160
cDNA(MLOC_54960) -- 1357
CircularRNA -- -
Cir_Forward.Primer -- -
Cir_Rreverse.Primer -- -
4.180 4.200 4.220 4.240
Genomic(MLOC_54960) TGATGGTGATGATTAATAATGCTTGCCAGGGAGATCATGCACGCGATGTGGAAGCCGGCCAAGTTCAAGTACATCTACCT 4240
cDNA(MLOC_54960) -----------------------------GGAGATCATGCACGCGATGTGGAAGCCGGCCAAGTTCAAGTACATCTACCT 1408
CircularRNA -----------------------------GGAGATCATGCACGCGATGTGGAAGCCGGCCAAGTTCAAGTACATCTACCT 51
Cir_Forward.Primer -- -
Cir_Rreverse.Primer -------------------------------AGATCATGCACGCGATGTGG----------------------------- 20
4.260 4.280 4.300 4.320
Genomic(MLOC_54960) GCTGGCGACGCTGTACGTGTTCACGCTGACGCTGCCGTCGGCGTCGGCCATGTACTGGGCGTACGGGGACGAGCTGCTGG 4320
cDNA(MLOC_54960) GCTGGCGACGCTGTACGTGTTCACGCTGACGCTGCCGTCGGCGTCGGCCATGTACTGGGCGTACGGGGACGAGCTGCTGG 1488
CircularRNA GCTGGCGACGCTGTACGTGTTCACGCTGACGCTGCCGTCGGCGTCGGCCATGTACTGGGCGTACGGGGACGAGCTGCTGG 131
Cir_Forward.Primer -- -
Cir_Rreverse.Primer -- 20
4.340 4.360 4.380 4.400
Genomic(MLOC_54960) CGCACGCCAACGCCTTCTCGCTGCTGCCCAAGACGGCGTGGCGGGACGCCGCCGTGATCCTCATGCTCATCCACCAGTTC 4400
cDNA(MLOC_54960) CGCACGCCAACGCCTTCTCGCTGCTGCCCAAGACGGCGTGGCGGGACGCCGCCGTGATCCTCATGCTCATCCACCAGTTC 1568
CircularRNA CGCACGCCAACGCCTTCTCGCTGCTGCCCAAGACGGCGTGGCGGGACGCCGCCGTGATCCTCATGCTCATCCACCAGTTC 211
Cir_Forward.Primer -- -
Cir_Rreverse.Primer -- 20
4.420 4.440 4.460 4.480
Genomic(MLOC_54960) ATCACCTTCGGCTTCGCCTGCACGCCGCTCTACTTCGTGTGGGAGAAGGTGATCGGCATGCACGACACCAAGAGCGTCTG 4480
cDNA(MLOC_54960) ATCACCTTCGGCTTCGCCTGCACGCCGCTCTACTTCGTGTGGGAGAAGGTGATCGGCATGCACGACACCAAGAGCGTCTG 1648
CircularRNA ATCACCTTCGGCTTCGCCTGCACGCCGCTCTACTTCGTGTGGGAGAAGGTGATCGGCATGCACGACACCAAGAGCGTCTG 291
Cir_Forward.Primer -- -
Cir_Rreverse.Primer -- 20

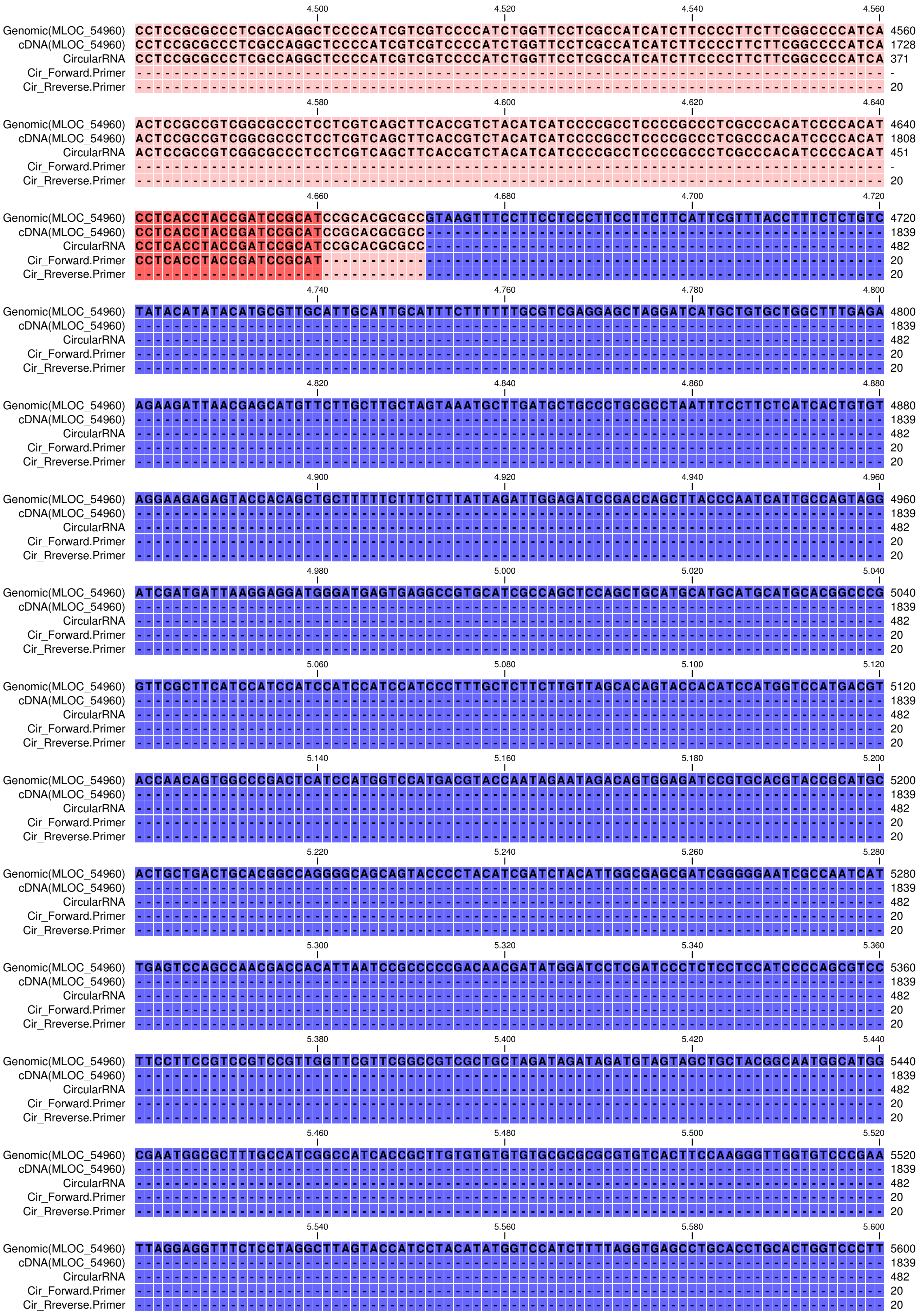

4.500 4.520 4.540 4.560
Genomic(MLOC_54960) CCTCCGCGCCCTCGCCAGGCTCCCCATCGTCGTCCCCATCTGGTTCCTCGCCATCATCTTCCCCTTCTTCGGCCCCATCA 4560
cDNA(MLOC_54960) CCTCCGCGCCCTCGCCAGGCTCCCCATCGTCGTCCCCATCTGGTTCCTCGCCATCATCTTCCCCTTCTTCGGCCCCATCA 1728
CircularRNA CCTCCGCGCCCTCGCCAGGCTCCCCATCGTCGTCCCCATCTGGTTCCTCGCCATCATCTTCCCCTTCTTCGGCCCCATCA 371
Cir_Forward.Primer -
Cir_Rreverse.Primer 20
4.580 4.600 4.620 4.640
Genomic(MLOC_54960) ACTCCGCCGTCGGCGCCCTCCTCGTCAGCTTCACCGTCTACATCATCCCCGCCTCCCCGCCCTCGCCCACATCCCCACAT 4640
cDNA(MLOC_54960) ACTCCGCCGTCGGCGCCCTCCTCGTCAGCTTCACCGTCTACATCATCCCCGCCTCCCCGCCCTCGCCCACATCCCCACAT 1808
CircularRNA ACTCCGCCGTCGGCGCCCTCCTCGTCAGCTTCACCGTCTACATCATCCCCGCCTCCCCGCCCTCGCCCACATCCCCACAT 451
Cir_Forward.Primer -
Cir_Rreverse.Primer 20
4.660 4.680 4.700 4.720
Genomic(MLOC_54960) CCTCACCTACCGATCCGCATCCGCACGCGCCGTAAGTTTCCTTCCTCCCTTCCTTCTTCATTCGTTTACCTTTCTCTGTC 4720
cDNA(MLOC_54960) CCTCACCTACCGATCCGCATCCGCACGCGCC 1839
CircularRNA CCTCACCTACCGATCCGCATCCGCACGCGCC 482
Cir_Forward.Primer CCTCACCTACCGATCCGCAT 20
Cir_Rreverse.Primer 20
4.740 4.760 4.780 4.800
Genomic(MLOC_54960) TATACATATACATGCGTTGCATTGCATTGCATTTCTTTTTTGCGTCGAGGAGCTAGGATCATGCTGTGCTGGCTTTGAGA 4800
cDNA(MLOC_54960) 1839
CircularRNA 482
Cir_Forward.Primer 20
Cir_Rreverse.Primer 20
4.820 4.840 4.860 4.880
Genomic(MLOC_54960) AGAAGATTAACGAGCATGTTCTTGCTTGCTAGTAAATGCTTGATGCTGCCCTGCGCCTAATTTCCTTCTCATCACTGTGT 4880
cDNA(MLOC_54960) 1839
CircularRNA 482
Cir_Forward.Primer 20
Cir_Rreverse.Primer 20
4.900 4.920 4.940 4.960
Genomic(MLOC_54960) AGGAAGAGAGTACCACAGCTGCTTTTTCTTTCTTTATTAGATTGGAGATCCGACCAGCTTACCCAATCATTGCCAGTAGG 4960
cDNA(MLOC_54960) 1839
CircularRNA 482
Cir_Forward.Primer 20
Cir_Rreverse.Primer 20
4.980 5.000 5.020 5.040
Genomic(MLOC_54960) ATCGATGATTAAGGAGGATGGGATGAGTGAGGCCGTGCATCGCCAGCTCCAGCTGCATGCATGCATGCATGCACGGCCCG 5040
cDNA(MLOC_54960) 1839
CircularRNA 482
Cir_Forward.Primer 20
Cir_Rreverse.Primer 20
5.060 5.080 5.100 5.120
Genomic(MLOC_54960) GTTCGCTTCATCCATCCATCCATCCATCCATCCCTTTGCTCTTCTTGTTAGCACAGTACCACATCCATGGTCCATGACGT 5120
cDNA(MLOC_54960) 1839
CircularRNA 482
Cir_Forward.Primer 20
Cir_Rreverse.Primer 20
5.140 5.160 5.180 5.200
Genomic(MLOC_54960) ACCAACAGTGGCCCGACTCATCCATGGTCCATGACGTACCAATAGAATAGACAGTGGAGATCCGTGCACGTACCGCATGC 5200
cDNA(MLOC_54960) 1839
CircularRNA 482
Cir_Forward.Primer 20
Cir_Rreverse.Primer 20
5.220 5.240 5.260 5.280
Genomic(MLOC_54960) ACTGCTGACTGCACGGCCAGGGGCAGCAGTACCCCTACATCGATCTACATTGGCGAGCGATCGGGGGAATCGCCAATCAT 5280
cDNA(MLOC_54960) 1839
CircularRNA 482
Cir_Forward.Primer 20
Cir_Rreverse.Primer 20
5.300 5.320 5.340 5.360
Genomic(MLOC_54960) TGAGTCCAGCCAACGACCACATTAATCCGCCCCCGACAACGATATGGATCCTCGATCCCTCTCCTCCATCCCCAGCGTCC 5360
cDNA(MLOC_54960) 1839
CircularRNA 482
Cir_Forward.Primer 20
Cir_Rreverse.Primer 20
5.380 5.400 5.420 5.440
Genomic(MLOC_54960) TTCCTTCCGTCCGTCCGTTGGTTCGTTCGGCCGTCGCTGCTAGATAGATAGATGTAGTAGCTGCTACGGCAATGGCATGG 5440
cDNA(MLOC_54960) 1839
CircularRNA 482
Cir_Forward.Primer 20
Cir_Rreverse.Primer 20
5.460 5.480 5.500 5.520
Genomic(MLOC_54960) CGAATGGCGCTTTGCCATCGGCCATCACCGCTTGTGTGTGTGTGCGCGCGCGTGTCACTTCCAAGGGTTGGTGTCCCGAA 5520
cDNA(MLOC_54960) 1839
CircularRNA 482
Cir_Forward.Primer 20
Cir_Rreverse.Primer 20
5.540 5.560 5.580 5.600
Genomic(MLOC_54960) TTAGGAGGTTTCTCCTAGGCTTAGTACCATCCTACATATGGTCCATCTTTTAGGTGAGCCTGCACCTGCACTGGTCCCTT 5600
cDNA(MLOC_54960) 1839
CircularRNA 482
Cir_Forward.Primer 20
Cir_Rreverse.Primer 20

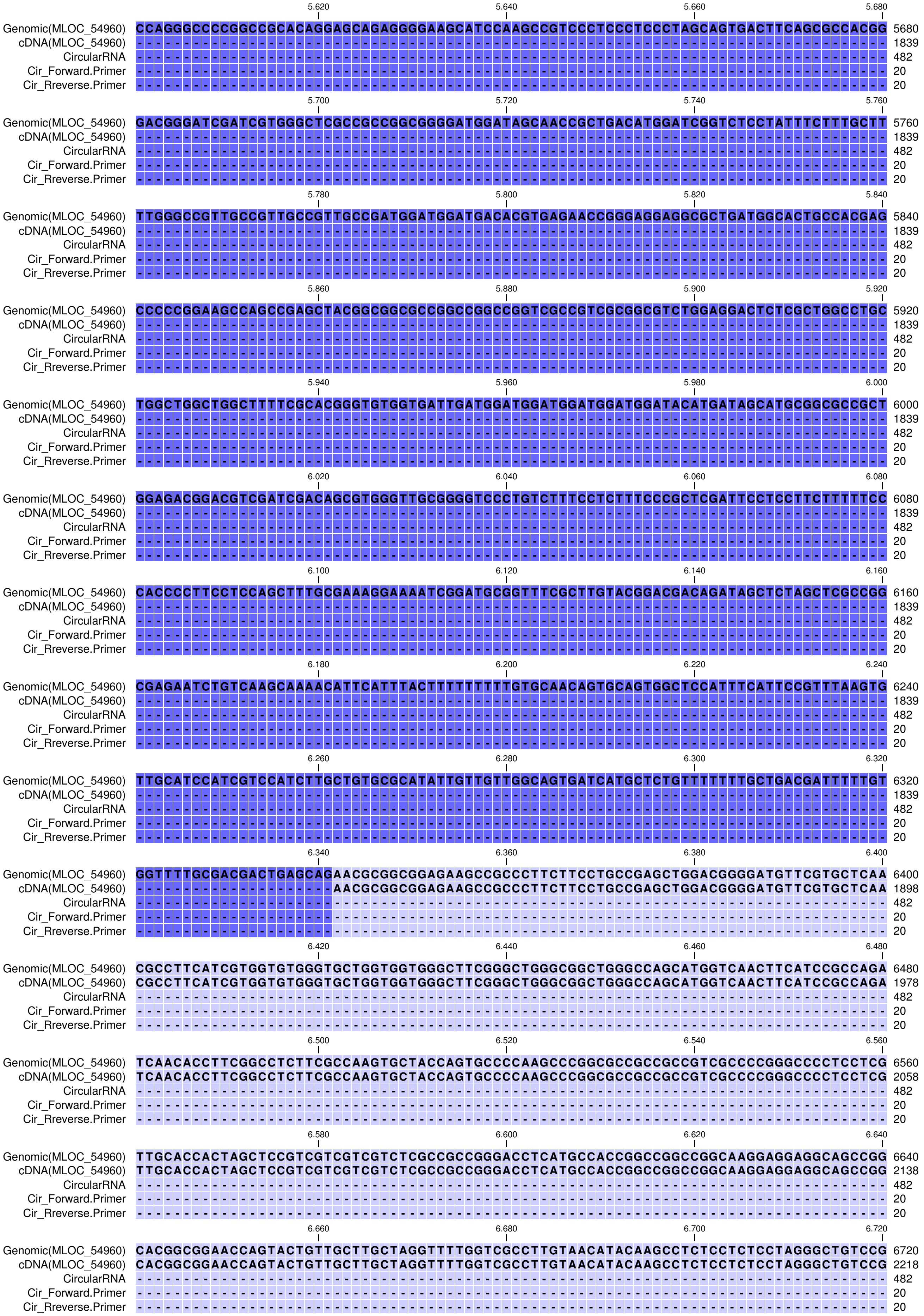

Genomic(MLOC_54960)
cDNA(MLOC_54960)
CircularRNA
Cir_Forward.Primer
Cir_Rreverse.Primer
5.620 5.640 5.660 5.680
5680 1839 482 20 20
5.700 5.720 5.740 5.760
5760 1839 482 20 20
5.780 5.800 5.820 5.840
5840 1839 482 20 20
5.860 5.880 5.900 5.920
5920 1839 482 20 20
5.940 5.960 5.980 6.000
6000 1839 482 20 20
6.020 6.040 6.060 6.080
6080 1839 482 20 20
6.100 6.120 6.140 6.160
6160 1839 482 20 20
6.180 6.200 6.220 6.240
6240 1839 482 20 20
6.260 6.280 6.300 6.320
6320 1839 482 20 20
6.340 6.360 6.380 6.400
6400 1898 482 20 20
6.420 6.440 6.460 6.480
6480 1978 482 20 20
6.500 6.520 6.540 6.560
6560 2058 482 20 20
6.580 6.600 6.620 6.640
6640 2138 482 20 20
6.660 6.680 6.700 6.720
6720 2218 482 20 20

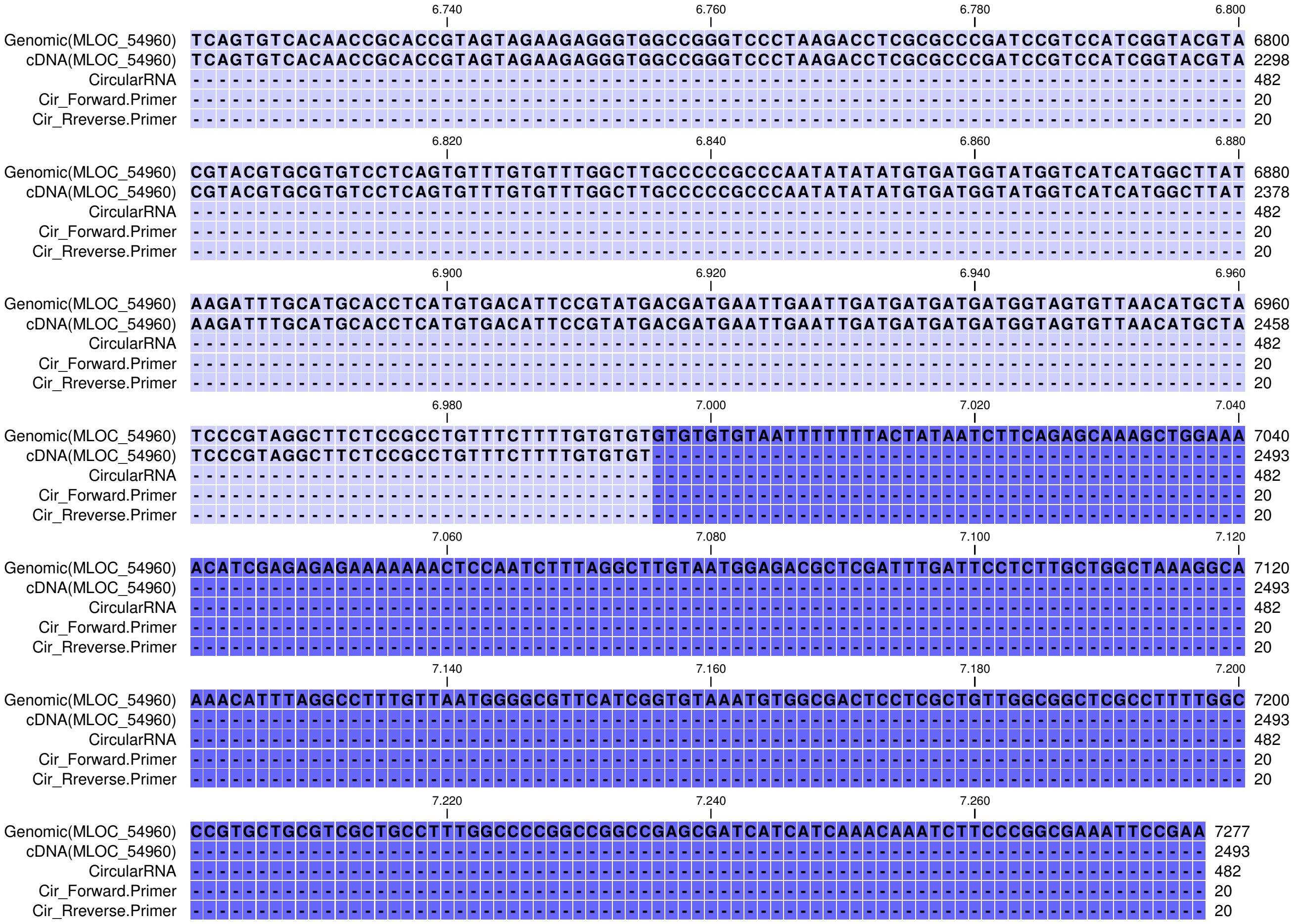
6.740 6.760 6.780 6.800
Genomic(MLOC_54960) TCAGTGTCACAACCGCACCGTAGTAGAAGAGGGTGGCCGGGTCCCTAAGACCTCGCGCCCGATCCGTCCATCGGTACGTA 6800
cDNA(MLOC_54960) TCAGTGTCACAACCGCACCGTAGTAGAAGAGGGTGGCCGGGTCCCTAAGACCTCGCGCCCGATCCGTCCATCGGTACGTA 2298
CircularRNA 482
Cir_Forward.Primer 20
Cir_Rreverse.Primer 20
6.820 6.840 6.860 6.880
Genomic(MLOC_54960) CGTACGTGCGTGTCCTCAGTGTTTGTGTTTGGCTTGCCCCCGCCCAATATATATGTGATGGTATGGTCATCATGGCTTAT 6880
cDNA(MLOC_54960) CGTACGTGCGTGTCCTCAGTGTTTGTGTTTGGCTTGCCCCCGCCCAATATATATGTGATGGTATGGTCATCATGGCTTAT 2378
CircularRNA 482
Cir_Forward.Primer 20
Cir_Rreverse.Primer 20
6.900 6.920 6.940 6.960
Genomic(MLOC_54960) AAGATTTGCATGCACCTCATGTGACATTCCGTATGACGATGAATTGAATTGATGATGATGATGGTAGTGTTAACATGCTA 6960
cDNA(MLOC_54960) AAGATTTGCATGCACCTCATGTGACATTCCGTATGACGATGAATTGAATTGATGATGATGATGGTAGTGTTAACATGCTA 2458
CircularRNA 482
Cir_Forward.Primer 20
Cir_Rreverse.Primer 20
6.980 7.000 7.020 7.040
Genomic(MLOC_54960) TCCCGTAGGCTTCTCCGCCTGTTTCTTTTGTGTGTGTGTGTGTAATTTTTTTACTATAATCTTCAGAGCAAAGCTGGAAA 7040
cDNA(MLOC_54960) TCCCGTAGGCTTCTCCGCCTGTTTCTTTTGTGTGT 2493
CircularRNA 482
Cir_Forward.Primer 20
Cir_Rreverse.Primer 20
7.060 7.080 7.100 7.120
Genomic(MLOC_54960) ACATCGAGAGAGAAAAAAACTCCAATCTTTAGGCTTGTAATGGAGACGCTCGATTTGATTCCTCTTGCTGGCTAAAGGCA 7120
cDNA(MLOC_54960) 2493
CircularRNA 482
Cir_Forward.Primer 20
Cir_Rreverse.Primer 20
7.140 7.160 7.180 7.200
Genomic(MLOC_54960) AAACATTTAGGCCTTTGTTAATGGGGCGTTCATCGGTGTAAATGTGGCGACTCCTCGCTGTTGGCGGCTCGCCTTTTGGC 7200
cDNA(MLOC_54960) 2493
CircularRNA 482
Cir_Forward.Primer 20
Cir_Rreverse.Primer 20
7.220 7.240 7.260
Genomic(MLOC_54960) CCGTGCTGCGTCGCTGCCTTTGGCCCCGGCCGGCCGAGCGATCATCATCAAACAAATCTTCCCGGCGAAATTCCGAA 7277
cDNA(MLOC_54960) 2493
CircularRNA 482
Cir_Forward.Primer 20
Cir_Rreverse.Primer 20

Real-Time PCR for the junction region of Aux1_circular RNA (ID: Ch3:486789367-486789848)

By divergent primers ◀▶ on genomic DNA

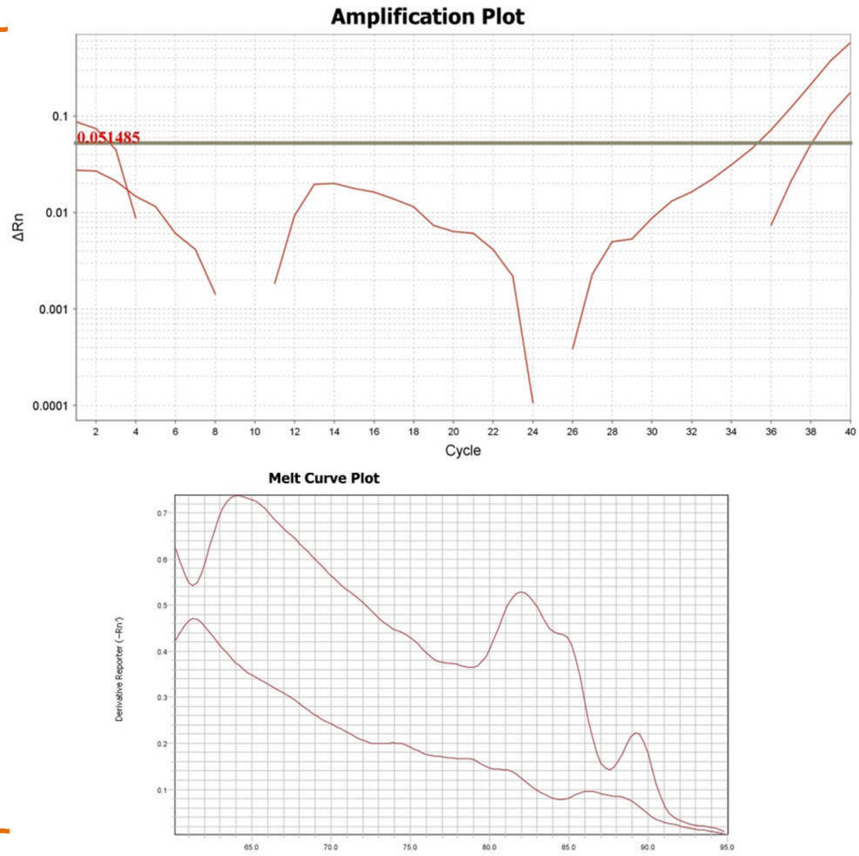

By divergent primers ◀▶ on cDNAs

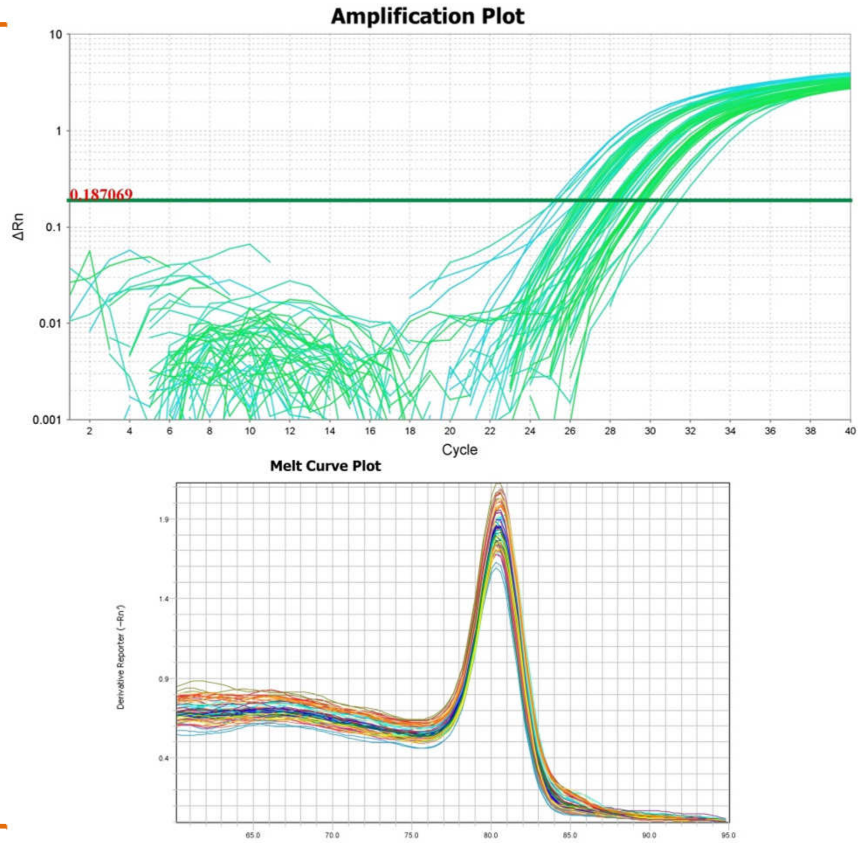

Real-Time PCR for the junction region of Aux1_circular RNA (ID: Ch3:486789367-486789848)

By divergent primers ◀▶ & with no template

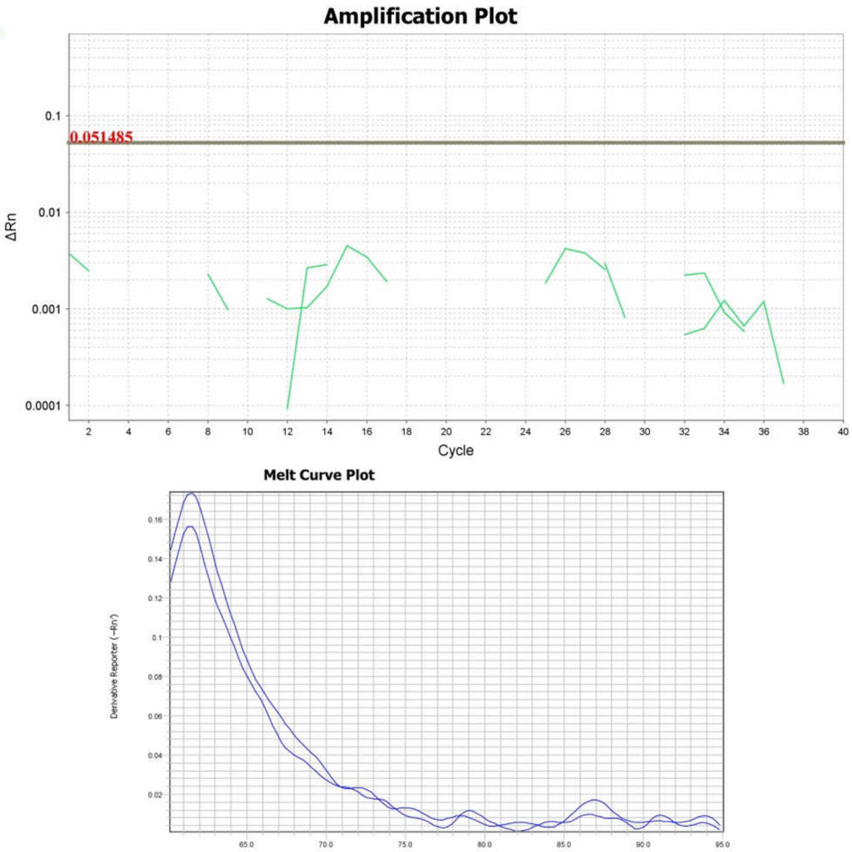

Real-Time PCR for Aux1

By convergent primers ▶◀ on cDNAs

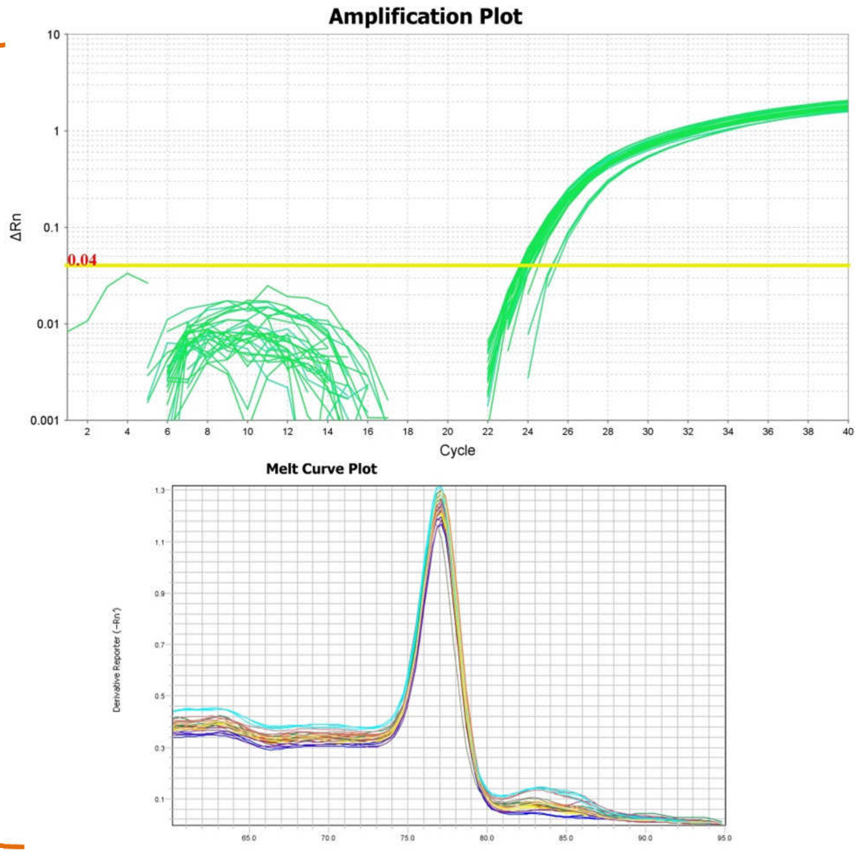

By convergent primers ▶◀ & with no template

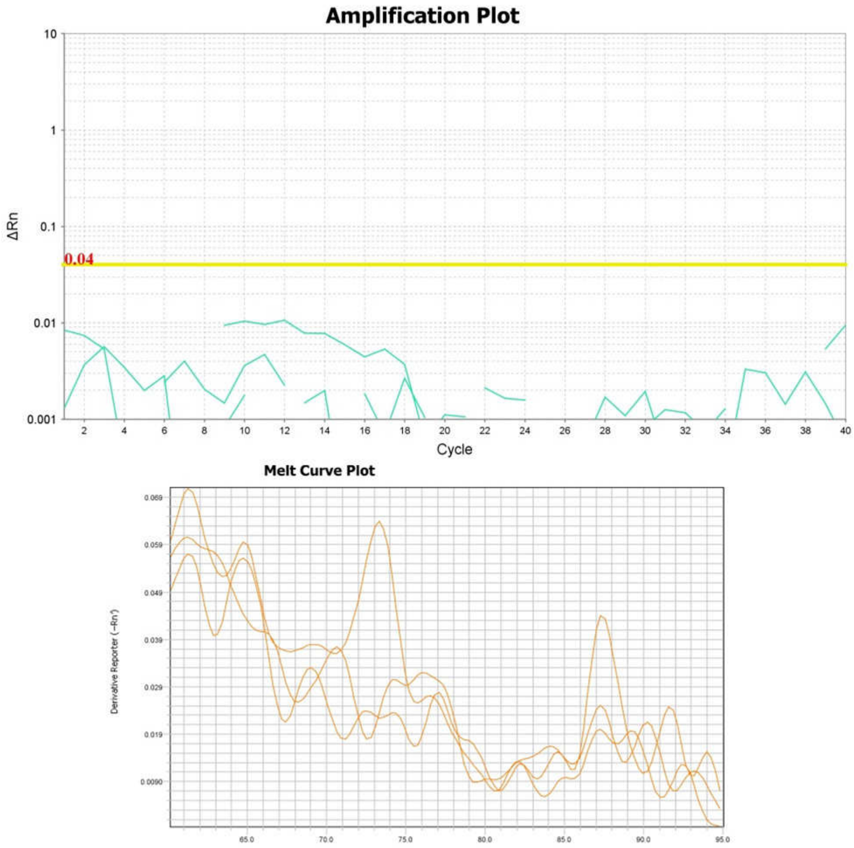

ATP-binding cassette Abcl3_circular RNA (ID: Morex_contig1661226:956-1173)

<u>G</u>TGGAAATTCTCGTATTTCGTATGTACATGTTCCTGCGGCTCGGATGAGTATAGTTATTTATATC
GCGACAGCTATAAACAGTTCCTTGTTCCCATTAACAAAACATCCCCTTTTTCTTCGCTCTTCCGG
AACCGGTACAGAAATTGGTGCTTTTTCTACTTTGTTTACGTTAGTGACTGGGG**GGTTTTGGGGAA**
GGCCTATGTGGGGTACCTTTCG<u>G</u>

The nucleotides of junction-region are underlined. The nucleotides of junction-region which are supported by the junction-spaning sequencing reads are shown in red. Introns are not shown if the absence is supported by sequencing reads. In the absence of supporting sequencing reads, the intronic nucletides are shown as N.

Structural relationship between the circular RNA and its parental gene

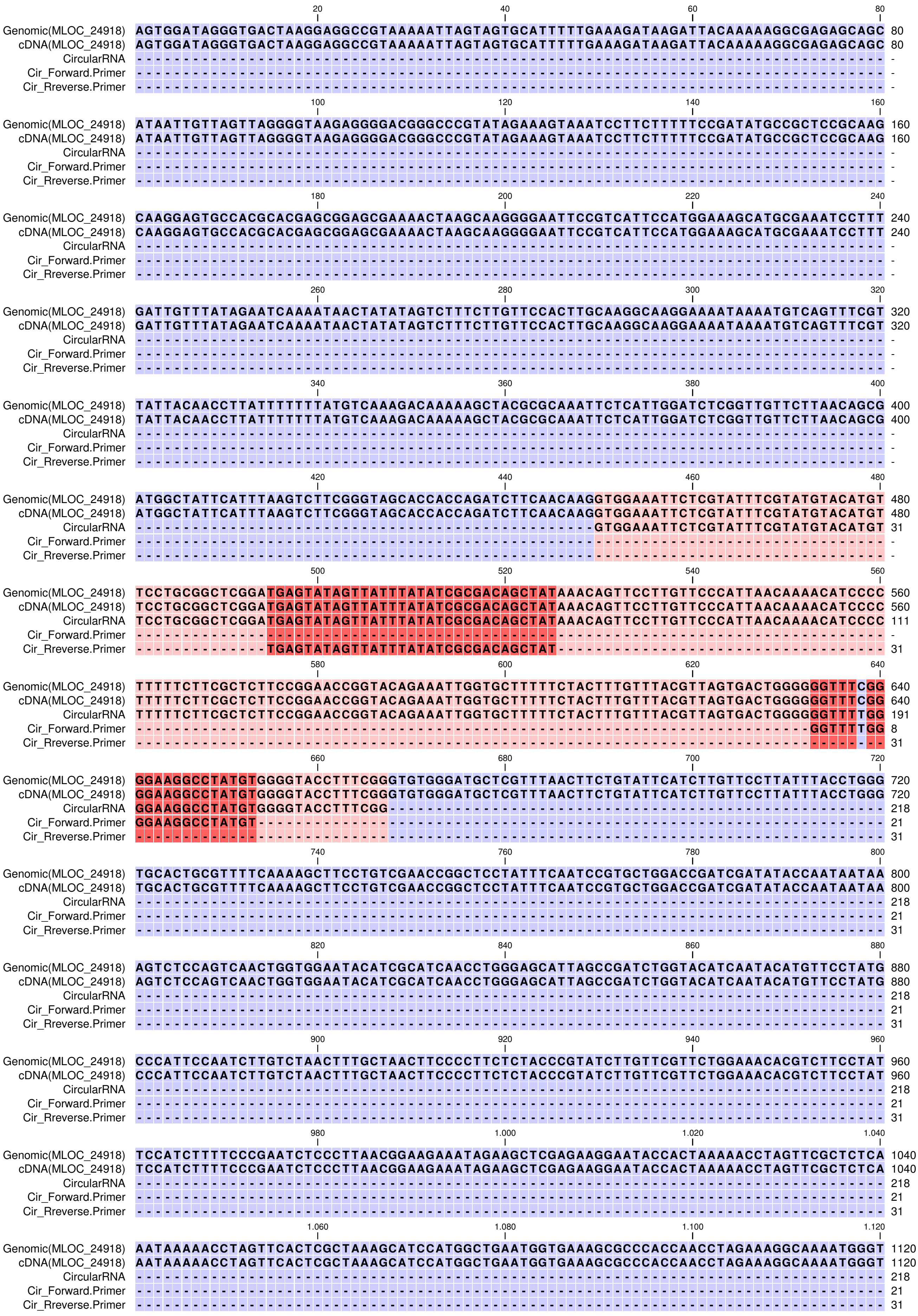

Genomic(MLOC_24918)
cDNA(MLOC_24918)
CircularRNA
Cir_Forward.Primer
Cir_Rreverse.Primer

```
                                         1.140               1.160               1.180               1.200
                                             |                   |                   |                   |
Genomic(MLOC_24918)  CAAAAAGTAGGTTCGATTCCTGCCGGATGCACTGCCTCTTAAAGACAGAAACAGAGGAGGGAAAGAAGGTAGTATAGGGA 1200
cDNA(MLOC_24918)     CAAAAAGTAGGTTCGATTCCTGCCGGATGCACTGCCTCTTAAAGACAGAAACAGAGGAGGGAAAGAAGGTAGTATAGGGA 1200
CircularRNA          -------------------------------------------------------------------------------- 218
Cir_Forward.Primer   -------------------------------------------------------------------------------- 21
Cir_Rreverse.Primer  -------------------------------------------------------------------------------- 31

                                         1.220               1.240               1.260               1.280
                                             |                   |                   |                   |
Genomic(MLOC_24918)  ACTTGCTTTTGGATTCTTATCGAAAGTGAATCCCTCTAACACTTCTGTTAGTGTTTTATGAGAAAATCTTTTATAGACAC 1280
cDNA(MLOC_24918)     ACTTGCTTTTGGATTCTTATCGAAAGTGAATCCCTCTAACACTTCTGTTAGTGTTTTATGAGAAAATCTTTTATAGACAC 1280
CircularRNA          -------------------------------------------------------------------------------- 218
Cir_Forward.Primer   -------------------------------------------------------------------------------- 21
Cir_Rreverse.Primer  -------------------------------------------------------------------------------- 31

                                         1.300               1.320               1.340               1.360
                                             |                   |                   |                   |
Genomic(MLOC_24918)  AACAAGTGTGAAAATCCTTAGTCACTAGGCAAGAAAGCTCGATGGGAACAAAAAGGATACAATTAGTTCAGAATCAAAAA 1360
cDNA(MLOC_24918)     AACAAGTGTGAAAATCCTTAGTCACTAGGCAAGAAAGCTCGATGGGAACAAAAAGGATACAATTAGTTCAGAATCAAAAA 1360
CircularRNA          -------------------------------------------------------------------------------- 218
Cir_Forward.Primer   -------------------------------------------------------------------------------- 21
Cir_Rreverse.Primer  -------------------------------------------------------------------------------- 31

                                         1.380               1.400               1.420               1.440
                                             |                   |                   |                   |
Genomic(MLOC_24918)  AATGTACAATTTCAAAGGAAAGAAGGAATGGCAAGTTTCCCTCCATTGAACATCAATCAATCTAGAAATAGGTAAAGGAA 1440
cDNA(MLOC_24918)     AATGTACAATTTCAAAGGAAAGAAGGAATGGCAAGTTTCCCTCCATTGAACATCAATCAATCTAGAAATAGGTAAAGGAA 1440
CircularRNA          -------------------------------------------------------------------------------- 218
Cir_Forward.Primer   -------------------------------------------------------------------------------- 21
Cir_Rreverse.Primer  -------------------------------------------------------------------------------- 31

                                         1.460               1.480               1.500               1.520
                                             |                   |                   |                   |
Genomic(MLOC_24918)  GGCGTATCACAGGGGTATTTTTCTTTTTTCTATTCACTTCCGTGGGGTTCTAAATTGAAAACATGAACACAAACGAAATC 1520
cDNA(MLOC_24918)     GGCGTATCACAGGGGTATTTTTCTTTTTTCTATTCACTTCCGTGGGGTTCTAAATTGAAAACATGAACACAAACGAAATC 1520
CircularRNA          -------------------------------------------------------------------------------- 218
Cir_Forward.Primer   -------------------------------------------------------------------------------- 21
Cir_Rreverse.Primer  -------------------------------------------------------------------------------- 31

                                         1.540
                                             |
Genomic(MLOC_24918)  GTATTGAAATTACATAAAAAAAGAAAAGGGAGG 1553
cDNA(MLOC_24918)     GTATTGAAATTACATAAAAAAAGAAAAGGGAGG 1553
CircularRNA          --------------------------------- 218
Cir_Forward.Primer   --------------------------------- 21
Cir_Rreverse.Primer  --------------------------------- 31
```

Real-Time PCR for the junction region of ATP-binding cassette

Abcl3_circular RNA (ID: Morex_contig1661226:956-1173)

By divergent primers ◀▶ on genomic DNA

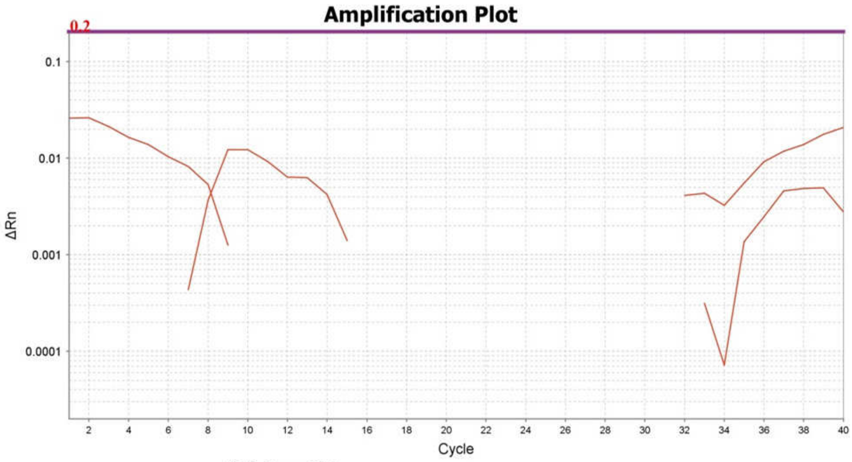

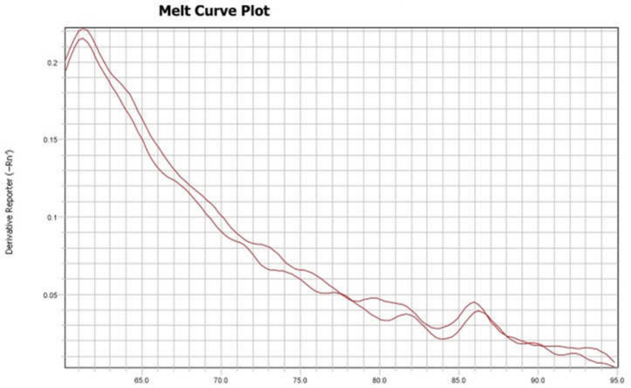

By divergent primers ◀▶ on cDNAs

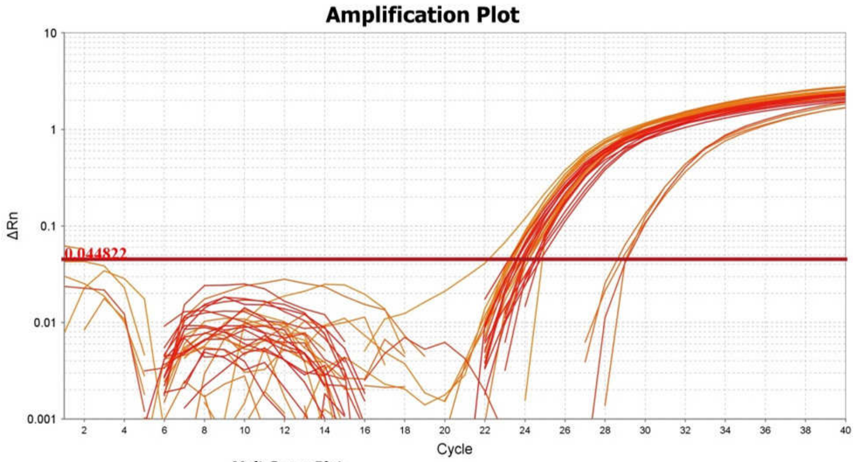

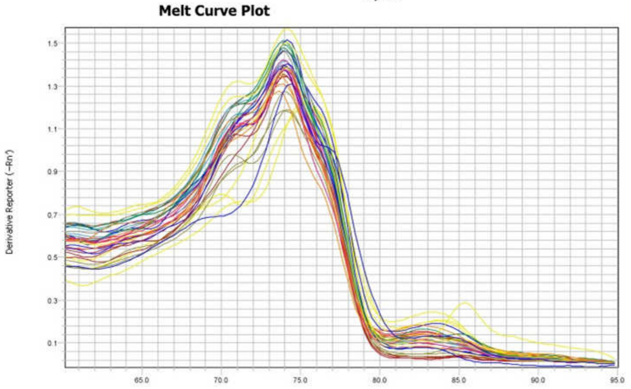

Real-Time PCR for the junction region of ATP-binding cassette

Abcl3_circular RNA (ID: Morex_contig1661226:956-1173**)**

By divergent primers ◀▶ & with no template

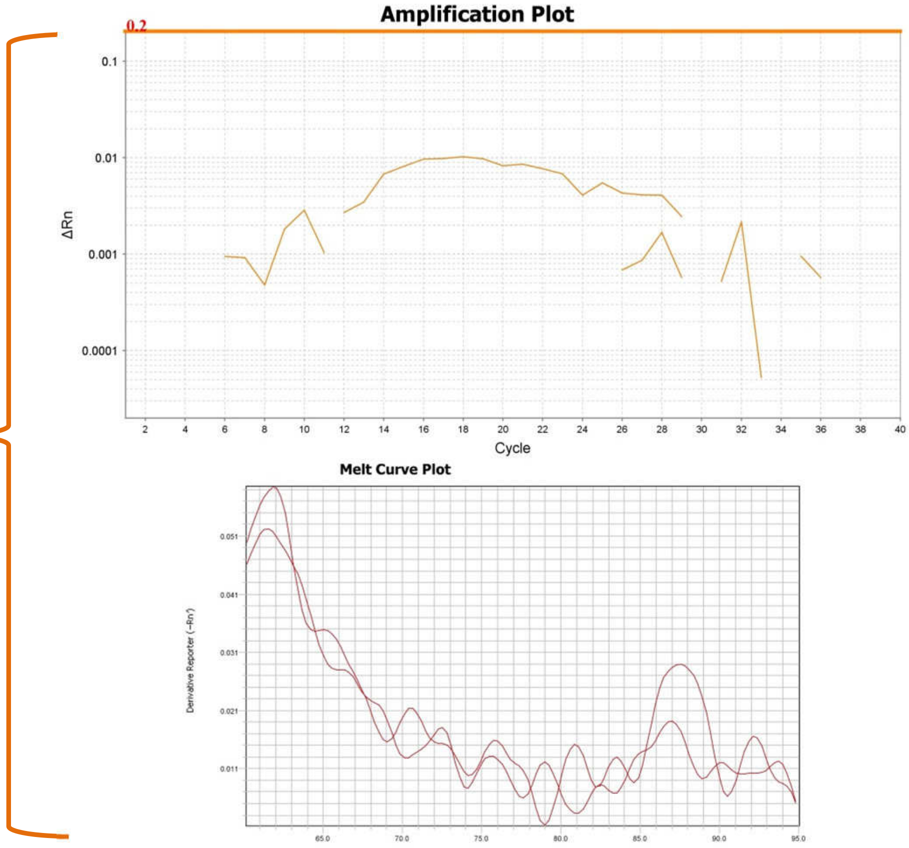

Real-Time PCR for ATP-binding cassette Abcl3

By convergent primers ▶◀ on cDNAs

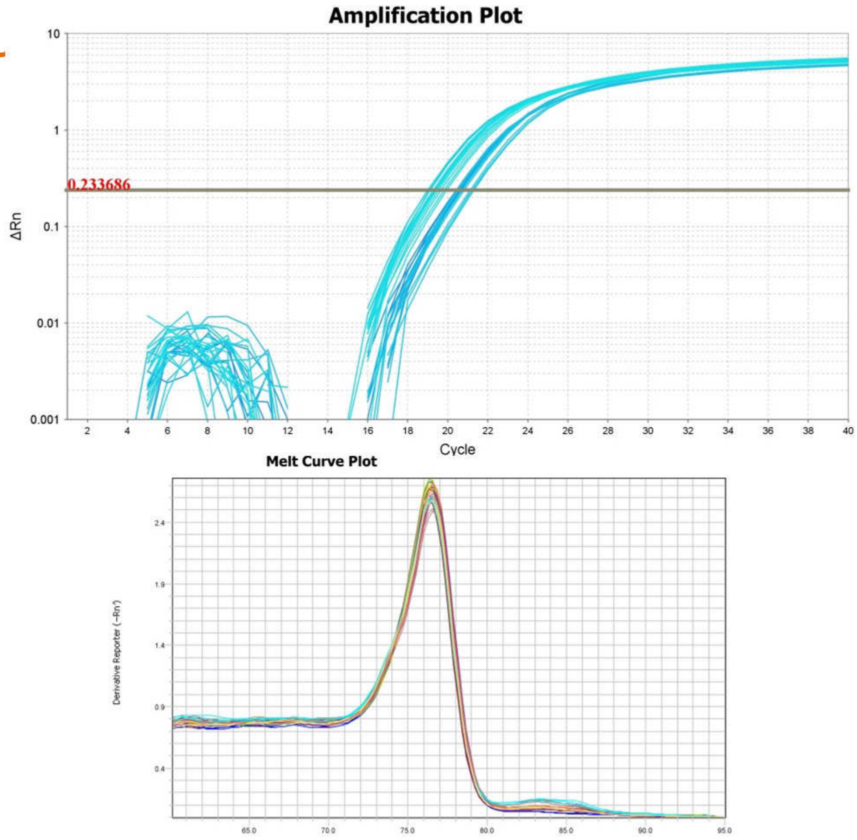

By convergent primers ▶◀ & with no template

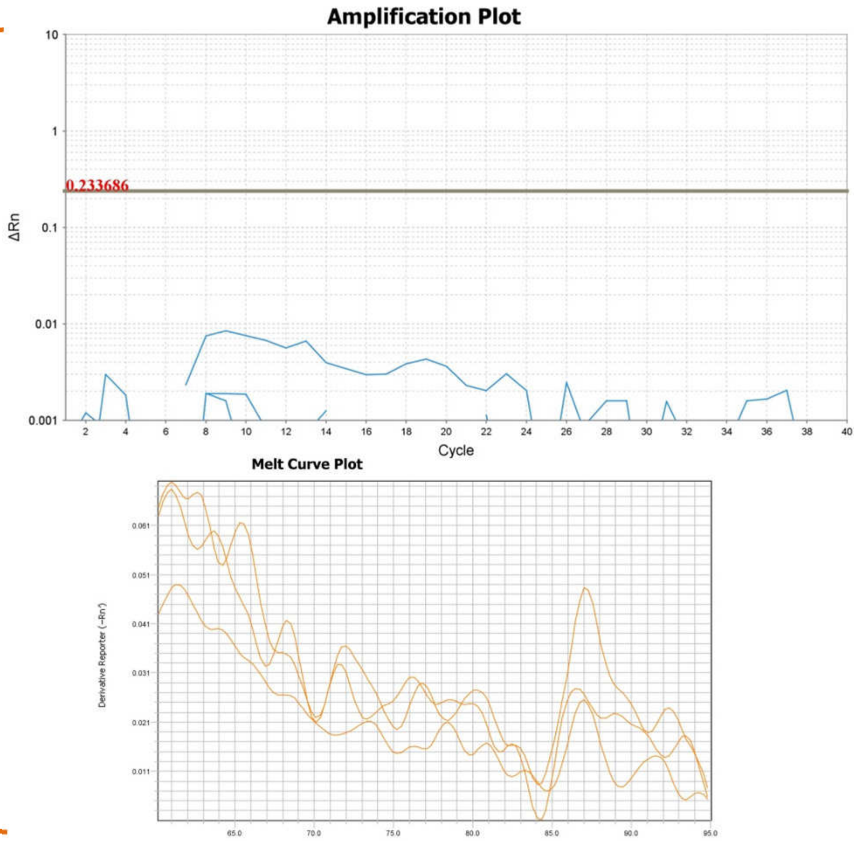

18S rRNA_circular RNA1 (ID: Ch1:429515268-429515439)

TTACCAGGTCCAGACATAGCAAGGATTGACAGACTGAGAGCTCTTTCTTGATTCTATGGGTGG
TGGTGCATGGCC**GTTCTTAGTTGGTGGAGCGATTTGTCTGGTTAATTCCGTTAACGAACGAGA**
CCTCAGCCTGCTAACTAGCTATGCGGAGCCATCCCTCCGCAGCTAG

18S rRNA_circular RNA2 (ID: Ch1:429515283-429515435)

ATAGCAAGGATTGACAGACTGAGAGCTCTTTCTTGATTCTATGGGTGGTGGTGCATGGCCGTT
CTTAGTTGGTGGA**GCGATTTGTCTGGTTAATTCCGTTAACGAACGAGACCTCAGCCTGCTAAC**
TAGCTATGCGGAGCCATCCCTCCGC

18S rRNA_circular RNA3 (ID: Ch1:429515253-429515394)

AACACGGGGAAACTTACCAGGTCCAGACATAGCAAGGATTGACAGACTGAGAGCTCTTTCTTG
ATTCTATGGGTGG**TGGTGCATGGCCGTTCTTAGTTGGTGGAGCGATTTGTCTGGTTAATTCCGT**
TAACGAACGAGACCTCA

18S rRNA_circular RNA4 (ID: Ch1:429515283-429515394)

ATAGCAAGGATTGACAGACTGAGAGCTCTTTCTTGATTCTATGGGTGGTGGTGCATGGCCGTT
CTTAGTTGGTGGAGCGATTTGTCTGGTTAATTCCGTTAACGAACGAGAC

18S rRNA_circular RNA5 (ID: Ch1:429515275-429515370)

GTCCAGACATAGCAAGGATTGACAGACTGAGAGCTCTTTCTTGATTCTATGGGTGGTGGTGCA
TGGCCGTTCTTAGTTGGTGGAGCGATTTGTCTG

18S rRNA_circular RNA6 (ID: Ch1:429515253-429515403)

TCAACACGGGGAAACTTACCAGGTCCAGACATAGCAAGGATTGACAGACTGAGAGCTCTTTCT
TGATTCTATGGGTGGTGGTGCATGGCCGTTCTTAGTTGGTGGAGCGAT**TTGTCTGGTTAATTCC**
GTTAACGAACGAGACCTCAG

18S rRNA_circular RNA7 (ID: Ch1:429513196-429513343)

TTAAAAAGCTCGTAGTTGGACCTTGGGCCGGGTCGGCCGGTCCGCCTCACGGCGAGCACCGA
CCTACTCGACCCTTCGGCCGGCATCGCGCTCCTAGCCTTAATTGGCCGGGTCGTGTTTTCGGC
ATCGTTACTTTGAAGAAATTAGA

The nucleotides of junction-region are underlined. The nucleotides of junction-region which are supported by the junction-spaning sequencing reads are shown in red. Introns are not shown if the absence is supported by sequencing reads. In the absence of supporting sequencing reads, the intronic nucletides are shown as N.

Structural relationship between the circular RNA and its parental gene

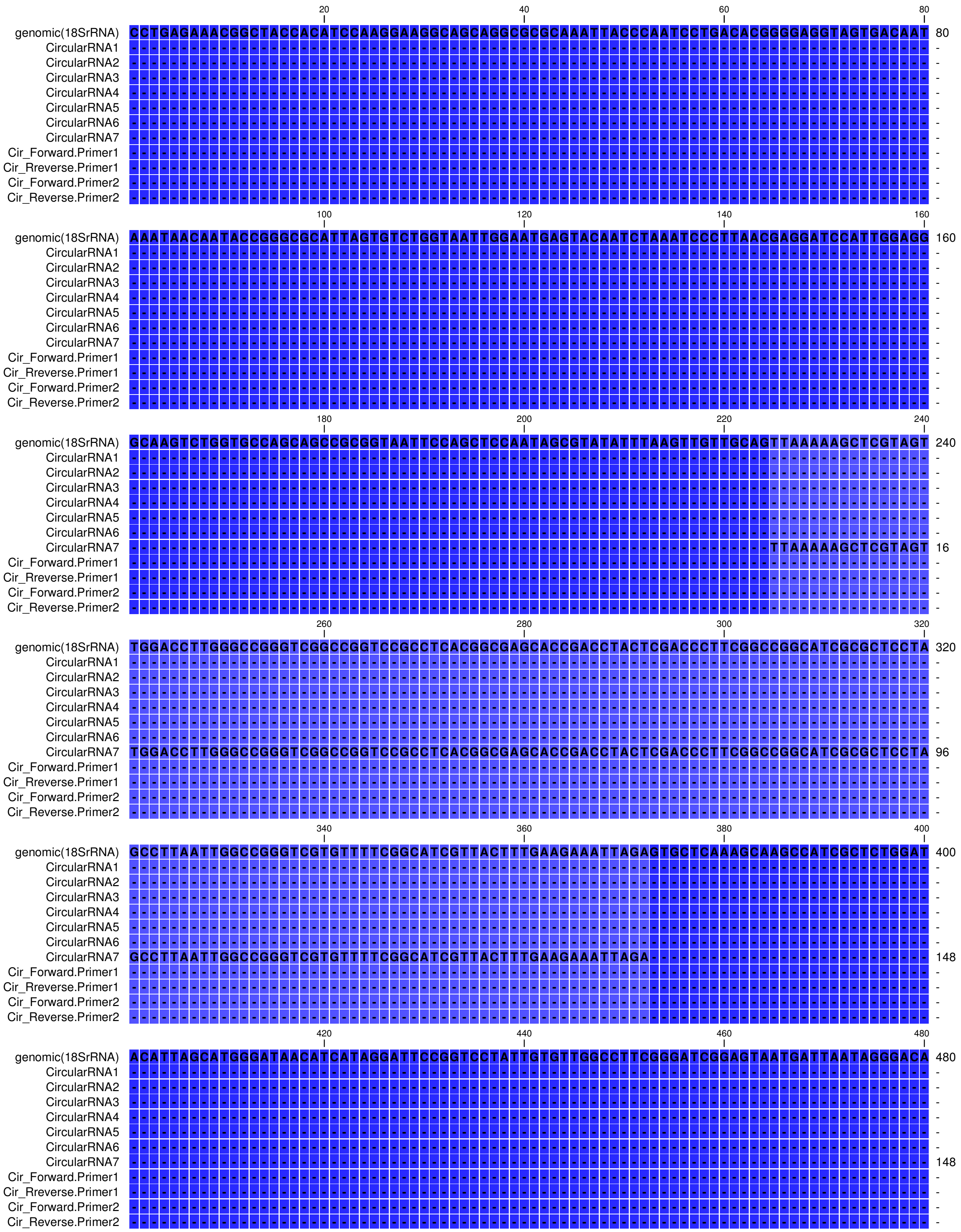
20
40
60
80
genomic(18SrRNA) CCTGAGAAACGGCTACCACATCCAAGGAAGGCAGCAGGCGCGCAAATTACCCAATCCTGACACGGGGAGGTAGTGACAAT 80
CircularRNA1
CircularRNA2
CircularRNA3
CircularRNA4
CircularRNA5
CircularRNA6
CircularRNA7
Cir_Forward.Primer1
Cir_Rreverse.Primer1
Cir_Forward.Primer2
Cir_Reverse.Primer2
100
120
140
160
genomic(18SrRNA) AAATAACAATACCGGGCGCATTAGTGTCTGGTAATTGGAATGAGTACAATCTAAATCCCTTAACGAGGATCCATTGGAGG 160
180
200
220
240
genomic(18SrRNA) GCAAGTCTGGTGCCAGCAGCCGCGGTAATTCCAGCTCCAATAGCGTATATTTAAGTTGTTGCAGTTAAAAAGCTCGTAGT 240
CircularRNA7 TTAAAAAGCTCGTAGT 16
260
280
300
320
genomic(18SrRNA) TGGACCTTGGGCCGGGTCGGCCGGTCCGCCTCACGGCGAGCACCGACCTACTCGACCCTTCGGCCGGCATCGCGCTCCTA 320
CircularRNA7 TGGACCTTGGGCCGGGTCGGCCGGTCCGCCTCACGGCGAGCACCGACCTACTCGACCCTTCGGCCGGCATCGCGCTCCTA 96
340
360
380
400
genomic(18SrRNA) GCCTTAATTGGCCGGGTCGTGTTTTCGGCATCGTTACTTTGAAGAAATTAGAGTGCTCAAAGCAAGCCATCGCTCTGGAT 400
CircularRNA7 GCCTTAATTGGCCGGGTCGTGTTTTCGGCATCGTTACTTTGAAGAAATTAGA 148
420
440
460
480
genomic(18SrRNA) ACATTAGCATGGGATAACATCATAGGATTCCGGTCCTATTGTGTTGGCCTTCGGGATCGGAGTAATGATTAATAGGGACA 480
CircularRNA7 148

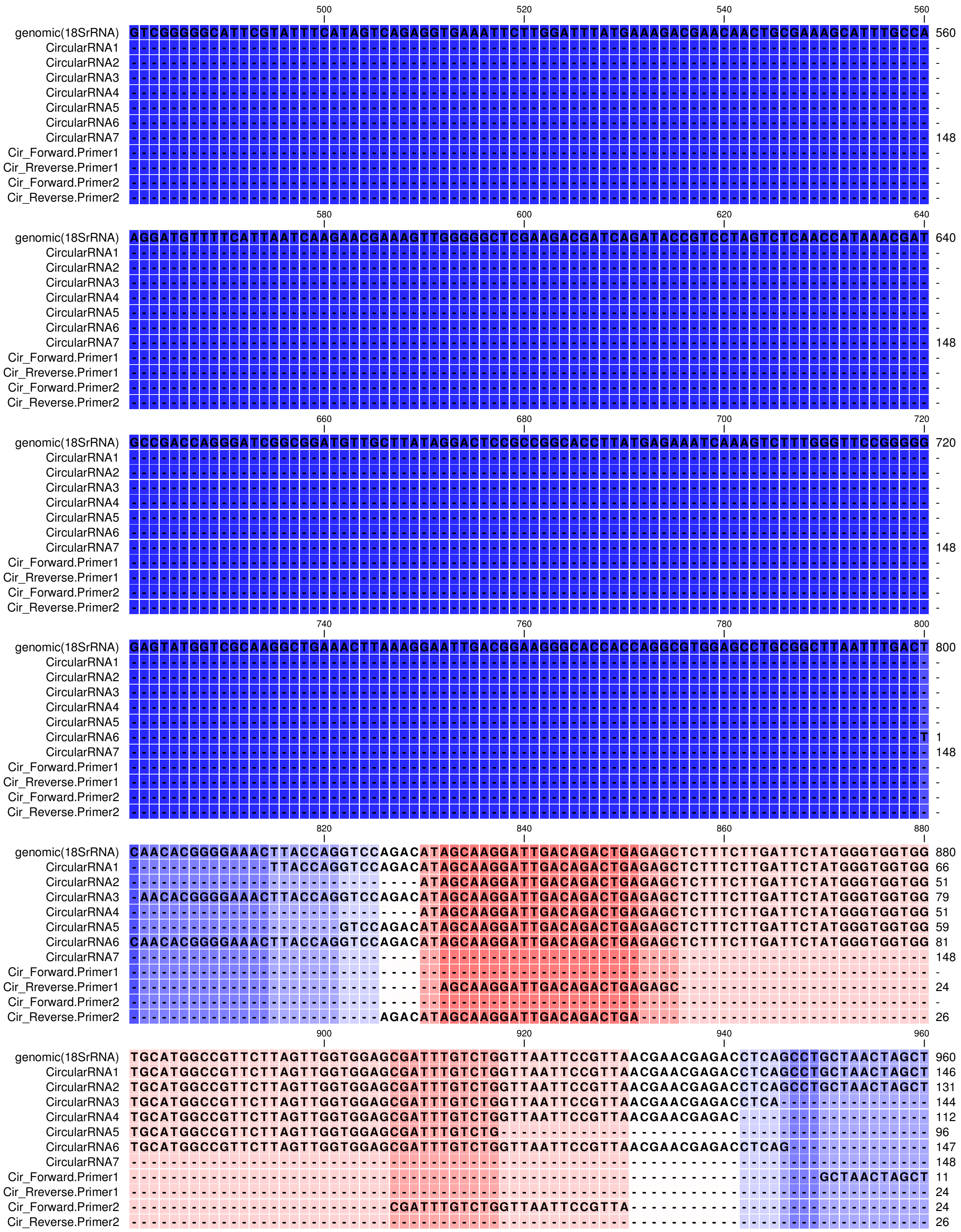

500 520 540 560
genomic(18SrRNA) GTCGGGGGCATTCGTATTTCATAGTCAGAGGTGAAATTCTTGGATTTATGAAAGACGAACAACTGCGAAAGCATTTGCCA 560
CircularRNA1
CircularRNA2
CircularRNA3
CircularRNA4
CircularRNA5
CircularRNA6
CircularRNA7 148
Cir_Forward.Primer1
Cir_Rreverse.Primer1
Cir_Forward.Primer2
Cir_Reverse.Primer2
580 600 620 640
genomic(18SrRNA) AGGATGTTTTCATTAATCAAGAACGAAAGTTGGGGGCTCGAAGACGATCAGATACCGTCCTAGTCTCAACCATAAACGAT 640
660 680 700 720
genomic(18SrRNA) GCCGACCAGGGATCGGCGGATGTTGCTTATAGGACTCCGCCGGCACCTTATGAGAAATCAAAGTCTTTGGGTTCCGGGGG 720
740 760 780 800
genomic(18SrRNA) GAGTATGGTCGCAAGGCTGAAACTTAAAGGAATTGACGGAAGGGCACCACCAGGCGTGGAGCCTGCGGCTTAATTTGACT 800
CircularRNA6 T 1
820 840 860 880
genomic(18SrRNA) CAACACGGGGAAACTTACCAGGTCCAGACATAGCAAGGATTGACAGACTGAGAGCTCTTTCTTGATTCTATGGGTGGTGG 880
CircularRNA1 TTACCAGGTCCAGACATAGCAAGGATTGACAGACTGAGAGCTCTTTCTTGATTCTATGGGTGGTGG 66
CircularRNA2 ATAGCAAGGATTGACAGACTGAGAGCTCTTTCTTGATTCTATGGGTGGTGG 51
CircularRNA3 AACACGGGGAAACTTACCAGGTCCAGACATAGCAAGGATTGACAGACTGAGAGCTCTTTCTTGATTCTATGGGTGGTGG 79
CircularRNA4 ATAGCAAGGATTGACAGACTGAGAGCTCTTTCTTGATTCTATGGGTGGTGG 51
CircularRNA5 GTCCAGACATAGCAAGGATTGACAGACTGAGAGCTCTTTCTTGATTCTATGGGTGGTGG 59
CircularRNA6 CAACACGGGGAAACTTACCAGGTCCAGACATAGCAAGGATTGACAGACTGAGAGCTCTTTCTTGATTCTATGGGTGGTGG 81
CircularRNA7 148
Cir_Rreverse.Primer1 AGCAAGGATTGACAGACTGAGAGC 24
Cir_Reverse.Primer2 AGACATAGCAAGGATTGACAGACTGA 26
900 920 940 960
genomic(18SrRNA) TGCATGGCCGTTCTTAGTTGGTGGAGCGATTTGTCTGGTTAATTCCGTTAACGAACGAGACCTCAGCCTGCTAACTAGCT 960
CircularRNA1 TGCATGGCCGTTCTTAGTTGGTGGAGCGATTTGTCTGGTTAATTCCGTTAACGAACGAGACCTCAGCCTGCTAACTAGCT 146
CircularRNA2 TGCATGGCCGTTCTTAGTTGGTGGAGCGATTTGTCTGGTTAATTCCGTTAACGAACGAGACCTCAGCCTGCTAACTAGCT 131
CircularRNA3 TGCATGGCCGTTCTTAGTTGGTGGAGCGATTTGTCTGGTTAATTCCGTTAACGAACGAGACCTCA 144
CircularRNA4 TGCATGGCCGTTCTTAGTTGGTGGAGCGATTTGTCTGGTTAATTCCGTTAACGAACGAGAC 112
CircularRNA5 TGCATGGCCGTTCTTAGTTGGTGGAGCGATTTGTCTG 96
CircularRNA6 TGCATGGCCGTTCTTAGTTGGTGGAGCGATTTGTCTGGTTAATTCCGTTAACGAACGAGACCTCAG 147
CircularRNA7 148
Cir_Forward.Primer1 GCTAACTAGCT 11
Cir_Rreverse.Primer1 24
Cir_Forward.Primer2 CGATTTGTCTGGTTAATTCCGTTA 24
Cir_Reverse.Primer2 26

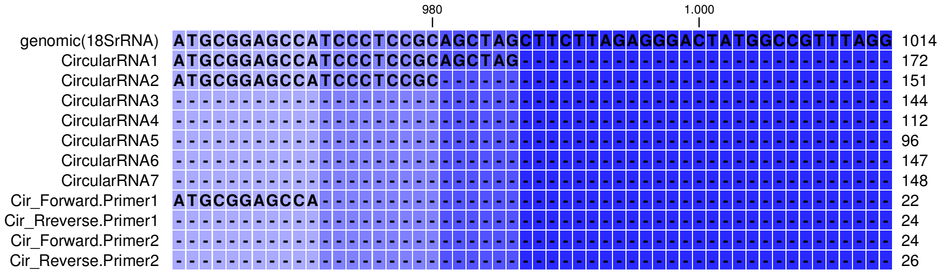
980
1.000
genomic(18SrRNA) ATGCGGAGCCATCCCTCCGCAGCTAGCTTCTTAGAGGGACTATGGCCGTTTAGG 1014
CircularRNA1 ATGCGGAGCCATCCCTCCGCAGCTAG 172
CircularRNA2 ATGCGGAGCCATCCCTCCGC 151
CircularRNA3 144
CircularRNA4 112
CircularRNA5 96
CircularRNA6 147
CircularRNA7 148
Cir_Forward.Primer1 ATGCGGAGCCA 22
Cir_Rreverse.Primer1 24
Cir_Forward.Primer2 24
Cir_Reverse.Primer2 26

Real-Time PCR for the junction region of 18S rRNA_circular RNA1 & 2

(ID: Ch1:429515268-429515439; Ch1:429515283-429515435)

By divergent primers ◀▶ no. 1 on genomic DNA

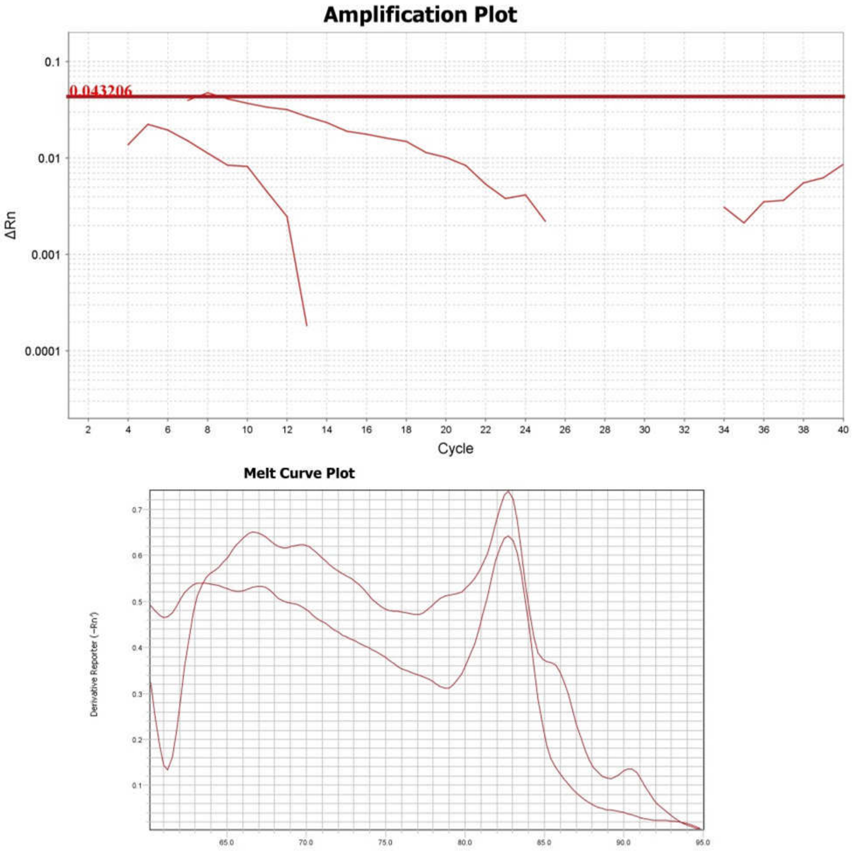

By divergent primers ◀▶ no. 1 on cDNAs

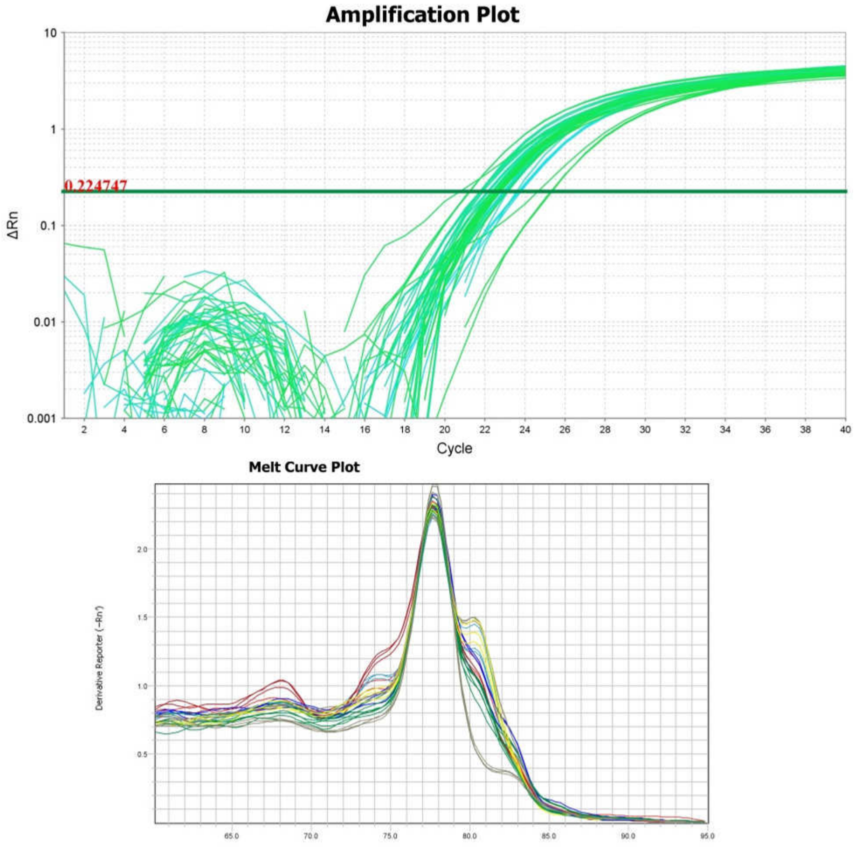

Real-Time PCR for the junction region of 18S rRNA_circular RNA1 & 2

(ID: Ch1:429515268-429515439; Ch1:429515283-429515435**)**

By divergent primers ◀▶ no. 1 with no template

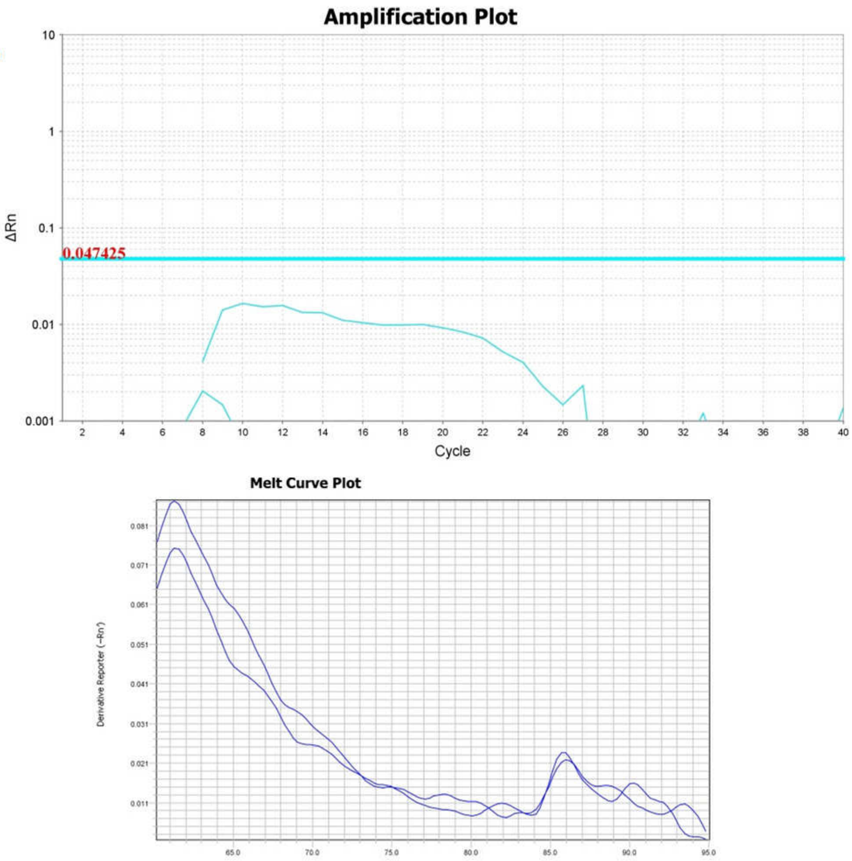

Real-Time PCR for the junction region of 18S rRNA_circular RNA1, 3, 4, & 6 (ID: Ch1:429515268-429515439; Ch1:429515253-429515394; Ch1:429515283-429515394; Ch1:429515253-429515403**)**

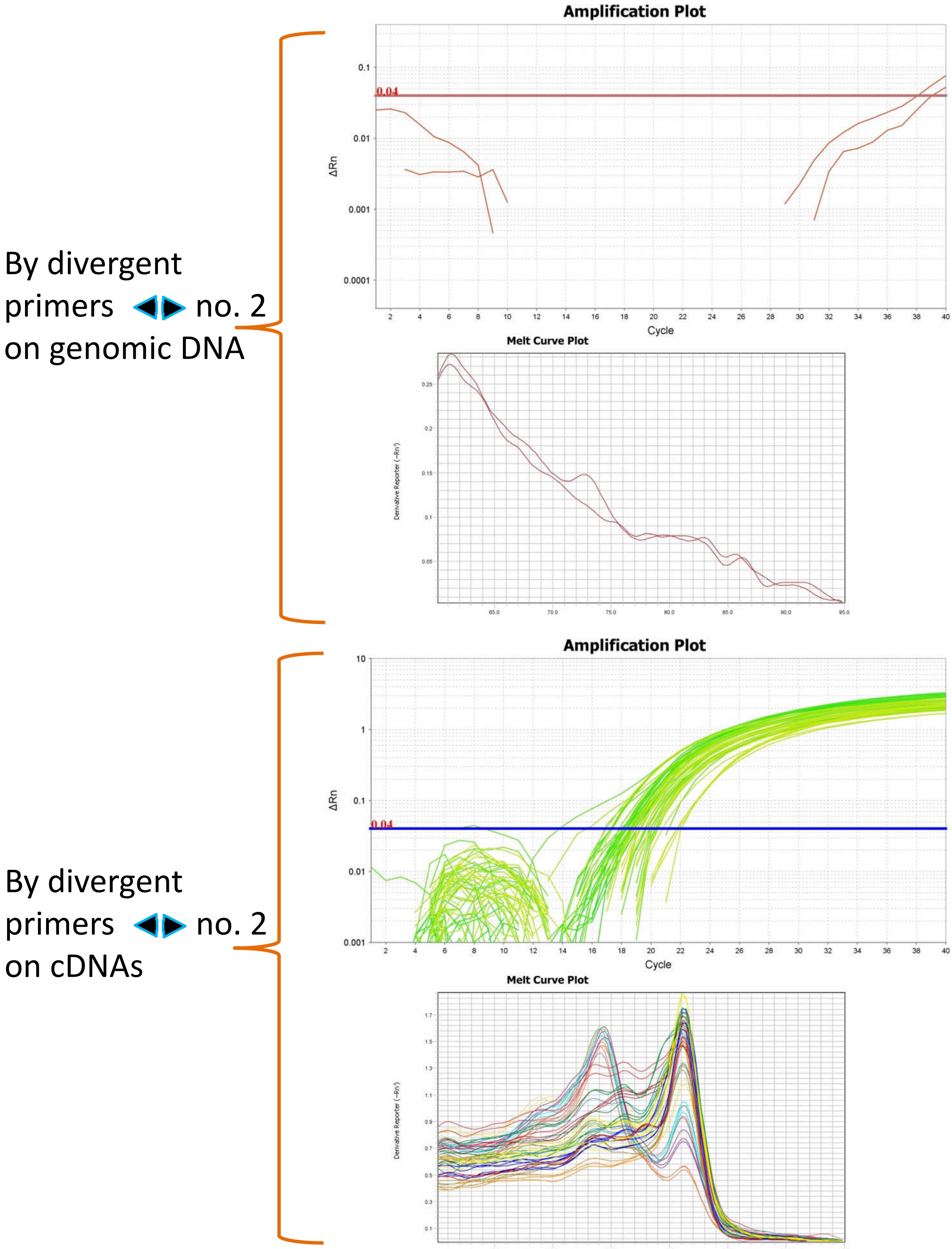

Real-Time PCR for the junction region of 18S rRNA_circular RNA1, 3, 4, & 6 (ID: Ch1:429515268-429515439; Ch1:429515253-429515394; Ch1:429515283-429515394; Ch1:429515253-429515403**)**

By divergent primers ◀▶ no. 2 with no template

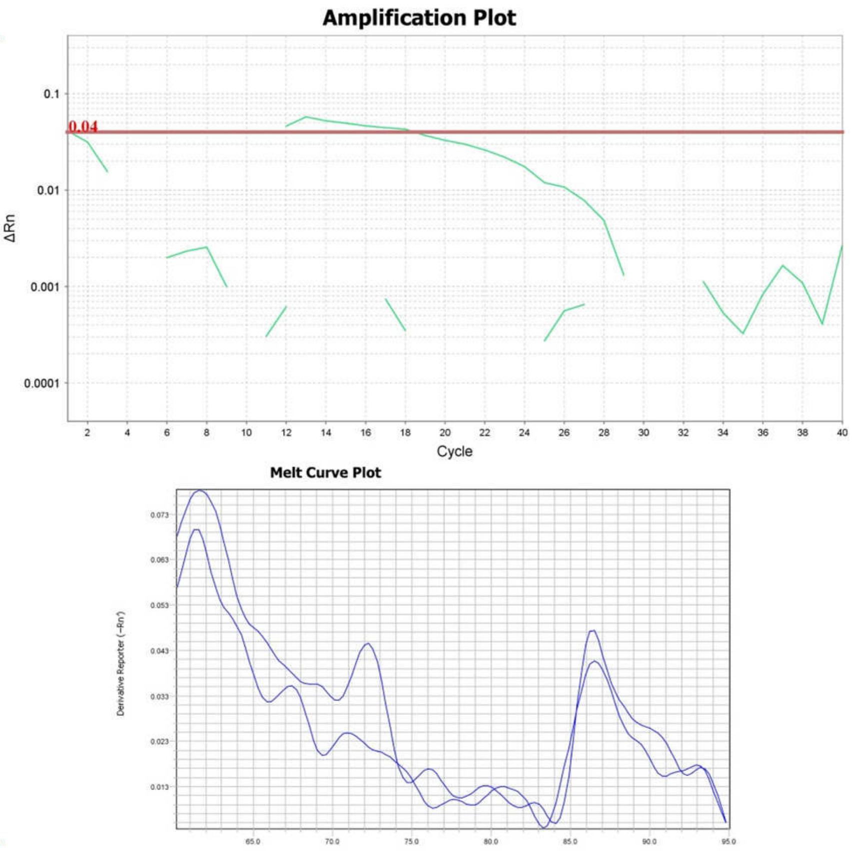

Real-Time PCR for 18S rRNA

By convergent primers ▶◀ on cDNAs

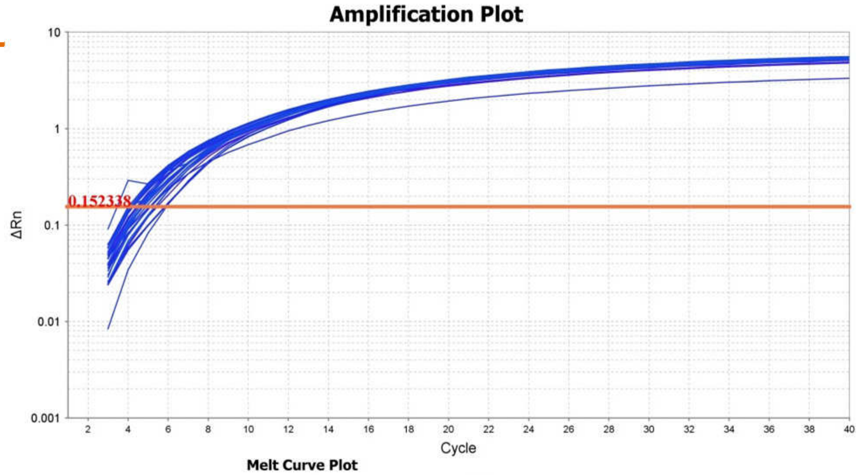

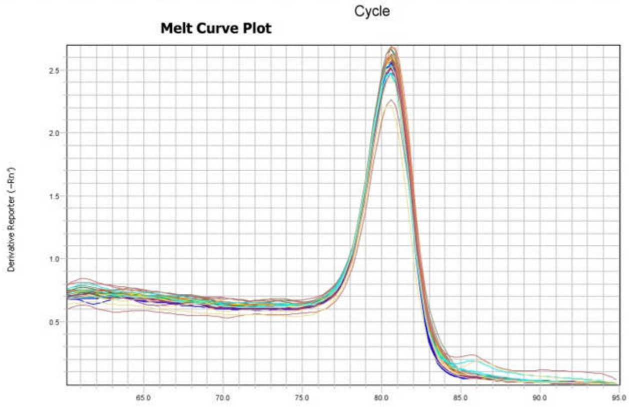

By convergent primers ▶◀ & with no template

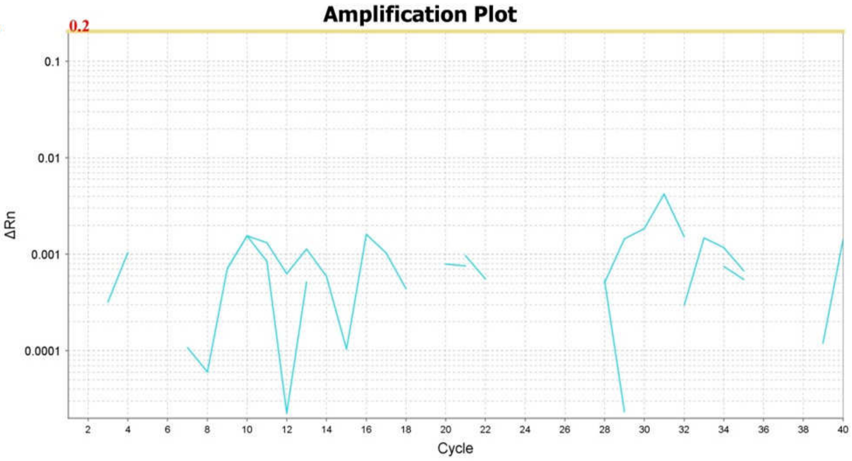

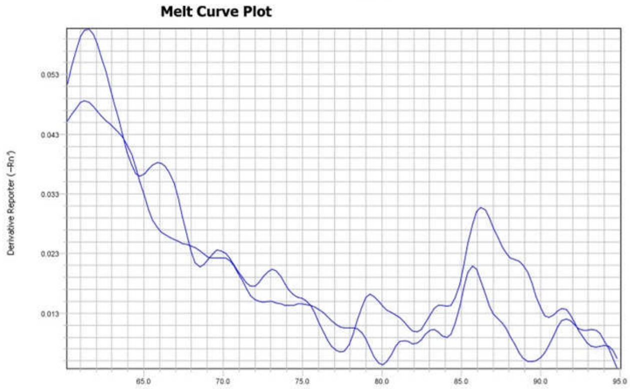

Internal transcribed spacer 2 of 18S,5.8S,26S rRNA_circular RNA1
(ID: Ch1:429512475-429512569)

<u>T</u>CTAGTCCGACTTTGTGAAATGACTTGAGAGGTGTAGGATAAGTGGGAGCCCTTACGGGCGC
AAGTGAAATACCACTACTTTTAACGTACACCT<u>C</u>

Internal transcribed spacer 2 of 18S,5.8S,26S rRNA_circular RNA2
(ID: Ch1:177434203-177434347)

<u>G</u>GGTCCTAAGATGAGCTCAACAAGAACACAAATCTCATGTGGAACAAAAGGGTAAAAGATCG
TTTGGTTCTGAATTCCAGTATGAATAAGAACCATGGAAGCATGGCCTATCGATCCTTTAGATCT
TCGGAGTTTGAAGCTAGA<u>G</u>

The nucleotides of junction-region are underlined. The nucleotides of junction-region which are supported by the junction-spaning sequencing reads are shown in red. Introns are not shown if the absence is supported by sequencing reads. In the absence of supporting sequencing reads, the intronic nucletides are shown as N.

NADH dehydrogenase Nad9_circular RNA (ID: Morex_contig_70567:2809-3204**)**

GTTTCGATCGATATTTGCGGAGTGGATCATCCCTCTCGAAAACGCAGATTTGAAGTTGTCCATAA
TTTACTGAGTACTCGGTATAACTCACGCATTCGTGTACAAACAAGTGCAGACGAAGTAACACGAAT
ATCTCCGGTAGTCAGTCTATTTCCATCAGCCGGCCGGTGGGAGCGAGAAGTATGGGATATGTCTG
GTGTTTCTTCCATCAATCATCCGGATTTACGCCGTATATCAACAGATTATGGTTTCGAGGGTCATC
CATTACGAAAAGACTTTCCTCTGAGTGGATATGTGGAAGTACGCTATGATGATCCAGAGAAACGTG
TGGTTTCTGAACCCATTGAGATGACCCAAGAA**TTTCGCTATTTCGATTTTGCTAGTCCTTGGGAAC
AG**

The nucleotides of junction-region are underlined. The nucleotides of junction-region which are supported by the junction-spaning sequencing reads are shown in red. Introns are not shown if the absence is supported by sequencing reads. In the absence of supporting sequencing reads, the intronic nucletides are shown as N.

Structural relationship between the circular RNA and its parental gene

```
                                        20                  40                  60                  80
Genomic(MLOC_76215)  GGATGATCGGCAGAAAAGAGTAATAAACTTTTTGACAAATCACGCTCATGAAAATGCAATACACCTGAACGGTAGATTCT  80
cDNA(MLOC_76215)     GGATGATCGGCAGAAAAGAGTAATAAACTTTTTGACAAATCACGCTCATGAAAATGCAATACACCTGAACGGTAGATTCT  80
CircularRNA          --------------------------------------------------------------------------------  -
Cir_Forward.Primer   --------------------------------------------------------------------------------  -
Cir_Reverse.Primer   --------------------------------------------------------------------------------  -

                                       100                 120                 140                 160
Genomic(MLOC_76215)  ACCACGGAAGTCCATGAATGCTGGCAAATAGAATTGATAACCTTCGTATGCTGACGCAAGTCTTATTAAAAAATCTTCAT  160
cDNA(MLOC_76215)     ACCACGGAAGTCCATGAATGCTGGCAAATAGAATTGATAACCTTCGTATGCTGACGCAAGTCTTATTAAAAAATCTTCAT  160
CircularRNA          --------------------------------------------------------------------------------  -
Cir_Forward.Primer   --------------------------------------------------------------------------------  -
Cir_Reverse.Primer   --------------------------------------------------------------------------------  -

                                       180                 200                 220                 240
Genomic(MLOC_76215)  AACTAGCCTGCTGCACCTGTTTTCATAATTTATTTAACAGTATGTTATAGCTACAAGCATTCTTAATACCTTTGACGTTA  240
cDNA(MLOC_76215)     AACTAGCCTGCTGCACCTGTTTTCATAATTTATTTAACAGTATGTTATAGCTACAAGCATTCTTAATACCTTTGACGTTA  240
CircularRNA          --------------------------------------------------------------------------------  -
Cir_Forward.Primer   --------------------------------------------------------------------------------  -
Cir_Reverse.Primer   --------------------------------------------------------------------------------  -

                                       260                 280                 300                 320
Genomic(MLOC_76215)  TTGAAGTAGCACTCCCTCAATATGTCTGAGGCTTCTGTCGGATTCACGCGAGCCAGAGCATTTGGTAAAAGAAGCCCACA  320
cDNA(MLOC_76215)     TTGAAGTAGCACTCCCTCAATATGTCTGAGGCTTCTGTCGGATTCACGCGAGCCAGAGCATTTGGTAAAAGAAGCCCACA  320
CircularRNA          --------------------------------------------------------------------------------  -
Cir_Forward.Primer   --------------------------------------------------------------------------------  -
Cir_Reverse.Primer   --------------------------------------------------------------------------------  -

                                       340                 360                 380                 400
Genomic(MLOC_76215)  CTCAACTAAACATTGACGATTCTTTTCCAGAAATCTCAAGAGCCTTTTGTTTATTTCAAACGCTTGAGACTGAAGCCCAT  400
cDNA(MLOC_76215)     CTCAACTAAACATTGACGATTCTTTTCCAGAAATCTCAAGAGCCTTTTGTTTATTTCAAACGCTTGAGACTGAAGCCCAT  400
CircularRNA          --------------------------------------------------------------------------------  -
Cir_Forward.Primer   --------------------------------------------------------------------------------  -
Cir_Reverse.Primer   --------------------------------------------------------------------------------  -

                                       420                 440                 460                 480
Genomic(MLOC_76215)  TCAAAATATCACACATCTGCTTGTATTCTTTTTTATGTAACTTTATATAGAAGTGGTTTAAGTCATGGGAAGTTAATAGC  480
cDNA(MLOC_76215)     TCAAAATATCACACATCTGCTTGTATTCTTTTTTATGTAACTTTATATAGAAGTGGTTTAAGTCATGGGAAGTTAATAGC  480
CircularRNA          --------------------------------------------------------------------------------  -
Cir_Forward.Primer   --------------------------------------------------------------------------------  -
Cir_Reverse.Primer   --------------------------------------------------------------------------------  -

                                       500                 520                 540                 560
Genomic(MLOC_76215)  CGAAAGTGATTCTAGATATCCCCCGTGGGTTTGCATAAGTAACCTCCTATCAGATCCGATAATGTATCAGGATCAGACCC  560
cDNA(MLOC_76215)     CGAAAGTGATTCTAGATATCCCCCGTGGGTTTGCATAAGTAACCTCCTATCAGATCCGATAATGTATCAGGATCAGACCC  560
CircularRNA          --------------------------------------------------------------------------------  -
Cir_Forward.Primer   --------------------------------------------------------------------------------  -
Cir_Reverse.Primer   --------------------------------------------------------------------------------  -

                                       580                 600                 620                 640
Genomic(MLOC_76215)  CCTCTCTGCTGGTTGCCAATCCAAGGGTTTGCAAAGCATCGGAAGATTTAGTTTGATAGGGAGCAGATTTATGTTCAAAT  640
cDNA(MLOC_76215)     CCTCTCTGCTGGTTGCCAATCCAAGGGTTTGCAAAGCATCGGAAGATTTAGTTTGATAGGGAGCAGATTTATGTTCAAAT  640
CircularRNA          --------------------------------------------------------------------------------  -
Cir_Forward.Primer   --------------------------------------------------------------------------------  -
Cir_Reverse.Primer   --------------------------------------------------------------------------------  -

                                       660                 680                 700                 720
Genomic(MLOC_76215)  TGCACATAACATAACAAGAATTCTCTATATACCCTTGACCTTTCTTAAGAACCAGCACTGGATCTTCTTTTGTTACTCCC  720
cDNA(MLOC_76215)     TGCACATAACATAACAAGAATTCTCTATATACCCTTGACCTTTCTTAAGAACCAGCACTGGATCTTCTTTTGTTACTCCC  720
CircularRNA          --------------------------------------------------------------------------------  -
Cir_Forward.Primer   --------------------------------------------------------------------------------  -
Cir_Reverse.Primer   --------------------------------------------------------------------------------  -

                                       740                 760                 780                 800
Genomic(MLOC_76215)  CTATCTGTTGATATATGTAGAACATTTCTTTCAATCATGAATTGGAGCAAGTATTTCCCGATATCATAATGACACTGAAT  800
cDNA(MLOC_76215)     CTATCTGTTGATATATGTAGAACATTTCTTTCAATCATGAATTGGAGCAAGTATTTCCCGATATCATAATGACACTGAAT  800
CircularRNA          --------------------------------------------------------------------------------  -
Cir_Forward.Primer   --------------------------------------------------------------------------------  -
Cir_Reverse.Primer   --------------------------------------------------------------------------------  -

                                       820                 840                 860                 880
Genomic(MLOC_76215)  TTTTTTCTTTTTCTCTTTTTTGCTAGCACCTTTGGGCTGAACAACATCCTTAACTGATTCTACCTTACTAGTGAGTACTG  880
cDNA(MLOC_76215)     TTTTTTCTTTTTCTCTTTTTTGCTAGCACCTTTGGGCTGAACAACATCCTTAACTGATTCTACCTTACTAGTGAGTACTG  880
CircularRNA          --------------------------------------------------------------------------------  -
Cir_Forward.Primer   --------------------------------------------------------------------------------  -
Cir_Reverse.Primer   --------------------------------------------------------------------------------  -

                                       900                 920                 940                 960
Genomic(MLOC_76215)  CTTCCACCTTACCACTACCTGGTGCTTTGTATTGTAGAAACCTTGCTTGTTCTCGTACAGTTGAATTAAGTTGATCTATA  960
cDNA(MLOC_76215)     CTTCCACCTTACCACTACCTGGTGCTTTGTATTGTAGAAACCTTGCTTGTTCTCGTACAGTTGAATTAAGTTGATCTATA  960
CircularRNA          --------------------------------------------------------------------------------  -
Cir_Forward.Primer   --------------------------------------------------------------------------------  -
Cir_Reverse.Primer   --------------------------------------------------------------------------------  -

                                       980               1.000               1.020               1.040
Genomic(MLOC_76215)  AATCTAGCTGCCTTAACTGCGGATGATTCCTGAAGAGTGTTGAATACCAAGCCTAGTACATGAATCATGATAGCCTCAAG  1040
cDNA(MLOC_76215)     AATCTAGCTGCCTTAACTGCGGATGATTCCTGAAGAGTGTTGAATACCAAGCCTAGTACATGAATCATGATAGCCTCAAG  1040
CircularRNA          --------------------------------------------------------------------------------  -
Cir_Forward.Primer   --------------------------------------------------------------------------------  -
Cir_Reverse.Primer   --------------------------------------------------------------------------------  -

                                     1.060               1.080               1.100               1.120
Genomic(MLOC_76215)  CGTATAGATACCAAACATCTTAAGCATAGGTAAGCTACTTGGTAGGCAACCCTTTAGATATTGCTTAGCGTTAGGCTGAT  1120
cDNA(MLOC_76215)     CGTATAGATACCAAACATCTTAAGCATAGGTAAGCTACTTGGTAGGCAACCCTTTAGATATTGCTTAGCGTTAGGCTGAT  1120
CircularRNA          --------------------------------------------------------------------------------  -
Cir_Forward.Primer   --------------------------------------------------------------------------------  -
Cir_Reverse.Primer   --------------------------------------------------------------------------------  -
```

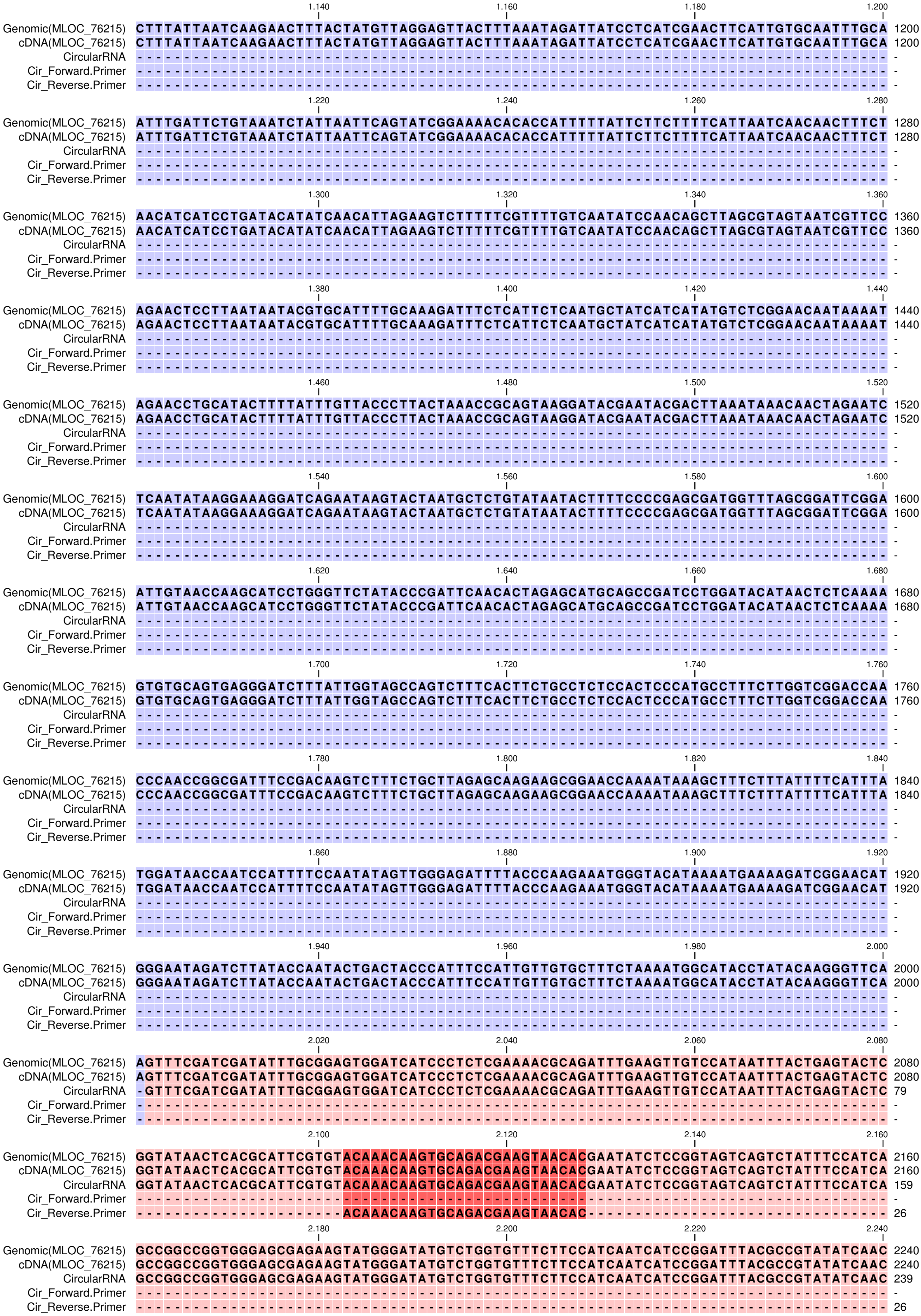

Genomic(MLOC_76215)
cDNA(MLOC_76215)
CircularRNA
Cir_Forward.Primer
Cir_Reverse.Primer
1.140
1.160
1.180
1.200
2.240

```
                          2.260               2.280               2.300               2.320
Genomic(MLOC_76215)  AGATTATGGTTTCGAGGGTCATCCATTACGAAAAGACTTTCCTCTGAGTGGATATGTGGAAGTACGCTATGATGATCCAG 2320
cDNA(MLOC_76215)     AGATTATGGTTTCGAGGGTCATCCATTACGAAAAGACTTTCCTCTGAGTGGATATGTGGAAGTACGCTATGATGATCCAG 2320
CircularRNA          AGATTATGGTTTCGAGGGTCATCCATTACGAAAAGACTTTCCTCTGAGTGGATATGTGGAAGTACGCTATGATGATCCAG 319
Cir_Forward.Primer   -------------------------------------------------------------------------------- -
Cir_Reverse.Primer   -------------------------------------------------------------------------------- 26

                          2.340               2.360               2.380               2.400
Genomic(MLOC_76215)  AGAAACGTGTGGTTTCTGAACCCATTGAGATGACCCAAGAATTTCGCTATTTCGATTTTGCTAGTCCTTGGGAACAGCGT 2400
cDNA(MLOC_76215)     AGAAACGTGTGGTTTCTGAACCCATTGAGATGACCCAAGAATTTCGCTATTTCGATTTTGCTAGTCCTTGGGAACAGCGT 2400
CircularRNA          AGAAACGTGTGGTTTCTGAACCCATTGAGATGACCCAAGAATTTCGCTATTTCGATTTTGCTAGTCCTTGGGAACAG--- 396
Cir_Forward.Primer   ----------------------------------------------------CGATTTTGCTAGTCCTTGGGAA------ 22
Cir_Reverse.Primer   -------------------------------------------------------------------------------- 26

                          2.420               2.440               2.460               2.480
Genomic(MLOC_76215)  AGCGACGGATAATTCCGAATCTACATAGGTCTAGTCCAGGGGACAAATCAATAGGAAATGCTATTTGCTTCTTAAGAAGA 2480
cDNA(MLOC_76215)     AGCGACGGATAATTCCGAATCTACATAGGTCTAGTCCAGGGGACAAATCAATAGGAAATGCTATTTGCTTCTTAAGAAGA 2480
CircularRNA          -------------------------------------------------------------------------------- 396
Cir_Forward.Primer   -------------------------------------------------------------------------------- 22
Cir_Reverse.Primer   -------------------------------------------------------------------------------- 26

                          2.500               2.520               2.540               2.560
Genomic(MLOC_76215)  AGAACTTTTTTGAAATGAAAGAGTTTCCACGGGCGTCGGATATCATTTCTTCAAATAAGGAAGAAGTGTTATCGAAGGAT 2560
cDNA(MLOC_76215)     AGAACTTTTTTGAAATGAAAGAGTTTCCACGGGCGTCGGATATCATTTCTTCAAATAAGGAAGAAGTGTTATCGAAGGAT 2560
CircularRNA          -------------------------------------------------------------------------------- 396
Cir_Forward.Primer   -------------------------------------------------------------------------------- 22
Cir_Reverse.Primer   -------------------------------------------------------------------------------- 26

                          2.580               2.600               2.620               2.640
Genomic(MLOC_76215)  TTCCTTCCATTCTTCCTAGTATGGAAGAAGTAAAGAAAAAGTACTGCGTCTCGATCTATACGAAAGGGCACTGGTTTTTT 2640
cDNA(MLOC_76215)     TTCCTTCCATTCTTCCTAGTATGGAAGAAGTAAAGAAAAAGTACTGCGTCTCGATCTATACGAAAGGGCACTGGTTTTTT 2640
CircularRNA          -------------------------------------------------------------------------------- 396
Cir_Forward.Primer   -------------------------------------------------------------------------------- 22
Cir_Reverse.Primer   -------------------------------------------------------------------------------- 26

                          2.660               2.680               2.700               2.720
Genomic(MLOC_76215)  CTTTCGGTTTCATTGATAGCAGAAGAGTACGCTAGCTAGCGGAGCCTGAAAACGCTTGCTTGCGCCCCACCCCTACGGAA 2720
cDNA(MLOC_76215)     CTTTCGGTTTCATTGATAGCAGAAGAGTACGCTAGCTAGCGGAGCCTGAAAACGCTTGCTTGCGCCCCACCCCTACGGAA 2720
CircularRNA          -------------------------------------------------------------------------------- 396
Cir_Forward.Primer   -------------------------------------------------------------------------------- 22
Cir_Reverse.Primer   -------------------------------------------------------------------------------- 26

                          2.740               2.760               2.780               2.800
Genomic(MLOC_76215)  ACTATTGCCTATGCTTGCTGCTCGCTCAGTTTCAGTAGAAGGGAAGAAAAGATGGTCACTCCTTTTAGGAACATGGCGAG 2800
cDNA(MLOC_76215)     ACTATTGCCTATGCTTGCTGCTCGCTCAGTTTCAGTAGAAGGGAAGAAAAGATGGTCACTCCTTTTAGGAACATGGCGAG 2800
CircularRNA          -------------------------------------------------------------------------------- 396
Cir_Forward.Primer   -------------------------------------------------------------------------------- 22
Cir_Reverse.Primer   -------------------------------------------------------------------------------- 26

                          2.820               2.840               2.860               2.880
Genomic(MLOC_76215)  CCTTACTTACGACTGTTGCACTTCACTCGCTGCTCGTTCATTCACTTGCTCATGAACTCGCTGCTTCGCCAGAAGCGACT 2880
cDNA(MLOC_76215)     CCTTACTTACGACTGTTGCACTTCACTCGCTGCTCGTTCATTCACTTGCTCATGAACTCGCTGCTTCGCCAGAAGCGACT 2880
CircularRNA          -------------------------------------------------------------------------------- 396
Cir_Forward.Primer   -------------------------------------------------------------------------------- 22
Cir_Reverse.Primer   -------------------------------------------------------------------------------- 26

                          2.900               2.920               2.940               2.960
Genomic(MLOC_76215)  AGTCGCCTCCTTTCCTCCGCCGAAAGATGCTTAGCCAGAAGCGACGAAGGAGGGGAGCTGGGGAAGGCCGACGATGGCAA 2960
cDNA(MLOC_76215)     AGTCGCCTCCTTTCCTCCGCCGAAAGATGCTTAGCCAGAAGCGACGAAGGAGGGGAGCTGGGGAAGGCCGACGATGGCAA 2960
CircularRNA          -------------------------------------------------------------------------------- 396
Cir_Forward.Primer   -------------------------------------------------------------------------------- 22
Cir_Reverse.Primer   -------------------------------------------------------------------------------- 26

                          2.980               3.000               3.020               3.040
Genomic(MLOC_76215)  TGAGGGGGAAGCTGTCGTAGAAGCTTTGCCCCTTGCTTTACATAAATTGTTATGAACTTACTAAATGACCTTATTTATTC 3040
cDNA(MLOC_76215)     TGAGGGGGAAGCTGTCGTAGAAGCTTTGCCCCTTGCTTTACATAAATTGTTATGAACTTACTAAATGACCTTATTTATTC 3040
CircularRNA          -------------------------------------------------------------------------------- 396
Cir_Forward.Primer   -------------------------------------------------------------------------------- 22
Cir_Reverse.Primer   -------------------------------------------------------------------------------- 26

                          3.060               3.080               3.100               3.120
Genomic(MLOC_76215)  TCCCGGAACGCTAGCAAATCTAATAGTTCCATCTGCTCTTCTTAACTTAAGAACGAAGGCCGCCATCCTTCTTATTCAGG 3120
cDNA(MLOC_76215)     TCCCGGAACGCTAGCAAATCTAATAGTTCCATCTGCTCTTCTTAACTTAAGAACGAAGGCCGCCATCCTTCTTATTCAGG 3120
CircularRNA          -------------------------------------------------------------------------------- 396
Cir_Forward.Primer   -------------------------------------------------------------------------------- 22
Cir_Reverse.Primer   -------------------------------------------------------------------------------- 26

                          3.140               3.160               3.180               3.200
Genomic(MLOC_76215)  AACCCTTTGTTTGAGGAGGGCGTATCCCCGGGAAGGGGAGAGTGGCCGAGCGGTCAAAAGCGACAGACTGTAAATCTGTT 3200
cDNA(MLOC_76215)     AACCCTTTGTTTGAGGAGGGCGTATCCCCGGGAAGGGGAGAGTGGCCGAGCGGTCAAAAGCGACAGACTGTAAATCTGTT 3200
CircularRNA          -------------------------------------------------------------------------------- 396
Cir_Forward.Primer   -------------------------------------------------------------------------------- 22
Cir_Reverse.Primer   -------------------------------------------------------------------------------- 26

                          3.220               3.240               3.260               3.280
Genomic(MLOC_76215)  GAAGGTTTTCTACGTAGGTTCGAATCCTGCCTCTCCCACTTGTTGTAGACTTAAGAGAAGAGAAAGTAGGCGGAAGCCTA 3280
cDNA(MLOC_76215)     GAAGGTTTTCTACGTAGGTTCGAATCCTGCCTCTCCCACTTGTTGTAGACTTAAGAGAAGAGAAAGTAGGCGGAAGCCTA 3280
CircularRNA          -------------------------------------------------------------------------------- 396
Cir_Forward.Primer   -------------------------------------------------------------------------------- 22
Cir_Reverse.Primer   -------------------------------------------------------------------------------- 26

                          3.300               3.320               3.340               3.360
Genomic(MLOC_76215)  GGAGGGCCAACCGAGCGAAGCTCTTTTTTTTTGCCGTGCCGTGAAGTGCAATTTTATCGTATGCGTTTTAGAGAGGTTGT 3360
cDNA(MLOC_76215)     GGAGGGCCAACCGAGCGAAGCTCTTTTTTTTTGCCGTGCCGTGAAGTGCAATTTTATCGTATGCGTTTTAGAGAGGTTGT 3360
CircularRNA          -------------------------------------------------------------------------------- 396
Cir_Forward.Primer   -------------------------------------------------------------------------------- 22
Cir_Reverse.Primer   -------------------------------------------------------------------------------- 26
```

```
                                       3.380               3.400               3.420               3.440
Genomic(MLOC_76215)  CGAAAAAAGACGATCTATAGAGATTCCCCATCTATATCCAATCGAGAATAGAAGCGAGTCGCTTCCGGCGTCGGCTTGTA 3440
cDNA(MLOC_76215)     CGAAAAAAGACGATCTATAGAGATTCCCCATCTATATCCAATCGAGAATAGAAGCGAGTCGCTTCCGGCGTCGGCTTGTA 3440
CircularRNA          -------------------------------------------------------------------------------- 396
Cir_Forward.Primer   -------------------------------------------------------------------------------- 22
Cir_Reverse.Primer   -------------------------------------------------------------------------------- 26

                                       3.460               3.480               3.500               3.520
Genomic(MLOC_76215)  GTCTCTGCAGTCGGCACACGCACGCGCCCGCCATCATGCCTCCTTGGTTCGGGACGAAGCCAAGCGAGACATACGATAGA 3520
cDNA(MLOC_76215)     GTCTCTGCAGTCGGCACACGCACGCGCCCGCCATCATGCCTCCTTGGTTCGGGACGAAGCCAAGCGAGACATACGATAGA 3520
CircularRNA          -------------------------------------------------------------------------------- 396
Cir_Forward.Primer   -------------------------------------------------------------------------------- 22
Cir_Reverse.Primer   -------------------------------------------------------------------------------- 26

                                       3.540               3.560               3.580               3.600
Genomic(MLOC_76215)  CGAAGGAAGCGCCCACCCAGCAGGAGGGCTAAAACCTGTAGTTTTGAAGTTGCAAACTCCAGCTTCGAAGCTGGAGTGCT 3600
cDNA(MLOC_76215)     CGAAGGAAGCGCCCACCCAGCAGGAGGGCTAAAACCTGTAGTTTTGAAGTTGCAAACTCCAGCTTCGAAGCTGGAGTGCT 3600
CircularRNA          -------------------------------------------------------------------------------- 396
Cir_Forward.Primer   -------------------------------------------------------------------------------- 22
Cir_Reverse.Primer   -------------------------------------------------------------------------------- 26

                                       3.620               3.640               3.660               3.680
Genomic(MLOC_76215)  TTAGCCCTTTTTTAAGCAAGAATCACCGTCTTGAGCGCCTTGCGCGCTCAAGAACAAGCAAGGGATGAGCGCTTTGTTGC 3680
cDNA(MLOC_76215)     TTAGCCCTTTTTTAAGCAAGAATCACCGTCTTGAGCGCCTTGCGCGCTCAAGAACAAGCAAGGGATGAGCGCTTTGTTGC 3680
CircularRNA          -------------------------------------------------------------------------------- 396
Cir_Forward.Primer   -------------------------------------------------------------------------------- 22
Cir_Reverse.Primer   -------------------------------------------------------------------------------- 26

                                       3.700               3.720               3.740               3.760
Genomic(MLOC_76215)  TAAAGCGGATACGTTTTCTTGCTGGGGAAGGCTAGTTTGATCGAGGATTGGAGAGGAGGGAGAGGTGGAATAAAAAGCTC 3760
cDNA(MLOC_76215)     TAAAGCGGATACGTTTTCTTGCTGGGGAAGGCTAGTTTGATCGAGGATTGGAGAGGAGGGAGAGGTGGAATAAAAAGCTC 3760
CircularRNA          -------------------------------------------------------------------------------- 396
Cir_Forward.Primer   -------------------------------------------------------------------------------- 22
Cir_Reverse.Primer   -------------------------------------------------------------------------------- 26

                                       3.780               3.800               3.820               3.840
Genomic(MLOC_76215)  GGGATGGATTTTGGAGTCTTTGTGCGAGCCGTATGCGGTGAGAGTCGCACGTACGGTAAGGAGGGGGGTTCGCGTCTATA 3840
cDNA(MLOC_76215)     GGGATGGATTTTGGAGTCTTTGTGCGAGCCGTATGCGGTGAGAGTCGCACGTACGGTAAGGAGGGGGGTTCGCGTCTATA 3840
CircularRNA          -------------------------------------------------------------------------------- 396
Cir_Forward.Primer   -------------------------------------------------------------------------------- 22
Cir_Reverse.Primer   -------------------------------------------------------------------------------- 26

                                       3.860               3.880               3.900               3.920
Genomic(MLOC_76215)  CGTGTAGTGTGGTGGTTGGGCCTACCCACCCTATTTGTTCCATGATCTATGGGTCTACTGGAGCTACCCACTTCGATCAA 3920
cDNA(MLOC_76215)     CGTGTAGTGTGGTGGTTGGGCCTACCCACCCTATTTGTTCCATGATCTATGGGTCTACTGGAGCTACCCACTTCGATCAA 3920
CircularRNA          -------------------------------------------------------------------------------- 396
Cir_Forward.Primer   -------------------------------------------------------------------------------- 22
Cir_Reverse.Primer   -------------------------------------------------------------------------------- 26

                                       3.940               3.960               3.980               4.000
Genomic(MLOC_76215)  TTAGCCAAGATTTTGACCGGATACGAAATCACTGGTGCTCGATCTAGTGGTATTTTTATGGGGATTCTTTTTATCGCTGT 4000
cDNA(MLOC_76215)     TTAGCCAAGATTTTGACCGGATACGAAATCACTG---------------------------------------------- 3954
CircularRNA          -------------------------------------------------------------------------------- 396
Cir_Forward.Primer   -------------------------------------------------------------------------------- 22
Cir_Reverse.Primer   -------------------------------------------------------------------------------- 26

                                       4.020               4.040               4.060               4.080
Genomic(MLOC_76215)  AGGATCCCTATTCAAGATTACTGCAGTTCCTTTTCGGGCGGCTGTAGGACGGACGGCCCCCTATAGGTAGTAGGGTAGGG 4080
cDNA(MLOC_76215)     -------------------------------------------------------------------------------- 3954
CircularRNA          -------------------------------------------------------------------------------- 396
Cir_Forward.Primer   -------------------------------------------------------------------------------- 22
Cir_Reverse.Primer   -------------------------------------------------------------------------------- 26

                                       4.100               4.120               4.140               4.160
Genomic(MLOC_76215)  TGGGTGGTACCGCTCAGATAGCGGCCAATCCTCCTAACTGCGCGCGGGCCGGGCTTAGAGCGCGTGAAACTCATCACTAC 4160
cDNA(MLOC_76215)     -------------------------------------------------------------------------------- 3954
CircularRNA          -------------------------------------------------------------------------------- 396
Cir_Forward.Primer   -------------------------------------------------------------------------------- 22
Cir_Reverse.Primer   -------------------------------------------------------------------------------- 26

                                       4.180               4.200               4.220               4.240
Genomic(MLOC_76215)  CTCGTAAGGGCGTTGAGACCATAGCATGTTACACAAAAGCGCCGCTTTCTCAGGAGTGTTGTCACACAGCTGCCCGACTA 4240
cDNA(MLOC_76215)     ---------------------------------------------------------------------CTGCCCGACTA 3965
CircularRNA          -------------------------------------------------------------------------------- 396
Cir_Forward.Primer   -------------------------------------------------------------------------------- 22
Cir_Reverse.Primer   -------------------------------------------------------------------------------- 26

                                       4.260               4.280               4.300               4.320
Genomic(MLOC_76215)  GAAGAGCTACTCGCTCTGTAGTGTTGTCACACAAGATAAGCACGCCGCCCGCCTGCTGGCCGGGCGAATCGAAGTTATCT 4320
cDNA(MLOC_76215)     GAAGAGCTACTCGCTCTGTAGTGTTGTCACACAAGATAAGCACGCCGCCCGCCTGCTGGCCGGGCGAATCGAAGTTATCT 4045
CircularRNA          -------------------------------------------------------------------------------- 396
Cir_Forward.Primer   -------------------------------------------------------------------------------- 22
Cir_Reverse.Primer   -------------------------------------------------------------------------------- 26

                                       4.340               4.360               4.380               4.400
Genomic(MLOC_76215)  TCCGGTCAACTGTCCACCCAGTCAAGTGCAAAAAACACAGTGGAATCACGCAACGCACGCTGCTGGTGTGCTTCCTGCCC 4400
cDNA(MLOC_76215)     TCCGGTCAACTGTCCACCCAGTCAAGTGCAAAAAACACAGTGGAATCACGCAACGCACGCTGCTGGTGTGCTTCCTGCCC 4125
CircularRNA          -------------------------------------------------------------------------------- 396
Cir_Forward.Primer   -------------------------------------------------------------------------------- 22
Cir_Reverse.Primer   -------------------------------------------------------------------------------- 26

                                       4.420               4.440               4.460               4.480
Genomic(MLOC_76215)  ACGAGGAAAGAAGAGCGACAAGGTTCAGATTTGACTGTTTGCAGCATGGGAGCTGATTACCCAAAAAATCCCTGGGAAAA 4480
cDNA(MLOC_76215)     ACGAGGAAAGAAGAGCGACAAGGTTCAGATTTGACTGTTTGCAGCATGGGAGCTGATTACCCAAAAAATCCCTGGGAAAA 4205
CircularRNA          -------------------------------------------------------------------------------- 396
Cir_Forward.Primer   -------------------------------------------------------------------------------- 22
Cir_Reverse.Primer   -------------------------------------------------------------------------------- 26
```

```
                                    4.500               4.520               4.540               4.560
Genomic(MLOC_76215) AAGAAAAGATATCTCGGTAACGAAAACCATAGGAGGCTGTATTGGCGAGATCCAAGGGTTCACAGCAGCCCAAAAGAAAA 4560
   cDNA(MLOC_76215) AAGAAAAGATATCTCGGTAACGAAAACCATAGGAGGCTGTATTGGCGAGATCCAAGGGTTCACAGCAGCCCAAAAGAAAA 4285
        CircularRNA -------------------------------------------------------------------------------- 396
  Cir_Forward.Primer -------------------------------------------------------------------------------- 22
  Cir_Reverse.Primer -------------------------------------------------------------------------------- 26
                                    4.580               4.600               4.620               4.640
Genomic(MLOC_76215) ACCGCCTGGAAGTCCGAGGACCTTTAGTACCGTACCGAACCAGCAGCCTTCGCGCCAAGCGACGACCGCCCTTGTCCCTT 4640
   cDNA(MLOC_76215) ACCGCCTGGAAGTCCGAGGACCTTTAGTACCGTACCGAACCAGCAGCCTTCGCGCCAAGCGACGACCGCCCTTGTCCCTT 4365
        CircularRNA -------------------------------------------------------------------------------- 396
  Cir_Forward.Primer -------------------------------------------------------------------------------- 22
  Cir_Reverse.Primer -------------------------------------------------------------------------------- 26
                                    4.660               4.680               4.700               4.720
Genomic(MLOC_76215) TCCTTTTTCCATTCAGCCTACTTCTTAGCTTTGTTCCGTCAGTCTAAGGCAAAGCTTAAGTGGTTTGCCTACCTTACCCA 4720
   cDNA(MLOC_76215) TCCTTTTTCCATTCAGCCTACTTCTTAGCTTTGTTCCGTCAGTCTAAGGCAAAGCTTAAGTGGTTTGCCTACCTTACCCA 4445
        CircularRNA -------------------------------------------------------------------------------- 396
  Cir_Forward.Primer -------------------------------------------------------------------------------- 22
  Cir_Reverse.Primer -------------------------------------------------------------------------------- 26
                                    4.740               4.760               4.780               4.800
Genomic(MLOC_76215) CTTGATGAAAGGGAACGAACTTCGTTTCCTTGGCGGCTTTATGGATGGATTCAGTCAGAAAAAACTCCTTCCTTTTGAAA 4800
   cDNA(MLOC_76215) CTTGATGAAAGGGAACGAACTTCGTTTCCTTGGCGGCTTTATGGATGGATTCAGTCAGAAAAAACTCCTTCCTTTTGAAA 4525
        CircularRNA -------------------------------------------------------------------------------- 396
  Cir_Forward.Primer -------------------------------------------------------------------------------- 22
  Cir_Reverse.Primer -------------------------------------------------------------------------------- 26
                                    4.820               4.840               4.860               4.880
Genomic(MLOC_76215) AAAGTGACGCTTTGCGTCTTCGCTTCCGAGGGTTAGGGTATAGGCCGTTTCGAGCAAGCTTTCGCTTTTTCTCCCGGCTT 4880
   cDNA(MLOC_76215) AAAGTGACGCTTTGCGTCTTCGCTTCCGAGGGTTAGGGTATAGGCCGTTTCGAGCAAGCTTTCGCTTTTTCTCCCGGCTT 4605
        CircularRNA -------------------------------------------------------------------------------- 396
  Cir_Forward.Primer -------------------------------------------------------------------------------- 22
  Cir_Reverse.Primer -------------------------------------------------------------------------------- 26
                                    4.900               4.920               4.940               4.960
Genomic(MLOC_76215) TCTACAACTTTCGCTTTAGCTTCCAAAGGCGACCCAATGACCTTTCCGGAATTGGAAGTATTCGTCGCCCTACCCTAAGA 4960
   cDNA(MLOC_76215) TCTACAACTTTCGCTTTAGCTTCCAAAGGCGACCCAATGACCTTTCCGGAATTGGAAGTATTCGTCGCCCTACCCTAAGA 4685
        CircularRNA -------------------------------------------------------------------------------- 396
  Cir_Forward.Primer -------------------------------------------------------------------------------- 22
  Cir_Reverse.Primer -------------------------------------------------------------------------------- 26
                                    4.980               5.000               5.020               5.040
Genomic(MLOC_76215) AAGAAGTCACTATAAAACTGCTTGCCCTCCAGAAGTACCAAAGGTGCGCGGAGCCCGGTGAAAATGAATATGTTTGCTAC 5040
   cDNA(MLOC_76215) AAGAAGTCACTATAAAACTGCTTGCCCTCCAGAAGTACCAAAGGTGCGCGGAGCCCGGTGAAAATGAATATGTTTGCTAC 4765
        CircularRNA -------------------------------------------------------------------------------- 396
  Cir_Forward.Primer -------------------------------------------------------------------------------- 22
  Cir_Reverse.Primer -------------------------------------------------------------------------------- 26
                                    5.060               5.080               5.100               5.120
Genomic(MLOC_76215) TGAAAGAAGCCTGCCTATTGACTAATATTCGTCTTTAGAATGAAAGTAGCTATGAAGCCCAATCTACTGTCGATTCAAAA 5120
   cDNA(MLOC_76215) TGAAAGAAGCCTGCCTATTGACTAATATTCGTCTTTAGAATGAAAGTAGCTATGAAGCCCAATCTACTGTCGATTCAAAA 4845
        CircularRNA -------------------------------------------------------------------------------- 396
  Cir_Forward.Primer -------------------------------------------------------------------------------- 22
  Cir_Reverse.Primer -------------------------------------------------------------------------------- 26
                                    5.140               5.160               5.180               5.200
Genomic(MLOC_76215) GGGGGCATAGTCATATGGGTGGGGTGTTTGTGGGGAGCTGCCTTGGATATGAGGAATTCCTCAAATCTAAAAAAAAAAGA 5200
   cDNA(MLOC_76215) GGGGGCATAGTCATATGGGTGGGGTGTTTGTGGGGAGCTGCCTTGGATATGAGGAATTCCTCAAATCTAAAAAAAAAAGA 4925
        CircularRNA -------------------------------------------------------------------------------- 396
  Cir_Forward.Primer -------------------------------------------------------------------------------- 22
  Cir_Reverse.Primer -------------------------------------------------------------------------------- 26
                                    5.220               5.240               5.260               5.280
Genomic(MLOC_76215) ACAAAGACAAAGTCCGTGACGAAGATCTTTCTTTTCTATCAATAGATAGGAATGAGTGTTCGTTATAGGGGATAGGATCC 5280
   cDNA(MLOC_76215) ACAAAGACAAAGTCCGTGACGAAGATCTTTCTTTTCTATCAATAGATAGGAATGAGTGTTCGTTATAGGGGATAGGATCC 5005
        CircularRNA -------------------------------------------------------------------------------- 396
  Cir_Forward.Primer -------------------------------------------------------------------------------- 22
  Cir_Reverse.Primer -------------------------------------------------------------------------------- 26
                                    5.300               5.320               5.340               5.360
Genomic(MLOC_76215) ATCTGCTCTCTCTCTCTCTCTATTTTCTTTGATGCAATTTAGAGAGAAAGAGCAGTGCGAATTAGAAAAAAAAGAGAATC 5360
   cDNA(MLOC_76215) ATCTGCTCTCTCTCTCTCTCTATTTTCTTTGATGCAATTTAGAGAGAAAGAGCAGTGCGAATTAGAAAAAAAAGAGAATC 5085
        CircularRNA -------------------------------------------------------------------------------- 396
  Cir_Forward.Primer -------------------------------------------------------------------------------- 22
  Cir_Reverse.Primer -------------------------------------------------------------------------------- 26
                                    5.380               5.400               5.420               5.440
Genomic(MLOC_76215) GGGAGATGATTCCAAAATAGATACATAGACATCTAAAGTAAAGGGATGTATATATTATTCCTATATATATCATCTATCTA 5440
   cDNA(MLOC_76215) GGGAGATGATTCCAAAATAGATACATAGACATCTAAAGTAAAGGGATGTATATATTATTCCTATATATATCATCTATCTA 5165
        CircularRNA -------------------------------------------------------------------------------- 396
  Cir_Forward.Primer -------------------------------------------------------------------------------- 22
  Cir_Reverse.Primer -------------------------------------------------------------------------------- 26
                                    5.460               5.480               5.500               5.520
Genomic(MLOC_76215) TGAAAAAAGTAAACAGAGTTCGTTTTTGGGTTCCCCGAAGCCCCGAATGGATTGGATCGTTTCTGCTTCTGCCAACGAAA 5520
   cDNA(MLOC_76215) TGAAAAAAGTAAACAGAGTTCGTTTTTGGGTTCCCCGAAGCCCCGAATGGATTGGATCGTTTCTGCTTCTGCCAACGAAA 5245
        CircularRNA -------------------------------------------------------------------------------- 396
  Cir_Forward.Primer -------------------------------------------------------------------------------- 22
  Cir_Reverse.Primer -------------------------------------------------------------------------------- 26
                                    5.540               5.560               5.580               5.600
Genomic(MLOC_76215) TGAAGGCCTCGCCCCTTCCGAATGCTTCTCCGATCCTGAAGTTCTCGCGAAGAGAATAAGATGCAGCTCCCCCTCCCTCT 5600
   cDNA(MLOC_76215) TGAAGGCCTCGCCCCTTCCGAATGCTTCTCCGATCCTGAAGTTCTCGCGAAGAGAATAAGATGCAGCTCCCCCTCCCTCT 5325
        CircularRNA -------------------------------------------------------------------------------- 396
  Cir_Forward.Primer -------------------------------------------------------------------------------- 22
  Cir_Reverse.Primer -------------------------------------------------------------------------------- 26
```

```
                                    5.620               5.640               5.660               5.680
Genomic(MLOC_76215)  GTCTTTTTCCGCTTTGCTAATCTTCCCCTCTAACGCGGGCCGGGCTTAGGCGGGCGCGGGAGGAAGAAAGAAAGTCGAAG 5680
cDNA(MLOC_76215)     GTCTTTTTCCGCTTTGCTAATCTTCCCCTCTAACGCGGGCCGGGCTTAGGCGGGCGCGGGAGGAAGAAAGAAAGTCGAAG 5405
CircularRNA          -------------------------------------------------------------------------------- 396
Cir_Forward.Primer   -------------------------------------------------------------------------------- 22
Cir_Reverse.Primer   -------------------------------------------------------------------------------- 26

                                    5.700               5.720               5.740               5.760
Genomic(MLOC_76215)  AGCGTCTTCTCCTTTGTCCTTTTTTCAGTGCAACACAGGAAAGCACCCTCTTTTTGTAATCCCTGCAGCTTCCCAGAGGC 5760
cDNA(MLOC_76215)     AGCGTCTTCTCCTTTGTCCTTTTTTCAGTGCAACACAGGAAAGCACCCTCTTTTTGTAATCCCTGCAGCTTCCCAGAGGC 5485
CircularRNA          -------------------------------------------------------------------------------- 396
Cir_Forward.Primer   -------------------------------------------------------------------------------- 22
Cir_Reverse.Primer   -------------------------------------------------------------------------------- 26

                                    5.780               5.800               5.820               5.840
Genomic(MLOC_76215)  TTGCTTTTTTTTTATTGAACGCATGGCGTAGCTAGGACCCCTCCAATCATGTTTGAGCCTATGTTCACCCGGGGCCAAAA 5840
cDNA(MLOC_76215)     TTGCTTTTTTTTTATTGAACGCATGGCGTAGCTAGGACCCCTCCAATCATGTTTGAGCCTATGTTCACCCGGGGCCAAAA 5565
CircularRNA          -------------------------------------------------------------------------------- 396
Cir_Forward.Primer   -------------------------------------------------------------------------------- 22
Cir_Reverse.Primer   -------------------------------------------------------------------------------- 26

                                    5.860               5.880               5.900               5.920
Genomic(MLOC_76215)  AAGGAGAATTCCGGAATGCCTGCCGACGTTCAGATAAGGTGCATATCCCTTACCACTAAAGGAAAGGCTCGACGAAGGGG 5920
cDNA(MLOC_76215)     AAGGAGAATTCCGGAATGCCTGCCGACGTTCAGATAAGGTGCATATCCCTTACCACTAAAGGAAAGGCTCGACGAAGGGG 5645
CircularRNA          -------------------------------------------------------------------------------- 396
Cir_Forward.Primer   -------------------------------------------------------------------------------- 22
Cir_Reverse.Primer   -------------------------------------------------------------------------------- 26

                                    5.940               5.960               5.980               6.000
Genomic(MLOC_76215)  GGGATATTAGCTGGGTAAGGAAAACGCTTTCGGAGATTGAGATGTCGATTTGTTTTTCATCGAAAAGGAAGAAGGCCGAG 6000
cDNA(MLOC_76215)     GGGATATTAGCTGGGTAAGGAAAACGCTTTCGGAGATTGAGATGTCGATTTGTTTTTCATCGAAAAGGAAGAAGGCCGAG 5725
CircularRNA          -------------------------------------------------------------------------------- 396
Cir_Forward.Primer   -------------------------------------------------------------------------------- 22
Cir_Reverse.Primer   -------------------------------------------------------------------------------- 26

                                    6.020               6.040               6.060               6.080
Genomic(MLOC_76215)  GGGATAGCAGCCTTCCTTCGGGCTTTGCCTGCCGACGTTCTAATAAAGAATTCCGGCTCGGCCCGGGAAAGCGCTGGCAA 6080
cDNA(MLOC_76215)     GGGATAGCAGCCTTCCTTCGGGCTTTGCCTGCCGACGTTCTAATAAAGAATTCCGGCTCGGCCCGGGAAAGCGCTGGCAA 5805
CircularRNA          -------------------------------------------------------------------------------- 396
Cir_Forward.Primer   -------------------------------------------------------------------------------- 22
Cir_Reverse.Primer   -------------------------------------------------------------------------------- 26

                                    6.100               6.120               6.140
Genomic(MLOC_76215)  CAACATAAAGAAAGGGGTCCATGTAGCTGCTGCGCCCGCCCACTGAGCAGCAGGTTCGGCATCTACTACAAAAG 6154
cDNA(MLOC_76215)     CAACATAAAGAAAGGGGTCCATGTAGCTGCTGCGCCCGCCCACTGAGCAGCAGGTTCGGCATCTACTACAAAAG 5879
CircularRNA          -------------------------------------------------------------------------- 396
Cir_Forward.Primer   -------------------------------------------------------------------------- 22
Cir_Reverse.Primer   -------------------------------------------------------------------------- 26
```

Real-Time PCR for the junction region of NADH dehydrogenase

Nad9_circular RNA (ID: Morex_contig_70567:2809-3204**)**

By divergent primers ◀▶ on genomic DNA

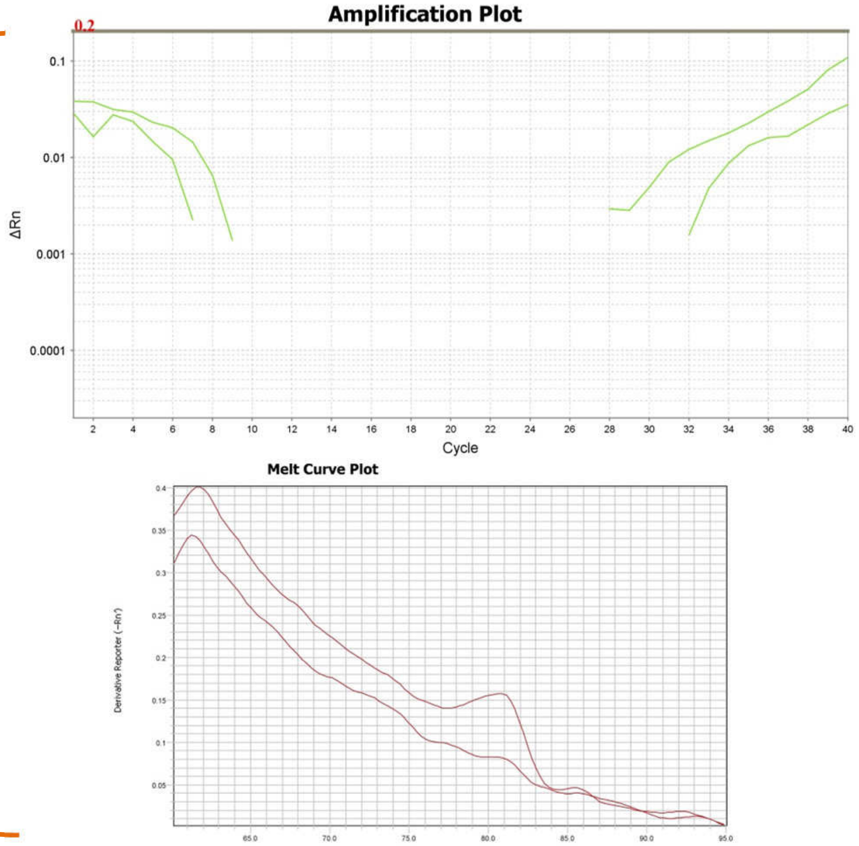

By divergent primers ◀▶ on cDNAs

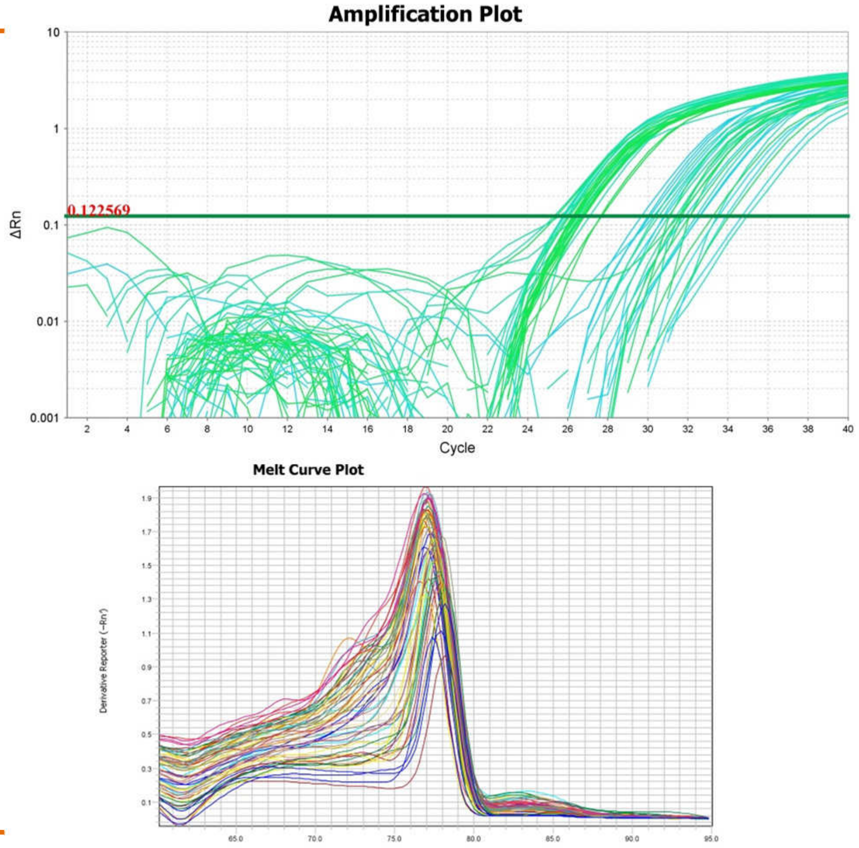

Real-Time PCR for the junction region of NADH dehydrogenase

Nad9_circular RNA (ID: Morex_contig_70567:2809-3204**)**

By divergent primers ◀▶ & with no template

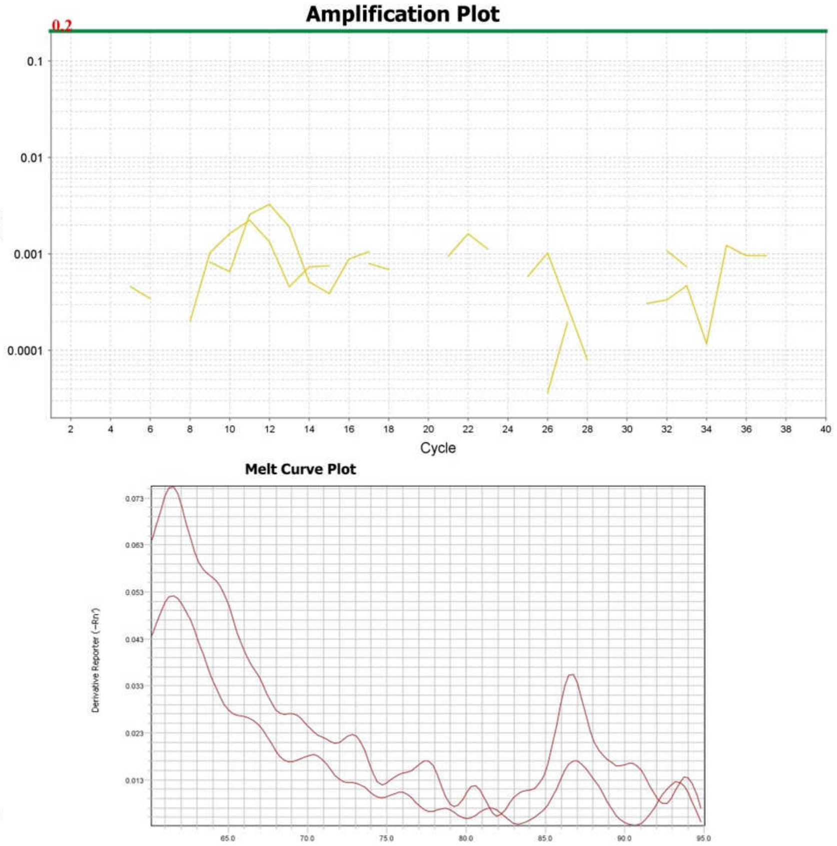

Real-Time PCR for NADH dehydrogenase Nad9

By convergent primers ▶◀ on cDNAs

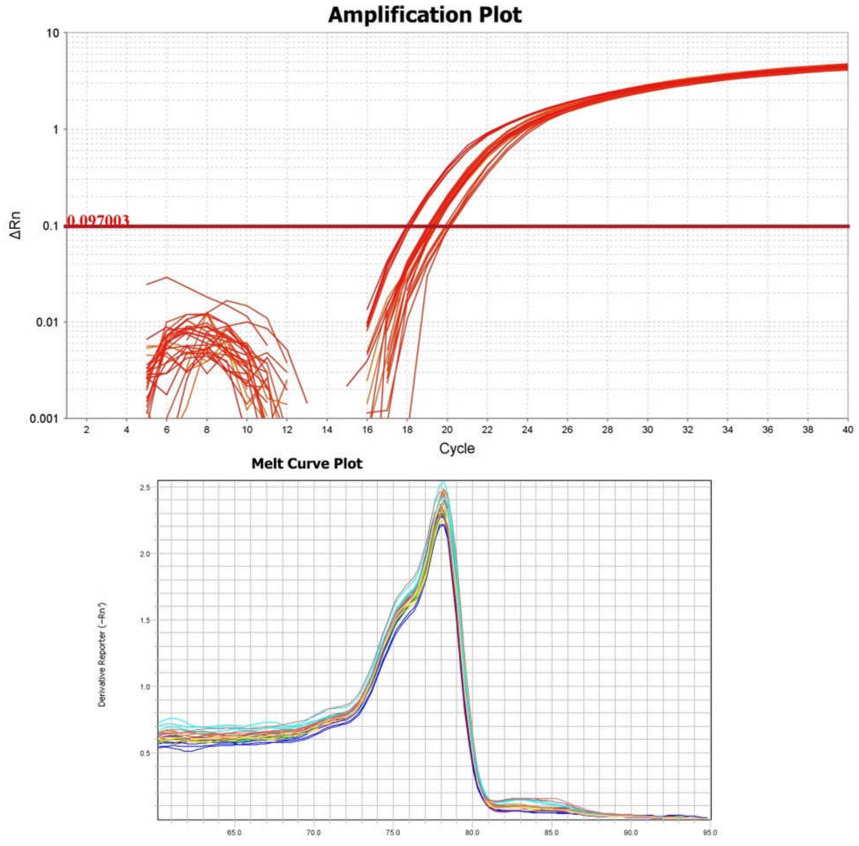

By convergent primers ▶◀ & with no template

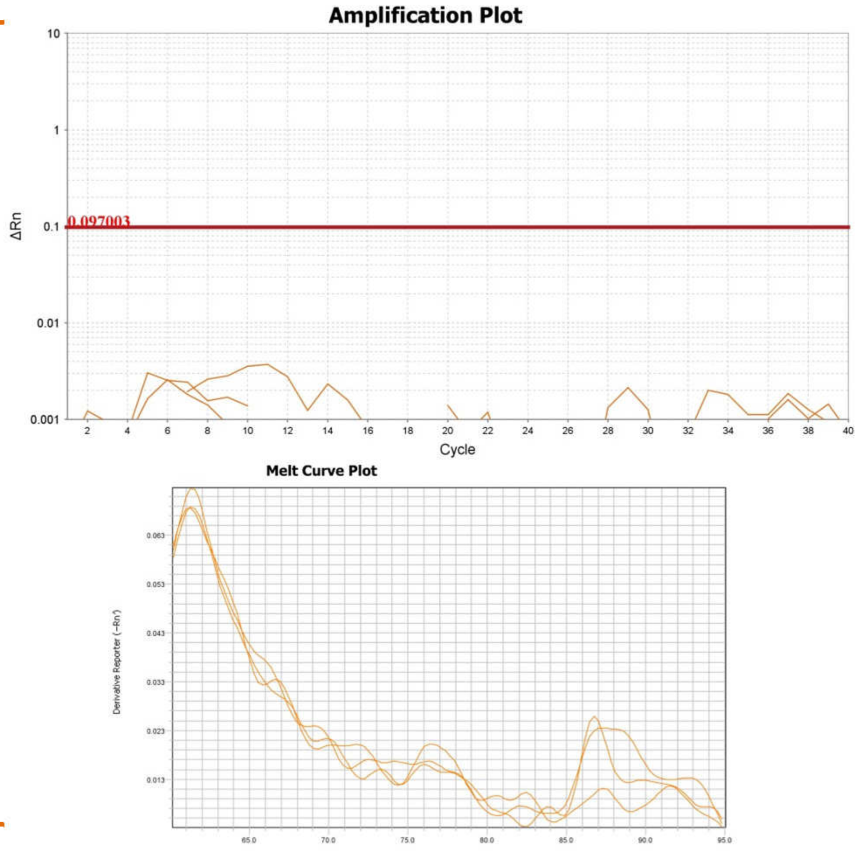

Mit. Apocytochrome b_circular RNA (ID: Morex_contig_42365:12844-13477**)**

<u>C</u>TTCATATTTTTCGTGGTCTATATCATGCGAGTTATAGCAGTCCTAGGGAATTTGTTCGGTGTCT
CGGAGTTGTCATATTCCTATTAATGATTGTGACAGCTTTTATAGGATACGTACCACCTTGGGGTCA
GATGAGCTTTTGGGGAGCAACAGTAATTACAAGCTTAGCTAGCGCCATACCAGTAGTAGGAGATA
CCATAGTGACTTGGCTTTGGGGTGGTTTCTCCGTGGACAATGCCACCTTAAATCGTTTTTTTAGTC
TCCATCATTTACTCCCCCTTATTTTAGTAGGCGCCAGTCTTCTTCATCTGGCCGCATTGCATCAATA
TGGATCAAATAATCCATTGGGTGTACATTCTGAGATGGATAAAATTGCTTCTTACCCTTATTTTTAT
GTAAAGGATCTTGTAGGTCGGGTAGCTTCTGCTATCTTTTTTTCCATTTGGATTTTTTTTGCTCCTA
ATGTTTTGGGGCATCCCGACAATTATATACCTGCTAATCCGATGCCCACCCCGCCTCATATTGTGC
CGGAATGGTATTTCCTACCGATCCATGCCATTCTTCGCAGTATACCTGACAAAGCGGGAGGTGTA
GC**CAGCGCAATAGCACTAGTTTTTATATCTCTCTTGGCTTTA<u>C</u>**

The nucleotides of junction-region are underlined. The nucleotides of junction-region which are supported by the junction-spaning sequencing reads are shown in red. Introns are not shown if the absence is supported by sequencing reads. In the absence of supporting sequencing reads, the intronic nucletides are shown as N.

Structural relationship between the circular RNA and its parental gene

```
                                     20                  40                  60                  80
Genomic(MLOC_58118)  CTTGGTATTCACAATCAAAGGTCTCCCCAACCATTATAACAAGAACCAACCGTTGGATAGGGCCACCGCTCGATGGGTTG  80
cDNA(MLOC_58118)     CTTGGTATTCACAATCAAAGGTCTCCCCAACCATTATAACAAGAACCAACCGTTGGATAGGGCCACCGCTCGATGGGTTG  80
CircularRNA          --------------------------------------------------------------------------------  -
Cir_Forward.Primer   --------------------------------------------------------------------------------  -
Cir_Reverse.Primer   --------------------------------------------------------------------------------  -

                                    100                 120                 140                 160
Genomic(MLOC_58118)  GATCCTGCTCACCACGCGGAAAACAACCATACAATTTAGTAATTCCGGGGCTTACTATTCAAGGGCAAATCAAGTAGAAT  160
cDNA(MLOC_58118)     GATCCTGCTCACCACGCGGAAAACAACCATACAATTTAGTAATTCCGGGGCTTACTATTCAAGGGCAAATCAAGTAGAAT  160
CircularRNA          --------------------------------------------------------------------------------  -
Cir_Forward.Primer   --------------------------------------------------------------------------------  -
Cir_Reverse.Primer   --------------------------------------------------------------------------------  -

                                    180                 200                 220                 240
Genomic(MLOC_58118)  TGTGTATATAGATGAAAAAGGAGAATGGCGAAGAAAGTCAATTTGTCCAAAGCACGGTGCCGACCTCCTTCTTTTATACT  240
cDNA(MLOC_58118)     TGTGTATATAGATGAAAAAGGAGAATGGCGAAGAAAGTCAATTTGTCCAAAGCACGGTGCCGACCTCCTTCTTTTATACT  240
CircularRNA          --------------------------------------------------------------------------------  -
Cir_Forward.Primer   --------------------------------------------------------------------------------  -
Cir_Reverse.Primer   --------------------------------------------------------------------------------  -

                                    260                 280                 300                 320
Genomic(MLOC_58118)  ATACGAACGAGAATTCTCATTTCTTGTGAATGGGCCCAATTTCCCTTTTGTTACTGGTCGGGACGACCTAAATCCTATAT  320
cDNA(MLOC_58118)     ATACGAACGAGAATTCTCATTTCTTGTGAATGGGCCCAATTTCCCTTTTGTTACTGGTCGGGACGACCTAAATCCTATAT  320
CircularRNA          --------------------------------------------------------------------------------  -
Cir_Forward.Primer   --------------------------------------------------------------------------------  -
Cir_Reverse.Primer   --------------------------------------------------------------------------------  -

                                    340                 360                 380                 400
Genomic(MLOC_58118)  CGGTTTCTCAAGTGCCAAATAGAATAGTTCTTTTCCAAGTTTGGGTAGGATTCCCGACACCAATCACATCTGTAAGTAGC  400
cDNA(MLOC_58118)     CGGTTTCTCAAGTGCCAAATAGAATAGTTCTTTTCCAAGTTTGGGTAGGATTCCCGACACCAATCACATCTGTAAGTAGC  400
CircularRNA          --------------------------------------------------------------------------------  -
Cir_Forward.Primer   --------------------------------------------------------------------------------  -
Cir_Reverse.Primer   --------------------------------------------------------------------------------  -

                                    420                 440                 460                 480
Genomic(MLOC_58118)  AAAGATTTAAACAAATAATGAGAGGCCTTTATCTAATTAAGTTGAATAATACACAGATTGTAATAAAGGAGCGTGCTAAC  480
cDNA(MLOC_58118)     AAAGATTTAAACAAATAATGAGAGGCCTTTATCTAATTAAGTTGAATAATACACAGATTGTAATAAAGGAGCGTGCTAAC  480
CircularRNA          --------------------------------------------------------------------------------  -
Cir_Forward.Primer   --------------------------------------------------------------------------------  -
Cir_Reverse.Primer   --------------------------------------------------------------------------------  -

                                    500                 520                 540                 560
Genomic(MLOC_58118)  TGTCACAGATAGCGTGGAGATAAACAGCACGTACCGACCAAGTGTCGCGGTAGGTTGGCTGGTGCAGGCAGGTTTGATAA  560
cDNA(MLOC_58118)     TGTCACAGATAGCGTGGAGATAAACAGCACGTACCGACCAAGTGTCGCGGTAGGTTGGCTGGTGCAGGCAGGTTTGATAA  560
CircularRNA          --------------------------------------------------------------------------------  -
Cir_Forward.Primer   --------------------------------------------------------------------------------  -
Cir_Reverse.Primer   --------------------------------------------------------------------------------  -

                                    580                 600                 620                 640
Genomic(MLOC_58118)  CTCAGTAAGAGAGTATGACCACTTTGGGAAATCCCCTAGCTTTCGTCCCTCACTGTCTACAGCGTAGCCGTCATGGAACC  640
cDNA(MLOC_58118)     CTCAGTAAGAGAGTATGACCACTTTGGGAAATCCCCTAGCTTTCGTCCCTCACTGTCTACAGCGTAGCCGTCATGGAACC  640
CircularRNA          --------------------------------------------------------------------------------  -
Cir_Forward.Primer   --------------------------------------------------------------------------------  -
Cir_Reverse.Primer   --------------------------------------------------------------------------------  -

                                    660                 680                 700                 720
Genomic(MLOC_58118)  CCAGTGTCTTCGTCAAGCATTCCACTCTAGTTTTCGCTACCAGTGTTTCAACAACACACAATACCGCCGGCCCCCATGCC  720
cDNA(MLOC_58118)     CCAGTGTCTTCGTCAAGCATTCCACTCTAGTTTTCGCTACCAGTGTTTCAACAACACACAATACCGCCGGCCCCCATGCC  720
CircularRNA          --------------------------------------------------------------------------------  -
Cir_Forward.Primer   --------------------------------------------------------------------------------  -
Cir_Reverse.Primer   --------------------------------------------------------------------------------  -

                                    740                 760                 780                 800
Genomic(MLOC_58118)  ACACATGTCAAATTATAGAATAGATCTCGAGTAGAGACAAGAAGACAGCACGCTAAAGCGATCGAAATGGTTGTACATGA  800
cDNA(MLOC_58118)     ACACATGTCAAATTATAGAATAGATCTCGAGTAGAGACAAGAAGACAGCACGCTAAAGCGATCGAAATGGTTGTACATGA  800
CircularRNA          --------------------------------------------------------------------------------  -
Cir_Forward.Primer   --------------------------------------------------------------------------------  -
Cir_Reverse.Primer   --------------------------------------------------------------------------------  -

                                    820                 840                 860                 880
Genomic(MLOC_58118)  CTAGTCGTCTCCTGCGGCTCGCGATAACATGAATATCTCTCATTCTCTTCCATTCTTAGGCCTTCATAAAATAAGACAAA  880
cDNA(MLOC_58118)     CTAGTCGTCTCCTGCGGCTCGCGATAACATGAATATCTCTCATTCTCTTCCATTCTTAGGCCTTCATAAAATAAGACAAA  880
CircularRNA          --------------------------------------------------------------------------------  -
Cir_Forward.Primer   --------------------------------------------------------------------------------  -
Cir_Reverse.Primer   --------------------------------------------------------------------------------  -

                                    900                 920                 940                 960
Genomic(MLOC_58118)  TATAAAGCAATCGAGCCAAAGAGAAAATCATGAAGCATCTAAGGTTTCCTTATCTTTTGACAATCTAGCTCCTCCGGATA  960
cDNA(MLOC_58118)     TATAAAGCAATCGAGCCAAAGAGAAAATCATGAAGCATCTAAGGTTTCCTTATCTTTTGACAATCTAGCTCCTCCGGATA  960
CircularRNA          --------------------------------------------------------------------------------  -
Cir_Forward.Primer   --------------------------------------------------------------------------------  -
Cir_Reverse.Primer   --------------------------------------------------------------------------------  -

                                    980               1.000               1.020               1.040
Genomic(MLOC_58118)  CGATCATGCTTAATGTTACTGGCGCATGTTAATGTCTTAATCTCATAATAGACGAAAGAACGATAGGGAGTTGGTATCAA  1040
cDNA(MLOC_58118)     CGATCATGCTTAATGTTACTGGCGCATGTTAATGTCTTAATCTCATAATAGACGAAAGAACGATAGGGAGTTGGTATCAA  1040
CircularRNA          --------------------------------------------------------------------------------  -
Cir_Forward.Primer   --------------------------------------------------------------------------------  -
Cir_Reverse.Primer   --------------------------------------------------------------------------------  -

                                  1.060               1.080               1.100               1.120
Genomic(MLOC_58118)  ATCCATACCGATGATGAATGCATTCCATTCCATCGGTTAAGTTGGTATCCTCAGGAAAAAACAAAGCAGCGGAGTACCCA  1120
cDNA(MLOC_58118)     ATCCATACCGATGATGAATGCATTCCATTCCATCGGTTAAGTTGGTATCCTCAGGAAAAAACAAAGCAGCGGAGTACCCA  1120
CircularRNA          --------------------------------------------------------------------------------  -
Cir_Forward.Primer   --------------------------------------------------------------------------------  -
Cir_Reverse.Primer   --------------------------------------------------------------------------------  -
```

```
                     1.140               1.160               1.180               1.200
Genomic(MLOC_58118)  TTTTTACATGAGAAAGAAAATGAGCTGATTTGAAGCTCTGCTCTTGTTATGTTGAACAAACGCCCTGCGGGGCACGCCCT 1200
cDNA(MLOC_58118)     TTTTTACATGAGAAAGAAAATGAGCTGATTTGAAGCTCTGCTCTTGTTATGTTGAACAAACGCCCTGCGGGGCACGCCCT 1200
CircularRNA          -------------------------------------------------------------------------------- -
Cir_Forward.Primer   -------------------------------------------------------------------------------- -
Cir_Reverse.Primer   -------------------------------------------------------------------------------- -

                     1.220               1.240               1.260               1.280
Genomic(MLOC_58118)  CCGGGGGTTTACGCCAGGAATTCCGTAGAAGAGAGTCTTGGTAAGAAATAAATAAAAAAGGTACAGTCTACCGCAAGTAA 1280
cDNA(MLOC_58118)     CCGGGGGTTTACGCCAGGAATTCCGTAGAAGAGAGTCTTGGTAAGAAATAAATAAAAAAGGTACAGTCTACCGCAAGTAA 1280
CircularRNA          -------------------------------------------------------------------------------- -
Cir_Forward.Primer   -------------------------------------------------------------------------------- -
Cir_Reverse.Primer   -------------------------------------------------------------------------------- -

                     1.300               1.320               1.340               1.360
Genomic(MLOC_58118)  AAAACCCATTGCTTCTATCGCTGACGGGGCCTTTTAGGCTATATAAAATGCCACCATCGTAAAGGAGGATTCGAAGAAGA 1360
cDNA(MLOC_58118)     AAAACCCATTGCTTCTATCGCTGACGGGGCCTTTTAGGCTATATAAAATGCCACCATCGTAAAGGAGGATTCGAAGAAGA 1360
CircularRNA          -------------------------------------------------------------------------------- -
Cir_Forward.Primer   -------------------------------------------------------------------------------- -
Cir_Reverse.Primer   -------------------------------------------------------------------------------- -

                     1.380               1.400               1.420               1.440
Genomic(MLOC_58118)  CGGCATGACCGGGAGAGAGACGCAAGGATGGGACTGGAACGATTGAGAAGCACTCATTTATCACGGATGAATAAAAATAT 1440
cDNA(MLOC_58118)     CGGCATGACCGGGAGAGAGACGCAAGGATGGGACTGGAACGATTGAGAAGCACTCATTTATCACGGATGAATAAAAATAT 1440
CircularRNA          -------------------------------------------------------------------------------- -
Cir_Forward.Primer   -------------------------------------------------------------------------------- -
Cir_Reverse.Primer   -------------------------------------------------------------------------------- -

                     1.460               1.480               1.500               1.520
Genomic(MLOC_58118)  GCGTATAGTAAAGACATACGCCCCATATCTGCGGATGATAGGCTCTAATTGCATGTGACCAGTACGGGAACCGAGTTCAT 1520
cDNA(MLOC_58118)     GCGTATAGTAAAGACATACGCCCCATATCTGCGGATGATAGGCTCTAATTGCATGTGACCAGTACGGGAACCGAGTTCAT 1520
CircularRNA          -------------------------------------------------------------------------------- -
Cir_Forward.Primer   -------------------------------------------------------------------------------- -
Cir_Reverse.Primer   -------------------------------------------------------------------------------- -

                     1.540               1.560               1.580               1.600
Genomic(MLOC_58118)  GTCTTTCCGGGAGTAGAGCTTGCGTTAAACTTGCTTCAATGGCGATGAAAATAGCTCAGTTGGTAGACGATGTCATGTGC 1600
cDNA(MLOC_58118)     GTCTTTCCGGGAGTAGAGCTTGCGTTAAACTTGCTTCAATGGCGATGAAAATAGCTCAGTTGGTAGACGATGTCATGTGC 1600
CircularRNA          -------------------------------------------------------------------------------- -
Cir_Forward.Primer   -------------------------------------------------------------------------------- -
Cir_Reverse.Primer   -------------------------------------------------------------------------------- -

                     1.620               1.640               1.660               1.680
Genomic(MLOC_58118)  CAGCTTGTTTTCTCGTTGAAGCATTCTTAGACTGATAGCTAGGGAAGTCCCATTTTGCAAGGGCTCGCGGAGTAGCCACA 1680
cDNA(MLOC_58118)     CAGCTTGTTTTCTCGTTGAAGCATTCTTAGACTGATAGCTAGGGAAGTCCCATTTTGCAAGGGCTCGCGGAGTAGCCACA 1680
CircularRNA          -------------------------------------------------------------------------------- -
Cir_Forward.Primer   -------------------------------------------------------------------------------- -
Cir_Reverse.Primer   -------------------------------------------------------------------------------- -

                     1.700               1.720               1.740               1.760
Genomic(MLOC_58118)  GATAGAAATTATCAAGTACCAACCAAATAGTTCTTCCAAGGCTAAAGGCAGGCTGACCCGCTAATTATGTCGTAGGTAGG 1760
cDNA(MLOC_58118)     GATAGAAATTATCAAGTACCAACCAAATAGTTCTTCCAAGGCTAAAGGCAGGCTGACCCGCTAATTATGTCGTAGGTAGG 1760
CircularRNA          -------------------------------------------------------------------------------- -
Cir_Forward.Primer   -------------------------------------------------------------------------------- -
Cir_Reverse.Primer   -------------------------------------------------------------------------------- -

                     1.780               1.800               1.820               1.840
Genomic(MLOC_58118)  GATCTCGTTTGAAAAAAATCGTATAACAATGAAGCGAGAAAACGTATGCGCCCGCAAACGGCTTTCGAGGCGATCCTGAC 1840
cDNA(MLOC_58118)     GATCTCGTTTGAAAAAAATCGTATAACAATGAAGCGAGAAAACGTATGCGCCCGCAAACGGCTTTCGAGGCGATCCTGAC 1840
CircularRNA          -------------------------------------------------------------------------------- -
Cir_Forward.Primer   -------------------------------------------------------------------------------- -
Cir_Reverse.Primer   -------------------------------------------------------------------------------- -

                     1.860               1.880               1.900               1.920
Genomic(MLOC_58118)  CAGCAAACGAACTCAAATTCAAGCGACCCTCAAAGCGAGGGGAAGACCCCCATTCTTTTTGTCGAAATCTTACTGTACTT 1920
cDNA(MLOC_58118)     CAGCAAACGAACTCAAATTCAAGCGACCCTCAAAGCGAGGGGAAGACCCCCATTCTTTTTGTCGAAATCTTACTGTACTT 1920
CircularRNA          -------------------------------------------------------------------------------- -
Cir_Forward.Primer   -------------------------------------------------------------------------------- -
Cir_Reverse.Primer   -------------------------------------------------------------------------------- -

                     1.940               1.960               1.980               2.000
Genomic(MLOC_58118)  AAAAAGAACAATGTTGTTACTTCTTCAAATCTCTATTGTGTAGGTGTTCAGTCCCAGGTAGGAATAAGTCAAGGCTGTTT 2000
cDNA(MLOC_58118)     AAAAAGAACAATGTTGTTACTTCTTCAAATCTCTATTGTGTAGGTGTTCAGTCCCAGGTAGGAATAAGTCAAGGCTGTTT 2000
CircularRNA          -------------------------------------------------------------------------------- -
Cir_Forward.Primer   -------------------------------------------------------------------------------- -
Cir_Reverse.Primer   -------------------------------------------------------------------------------- -

                     2.020               2.040               2.060               2.080
Genomic(MLOC_58118)  CTTGTTAGTCTGTCTCTTTCTCTTTAATAGCAGGTTCTTGGGTAAAAAACCCAGTCACTTTAGAAAGTCCCCCTTTTTTT 2080
cDNA(MLOC_58118)     CTTGTTAGTCTGTCTCTTTCTCTTTAATAGCAGGTTCTTGGGTAAAAAACCCAGTCACTTTAGAAAGTCCCCCTTTTTTT 2080
CircularRNA          -------------------------------------------------------------------------------- -
Cir_Forward.Primer   -------------------------------------------------------------------------------- -
Cir_Reverse.Primer   -------------------------------------------------------------------------------- -

                     2.100               2.120               2.140               2.160
Genomic(MLOC_58118)  AGGGAGCAGAGCTGAATAAAAATGGGAAATTCCAATGAATGATAGAGATTTGATTCTCTATGGAGATATCACGTCCAGTG 2160
cDNA(MLOC_58118)     AGGGAGCAGAGCTGAATAAAAATGGGAAATTCCAATGAATGATAGAGATTTGATTCTCTATGGAGATATCACGTCCAGTG 2160
CircularRNA          -------------------------------------------------------------------------------- -
Cir_Forward.Primer   -------------------------------------------------------------------------------- -
Cir_Reverse.Primer   -------------------------------------------------------------------------------- -

                     2.180               2.200               2.220               2.240
Genomic(MLOC_58118)  ATATACGCTTTTATCAAGAAACTCAAACTAATATGTCTTTTTTTTAGTCTCTCATCAACTAGGTCAGATGCGGAAAGCGCT 2240
cDNA(MLOC_58118)     ATATACGCTTTTATCAAGAAACTCAAACTAATATGTCTTTTTTTTAGTCTCTCATCAACTAGGTCAGATGCGGAAAGCGCT 2240
CircularRNA          -------------------------------------------------------------------------------- -
Cir_Forward.Primer   -------------------------------------------------------------------------------- -
Cir_Reverse.Primer   -------------------------------------------------------------------------------- -
```

```
                      2.260                        2.280                        2.300                        2.320
Genomic(MLOC_58118)  CCGCCGGAGGCTTCCGACTTGTTTATGAGGATTTGTGCAGAATGCACAAGATTAGTGGAGCAAGAAGGGAAACAGATACC 2320
cDNA(MLOC_58118)     CCGCCGGAGGCTTCCGACTTGTTTATGAGGATTTGTGCAGAATGCACAAGATTAGTGGAGCAAGAAGGGAAACAGATACC 2320
CircularRNA          -------------------------------------------------------------------------------- -
Cir_Forward.Primer   -------------------------------------------------------------------------------- -
Cir_Reverse.Primer   -------------------------------------------------------------------------------- -

                      2.340                        2.360                        2.380                        2.400
Genomic(MLOC_58118)  CGCACAATGGGACATGCCTGACCTTCTTCGGGCTGTGAACGAAAACATAATAGTAATGCCAGACTTTCTAAGAGAGACTT 2400
cDNA(MLOC_58118)     CGCACAATGGGACATGCCTGACCTTCTTCGGGCTGTGAACGAAAACATAATAGTAATGCCAGACTTTCTAAGAGAGACTT 2400
CircularRNA          -------------------------------------------------------------------------------- -
Cir_Forward.Primer   -------------------------------------------------------------------------------- -
Cir_Reverse.Primer   -------------------------------------------------------------------------------- -

                      2.420                        2.440                        2.460                        2.480
Genomic(MLOC_58118)  ATTATGATGTCATGTTACGTGGACCTCATAGTTGGTTATTCCTTGATGTTAGCAACTGGATTGATTTAGTCAACTACGCC 2480
cDNA(MLOC_58118)     ATTATGATGTCATGTTACGTGGACCTCATAGTTGGTTATTCCTTGATGTTAGCAACTGGATTGATTTAGTCAACTACGCC 2480
CircularRNA          -------------------------------------------------------------------------------- -
Cir_Forward.Primer   -------------------------------------------------------------------------------- -
Cir_Reverse.Primer   -------------------------------------------------------------------------------- -

                      2.500                        2.520                        2.540                        2.560
Genomic(MLOC_58118)  CCCCAAGTATGAAGGGGCGGGTGTTGGGAGATTTTACCCAAGAAATGGGTACATAAAATGAAAAGATCGGAACATGGGAA 2560
cDNA(MLOC_58118)     CCCCAAGTATGAAGGGGCGGGTGTTGGGAGATTTTACCCAAGAAATGGGTACATAAAATGAAAAGATCGGAACATGGGAA 2560
CircularRNA          -------------------------------------------------------------------------------- -
Cir_Forward.Primer   -------------------------------------------------------------------------------- -
Cir_Reverse.Primer   -------------------------------------------------------------------------------- -

                      2.580                        2.600                        2.620                        2.640
Genomic(MLOC_58118)  TAGATCTTATACCAATACTGACTACCCATTTCCATTATGTACTTTCTATGGGAGCCGTTTTTGCTTTATTTGCTGGATTT 2640
cDNA(MLOC_58118)     TAGATCTTATACCAATACTGACTACCCATTTCCATTATGTACTTTCTATGGGAGCCGTTTTTGCTTTATTTGCTGGATTT 2640
CircularRNA          -------------------------------------------------------------------------------- -
Cir_Forward.Primer   -------------------------------------------------------------------------------- -
Cir_Reverse.Primer   -------------------------------------------------------------------------------- -

                      2.660                        2.680                        2.700                        2.720
Genomic(MLOC_58118)  TACTATTGGGTTCGCGGTATGAGAATGGAGGTTCAGCTTCTCGAGCCAAGGAACTCCCATGTCTCCCCCCCTAAAACGGG 2720
cDNA(MLOC_58118)     TACTATTGGGTTCGCGGTATGAGAATGGAGGTTCAGCTTCTCGAGCCAAGGAACTCCCATGTCTCCCCCCCTAAAACGGG 2720
CircularRNA          -------------------------------------------------------------------------------- -
Cir_Forward.Primer   -------------------------------------------------------------------------------- -
Cir_Reverse.Primer   -------------------------------------------------------------------------------- -

                      2.740                        2.760                        2.780                        2.800
Genomic(MLOC_58118)  GGCCTAATAAGAAAGACCAATCCAAAACTTATTCGTTTAGTTGGTAGAACCCGCGCCAATATAATATTGACTCTCTCTCG 2800
cDNA(MLOC_58118)     GGCCTAATAAGAAAGACCAATCCAAAACTTATTCGTTTAGTTGGTAGAACCCGCGCCAATATAATATTGACTCTCTCTCG 2800
CircularRNA          -------------------------------------------------------------------------------- -
Cir_Forward.Primer   -------------------------------------------------------------------------------- -
Cir_Reverse.Primer   -------------------------------------------------------------------------------- -

                      2.820                        2.840                        2.860                        2.880
Genomic(MLOC_58118)  TCCAATAAGAGTTTCCGAGAGTTACTTTATTCAAATTCTCTCCTTTCCAATTTCCATCAAGGCAAGGGGGTAAATAAGGG 2880
cDNA(MLOC_58118)     TCCAATAAGAGTTTCCGAGAGTTACTTTATTCAAATTCTCTCCTTTCCAATTTCCATCAAGGCAAGGGGGTAAATAAGGG 2880
CircularRNA          -------------------------------------------------------------------------------- -
Cir_Forward.Primer   -------------------------------------------------------------------------------- -
Cir_Reverse.Primer   -------------------------------------------------------------------------------- -

                      2.900                        2.920                        2.940                        2.960
Genomic(MLOC_58118)  GGAAGAGGAGTTGTAACGATAGAAAAGAGAAATGACTATAAGGAACCAACGATTCTCTCTTCTTAAACAACCTATATACT 2960
cDNA(MLOC_58118)     GGAAGAGGAGTTGTAACGATAGAAAAGAGAAATGACTATAAGGAACCAACGATTCTCTCTTCTTAAACAACCTATATACT 2960
CircularRNA          -------------------------------------------------------------------------------- -
Cir_Forward.Primer   -------------------------------------------------------------------------------- -
Cir_Reverse.Primer   -------------------------------------------------------------------------------- -

                      2.980                        3.000                        3.020                        3.040
Genomic(MLOC_58118)  CCACACTTAACCAGCATTTGATAGATTATCCAACCCCGAGCAATCTTAGTTATTGGTGGGGGTTCGGTTCGTTAGCAGGT 3040
cDNA(MLOC_58118)     CCACACTTAACCAGCATTTGATAGATTATCCAACCCCGAGCAATCTTAGTTATTGGTGGGGGTTCGGTTCGTTAGCAGGT 3040
CircularRNA          -------------------------------------------------------------------------------- -
Cir_Forward.Primer   -------------------------------------------------------------------------------- -
Cir_Reverse.Primer   -------------------------------------------------------------------------------- -

                      3.060                        3.080                        3.100                        3.120
Genomic(MLOC_58118)  ATTTGTTTAGTCATTCAGATAGTGACTGGCGTTTTTTTAGCTATGCATCACACACCTCATGTGGATCTAGCTTTCAACAG 3120
cDNA(MLOC_58118)     ATTTGTTTAGTCATTCAGATAGTGACTGGCGTTTTTTTAGCTATGCATCACACACCTCATGTGGATCTAGCTTTCAACAG 3120
CircularRNA          -------------------------------------------------------------------------------- -
Cir_Forward.Primer   -------------------------------------------------------------------------------- -
Cir_Reverse.Primer   -------------------------------------------------------------------------------- -

                      3.140                        3.160                        3.180                        3.200
Genomic(MLOC_58118)  CGTAGAACACATTATGAGAGATGTTGAAGGGGGCTGGTTGCTCCGTTATATGCATGCTAATGGGGCAAGTATGTTTCTCA 3200
cDNA(MLOC_58118)     CGTAGAACACATTATGAGAGATGTTGAAGGGGGCTGGTTGCTCCGTTATATGCATGCTAATGGGGCAAGTATGTTTCTCA 3200
CircularRNA          -------------------------------------------------------------------------------- -
Cir_Forward.Primer   -------------------------------------------------------------------------------- -
Cir_Reverse.Primer   -------------------------------------------------------------------------------- -

                      3.220                        3.240                        3.260                        3.280
Genomic(MLOC_58118)  TTGTGGTTCACCTTCATATTTTTCGTGGTCTATATCATGCGAGTTATAGCAGTCCTAGGGAATTTGTTCGGTGTCTCGGA 3280
cDNA(MLOC_58118)     TTGTGGTTCACCTTCATATTTTTCGTGGTCTATATCATGCGAGTTATAGCAGTCCTAGGGAATTTGTTCGGTGTCTCGGA 3280
CircularRNA          -----------CTTCATATTTTTCGTGGTCTATATCATGCGAGTTATAGCAGTCCTAGGGAATTTGTTCGGTGTCTCGGA 69
Cir_Forward.Primer   -------------------------------------------------------------------------------- -
Cir_Reverse.Primer   -------------------------------------TGCGAGTTATAGCAGTCCTAGGGA------------------- 24

                      3.300                        3.320                        3.340                        3.360
Genomic(MLOC_58118)  GTTGTCATATTCCTATTAATGATTGTGACAGCTTTTATAGGATACGTACCACCTTGGGGTCAGATGAGCTTTTGGGGAGC 3360
cDNA(MLOC_58118)     GTTGTCATATTCCTATTAATGATTGTGACAGCTTTTATAGGATACGTACCACCTTGGGGTCAGATGAGCTTTTGGGGAGC 3360
CircularRNA          GTTGTCATATTCCTATTAATGATTGTGACAGCTTTTATAGGATACGTACCACCTTGGGGTCAGATGAGCTTTTGGGGAGC 149
Cir_Forward.Primer   -------------------------------------------------------------------------------- -
Cir_Reverse.Primer   -------------------------------------------------------------------------------- 24
```

```
                              3.380               3.400               3.420               3.440
Genomic(MLOC_58118)  AACAGTAATTACAAGCTTAGCTAGCGCCATACCAGTAGTAGGAGATACCATAGTGACTTGGCTTTGGGGTGGTTTCTCCG 3440
   cDNA(MLOC_58118)  AACAGTAATTACAAGCTTAGCTAGCGCCATACCAGTAGTAGGAGATACCATAGTGACTTGGCTTTGGGGTGGTTTCTCCG 3440
        CircularRNA  AACAGTAATTACAAGCTTAGCTAGCGCCATACCAGTAGTAGGAGATACCATAGTGACTTGGCTTTGGGGTGGTTTCTCCG 229
  Cir_Forward.Primer --------------------------------------------------------------------------------- -
  Cir_Reverse.Primer --------------------------------------------------------------------------------- 24

                              3.460               3.480               3.500               3.520
Genomic(MLOC_58118)  TGGACAATGCCACCTTAAATCGTTTTTTTAGTCTCCATCATTTACTCCCCCTTATTTTAGTAGGCGCCAGTCTTCTTCAT 3520
   cDNA(MLOC_58118)  TGGACAATGCCACCTTAAATCGTTTTTTTAGTCTCCATCATTTACTCCCCCTTATTTTAGTAGGCGCCAGTCTTCTTCAT 3520
        CircularRNA  TGGACAATGCCACCTTAAATCGTTTTTTTAGTCTCCATCATTTACTCCCCCTTATTTTAGTAGGCGCCAGTCTTCTTCAT 309
  Cir_Forward.Primer --------------------------------------------------------------------------------- -
  Cir_Reverse.Primer --------------------------------------------------------------------------------- 24

                              3.540               3.560               3.580               3.600
Genomic(MLOC_58118)  CTGGCCGCATTGCATCAATATGGATCAAATAATCCATTGGGTGTACATTCTGAGATGGATAAAATTGCTTCTTACCCTTA 3600
   cDNA(MLOC_58118)  CTGGCCGCATTGCATCAATATGGATCAAATAATCCATTGGGTGTACATTCTGAGATGGATAAAATTGCTTCTTACCCTTA 3600
        CircularRNA  CTGGCCGCATTGCATCAATATGGATCAAATAATCCATTGGGTGTACATTCTGAGATGGATAAAATTGCTTCTTACCCTTA 389
  Cir_Forward.Primer --------------------------------------------------------------------------------- -
  Cir_Reverse.Primer --------------------------------------------------------------------------------- 24

                              3.620               3.640               3.660               3.680
Genomic(MLOC_58118)  TTTTTATGTAAAGGATCTTGTAGGTCGGGTAGCTTCTGCTATCTTTTTTTCCATTTGGATTTTTTTTGCTCCTAATGTTT 3680
   cDNA(MLOC_58118)  TTTTTATGTAAAGGATCTTGTAGGTCGGGTAGCTTCTGCTATCTTTTTTTCCATTTGGATTTTTTTTGCTCCTAATGTTT 3680
        CircularRNA  TTTTTATGTAAAGGATCTTGTAGGTCGGGTAGCTTCTGCTATCTTTTTTTCCATTTGGATTTTTTTTGCTCCTAATGTTT 469
  Cir_Forward.Primer --------------------------------------------------------------------------------- -
  Cir_Reverse.Primer --------------------------------------------------------------------------------- 24

                              3.700               3.720               3.740               3.760
Genomic(MLOC_58118)  TGGGGCATCCCGACAATTATATACCTGCTAATCCGATGCCCACCCCGCCTCATATTGTGCCGGAATGGTATTTCCTACCG 3760
   cDNA(MLOC_58118)  TGGGGCATCCCGACAATTATATACCTGCTAATCCGATGCCCACCCCGCCTCATATTGTGCCGGAATGGTATTTCCTACCG 3760
        CircularRNA  TGGGGCATCCCGACAATTATATACCTGCTAATCCGATGCCCACCCCGCCTCATATTGTGCCGGAATGGTATTTCCTACCG 549
  Cir_Forward.Primer --------------------------------------------------------------------------------- -
  Cir_Reverse.Primer --------------------------------------------------------------------------------- 24

                              3.780               3.800               3.820               3.840
Genomic(MLOC_58118)  ATCCATGCCATTCTTCGCAGTATACCTGACAAAGCGGGAGGTGTAGCCGCAATAGCACCAGTTTTTATATCTCTCTTGGC 3840
   cDNA(MLOC_58118)  ATCCATGCCATTCTTCGCAGTATACCTGACAAAGCGGGAGGTGTAGCCGCAATAGCACCAGTTTTTATATCTCTCTTGGC 3840
        CircularRNA  ATCCATGCCATTCTTCGCAGTATACCTGACAAAGCGGGAGGTGTAGCCGCAATAGCACTAGTTTTTATATCTCTCTTGGC 629
  Cir_Forward.Primer ------------------------------------------------------GCACTAGTTTTTATATCTCTCTTGGC 26
  Cir_Reverse.Primer -------------------------------------------------------------------------------- 24

                              3.860               3.880               3.900               3.920
Genomic(MLOC_58118)  TTTACCTTTTTTTAAAGAAATGTATGTGCGTAGTTCAAGTTTTCGACCGATTCACCAAGGAATATTTTGGTTGCTTTTGG 3920
   cDNA(MLOC_58118)  TTTACCTTTTTTTAAAGAAATGTATGTGCGTAGTTCAAGTTTTCGACCGATTCACCAAGGAATATTTTGGTTGCTTTTGG 3920
        CircularRNA  TTTAC--------------------------------------------------------------------------- 634
  Cir_Forward.Primer T------------------------------------------------------------------------------- 27
  Cir_Reverse.Primer -------------------------------------------------------------------------------- 24

                              3.940               3.960               3.980               4.000
Genomic(MLOC_58118)  CGGATTGCTTACTACTAGGTTGGATCGGATGTCAACCTGTGGAGGCACCATTTGTTACTATTGGACAAATTCCTTCTGTC 4000
   cDNA(MLOC_58118)  CGGATTGCTTACTACTAGGTTGGATCGGATGTCAACCTGTGGAGGCACCATTTGTTACTATTGGACAAATTCCTTCTGTC 4000
        CircularRNA  -------------------------------------------------------------------------------- 634
  Cir_Forward.Primer -------------------------------------------------------------------------------- 27
  Cir_Reverse.Primer -------------------------------------------------------------------------------- 24

                              4.020               4.040               4.060               4.080
Genomic(MLOC_58118)  TTTTTCTTCTTGTTCTTTGCCATAACGCCCATTCCGGGACGAGTTGGAAGAGGAATTCCAAAATATTACACGGATGAGAC 4080
   cDNA(MLOC_58118)  TTTTTCTTCTTGTTCTTTGCCATAACGCCCATTCCGGGACGAGTTGGAAGAGGAATTCCAAAATATTACACGGATGAGAC 4080
        CircularRNA  -------------------------------------------------------------------------------- 634
  Cir_Forward.Primer -------------------------------------------------------------------------------- 27
  Cir_Reverse.Primer -------------------------------------------------------------------------------- 24

                              4.100               4.120               4.140               4.160
Genomic(MLOC_58118)  TCATCGCACCGGATCCTTAGACGGATGAGACTGATCACACCTGATCAGTGATCAATTCTGGCACAATGAATTTACGAGTT 4160
   cDNA(MLOC_58118)  TCATCGCACCGGATCCTTAGACGGATGAGACTGATCACACCTGATCAGTGATCAATTCTGGCACAATGAATTTACGAGTT 4160
        CircularRNA  -------------------------------------------------------------------------------- 634
  Cir_Forward.Primer -------------------------------------------------------------------------------- 27
  Cir_Reverse.Primer -------------------------------------------------------------------------------- 24

                              4.180               4.200               4.220               4.240
Genomic(MLOC_58118)  ATTTTACACAATGAATTTACAAGCGAGTTTGCAACGGTAGACCTATCTCCTGAAAAGAGTTTGGTGGTCGTAGGCTCACT 4240
   cDNA(MLOC_58118)  ATTTTACACAATGAATTTACAAGCGAGTTTGCAACGGTAGACCTATCTCCTGAAAAGAGTTTGGTGGTCGTAGGCTCACT 4240
        CircularRNA  -------------------------------------------------------------------------------- 634
  Cir_Forward.Primer -------------------------------------------------------------------------------- 27
  Cir_Reverse.Primer -------------------------------------------------------------------------------- 24

                              4.260               4.280               4.300               4.320
Genomic(MLOC_58118)  CACCGGACAATCCAGTGTATGCGTTACAAGGCAACTAGCATTTAGTTCGTGAAAGAATGTTTCGTTGGAAAAACCAACGC 4320
   cDNA(MLOC_58118)  CACCGGACAATCCAGTGTATGCGTTACAAGGCAACTAGCATTTAGTTCGTGAAAGAATGTTTCGTTGGAAAAACCAACGC 4320
        CircularRNA  -------------------------------------------------------------------------------- 634
  Cir_Forward.Primer -------------------------------------------------------------------------------- 27
  Cir_Reverse.Primer -------------------------------------------------------------------------------- 24

                              4.340               4.360               4.380               4.400
Genomic(MLOC_58118)  CGACGTCAAGATCAGTCTCCTTTCTCTTTTCGGGAGCAGAGCTGAAAAAGATGGGAAGTAACGATGGATCCTTGGATTTT 4400
   cDNA(MLOC_58118)  CGACGTCAAGATCAGTCTCCTTTCTCTTTTCGGGAGCAGAGCTGAAAAAGATGGGAAGTAACGATGGATCCTTGGATTTT 4400
        CircularRNA  -------------------------------------------------------------------------------- 634
  Cir_Forward.Primer -------------------------------------------------------------------------------- 27
  Cir_Reverse.Primer -------------------------------------------------------------------------------- 24

                              4.420               4.440               4.460               4.480
Genomic(MLOC_58118)  TCCATCATCAATAGACGGGATGCTTATCGAATTTGACCCTTCGTCGTCATACCCAAATTCGGTCAATGGACCGGCTTTGA 4480
   cDNA(MLOC_58118)  TCCATCATCAATAGACGGGATGCTTATCGAATTTGACCCTTCGTCGTCATACCCAAATTCGGTCAATGGACCGGCTTTGA 4480
        CircularRNA  -------------------------------------------------------------------------------- 634
  Cir_Forward.Primer -------------------------------------------------------------------------------- 27
  Cir_Reverse.Primer -------------------------------------------------------------------------------- 24
```

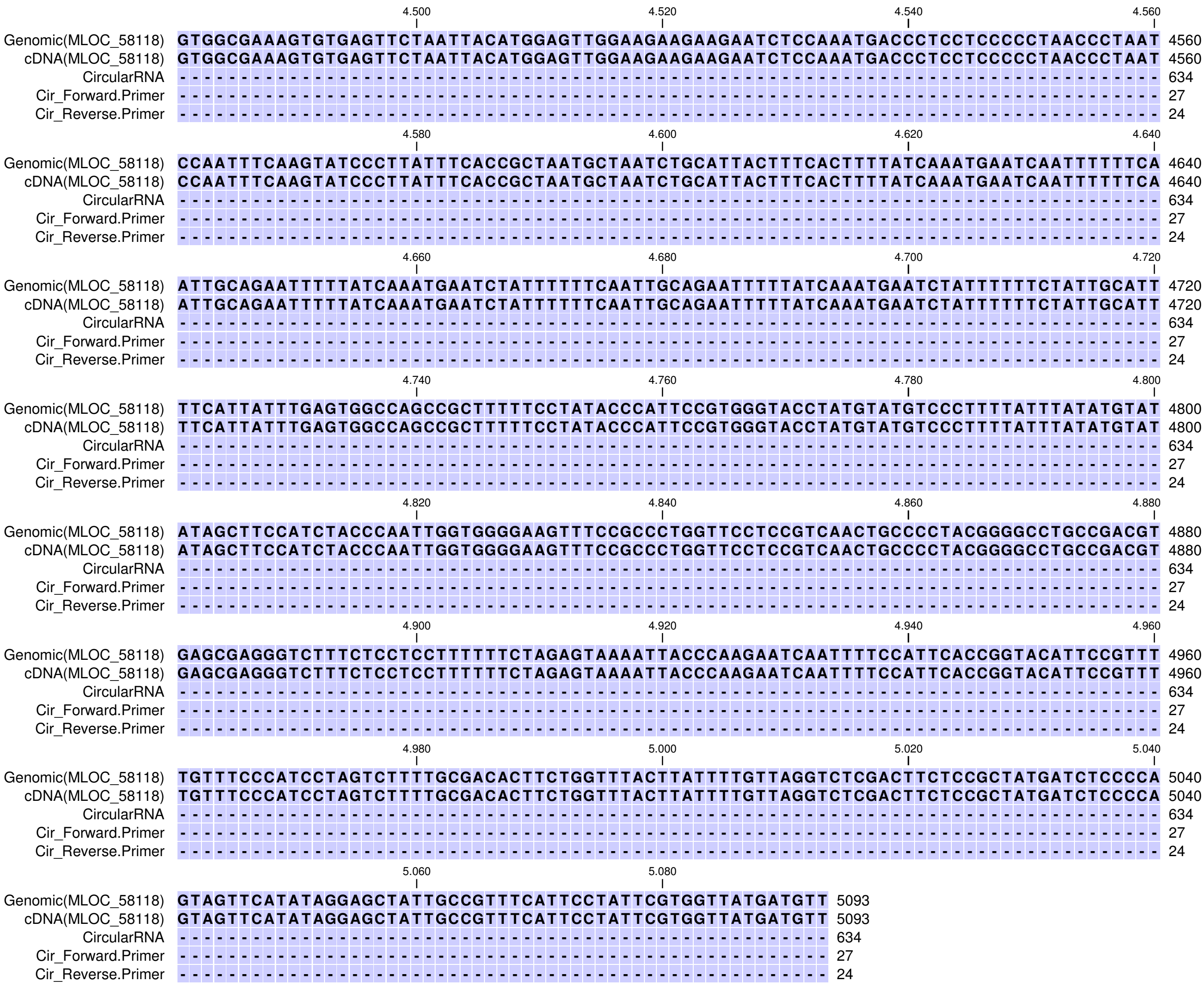

4.500 4.520 4.540 4.560
Genomic(MLOC_58118) GTGGCGAAAGTGTGAGTTCTAATTACATGGAGTTGGAAGAAGAAGAATCTCCAAATGACCCTCCTCCCCCTAACCCTAAT 4560
cDNA(MLOC_58118) GTGGCGAAAGTGTGAGTTCTAATTACATGGAGTTGGAAGAAGAAGAATCTCCAAATGACCCTCCTCCCCCTAACCCTAAT 4560
CircularRNA 634
Cir_Forward.Primer 27
Cir_Reverse.Primer 24
4.580 4.600 4.620 4.640
Genomic(MLOC_58118) CCAATTTCAAGTATCCCTTATTTCACCGCTAATGCTAATCTGCATTACTTTCACTTTTATCAAATGAATCAATTTTTTCA 4640
cDNA(MLOC_58118) CCAATTTCAAGTATCCCTTATTTCACCGCTAATGCTAATCTGCATTACTTTCACTTTTATCAAATGAATCAATTTTTTCA 4640
CircularRNA 634
Cir_Forward.Primer 27
Cir_Reverse.Primer 24
4.660 4.680 4.700 4.720
Genomic(MLOC_58118) ATTGCAGAATTTTTATCAAATGAATCTATTTTTTCAATTGCAGAATTTTTATCAAATGAATCTATTTTTTCTATTGCATT 4720
cDNA(MLOC_58118) ATTGCAGAATTTTTATCAAATGAATCTATTTTTTCAATTGCAGAATTTTTATCAAATGAATCTATTTTTTCTATTGCATT 4720
CircularRNA 634
Cir_Forward.Primer 27
Cir_Reverse.Primer 24
4.740 4.760 4.780 4.800
Genomic(MLOC_58118) TTCATTATTTGAGTGGCCAGCCGCTTTTTCCTATACCCATTCCGTGGGTACCTATGTATGTCCCTTTTATTTATATGTAT 4800
cDNA(MLOC_58118) TTCATTATTTGAGTGGCCAGCCGCTTTTTCCTATACCCATTCCGTGGGTACCTATGTATGTCCCTTTTATTTATATGTAT 4800
CircularRNA 634
Cir_Forward.Primer 27
Cir_Reverse.Primer 24
4.820 4.840 4.860 4.880
Genomic(MLOC_58118) ATAGCTTCCATCTACCCAATTGGTGGGGAAGTTTCCGCCCTGGTTCCTCCGTCAACTGCCCCTACGGGGCCTGCCGACGT 4880
cDNA(MLOC_58118) ATAGCTTCCATCTACCCAATTGGTGGGGAAGTTTCCGCCCTGGTTCCTCCGTCAACTGCCCCTACGGGGCCTGCCGACGT 4880
CircularRNA 634
Cir_Forward.Primer 27
Cir_Reverse.Primer 24
4.900 4.920 4.940 4.960
Genomic(MLOC_58118) GAGCGAGGGTCTTTCTCCTCCTTTTTTCTAGAGTAAAATTACCCAAGAATCAATTTTCCATTCACCGGTACATTCCGTTT 4960
cDNA(MLOC_58118) GAGCGAGGGTCTTTCTCCTCCTTTTTTCTAGAGTAAAATTACCCAAGAATCAATTTTCCATTCACCGGTACATTCCGTTT 4960
CircularRNA 634
Cir_Forward.Primer 27
Cir_Reverse.Primer 24
4.980 5.000 5.020 5.040
Genomic(MLOC_58118) TGTTTCCCATCCTAGTCTTTTGCGACACTTCTGGTTTACTTATTTTGTTAGGTCTCGACTTCTCCGCTATGATCTCCCCA 5040
cDNA(MLOC_58118) TGTTTCCCATCCTAGTCTTTTGCGACACTTCTGGTTTACTTATTTTGTTAGGTCTCGACTTCTCCGCTATGATCTCCCCA 5040
CircularRNA 634
Cir_Forward.Primer 27
Cir_Reverse.Primer 24
5.060 5.080
Genomic(MLOC_58118) GTAGTTCATATAGGAGCTATTGCCGTTTCATTCCTATTCGTGGTTATGATGTT 5093
cDNA(MLOC_58118) GTAGTTCATATAGGAGCTATTGCCGTTTCATTCCTATTCGTGGTTATGATGTT 5093
CircularRNA 634
Cir_Forward.Primer 27
Cir_Reverse.Primer 24

Real-Time PCR for the junction region of apocytochrome b_circular RNA

(ID: Morex_contig_42365:12844-13477**)**

By divergent primers ◀▶ on genomic DNA

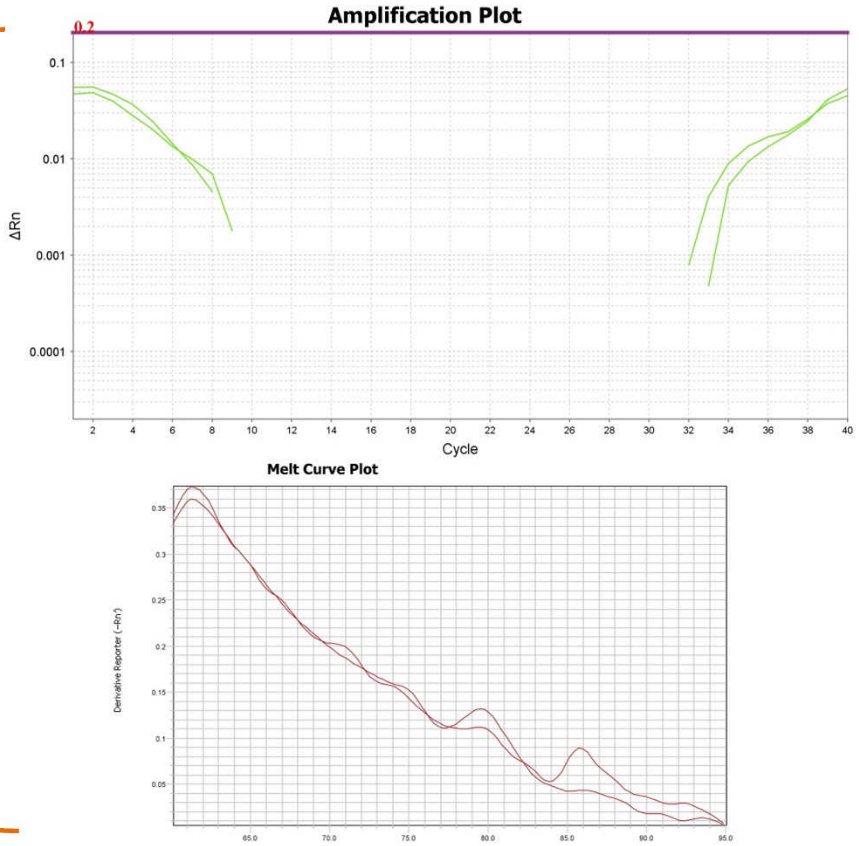

By divergent primers ◀▶ on cDNAs

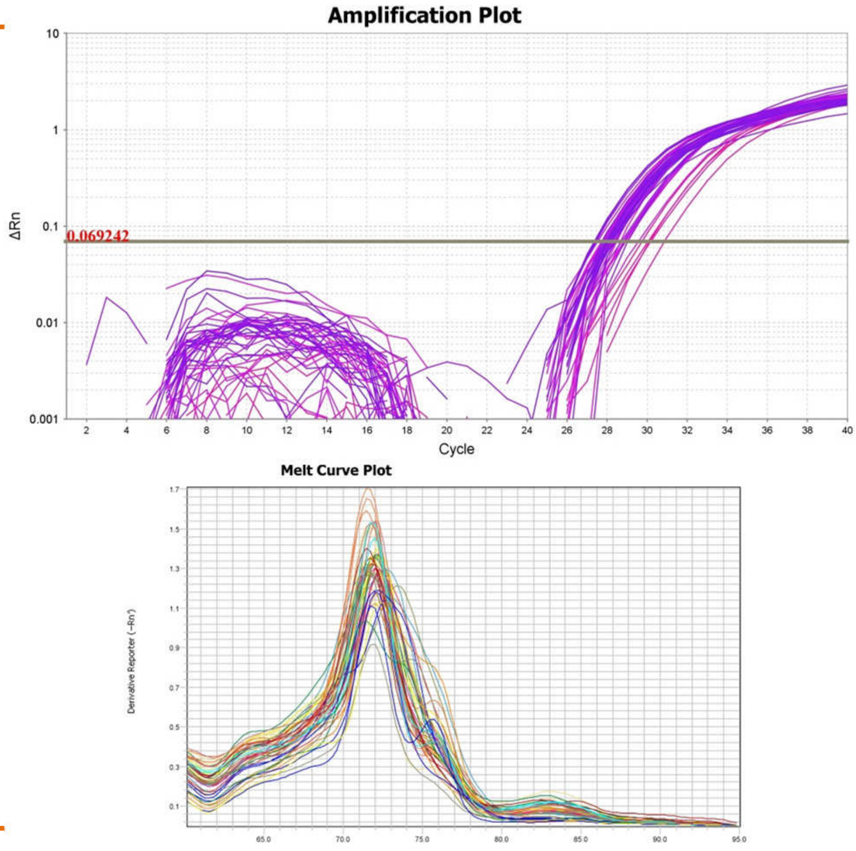

Real-Time PCR for the junction region of apocytochrome b_circular RNA

(ID: Morex_contig_42365:12844-13477**)**

By divergent primers ◀▶ & with no template

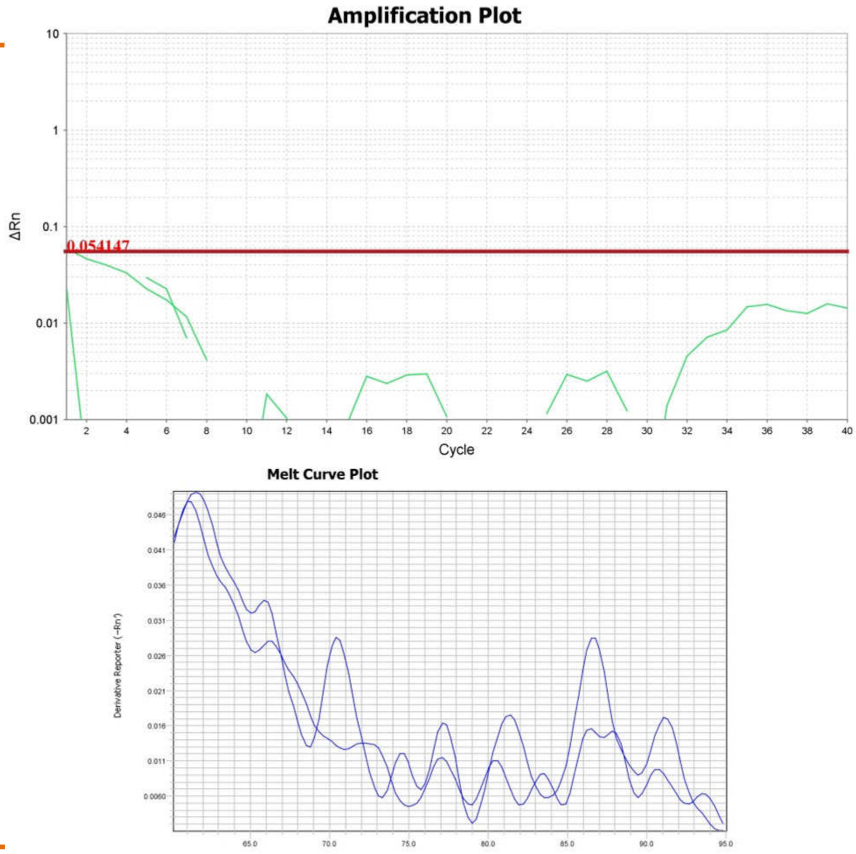

Real-Time PCR for apocytochrome b

By convergent primers ▶◀ on cDNAs

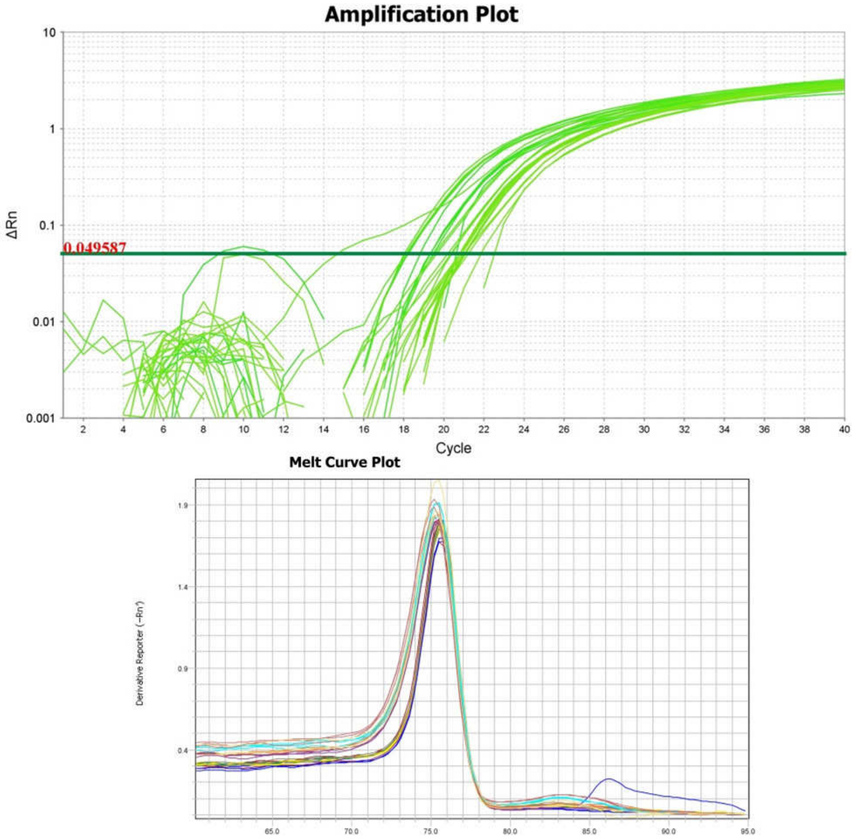

By convergent primers ▶◀ & with no template

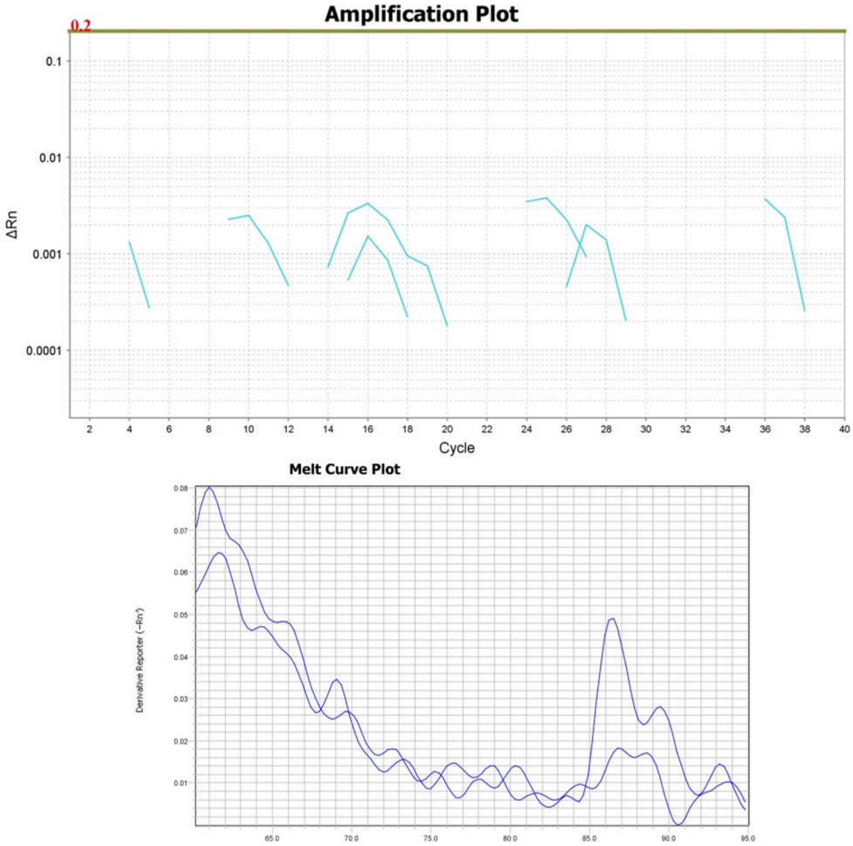

Cytochrome c oxidase Cox1_circular RNA1 (ID: Ch1:23865393-238657407**)**

TATTGGGACTCTCTATTTCATCTTCGGTGCCATTGCAGGAGTGATGGGCACATGCTTCTCCGTAC
TGATTCGTATGGAATTAGCCCGACCCGGCGATCAAATTCTTGGTGGGAATCATCAACTTTATAATG
TTTTAATAACGGCTCACGCTTTTTTAATGATCTTTTTTATGGTTATGCCGGCGATGATAGGTGGATT
TGGTAATTGGTTTGTTCCGATTCTGATAGGTGCACCTGACATGGCATTTCCACGATTAAATAATATA
TCATTCTGG**TTGTTGCCACCAAGTCTCTTGCTCCTATTAAGCTCAGCCTTAGTAGAAGTGGGCAG
CGGCACTGGGTGGACAG**

Cytochrome c oxidase Cox1_circular RNA2 (ID: Ch1:23865228-23865597**)**

GGCTCACGCTTTTTTAATGATCTTTTTTATGGTTATGCCGGCGATGATAGGTGGATTTGGTAATTG
GTTTGTTCCGATTCTGATAGGTGCACCTGACATGGCATTTCCACGATTAAATAATATATCATTCTGG
TTGTTGCCACCAAGTCTCTTGCTCCTATTAAGCTCAGCCTTAGTAGAAGTGGGCAGCGGCACTGG
GTGGACAGTCTATCCGCCCTTAAGTGGTATTACCAGCCATTCTGGAGGAGCAGTTGATTTAGCAA
TTTTTAGTCTTCATCTATCAGGTATTTCATCAATTTTAGGTTCTATCAATTTTATAACAACTA**TCTTCA
ACATGCGTGGACCTGGAATGACTATGCATAGATTACCA**

Cytochrome c oxidase Cox1_circular RNA3 (ID: Ch1:23865348-23865740**)**

GATATTGGGACTCTCTATTTCATCTTCGGTGCCATTGCAGGAGTGATGGGCACATGCTTCTCCGT
ACTGATTCGTATGGAATTAGCCCGACCCGGCGATCAAATTCTTGGTGGGAATCATCAACTTTATAA
TGTTTTAATAACGGCTCACGCTTTTTTAATGATCTTTTTTATGGTTATGCCGGCGATGATAGGTGGA
TTTGGTAATTGGTTTGTTCCGATTCTGATAGGTGCACCTGACATGGCATTTCCACGATTAAATAATA
TATCATTCTGGTTGTTGCCACCAAGTCTCTTGCTCCTATTAAGCTCAGCCTTAGTAGAAGTGGGCA
GCGGCACTGGGTGGACAGTCTATCC**GCCCTTAAGTGGTATTACCAGCCATTCTGGAGGAGCA**

The nucleotides of junction-region are underlined. The nucleotides of junction-region which are supported by the junction-spaning sequencing reads are shown in red. Introns are not shown if the absence is supported by sequencing reads. In the absence of supporting sequencing reads, the intronic nucletides are shown as N.

Cytochrome c oxidase Cox1_circular RNA4 (ID: Ch1:23864364-23866057)

GGCGGAAAGCTCCGCAAGGAGGTGCCTGGAAGTGGAACAGAGGATCCGGTGGGAAGAAATCC
CCAAGAGCAAGGCAAGTCTCGAAAAGGCGGAGCACGAGCATGCTCTCGACTTGTTTAAGTCGGA
GGATCTTAGAAGGGAATTAGAAAAAAAAAGAGCGGGGTAGCTCAGTAATTCTGATTCTTTTCTCTT
CCAGCGTTTACTATTCTCTTTCTTCTCTTCCAGCCCCCCGGCCCCTCTTTGATAAGGAAAGTTTTC
ATTTCTAAAAAACAGCAAATGACAAATCTGGTTCGATGGCTCTTCTCTACTAACCACAAGGATATTG
GGACTCTCTATTTCATCTTCGGTGCCATTGCAGGAGTGATGGGCACATGCTTCTCCGTACTGATTC
GTATGGAATTAGCCCGACCCGGCGATCAAATTCTTGGTGGGAATCATCAACTTTATAATGTTTTAA
TAACGGCTCACGCTTTTTTAATGATCTTTTTTATGGTTATGCCGGCGATGATAGGTGGATTTGGTAA
TTGGTTTGTTCCGATTCTGATAGGTGCACCTGACATGGCATTTCCACGATTAAATAATATATCATTC
TGGTTGTTGCCACCAAGTCTCTTGCTCCTATTAAGCTCAGCCTTAGTAGAAGTGGGCAGCGGCAC
TGGGTGGACAGTCTATCCGCCCTTAAGTGGTATTACCAGCCATTCTGGAGGAGCAGTTGATTTAG
CAATTTTTAGTCTTCATCTATCAGGTATTTCATCAATTTTAGGTTCTATCAATTTTATAACAACTATCT
TCAACATGCGTGGACCTGGAATGACTATGCATAGATTACCACTTTTTGTGTGGTCCGTTCTAGTGA
CAGCATTCCTACTTTTATTATCACTTCCGGTACTGGCGGGGGCAATTACAATGTTATTAACCGATC
GAAACTTTAATACAACCTTTTTTGATCCTGCAGGAGGGGGAGACCCAATATTATACCAGCATCTCT
TTTGGTTCTTCGGTCATCCAGAGGTGTATATTCTCATTCTGCCTGGATTTGGTATTATTAGTCATAT
CGTATCGACCTTTTCAAGAAAACCGGTCTTCGGGTATCTAGGCATGGTTTATGCCATGATAAGTAT
AGGTGTTCTTGGATTTCTAGTTTGGGCTCATCATATGTTTACTGTGGGCTTAGACGTTGATACGCG
TGCCTACTTCACCGCAGCTACCATGATCATAGCTGTGCCCACAGGAATCAAAATCTTTAGTTGGAT
CGCTACCATGTGGGGAGGTTCGATACAATACAAAACACCCATGTTATTTGCTGTAGGGTTCATCTT
TTTGTTCACCATAGGAGGGCTCACTGGAATAGTTCTAGCAAACTCTGGGCTAGACATTGCTCTACA
TGATACTTATTATGTGGTTGCACATTTCCATTATGTACTTTCTATGGGAGCCGTTTTTGCTTTATTTG
CTGGATTTTACTATTGGGTGGGTAAAATCTTTGGTCGGACATATCCTGAAACTTTAGGCCAAATCC
ATTTTTGGATCACTTTTTTCGGGGTTAATCTGACCTTCTTTCCCATGCATTTCTTAGGGCTTTCGGG
TATGCCGCGTCGTATTCCAGATTATCCAGATGCTTACGCCGGATGGAATGCTCTGAGCAGTTTCG
G**TTCTTATATATCCGTAGTTGGGATTCGTCGTTTCTTCGTAGT**

The nucleotides of junction-region are underlined. The nucleotides of junction-region which are supported by the junction-spaning sequencing reads are shown in red. Introns are not shown if the absence is supported by sequencing reads. In the absence of supporting sequencing reads, the intronic nucletides are shown as N.

Cytochrome c oxidase Cox1_circular RNA5 (ID: Ch1:23864474-23866037**)**

GGTGCCTGGAAGTGGAACAGAGGATCCGGTGGGAAGAAATCCCCAAGAGCAAGGCAAGTCTCG
AAAAGGCGGAGCACGAGCATGCTCTCGACTTGTTTAAGTCGGAGGATCTTAGAAGGGAATTAGAA
AAAAAAAGAGCGGGGTAGCTCAGTAATTCTGATTCTTTTCTCTTCCAGCGTTTACTATTCTCTTTCT
TCTCTTCCAGCCCCCCGGCCCCTCTTTGATAAGGAAAGTTTTCATTTCTAAAAAACAGCAAATGAC
AAATCTGGTTCGATGGCTCTTCTCTACTAACCACAAGGATATTGGGACTCTCTATTTCATCTTCGGT
GCCATTGCAGGAGTGATGGGCACATGCTTCTCCGTACTGATTCGTATGGAATTAGCCCGACCCGG
CGATCAAATTCTTGGTGGGAATCATCAACTTTATAATGTTTTAATAACGGCTCACGCTTTTTTAATG
ATCTTTTTTATGGTTATGCCGGCGATGATAGGTGGATTTGGTAATTGGTTTGTTCCGATTCTGATAG
GTGCACCTGACATGGCATTTCCACGATTAAATAATATATCATTCTGGTTGTTGCCACCAAGTCTCTT
GCTCCTATTAAGCTCAGCCTTAGTAGAAGTGGGCAGCGGCACTGGGTGGACAGTCTATCCGCCC
TTAAGTGGTATTACCAGCCATTCTGGAGGAGCAGTTGATTTAGCAATTTTTAGTCTTCATCTATCAG
GTATTTCATCAATTTTAGGTTCTATCAATTTTATAACAACTATCTTCAACATGCGTGGACCTGGAAT
GACTATGCATAGATTACCACTTTTTGTGTGGTCCGTTCTAGTGACAGCATTCCTACTTTTATTATCA
CTTCCGGTACTGGCGGGGGCAATTACAATGTTATTAACCGATCGAAACTTTAATACAACCTTTTTT
GATCCTGCAGGAGGGGGAGACCCAATATTATACCAGCATCTCTTTTGGTTCTTCGGTCATCCAGA
GGTGTATATTCTCATTCTGCCTGGATTTGGTATTATTAGTCATATCGTATCGACCTTTTCAAGAAAA
CCGGTCTTCGGGTATCTAGGCATGGTTTATGCCATGATAAGTATAGGTGTTCTTGGATTTCTAGTT
TGGGCTCATCATATGTTTACTGTGGGCTTAGACGTTGATACGCGTGCCTACTTCACCGCAGCTAC
CATGATCATAGCTGTGCCCACAGGAATCAAAATCTTTAGTTGGATCGCTACCATGTGGGGAGGTT
CGATACAATACAAAACACCCATGTTATTTGCTGTAGGGTTCATCTTTTTGTTCACCATAGGAGGGC
TCACTGGAATAGTTCTAGCAAACTCTGGGCTAGACATTGCTCTACATGATACTTATTATGTGGTTG
CACATTTCCATTATGTACTTTCTATGGGAGCCGTTTTTGCTTTATTTGCTGGATTTTACTATTGGGT
GGGTAAAATCTTTGGTCGGACATATCCTGAAACTTTAGGCCAAATCCATTTTTGGATCACTTTTTTC
GGGGT**TAATCTGACCTTCTTTCCCATGCATTTCTTAGGGCTTTCG**

The nucleotides of junction-region are underlined. The nucleotides of junction-region which are supported by the junction-spaning sequencing reads are shown in red. Introns are not shown if the absence is supported by sequencing reads. In the absence of supporting sequencing reads, the intronic nucletides are shown as N.

Cytochrome c oxidase Cox1_circular RNA6 (ID: Ch1:23864318-23866037)

AGGTGCCTGGAAGTGGAACAGAGGATCCGGTGGGAAGAAATCCCCAAGAGCAAGGCAAGTCTC
GAAAAGGCGGAGCACGAGCATGCTCTCGACTTGTTTAAGTCGGAGGATCTTAGAAGGGAATTAGA
AAAAAAAAGAGCGGGGTAGCTCAGTAATTCTGATTCTTTTCTCTTCCAGCGTTTACTATTCTCTTTC
TTCTCTTCCAGCCCCCCGGCCCCTCTTTGATAAGGAAAGTTTTCATTTCTAAAAAACAGCAAATGA
CAAATCTGGTTCGATGGCTCTTCTCTACTAACCACAAGGATATTGGGACTCTCTATTTCATCTTCG
GTGCCATTGCAGGAGTGATGGGCACATGCTTCTCCGTACTGATTCGTATGGAATTAGCCCGACCC
GGCGATCAAATTCTTGGTGGGAATCATCAACTTTATAATGTTTTAATAACGGCTCACGCTTTTTTAA
TGATCTTTTTTATGGTTATGCCGGCGATGATAGGTGGATTTGGTAATTGGTTTGTTCCGATTCTGAT
AGGTGCACCTGACATGGCATTTCCACGATTAAATAATATATCATTCTGGTTGTTGCCACCAAGTCT
CTTGCTCCTATTAAGCTCAGCCTTAGTAGAAGTGGGCAGCGGCACTGGGTGGACAGTCTATCCGC
CCTTAAGTGGTATTACCAGCCATTCTGGAGGAGCAGTTGATTTAGCAATTTTTAGTCTTCATCTATC
AGGTATTTCATCAATTTTAGGTTCTATCAATTTTATAACAACTATCTTCAACATGCGTGGACCTGGA
ATGACTATGCATAGATTACCACTTTTTGTGTGGTCCGTTCTAGTGACAGCATTCCTACTTTTATTAT
CACTTCCGGTACTGGCGGGGGCAATTACAATGTTATTAACCGATCGAAACTTTAATACAACCTTTT
TTGATCCTGCAGGAGGGGGAGACCCAATATTATACCAGCATCTCTTTTGGTTCTTCGGTCATCCAG
AGGTGTATATTCTCATTCTGCCTGGATTTGGTATTATTAGTCATATCGTATCGACCTTTTCAAGAAA
ACCGGTCTTCGGGTATCTAGGCATGGTTTATGCCATGATAAGTATAGGTGTTCTTGGATTTCTAGT
TTGGGCTCATCATATGTTTACTGTGGGCTTAGACGTTGATACGCGTGCCTACTTCACCGCAGCTAC
CATGATCATAGCTGTGCCCACAGGAATCAAAATCTTTAGTTGGATCGCTACCATGTGGGGAGGTT
CGATACAATACAAAACACCCATGTTATTTGCTGTAGGGTTCATCTTTTTGTTCACCATAGGAGGGC
TCACTGGAATAGTTCTAGCAAACTCTGGGCTAGACATTGCTCTACATGATACTTATTATGTGGTTG
CACATTTCCATTATGTACTTTCTATGGGAGCCGTTTTTGCTTTATTTGCTGGATTTTACTATTGGGT
GGGTAAAATCTTTGGTCGGACATATCCTGAAACTTTAGGCCAAATCCATTTTTGGATCACTTTTTTC
GGGGTTAATCTGACCTTCTTTCCCATGCATTTCTTAGGGCTTTCGGGTATGCCGCGTCGTATTCCA
GATTATCCAGATGCTTACGCCGGATGGAATGCTCTGAGCAGTTTCGGTTCTTATATATCCGTAGTT
GGGATTCGTCGTTTCTTCGTAGTTGTC**GCAATCACTTCAAGCAGTGGAAAGAACAAAAAATGTG**
CGGA

The nucleotides of junction-region are underlined. The nucleotides of junction-region which are supported by the junction-spaning sequencing reads are shown in red. Introns are not shown if the absence is supported by sequencing reads. In the absence of supporting sequencing reads, the intronic nucletides are shown as N.

Structural relationship between the circular RNA and its parental gene

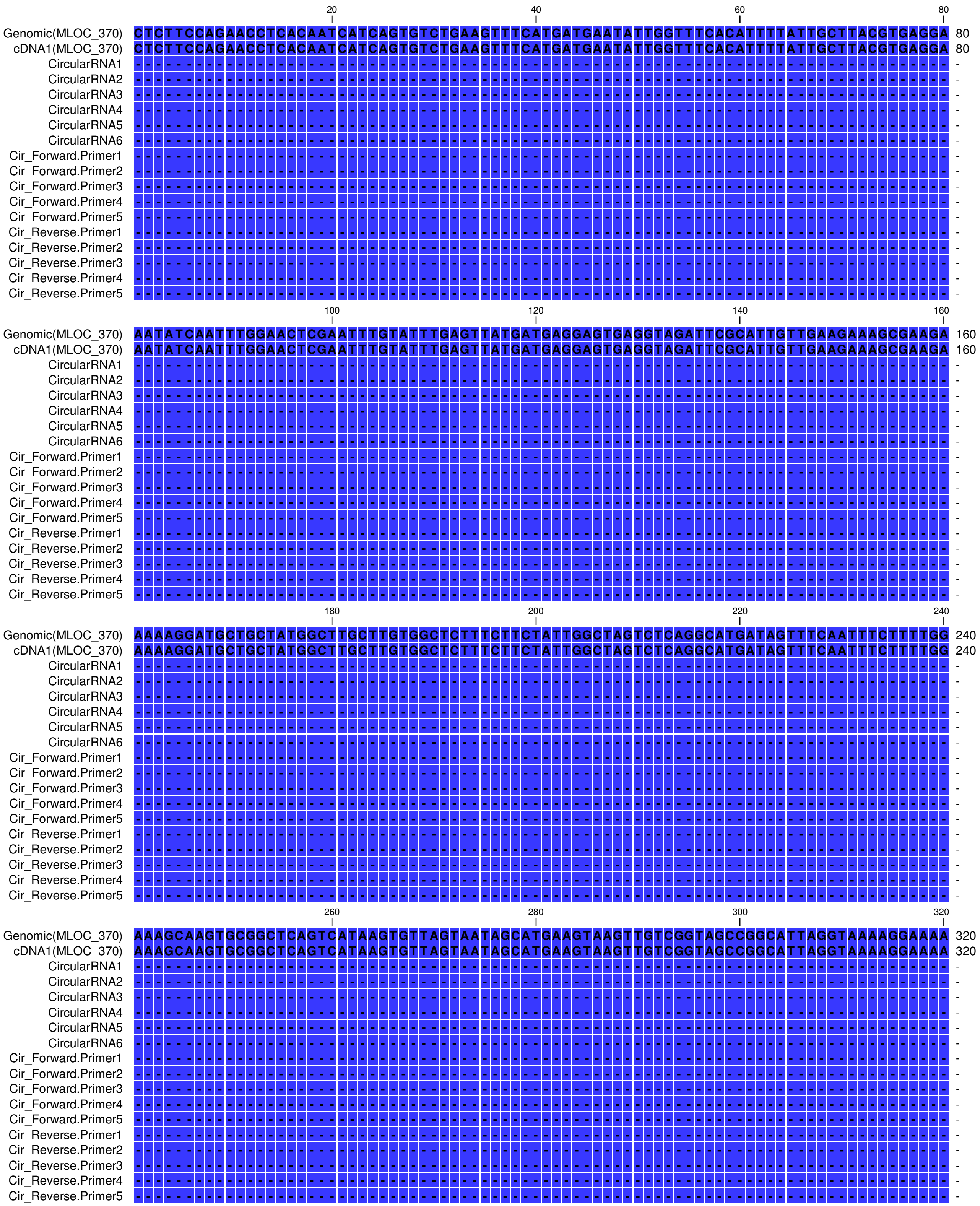

20 40 60 80
Genomic(MLOC_370) CTCTTCCAGAACCTCACAATCATCAGTGTCTGAAGTTTCATGATGAATATTGGTTTCACATTTTATTGCTTACGTGAGGA 80
cDNA1(MLOC_370) CTCTTCCAGAACCTCACAATCATCAGTGTCTGAAGTTTCATGATGAATATTGGTTTCACATTTTATTGCTTACGTGAGGA 80
CircularRNA1 -
CircularRNA2 -
CircularRNA3 -
CircularRNA4 -
CircularRNA5 -
CircularRNA6 -
Cir_Forward.Primer1 -
Cir_Forward.Primer2 -
Cir_Forward.Primer3 -
Cir_Forward.Primer4 -
Cir_Forward.Primer5 -
Cir_Reverse.Primer1 -
Cir_Reverse.Primer2 -
Cir_Reverse.Primer3 -
Cir_Reverse.Primer4 -
Cir_Reverse.Primer5 -
100 120 140 160
Genomic(MLOC_370) AATATCAATTTGGAACTCGAATTTGTATTTGAGTTATGATGAGGAGTGAGGTAGATTCGCATTGTTGAAGAAAGCGAAGA 160
cDNA1(MLOC_370) AATATCAATTTGGAACTCGAATTTGTATTTGAGTTATGATGAGGAGTGAGGTAGATTCGCATTGTTGAAGAAAGCGAAGA 160
CircularRNA1 -
CircularRNA2 -
CircularRNA3 -
CircularRNA4 -
CircularRNA5 -
CircularRNA6 -
Cir_Forward.Primer1 -
Cir_Forward.Primer2 -
Cir_Forward.Primer3 -
Cir_Forward.Primer4 -
Cir_Forward.Primer5 -
Cir_Reverse.Primer1 -
Cir_Reverse.Primer2 -
Cir_Reverse.Primer3 -
Cir_Reverse.Primer4 -
Cir_Reverse.Primer5 -
180 200 220 240
Genomic(MLOC_370) AAAAGGATGCTGCTATGGCTTGCTTGTGGCTCTTTCTTCTATTGGCTAGTCTCAGGCATGATAGTTTCAATTTCTTTTGG 240
cDNA1(MLOC_370) AAAAGGATGCTGCTATGGCTTGCTTGTGGCTCTTTCTTCTATTGGCTAGTCTCAGGCATGATAGTTTCAATTTCTTTTGG 240
CircularRNA1 -
CircularRNA2 -
CircularRNA3 -
CircularRNA4 -
CircularRNA5 -
CircularRNA6 -
Cir_Forward.Primer1 -
Cir_Forward.Primer2 -
Cir_Forward.Primer3 -
Cir_Forward.Primer4 -
Cir_Forward.Primer5 -
Cir_Reverse.Primer1 -
Cir_Reverse.Primer2 -
Cir_Reverse.Primer3 -
Cir_Reverse.Primer4 -
Cir_Reverse.Primer5 -
260 280 300 320
Genomic(MLOC_370) AAAGCAAGTGCGGCTCAGTCATAAGTGTTAGTAATAGCATGAAGTAAGTTGTCGGTAGCCGGCATTAGGTAAAAGGAAAA 320
cDNA1(MLOC_370) AAAGCAAGTGCGGCTCAGTCATAAGTGTTAGTAATAGCATGAAGTAAGTTGTCGGTAGCCGGCATTAGGTAAAAGGAAAA 320
CircularRNA1 -
CircularRNA2 -
CircularRNA3 -
CircularRNA4 -
CircularRNA5 -
CircularRNA6 -
Cir_Forward.Primer1 -
Cir_Forward.Primer2 -
Cir_Forward.Primer3 -
Cir_Forward.Primer4 -
Cir_Forward.Primer5 -
Cir_Reverse.Primer1 -
Cir_Reverse.Primer2 -
Cir_Reverse.Primer3 -
Cir_Reverse.Primer4 -
Cir_Reverse.Primer5 -

```
                                                                            340                 360                 380                 400
                                                                              |                   |                   |                   |
Genomic(MLOC_370)  GCTTCTCAAAAGCATCCTGAAATATCATAGAATGGTGATCGAGATCACCTTCACGGGAATCATCCTTTGCAAATTGGGGG 400
cDNA1(MLOC_370)    GCTTCTCAAAAGCATCCTGAAATATCATAGAATGGTGATCGAGATCACCTTCACGGGAATCATCCTTTGCAAATTGGGGG 400
CircularRNA1       -------------------------------------------------------------------------------- -
CircularRNA2       -------------------------------------------------------------------------------- -
CircularRNA3       -------------------------------------------------------------------------------- -
CircularRNA4       -------------------------------------------------------------------------------- -
CircularRNA5       -------------------------------------------------------------------------------- -
CircularRNA6       -------------------------------------------------------------------------------- -
Cir_Forward.Primer1 -------------------------------------------------------------------------------- -
Cir_Forward.Primer2 -------------------------------------------------------------------------------- -
Cir_Forward.Primer3 -------------------------------------------------------------------------------- -
Cir_Forward.Primer4 -------------------------------------------------------------------------------- -
Cir_Forward.Primer5 -------------------------------------------------------------------------------- -
Cir_Reverse.Primer1 -------------------------------------------------------------------------------- -
Cir_Reverse.Primer2 -------------------------------------------------------------------------------- -
Cir_Reverse.Primer3 -------------------------------------------------------------------------------- -
Cir_Reverse.Primer4 -------------------------------------------------------------------------------- -
Cir_Reverse.Primer5 -------------------------------------------------------------------------------- -

                                                                            420                 440                 460                 480
                                                                              |                   |                   |                   |
Genomic(MLOC_370)  AAGGGAACCAATCAAGTCACCAAGAAGTTCCCGAAACTTGGTTTAGTATTAAGGTTCTTCTCTTCCAGCGTTTACTATTC 480
cDNA1(MLOC_370)    AAGGGAACCAATCAAGTCACCAAGAAGTTCCCGAAACTTGGTTTAGTATTAAGGTTCTTCTCTTCCAGCGTTTACTATTC 480
CircularRNA1       -------------------------------------------------------------------------------- -
CircularRNA2       -------------------------------------------------------------------------------- -
CircularRNA3       -------------------------------------------------------------------------------- -
CircularRNA4       -------------------------------------------------------------------------------- -
CircularRNA5       -------------------------------------------------------------------------------- -
CircularRNA6       -------------------------------------------------------------------------------- -
Cir_Forward.Primer1 -------------------------------------------------------------------------------- -
Cir_Forward.Primer2 -------------------------------------------------------------------------------- -
Cir_Forward.Primer3 -------------------------------------------------------------------------------- -
Cir_Forward.Primer4 -------------------------------------------------------------------------------- -
Cir_Forward.Primer5 -------------------------------------------------------------------------------- -
Cir_Reverse.Primer1 -------------------------------------------------------------------------------- -
Cir_Reverse.Primer2 -------------------------------------------------------------------------------- -
Cir_Reverse.Primer3 -------------------------------------------------------------------------------- -
Cir_Reverse.Primer4 -------------------------------------------------------------------------------- -
Cir_Reverse.Primer5 -------------------------------------------------------------------------------- -

                                                                            500                 520                 540                 560
                                                                              |                   |                   |                   |
Genomic(MLOC_370)  AAGTTCTTCTCTTCCAGCCCCCTGGCCCCTCTCTGATAAGGAAAGTTTTCAAAACGAAAAGAAAATGACAAATCTGGTTC 560
cDNA1(MLOC_370)    AAGTTCTTCTCTTCCAGCCCCCTGGCCCCTCTCTGATAAGGAAAGTTTTCAAAACGAAAAGAAAATGACAAATCTGGTTC 560
CircularRNA1       -------------------------------------------------------------------------------- -
CircularRNA2       -------------------------------------------------------------------------------- -
CircularRNA3       -------------------------------------------------------------------------------- -
CircularRNA4       -------------------------------------------------------------------------------- -
CircularRNA5       -------------------------------------------------------------------------------- -
CircularRNA6       -------------------------------------------------------------------------------- -
Cir_Forward.Primer1 -------------------------------------------------------------------------------- -
Cir_Forward.Primer2 -------------------------------------------------------------------------------- -
Cir_Forward.Primer3 -------------------------------------------------------------------------------- -
Cir_Forward.Primer4 -------------------------------------------------------------------------------- -
Cir_Forward.Primer5 -------------------------------------------------------------------------------- -
Cir_Reverse.Primer1 -------------------------------------------------------------------------------- -
Cir_Reverse.Primer2 -------------------------------------------------------------------------------- -
Cir_Reverse.Primer3 -------------------------------------------------------------------------------- -
Cir_Reverse.Primer4 -------------------------------------------------------------------------------- -
Cir_Reverse.Primer5 -------------------------------------------------------------------------------- -

                                                                            580                 600                 620                 640
                                                                              |                   |                   |                   |
Genomic(MLOC_370)  GATGGCTCTTCTCCACTACTCGCTTTACTACTTTCTATTTTTTCTTATGTATTAAGTTTCCCTTAATATATAATTTTATA 640
cDNA1(MLOC_370)    GATGGCTCTTCTCCACTACTCGCTTTACTACTTTCTATTTTTTCTTATGTATTAAGTTTCCCTTAATATATAATTTTATA 640
CircularRNA1       -------------------------------------------------------------------------------- -
CircularRNA2       -------------------------------------------------------------------------------- -
CircularRNA3       -------------------------------------------------------------------------------- -
CircularRNA4       -------------------------------------------------------------------------------- -
CircularRNA5       -------------------------------------------------------------------------------- -
CircularRNA6       -------------------------------------------------------------------------------- -
Cir_Forward.Primer1 -------------------------------------------------------------------------------- -
Cir_Forward.Primer2 -------------------------------------------------------------------------------- -
Cir_Forward.Primer3 -------------------------------------------------------------------------------- -
Cir_Forward.Primer4 -------------------------------------------------------------------------------- -
Cir_Forward.Primer5 -------------------------------------------------------------------------------- -
Cir_Reverse.Primer1 -------------------------------------------------------------------------------- -
Cir_Reverse.Primer2 -------------------------------------------------------------------------------- -
Cir_Reverse.Primer3 -------------------------------------------------------------------------------- -
Cir_Reverse.Primer4 -------------------------------------------------------------------------------- -
Cir_Reverse.Primer5 -------------------------------------------------------------------------------- -
```

```
                                                                                   660                 680                 700                 720
                                                                                     |                   |                   |                   |
Genomic(MLOC_370)      TTACTTTCGATTTGTATATTTTTATTCCTTTGGCGTTTTATATTAATAGTAATAGCCGTTTTCAATCTTTTTTGTTCGTG 720
cDNA1(MLOC_370)        TTACTTTCGATTTGTATATTTTTATTCCTTTGGCGTTTTATATTAATAGTAATAGCCGTTTTCAATCTTTTTTGTTCGTG 720
CircularRNA1           -------------------------------------------------------------------------------- -
CircularRNA2           -------------------------------------------------------------------------------- -
CircularRNA3           -------------------------------------------------------------------------------- -
CircularRNA4           -------------------------------------------------------------------------------- -
CircularRNA5           -------------------------------------------------------------------------------- -
CircularRNA6           -------------------------------------------------------------------------------- -
Cir_Forward.Primer1    -------------------------------------------------------------------------------- -
Cir_Forward.Primer2    -------------------------------------------------------------------------------- -
Cir_Forward.Primer3    -------------------------------------------------------------------------------- -
Cir_Forward.Primer4    -------------------------------------------------------------------------------- -
Cir_Forward.Primer5    -------------------------------------------------------------------------------- -
Cir_Reverse.Primer1    -------------------------------------------------------------------------------- -
Cir_Reverse.Primer2    -------------------------------------------------------------------------------- -
Cir_Reverse.Primer3    -------------------------------------------------------------------------------- -
Cir_Reverse.Primer4    -------------------------------------------------------------------------------- -
Cir_Reverse.Primer5    -------------------------------------------------------------------------------- -

                                                                                   740                 760                 780                 800
                                                                                     |                   |                   |                   |
Genomic(MLOC_370)      CTTCGTCGGCTCCTCCTTTCTGATCACTCTCCCGCCGGAGATTCAAGACCCCCAGGCTCTAGCTCATTTAGCAGGGCTAA 800
cDNA1(MLOC_370)        CTTCGTCGGCTCCTCCTTTCTGATCACTCTCCCGCCGGAGATTCAAGACCCCCAGGCTCTAGCTCATTTAGCAGGGCTAA 800
CircularRNA1           -------------------------------------------------------------------------------- -
CircularRNA2           -------------------------------------------------------------------------------- -
CircularRNA3           -------------------------------------------------------------------------------- -
CircularRNA4           -------------------------------------------------------------------------------- -
CircularRNA5           -------------------------------------------------------------------------------- -
CircularRNA6           -------------------------------------------------------------------------------- -
Cir_Forward.Primer1    -------------------------------------------------------------------------------- -
Cir_Forward.Primer2    -------------------------------------------------------------------------------- -
Cir_Forward.Primer3    -------------------------------------------------------------------------------- -
Cir_Forward.Primer4    -------------------------------------------------------------------------------- -
Cir_Forward.Primer5    -------------------------------------------------------------------------------- -
Cir_Reverse.Primer1    -------------------------------------------------------------------------------- -
Cir_Reverse.Primer2    -------------------------------------------------------------------------------- -
Cir_Reverse.Primer3    -------------------------------------------------------------------------------- -
Cir_Reverse.Primer4    -------------------------------------------------------------------------------- -
Cir_Reverse.Primer5    -------------------------------------------------------------------------------- -

                                                                                   820                 840                 860                 880
                                                                                     |                   |                   |                   |
Genomic(MLOC_370)      ACTTCTATCTGAGTCTTTACGAGCAGGATCCGGGATGGGTTACGTTCATTCAGAACGAGCTGTTTCACAATACCCCCCTG 880
cDNA1(MLOC_370)        ACTTCTATCTGAGTCTTTACGAGCAGGATCCGGGATGGGTTACGTTCATTCAGAACGAGCTGTTTCACAATACCCCCCTG 880
CircularRNA1           -------------------------------------------------------------------------------- -
CircularRNA2           -------------------------------------------------------------------------------- -
CircularRNA3           -------------------------------------------------------------------------------- -
CircularRNA4           -------------------------------------------------------------------------------- -
CircularRNA5           -------------------------------------------------------------------------------- -
CircularRNA6           -------------------------------------------------------------------------------- -
Cir_Forward.Primer1    -------------------------------------------------------------------------------- -
Cir_Forward.Primer2    -------------------------------------------------------------------------------- -
Cir_Forward.Primer3    -------------------------------------------------------------------------------- -
Cir_Forward.Primer4    -------------------------------------------------------------------------------- -
Cir_Forward.Primer5    -------------------------------------------------------------------------------- -
Cir_Reverse.Primer1    -------------------------------------------------------------------------------- -
Cir_Reverse.Primer2    -------------------------------------------------------------------------------- -
Cir_Reverse.Primer3    -------------------------------------------------------------------------------- -
Cir_Reverse.Primer4    -------------------------------------------------------------------------------- -
Cir_Reverse.Primer5    -------------------------------------------------------------------------------- -

                                                                                   900                 920                 940                 960
                                                                                     |                   |                   |                   |
Genomic(MLOC_370)      GAAGACATACCTGGGCGGCTTAAGCTCTTCCTAATGGAAGAAAAGCTCTCTTCTATGCGACAAGATGTCATTCAGGAATT 960
cDNA1(MLOC_370)        GAAGACATACCTGGGCGGCTTAAGCTCTTCCTAATGGAAGAAAAGCTCTCTTCTATGCGACAAGATGTCATTCAGGAATT 960
CircularRNA1           -------------------------------------------------------------------------------- -
CircularRNA2           -------------------------------------------------------------------------------- -
CircularRNA3           -------------------------------------------------------------------------------- -
CircularRNA4           -------------------------------------------------------------------------------- -
CircularRNA5           -------------------------------------------------------------------------------- -
CircularRNA6           -------------------------------------------------------------------------------- -
Cir_Forward.Primer1    -------------------------------------------------------------------------------- -
Cir_Forward.Primer2    -------------------------------------------------------------------------------- -
Cir_Forward.Primer3    -------------------------------------------------------------------------------- -
Cir_Forward.Primer4    -------------------------------------------------------------------------------- -
Cir_Forward.Primer5    -------------------------------------------------------------------------------- -
Cir_Reverse.Primer1    -------------------------------------------------------------------------------- -
Cir_Reverse.Primer2    -------------------------------------------------------------------------------- -
Cir_Reverse.Primer3    -------------------------------------------------------------------------------- -
Cir_Reverse.Primer4    -------------------------------------------------------------------------------- -
Cir_Reverse.Primer5    -------------------------------------------------------------------------------- -
```

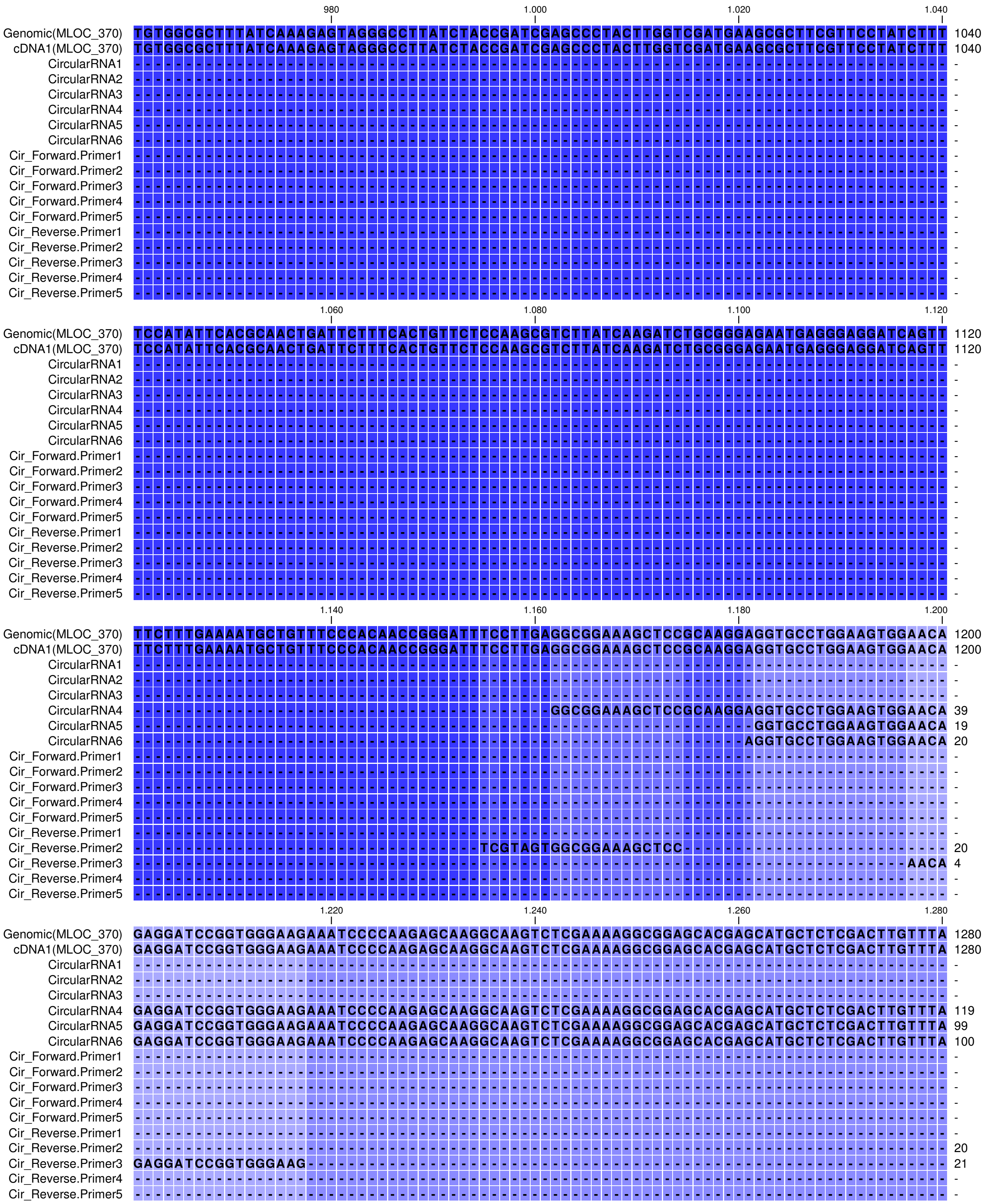

980
1.000
1.020
1.040
Genomic(MLOC_370) TGTGGCGCTTTATCAAAGAGTAGGGCCTTATCTACCGATCGAGCCCTACTTGGTCGATGAAGCGCTTCGTTCCTATCTTT 1040
cDNA1(MLOC_370) TGTGGCGCTTTATCAAAGAGTAGGGCCTTATCTACCGATCGAGCCCTACTTGGTCGATGAAGCGCTTCGTTCCTATCTTT 1040
CircularRNA1
CircularRNA2
CircularRNA3
CircularRNA4
CircularRNA5
CircularRNA6
Cir_Forward.Primer1
Cir_Forward.Primer2
Cir_Forward.Primer3
Cir_Forward.Primer4
Cir_Forward.Primer5
Cir_Reverse.Primer1
Cir_Reverse.Primer2
Cir_Reverse.Primer3
Cir_Reverse.Primer4
Cir_Reverse.Primer5
1.060
1.080
1.100
1.120
Genomic(MLOC_370) TCCATATTCACGCAACTGATTCTTTCACTGTTCTCCAAGCGTCTTATCAAGATCTGCGGGAGAATGAGGGAGGATCAGTT 1120
cDNA1(MLOC_370) TCCATATTCACGCAACTGATTCTTTCACTGTTCTCCAAGCGTCTTATCAAGATCTGCGGGAGAATGAGGGAGGATCAGTT 1120
1.140
1.160
1.180
1.200
Genomic(MLOC_370) TTCTTTGAAAATGCTGTTTCCCACAACCGGGATTTCCTTGAGGCGGAAAGCTCCGCAAGGAGGTGCCTGGAAGTGGAACA 1200
cDNA1(MLOC_370) TTCTTTGAAAATGCTGTTTCCCACAACCGGGATTTCCTTGAGGCGGAAAGCTCCGCAAGGAGGTGCCTGGAAGTGGAACA 1200
CircularRNA4 GGCGGAAAGCTCCGCAAGGAGGTGCCTGGAAGTGGAACA 39
CircularRNA5 GGTGCCTGGAAGTGGAACA 19
CircularRNA6 AGGTGCCTGGAAGTGGAACA 20
Cir_Reverse.Primer2 TCGTAGTGGCGGAAAGCTCC 20
Cir_Reverse.Primer3 AACA 4
1.220
1.240
1.260
1.280
Genomic(MLOC_370) GAGGATCCGGTGGGAAGAAATCCCCAAGAGCAAGGCAAGTCTCGAAAAGGCGGAGCACGAGCATGCTCTCGACTTGTTTA 1280
cDNA1(MLOC_370) GAGGATCCGGTGGGAAGAAATCCCCAAGAGCAAGGCAAGTCTCGAAAAGGCGGAGCACGAGCATGCTCTCGACTTGTTTA 1280
CircularRNA4 GAGGATCCGGTGGGAAGAAATCCCCAAGAGCAAGGCAAGTCTCGAAAAGGCGGAGCACGAGCATGCTCTCGACTTGTTTA 119
CircularRNA5 GAGGATCCGGTGGGAAGAAATCCCCAAGAGCAAGGCAAGTCTCGAAAAGGCGGAGCACGAGCATGCTCTCGACTTGTTTA 99
CircularRNA6 GAGGATCCGGTGGGAAGAAATCCCCAAGAGCAAGGCAAGTCTCGAAAAGGCGGAGCACGAGCATGCTCTCGACTTGTTTA 100
Cir_Reverse.Primer2 20
Cir_Reverse.Primer3 GAGGATCCGGTGGGAAG 21

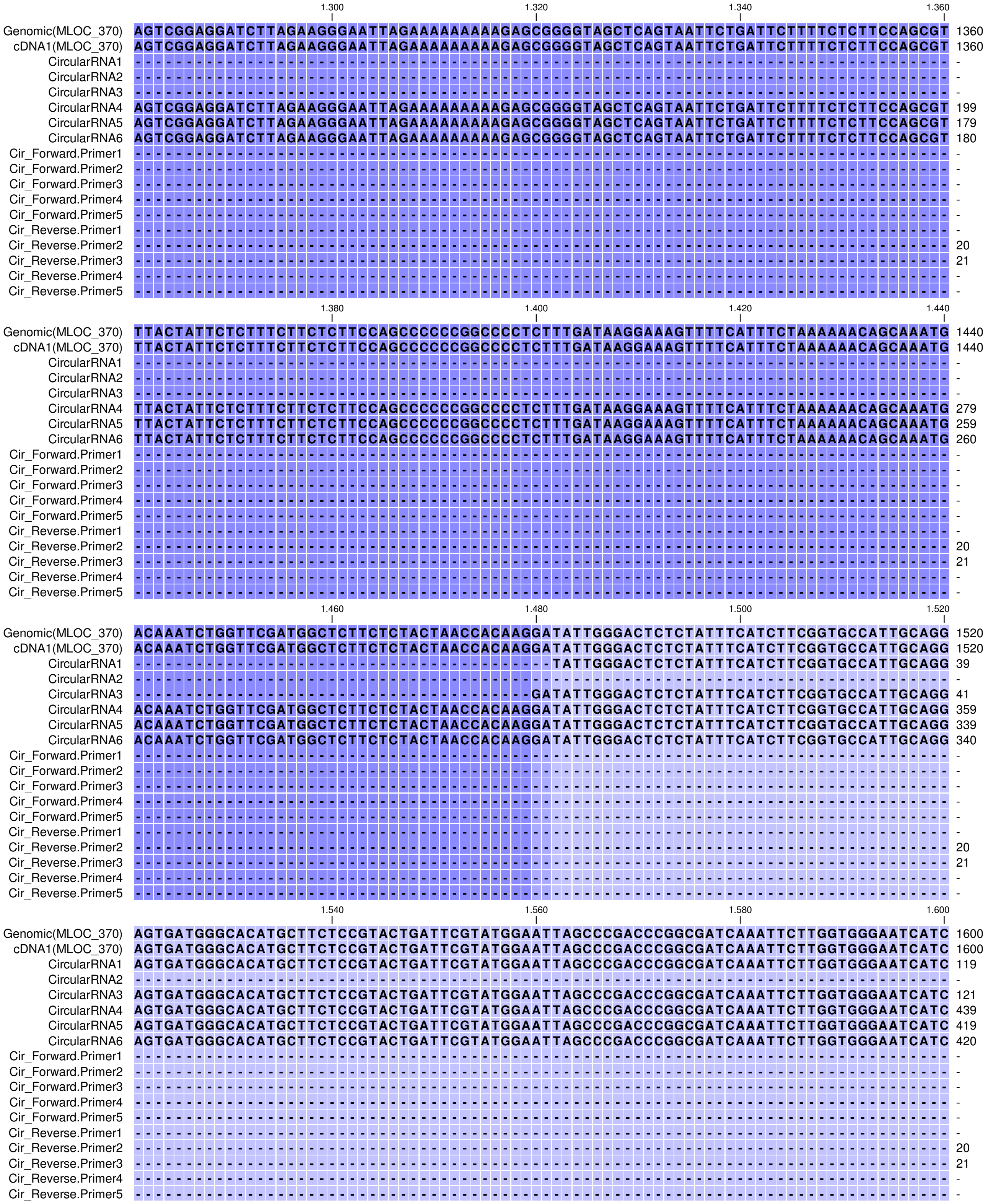
1.300 1.320 1.340 1.360
Genomic(MLOC_370) AGTCGGAGGATCTTAGAAGGGAATTAGAAAAAAAAAAGAGCGGGGTAGCTCAGTAATTCTGATTCTTTTCTCTTCCAGCGT 1360
cDNA1(MLOC_370) AGTCGGAGGATCTTAGAAGGGAATTAGAAAAAAAAAAGAGCGGGGTAGCTCAGTAATTCTGATTCTTTTCTCTTCCAGCGT 1360
CircularRNA1 -
CircularRNA2 -
CircularRNA3 -
CircularRNA4 AGTCGGAGGATCTTAGAAGGGAATTAGAAAAAAAAAAGAGCGGGGTAGCTCAGTAATTCTGATTCTTTTCTCTTCCAGCGT 199
CircularRNA5 AGTCGGAGGATCTTAGAAGGGAATTAGAAAAAAAAAAGAGCGGGGTAGCTCAGTAATTCTGATTCTTTTCTCTTCCAGCGT 179
CircularRNA6 AGTCGGAGGATCTTAGAAGGGAATTAGAAAAAAAAAAGAGCGGGGTAGCTCAGTAATTCTGATTCTTTTCTCTTCCAGCGT 180
Cir_Forward.Primer1 -
Cir_Forward.Primer2 -
Cir_Forward.Primer3 -
Cir_Forward.Primer4 -
Cir_Forward.Primer5 -
Cir_Reverse.Primer1 -
Cir_Reverse.Primer2 20
Cir_Reverse.Primer3 21
Cir_Reverse.Primer4 -
Cir_Reverse.Primer5 -
1.380 1.400 1.420 1.440
Genomic(MLOC_370) TTACTATTCTCTTTCTTCTCTTCCAGCCCCCCGGCCCCTCTTTGATAAGGAAAGTTTTCATTTCTAAAAAACAGCAAATG 1440
cDNA1(MLOC_370) TTACTATTCTCTTTCTTCTCTTCCAGCCCCCCGGCCCCTCTTTGATAAGGAAAGTTTTCATTTCTAAAAAACAGCAAATG 1440
CircularRNA1 -
CircularRNA2 -
CircularRNA3 -
CircularRNA4 TTACTATTCTCTTTCTTCTCTTCCAGCCCCCCGGCCCCTCTTTGATAAGGAAAGTTTTCATTTCTAAAAAACAGCAAATG 279
CircularRNA5 TTACTATTCTCTTTCTTCTCTTCCAGCCCCCCGGCCCCTCTTTGATAAGGAAAGTTTTCATTTCTAAAAAACAGCAAATG 259
CircularRNA6 TTACTATTCTCTTTCTTCTCTTCCAGCCCCCCGGCCCCTCTTTGATAAGGAAAGTTTTCATTTCTAAAAAACAGCAAATG 260
Cir_Forward.Primer1 -
Cir_Forward.Primer2 -
Cir_Forward.Primer3 -
Cir_Forward.Primer4 -
Cir_Forward.Primer5 -
Cir_Reverse.Primer1 -
Cir_Reverse.Primer2 20
Cir_Reverse.Primer3 21
Cir_Reverse.Primer4 -
Cir_Reverse.Primer5 -
1.460 1.480 1.500 1.520
Genomic(MLOC_370) ACAAATCTGGTTCGATGGCTCTTCTCTACTAACCACAAGGATATTGGGACTCTCTATTTCATCTTCGGTGCCATTGCAGG 1520
cDNA1(MLOC_370) ACAAATCTGGTTCGATGGCTCTTCTCTACTAACCACAAGGATATTGGGACTCTCTATTTCATCTTCGGTGCCATTGCAGG 1520
CircularRNA1 TATTGGGACTCTCTATTTCATCTTCGGTGCCATTGCAGG 39
CircularRNA2 -
CircularRNA3 GATATTGGGACTCTCTATTTCATCTTCGGTGCCATTGCAGG 41
CircularRNA4 ACAAATCTGGTTCGATGGCTCTTCTCTACTAACCACAAGGATATTGGGACTCTCTATTTCATCTTCGGTGCCATTGCAGG 359
CircularRNA5 ACAAATCTGGTTCGATGGCTCTTCTCTACTAACCACAAGGATATTGGGACTCTCTATTTCATCTTCGGTGCCATTGCAGG 339
CircularRNA6 ACAAATCTGGTTCGATGGCTCTTCTCTACTAACCACAAGGATATTGGGACTCTCTATTTCATCTTCGGTGCCATTGCAGG 340
Cir_Forward.Primer1 -
Cir_Forward.Primer2 -
Cir_Forward.Primer3 -
Cir_Forward.Primer4 -
Cir_Forward.Primer5 -
Cir_Reverse.Primer1 -
Cir_Reverse.Primer2 20
Cir_Reverse.Primer3 21
Cir_Reverse.Primer4 -
Cir_Reverse.Primer5 -
1.540 1.560 1.580 1.600
Genomic(MLOC_370) AGTGATGGGCACATGCTTCTCCGTACTGATTCGTATGGAATTAGCCCGACCCGGCGATCAAATTCTTGGTGGGAATCATC 1600
cDNA1(MLOC_370) AGTGATGGGCACATGCTTCTCCGTACTGATTCGTATGGAATTAGCCCGACCCGGCGATCAAATTCTTGGTGGGAATCATC 1600
CircularRNA1 AGTGATGGGCACATGCTTCTCCGTACTGATTCGTATGGAATTAGCCCGACCCGGCGATCAAATTCTTGGTGGGAATCATC 119
CircularRNA2 -
CircularRNA3 AGTGATGGGCACATGCTTCTCCGTACTGATTCGTATGGAATTAGCCCGACCCGGCGATCAAATTCTTGGTGGGAATCATC 121
CircularRNA4 AGTGATGGGCACATGCTTCTCCGTACTGATTCGTATGGAATTAGCCCGACCCGGCGATCAAATTCTTGGTGGGAATCATC 439
CircularRNA5 AGTGATGGGCACATGCTTCTCCGTACTGATTCGTATGGAATTAGCCCGACCCGGCGATCAAATTCTTGGTGGGAATCATC 419
CircularRNA6 AGTGATGGGCACATGCTTCTCCGTACTGATTCGTATGGAATTAGCCCGACCCGGCGATCAAATTCTTGGTGGGAATCATC 420
Cir_Forward.Primer1 -
Cir_Forward.Primer2 -
Cir_Forward.Primer3 -
Cir_Forward.Primer4 -
Cir_Forward.Primer5 -
Cir_Reverse.Primer1 -
Cir_Reverse.Primer2 20
Cir_Reverse.Primer3 21
Cir_Reverse.Primer4 -
Cir_Reverse.Primer5 -

```
                                  1.620               1.640               1.660               1.680
Genomic(MLOC_370)  AACTTTATAATGTTTTAATAACGGCTCACGCTTTTTTAATGATCTTTTTTATGGTTATGCCGGCGATGATAGGTGGATTT 1680
cDNA1(MLOC_370)    AACTTTATAATGTTTTAATAACGGCTCACGCTTTTTTAATGATCTTTTTTATGGTTATGCCGGCGATGATAGGTGGATTT 1680
CircularRNA1       AACTTTATAATGTTTTAATAACGGCTCACGCTTTTTTAATGATCTTTTTTATGGTTATGCCGGCGATGATAGGTGGATTT 199
CircularRNA2       ----------------------GGCTCACGCTTTTTTAATGATCTTTTTTATGGTTATGCCGGCGATGATAGGTGGATTT 58
CircularRNA3       AACTTTATAATGTTTTAATAACGGCTCACGCTTTTTTAATGATCTTTTTTATGGTTATGCCGGCGATGATAGGTGGATTT 201
CircularRNA4       AACTTTATAATGTTTTAATAACGGCTCACGCTTTTTTAATGATCTTTTTTATGGTTATGCCGGCGATGATAGGTGGATTT 519
CircularRNA5       AACTTTATAATGTTTTAATAACGGCTCACGCTTTTTTAATGATCTTTTTTATGGTTATGCCGGCGATGATAGGTGGATTT 499
CircularRNA6       AACTTTATAATGTTTTAATAACGGCTCACGCTTTTTTAATGATCTTTTTTATGGTTATGCCGGCGATGATAGGTGGATTT 500
Cir_Forward.Primer1 -------------------------------------------------------------------------------- -
Cir_Forward.Primer2 -------------------------------------------------------------------------------- -
Cir_Forward.Primer3 -------------------------------------------------------------------------------- -
Cir_Forward.Primer4 -------------------------------------------------------------------------------- -
Cir_Forward.Primer5 -------------------------------------------------------------------------------- -
Cir_Reverse.Primer1 -------------------------------------------------------------------------------- -
Cir_Reverse.Primer2 -------------------------------------------------------------------------------- 20
Cir_Reverse.Primer3 -------------------------------------------------------------------------------- 21
Cir_Reverse.Primer4 -------------------------------------------------------------------------------- -
Cir_Reverse.Primer5 -------------------------------------------------------------------------------- -

                                  1.700               1.720               1.740               1.760
Genomic(MLOC_370)  GGTAATTGGTTTGTTCCGATTCTGATAGGTGCACCTGACATGGCATTTCCACGATTAAATAATATATCATTCTGGTTGTT 1760
cDNA1(MLOC_370)    GGTAATTGGTTTGTTCCGATTCTGATAGGTGCACCTGACATGGCATTTCCACGATTAAATAATATATCATTCTGGTTGTT 1760
CircularRNA1       GGTAATTGGTTTGTTCCGATTCTGATAGGTGCACCTGACATGGCATTTCCACGATTAAATAATATATCATTCTGGTTGTT 279
CircularRNA2       GGTAATTGGTTTGTTCCGATTCTGATAGGTGCACCTGACATGGCATTTCCACGATTAAATAATATATCATTCTGGTTGTT 138
CircularRNA3       GGTAATTGGTTTGTTCCGATTCTGATAGGTGCACCTGACATGGCATTTCCACGATTAAATAATATATCATTCTGGTTGTT 281
CircularRNA4       GGTAATTGGTTTGTTCCGATTCTGATAGGTGCACCTGACATGGCATTTCCACGATTAAATAATATATCATTCTGGTTGTT 599
CircularRNA5       GGTAATTGGTTTGTTCCGATTCTGATAGGTGCACCTGACATGGCATTTCCACGATTAAATAATATATCATTCTGGTTGTT 579
CircularRNA6       GGTAATTGGTTTGTTCCGATTCTGATAGGTGCACCTGACATGGCATTTCCACGATTAAATAATATATCATTCTGGTTGTT 580
Cir_Forward.Primer1 -------------------------------------------------------------------------------- -
Cir_Forward.Primer2 -------------------------------------------------------------------------------- -
Cir_Forward.Primer3 -------------------------------------------------------------------------------- -
Cir_Forward.Primer4 -------------------------------------------------------------------------------- -
Cir_Forward.Primer5 -------------------------------------------------------------------------------- -
Cir_Reverse.Primer1 ------TGGTTTGTTCCGATTCTGATAGG--------------------------------------------------- 23
Cir_Reverse.Primer2 -------------------------------------------------------------------------------- 20
Cir_Reverse.Primer3 -------------------------------------------------------------------------------- 21
Cir_Reverse.Primer4 -------------------------------------------------------------------------------- -
Cir_Reverse.Primer5 -------------------------------------------------------------------------------- -

                                  1.780               1.800               1.820               1.840
Genomic(MLOC_370)  GCCACCAAGTCTCTTGCTCCTATTAAGCTCAGCCTTAGTAGAAGTGGGCAGCGGCACTGGGTGGACAGTCTATCCGCCCT 1840
cDNA1(MLOC_370)    GCCACCAAGTCTCTTGCTCCTATTAAGCTCAGCCTTAGTAGAAGTGGGCAGCGGCACTGGGTGGACAGTCTATCCGCCCT 1840
CircularRNA1       GCCACCAAGTCTCTTGCTCCTATTAAGCTCAGCCTTAGTAGAAGTGGGCAGCGGCACTGGGTGGACAG------------ 347
CircularRNA2       GCCACCAAGTCTCTTGCTCCTATTAAGCTCAGCCTTAGTAGAAGTGGGCAGCGGCACTGGGTGGACAGTCTATCCGCCCT 218
CircularRNA3       GCCACCAAGTCTCTTGCTCCTATTAAGCTCAGCCTTAGTAGAAGTGGGCAGCGGCACTGGGTGGACAGTCTATCCGCCCT 361
CircularRNA4       GCCACCAAGTCTCTTGCTCCTATTAAGCTCAGCCTTAGTAGAAGTGGGCAGCGGCACTGGGTGGACAGTCTATCCGCCCT 679
CircularRNA5       GCCACCAAGTCTCTTGCTCCTATTAAGCTCAGCCTTAGTAGAAGTGGGCAGCGGCACTGGGTGGACAGTCTATCCGCCCT 659
CircularRNA6       GCCACCAAGTCTCTTGCTCCTATTAAGCTCAGCCTTAGTAGAAGTGGGCAGCGGCACTGGGTGGACAGTCTATCCGCCCT 660
Cir_Forward.Primer1 ------------------------------------------------------CACTGGGTGGACAGTATTGGGA---- 22
Cir_Forward.Primer2 -------------------------------------------------------------------------------- -
Cir_Forward.Primer3 -------------------------------------------------------------------------------- -
Cir_Forward.Primer4 -------------------------------------------------------------------------------- -
Cir_Forward.Primer5 -------------------------------------------------------------------------------- -
Cir_Reverse.Primer1 -------------------------------------------------------------------------------- 23
Cir_Reverse.Primer2 -------------------------------------------------------------------------------- 20
Cir_Reverse.Primer3 -------------------------------------------------------------------------------- 21
Cir_Reverse.Primer4 -------------TTGCTCCTATTAAGCTCAGCCTTA------------------------------------------- 24
Cir_Reverse.Primer5 ------AAGTCTCTTGCTCCTATTAAGCTCAG------------------------------------------------ 26

                                  1.860               1.880               1.900               1.920
Genomic(MLOC_370)  TAAGTGGTATTACCAGCCATTCTGGAGGAGCAGTTGATTTAGCAATTTTTAGTCTTCATCTATCAGGTATTTCATCAATT 1920
cDNA1(MLOC_370)    TAAGTGGTATTACCAGCCATTCTGGAGGAGCAGTTGATTTAGCAATTTTTAGTCTTCATCTATCAGGTATTTCATCAATT 1920
CircularRNA1       -------------------------------------------------------------------------------- 347
CircularRNA2       TAAGTGGTATTACCAGCCATTCTGGAGGAGCAGTTGATTTAGCAATTTTTAGTCTTCATCTATCAGGTATTTCATCAATT 298
CircularRNA3       TAAGTGGTATTACCAGCCATTCTGGAGGAGCA------------------------------------------------ 393
CircularRNA4       TAAGTGGTATTACCAGCCATTCTGGAGGAGCAGTTGATTTAGCAATTTTTAGTCTTCATCTATCAGGTATTTCATCAATT 759
CircularRNA5       TAAGTGGTATTACCAGCCATTCTGGAGGAGCAGTTGATTTAGCAATTTTTAGTCTTCATCTATCAGGTATTTCATCAATT 739
CircularRNA6       TAAGTGGTATTACCAGCCATTCTGGAGGAGCAGTTGATTTAGCAATTTTTAGTCTTCATCTATCAGGTATTTCATCAATT 740
Cir_Forward.Primer1 -------------------------------------------------------------------------------- 22
Cir_Forward.Primer2 -------------------------------------------------------------------------------- -
Cir_Forward.Primer3 -------------------------------------------------------------------------------- -
Cir_Forward.Primer4 ---------------GCCATTCTGGAGGAGCAGATATT------------------------------------------ 23
Cir_Forward.Primer5 -------------------------------------------------------------------------------- -
Cir_Reverse.Primer1 -------------------------------------------------------------------------------- 23
Cir_Reverse.Primer2 -------------------------------------------------------------------------------- 20
Cir_Reverse.Primer3 -------------------------------------------------------------------------------- 21
Cir_Reverse.Primer4 -------------------------------------------------------------------------------- 24
Cir_Reverse.Primer5 -------------------------------------------------------------------------------- 26
```

```
                                          1.940               1.960               1.980               2.000
Genomic(MLOC_370)     TTAGGTTCTATCAATTTTATAACAACTATCTTCAACATGCGTGGACCTGGAATGACTATGCATAGATTACCACTTTTTGT 2000
cDNA1(MLOC_370)       TTAGGTTCTATCAATTTTATAACAACTATCTTCAACATGCGTGGACCTGGAATGACTATGCATAGATTACCACTTTTTGT 2000
CircularRNA1          -------------------------------------------------------------------------------- 347
CircularRNA2          TTAGGTTCTATCAATTTTATAACAACTATCTTCAACATGCGTGGACCTGGAATGACTATGCATAGATTACCA-------- 370
CircularRNA3          -------------------------------------------------------------------------------- 393
CircularRNA4          TTAGGTTCTATCAATTTTATAACAACTATCTTCAACATGCGTGGACCTGGAATGACTATGCATAGATTACCACTTTTTGT 839
CircularRNA5          TTAGGTTCTATCAATTTTATAACAACTATCTTCAACATGCGTGGACCTGGAATGACTATGCATAGATTACCACTTTTTGT 819
CircularRNA6          TTAGGTTCTATCAATTTTATAACAACTATCTTCAACATGCGTGGACCTGGAATGACTATGCATAGATTACCACTTTTTGT 820
Cir_Forward.Primer1   -------------------------------------------------------------------------------- 22
Cir_Forward.Primer2   -------------------------------------------------------------------------------- -
Cir_Forward.Primer3   -------------------------------------------------------------------------------- -
Cir_Forward.Primer4   -------------------------------------------------------------------------------- 23
Cir_Forward.Primer5   -----------------------------------------------------------GCATAGATTACCAGGCTCACG 21
Cir_Reverse.Primer1   -------------------------------------------------------------------------------- 23
Cir_Reverse.Primer2   -------------------------------------------------------------------------------- 20
Cir_Reverse.Primer3   -------------------------------------------------------------------------------- 21
Cir_Reverse.Primer4   -------------------------------------------------------------------------------- 24
Cir_Reverse.Primer5   -------------------------------------------------------------------------------- 26

                                          2.020               2.040               2.060               2.080
Genomic(MLOC_370)     GTGGTCCGTTCTAGTGACAGCATTCCTACTTTTATTATCACTTCCGGTACTGGCGGGGGCAATTACAATGTTATTAACCG 2080
cDNA1(MLOC_370)       GTGGTCCGTTCTAGTGACAGCATTCCTACTTTTATTATCACTTCCGGTACTGGCGGGGGCAATTACAATGTTATTAACCG 2080
CircularRNA1          -------------------------------------------------------------------------------- 347
CircularRNA2          -------------------------------------------------------------------------------- 370
CircularRNA3          -------------------------------------------------------------------------------- 393
CircularRNA4          GTGGTCCGTTCTAGTGACAGCATTCCTACTTTTATTATCACTTCCGGTACTGGCGGGGGCAATTACAATGTTATTAACCG 919
CircularRNA5          GTGGTCCGTTCTAGTGACAGCATTCCTACTTTTATTATCACTTCCGGTACTGGCGGGGGCAATTACAATGTTATTAACCG 899
CircularRNA6          GTGGTCCGTTCTAGTGACAGCATTCCTACTTTTATTATCACTTCCGGTACTGGCGGGGGCAATTACAATGTTATTAACCG 900
Cir_Forward.Primer1   -------------------------------------------------------------------------------- 22
Cir_Forward.Primer2   -------------------------------------------------------------------------------- -
Cir_Forward.Primer3   -------------------------------------------------------------------------------- -
Cir_Forward.Primer4   -------------------------------------------------------------------------------- 23
Cir_Forward.Primer5   CTT----------------------------------------------------------------------------- 24
Cir_Reverse.Primer1   -------------------------------------------------------------------------------- 23
Cir_Reverse.Primer2   -------------------------------------------------------------------------------- 20
Cir_Reverse.Primer3   -------------------------------------------------------------------------------- 21
Cir_Reverse.Primer4   -------------------------------------------------------------------------------- 24
Cir_Reverse.Primer5   -------------------------------------------------------------------------------- 26

                                          2.100               2.120               2.140               2.160
Genomic(MLOC_370)     ATCGAAACTTTAATACAACCTTTTTTGATCCTGCAGGAGGGGGAGACCCAATATTATACCAGCATCTCTTTTGGTTCTTC 2160
cDNA1(MLOC_370)       ATCGAAACTTTAATACAACCTTTTTTGATCCTGCAGGAGGGGGAGACCCAATATTATACCAGCATCTCTTTTGGTTCTTC 2160
CircularRNA1          -------------------------------------------------------------------------------- 347
CircularRNA2          -------------------------------------------------------------------------------- 370
CircularRNA3          -------------------------------------------------------------------------------- 393
CircularRNA4          ATCGAAACTTTAATACAACCTTTTTTGATCCTGCAGGAGGGGGAGACCCAATATTATACCAGCATCTCTTTTGGTTCTTC 999
CircularRNA5          ATCGAAACTTTAATACAACCTTTTTTGATCCTGCAGGAGGGGGAGACCCAATATTATACCAGCATCTCTTTTGGTTCTTC 979
CircularRNA6          ATCGAAACTTTAATACAACCTTTTTTGATCCTGCAGGAGGGGGAGACCCAATATTATACCAGCATCTCTTTTGGTTCTTC 980
Cir_Forward.Primer1   -------------------------------------------------------------------------------- 22
Cir_Forward.Primer2   -------------------------------------------------------------------------------- -
Cir_Forward.Primer3   -------------------------------------------------------------------------------- -
Cir_Forward.Primer4   -------------------------------------------------------------------------------- 23
Cir_Forward.Primer5   -------------------------------------------------------------------------------- 24
Cir_Reverse.Primer1   -------------------------------------------------------------------------------- 23
Cir_Reverse.Primer2   -------------------------------------------------------------------------------- 20
Cir_Reverse.Primer3   -------------------------------------------------------------------------------- 21
Cir_Reverse.Primer4   -------------------------------------------------------------------------------- 24
Cir_Reverse.Primer5   -------------------------------------------------------------------------------- 26

                                          2.180               2.200               2.220               2.240
Genomic(MLOC_370)     GGTCATCCAGAGGTGTATATTCTCATTCTGCCTGGATTTGGTATTATTAGTCATATCGTATCGACCTTTTCAAGAAAACC 2240
cDNA1(MLOC_370)       GGTCATCCAGAGGTGTATATTCTCATTCTGCCTGGATTTGGTATTATTAGTCATATCGTATCGACCTTTTCAAGAAAACC 2240
CircularRNA1          -------------------------------------------------------------------------------- 347
CircularRNA2          -------------------------------------------------------------------------------- 370
CircularRNA3          -------------------------------------------------------------------------------- 393
CircularRNA4          GGTCATCCAGAGGTGTATATTCTCATTCTGCCTGGATTTGGTATTATTAGTCATATCGTATCGACCTTTTCAAGAAAACC 1079
CircularRNA5          GGTCATCCAGAGGTGTATATTCTCATTCTGCCTGGATTTGGTATTATTAGTCATATCGTATCGACCTTTTCAAGAAAACC 1059
CircularRNA6          GGTCATCCAGAGGTGTATATTCTCATTCTGCCTGGATTTGGTATTATTAGTCATATCGTATCGACCTTTTCAAGAAAACC 1060
Cir_Forward.Primer1   -------------------------------------------------------------------------------- 22
Cir_Forward.Primer2   -------------------------------------------------------------------------------- -
Cir_Forward.Primer3   -------------------------------------------------------------------------------- -
Cir_Forward.Primer4   -------------------------------------------------------------------------------- 23
Cir_Forward.Primer5   -------------------------------------------------------------------------------- 24
Cir_Reverse.Primer1   -------------------------------------------------------------------------------- 23
Cir_Reverse.Primer2   -------------------------------------------------------------------------------- 20
Cir_Reverse.Primer3   -------------------------------------------------------------------------------- 21
Cir_Reverse.Primer4   -------------------------------------------------------------------------------- 24
Cir_Reverse.Primer5   -------------------------------------------------------------------------------- 26
```

```
                                                                        2.260               2.280               2.300               2.320
Genomic(MLOC_370)     GGTCTTCGGGTATCTAGGCATGGTTTATGCCATGATAAGTATAGGTGTTCTTGGATTTCTAGTTTGGGCTCATCATATGT 2320
cDNA1(MLOC_370)       GGTCTTCGGGTATCTAGGCATGGTTTATGCCATGATAAGTATAGGTGTTCTTGGATTTCTAGTTTGGGCTCATCATATGT 2320
CircularRNA1          -------------------------------------------------------------------------------- 347
CircularRNA2          -------------------------------------------------------------------------------- 370
CircularRNA3          -------------------------------------------------------------------------------- 393
CircularRNA4          GGTCTTCGGGTATCTAGGCATGGTTTATGCCATGATAAGTATAGGTGTTCTTGGATTTCTAGTTTGGGCTCATCATATGT 1159
CircularRNA5          GGTCTTCGGGTATCTAGGCATGGTTTATGCCATGATAAGTATAGGTGTTCTTGGATTTCTAGTTTGGGCTCATCATATGT 1139
CircularRNA6          GGTCTTCGGGTATCTAGGCATGGTTTATGCCATGATAAGTATAGGTGTTCTTGGATTTCTAGTTTGGGCTCATCATATGT 1140
Cir_Forward.Primer1   -------------------------------------------------------------------------------- 22
Cir_Forward.Primer2   -------------------------------------------------------------------------------- -
Cir_Forward.Primer3   -------------------------------------------------------------------------------- -
Cir_Forward.Primer4   -------------------------------------------------------------------------------- 23
Cir_Forward.Primer5   -------------------------------------------------------------------------------- 24
Cir_Reverse.Primer1   -------------------------------------------------------------------------------- 23
Cir_Reverse.Primer2   -------------------------------------------------------------------------------- 20
Cir_Reverse.Primer3   -------------------------------------------------------------------------------- 21
Cir_Reverse.Primer4   -------------------------------------------------------------------------------- 24
Cir_Reverse.Primer5   -------------------------------------------------------------------------------- 26
```

```
                                                                        2.340               2.360               2.380               2.400
Genomic(MLOC_370)     TTACTGTGGGCTTAGACGTTGATACGCGTGCCTACTTCACCGCAGCTACCATGATCATAGCTGTGCCCACAGGAATCAAA 2400
cDNA1(MLOC_370)       TTACTGTGGGCTTAGACGTTGATACGCGTGCCTACTTCACCGCAGCTACCATGATCATAGCTGTGCCCACAGGAATCAAA 2400
CircularRNA1          -------------------------------------------------------------------------------- 347
CircularRNA2          -------------------------------------------------------------------------------- 370
CircularRNA3          -------------------------------------------------------------------------------- 393
CircularRNA4          TTACTGTGGGCTTAGACGTTGATACGCGTGCCTACTTCACCGCAGCTACCATGATCATAGCTGTGCCCACAGGAATCAAA 1239
CircularRNA5          TTACTGTGGGCTTAGACGTTGATACGCGTGCCTACTTCACCGCAGCTACCATGATCATAGCTGTGCCCACAGGAATCAAA 1219
CircularRNA6          TTACTGTGGGCTTAGACGTTGATACGCGTGCCTACTTCACCGCAGCTACCATGATCATAGCTGTGCCCACAGGAATCAAA 1220
Cir_Forward.Primer1   -------------------------------------------------------------------------------- 22
Cir_Forward.Primer2   -------------------------------------------------------------------------------- -
Cir_Forward.Primer3   -------------------------------------------------------------------------------- -
Cir_Forward.Primer4   -------------------------------------------------------------------------------- 23
Cir_Forward.Primer5   -------------------------------------------------------------------------------- 24
Cir_Reverse.Primer1   -------------------------------------------------------------------------------- 23
Cir_Reverse.Primer2   -------------------------------------------------------------------------------- 20
Cir_Reverse.Primer3   -------------------------------------------------------------------------------- 21
Cir_Reverse.Primer4   -------------------------------------------------------------------------------- 24
Cir_Reverse.Primer5   -------------------------------------------------------------------------------- 26
```

```
                                                                        2.420               2.440               2.460               2.480
Genomic(MLOC_370)     ATCTTTAGTTGGATCGCTACCATGTGGGGAGGTTCGATACAATACAAAACACCCATGTTATTTGCTGTAGGGTTCATCTT 2480
cDNA1(MLOC_370)       ATCTTTAGTTGGATCGCTACCATGTGGGGAGGTTCGATACAATACAAAACACCCATGTTATTTGCTGTAGGGTTCATCTT 2480
CircularRNA1          -------------------------------------------------------------------------------- 347
CircularRNA2          -------------------------------------------------------------------------------- 370
CircularRNA3          -------------------------------------------------------------------------------- 393
CircularRNA4          ATCTTTAGTTGGATCGCTACCATGTGGGGAGGTTCGATACAATACAAAACACCCATGTTATTTGCTGTAGGGTTCATCTT 1319
CircularRNA5          ATCTTTAGTTGGATCGCTACCATGTGGGGAGGTTCGATACAATACAAAACACCCATGTTATTTGCTGTAGGGTTCATCTT 1299
CircularRNA6          ATCTTTAGTTGGATCGCTACCATGTGGGGAGGTTCGATACAATACAAAACACCCATGTTATTTGCTGTAGGGTTCATCTT 1300
Cir_Forward.Primer1   -------------------------------------------------------------------------------- 22
Cir_Forward.Primer2   -------------------------------------------------------------------------------- -
Cir_Forward.Primer3   -------------------------------------------------------------------------------- -
Cir_Forward.Primer4   -------------------------------------------------------------------------------- 23
Cir_Forward.Primer5   -------------------------------------------------------------------------------- 24
Cir_Reverse.Primer1   -------------------------------------------------------------------------------- 23
Cir_Reverse.Primer2   -------------------------------------------------------------------------------- 20
Cir_Reverse.Primer3   -------------------------------------------------------------------------------- 21
Cir_Reverse.Primer4   -------------------------------------------------------------------------------- 24
Cir_Reverse.Primer5   -------------------------------------------------------------------------------- 26
```

```
                                                                        2.500               2.520               2.540               2.560
Genomic(MLOC_370)     TTTGTTCACCATAGGAGGGCTCACTGGAATAGTTCTAGCAAACTCTGGGCTAGACATTGCTCTACATGATACTTATTATG 2560
cDNA1(MLOC_370)       TTTGTTCACCATAGGAGGGCTCACTGGAATAGTTCTAGCAAACTCTGGGCTAGACATTGCTCTACATGATACTTATTATG 2560
CircularRNA1          -------------------------------------------------------------------------------- 347
CircularRNA2          -------------------------------------------------------------------------------- 370
CircularRNA3          -------------------------------------------------------------------------------- 393
CircularRNA4          TTTGTTCACCATAGGAGGGCTCACTGGAATAGTTCTAGCAAACTCTGGGCTAGACATTGCTCTACATGATACTTATTATG 1399
CircularRNA5          TTTGTTCACCATAGGAGGGCTCACTGGAATAGTTCTAGCAAACTCTGGGCTAGACATTGCTCTACATGATACTTATTATG 1379
CircularRNA6          TTTGTTCACCATAGGAGGGCTCACTGGAATAGTTCTAGCAAACTCTGGGCTAGACATTGCTCTACATGATACTTATTATG 1380
Cir_Forward.Primer1   -------------------------------------------------------------------------------- 22
Cir_Forward.Primer2   -------------------------------------------------------------------------------- -
Cir_Forward.Primer3   -------------------------------------------------------------------------------- -
Cir_Forward.Primer4   -------------------------------------------------------------------------------- 23
Cir_Forward.Primer5   -------------------------------------------------------------------------------- 24
Cir_Reverse.Primer1   -------------------------------------------------------------------------------- 23
Cir_Reverse.Primer2   -------------------------------------------------------------------------------- 20
Cir_Reverse.Primer3   -------------------------------------------------------------------------------- 21
Cir_Reverse.Primer4   -------------------------------------------------------------------------------- 24
Cir_Reverse.Primer5   -------------------------------------------------------------------------------- 26
```

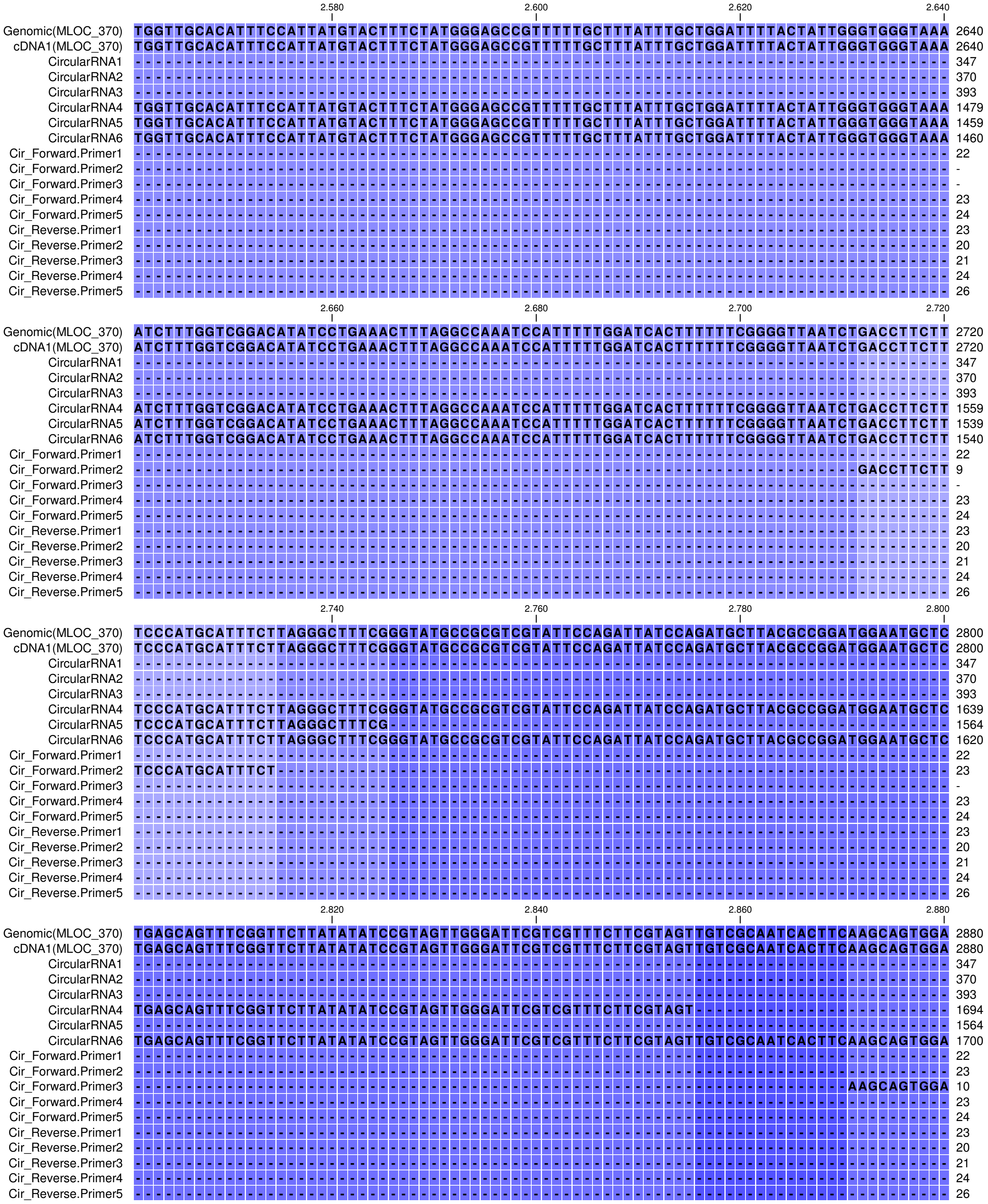
2.580 2.600 2.620 2.640
Genomic(MLOC_370) TGGTTGCACATTTCCATTATGTACTTTCTATGGGAGCCGTTTTTGCTTTATTTGCTGGATTTTACTATTGGGTGGGTAAA 2640
cDNA1(MLOC_370) TGGTTGCACATTTCCATTATGTACTTTCTATGGGAGCCGTTTTTGCTTTATTTGCTGGATTTTACTATTGGGTGGGTAAA 2640
CircularRNA1 347
CircularRNA2 370
CircularRNA3 393
CircularRNA4 TGGTTGCACATTTCCATTATGTACTTTCTATGGGAGCCGTTTTTGCTTTATTTGCTGGATTTTACTATTGGGTGGGTAAA 1479
CircularRNA5 TGGTTGCACATTTCCATTATGTACTTTCTATGGGAGCCGTTTTTGCTTTATTTGCTGGATTTTACTATTGGGTGGGTAAA 1459
CircularRNA6 TGGTTGCACATTTCCATTATGTACTTTCTATGGGAGCCGTTTTTGCTTTATTTGCTGGATTTTACTATTGGGTGGGTAAA 1460
Cir_Forward.Primer1 22
Cir_Forward.Primer2 -
Cir_Forward.Primer3 -
Cir_Forward.Primer4 23
Cir_Forward.Primer5 24
Cir_Reverse.Primer1 23
Cir_Reverse.Primer2 20
Cir_Reverse.Primer3 21
Cir_Reverse.Primer4 24
Cir_Reverse.Primer5 26
2.660 2.680 2.700 2.720
Genomic(MLOC_370) ATCTTTGGTCGGACATATCCTGAAACTTTAGGCCAAATCCATTTTTGGATCACTTTTTTCGGGGTTAATCTGACCTTCTT 2720
cDNA1(MLOC_370) ATCTTTGGTCGGACATATCCTGAAACTTTAGGCCAAATCCATTTTTGGATCACTTTTTTCGGGGTTAATCTGACCTTCTT 2720
CircularRNA1 347
CircularRNA2 370
CircularRNA3 393
CircularRNA4 ATCTTTGGTCGGACATATCCTGAAACTTTAGGCCAAATCCATTTTTGGATCACTTTTTTCGGGGTTAATCTGACCTTCTT 1559
CircularRNA5 ATCTTTGGTCGGACATATCCTGAAACTTTAGGCCAAATCCATTTTTGGATCACTTTTTTCGGGGTTAATCTGACCTTCTT 1539
CircularRNA6 ATCTTTGGTCGGACATATCCTGAAACTTTAGGCCAAATCCATTTTTGGATCACTTTTTTCGGGGTTAATCTGACCTTCTT 1540
Cir_Forward.Primer1 22
Cir_Forward.Primer2 GACCTTCTT 9
Cir_Forward.Primer3 -
Cir_Forward.Primer4 23
Cir_Forward.Primer5 24
Cir_Reverse.Primer1 23
Cir_Reverse.Primer2 20
Cir_Reverse.Primer3 21
Cir_Reverse.Primer4 24
Cir_Reverse.Primer5 26
2.740 2.760 2.780 2.800
Genomic(MLOC_370) TCCCATGCATTTCTTAGGGCTTTCGGGTATGCCGCGTCGTATTCCAGATTATCCAGATGCTTACGCCGGATGGAATGCTC 2800
cDNA1(MLOC_370) TCCCATGCATTTCTTAGGGCTTTCGGGTATGCCGCGTCGTATTCCAGATTATCCAGATGCTTACGCCGGATGGAATGCTC 2800
CircularRNA1 347
CircularRNA2 370
CircularRNA3 393
CircularRNA4 TCCCATGCATTTCTTAGGGCTTTCGGGTATGCCGCGTCGTATTCCAGATTATCCAGATGCTTACGCCGGATGGAATGCTC 1639
CircularRNA5 TCCCATGCATTTCTTAGGGCTTTCG 1564
CircularRNA6 TCCCATGCATTTCTTAGGGCTTTCGGGTATGCCGCGTCGTATTCCAGATTATCCAGATGCTTACGCCGGATGGAATGCTC 1620
Cir_Forward.Primer1 22
Cir_Forward.Primer2 TCCCATGCATTTCT 23
Cir_Forward.Primer3 -
Cir_Forward.Primer4 23
Cir_Forward.Primer5 24
Cir_Reverse.Primer1 23
Cir_Reverse.Primer2 20
Cir_Reverse.Primer3 21
Cir_Reverse.Primer4 24
Cir_Reverse.Primer5 26
2.820 2.840 2.860 2.880
Genomic(MLOC_370) TGAGCAGTTTCGGTTCTTATATATCCGTAGTTGGGATTCGTCGTTTCTTCGTAGTTGTCGCAATCACTTCAAGCAGTGGA 2880
cDNA1(MLOC_370) TGAGCAGTTTCGGTTCTTATATATCCGTAGTTGGGATTCGTCGTTTCTTCGTAGTTGTCGCAATCACTTCAAGCAGTGGA 2880
CircularRNA1 347
CircularRNA2 370
CircularRNA3 393
CircularRNA4 TGAGCAGTTTCGGTTCTTATATATCCGTAGTTGGGATTCGTCGTTTCTTCGTAGT 1694
CircularRNA5 1564
CircularRNA6 TGAGCAGTTTCGGTTCTTATATATCCGTAGTTGGGATTCGTCGTTTCTTCGTAGTTGTCGCAATCACTTCAAGCAGTGGA 1700
Cir_Forward.Primer1 22
Cir_Forward.Primer2 23
Cir_Forward.Primer3 AAGCAGTGGA 10
Cir_Forward.Primer4 23
Cir_Forward.Primer5 24
Cir_Reverse.Primer1 23
Cir_Reverse.Primer2 20
Cir_Reverse.Primer3 21
Cir_Reverse.Primer4 24
Cir_Reverse.Primer5 26

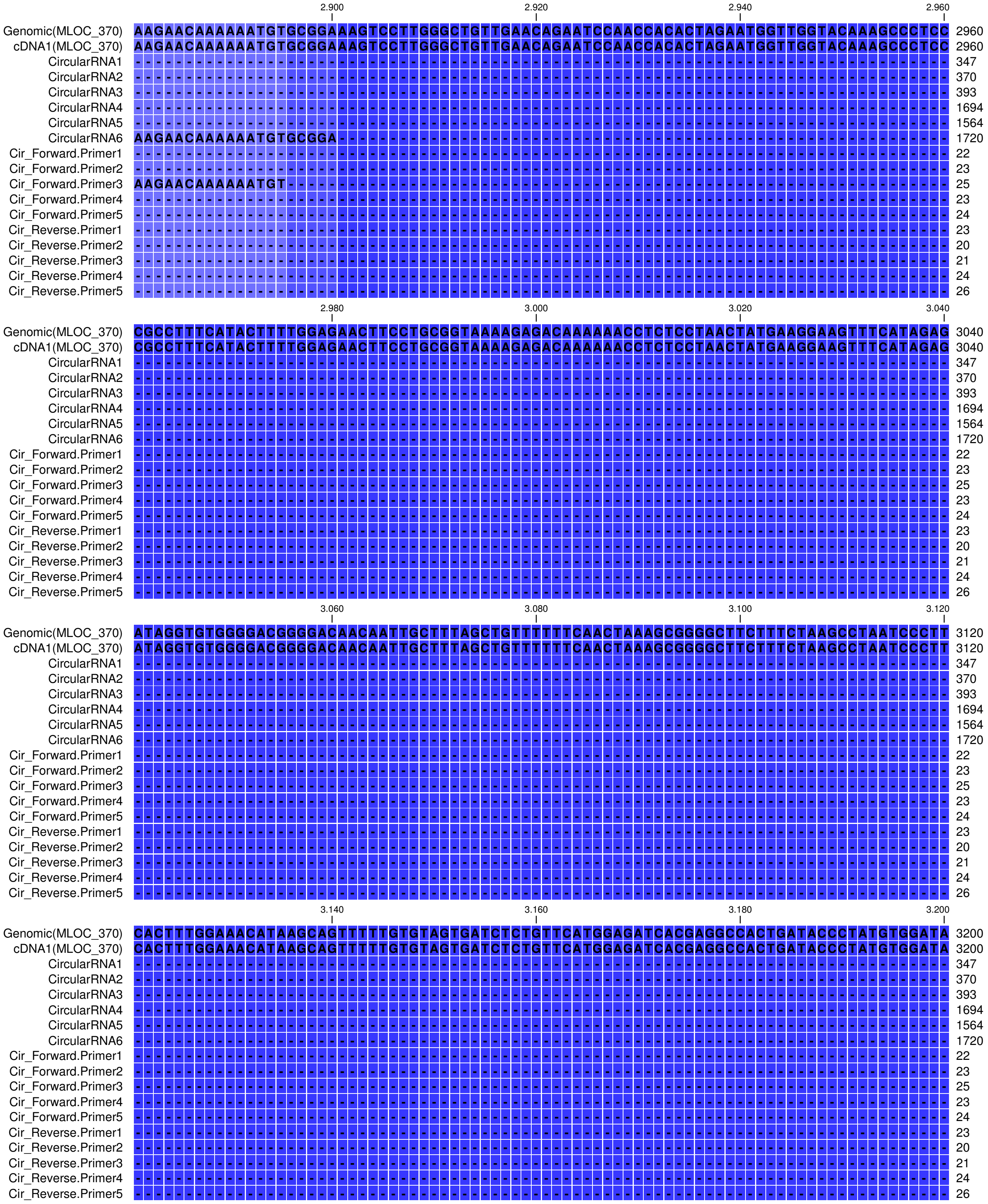

2.900 2.920 2.940 2.960
Genomic(MLOC_370) AAGAACAAAAAATGTGCGGAAAGTCCTTGGGCTGTTGAACAGAATCCAACCACACTAGAATGGTTGGTACAAAGCCCTCC 2960
cDNA1(MLOC_370) AAGAACAAAAAATGTGCGGAAAGTCCTTGGGCTGTTGAACAGAATCCAACCACACTAGAATGGTTGGTACAAAGCCCTCC 2960
CircularRNA1 347
CircularRNA2 370
CircularRNA3 393
CircularRNA4 1694
CircularRNA5 1564
CircularRNA6 AAGAACAAAAAATGTGCGGA 1720
Cir_Forward.Primer1 22
Cir_Forward.Primer2 23
Cir_Forward.Primer3 AAGAACAAAAAATGT 25
Cir_Forward.Primer4 23
Cir_Forward.Primer5 24
Cir_Reverse.Primer1 23
Cir_Reverse.Primer2 20
Cir_Reverse.Primer3 21
Cir_Reverse.Primer4 24
Cir_Reverse.Primer5 26
2.980 3.000 3.020 3.040
Genomic(MLOC_370) CGCCTTTCATACTTTTGGAGAACTTCCTGCGGTAAAAGAGACAAAAAACCTCTCCTAACTATGAAGGAAGTTTCATAGAG 3040
cDNA1(MLOC_370) CGCCTTTCATACTTTTGGAGAACTTCCTGCGGTAAAAGAGACAAAAAACCTCTCCTAACTATGAAGGAAGTTTCATAGAG 3040
CircularRNA1 347
CircularRNA2 370
CircularRNA3 393
CircularRNA4 1694
CircularRNA5 1564
CircularRNA6 1720
Cir_Forward.Primer1 22
Cir_Forward.Primer2 23
Cir_Forward.Primer3 25
Cir_Forward.Primer4 23
Cir_Forward.Primer5 24
Cir_Reverse.Primer1 23
Cir_Reverse.Primer2 20
Cir_Reverse.Primer3 21
Cir_Reverse.Primer4 24
Cir_Reverse.Primer5 26
3.060 3.080 3.100 3.120
Genomic(MLOC_370) ATAGGTGTGGGGACGGGGACAACAATTGCTTTAGCTGTTTTTTCAACTAAAGCGGGGCTTCTTTCTAAGCCTAATCCCTT 3120
cDNA1(MLOC_370) ATAGGTGTGGGGACGGGGACAACAATTGCTTTAGCTGTTTTTTCAACTAAAGCGGGGCTTCTTTCTAAGCCTAATCCCTT 3120
CircularRNA1 347
CircularRNA2 370
CircularRNA3 393
CircularRNA4 1694
CircularRNA5 1564
CircularRNA6 1720
Cir_Forward.Primer1 22
Cir_Forward.Primer2 23
Cir_Forward.Primer3 25
Cir_Forward.Primer4 23
Cir_Forward.Primer5 24
Cir_Reverse.Primer1 23
Cir_Reverse.Primer2 20
Cir_Reverse.Primer3 21
Cir_Reverse.Primer4 24
Cir_Reverse.Primer5 26
3.140 3.160 3.180 3.200
Genomic(MLOC_370) CACTTTGGAAACATAAGCAGTTTTTGTGTAGTGATCTCTGTTCATGGAGATCACGAGGCCACTGATACCCTATGTGGATA 3200
cDNA1(MLOC_370) CACTTTGGAAACATAAGCAGTTTTTGTGTAGTGATCTCTGTTCATGGAGATCACGAGGCCACTGATACCCTATGTGGATA 3200
CircularRNA1 347
CircularRNA2 370
CircularRNA3 393
CircularRNA4 1694
CircularRNA5 1564
CircularRNA6 1720
Cir_Forward.Primer1 22
Cir_Forward.Primer2 23
Cir_Forward.Primer3 25
Cir_Forward.Primer4 23
Cir_Forward.Primer5 24
Cir_Reverse.Primer1 23
Cir_Reverse.Primer2 20
Cir_Reverse.Primer3 21
Cir_Reverse.Primer4 24
Cir_Reverse.Primer5 26

```
                                                            3.220               3.240               3.260               3.280
                                                                |                   |                   |                   |
Genomic(MLOC_370)    GGTGGGATAGAAGAGTGGGAAAGCGGGCTTCTATCACATAGTTTATCTTTCAGGGTACATAGGACGGCCCTCTCGTCATT 3280
cDNA1(MLOC_370)      GGTGGGATAGAAGAGTGGGAAAGCGGGCTTCTATCACATAGTTTATCTTTCAGGGTACATAGGACGGCCCTCTCGTCATT 3280
CircularRNA1         -------------------------------------------------------------------------------- 347
CircularRNA2         -------------------------------------------------------------------------------- 370
CircularRNA3         -------------------------------------------------------------------------------- 393
CircularRNA4         -------------------------------------------------------------------------------- 1694
CircularRNA5         -------------------------------------------------------------------------------- 1564
CircularRNA6         -------------------------------------------------------------------------------- 1720
Cir_Forward.Primer1  -------------------------------------------------------------------------------- 22
Cir_Forward.Primer2  -------------------------------------------------------------------------------- 23
Cir_Forward.Primer3  -------------------------------------------------------------------------------- 25
Cir_Forward.Primer4  -------------------------------------------------------------------------------- 23
Cir_Forward.Primer5  -------------------------------------------------------------------------------- 24
Cir_Reverse.Primer1  -------------------------------------------------------------------------------- 23
Cir_Reverse.Primer2  -------------------------------------------------------------------------------- 20
Cir_Reverse.Primer3  -------------------------------------------------------------------------------- 21
Cir_Reverse.Primer4  -------------------------------------------------------------------------------- 24
Cir_Reverse.Primer5  -------------------------------------------------------------------------------- 26

                                                            3.300               3.320               3.340               3.360
                                                                |                   |                   |                   |
Genomic(MLOC_370)    ACCTTTTGTGCTTACCCGTAGCTAGCTGTGCCTGAAGTCATGGATGATGCTATTCCCACTGAGAGATGTGCTGCAAAATG 3360
cDNA1(MLOC_370)      ACCTTTTGTGCTTACCCGTAGCTAGCTGTGCCTGAAGTCATGGATGATGCTATTCCCACTGAGAGATGTGCTGCAAAATG 3360
CircularRNA1         -------------------------------------------------------------------------------- 347
CircularRNA2         -------------------------------------------------------------------------------- 370
CircularRNA3         -------------------------------------------------------------------------------- 393
CircularRNA4         -------------------------------------------------------------------------------- 1694
CircularRNA5         -------------------------------------------------------------------------------- 1564
CircularRNA6         -------------------------------------------------------------------------------- 1720
Cir_Forward.Primer1  -------------------------------------------------------------------------------- 22
Cir_Forward.Primer2  -------------------------------------------------------------------------------- 23
Cir_Forward.Primer3  -------------------------------------------------------------------------------- 25
Cir_Forward.Primer4  -------------------------------------------------------------------------------- 23
Cir_Forward.Primer5  -------------------------------------------------------------------------------- 24
Cir_Reverse.Primer1  -------------------------------------------------------------------------------- 23
Cir_Reverse.Primer2  -------------------------------------------------------------------------------- 20
Cir_Reverse.Primer3  -------------------------------------------------------------------------------- 21
Cir_Reverse.Primer4  -------------------------------------------------------------------------------- 24
Cir_Reverse.Primer5  -------------------------------------------------------------------------------- 26

                                                            3.380               3.400               3.420               3.440
                                                                |                   |                   |                   |
Genomic(MLOC_370)    GAAGCTGTGTTAGTCCTCGGCTAAAATCATCTTCCTTTATTAAAGACCATATATATCCATATGTAGATAGAAGAGGGGAT 3440
cDNA1(MLOC_370)      GAAGCTGTGTTAGTCCTCGGCTAAAATCATCTTCCTTTATTAAAGACCATATATATCCATATGTAGATAGAAGAGGGGAT 3440
CircularRNA1         -------------------------------------------------------------------------------- 347
CircularRNA2         -------------------------------------------------------------------------------- 370
CircularRNA3         -------------------------------------------------------------------------------- 393
CircularRNA4         -------------------------------------------------------------------------------- 1694
CircularRNA5         -------------------------------------------------------------------------------- 1564
CircularRNA6         -------------------------------------------------------------------------------- 1720
Cir_Forward.Primer1  -------------------------------------------------------------------------------- 22
Cir_Forward.Primer2  -------------------------------------------------------------------------------- 23
Cir_Forward.Primer3  -------------------------------------------------------------------------------- 25
Cir_Forward.Primer4  -------------------------------------------------------------------------------- 23
Cir_Forward.Primer5  -------------------------------------------------------------------------------- 24
Cir_Reverse.Primer1  -------------------------------------------------------------------------------- 23
Cir_Reverse.Primer2  -------------------------------------------------------------------------------- 20
Cir_Reverse.Primer3  -------------------------------------------------------------------------------- 21
Cir_Reverse.Primer4  -------------------------------------------------------------------------------- 24
Cir_Reverse.Primer5  -------------------------------------------------------------------------------- 26

                                                            3.460               3.480               3.500               3.520
                                                                |                   |                   |                   |
Genomic(MLOC_370)    ATAGTCGTTCAAAGCTAAGGTGAGTCTGTTCCAGCTAATTCCTCAGATTCCACTTTCCTGTCTGTCCATGTAAAAAGGAG 3520
cDNA1(MLOC_370)      ATAGTCGTTCAAAGCTAAGGTGAGTCTGTTCCAGCTAATTCCTCAGATTCCACTTTCCTGTCTGTCCATGTAAAAAGGAG 3520
CircularRNA1         -------------------------------------------------------------------------------- 347
CircularRNA2         -------------------------------------------------------------------------------- 370
CircularRNA3         -------------------------------------------------------------------------------- 393
CircularRNA4         -------------------------------------------------------------------------------- 1694
CircularRNA5         -------------------------------------------------------------------------------- 1564
CircularRNA6         -------------------------------------------------------------------------------- 1720
Cir_Forward.Primer1  -------------------------------------------------------------------------------- 22
Cir_Forward.Primer2  -------------------------------------------------------------------------------- 23
Cir_Forward.Primer3  -------------------------------------------------------------------------------- 25
Cir_Forward.Primer4  -------------------------------------------------------------------------------- 23
Cir_Forward.Primer5  -------------------------------------------------------------------------------- 24
Cir_Reverse.Primer1  -------------------------------------------------------------------------------- 23
Cir_Reverse.Primer2  -------------------------------------------------------------------------------- 20
Cir_Reverse.Primer3  -------------------------------------------------------------------------------- 21
Cir_Reverse.Primer4  -------------------------------------------------------------------------------- 24
Cir_Reverse.Primer5  -------------------------------------------------------------------------------- 26
```

```
                                                     3.540               3.560               3.580               3.600
                                                         |                   |                   |                   |
Genomic(MLOC_370)   TCCAGCCCACAAGGAGAGGGAATCCACAAAAATATCTGTTTTAGATGCTTTCTACTATGTTTCAAGTGATCAAAGAATAG 3600
cDNA1(MLOC_370)     TCCAGCCCACAAGGAGAGGGAATCCACAAAAATATCTGTTTTAGATGCTTTCTACTATGTTTCAAGTGATCAAAGAATAG 3600
CircularRNA1        -------------------------------------------------------------------------------- 347
CircularRNA2        -------------------------------------------------------------------------------- 370
CircularRNA3        -------------------------------------------------------------------------------- 393
CircularRNA4        -------------------------------------------------------------------------------- 1694
CircularRNA5        -------------------------------------------------------------------------------- 1564
CircularRNA6        -------------------------------------------------------------------------------- 1720
Cir_Forward.Primer1 -------------------------------------------------------------------------------- 22
Cir_Forward.Primer2 -------------------------------------------------------------------------------- 23
Cir_Forward.Primer3 -------------------------------------------------------------------------------- 25
Cir_Forward.Primer4 -------------------------------------------------------------------------------- 23
Cir_Forward.Primer5 -------------------------------------------------------------------------------- 24
Cir_Reverse.Primer1 -------------------------------------------------------------------------------- 23
Cir_Reverse.Primer2 -------------------------------------------------------------------------------- 20
Cir_Reverse.Primer3 -------------------------------------------------------------------------------- 21
Cir_Reverse.Primer4 -------------------------------------------------------------------------------- 24
Cir_Reverse.Primer5 -------------------------------------------------------------------------------- 26

                                                     3.620               3.640               3.660               3.680
                                                         |                   |                   |                   |
Genomic(MLOC_370)   TATAGTATCTTTAGACAGAACTGAAATAGCAGATGCAGATTCGAATGTTAAGGTTATTAAAGCTTAAAAGAAAAAGCAGT 3680
cDNA1(MLOC_370)     TATAGTATCTTTAGACAGAACTGAAATAGCAGATGCAGATTCGAATGTTAAGGTTATTAAAGCTTAAAAGAAAAAGCAGT 3680
CircularRNA1        -------------------------------------------------------------------------------- 347
CircularRNA2        -------------------------------------------------------------------------------- 370
CircularRNA3        -------------------------------------------------------------------------------- 393
CircularRNA4        -------------------------------------------------------------------------------- 1694
CircularRNA5        -------------------------------------------------------------------------------- 1564
CircularRNA6        -------------------------------------------------------------------------------- 1720
Cir_Forward.Primer1 -------------------------------------------------------------------------------- 22
Cir_Forward.Primer2 -------------------------------------------------------------------------------- 23
Cir_Forward.Primer3 -------------------------------------------------------------------------------- 25
Cir_Forward.Primer4 -------------------------------------------------------------------------------- 23
Cir_Forward.Primer5 -------------------------------------------------------------------------------- 24
Cir_Reverse.Primer1 -------------------------------------------------------------------------------- 23
Cir_Reverse.Primer2 -------------------------------------------------------------------------------- 20
Cir_Reverse.Primer3 -------------------------------------------------------------------------------- 21
Cir_Reverse.Primer4 -------------------------------------------------------------------------------- 24
Cir_Reverse.Primer5 -------------------------------------------------------------------------------- 26

                                                     3.700               3.720               3.740               3.760
                                                         |                   |                   |                   |
Genomic(MLOC_370)   CATAGGTTAGGTGACTAAGAAAAGAATGTGGAAAGGGTCACGGATAGGATTGGTATATTAGTTTCAGCTATAACAGAGAT 3760
cDNA1(MLOC_370)     CATAGGTTAGGTGACTAAGAAAAGAATGTGGAAAGGGTCACGGATAGGATTGGTATATTAGTTTCAGCTATAACAGAGAT 3760
CircularRNA1        -------------------------------------------------------------------------------- 347
CircularRNA2        -------------------------------------------------------------------------------- 370
CircularRNA3        -------------------------------------------------------------------------------- 393
CircularRNA4        -------------------------------------------------------------------------------- 1694
CircularRNA5        -------------------------------------------------------------------------------- 1564
CircularRNA6        -------------------------------------------------------------------------------- 1720
Cir_Forward.Primer1 -------------------------------------------------------------------------------- 22
Cir_Forward.Primer2 -------------------------------------------------------------------------------- 23
Cir_Forward.Primer3 -------------------------------------------------------------------------------- 25
Cir_Forward.Primer4 -------------------------------------------------------------------------------- 23
Cir_Forward.Primer5 -------------------------------------------------------------------------------- 24
Cir_Reverse.Primer1 -------------------------------------------------------------------------------- 23
Cir_Reverse.Primer2 -------------------------------------------------------------------------------- 20
Cir_Reverse.Primer3 -------------------------------------------------------------------------------- 21
Cir_Reverse.Primer4 -------------------------------------------------------------------------------- 24
Cir_Reverse.Primer5 -------------------------------------------------------------------------------- 26

                                                     3.780               3.800               3.820               3.840
                                                         |                   |                   |                   |
Genomic(MLOC_370)   TTCTCAGCATGTTTCTATCTGGTTGGTATCCGCTGGTACCATTCATTGTGAGTGCTTTTAGGGGTTGCTCAACTCTCGGC 3840
cDNA1(MLOC_370)     TTCTCAGCATGTTTCTATCTGGTTGGTATCCGCTGGTACCATTCATTGTGAGTGCTTTTAGGGGTTGCTCAACTCTCGGC 3840
CircularRNA1        -------------------------------------------------------------------------------- 347
CircularRNA2        -------------------------------------------------------------------------------- 370
CircularRNA3        -------------------------------------------------------------------------------- 393
CircularRNA4        -------------------------------------------------------------------------------- 1694
CircularRNA5        -------------------------------------------------------------------------------- 1564
CircularRNA6        -------------------------------------------------------------------------------- 1720
Cir_Forward.Primer1 -------------------------------------------------------------------------------- 22
Cir_Forward.Primer2 -------------------------------------------------------------------------------- 23
Cir_Forward.Primer3 -------------------------------------------------------------------------------- 25
Cir_Forward.Primer4 -------------------------------------------------------------------------------- 23
Cir_Forward.Primer5 -------------------------------------------------------------------------------- 24
Cir_Reverse.Primer1 -------------------------------------------------------------------------------- 23
Cir_Reverse.Primer2 -------------------------------------------------------------------------------- 20
Cir_Reverse.Primer3 -------------------------------------------------------------------------------- 21
Cir_Reverse.Primer4 -------------------------------------------------------------------------------- 24
Cir_Reverse.Primer5 -------------------------------------------------------------------------------- 26
```

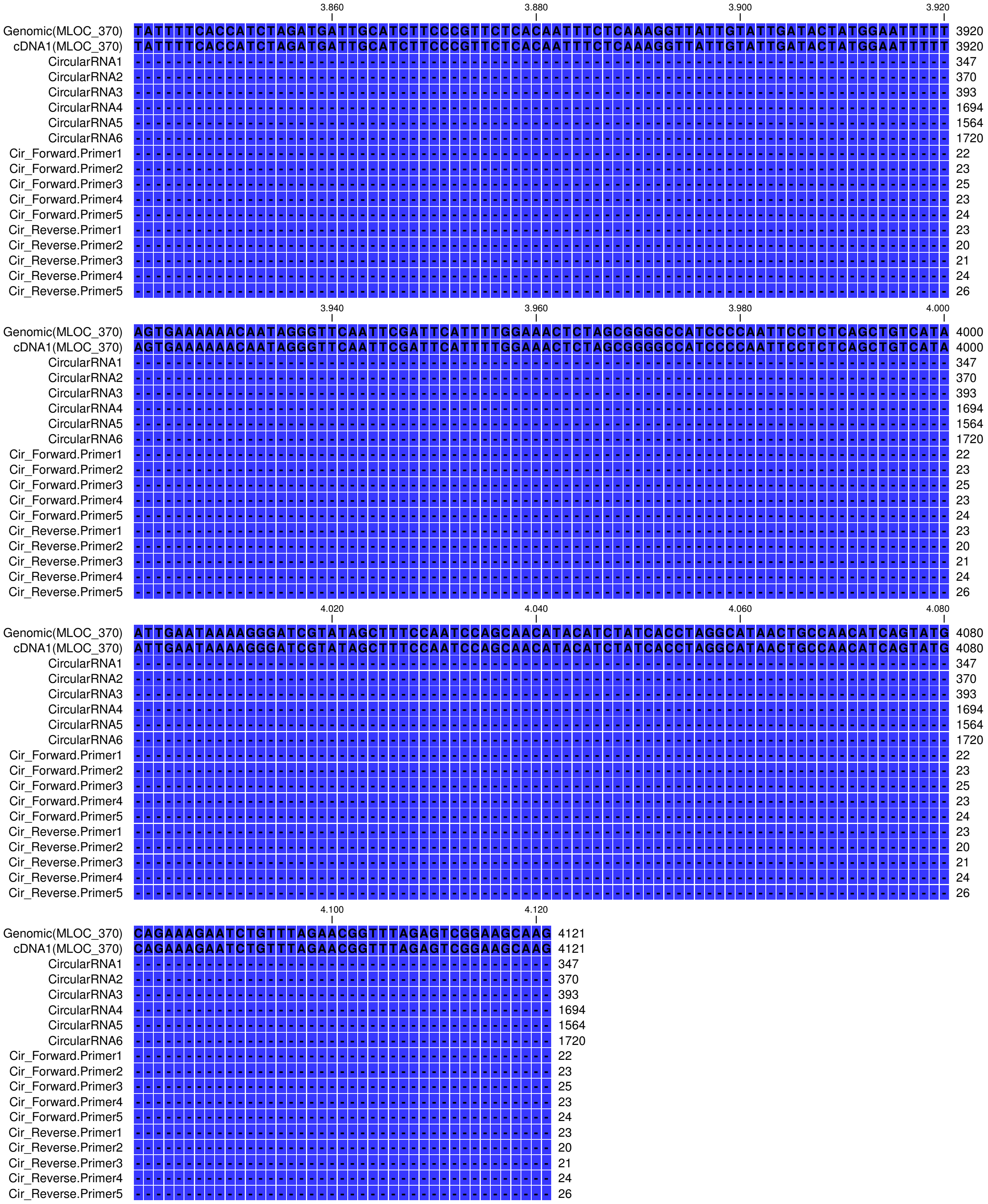
3.860 3.880 3.900 3.920
Genomic(MLOC_370) TATTTTCACCATCTAGATGATTGCATCTTCCCGTTCTCACAATTTCTCAAAGGTTATTGTATTGATACTATGGAATTTTT 3920
cDNA1(MLOC_370) TATTTTCACCATCTAGATGATTGCATCTTCCCGTTCTCACAATTTCTCAAAGGTTATTGTATTGATACTATGGAATTTTT 3920
CircularRNA1 -- 347
CircularRNA2 -- 370
CircularRNA3 -- 393
CircularRNA4 -- 1694
CircularRNA5 -- 1564
CircularRNA6 -- 1720
Cir_Forward.Primer1 -- 22
Cir_Forward.Primer2 -- 23
Cir_Forward.Primer3 -- 25
Cir_Forward.Primer4 -- 23
Cir_Forward.Primer5 -- 24
Cir_Reverse.Primer1 -- 23
Cir_Reverse.Primer2 -- 20
Cir_Reverse.Primer3 -- 21
Cir_Reverse.Primer4 -- 24
Cir_Reverse.Primer5 -- 26
3.940 3.960 3.980 4.000
Genomic(MLOC_370) AGTGAAAAAACAATAGGGTTCAATTCGATTCATTTTGGAAACTCTAGCGGGGCCATCCCCAATTCCTCTCAGCTGTCATA 4000
cDNA1(MLOC_370) AGTGAAAAAACAATAGGGTTCAATTCGATTCATTTTGGAAACTCTAGCGGGGCCATCCCCAATTCCTCTCAGCTGTCATA 4000
CircularRNA1 -- 347
CircularRNA2 -- 370
CircularRNA3 -- 393
CircularRNA4 -- 1694
CircularRNA5 -- 1564
CircularRNA6 -- 1720
Cir_Forward.Primer1 -- 22
Cir_Forward.Primer2 -- 23
Cir_Forward.Primer3 -- 25
Cir_Forward.Primer4 -- 23
Cir_Forward.Primer5 -- 24
Cir_Reverse.Primer1 -- 23
Cir_Reverse.Primer2 -- 20
Cir_Reverse.Primer3 -- 21
Cir_Reverse.Primer4 -- 24
Cir_Reverse.Primer5 -- 26
4.020 4.040 4.060 4.080
Genomic(MLOC_370) ATTGAATAAAAGGGATCGTATAGCTTTCCAATCCAGCAACATACATCTATCACCTAGGCATAACTGCCAACATCAGTATG 4080
cDNA1(MLOC_370) ATTGAATAAAAGGGATCGTATAGCTTTCCAATCCAGCAACATACATCTATCACCTAGGCATAACTGCCAACATCAGTATG 4080
CircularRNA1 -- 347
CircularRNA2 -- 370
CircularRNA3 -- 393
CircularRNA4 -- 1694
CircularRNA5 -- 1564
CircularRNA6 -- 1720
Cir_Forward.Primer1 -- 22
Cir_Forward.Primer2 -- 23
Cir_Forward.Primer3 -- 25
Cir_Forward.Primer4 -- 23
Cir_Forward.Primer5 -- 24
Cir_Reverse.Primer1 -- 23
Cir_Reverse.Primer2 -- 20
Cir_Reverse.Primer3 -- 21
Cir_Reverse.Primer4 -- 24
Cir_Reverse.Primer5 -- 26
4.100 4.120
Genomic(MLOC_370) CAGAAAGAATCTGTTTAGAACGGTTTAGAGTCGGAAGCAAG 4121
cDNA1(MLOC_370) CAGAAAGAATCTGTTTAGAACGGTTTAGAGTCGGAAGCAAG 4121
CircularRNA1 --- 347
CircularRNA2 --- 370
CircularRNA3 --- 393
CircularRNA4 --- 1694
CircularRNA5 --- 1564
CircularRNA6 --- 1720
Cir_Forward.Primer1 --- 22
Cir_Forward.Primer2 --- 23
Cir_Forward.Primer3 --- 25
Cir_Forward.Primer4 --- 23
Cir_Forward.Primer5 --- 24
Cir_Reverse.Primer1 --- 23
Cir_Reverse.Primer2 --- 20
Cir_Reverse.Primer3 --- 21
Cir_Reverse.Primer4 --- 24
Cir_Reverse.Primer5 --- 26

Real-Time PCR for the junction region of cytochrome c oxidase

Cox1_circular RNA1 (ID: Ch1:23865393-238657407)

By divergent primers no. 1 on genomic DNA

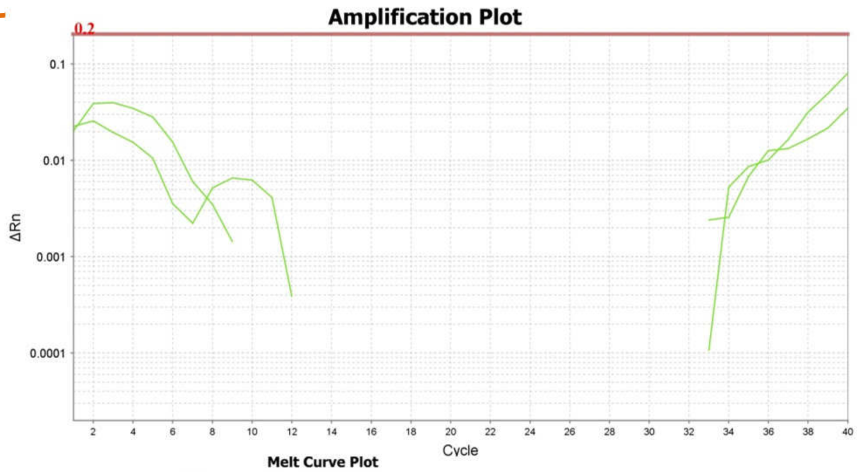

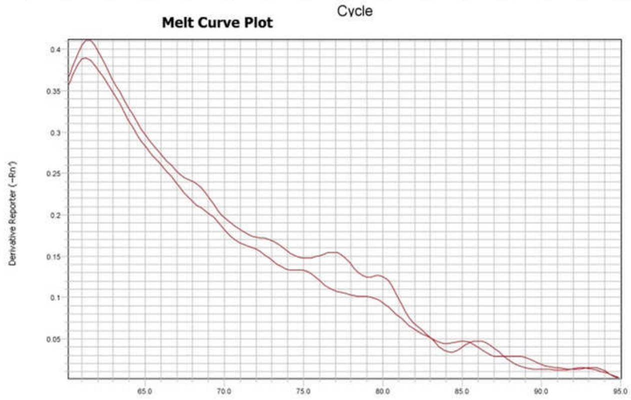

By divergent primers no. 1 on cDNAs

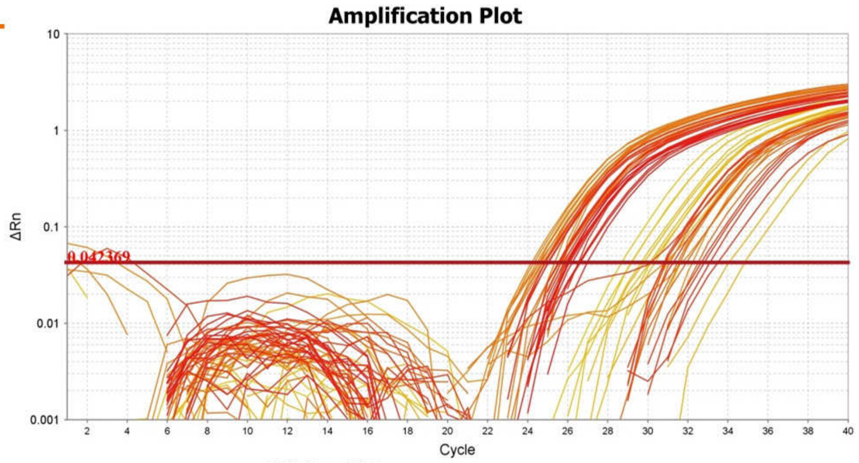

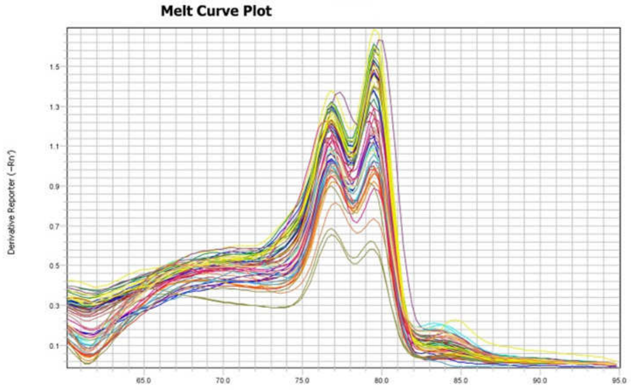

Real-Time PCR for the junction region of cytochrome c oxidase

Cox1_circular RNA1 (ID: Ch1:23865393-238657407**)**

By divergent primers ◀▶ no. 1 with no template

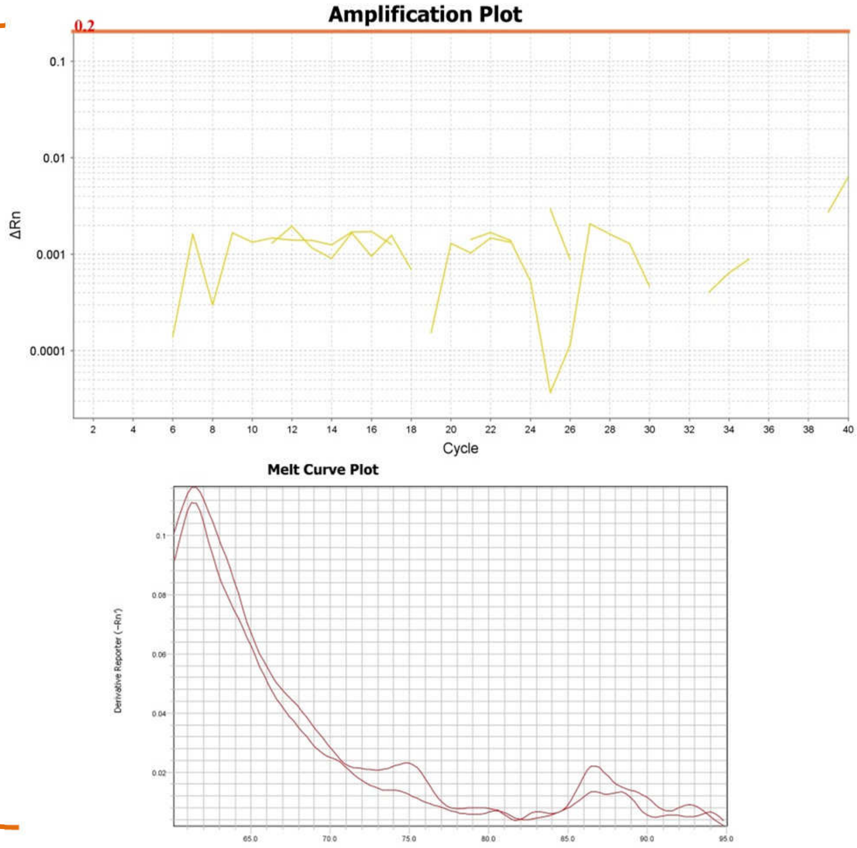

Real-Time PCR for the junction region of cytochrome c oxidase

Cox1_circular RNA4 (ID: Ch1:23864364-23866057)

By divergent primers ◀▶ no. 2 on genomic DNA

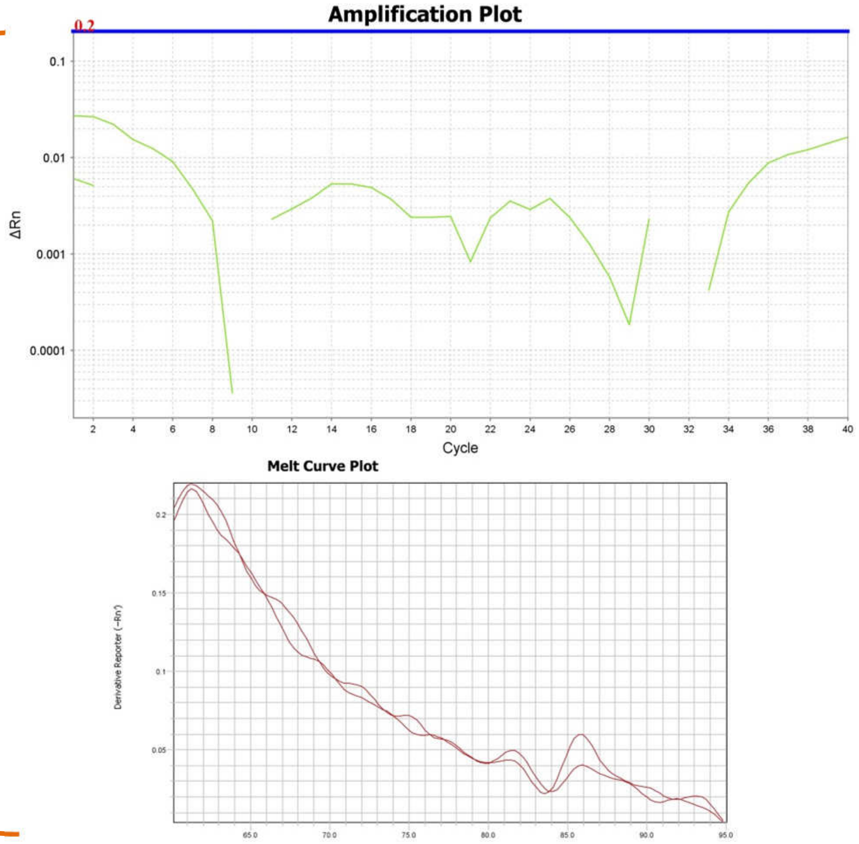

By divergent primers ◀▶ no. 2 on cDNAs

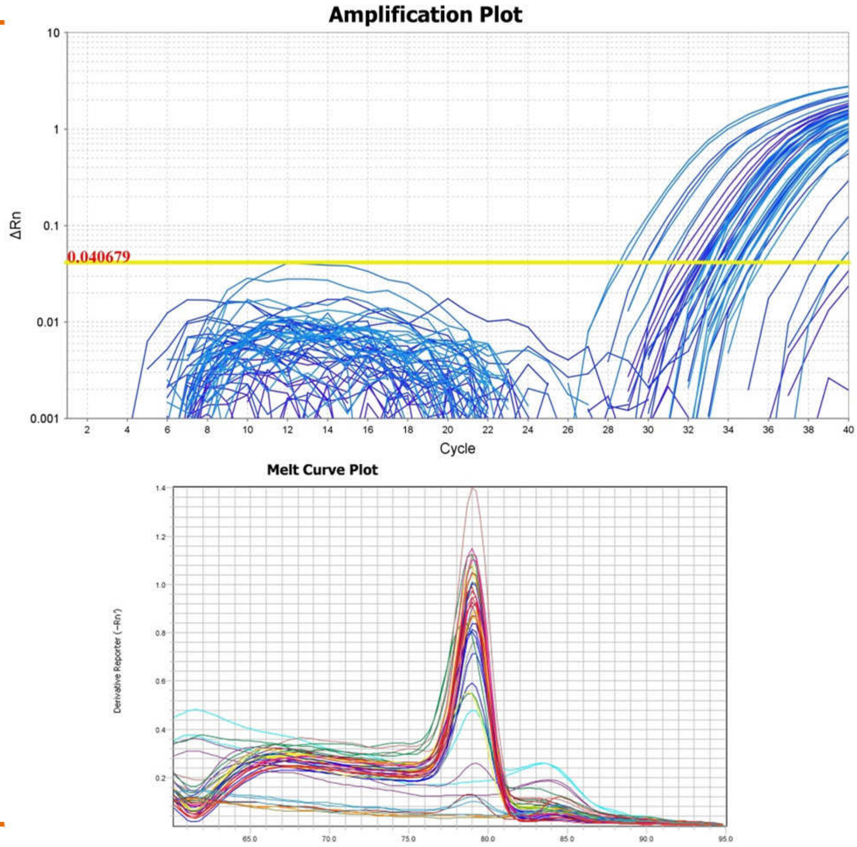

Real-Time PCR for the junction region of cytochrome c oxidase

Cox1_circular RNA4 (ID: Ch1:23864364-23866057**)**

By divergent primers ◀▶ no. 2 with no template

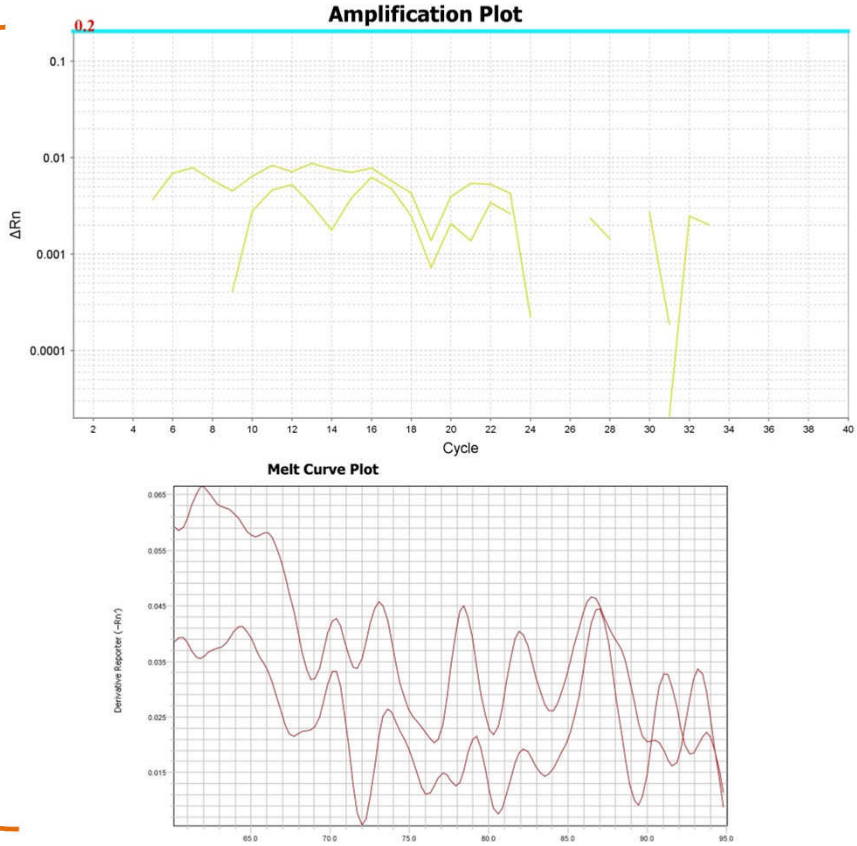

Real-Time PCR for the junction region of cytochrome c oxidase

Cox1_circular RNA6 (ID: Ch1:23864318-23866037)

By divergent primers ◀▶ no. 3 on genomic DNA

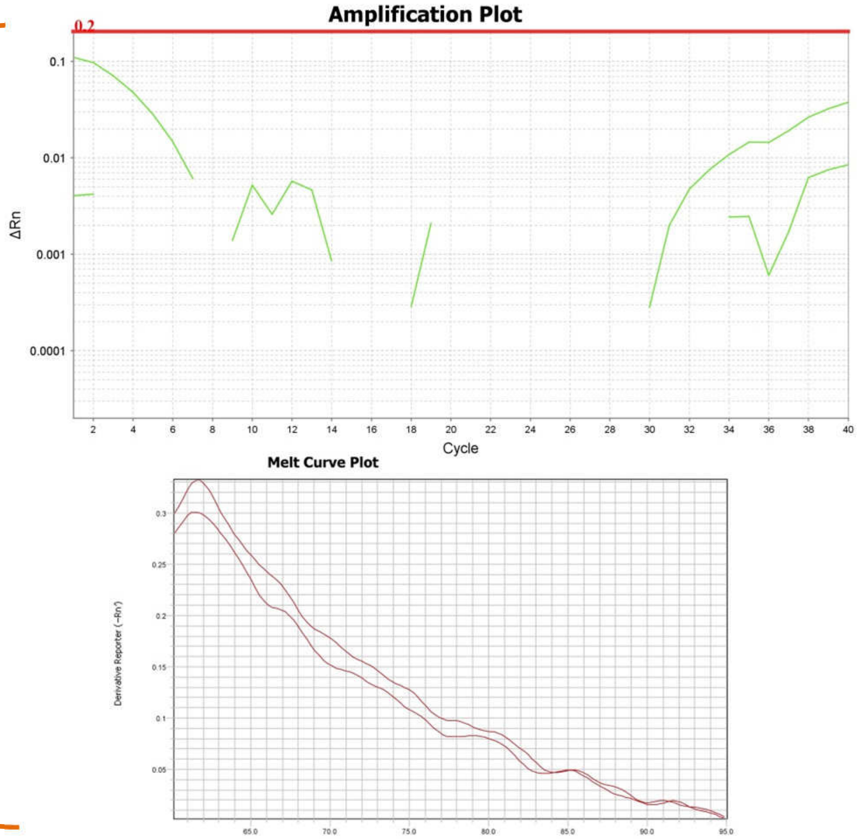

By divergent primers ◀▶ no. 3 on cDNAs

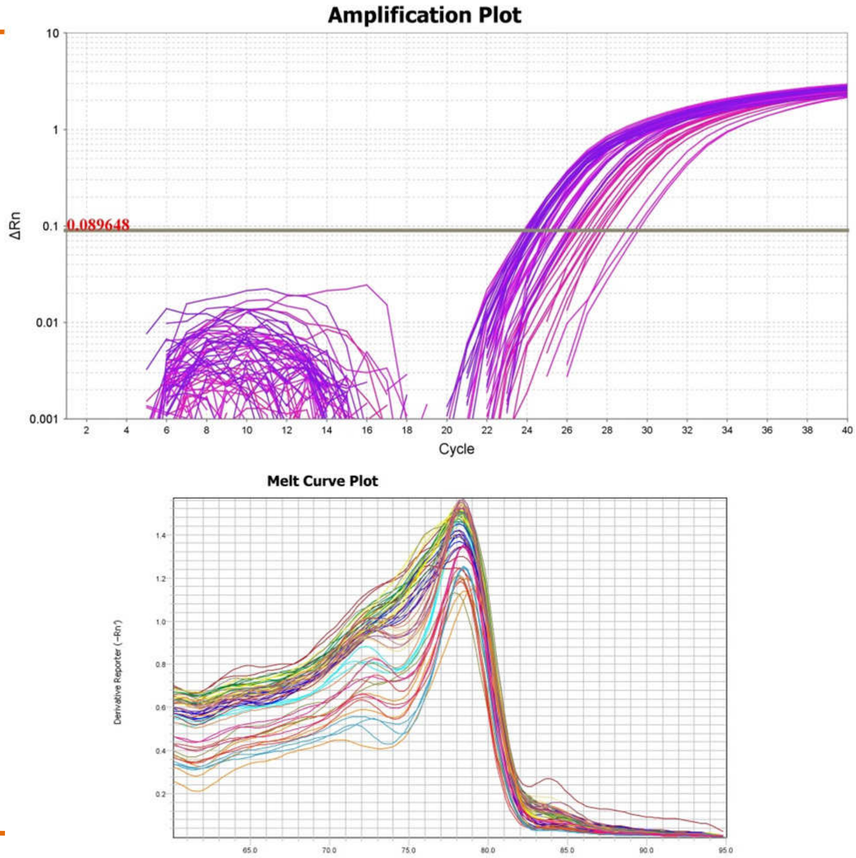

Real-Time PCR for the junction region of cytochrome c oxidase

Cox1_circular RNA6 (ID: Ch1:23864318-23866037**)**

By divergent primers ◀▶ no. 3 with no template

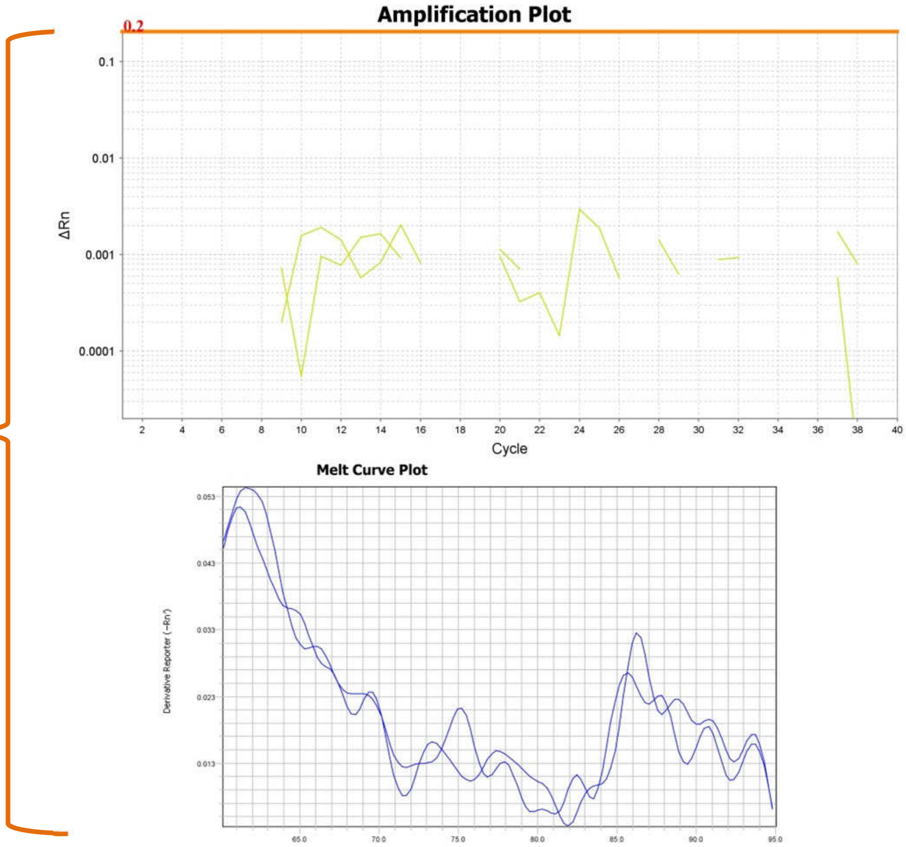

Real-Time PCR for the junction region of cytochrome c oxidase

Cox1_circular RNA3 (ID: Ch1:23865348-23865740)

By divergent primers ◀▶ no. 4 on genomic DNA

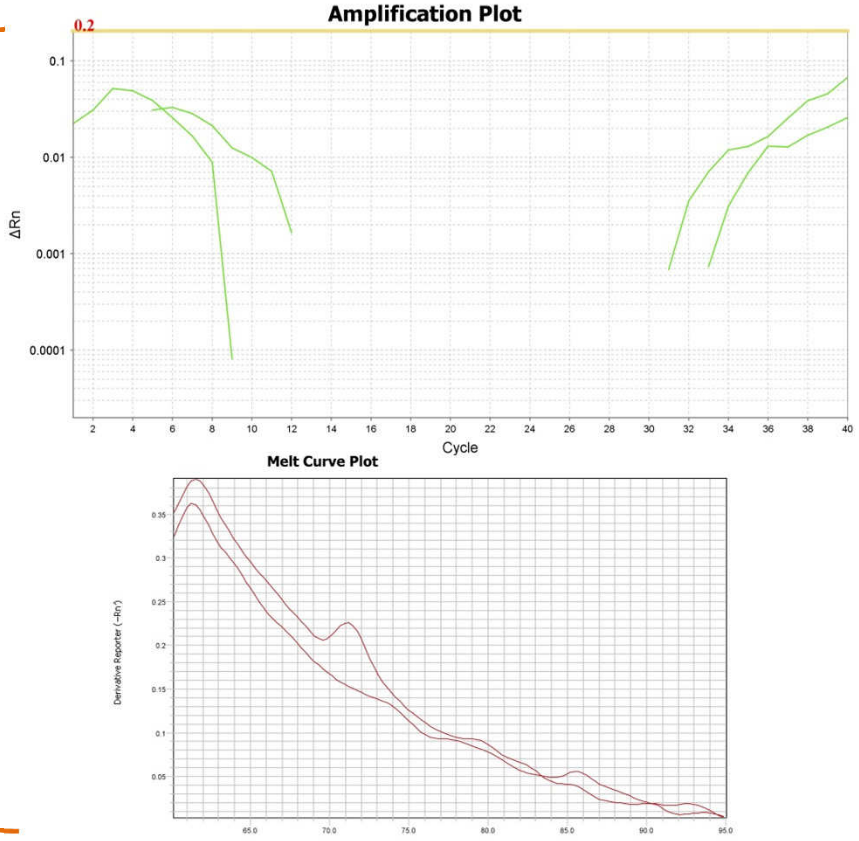

By divergent primers ◀▶ no. 4 on cDNAs

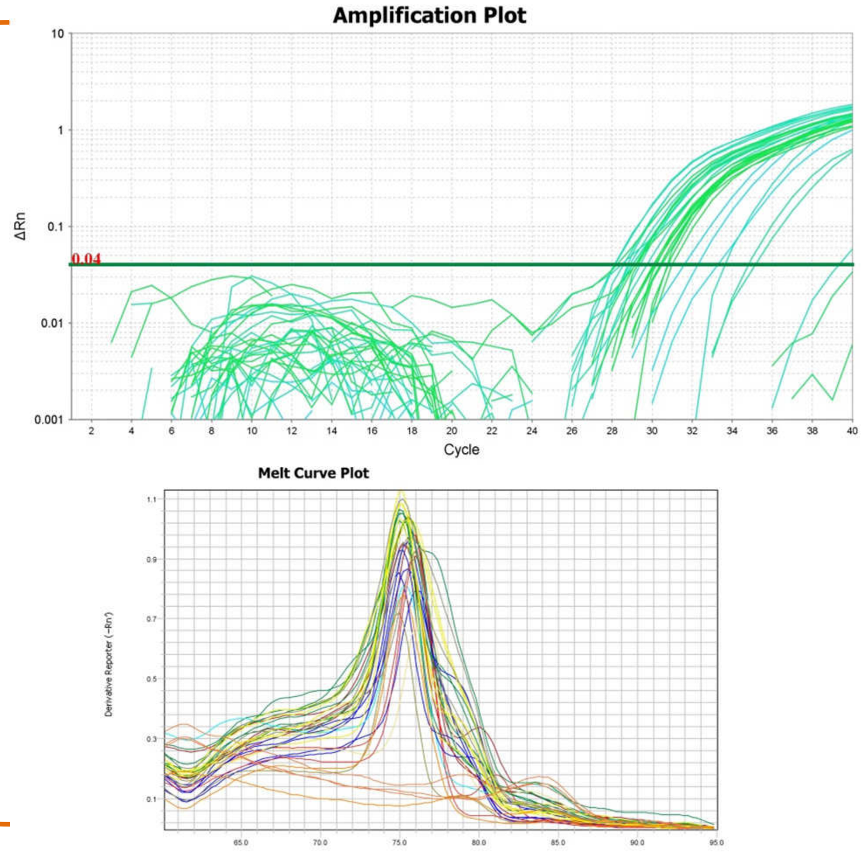

Real-Time PCR for the junction region of cytochrome c oxidase

Cox1_circular RNA3 (ID: Ch1:23865348-23865740**)**

By divergent primers ◀▶ no. 4 with no template

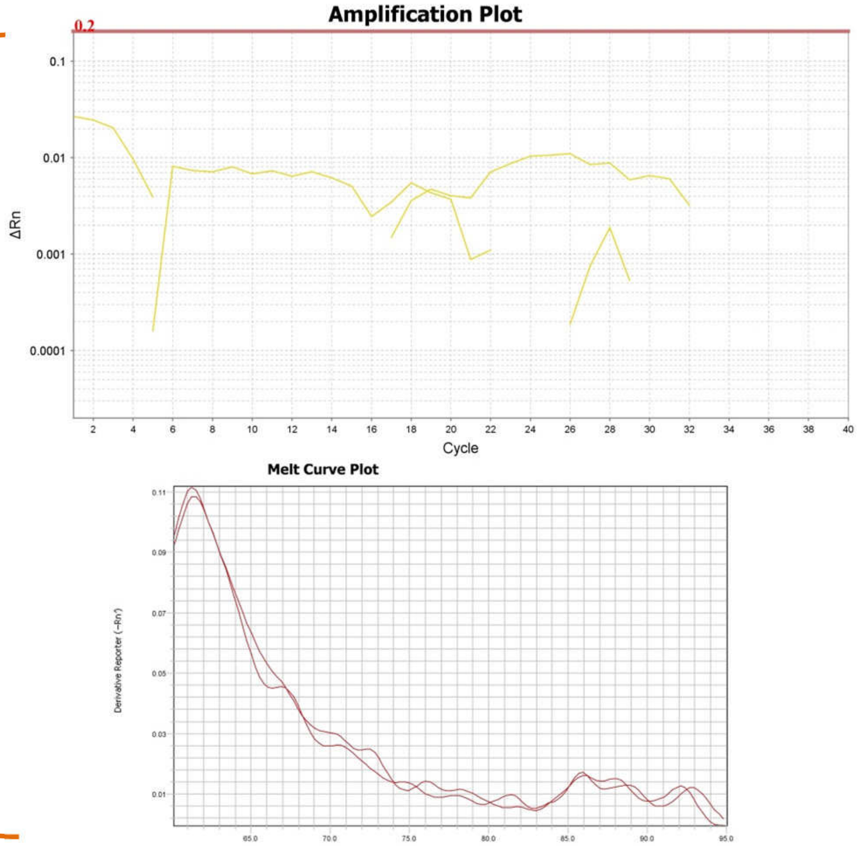

Real-Time PCR for the junction region of cytochrome c oxidase

Cox1_circular RNA2 (ID: Ch1:23865228-23865597)

By divergent primers ◀▶ no. 5 on genomic DNA

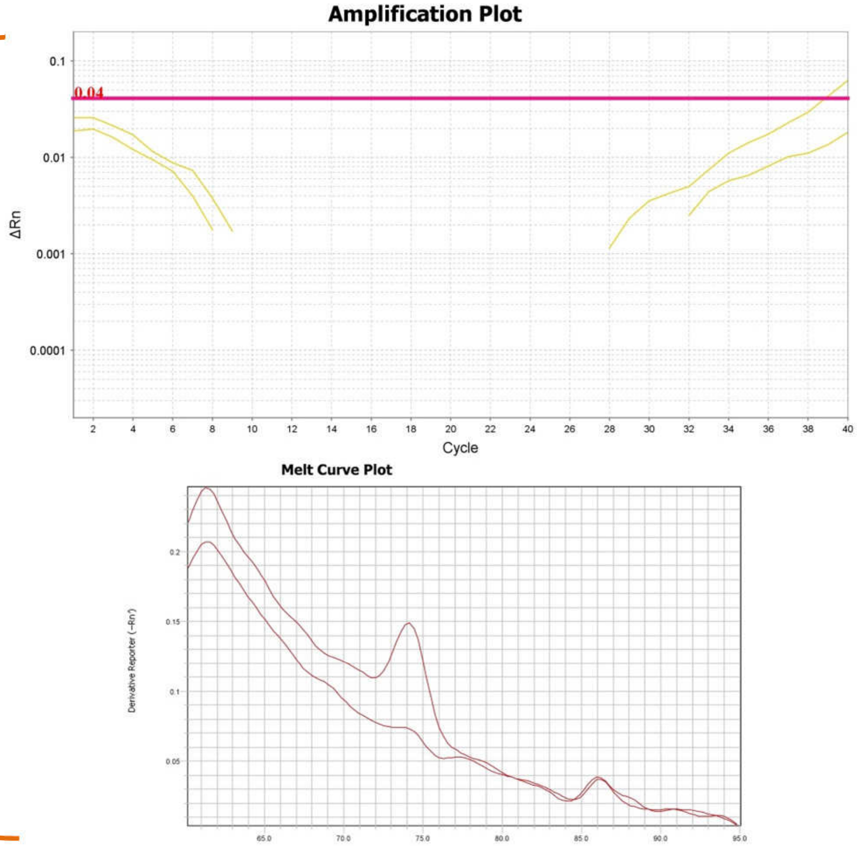

By divergent primers ◀▶ no. 5 on cDNAs

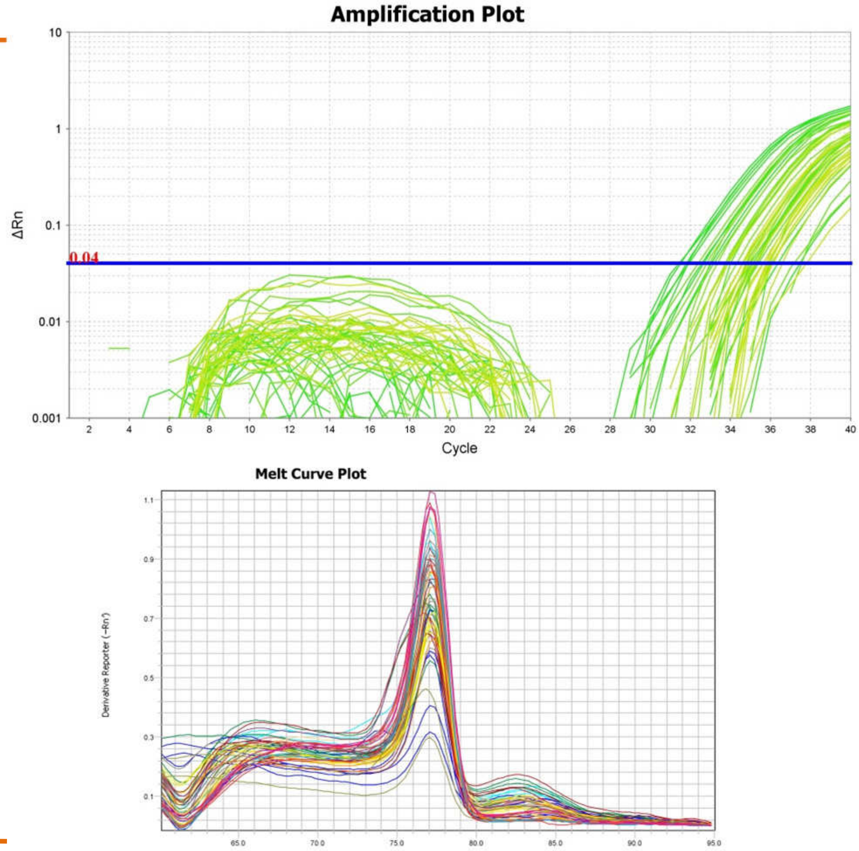

Real-Time PCR for the junction region of cytochrome c oxidase

Cox1_circular RNA2 (ID: Ch1:23865228-23865597**)**

By divergent primers ◀▶ no. 5 with no template

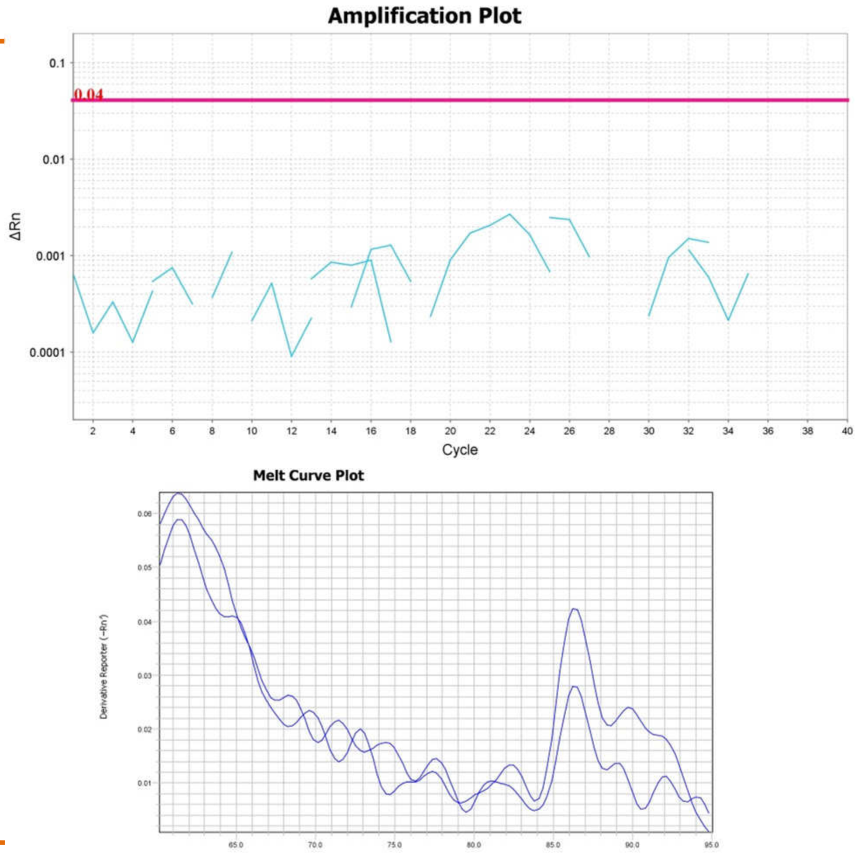

Real-Time PCR for cytochrome c oxidase Cox1

By convergent primers ▶◀ on cDNAs

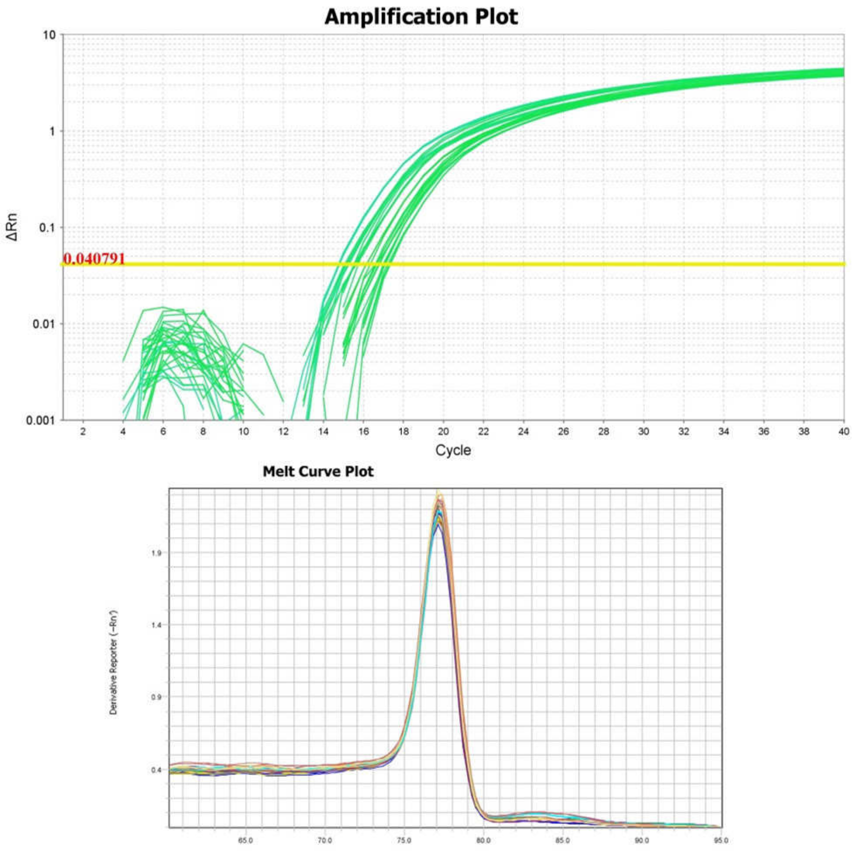

By convergent primers ▶◀ & with no template

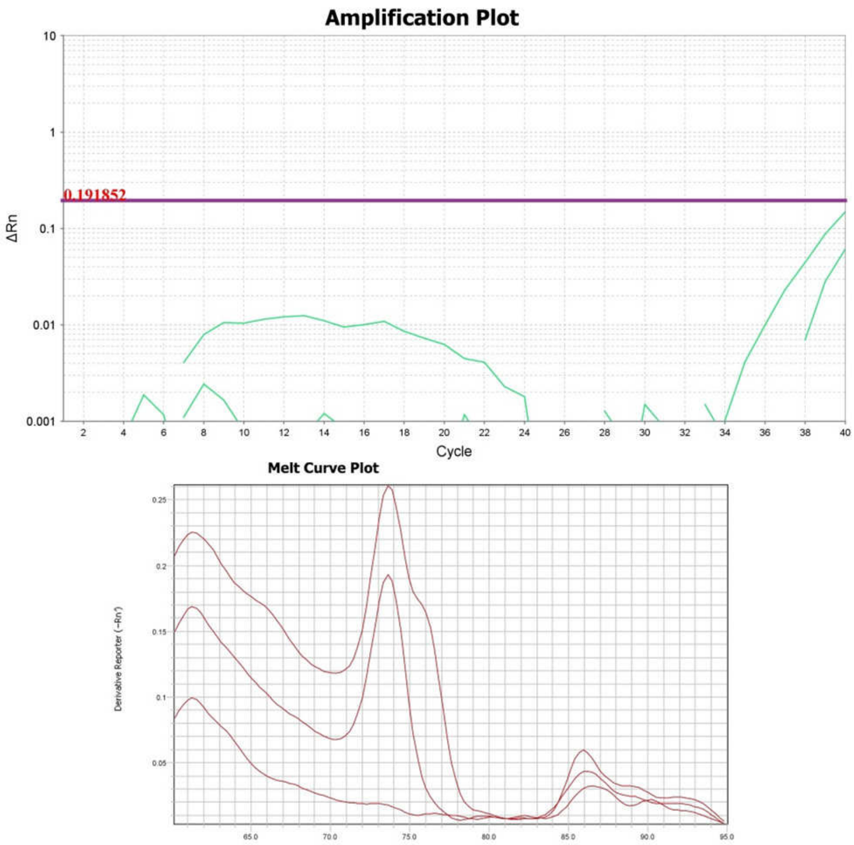

Cystathionine beta-lyase_circular RNA (ID: Ch7:53946083-53946582)

GATAATGCCCAGAAGATTGCTGAATTCTTAGCTTCTCATCCAAAGGTCAAGAAAGTGAATTATGC
TGGACTTCCTGATCATCCGGGCCGATCTTTACACTACTCTCAGGNNNNNNNNNNNN...NNNNNNN
NNNNCAAAGGGAGCAGGCTCTGTCCTCAGTTTTCTAACTGGTTCATTGTCTCTCTCGAAGCATGTT
GTCGAGACAACCAAGTACTTCAACGTAACAGTTAGCTTCG

The nucleotides of junction-region are underlined. The nucleotides of junction-region which are supported by the junction-spaning sequencing reads are shown in red. Introns are not shown if the absence is supported by sequencing reads. In the absence of supporting sequencing reads, the intronic nucletides are shown as N.

Structural relationship between the circular RNA and its parental gene

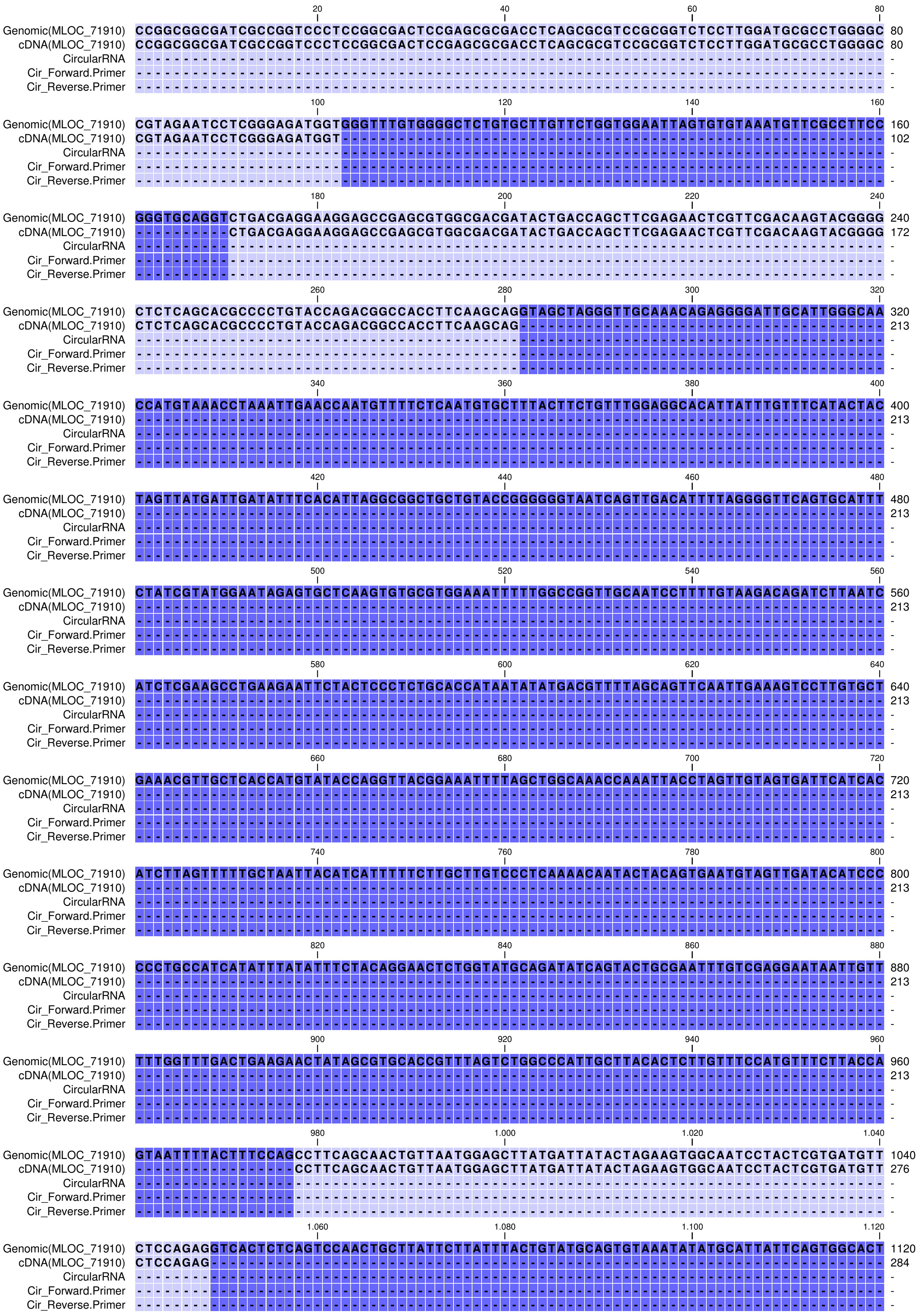
Genomic(MLOC_71910) CCGGCGGCGATCGCCGGTCCCTCCGGCGACTCCGAGCGCGACCTCAGCGCGTCCGCGGTCTCCTTGGATGCGCCTGGGGC 80
cDNA(MLOC_71910) CCGGCGGCGATCGCCGGTCCCTCCGGCGACTCCGAGCGCGACCTCAGCGCGTCCGCGGTCTCCTTGGATGCGCCTGGGGC 80
CircularRNA
Cir_Forward.Primer
Cir_Reverse.Primer
Genomic(MLOC_71910) CGTAGAATCCTCGGGAGATGGTGGGTTTGTGGGGCTCTGTGCTTGTTCTGGTGGAATTAGTGTGTAAATGTTCGCCTTCC 160
cDNA(MLOC_71910) CGTAGAATCCTCGGGAGATGGT 102
Genomic(MLOC_71910) GGGTGCAGGTCTGACGAGGAAGGAGCCGAGCGTGGCGACGATACTGACCAGCTTCGAGAACTCGTTCGACAAGTACGGGG 240
cDNA(MLOC_71910) CTGACGAGGAAGGAGCCGAGCGTGGCGACGATACTGACCAGCTTCGAGAACTCGTTCGACAAGTACGGGG 172
Genomic(MLOC_71910) CTCTCAGCACGCCCCTGTACCAGACGGCCACCTTCAAGCAGGTAGCTAGGGTTGCAAACAGAGGGGATTGCATTGGGCAA 320
cDNA(MLOC_71910) CTCTCAGCACGCCCCTGTACCAGACGGCCACCTTCAAGCAG 213
Genomic(MLOC_71910) CCATGTAAACCTAAATTGAACCAATGTTTTCTCAATGTGCTTTACTTCTGTTTGGAGGCACATTATTTGTTTCATACTAC 400
Genomic(MLOC_71910) TAGTTATGATTGATATTTCACATTAGGCGGCTGCTGTACCGGGGGGTAATCAGTTGACATTTTAGGGGTTCAGTGCATTT 480
Genomic(MLOC_71910) CTATCGTATGGAATAGAGTGCTCAAGTGTGCGTGGAAATTTTTGGCCGGTTGCAATCCTTTTGTAAGACAGATCTTAATC 560
Genomic(MLOC_71910) ATCTCGAAGCCTGAAGAATTCTACTCCCTCTGCACCATAATATATGACGTTTTAGCAGTTCAATTGAAAGTCCTTGTGCT 640
Genomic(MLOC_71910) GAAACGTTGCTCACCATGTATACCAGGTTACGGAAATTTTAGCTGGCAAACCAAATTACCTAGTTGTAGTGATTCATCAC 720
Genomic(MLOC_71910) ATCTTAGTTTTTGCTAATTACATCATTTTTCTTGCTTGTCCCTCAAAACAATACTACAGTGAATGTAGTTGATACATCCC 800
Genomic(MLOC_71910) CCCTGCCATCATATTTATATTTCTACAGGAACTCTGGTATGCAGATATCAGTACTGCGAATTTGTCGAGGAATAATTGTT 880
Genomic(MLOC_71910) TTTGGTTTGACTGAAGAACTATAGCGTGCACCGTTTAGTCTGGCCCATTGCTTACACTCTTGTTTCCATGTTTCTTACCA 960
Genomic(MLOC_71910) GTAATTTTACTTTCCAGCCTTCAGCAACTGTTAATGGAGCTTATGATTATACTAGAAGTGGCAATCCTACTCGTGATGTT 1040
cDNA(MLOC_71910) CCTTCAGCAACTGTTAATGGAGCTTATGATTATACTAGAAGTGGCAATCCTACTCGTGATGTT 276
Genomic(MLOC_71910) CTCCAGAGGTCACTCTCAGTCCAACTGCTTATTCTTATTTACTGTATGCAGTGTAAATATATGCATTATTCAGTGGCACT 1120
cDNA(MLOC_71910) CTCCAGAG 284

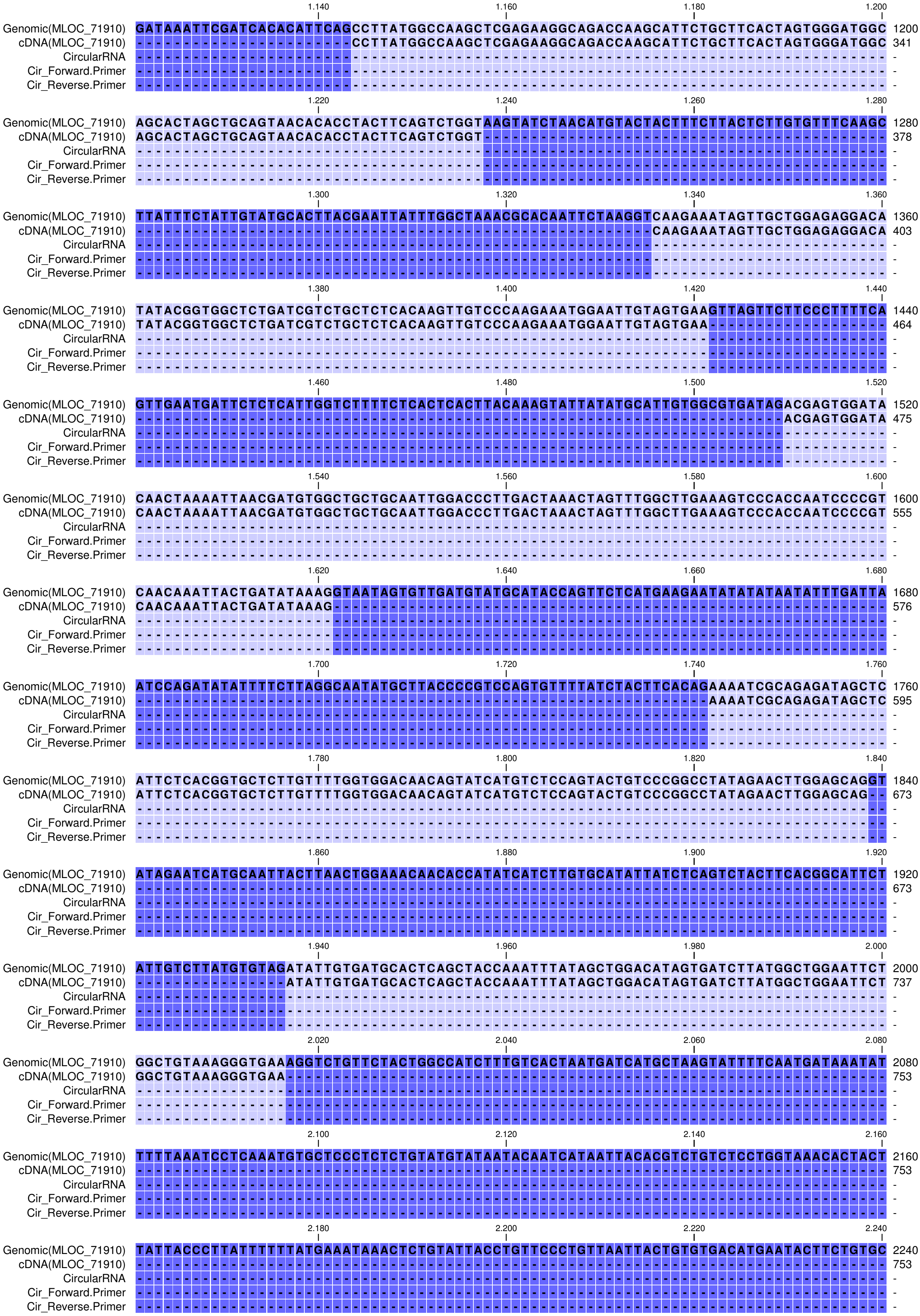
Genomic(MLOC_71910) GATAAATTCGATCACACATTCAGCCTTATGGCCAAGCTCGAGAAGGCAGACCAAGCATTCTGCTTCACTAGTGGGATGGC 1200
cDNA(MLOC_71910) CCTTATGGCCAAGCTCGAGAAGGCAGACCAAGCATTCTGCTTCACTAGTGGGATGGC 341
Genomic(MLOC_71910) AGCACTAGCTGCAGTAACACACCTACTTCAGTCTGGTAAGTATCTAACATGTACTACTTTCTTACTCTTGTGTTTCAAGC 1280
cDNA(MLOC_71910) AGCACTAGCTGCAGTAACACACCTACTTCAGTCTGGT 378
Genomic(MLOC_71910) TTATTTCTATTGTATGCACTTACGAATTATTTGGCTAAACGCACAATTCTAAGGTCAAGAAATAGTTGCTGGAGAGGACA 1360
cDNA(MLOC_71910) CAAGAAATAGTTGCTGGAGAGGACA 403
Genomic(MLOC_71910) TATACGGTGGCTCTGATCGTCTGCTCTCACAAGTTGTCCCAAGAAATGGAATTGTAGTGAAGTTAGTTCTTCCCTTTTCA 1440
cDNA(MLOC_71910) TATACGGTGGCTCTGATCGTCTGCTCTCACAAGTTGTCCCAAGAAATGGAATTGTAGTGAA 464
Genomic(MLOC_71910) GTTGAATGATTCTCTCATTGGTCTTTTCTCACTCACTTACAAAGTATTATATGCATTGTGGCGTGATAGACGAGTGGATA 1520
cDNA(MLOC_71910) ACGAGTGGATA 475
Genomic(MLOC_71910) CAACTAAAATTAACGATGTGGCTGCTGCAATTGGACCCTTGACTAAACTAGTTTGGCTTGAAAGTCCCACCAATCCCCGT 1600
cDNA(MLOC_71910) CAACTAAAATTAACGATGTGGCTGCTGCAATTGGACCCTTGACTAAACTAGTTTGGCTTGAAAGTCCCACCAATCCCCGT 555
Genomic(MLOC_71910) CAACAAATTACTGATATAAAGGTAATAGTGTTGATGTATGCATACCAGTTCTCATGAAGAATATATATAATATTTGATTA 1680
cDNA(MLOC_71910) CAACAAATTACTGATATAAAG 576
Genomic(MLOC_71910) ATCCAGATATATTTTCTTAGGCAATATGCTTACCCCGTCCAGTGTTTTATCTACTTCACAGAAAATCGCAGAGATAGCTC 1760
cDNA(MLOC_71910) AAAATCGCAGAGATAGCTC 595
Genomic(MLOC_71910) ATTCTCACGGTGCTCTTGTTTTGGTGGACAACAGTATCATGTCTCCAGTACTGTCCCGGCCTATAGAACTTGGAGCAGGT 1840
cDNA(MLOC_71910) ATTCTCACGGTGCTCTTGTTTTGGTGGACAACAGTATCATGTCTCCAGTACTGTCCCGGCCTATAGAACTTGGAGCAG 673
Genomic(MLOC_71910) ATAGAATCATGCAATTACTTAACTGGAAACAACACCATATCATCTTGTGCATATTATCTCAGTCTACTTCACGGCATTCT 1920
cDNA(MLOC_71910) 673
Genomic(MLOC_71910) ATTGTCTTATGTGTAGATATTGTGATGCACTCAGCTACCAAATTTATAGCTGGACATAGTGATCTTATGGCTGGAATTCT 2000
cDNA(MLOC_71910) ATATTGTGATGCACTCAGCTACCAAATTTATAGCTGGACATAGTGATCTTATGGCTGGAATTCT 737
Genomic(MLOC_71910) GGCTGTAAAGGGTGAAAGGTCTGTTCTACTGGCCATCTTTGTCACTAATGATCATGCTAAGTATTTTCAATGATAAATAT 2080
cDNA(MLOC_71910) GGCTGTAAAGGGTGAA 753
Genomic(MLOC_71910) TTTTAAATCCTCAAATGTGCTCCCTCTCTGTATGTATAATACAATCATAATTACACGTCTGTCTCCTGGTAAACACTACT 2160
cDNA(MLOC_71910) 753
Genomic(MLOC_71910) TATTACCCTTATTTTTTATGAAATAAACTCTGTATTACCTGTTCCCTGTTAATTACTGTGTGACATGAATACTTCTGTGC 2240
cDNA(MLOC_71910) 753
CircularRNA
Cir_Forward.Primer
Cir_Reverse.Primer

```
                                          2.260                                   2.280                                   2.300                                   2.320
Genomic(MLOC_71910)  TGCTGGCCTAAACACTAACAGGCGGTTTCTCTGTTTTACTGAATAGAGCTACTAAATACCCCTTCAAATTTATAGAGTTA 2320
cDNA(MLOC_71910)     -------------------------------------------------------------------------------- 753
CircularRNA          -------------------------------------------------------------------------------- -
Cir_Forward.Primer   -------------------------------------------------------------------------------- -
Cir_Reverse.Primer   -------------------------------------------------------------------------------- -

                                          2.340                                   2.360                                   2.380                                   2.400
Genomic(MLOC_71910)  ATTGAAGCAGCTATGAACTCTTAAAGTAATACTGTTCCAACTGCTAACAGCTTGGCGAAGGAGATTGCATTTCTACAAAA 2400
cDNA(MLOC_71910)     ------------------------------------------------AGCTTGGCGAAGGAGATTGCATTTCTACAAAA 785
CircularRNA          -------------------------------------------------------------------------------- -
Cir_Forward.Primer   -------------------------------------------------------------------------------- -
Cir_Reverse.Primer   -------------------------------------------------------------------------------- -

                                          2.420                                   2.440                                   2.460                                   2.480
Genomic(MLOC_71910)  CGCCGAAGGGTCAGGTTTGGCGCCTTTTGATTGCTGGCTTTGTTTAAGAGGGATTAAAACCATGGCCTTGCGGGTGGAAA 2480
cDNA(MLOC_71910)     CGCCGAAGGGTCAGGTTTGGCGCCTTTTGATTGCTGGCTTTGTTTAAGAGGGATTAAAACCATGGCCTTGCGGGTGGAAA 865
CircularRNA          -------------------------------------------------------------------------------- -
Cir_Forward.Primer   -------------------------------------------------------------------------------- -
Cir_Reverse.Primer   -------------------------------------------------------------------------------- -

                                          2.500                                   2.520                                   2.540                                   2.560
Genomic(MLOC_71910)  AGCAACAGGTGAAACTTTGTTGTGATACAAATAAATTGTATGCGGAAGTACATACTGGTTATTGAAATGGGAAAGTAGTG 2560
cDNA(MLOC_71910)     AGCAACAGG----------------------------------------------------------------------- 874
CircularRNA          -------------------------------------------------------------------------------- -
Cir_Forward.Primer   -------------------------------------------------------------------------------- -
Cir_Reverse.Primer   -------------------------------------------------------------------------------- -

                                          2.580                                   2.600                                   2.620                                   2.640
Genomic(MLOC_71910)  GCCGAACCCCGCTCGGTGCACCCACGCAAGAAAACGTATCAAAACATTTAAAAAAAAATCTGAAACTTTGTGATAATGAT 2640
cDNA(MLOC_71910)     -------------------------------------------------------------------------------- 874
CircularRNA          -------------------------------------------------------------------------------- -
Cir_Forward.Primer   -------------------------------------------------------------------------------- -
Cir_Reverse.Primer   -------------------------------------------------------------------------------- -

                                          2.660                                   2.680                                   2.700                                   2.720
Genomic(MLOC_71910)  CTTCAACAAATGTTATGGGCACATGCAAAGTTTGGTGGTCAAATAACATTCAAACAGCTCTGTACAAAAAAGACAAAATT 2720
cDNA(MLOC_71910)     -------------------------------------------------------------------------------- 874
CircularRNA          -------------------------------------------------------------------------------- -
Cir_Forward.Primer   -------------------------------------------------------------------------------- -
Cir_Reverse.Primer   -------------------------------------------------------------------------------- -

                                          2.740                                   2.760                                   2.780                                   2.800
Genomic(MLOC_71910)  ACAAAATCTGCTCAAACAGTGCACTTACATTTTGACTAATTTTCAGTGATTTTGTCTTTTTTGTACAGAGCTCCTCGAAT 2800
cDNA(MLOC_71910)     -------------------------------------------------------------------------------- 874
CircularRNA          -------------------------------------------------------------------------------- -
Cir_Forward.Primer   -------------------------------------------------------------------------------- -
Cir_Reverse.Primer   -------------------------------------------------------------------------------- -

                                          2.820                                   2.840                                   2.860                                   2.880
Genomic(MLOC_71910)  GTTATTTGACCACCAAACTTTGCAGGTGCCCATAACATTTGTTGAAGATCATCCCCACAAAGTTTCAGATTTTTATGAAA 2880
cDNA(MLOC_71910)     -------------------------------------------------------------------------------- 874
CircularRNA          -------------------------------------------------------------------------------- -
Cir_Forward.Primer   -------------------------------------------------------------------------------- -
Cir_Reverse.Primer   -------------------------------------------------------------------------------- -

                                          2.900                                   2.920                                   2.940                                   2.960
Genomic(MLOC_71910)  TATTTTGATATGTTTTCTTGCGTGGGTGCACTGAGCAGGGGTGTAGAAAAACCACTCTATTGAAATTTTGGAGGCCTTCA 2960
cDNA(MLOC_71910)     -------------------------------------------------------------------------------- 874
CircularRNA          -------------------------------------------------------------------------------- -
Cir_Forward.Primer   -------------------------------------------------------------------------------- -
Cir_Reverse.Primer   -------------------------------------------------------------------------------- -

                                          2.980                                   3.000                                   3.020                                   3.040
Genomic(MLOC_71910)  TAAAATGCTACTACAATTTTTCTCAGATTTGATTCATTTCATTTTGTTATAGAGTTCAATGTTTGTAGTCATTTTGATTT 3040
cDNA(MLOC_71910)     -------------------------------------------------------------------------------- 874
CircularRNA          -------------------------------------------------------------------------------- -
Cir_Forward.Primer   -------------------------------------------------------------------------------- -
Cir_Reverse.Primer   -------------------------------------------------------------------------------- -

                                          3.060                                   3.080                                   3.100                                   3.120
Genomic(MLOC_71910)  GTGAGGAGTCTGATGTTTACCTTAATATTTGTAGGATAATGCCCAGAAGATTGCTGAATTCTTAGCTTCTCATCCAAAGG 3120
cDNA(MLOC_71910)     -----------------------------------ATAATGCCCAGAAGATTGCTGAATTCTTAGCTTCTCATCCAAAGG 919
CircularRNA          ----------------------------------GATAATGCCCAGAAGATTGCTGAATTCTTAGCTTCTCATCCAAAGG 46
Cir_Forward.Primer   -------------------------------------------------------------------------------- -
Cir_Reverse.Primer   ------------------------------------TAATGCCCAGAAGATTGCTGAATT-------------------- 24

                                          3.140                                   3.160                                   3.180                                   3.200
Genomic(MLOC_71910)  TCAAGAAAGTGAATTATGCTGGACTTCCTGATCATCCGGGCCGATCTTTACACTACTCTCAGGTACTTGTTGGTTTTCAA 3200
cDNA(MLOC_71910)     TCAAGAAAGTGAATTATGCTGGACTTCCTGATCATCCGGGCCGATCTTTACACTACTCTCAGG----------------- 982
CircularRNA          TCAAGAAAGTGAATTATGCTGGACTTCCTGATCATCCGGGCCGATCTTTACACTACTCTCAGGNNNNNNNNNNNNNNNNN 126
Cir_Forward.Primer   -------------------------------------------------------------------------------- -
Cir_Reverse.Primer   -------------------------------------------------------------------------------- 24

                                          3.220                                   3.240                                   3.260                                   3.280
Genomic(MLOC_71910)  GTTGGCTGCATTGAGCAGCTTAAGTGATCCATCAATTTGCAGAGCACCAACGGCGGAGCTTAATTGGGGCCAGCAGGGGC 3280
cDNA(MLOC_71910)     -------------------------------------------------------------------------------- 982
CircularRNA          NNNNNNNNNNNNNNNNNNNNNNNNNNNNNNNNNNNNNNNNNNNNNNNNNNNNNNNNNNNNNNNNNNNNNNNNNNNNNNNN 206
Cir_Forward.Primer   -------------------------------------------------------------------------------- -
Cir_Reverse.Primer   -------------------------------------------------------------------------------- 24

                                          3.300                                   3.320                                   3.340                                   3.360
Genomic(MLOC_71910)  CATGGCCCCCCCAGCCTCACTACAAAACTTGAAGAATTTTTTTGGGGGGCTGTTTAGATTAGAACATTTTTAGCTTTTGG 3360
cDNA(MLOC_71910)     -------------------------------------------------------------------------------- 982
CircularRNA          NNNNNNNNNNNNNNNNNNNNNNNNNNNNNNNNNNNNNNNNNNNNNNNNNNNNNNNNNNNNNNNNNNNNNNNNNNNNNNNN 286
Cir_Forward.Primer   -------------------------------------------------------------------------------- -
Cir_Reverse.Primer   -------------------------------------------------------------------------------- 24
```

```
                      3.380               3.400               3.420               3.440
Genomic(MLOC_71910)   CCCCCCAAACAACATTATTTGCAACCTTGCCCCCCCTGAACATCCTGTCTAGCTCCGCCACTGCAGAGCACCCTTCTAAT 3440
cDNA(MLOC_71910)      -------------------------------------------------------------------------------- 982
CircularRNA           NNNNNNNNNNNNNNNNNNNNNNNNNNNNNNNNNNNNNNNNNNNNNNNNNNNNNNNNNNNNNNNNNNNNNNNNNNNNNNNN 366
Cir_Forward.Primer    -------------------------------------------------------------------------------- -
Cir_Reverse.Primer    -------------------------------------------------------------------------------- 24

                      3.460               3.480               3.500               3.520
Genomic(MLOC_71910)   ATGTTTTCCCTTCCATTTGCTTCCTGTGCAGGCAAAGGGAGCAGGCTCTGTCCTCAGTTTTCTAACTGGTTCATTGTCTC 3520
cDNA(MLOC_71910)      --------------------------------CAAAGGGAGCAGGCTCTGTCCTCAGTTTTCTAACTGGTTCATTGTCTC 1030
CircularRNA           NNNNNNNNNNNNNNNNNNNNNNNNNNNNNNNNCAAAGGGAGCAGGCTCTGTCCTCAGTTTTCTAACTGGTTCATTGTCTC 446
Cir_Forward.Primer    ----------------------------------------------------CTCAGTTTTCTAACTGGTTCATTGTCT- 27
Cir_Reverse.Primer    -------------------------------------------------------------------------------- 24

                      3.540               3.560               3.580               3.600
Genomic(MLOC_71910)   TCTCGAAGCATGTTGTCGAGACAACCAAGTACTTCAACGTAACAGTTAGCTTCGGTAAGCATTCTTATTGACCTGTTCCA 3600
cDNA(MLOC_71910)      TCTCGAAGCATGTTGTCGAGACAACCAAGTACTTCAACGTAACAGTTAGCTTCGG------------------------- 1085
CircularRNA           TCTCGAAGCATGTTGTCGAGACAACCAAGTACTTCAACGTAACAGTTAGCTTCG-------------------------- 500
Cir_Forward.Primer    -------------------------------------------------------------------------------- 27
Cir_Reverse.Primer    -------------------------------------------------------------------------------- 24

                      3.620               3.640               3.660               3.680
Genomic(MLOC_71910)   CCATCCTTTCGGAGTCACCTTGGCCTTGCTCATCTGTTTTATCAGCCAACACATTCCATTTAGAAATCCTCCACAAAACC 3680
cDNA(MLOC_71910)      -------------------------------------------------------------------------------- 1085
CircularRNA           -------------------------------------------------------------------------------- 500
Cir_Forward.Primer    -------------------------------------------------------------------------------- 27
Cir_Reverse.Primer    -------------------------------------------------------------------------------- 24

                      3.700               3.720               3.740               3.760
Genomic(MLOC_71910)   TATCCATTAAAAATAACGCGCACCGATCATTTCTGTACCAGTTTCTTAGCTGGATACTTTTCCTGTGAAACTATGCTCAT 3760
cDNA(MLOC_71910)      -------------------------------------------------------------------------------- 1085
CircularRNA           -------------------------------------------------------------------------------- 500
Cir_Forward.Primer    -------------------------------------------------------------------------------- 27
Cir_Reverse.Primer    -------------------------------------------------------------------------------- 24

                      3.780               3.800               3.820               3.840
Genomic(MLOC_71910)   TCTTGCTCACCCAATCTACGTGCCTATGCAGGAAGCGTGAAGTCGCTCATAAGCTTGCCCTGCTTCATGTCGCACGCGAG 3840
cDNA(MLOC_71910)      --------------------------------AAGCGTGAAGTCGCTCATAAGCTTGCCCTGCTTCATGTCGCACGCGAG 1133
CircularRNA           -------------------------------------------------------------------------------- 500
Cir_Forward.Primer    -------------------------------------------------------------------------------- 27
Cir_Reverse.Primer    -------------------------------------------------------------------------------- 24

                      3.860               3.880               3.900               3.920
Genomic(MLOC_71910)   CATCCCTTCCTTGGTACGAGAGGAGCGTGGGTTGACTGATGATCTAGTGCGGATATCCGTGGGCATTGAGGACGTGGACG 3920
cDNA(MLOC_71910)      CATCCCTTCCTTGGTACGAGAGGAGCGTGGGTTGACTGATGATCTAGTGCGGATATCCGTGGGCATTGAGGACGTGGACG 1213
CircularRNA           -------------------------------------------------------------------------------- 500
Cir_Forward.Primer    -------------------------------------------------------------------------------- 27
Cir_Reverse.Primer    -------------------------------------------------------------------------------- 24

                      3.940               3.960               3.980               4.000
Genomic(MLOC_71910)   ACCTCATAGCTGATCTTGATTACGCGCTCAGGTCCGGCCCAGCATAGAGCATACAAAATCTGCACTTTGGCGCTCAGGGT 4000
cDNA(MLOC_71910)      ACCTCATAGCTGATCTTGATTACGCGCTCAGGTCCGGCCCAGCATAGAGCATACAAAATCTGCACTTTGGCGCTCAGGGT 1293
CircularRNA           -------------------------------------------------------------------------------- 500
Cir_Forward.Primer    -------------------------------------------------------------------------------- 27
Cir_Reverse.Primer    -------------------------------------------------------------------------------- 24

                      4.020               4.040               4.060               4.080
Genomic(MLOC_71910)   TCCGGTTCATCAAGTTGTAGATGCGATTTGAATTGGTGATTCATTTGTTAAACTGCTACAGTAATAATAAACTTCTGCAT 4080
cDNA(MLOC_71910)      TCCGGTTCATCAAGTTGTAGATGCGATTTGAATTGGTGATTCATTTGTTAAACTGCTACAGTAATAATAAACTTCTGCAT 1373
CircularRNA           -------------------------------------------------------------------------------- 500
Cir_Forward.Primer    -------------------------------------------------------------------------------- 27
Cir_Reverse.Primer    -------------------------------------------------------------------------------- 24

                      4.100               4.120               4.140               4.160
Genomic(MLOC_71910)   GAGTATTTTCTAAAATGATGAGCATGCGGTCGTATGTGTTGCTCGTCACAGGCGTCAACAGAAAAACCTTGAGGCCAACT 4160
cDNA(MLOC_71910)      GAGTATTTTCTAAAATGATGAGCATGCGGTCGTATGTGTTGCTCGTCACAGGCGTCAACAGAAAAACCTTGAGGCCAACT 1453
CircularRNA           -------------------------------------------------------------------------------- 500
Cir_Forward.Primer    -------------------------------------------------------------------------------- 27
Cir_Reverse.Primer    -------------------------------------------------------------------------------- 24

                      4.180
Genomic(MLOC_71910)   GACAAGTAACAACATGCATAAACTTCACAACATCGAA 4197
cDNA(MLOC_71910)      GACAAGTAACAACATGCATAAACTTCACAACATCGAA 1490
CircularRNA           ------------------------------------- 500
Cir_Forward.Primer    ------------------------------------- 27
Cir_Reverse.Primer    ------------------------------------- 24
```

Real-Time PCR for the junction region of cystathionine beta-lyase_circular RNA (ID: Ch7:53946083-53946582)

By divergent primers ◀▶ on genomic DNA

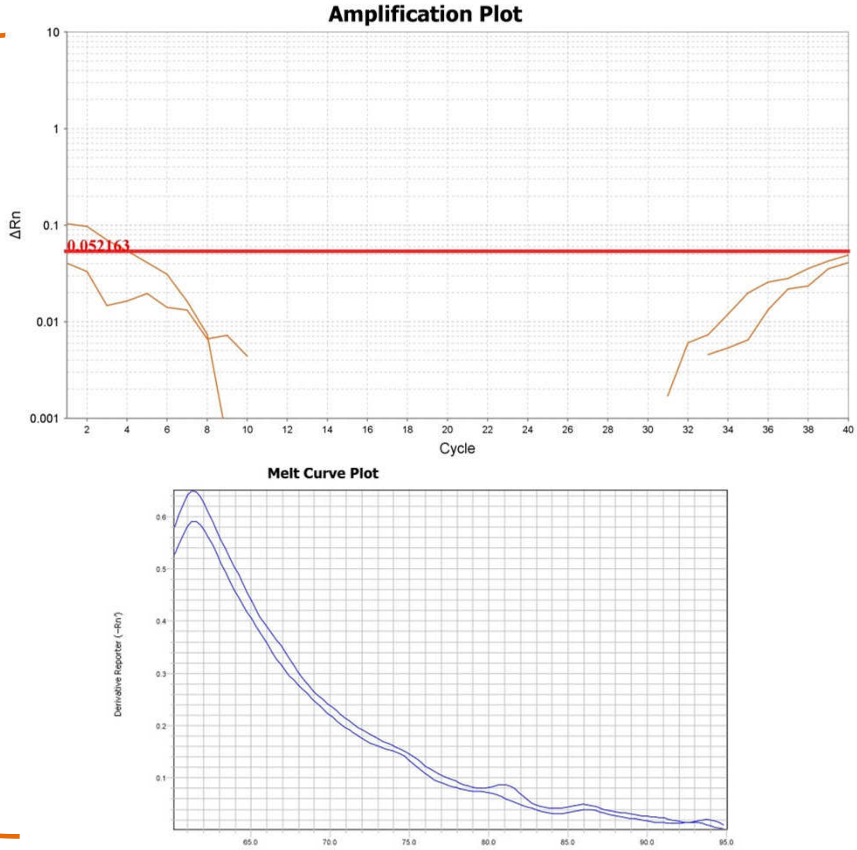

By divergent primers ◀▶ on cDNAs

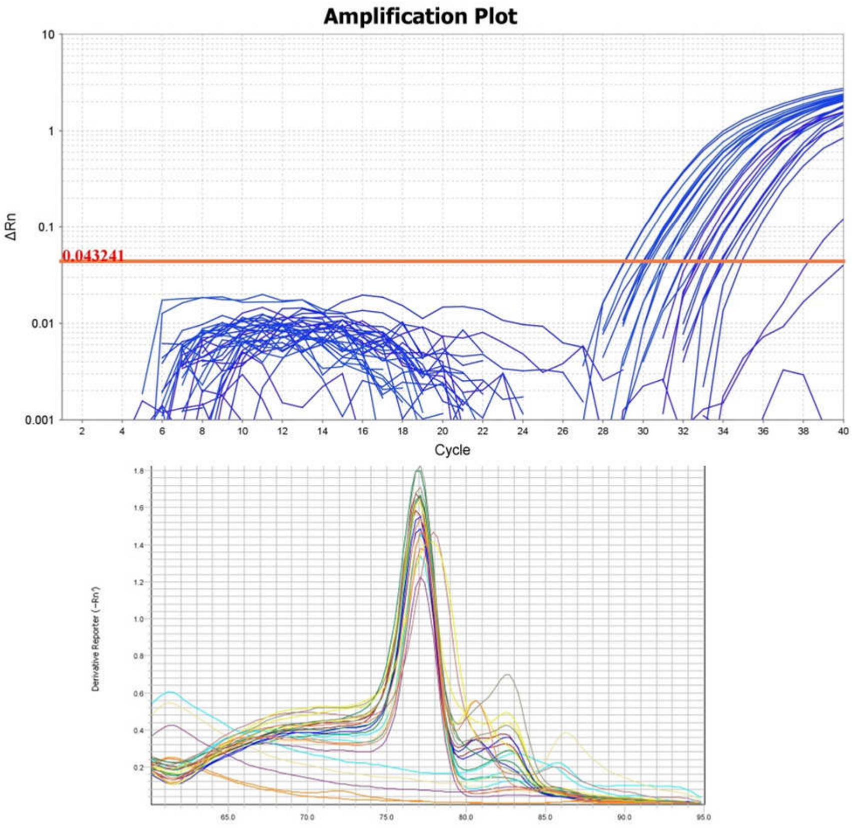

Real-Time PCR for the junction region of cystathionine beta-lyase_circular RNA (ID: Ch7:53946083-53946582)

By divergent primers ◀▶ & with no template

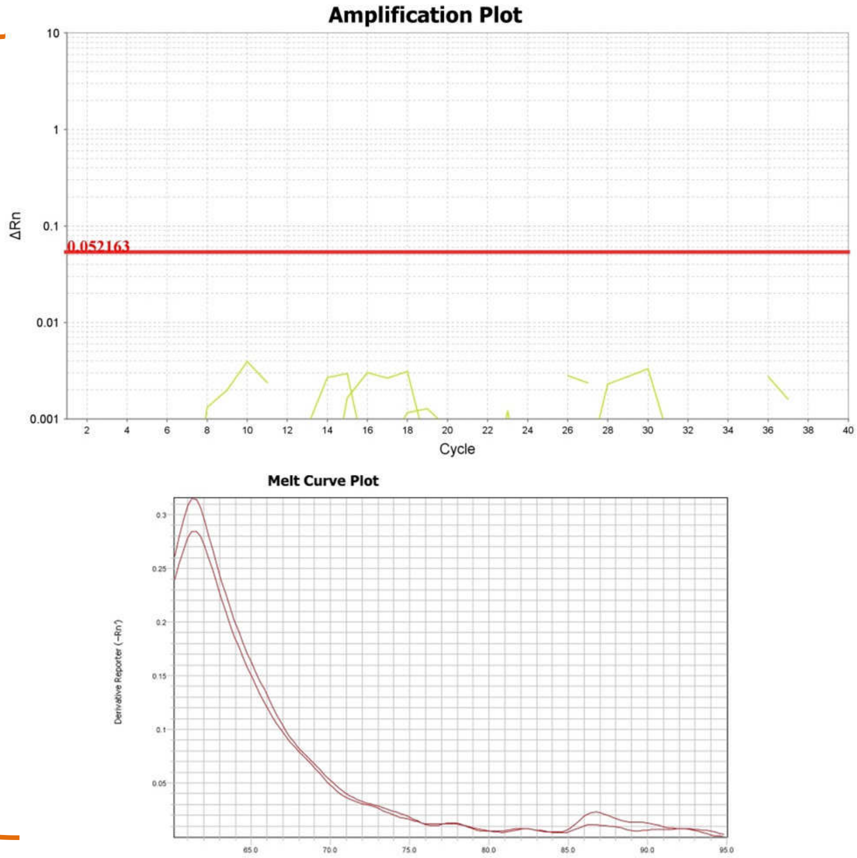

Real-Time PCR for cystathionine beta-lyase

By convergent primers ▶◀ on cDNAs

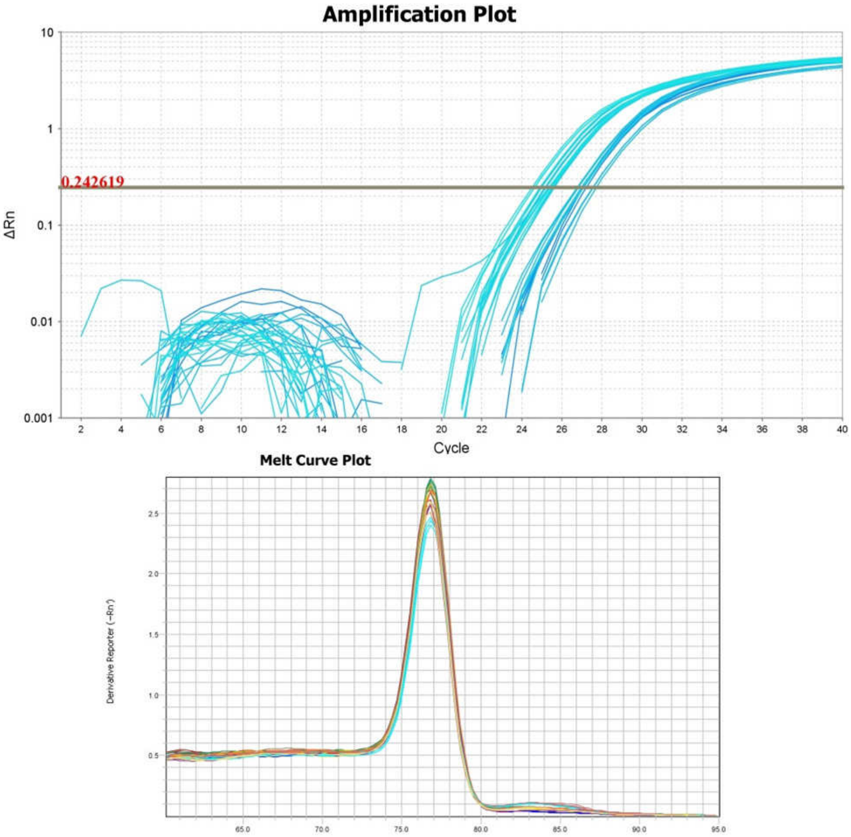

By convergent primers ▶◀ & with no template

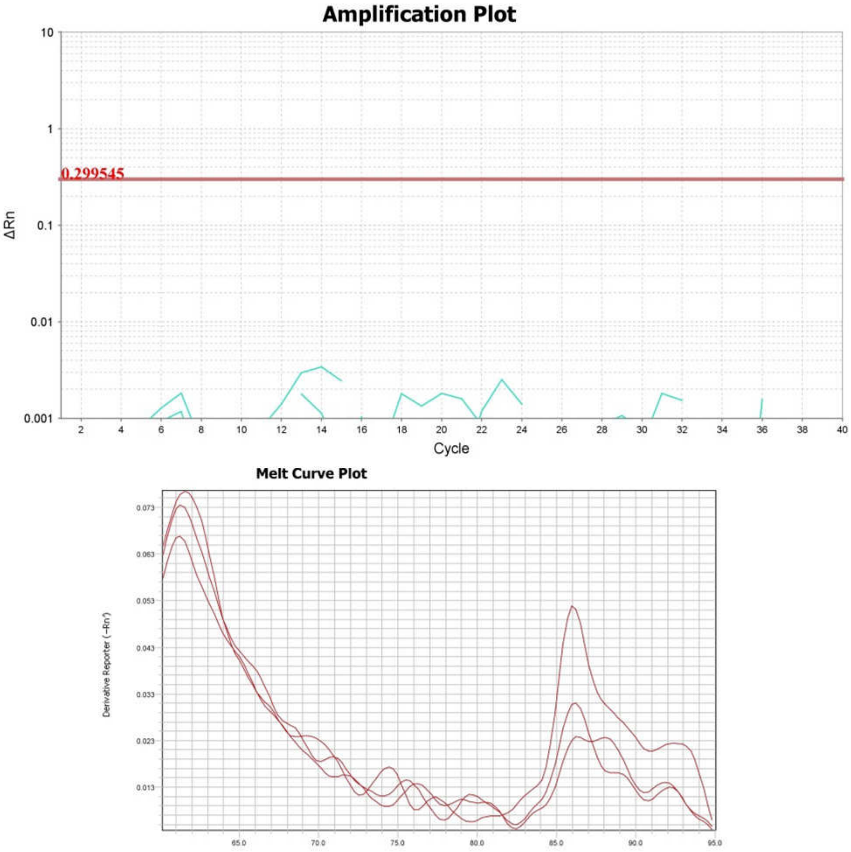

BZip11_circular RNA (ID: Ch6:52088926-52089401)

GTCCCCCTCCTCCTCCGCTCCCTCCCCTGCACCGCAGCCAGAGAGACAGAGTCCGTCTAGAGA
GAGAGAGAGAGAGAGAGAGAGAAGCAAGCTAGCAGTGAGCTGAGAGGTCGCCCGTCCGTCGGT
CCATCCATCCATTCTCCGGCGGGTGGTGGAGCTCTCTCTTCTTCCAGGAGGCAACCAAAGAAAGC
GGAGCGCACATGCTTCCCCGGGTACGCCTCAGAGTCCCCCGATGTCGCCGGTGATCAGCGAGAT
CCTCCTCTCCGGGTTTATGATCAACTCCACACTCCGGCGCCGAACCCACCTCGTCCAGTCATTCT
CCGTCGTCTTCCTCTACTGGTTCTATGTCTTCTCATAAACCACTCGCCCCCACACACCTCCTCCCC
CTCTCCCTCCTCCTCCTTTAGCCCTCCTCCTCCTTCCATCCTCTCCTCGTCCCT**CCCACTCGCGC**
GCTTACTCTCTCGTCGTCGTCACTC

The nucleotides of junction-region are underlined. The nucleotides of junction-region which are supported by the junction-spaning sequencing reads are shown in red. Introns are not shown if the absence is supported by sequencing reads. In the absence of supporting sequencing reads, the intronic nucletides are shown as N.

Structural relationship between the circular RNA and its parental gene

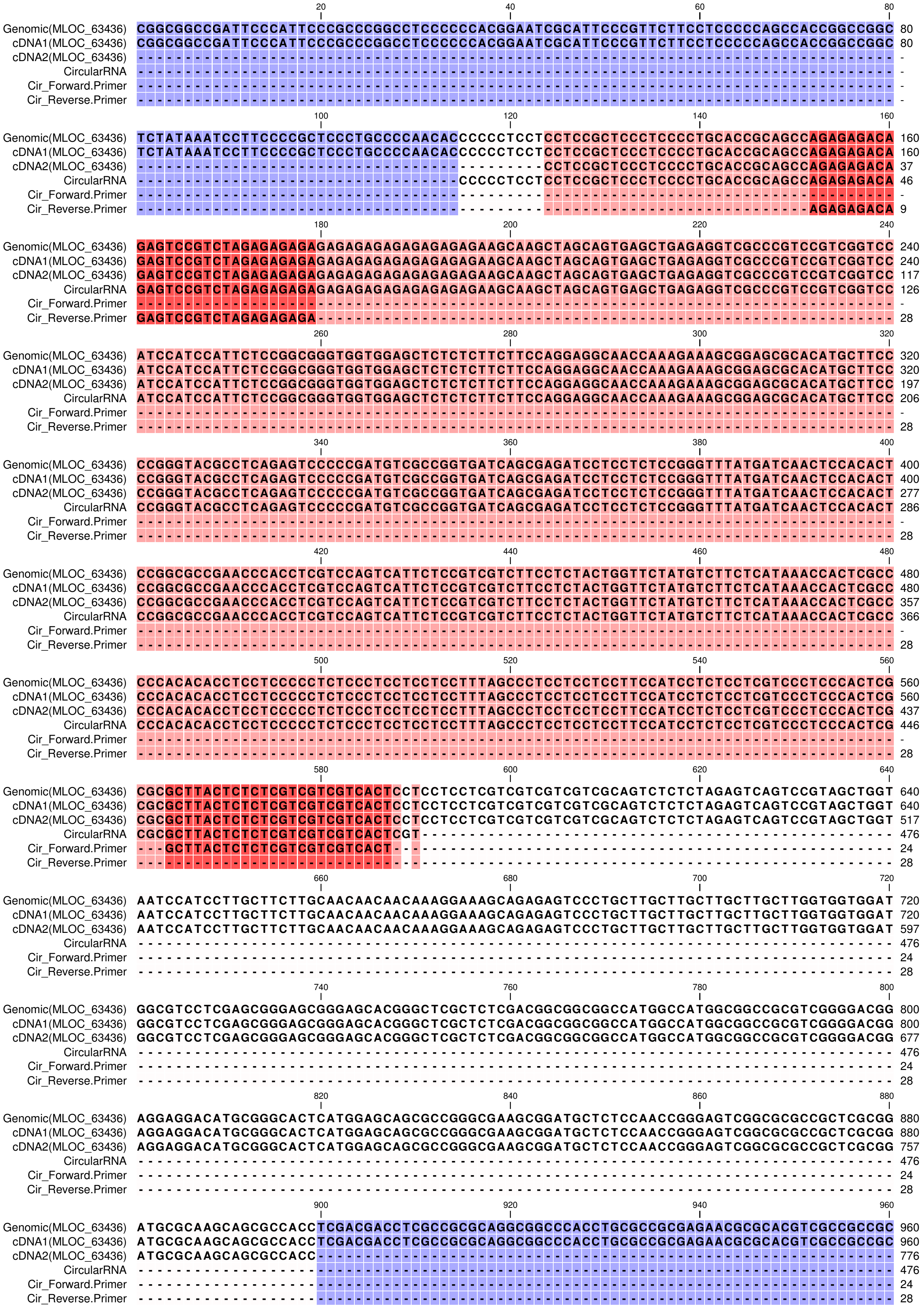

20 40 60 80
Genomic(MLOC_63436) CGGCGGCCGATTCCCATTCCCGCCCGGCCTCCCCCCACGGAATCGCATTCCCGTTCTTCCTCCCCCAGCCACCGGCCGGC 80
cDNA1(MLOC_63436) CGGCGGCCGATTCCCATTCCCGCCCGGCCTCCCCCCACGGAATCGCATTCCCGTTCTTCCTCCCCCAGCCACCGGCCGGC 80
cDNA2(MLOC_63436) -- -
CircularRNA -- -
Cir_Forward.Primer -- -
Cir_Reverse.Primer -- -
100 120 140 160
Genomic(MLOC_63436) TCTATAAATCCTTCCCCGCTCCCTGCCCCAACACCCCCCTCCTCCTCCGCTCCCTCCCCTGCACCGCAGCCAGAGAGACA 160
cDNA1(MLOC_63436) TCTATAAATCCTTCCCCGCTCCCTGCCCCAACACCCCCCTCCTCCTCCGCTCCCTCCCCTGCACCGCAGCCAGAGAGACA 160
cDNA2(MLOC_63436) ---CCTCCGCTCCCTCCCCTGCACCGCAGCCAGAGAGACA 37
CircularRNA ----------------------------------CCCCCTCCTCCTCCGCTCCCTCCCCTGCACCGCAGCCAGAGAGACA 46
Cir_Forward.Primer -- -
Cir_Reverse.Primer ---AGAGAGACA 9
180 200 220 240
Genomic(MLOC_63436) GAGTCCGTCTAGAGAGAGAGAGAGAGAGAGAGAGAGAGAAGCAAGCTAGCAGTGAGCTGAGAGGTCGCCCGTCCGTCGGTCC 240
cDNA1(MLOC_63436) GAGTCCGTCTAGAGAGAGAGAGAGAGAGAGAGAGAGAGAAGCAAGCTAGCAGTGAGCTGAGAGGTCGCCCGTCCGTCGGTCC 240
cDNA2(MLOC_63436) GAGTCCGTCTAGAGAGAGAGAGAGAGAGAGAGAGAGAGAAGCAAGCTAGCAGTGAGCTGAGAGGTCGCCCGTCCGTCGGTCC 117
CircularRNA GAGTCCGTCTAGAGAGAGAGAGAGAGAGAGAGAGAGAGAAGCAAGCTAGCAGTGAGCTGAGAGGTCGCCCGTCCGTCGGTCC 126
Cir_Forward.Primer -- -
Cir_Reverse.Primer GAGTCCGTCTAGAGAGAGA--- 28
260 280 300 320
Genomic(MLOC_63436) ATCCATCCATTCTCCGGCGGGTGGTGGAGCTCTCTCTTCTTCCAGGAGGCAACCAAAGAAAGCGGAGCGCACATGCTTCC 320
cDNA1(MLOC_63436) ATCCATCCATTCTCCGGCGGGTGGTGGAGCTCTCTCTTCTTCCAGGAGGCAACCAAAGAAAGCGGAGCGCACATGCTTCC 320
cDNA2(MLOC_63436) ATCCATCCATTCTCCGGCGGGTGGTGGAGCTCTCTCTTCTTCCAGGAGGCAACCAAAGAAAGCGGAGCGCACATGCTTCC 197
CircularRNA ATCCATCCATTCTCCGGCGGGTGGTGGAGCTCTCTCTTCTTCCAGGAGGCAACCAAAGAAAGCGGAGCGCACATGCTTCC 206
Cir_Forward.Primer -- -
Cir_Reverse.Primer -- 28
340 360 380 400
Genomic(MLOC_63436) CCGGGTACGCCTCAGAGTCCCCCGATGTCGCCGGTGATCAGCGAGATCCTCCTCTCCGGGTTTATGATCAACTCCACACT 400
cDNA1(MLOC_63436) CCGGGTACGCCTCAGAGTCCCCCGATGTCGCCGGTGATCAGCGAGATCCTCCTCTCCGGGTTTATGATCAACTCCACACT 400
cDNA2(MLOC_63436) CCGGGTACGCCTCAGAGTCCCCCGATGTCGCCGGTGATCAGCGAGATCCTCCTCTCCGGGTTTATGATCAACTCCACACT 277
CircularRNA CCGGGTACGCCTCAGAGTCCCCCGATGTCGCCGGTGATCAGCGAGATCCTCCTCTCCGGGTTTATGATCAACTCCACACT 286
Cir_Forward.Primer -- -
Cir_Reverse.Primer -- 28
420 440 460 480
Genomic(MLOC_63436) CCGGCGCCGAACCCACCTCGTCCAGTCATTCTCCGTCGTCTTCCTCTACTGGTTCTATGTCTTCTCATAAACCACTCGCC 480
cDNA1(MLOC_63436) CCGGCGCCGAACCCACCTCGTCCAGTCATTCTCCGTCGTCTTCCTCTACTGGTTCTATGTCTTCTCATAAACCACTCGCC 480
cDNA2(MLOC_63436) CCGGCGCCGAACCCACCTCGTCCAGTCATTCTCCGTCGTCTTCCTCTACTGGTTCTATGTCTTCTCATAAACCACTCGCC 357
CircularRNA CCGGCGCCGAACCCACCTCGTCCAGTCATTCTCCGTCGTCTTCCTCTACTGGTTCTATGTCTTCTCATAAACCACTCGCC 366
Cir_Forward.Primer -- -
Cir_Reverse.Primer -- 28
500 520 540 560
Genomic(MLOC_63436) CCCACACACCTCCTCCCCCTCTCCCTCCTCCTCCTTTAGCCCTCCTCCTCCTTCCATCCTCTCCTCGTCCCTCCCACTCG 560
cDNA1(MLOC_63436) CCCACACACCTCCTCCCCCTCTCCCTCCTCCTCCTTTAGCCCTCCTCCTCCTTCCATCCTCTCCTCGTCCCTCCCACTCG 560
cDNA2(MLOC_63436) CCCACACACCTCCTCCCCCTCTCCCTCCTCCTCCTTTAGCCCTCCTCCTCCTTCCATCCTCTCCTCGTCCCTCCCACTCG 437
CircularRNA CCCACACACCTCCTCCCCCTCTCCCTCCTCCTCCTTTAGCCCTCCTCCTCCTTCCATCCTCTCCTCGTCCCTCCCACTCG 446
Cir_Forward.Primer -- -
Cir_Reverse.Primer -- 28
580 600 620 640
Genomic(MLOC_63436) CGCGCTTACTCTCTCGTCGTCGTCACTCCTCCTCCTCGTCGTCGTCGTCGCAGTCTCTCTAGAGTCAGTCCGTAGCTGGT 640
cDNA1(MLOC_63436) CGCGCTTACTCTCTCGTCGTCGTCACTCCTCCTCCTCGTCGTCGTCGTCGCAGTCTCTCTAGAGTCAGTCCGTAGCTGGT 640
cDNA2(MLOC_63436) CGCGCTTACTCTCTCGTCGTCGTCACTCCTCCTCCTCGTCGTCGTCGTCGCAGTCTCTCTAGAGTCAGTCCGTAGCTGGT 517
CircularRNA CGCGCTTACTCTCTCGTCGTCGTCACTCGT-- 476
Cir_Forward.Primer ---GCTTACTCTCTCGTCGTCGTCACT--- 24
Cir_Reverse.Primer -- 28
660 680 700 720
Genomic(MLOC_63436) AATCCATCCTTGCTTCTTGCAACAACAACAAAGGAAAGCAGAGAGTCCCTGCTTGCTTGCTTGCTTGCTTGGTGGTGGAT 720
cDNA1(MLOC_63436) AATCCATCCTTGCTTCTTGCAACAACAACAAAGGAAAGCAGAGAGTCCCTGCTTGCTTGCTTGCTTGCTTGGTGGTGGAT 720
cDNA2(MLOC_63436) AATCCATCCTTGCTTCTTGCAACAACAACAAAGGAAAGCAGAGAGTCCCTGCTTGCTTGCTTGCTTGCTTGGTGGTGGAT 597
CircularRNA -- 476
Cir_Forward.Primer -- 24
Cir_Reverse.Primer -- 28
740 760 780 800
Genomic(MLOC_63436) GGCGTCCTCGAGCGGGAGCGGGAGCACGGGCTCGCTCTCGACGGCGGCGGCCATGGCCATGGCGGCCGCGTCGGGGACGG 800
cDNA1(MLOC_63436) GGCGTCCTCGAGCGGGAGCGGGAGCACGGGCTCGCTCTCGACGGCGGCGGCCATGGCCATGGCGGCCGCGTCGGGGACGG 800
cDNA2(MLOC_63436) GGCGTCCTCGAGCGGGAGCGGGAGCACGGGCTCGCTCTCGACGGCGGCGGCCATGGCCATGGCGGCCGCGTCGGGGACGG 677
CircularRNA -- 476
Cir_Forward.Primer -- 24
Cir_Reverse.Primer -- 28
820 840 860 880
Genomic(MLOC_63436) AGGAGGACATGCGGGCACTCATGGAGCAGCGCCGGGCGAAGCGGATGCTCTCCAACCGGGAGTCGGCGCGCCGCTCGCGG 880
cDNA1(MLOC_63436) AGGAGGACATGCGGGCACTCATGGAGCAGCGCCGGGCGAAGCGGATGCTCTCCAACCGGGAGTCGGCGCGCCGCTCGCGG 880
cDNA2(MLOC_63436) AGGAGGACATGCGGGCACTCATGGAGCAGCGCCGGGCGAAGCGGATGCTCTCCAACCGGGAGTCGGCGCGCCGCTCGCGG 757
CircularRNA -- 476
Cir_Forward.Primer -- 24
Cir_Reverse.Primer -- 28
900 920 940 960
Genomic(MLOC_63436) ATGCGCAAGCAGCGCCACCTCGACGACCTCGCCGCGCAGGCGGCCCACCTGCGCCGCGAGAACGCGCACGTCGCCGCCGC 960
cDNA1(MLOC_63436) ATGCGCAAGCAGCGCCACCTCGACGACCTCGCCGCGCAGGCGGCCCACCTGCGCCGCGAGAACGCGCACGTCGCCGCCGC 960
cDNA2(MLOC_63436) ATGCGCAAGCAGCGCCACC--- 776
CircularRNA -- 476
Cir_Forward.Primer -- 24
Cir_Reverse.Primer -- 28

```
                                 980                 1.000               1.020               1.040
Genomic(MLOC_63436)  GCTCGGGCTCACAGCGCGGGGCCTCCTCGCCGTCGACGCCGAGAACGCCGTCCTCCGCACCCAGGCCGCCGAGCTCGCCG 1040
cDNA1(MLOC_63436)    GCTCGGGCTCACAGCGCGGGGCCTCCTCGCCGTCGACGCCGAGAACGCCGTCCTCCGCACCCAGGCCGCCGAGCTCGCCG 1040
cDNA2(MLOC_63436)    -------------------------------------------------------------------------------- 776
CircularRNA          -------------------------------------------------------------------------------- 476
Cir_Forward.Primer   -------------------------------------------------------------------------------- 24
Cir_Reverse.Primer   -------------------------------------------------------------------------------- 28

                                 1.060               1.080               1.100               1.120
Genomic(MLOC_63436)  CGCGCCTCCAGTCGCTCAACGACATCATCGCCTGCATGAGCGCCAGCAACAACACCGCAGCCGCCGTCGCGCTCACCGTC 1120
cDNA1(MLOC_63436)    CGCGCCTCCAGTCGCTCAACGACATCATCGCCTGCATGAGCGCCAGCAACAACACCGCAGCCGCCGTCGCGCTCACCGTC 1120
cDNA2(MLOC_63436)    -------------------------------------------------------------------------------- 776
CircularRNA          -------------------------------------------------------------------------------- 476
Cir_Forward.Primer   -------------------------------------------------------------------------------- 24
Cir_Reverse.Primer   -------------------------------------------------------------------------------- 28

                                 1.140               1.160               1.180               1.200
Genomic(MLOC_63436)  GCCGCGGCAGCCACCGCCACCGCCACCGACCCGTTCGGCTTCGACGGCCCGTCGCTCGACGACTACCTCTGCAGATCCTC 1200
cDNA1(MLOC_63436)    GCCGCGGCAGCCACCGCCACCGCCACCGACCCGTTCGGCTTCGACGGCCCGTCGCTCGACGACTACCTCTGCAGATCCTC 1200
cDNA2(MLOC_63436)    ---------------------GCCACCGACCCGTTCGGCTTCGACGGCCCGTCGCTCGACGACTACCTCTGCAGATCCTC 835
CircularRNA          -------------------------------------------------------------------------------- 476
Cir_Forward.Primer   -------------------------------------------------------------------------------- 24
Cir_Reverse.Primer   -------------------------------------------------------------------------------- 28

                                 1.220               1.240               1.260               1.280
Genomic(MLOC_63436)  CCCCGAGATGTTCATGTTCCCAGCCCTCCAAAGCTAGCACCCACGGCCATGGCATGGCAGACGACAGCAGCCAGCACAAG 1280
cDNA1(MLOC_63436)    CCCCGAGATGTTCATGTTCCCAGCCCTCCAAAGCTAGCACCCACGGCCATGGCATGGCAGACGACAGCAGCCAGCACAAG 1280
cDNA2(MLOC_63436)    CCCCGAGATGTTCATGTTCCCAGCCCTCCAAAGCTAGCACCCACGGCCATGGCATGGCAGACGACAGCAGCCAGCACAAG 915
CircularRNA          -------------------------------------------------------------------------------- 476
Cir_Forward.Primer   -------------------------------------------------------------------------------- 24
Cir_Reverse.Primer   -------------------------------------------------------------------------------- 28

                                 1.300               1.320               1.340               1.360
Genomic(MLOC_63436)  ATCGAGCGAGCGAGCGCAGCAAGAAAGATCATCCATCCATCTTTTTTCCCTGATGCTTCTGGTGTTTCAGGTTAATTACG 1360
cDNA1(MLOC_63436)    ATCGAGCGAGCGAGCGCAGCAAGAAAGATCATCCATCCATCTTTTTTCCCTGATGCTTCTGGTGTTTCAGGTTAATTACG 1360
cDNA2(MLOC_63436)    ATCGAGCGAGCGAGCGCAGCAAGAAAGATCATCCATCCATCTTTTTTCCCTGATGCTTCTGGTGTTTCAGGTTAATTACG 995
CircularRNA          -------------------------------------------------------------------------------- 476
Cir_Forward.Primer   -------------------------------------------------------------------------------- 24
Cir_Reverse.Primer   -------------------------------------------------------------------------------- 28

                                 1.380               1.400               1.420               1.440
Genomic(MLOC_63436)  TACTGAAGATTTGGAGGGCCGTGGAGTTTTTACTGCACCTAACTAGAAAAGCTAGCTAGCTAGCAGCTGGGGTTAATTAA 1440
cDNA1(MLOC_63436)    TACTGAAGATTTGGAGGGCCGTGGAGTTTTTACTGCACCTAACTAGAAAAGCTAGCTAGCTAGCAGCTGGGGTTAATTAA 1440
cDNA2(MLOC_63436)    TACTGAAGATTTGGAGGGCCGTGGAGTTTTTACTGCACCTAACTAGAAAAGCTAGCTAGCTAGCAGCTGGGGTTAATTAA 1075
CircularRNA          -------------------------------------------------------------------------------- 476
Cir_Forward.Primer   -------------------------------------------------------------------------------- 24
Cir_Reverse.Primer   -------------------------------------------------------------------------------- 28

                                 1.460               1.480               1.500               1.520
Genomic(MLOC_63436)  TTAGTTACCGCGCCTAATCAATCATCATGTGTAATCTCAACTGGTGTGTGTGTACCAAGTGATGATGGTTCTTAAGCTTA 1520
cDNA1(MLOC_63436)    TTAGTTACCGCGCCTAATCAATCATCATGTGTAATCTCAACTGGTGTGTGTGTACCAAGTGATGATGGTTCTTAAGCTTA 1520
cDNA2(MLOC_63436)    TTAGTTACCGCGCCTAATCAATCATCATGTGTAATCTCAACTGGTGTGTGTGTACCAAGTGATGATGGTTCTTAAGCTTA 1155
CircularRNA          -------------------------------------------------------------------------------- 476
Cir_Forward.Primer   -------------------------------------------------------------------------------- 24
Cir_Reverse.Primer   -------------------------------------------------------------------------------- 28

                                 1.540               1.560               1.580               1.600
Genomic(MLOC_63436)  GCATGCATCACCATCATGTGATCCACTATGTAAGAAGAAGCAGTAAGCTCTCTCTCCCTGTGCTGTTGCTCGATCAGACA 1600
cDNA1(MLOC_63436)    GCATGCATCACCATCATGTGATCCACTATGTAAGAAGAAGCAGTAAGCTCTCTCTCCCTGTGCTGTTGCTCGATCAGACA 1600
cDNA2(MLOC_63436)    GCATGCATCACCATCATGTGATCCACTATGTAAGAAGAAGCAGTAAGCTCTCTCTCCCTGTGCTGTTGCTCGATCAGACA 1235
CircularRNA          -------------------------------------------------------------------------------- 476
Cir_Forward.Primer   -------------------------------------------------------------------------------- 24
Cir_Reverse.Primer   -------------------------------------------------------------------------------- 28

                                 1.620               1.640               1.660               1.680
Genomic(MLOC_63436)  AAGCAAGTGCCATATATAATTACTCTACTTACTTTTACTTACTCATCATCTCATGATTTCTTTTTTACTTCCATGCATGA 1680
cDNA1(MLOC_63436)    AAGCAAGTGCCATATATAATTACTCTACTTACTTTTACTTACTCATCATCTCATGATTTCTTTTTTACTTCCATGCATGA 1680
cDNA2(MLOC_63436)    AAGCAAGTGCCATATATAATTACTCTACTTACTTTTA------------------------------------------- 1272
CircularRNA          -------------------------------------------------------------------------------- 476
Cir_Forward.Primer   -------------------------------------------------------------------------------- 24
Cir_Reverse.Primer   -------------------------------------------------------------------------------- 28

                                 1.700               1.720               1.740               1.760
Genomic(MLOC_63436)  ACATTTTTTTTTTACTTATTTCTGCTACCTACCTTGGAGCAACCTTTTCTTCTCTCTGTCTTTGAATTGTGATGTGCTCT 1760
cDNA1(MLOC_63436)    ACATTTTTTTTTTACTTATTTCTGCTACCTACCTTGGAGCAACCTTTTCTTCTCTCTGTCTTTGAATTGTGATGTGCTCT 1760
cDNA2(MLOC_63436)    -------------------------------------------------------------------------------- 1272
CircularRNA          -------------------------------------------------------------------------------- 476
Cir_Forward.Primer   -------------------------------------------------------------------------------- 24
Cir_Reverse.Primer   -------------------------------------------------------------------------------- 28

                                 1.780               1.800
Genomic(MLOC_63436)  GCTAAAGTGCTGATGCCGCTTACTATCTTGCTCTTCCTGACTTAACTAGCTTC 1813
cDNA1(MLOC_63436)    GCTAAAGTGCTGATGCCGCTTACTATCTTGCTCTTCCTGACTTAACTAGCTTC 1813
cDNA2(MLOC_63436)    ----------------------------------------------------- 1272
CircularRNA          ----------------------------------------------------- 476
Cir_Forward.Primer   ----------------------------------------------------- 24
Cir_Reverse.Primer   ----------------------------------------------------- 28
```

Real-Time PCR for the junction region of BZip11_circular RNA (ID: Ch6:52088926-52089401**)**

By divergent primers ◀▶ on genomic DNA

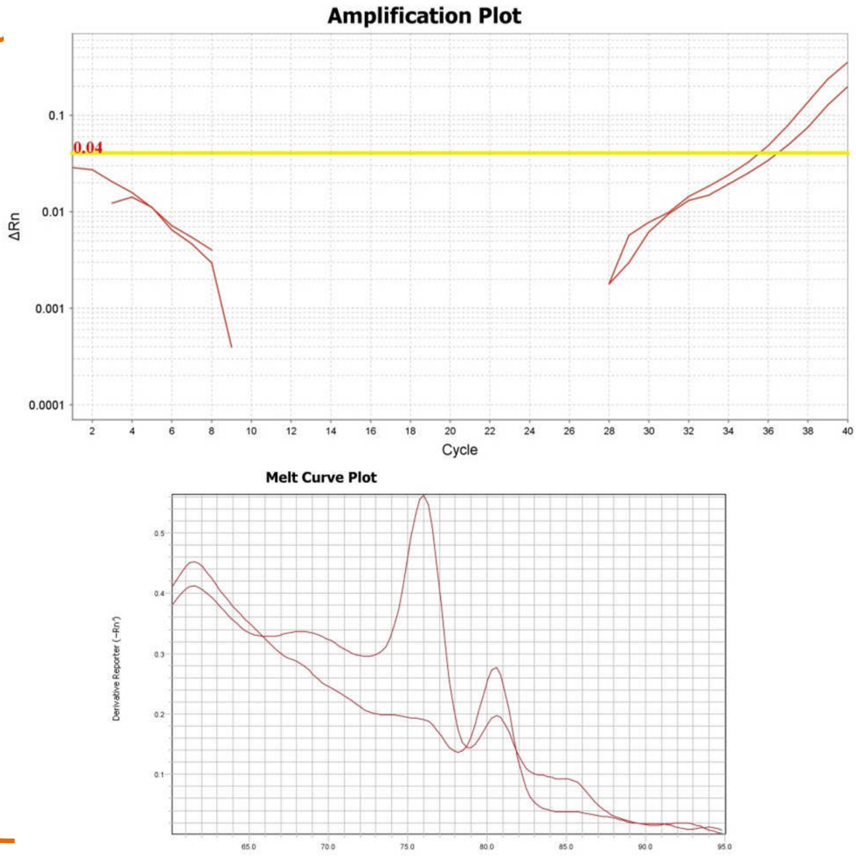

By divergent primers ◀▶ on cDNAs

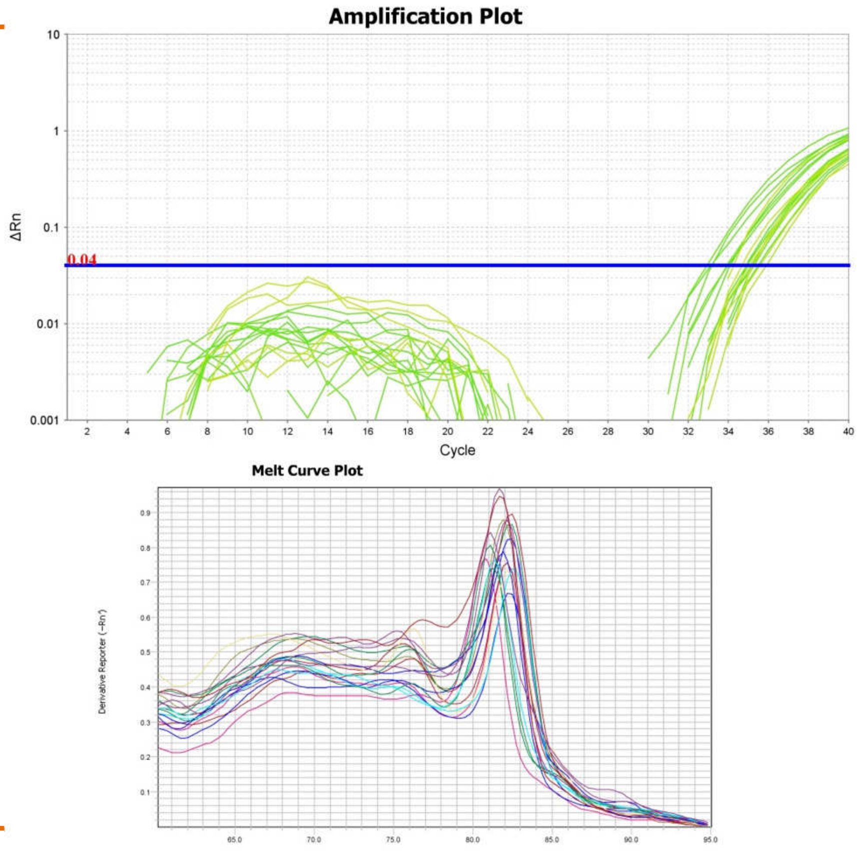

Real-Time PCR for the junction region of BZip11_circular RNA (ID: Ch6:52088926-52089401**)**

By divergent primers ◀▶ & with no template

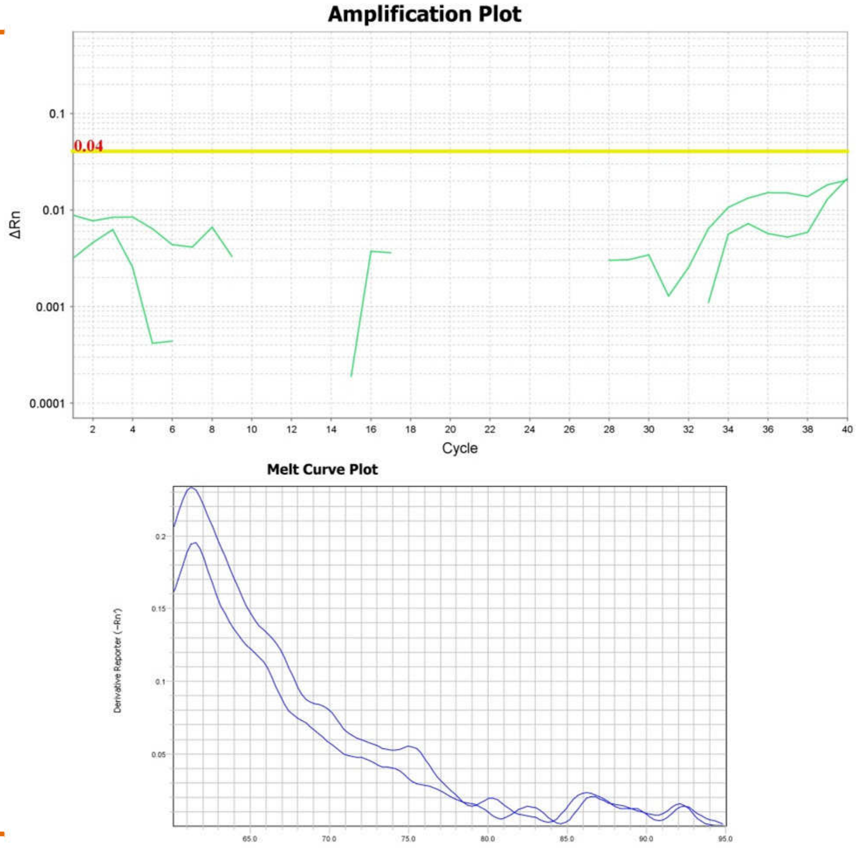

ADP-ribosylation factor 1_circular RNA (ID: Ch1:389129363-389130325)

GTACATCCAGAGCACATGTGCTACCTCTGGGGAGGGGTTGTATGAGGGGCTTGACTGGCTGTC
CAACAACATTGCCAACAAGGNNNNNNNNNNNNNN...NNNNNNNNNNNNNNCTTGAAGCATCTCG
GCAGGGGCTTCTATGGATCTGGATAAGCTGGATATTCGGACTAATGCTGCCTTTACGTATTATAAA
CTATATAGTCTTGACAGATAATGTGGGTGTTTTGGAAGGATAAAGCTCTTTGTGAAAGAGCTAGCT
ATCAGATTGTTTTCGTGGGTGTTTAATTGTCTGGTGCTGTTTGTTTTCCCCATCGTGGTCTTTACTA
TGTCAGCTCTGTACTCGCCACTCCGACAGACATCAGACATTGTAGTATTGACCTGTCTTGGCATAG
TTTGTCA**CTGTGAGGGCTGCCATGTCTCTTCTGAATTGCTATCGCTG**

The nucleotides of junction-region are underlined. The nucleotides of junction-region which are supported by the junction-spaning sequencing reads are shown in red. Introns are not shown if the absence is supported by sequencing reads. In the absence of supporting sequencing reads, the intronic nucletides are shown as N.

Structural relationship between the circular RNA and its parental gene

↓

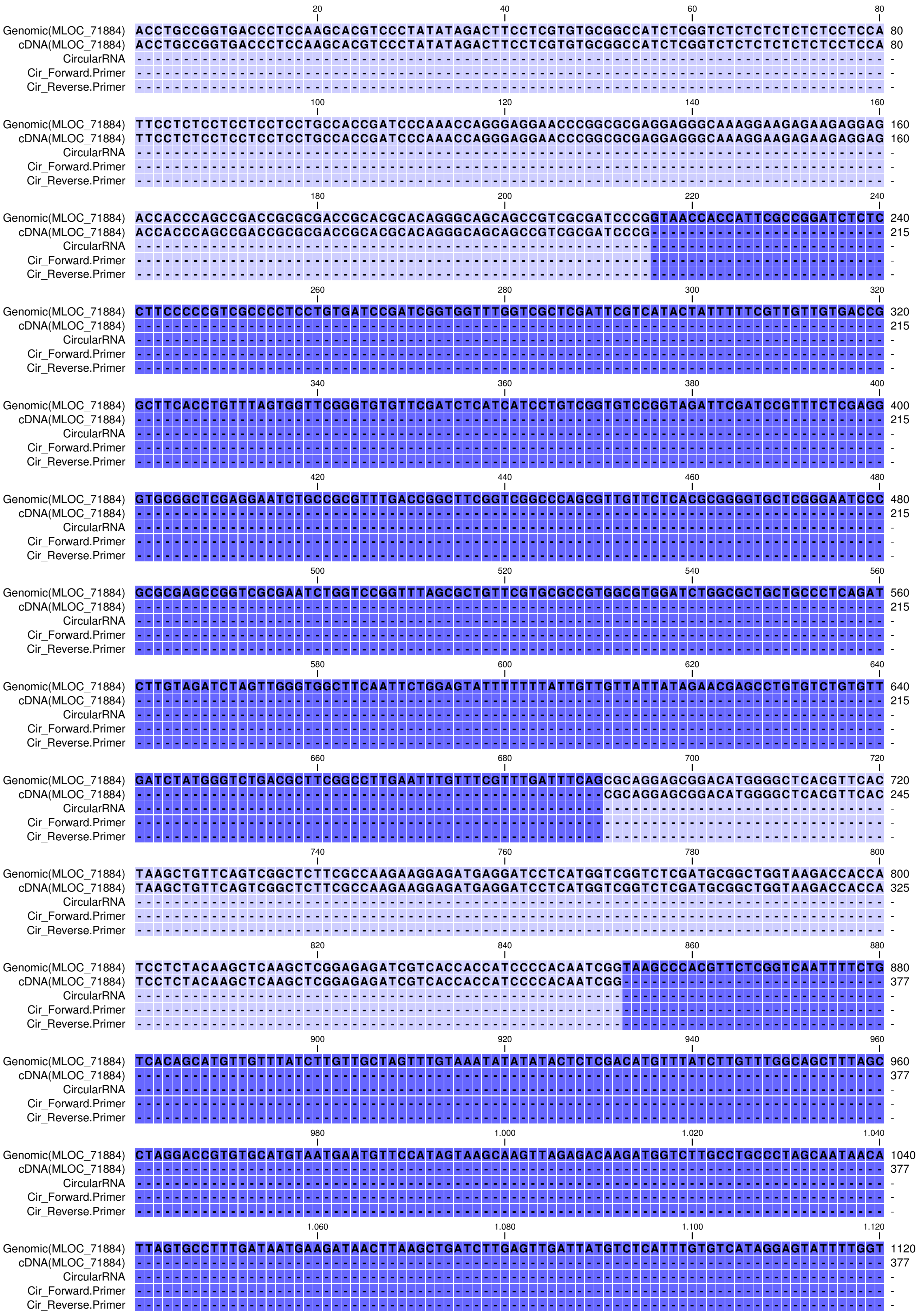

Genomic(MLOC_71884) ACCTGCCGGTGACCCTCCAAGCACGTCCCTATATAGACTTCCTCGTGTGCGGCCATCTCGGTCTCTCTCTCTCTCCTCCA 80
cDNA(MLOC_71884) ACCTGCCGGTGACCCTCCAAGCACGTCCCTATATAGACTTCCTCGTGTGCGGCCATCTCGGTCTCTCTCTCTCTCCTCCA 80
CircularRNA
Cir_Forward.Primer
Cir_Reverse.Primer
Genomic(MLOC_71884) TTCCTCTCCTCCTCCTCCTGCCACCGATCCCAAACCAGGGAGGAACCCGGCGCGAGGAGGGCAAAGGAAGAGAAGAGGAG 160
cDNA(MLOC_71884) TTCCTCTCCTCCTCCTCCTGCCACCGATCCCAAACCAGGGAGGAACCCGGCGCGAGGAGGGCAAAGGAAGAGAAGAGGAG 160
Genomic(MLOC_71884) ACCACCCAGCCGACCGCGCGACCGCACGCACAGGGCAGCAGCCGTCGCGATCCCGGTAACCACCATTCGCCGGATCTCTC 240
cDNA(MLOC_71884) ACCACCCAGCCGACCGCGCGACCGCACGCACAGGGCAGCAGCCGTCGCGATCCCG 215
Genomic(MLOC_71884) CTTCCCCCGTCGCCCCTCCTGTGATCCGATCGGTGGTTTGGTCGCTCGATTCGTCATACTATTTTTCGTTGTTGTGACCG 320
Genomic(MLOC_71884) GCTTCACCTGTTTAGTGGTTCGGGTGTGTTCGATCTCATCATCCTGTCGGTGTCCGGTAGATTCGATCCGTTTCTCGAGG 400
Genomic(MLOC_71884) GTGCGGCTCGAGGAATCTGCCGCGTTTGACCGGCTTCGGTCGGCCCAGCGTTGTTCTCACGCGGGGTGCTCGGGAATCCC 480
Genomic(MLOC_71884) GCGCGAGCCGGTCGCGAATCTGGTCCGGTTTAGCGCTGTTCGTGCGCCGTGGCGTGGATCTGGCGCTGCTGCCCTCAGAT 560
Genomic(MLOC_71884) CTTGTAGATCTAGTTGGGTGGCTTCAATTCTGGAGTATTTTTTTATTGTTGTTATTATAGAACGAGCCTGTGTCTGTGTT 640
Genomic(MLOC_71884) GATCTATGGGTCTGACGCTTCGGCCTTGAATTTGTTTCGTTTGATTTCAGCGCAGGAGCGGACATGGGGCTCACGTTCAC 720
cDNA(MLOC_71884) CGCAGGAGCGGACATGGGGCTCACGTTCAC 245
Genomic(MLOC_71884) TAAGCTGTTCAGTCGGCTCTTCGCCAAGAAGGAGATGAGGATCCTCATGGTCGGTCTCGATGCGGCTGGTAAGACCACCA 800
cDNA(MLOC_71884) TAAGCTGTTCAGTCGGCTCTTCGCCAAGAAGGAGATGAGGATCCTCATGGTCGGTCTCGATGCGGCTGGTAAGACCACCA 325
Genomic(MLOC_71884) TCCTCTACAAGCTCAAGCTCGGAGAGATCGTCACCACCATCCCCACAATCGGTAAGCCCACGTTCTCGGTCAATTTTCTG 880
cDNA(MLOC_71884) TCCTCTACAAGCTCAAGCTCGGAGAGATCGTCACCACCATCCCCACAATCGG 377
Genomic(MLOC_71884) TCACAGCATGTTGTTTATCTTGTTGCTAGTTTGTAAATATATATACTCTCGACATGTTTATCTTGTTTGGCAGCTTTAGC 960
Genomic(MLOC_71884) CTAGGACCGTGTGCATGTAATGAATGTTCCATAGTAAGCAAGTTAGAGACAAGATGGTCTTGCCTGCCCTAGCAATAACA 1040
Genomic(MLOC_71884) TTAGTGCCTTTGATAATGAAGATAACTTAAGCTGATCTTGAGTTGATTATGTCTCATTTGTGTCATAGGAGTATTTTGGT 1120

```
                      1.140                 1.160                 1.180                 1.200
Genomic(MLOC_71884)   AGCTTATGTATGTGCTACTTTTTGTAAATGAACATCATGCCCTATGTTATCTGTAAGTACATTGAAACTTTTACATTTGA 1200
cDNA(MLOC_71884)      -------------------------------------------------------------------------------- 377
CircularRNA           -------------------------------------------------------------------------------- -
Cir_Forward.Primer    -------------------------------------------------------------------------------- -
Cir_Reverse.Primer    -------------------------------------------------------------------------------- -

                      1.220                 1.240                 1.260                 1.280
Genomic(MLOC_71884)   AAAGCAACAATTCGTTGCCCAAATGAAGCCTAATAAAAAGCAAACCATTCTTGTTTAAGATTGCAAGTTGTCTGCTAATT 1280
cDNA(MLOC_71884)      -------------------------------------------------------------------------------- 377
CircularRNA           -------------------------------------------------------------------------------- -
Cir_Forward.Primer    -------------------------------------------------------------------------------- -
Cir_Reverse.Primer    -------------------------------------------------------------------------------- -

                      1.300                 1.320                 1.340                 1.360
Genomic(MLOC_71884)   GTTTTGCATATCACCAAGCTCAGTTTGGTAATTGAGCCTGGGAGTTCAGTCAAGGCAGGATAGAATGTCTGCACCATCAT 1360
cDNA(MLOC_71884)      -------------------------------------------------------------------------------- 377
CircularRNA           -------------------------------------------------------------------------------- -
Cir_Forward.Primer    -------------------------------------------------------------------------------- -
Cir_Reverse.Primer    -------------------------------------------------------------------------------- -

                      1.380                 1.400                 1.420                 1.440
Genomic(MLOC_71884)   TGTGATCGTATGGATCCCGGTGTGTCAGCTGTCTGCTAATTGTTTTGCATATCACCAAGTTAAGTTTGATAATTGAGCCT 1440
cDNA(MLOC_71884)      -------------------------------------------------------------------------------- 377
CircularRNA           -------------------------------------------------------------------------------- -
Cir_Forward.Primer    -------------------------------------------------------------------------------- -
Cir_Reverse.Primer    -------------------------------------------------------------------------------- -

                      1.460                 1.480                 1.500                 1.520
Genomic(MLOC_71884)   GGGAGTTCAGTCAAGGCAGGATAGAGTGTTTGCACCGTCGTTGTGATCATACCGATCTAGACGTGTCAGCACTCTCATAG 1520
cDNA(MLOC_71884)      -------------------------------------------------------------------------------- 377
CircularRNA           -------------------------------------------------------------------------------- -
Cir_Forward.Primer    -------------------------------------------------------------------------------- -
Cir_Reverse.Primer    -------------------------------------------------------------------------------- -

                      1.540                 1.560                 1.580                 1.600
Genomic(MLOC_71884)   TGATCACACAGATTCAGACGTGTCAGCACTCTCATAGTGATCACACAGATTCAGGCGTGTCGGCACTCTCATAGTGATCA 1600
cDNA(MLOC_71884)      -------------------------------------------------------------------------------- 377
CircularRNA           -------------------------------------------------------------------------------- -
Cir_Forward.Primer    -------------------------------------------------------------------------------- -
Cir_Reverse.Primer    -------------------------------------------------------------------------------- -

                      1.620                 1.640                 1.660                 1.680
Genomic(MLOC_71884)   TACACATTCATGTCATTTTCATTGTTACTAATATTGGATGAAAACAATTATGTTTAGTAACCATGAGTCCTAGCAAATGC 1680
cDNA(MLOC_71884)      -------------------------------------------------------------------------------- 377
CircularRNA           -------------------------------------------------------------------------------- -
Cir_Forward.Primer    -------------------------------------------------------------------------------- -
Cir_Reverse.Primer    -------------------------------------------------------------------------------- -

                      1.700                 1.720                 1.740                 1.760
Genomic(MLOC_71884)   CGAATCATTCCAAAGTGGCCAACTCATGAGCAGCATAACCTTCTGTCAGAGTTCTGACTGTGTGTTTGGGCTTGGCATGA 1760
cDNA(MLOC_71884)      -------------------------------------------------------------------------------- 377
CircularRNA           -------------------------------------------------------------------------------- -
Cir_Forward.Primer    -------------------------------------------------------------------------------- -
Cir_Reverse.Primer    -------------------------------------------------------------------------------- -

                      1.780                 1.800                 1.820                 1.840
Genomic(MLOC_71884)   TTGATATTTTTCTATTAACAACAATATGCAGCTCATGGTGTTGATGTGTGTTCAGATGCTTATTTTTTAAATGCTGATTT 1840
cDNA(MLOC_71884)      -------------------------------------------------------------------------------- 377
CircularRNA           -------------------------------------------------------------------------------- -
Cir_Forward.Primer    -------------------------------------------------------------------------------- -
Cir_Reverse.Primer    -------------------------------------------------------------------------------- -

                      1.860                 1.880                 1.900                 1.920
Genomic(MLOC_71884)   TACCGAGGATTTTGACTATGTTCATGTTCTAGATCTTTACTTTGCCACTTTGGCATCACCGTCTATAAACCTTGATGAAA 1920
cDNA(MLOC_71884)      -------------------------------------------------------------------------------- 377
CircularRNA           -------------------------------------------------------------------------------- -
Cir_Forward.Primer    -------------------------------------------------------------------------------- -
Cir_Reverse.Primer    -------------------------------------------------------------------------------- -

                      1.940                 1.960                 1.980                 2.000
Genomic(MLOC_71884)   TCTGAATGTTTTTGAATCTGCAGGGTTTAATGTTGAAACTGTAGAGTACAAGAACATTAGTTTCACCGTTTGGGATGTCG 2000
cDNA(MLOC_71884)      ------------------------GTTTAATGTTGAAACTGTAGAGTACAAGAACATTAGTTTCACCGTTTGGGATGTCG 433
CircularRNA           -------------------------------------------------------------------------------- -
Cir_Forward.Primer    -------------------------------------------------------------------------------- -
Cir_Reverse.Primer    -------------------------------------------------------------------------------- -

                      2.020                 2.040                 2.060                 2.080
Genomic(MLOC_71884)   GGGGTCAGGACAAGGTAAGCAAATTTCCTGAAGGCTAATAAGTTTTGTTTGGCCACATATACCTTTAGTTTAGTCATTTA 2080
cDNA(MLOC_71884)      GGGGTCAGGACAAG------------------------------------------------------------------ 447
CircularRNA           -------------------------------------------------------------------------------- -
Cir_Forward.Primer    -------------------------------------------------------------------------------- -
Cir_Reverse.Primer    -------------------------------------------------------------------------------- -

                      2.100                 2.120                 2.140                 2.160
Genomic(MLOC_71884)   TATATCAGTTGCAAGATTGGAATTATTGAAATTATACTGAGGTGCATGCCTTTTTTGAATCGGCGTTAACAGTTGGCACT 2160
cDNA(MLOC_71884)      -------------------------------------------------------------------------------- 447
CircularRNA           -------------------------------------------------------------------------------- -
Cir_Forward.Primer    -------------------------------------------------------------------------------- -
Cir_Reverse.Primer    -------------------------------------------------------------------------------- -

                      2.180                 2.200                 2.220                 2.240
Genomic(MLOC_71884)   ATAGTTTGGTTAACTTCATCTTCTAATTGGATTTTGGAACATGCCATCGTGCTGTTTTGAATATTTCTATCTTCATGTCA 2240
cDNA(MLOC_71884)      -------------------------------------------------------------------------------- 447
CircularRNA           -------------------------------------------------------------------------------- -
Cir_Forward.Primer    -------------------------------------------------------------------------------- -
Cir_Reverse.Primer    -------------------------------------------------------------------------------- -
```

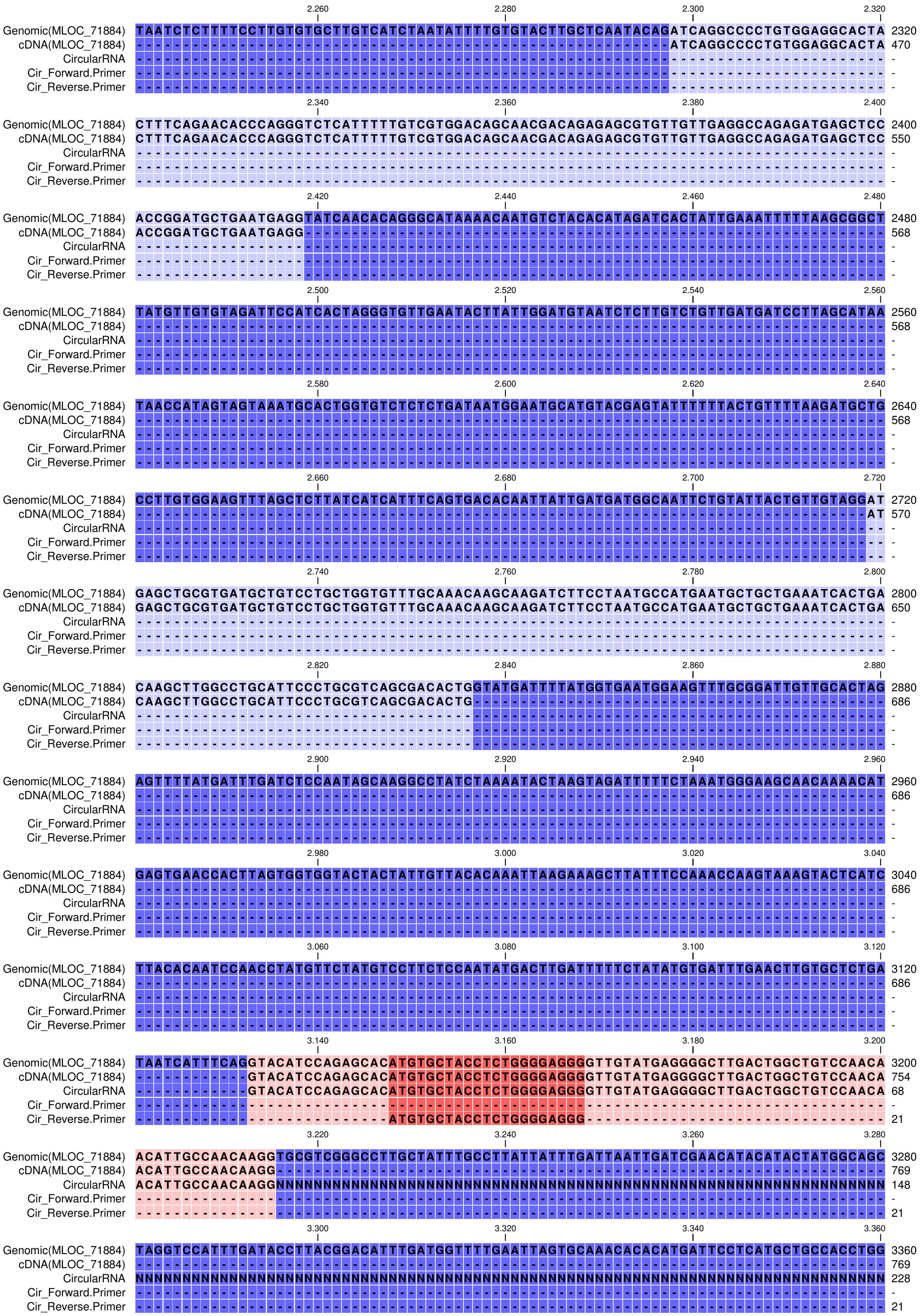
Genomic(MLOC_71884)
cDNA(MLOC_71884)
CircularRNA
Cir_Forward.Primer
Cir_Reverse.Primer

```
                                  3.380                3.400                3.420                3.440
Genomic(MLOC_71884)  TATATAGAAATAAACAAATATAAGACTGTTTGGATCACTACTCTAGTGATCTAAACGATCACTAGAGTAGTGATCCAAAC  3440
cDNA(MLOC_71884)     --------------------------------------------------------------------------------  769
CircularRNA          NNNNNNNNNNNNNNNNNNNNNNNNNNNNNNNNNNNNNNNNNNNNNNNNNNNNNNNNNNNNNNNNNNNNNNNNNNNNNNNN  308
Cir_Forward.Primer   --------------------------------------------------------------------------------  -
Cir_Reverse.Primer   --------------------------------------------------------------------------------  21

                                  3.460                3.480                3.500                3.520
Genomic(MLOC_71884)  AGCCTTATATTTGTTTACGGAGGGAGTACTTGGCAGCTTTAGTACTCCCTCCGTCTGAAAATACTTGTCCTAGAAATAGA  3520
cDNA(MLOC_71884)     --------------------------------------------------------------------------------  769
CircularRNA          NNNNNNNNNNNNNNNNNNNNNNNNNNNNNNNNNNNNNNNNNNNNNNNNNNNNNNNNNNNNNNNNNNNNNNNNNNNNNNNN  388
Cir_Forward.Primer   --------------------------------------------------------------------------------  -
Cir_Reverse.Primer   --------------------------------------------------------------------------------  21

                                  3.540                3.560                3.580                3.600
Genomic(MLOC_71884)  TGTATCTAGACTTATTTAAGTTATAGATACATCCATTTTATACATTTCTAGGACAAGTATTTCCGGACGGAGTAATATAT  3600
cDNA(MLOC_71884)     --------------------------------------------------------------------------------  769
CircularRNA          NNNNNNNNNNNNNNNNNNNNNNNNNNNNNNNNNNNNNNNNNNNNNNNNNNNNNNNNNNNNNNNNNNNNNNNNNNNNNNNN  468
Cir_Forward.Primer   --------------------------------------------------------------------------------  -
Cir_Reverse.Primer   --------------------------------------------------------------------------------  21

                                  3.620                3.640                3.660                3.680
Genomic(MLOC_71884)  GGAAATGCTGCGTCTGCATTTTCTCTTCTATTATTGCTGTTTAGTTTTACTCAGATTTGGGGATAATGAATCAACTGAGC  3680
cDNA(MLOC_71884)     --------------------------------------------------------------------------------  769
CircularRNA          NNNNNNNNNNNNNNNNNNNNNNNNNNNNNNNNNNNNNNNNNNNNNNNNNNNNNNNNNNNNNNNNNNNNNNNNNNNNNNNN  548
Cir_Forward.Primer   --------------------------------------------------------------------------------  -
Cir_Reverse.Primer   --------------------------------------------------------------------------------  21

                                  3.700                3.720                3.740                3.760
Genomic(MLOC_71884)  GCTCTTCCGAGATTTGTGTCTTTCCTCTCTGGGGTCTTACTTGATTGATTTCTGCAACTAACTACCTGTCTGTAATTTGC  3760
cDNA(MLOC_71884)     --------------------------------------------------------------------------------  769
CircularRNA          NNNNNNNNNNNNNNNNNNNNNNNNNNNNNNNNNNNNNNNNNNNNNNNNNNNNNNNNNNNNNNNNNNNNNNNNNNNNNNNN  628
Cir_Forward.Primer   --------------------------------------------------------------------------------  -
Cir_Reverse.Primer   --------------------------------------------------------------------------------  21

                                  3.780                3.800                3.820                3.840
Genomic(MLOC_71884)  TGATATAGGCTTGAAGCATCTCGGCAGGGGCTTCTATGGATCTGGATAAGCTGGATATTCGGACTAATGCTGCCTTTACG  3840
cDNA(MLOC_71884)     ---------CTTGAAGCATCTCGGCAGGGGCTTCTATGGATCTGGATAAGCTGGATATTCGGACTAATGCTGCCTTTACG  840
CircularRNA          NNNNNNNNNCTTGAAGCATCTCGGCAGGGGCTTCTATGGATCTGGATAAGCTGGATATTCGGACTAATGCTGCCTTTACG  708
Cir_Forward.Primer   --------------------------------------------------------------------------------  -
Cir_Reverse.Primer   --------------------------------------------------------------------------------  21

                                  3.860                3.880                3.900                3.920
Genomic(MLOC_71884)  TATTATAAACTATATAGTCTTGACAGATAATGTGGGTGTTTTGGAAGGATAAAGCTCTTTGTGAAAGAGCTAGCTATCAG  3920
cDNA(MLOC_71884)     TATTATAAACTATATAGTCTTGACAGATAATGTGGGTGTTTTGGAAGGATAAAGCTCTTTGTGAAAGAGCTAGCTATCAG  920
CircularRNA          TATTATAAACTATATAGTCTTGACAGATAATGTGGGTGTTTTGGAAGGATAAAGCTCTTTGTGAAAGAGCTAGCTATCAG  788
Cir_Forward.Primer   --------------------------------------------------------------------------------  -
Cir_Reverse.Primer   --------------------------------------------------------------------------------  21

                                  3.940                3.960                3.980                4.000
Genomic(MLOC_71884)  ATTGTTTTCGTGGGTGTTTAATTGTCTGGTGCTGTTTGTTTTCCCCATCGTGGTCTTTACTATGTCAGCTCTGTACTCGC  4000
cDNA(MLOC_71884)     ATTGTTTTCGTGGGTGTTTAATTGTCTGGTGCTGTTTGTTTTCCCCATCGTGGTCTTTACTATGTCAGCTCTGTACTCGC  1000
CircularRNA          ATTGTTTTCGTGGGTGTTTAATTGTCTGGTGCTGTTTGTTTTCCCCATCGTGGTCTTTACTATGTCAGCTCTGTACTCGC  868
Cir_Forward.Primer   --------------------------------------------------------------------------------  -
Cir_Reverse.Primer   --------------------------------------------------------------------------------  21

                                  4.020                4.040                4.060                4.080
Genomic(MLOC_71884)  CACTCCGACAGACATCAGACATTGTAGTATTGACCTGTCTTGGCATAGTTTGTCACTGTGAGGGCTGCCATGTCTCTTCT  4080
cDNA(MLOC_71884)     CACTCCGACAGACATCAGACATTGTA------------------------------------------------------  1026
CircularRNA          CACTCCGACAGACATCAGACATTGTAGTATTGACCTGTCTTGGCATAGTTTGTCACTGTGAGGGCTGCCATGTCTCTTCT  948
Cir_Forward.Primer   ----------------------------------CTGTCTTGGCATAGTTTGTCACTGT---------------------  25
Cir_Reverse.Primer   --------------------------------------------------------------------------------  21

                                  4.100                4.120
Genomic(MLOC_71884)  GAATTGCTATCGCTGGTGTTGATTTCATGTGCAACAGACATTTTCG  4126
cDNA(MLOC_71884)     ----------------------------------------------  1026
CircularRNA          GAATTGCTATCGCTG-------------------------------  963
Cir_Forward.Primer   ----------------------------------------------  25
Cir_Reverse.Primer   ----------------------------------------------  21
```

Real-Time PCR for the junction region of ADP-ribosylation factor 1_circular RNA (ID: Ch1:389129363-389130325)

By divergent primers ◀▶ on genomic DNA

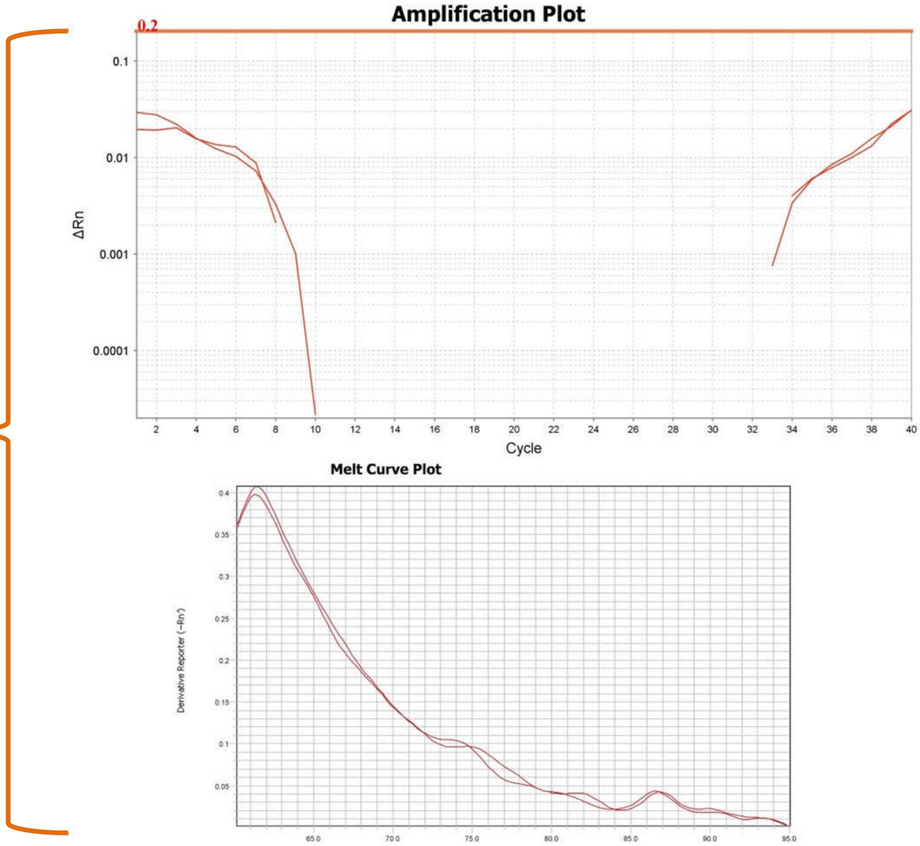

By divergent primers ◀▶ on cDNAs

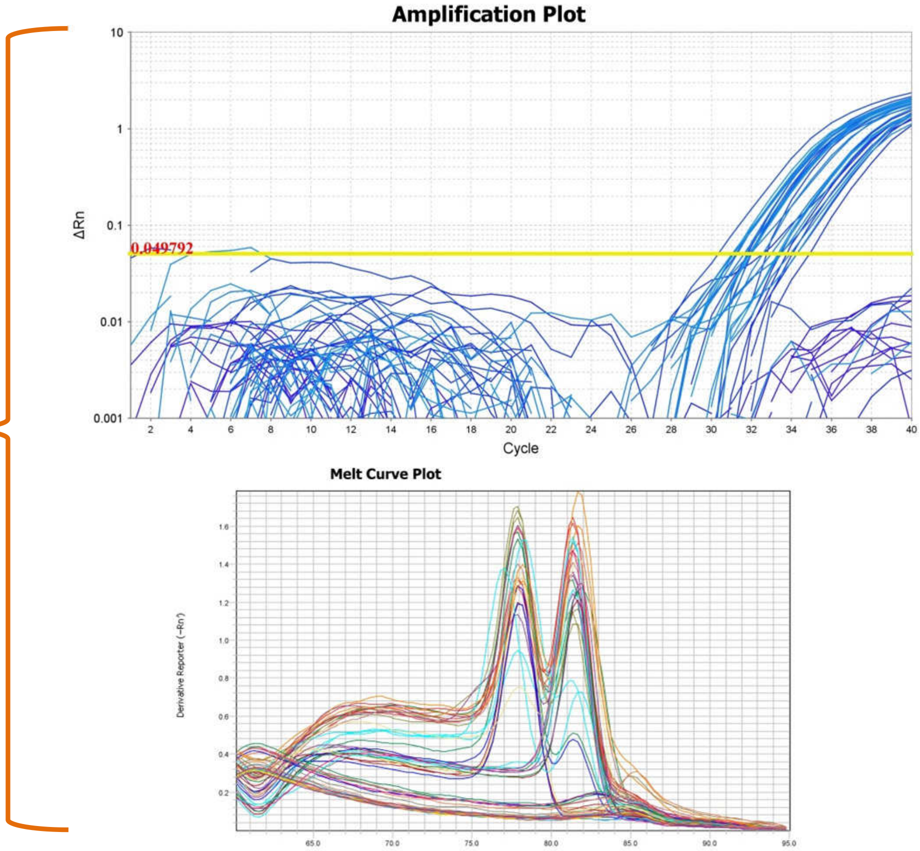

Real-Time PCR for the junction region of ADP-ribosylation factor 1_circular RNA (ID: Ch1:389129363-389130325**)**

By divergent primers ◀▶ & with no template

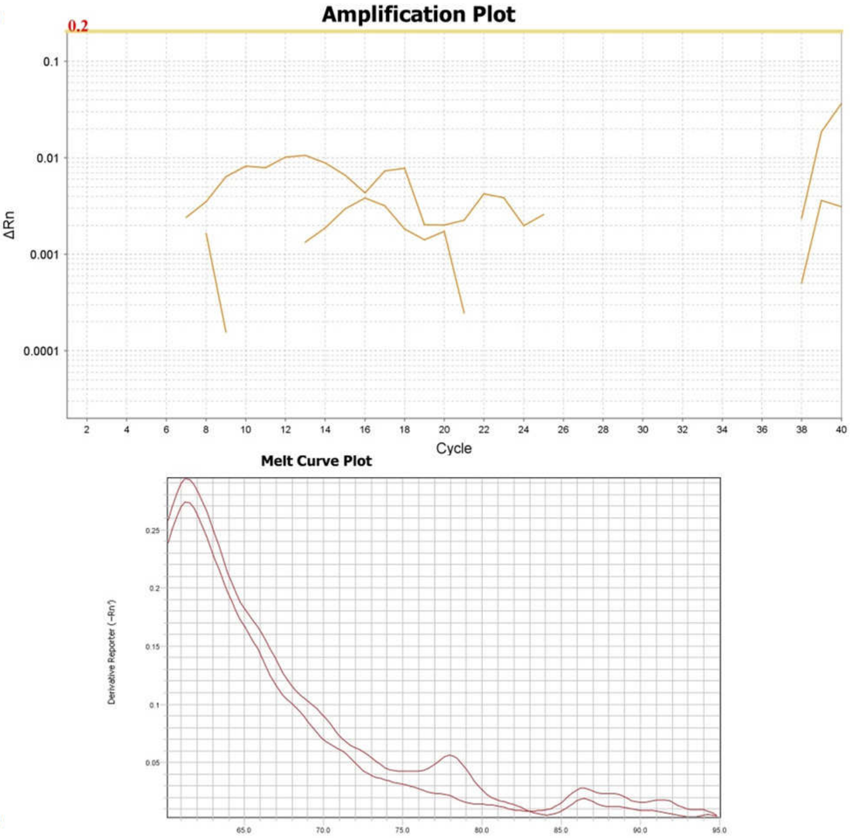

Sec23/Sec24 transport protein_circular RNA (ID: Ch2:482080734-482081397**)**

GTTATTTTGGCTTCCTTAAGTGATGTGGTAGAAGCAAGGCTATGGCTCCATGATTGGTTGGCAATT
TTCATTGCCCAGTACAACAAGGCATACAAGAGCGTCAGATCTGCTGATTCTGGAGTTTCTGATATT
GACGTTAATTTTTCGAACTGCTCACAGTTGCAGCCTCTGGCACGGCTTGTGTTTGCATTTCTAGTA
AGCCCCTTGCTTCAAGTTCAGGATGAACATATTCATCCAGATTACCAGACATATTTGCAGTGCCTC
TTCAGNNNNNNNNNNNNNN...NNNNNNNNNNNNNNTGTACTGGAACCGGCTTCTCTTCGGCAAGCT
ATATGTCCTACACTGAGTTCGTACTCCTCTCCTGATATAGAAGCAGAAGTGCATCAGTCGCTAAGT
CGCAGTGTATTCACCAGTGAGAGACCAATATTCCTTCTTGACGCTTACACTGACCTTTTGGTGTAT
TACTTGCCTACAGCCAGCCCTTCAATTCCCTTTCCTCCTCCACGTGACT

The nucleotides of junction-region are underlined. The nucleotides of junction-region which are supported by the junction-spaning sequencing reads are shown in red. Introns are not shown if the absence is supported by sequencing reads. In the absence of supporting sequencing reads, the intronic nucletides are shown as N.

Structural relationship between the circular RNA and its parental gene

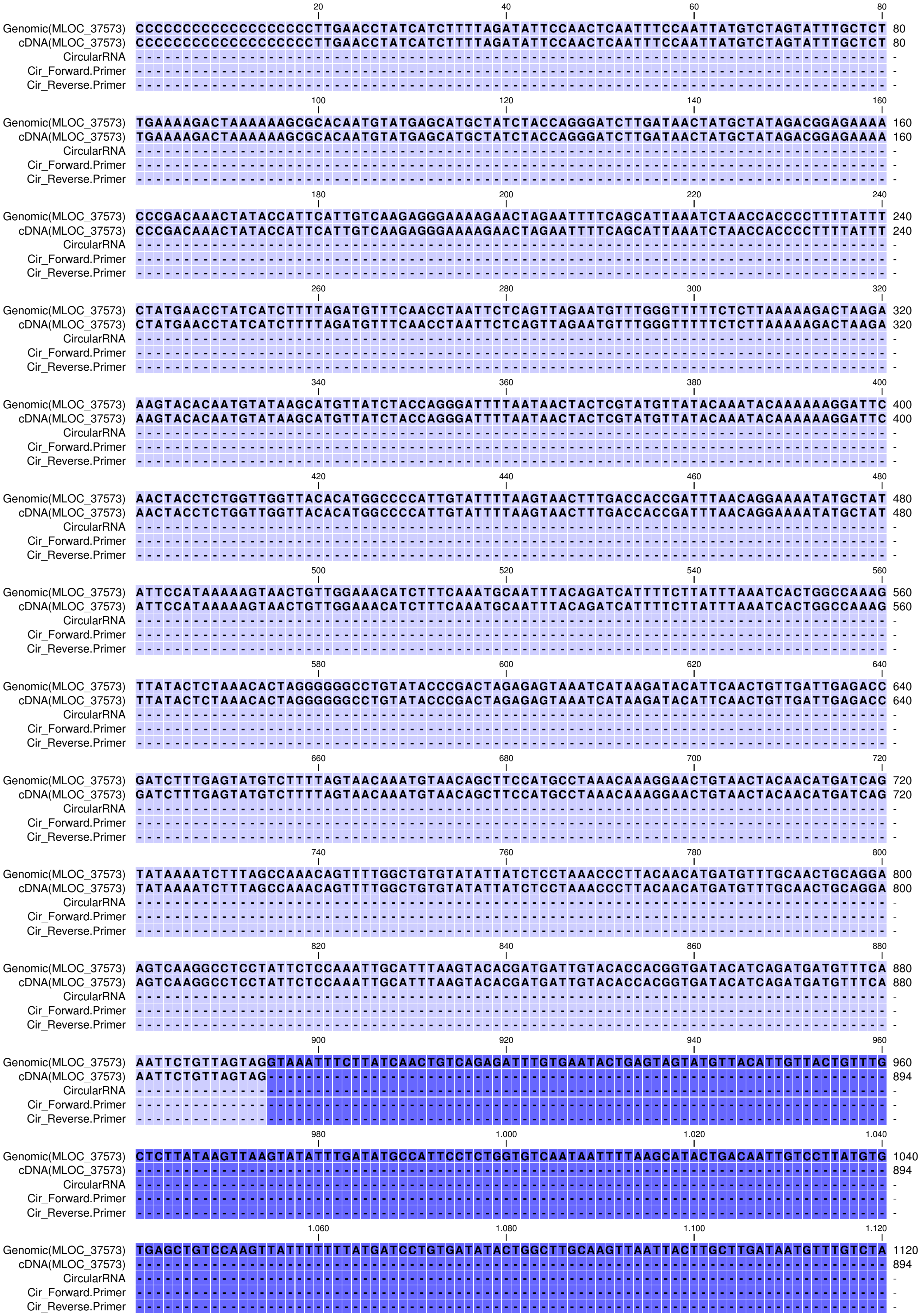
20 40 60 80
Genomic(MLOC_37573) CCCCCCCCCCCCCCCCCCCCTTGAACCTATCATCTTTTAGATATTCCAACTCAATTTCCAATTATGTCTAGTATTTGCTCT 80
cDNA(MLOC_37573) CCCCCCCCCCCCCCCCCCCCTTGAACCTATCATCTTTTAGATATTCCAACTCAATTTCCAATTATGTCTAGTATTTGCTCT 80
CircularRNA -
Cir_Forward.Primer -
Cir_Reverse.Primer -
100 120 140 160
Genomic(MLOC_37573) TGAAAAGACTAAAAAAGCGCACAATGTATGAGCATGCTATCTACCAGGGATCTTGATAACTATGCTATAGACGGAGAAAA 160
cDNA(MLOC_37573) TGAAAAGACTAAAAAAGCGCACAATGTATGAGCATGCTATCTACCAGGGATCTTGATAACTATGCTATAGACGGAGAAAA 160
CircularRNA -
Cir_Forward.Primer -
Cir_Reverse.Primer -
180 200 220 240
Genomic(MLOC_37573) CCCGACAAACTATACCATTCATTGTCAAGAGGGAAAAGAACTAGAATTTTCAGCATTAAATCTAACCACCCCTTTTATTT 240
cDNA(MLOC_37573) CCCGACAAACTATACCATTCATTGTCAAGAGGGAAAAGAACTAGAATTTTCAGCATTAAATCTAACCACCCCTTTTATTT 240
CircularRNA -
Cir_Forward.Primer -
Cir_Reverse.Primer -
260 280 300 320
Genomic(MLOC_37573) CTATGAACCTATCATCTTTTAGATGTTTCAACCTAATTCTCAGTTAGAATGTTTGGGTTTTTCTCTTAAAAAGACTAAGA 320
cDNA(MLOC_37573) CTATGAACCTATCATCTTTTAGATGTTTCAACCTAATTCTCAGTTAGAATGTTTGGGTTTTTCTCTTAAAAAGACTAAGA 320
CircularRNA -
Cir_Forward.Primer -
Cir_Reverse.Primer -
340 360 380 400
Genomic(MLOC_37573) AAGTACACAATGTATAAGCATGTTATCTACCAGGGATTTTAATAACTACTCGTATGTTATACAAATACAAAAAAGGATTC 400
cDNA(MLOC_37573) AAGTACACAATGTATAAGCATGTTATCTACCAGGGATTTTAATAACTACTCGTATGTTATACAAATACAAAAAAGGATTC 400
CircularRNA -
Cir_Forward.Primer -
Cir_Reverse.Primer -
420 440 460 480
Genomic(MLOC_37573) AACTACCTCTGGTTGGTTACACATGGCCCCATTGTATTTTAAGTAACTTTGACCACCGATTTAACAGGAAAATATGCTAT 480
cDNA(MLOC_37573) AACTACCTCTGGTTGGTTACACATGGCCCCATTGTATTTTAAGTAACTTTGACCACCGATTTAACAGGAAAATATGCTAT 480
CircularRNA -
Cir_Forward.Primer -
Cir_Reverse.Primer -
500 520 540 560
Genomic(MLOC_37573) ATTCCATAAAAAGTAACTGTTGGAAACATCTTTCAAATGCAATTTACAGATCATTTTCTTATTTAAATCACTGGCCAAAG 560
cDNA(MLOC_37573) ATTCCATAAAAAGTAACTGTTGGAAACATCTTTCAAATGCAATTTACAGATCATTTTCTTATTTAAATCACTGGCCAAAG 560
CircularRNA -
Cir_Forward.Primer -
Cir_Reverse.Primer -
580 600 620 640
Genomic(MLOC_37573) TTATACTCTAAACACTAGGGGGGCCTGTATACCCGACTAGAGAGTAAATCATAAGATACATTCAACTGTTGATTGAGACC 640
cDNA(MLOC_37573) TTATACTCTAAACACTAGGGGGGCCTGTATACCCGACTAGAGAGTAAATCATAAGATACATTCAACTGTTGATTGAGACC 640
CircularRNA -
Cir_Forward.Primer -
Cir_Reverse.Primer -
660 680 700 720
Genomic(MLOC_37573) GATCTTTGAGTATGTCTTTTAGTAACAAATGTAACAGCTTCCATGCCTAAACAAAGGAACTGTAACTACAACATGATCAG 720
cDNA(MLOC_37573) GATCTTTGAGTATGTCTTTTAGTAACAAATGTAACAGCTTCCATGCCTAAACAAAGGAACTGTAACTACAACATGATCAG 720
CircularRNA -
Cir_Forward.Primer -
Cir_Reverse.Primer -
740 760 780 800
Genomic(MLOC_37573) TATAAAATCTTTAGCCAAACAGTTTTGGCTGTGTATATTATCTCCTAAACCCTTACAACATGATGTTTGCAACTGCAGGA 800
cDNA(MLOC_37573) TATAAAATCTTTAGCCAAACAGTTTTGGCTGTGTATATTATCTCCTAAACCCTTACAACATGATGTTTGCAACTGCAGGA 800
CircularRNA -
Cir_Forward.Primer -
Cir_Reverse.Primer -
820 840 860 880
Genomic(MLOC_37573) AGTCAAGGCCTCCTATTCTCCAAATTGCATTTAAGTACACGATGATTGTACACCACGGTGATACATCAGATGATGTTTCA 880
cDNA(MLOC_37573) AGTCAAGGCCTCCTATTCTCCAAATTGCATTTAAGTACACGATGATTGTACACCACGGTGATACATCAGATGATGTTTCA 880
CircularRNA -
Cir_Forward.Primer -
Cir_Reverse.Primer -
900 920 940 960
Genomic(MLOC_37573) AATTCTGTTAGTAGGTAAATTTCTTATCAACTGTCAGAGATTTGTGAATACTGAGTAGTATGTTACATTGTTACTGTTTG 960
cDNA(MLOC_37573) AATTCTGTTAGTAG 894
CircularRNA -
Cir_Forward.Primer -
Cir_Reverse.Primer -
980 1.000 1.020 1.040
Genomic(MLOC_37573) CTCTTATAAGTTAAGTATATTTGATATGCCATTCCTCTGGTGTCAATAATTTTAAGCATACTGACAATTGTCCTTATGTG 1040
cDNA(MLOC_37573) 894
CircularRNA -
Cir_Forward.Primer -
Cir_Reverse.Primer -
1.060 1.080 1.100 1.120
Genomic(MLOC_37573) TGAGCTGTCCAAGTTATTTTTTTATGATCCTGTGATATACTGGCTTGCAAGTTAATTACTTGCTTGATAATGTTTGTCTA 1120
cDNA(MLOC_37573) 894
CircularRNA -
Cir_Forward.Primer -
Cir_Reverse.Primer -

```
                     1.140               1.160               1.180               1.200
Genomic(MLOC_37573)  ATTGTCAATTTGATATCGTCAACTTTGCAGATGTTCCATGCTAGCAGCAGTTTTATACACATTTTTATCTATATTTTGTA 1200
cDNA(MLOC_37573)     -------------------------------------------------------------------------------- 894
CircularRNA          -------------------------------------------------------------------------------- -
Cir_Forward.Primer   -------------------------------------------------------------------------------- -
Cir_Reverse.Primer   -------------------------------------------------------------------------------- -

                     1.220               1.240               1.260               1.280
Genomic(MLOC_37573)  ACTTATTAAAAATGCACCTTGTCCAGATCTAAGTTCTCATTGCAAAGGCGGCTAAGGGTGCGGACTATCCAATACAACAC 1280
cDNA(MLOC_37573)     --------------------------ATCTAAGTTCTCATTGCAAAGGCGGCTAAGGGTGCGGACTATCCAATACAACAC 948
CircularRNA          -------------------------------------------------------------------------------- -
Cir_Forward.Primer   -------------------------------------------------------------------------------- -
Cir_Reverse.Primer   -------------------------------------------------------------------------------- -

                     1.300               1.320               1.340               1.360
Genomic(MLOC_37573)  AACTGCTAATATCTGGGACCTGTATGACTTTGTTGACCCAGATGTGGTATTGACGATACTTGTACACCAGGTACAACCTC 1360
cDNA(MLOC_37573)     AACTGCTAATATCTGGGACCTGTATGACTTTGTTGACCCAGATGTGGTATTGACGATACTTGTACA-------------- 1014
CircularRNA          -------------------------------------------------------------------------------- -
Cir_Forward.Primer   -------------------------------------------------------------------------------- -
Cir_Reverse.Primer   -------------------------------------------------------------------------------- -

                     1.380               1.400               1.420               1.440
Genomic(MLOC_37573)  AGTTTTACATACATTTGTGTTATACTCCCTCCGTCCGAAAATAAGTGTCTTAAACTTAATACAACTTTGTACTAGCTCTA 1440
cDNA(MLOC_37573)     -------------------------------------------------------------------------------- 1014
CircularRNA          -------------------------------------------------------------------------------- -
Cir_Forward.Primer   -------------------------------------------------------------------------------- -
Cir_Reverse.Primer   -------------------------------------------------------------------------------- -

                     1.460               1.480               1.500               1.520
Genomic(MLOC_37573)  TTACAAAGTTGAGACAGTTATTTTGGGACGGAGGGAGTACAATTTTAATGGATAAACTGTTTGTCGTAAGCACAAAGGCT 1520
cDNA(MLOC_37573)     -------------------------------------------------------------------------------- 1014
CircularRNA          -------------------------------------------------------------------------------- -
Cir_Forward.Primer   -------------------------------------------------------------------------------- -
Cir_Reverse.Primer   -------------------------------------------------------------------------------- -

                     1.540               1.560               1.580               1.600
Genomic(MLOC_37573)  GTTAGCTGTACGACTATAGCATCTTACTACATCAATTAATTGCCACTACAAGAAACTTATAGCAATCAAATGTTGCCAAA 1600
cDNA(MLOC_37573)     -------------------------------------------------------------------------------- 1014
CircularRNA          -------------------------------------------------------------------------------- -
Cir_Forward.Primer   -------------------------------------------------------------------------------- -
Cir_Reverse.Primer   -------------------------------------------------------------------------------- -

                     1.620               1.640               1.660               1.680
Genomic(MLOC_37573)  GCCTCTGTAGGACTATAACATCTTACTACATCAATTAACTGCAACTCTACAAGAAAATTAGGGTAGGAACTTTGTCTGGG 1680
cDNA(MLOC_37573)     -------------------------------------------------------------------------------- 1014
CircularRNA          -------------------------------------------------------------------------------- -
Cir_Forward.Primer   -------------------------------------------------------------------------------- -
Cir_Reverse.Primer   -------------------------------------------------------------------------------- -

                     1.700               1.720               1.740               1.760
Genomic(MLOC_37573)  ATGCAGCTGGTGACAAGCTTAGCGTTTCTGTATTTTACTTAAACATTCATTGAAGAAATTTATTTGAATATTCATCTAAT 1760
cDNA(MLOC_37573)     -------------------------------------------------------------------------------- 1014
CircularRNA          -------------------------------------------------------------------------------- -
Cir_Forward.Primer   -------------------------------------------------------------------------------- -
Cir_Reverse.Primer   -------------------------------------------------------------------------------- -

                     1.780               1.800               1.820               1.840
Genomic(MLOC_37573)  GGTTTTATTTGAATGTTCGTCAACAGTTTTCTATCTTAACATCATTACAAAGATTGATATGATTCCACCCTTCTAGGCTT 1840
cDNA(MLOC_37573)     -------------------------------------------------------------------------------- 1014
CircularRNA          -------------------------------------------------------------------------------- -
Cir_Forward.Primer   -------------------------------------------------------------------------------- -
Cir_Reverse.Primer   -------------------------------------------------------------------------------- -

                     1.860               1.880               1.900               1.920
Genomic(MLOC_37573)  AGGAACTATTAGGTCCCTATTTTGGCAGGGCTGGTTTATCTTGTTCCCTGCAATCTGAAGCTAGGTTTACTCTGGCTGTC 1920
cDNA(MLOC_37573)     -------------------------------------------------------------------------------- 1014
CircularRNA          -------------------------------------------------------------------------------- -
Cir_Forward.Primer   -------------------------------------------------------------------------------- -
Cir_Reverse.Primer   -------------------------------------------------------------------------------- -

                     1.940               1.960               1.980               2.000
Genomic(MLOC_37573)  TGGCACTAGTTTCCGCCTTTCTGATTTATTGAAGCTGATCCAACCCAACAGGTTTCGAATTCTAGGTTTCCAAAATGCTC 2000
cDNA(MLOC_37573)     -------------------------------------------------------------------------------- 1014
CircularRNA          -------------------------------------------------------------------------------- -
Cir_Forward.Primer   -------------------------------------------------------------------------------- -
Cir_Reverse.Primer   -------------------------------------------------------------------------------- -

                     2.020               2.040               2.060               2.080
Genomic(MLOC_37573)  TCAGGACTCTGTTATATGGAAGAAGAGAAGCTAAATTTGACTGCAGTCATTGTATGTTAATCTATTAATTCACCATAAGG 2080
cDNA(MLOC_37573)     -------------------------------------------------------------------------------- 1014
CircularRNA          -------------------------------------------------------------------------------- -
Cir_Forward.Primer   -------------------------------------------------------------------------------- -
Cir_Reverse.Primer   -------------------------------------------------------------------------------- -

                     2.100               2.120               2.140               2.160
Genomic(MLOC_37573)  AATCCTAGTCTAGCTTAAATCCTTATCTGAATCAGCTTCTTTACTGAATACGGTTCTGCAGGGAGGTAATATATATTGTA 2160
cDNA(MLOC_37573)     -------------------------------------------------------------------------------- 1014
CircularRNA          -------------------------------------------------------------------------------- -
Cir_Forward.Primer   -------------------------------------------------------------------------------- -
Cir_Reverse.Primer   -------------------------------------------------------------------------------- -

                     2.180               2.200               2.220               2.240
Genomic(MLOC_37573)  GTTTTGGCATTCTGTGGTATTTTTGTTATAAATGCTAAGCTTGGTGACCGTAAAAACAGTAGAGAATGTTCATGATCTTC 2240
cDNA(MLOC_37573)     -------------------------------------------------------------------------------- 1014
CircularRNA          -------------------------------------------------------------------------------- -
Cir_Forward.Primer   -------------------------------------------------------------------------------- -
Cir_Reverse.Primer   -------------------------------------------------------------------------------- -
```

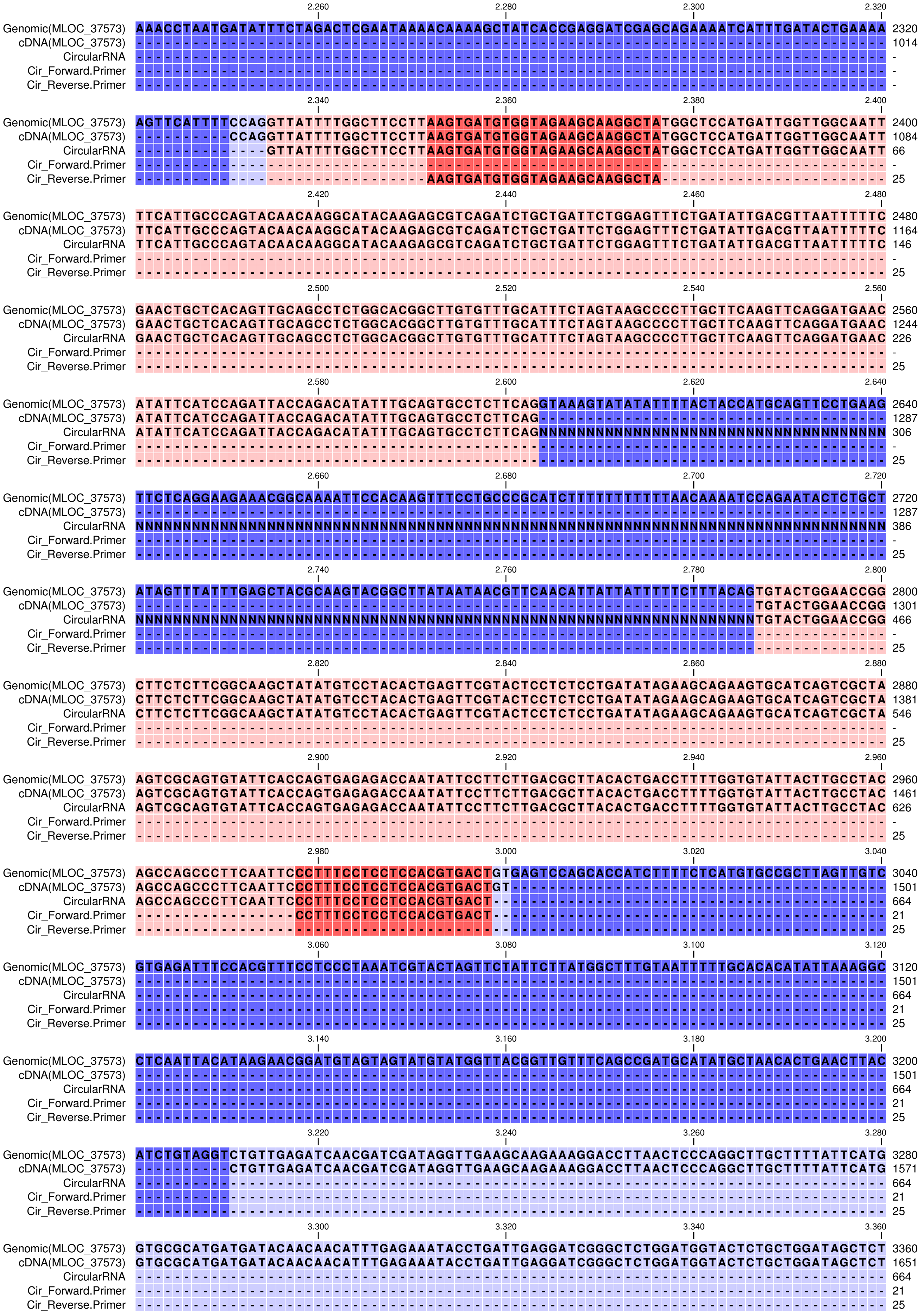
Genomic(MLOC_37573)
cDNA(MLOC_37573)
CircularRNA
Cir_Forward.Primer
Cir_Reverse.Primer
AAACCTAATGATATTTCTAGACTCGAATAAAACAAAAGCTATCACCGAGGATCGAGCAGAAAATCATTTGATACTGAAAA 2320
AGTTCATTTTCCAGGTTATTTTGGCTTCCTTAAGTGATGTGGTAGAAGCAAGGCTATGGCTCCATGATTGGTTGGCAATT 2400
AAGTGATGTGGTAGAAGCAAGGCTA 25
TTCATTGCCCAGTACAACAAGGCATACAAGAGCGTCAGATCTGCTGATTCTGGAGTTTCTGATATTGACGTTAATTTTTC 2480
GAACTGCTCACAGTTGCAGCCTCTGGCACGGCTTGTGTTTGCATTTCTAGTAAGCCCCTTGCTTCAAGTTCAGGATGAAC 2560
ATATTCATCCAGATTACCAGACATATTTGCAGTGCCTCTTCAGGTAAAGTATATATTTTACTACCATGCAGTTCCTGAAG 2640
TTCTCAGGAAGAAACGGCAAAATTCCACAAGTTTCCTGCCCGCATCTTTTTTTTTTTAACAAAATCCAGAATACTCTGCT 2720
ATAGTTTATTTGAGCTACGCAAGTACGGCTTATAATAACGTTCAACATTATTATTTTTCTTTACAGTGTACTGGAACCGG 2800
CTTCTCTTCGGCAAGCTATATGTCCTACACTGAGTTCGTACTCCTCTCCTGATATAGAAGCAGAAGTGCATCAGTCGCTA 2880
AGTCGCAGTGTATTCACCAGTGAGAGACCAATATTCCTTCTTGACGCTTACACTGACCTTTTGGTGTATTACTTGCCTAC 2960
AGCCAGCCCTTCAATTCCCTTTCCTCCTCCACGTGACTGTGAGTCCAGCACCATCTTTTCTCATGTGCCGCTTAGTTGTC 3040
CCTTTCCTCCTCCACGTGACT 21
GTGAGATTTCCACGTTTCCTCCCTAAATCGTACTAGTTCTATTCTTATGGCTTTGTAATTTTTGCACACATATTAAAGGC 3120
CTCAATTACATAAGAACGGATGTAGTAGTATGTATGGTTACGGTTGTTTCAGCCGATGCATATGCTAACACTGAACTTAC 3200
ATCTGTAGGTCTGTTGAGATCAACGATCGATAGGTTGAAGCAAGAAAGGACCTTAACTCCCAGGCTTGCTTTTATTCATG 3280
GTGCGCATGATGATACAACAACATTTGAGAAATACCTGATTGAGGATCGGGCTCTGGATGGTACTCTGCTGGATAGCTCT 3360

```
                                                   3.380               3.400               3.420               3.440
Genomic(MLOC_37573)  ACAGGTTTCAGATCATTCCTTGAGGAGGTTAGAAGCAGAGTAACTGAGTTTGGAATATAGAGGCCTGTCTAAGCTGTTCA 3440
   cDNA(MLOC_37573)  ACAGGTTTCAGATCATTCCTTGAGGAGGTTAGAAGCAGAGTAACTGAGTTTGGAATATAGAGGCCTGTCTAAGCTGTTCA 1731
        CircularRNA  -------------------------------------------------------------------------------- 664
 Cir_Forward.Primer  -------------------------------------------------------------------------------- 21
 Cir_Reverse.Primer  -------------------------------------------------------------------------------- 25

                                                   3.460               3.480               3.500               3.520
Genomic(MLOC_37573)  ATATGCAAGATGGATGATTGTGGCCATCCTGGGCCTTTTGGCTAGGACCCGTTGTGTTGCTTAGCTTACTTTTGTGGTTG 3520
   cDNA(MLOC_37573)  ATATGCAAGATGGATGATTGTGGCCATCCTGGGCCTTTTGGCTAGGACCCGTTGTGTTGCTTAGCTTACTTTTGTGGTTG 1811
        CircularRNA  -------------------------------------------------------------------------------- 664
 Cir_Forward.Primer  -------------------------------------------------------------------------------- 21
 Cir_Reverse.Primer  -------------------------------------------------------------------------------- 25

                                                   3.540               3.560               3.580               3.600
Genomic(MLOC_37573)  CCAGGGGCACTCCATTGGTATGATATGAAACCAGAATGCGTACCTGGAGTTCATAGTCCATGTTGAATATTCATGTCTTG 3600
   cDNA(MLOC_37573)  CCAGGGGCACTCCATTGGTATGATATGAAACCAGAATGCGTACCTGGAGTTCATAGTCCATGTTGAATATTCATGTCTTG 1891
        CircularRNA  -------------------------------------------------------------------------------- 664
 Cir_Forward.Primer  -------------------------------------------------------------------------------- 21
 Cir_Reverse.Primer  -------------------------------------------------------------------------------- 25

                                                   3.620               3.640               3.660               3.680
Genomic(MLOC_37573)  AACATGAGTGTAATTTGTACATTGCCCAAACACACTTTGGTGACGATTTTGCTTTGTATGCTATTACTTGTTATAATGTT 3680
   cDNA(MLOC_37573)  AACATGAGTGTAATTTGTACATTGCCCAAACACACTTTGGTGACGATTTTGCTTTGTATGCTATTACTTGTTATAATGTT 1971
        CircularRNA  -------------------------------------------------------------------------------- 664
 Cir_Forward.Primer  -------------------------------------------------------------------------------- 21
 Cir_Reverse.Primer  -------------------------------------------------------------------------------- 25

                                                   3.700               3.720
Genomic(MLOC_37573)  TGTGCACTTGCTTTTTTGTATAGAAAAGTAAATATTGCCC 3720
   cDNA(MLOC_37573)  TGTGCACTTGCTTTTTTGTATAGAAAAGTAAATATTGCCC 2011
        CircularRNA  ---------------------------------------- 664
 Cir_Forward.Primer  ---------------------------------------- 21
 Cir_Reverse.Primer  ---------------------------------------- 25
```

Real-Time PCR for the junction region of Sec23/Sec24 transport protein_circular RNA (ID: Ch2:482080734-482081397**)**

By divergent primers ◀▶ on genomic DNA

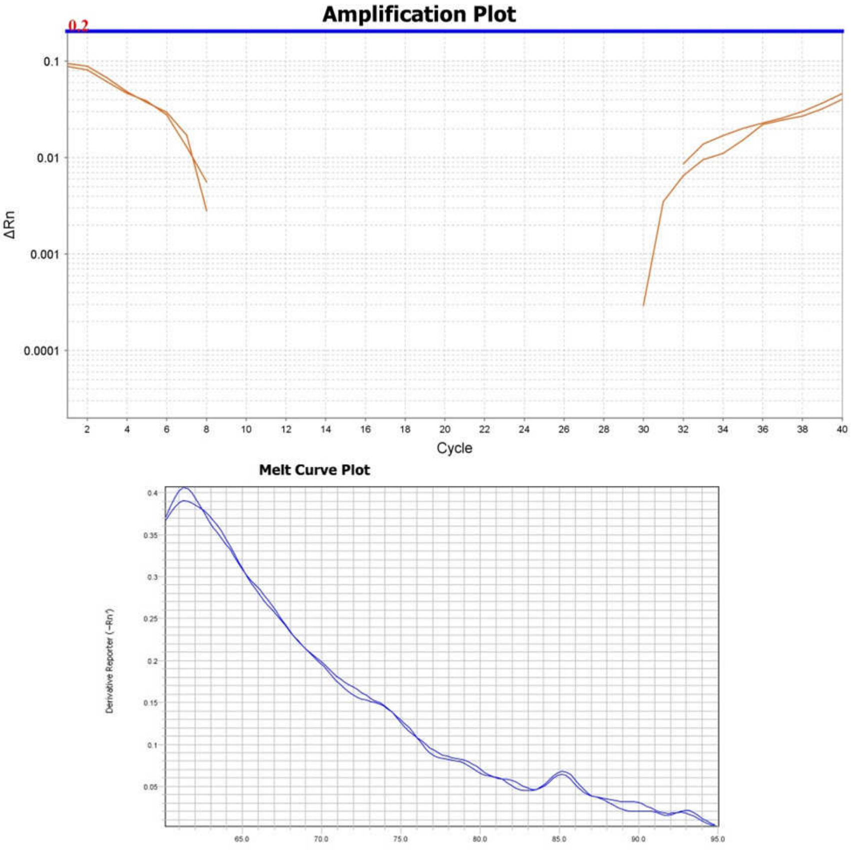

By divergent primers ◀▶ on cDNAs

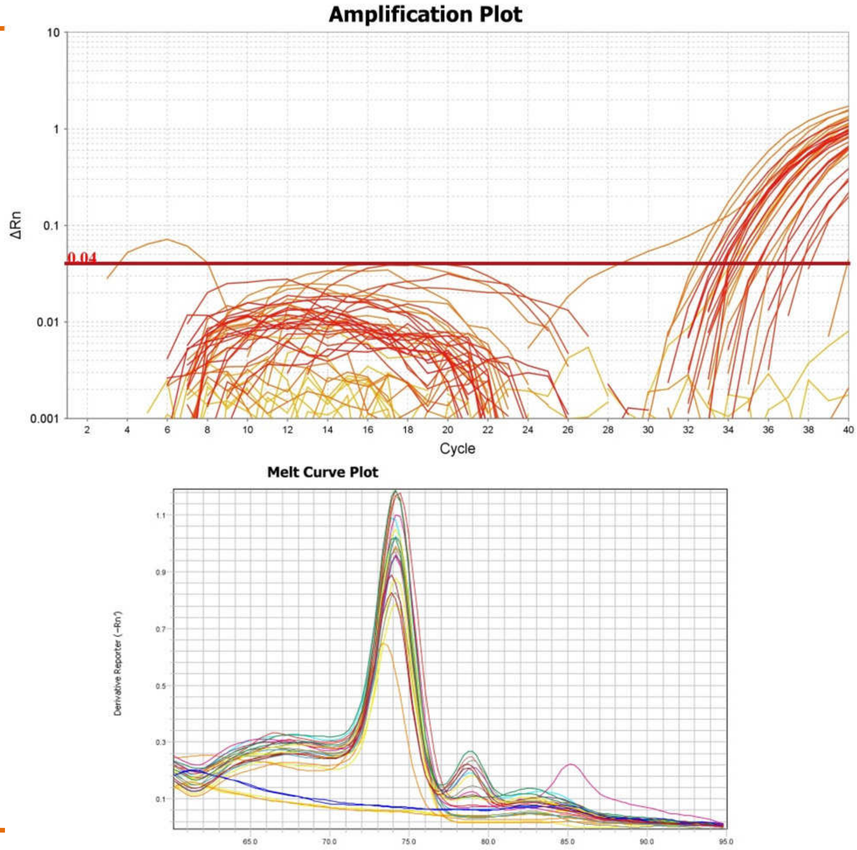

Real-Time PCR for the junction region of Sec23/Sec24 transport protein_circular RNA (ID: Ch2:482080734-482081397**)**

By divergent primers ◀▶ & with no template

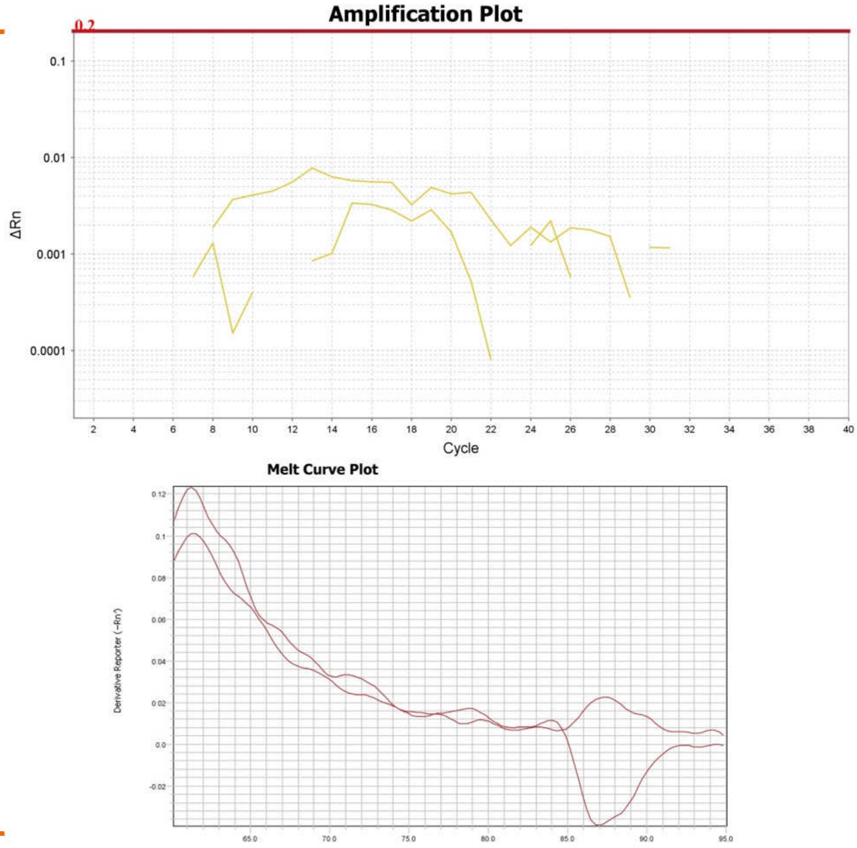

Fumarase 2 _circular RNA (ID: Ch4:243159573-243159671**)**

<u>T</u>CATTGAGATTATTAGGGGATGCATCTGTATCCTTTGAGAAAAACTGTGTCAGGGGAATAGAAG
CAAACCATAAGAGAATTTCACAATTGTTGCACGA<u>G</u>

The nucleotides of junction-region are underlined. The nucleotides of junction-region which are supported by the junction-spaning sequencing reads are shown in red. Introns are not shown if the absence is supported by sequencing reads. In the absence of supporting sequencing reads, the intronic nucletides are shown as N.

Structural relationship between the circular RNA and its parental gene

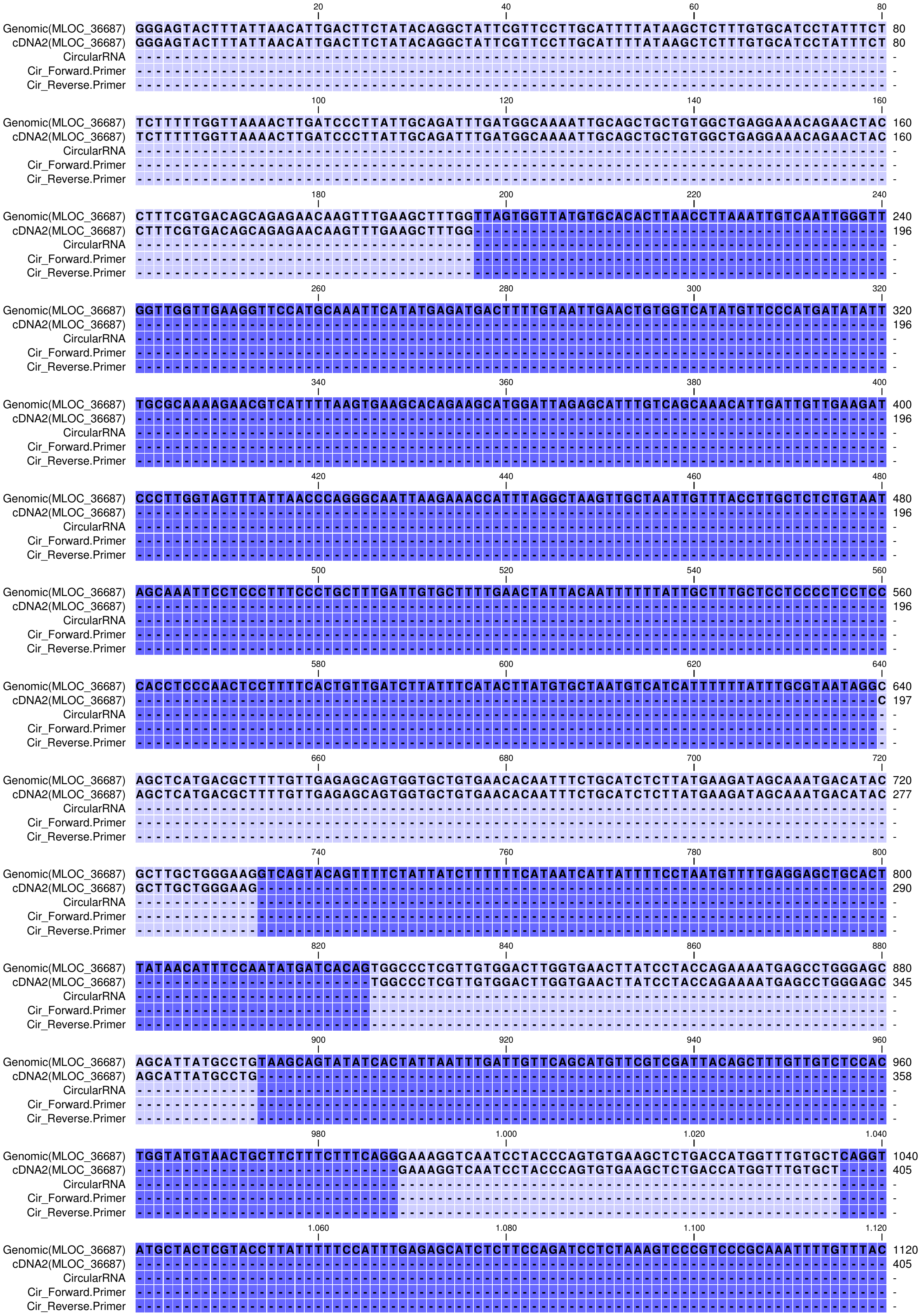

20 40 60 80
Genomic(MLOC_36687) GGGAGTACTTTATTAACATTGACTTCTATACAGGCTATTCGTTCCTTGCATTTTATAAGCTCTTTGTGCATCCTATTTCT 80
cDNA2(MLOC_36687) GGGAGTACTTTATTAACATTGACTTCTATACAGGCTATTCGTTCCTTGCATTTTATAAGCTCTTTGTGCATCCTATTTCT 80
CircularRNA -- -
Cir_Forward.Primer -- -
Cir_Reverse.Primer -- -
100 120 140 160
Genomic(MLOC_36687) TCTTTTTGGTTAAAACTTGATCCCTTATTGCAGATTTGATGGCAAAATTGCAGCTGCTGTGGCTGAGGAAACAGAACTAC 160
cDNA2(MLOC_36687) TCTTTTTGGTTAAAACTTGATCCCTTATTGCAGATTTGATGGCAAAATTGCAGCTGCTGTGGCTGAGGAAACAGAACTAC 160
CircularRNA -- -
Cir_Forward.Primer -- -
Cir_Reverse.Primer -- -
180 200 220 240
Genomic(MLOC_36687) CTTTCGTGACAGCAGAGAACAAGTTTGAAGCTTTGGTTAGTGGTTATGTGCACACTTAACCTTAAATTGTCAATTGGGTT 240
cDNA2(MLOC_36687) CTTTCGTGACAGCAGAGAACAAGTTTGAAGCTTTGG-- 196
CircularRNA -- -
Cir_Forward.Primer -- -
Cir_Reverse.Primer -- -
260 280 300 320
Genomic(MLOC_36687) GGTTGGTTGAAGGTTCCATGCAAATTCATATGAGATGACTTTTGTAATTGAACTGTGGTCATATGTTCCCATGATATATT 320
cDNA2(MLOC_36687) -- 196
CircularRNA -- -
Cir_Forward.Primer -- -
Cir_Reverse.Primer -- -
340 360 380 400
Genomic(MLOC_36687) TGCGCAAAAGAACGTCATTTTAAGTGAAGCACAGAAGCATGGATTAGAGCATTTGTCAGCAAACATTGATTGTTGAAGAT 400
cDNA2(MLOC_36687) -- 196
CircularRNA -- -
Cir_Forward.Primer -- -
Cir_Reverse.Primer -- -
420 440 460 480
Genomic(MLOC_36687) CCCTTGGTAGTTTATTAACCCAGGGCAATTAAGAAACCATTTAGGCTAAGTTGCTAATTGTTTACCTTGCTCTCTGTAAT 480
cDNA2(MLOC_36687) -- 196
CircularRNA -- -
Cir_Forward.Primer -- -
Cir_Reverse.Primer -- -
500 520 540 560
Genomic(MLOC_36687) AGCAAATTCCTCCCTTTCCCTGCTTTGATTGTGCTTTTGAACTATTACAATTTTTTATTGCTTTGCTCCTCCCCTCCTCC 560
cDNA2(MLOC_36687) -- 196
CircularRNA -- -
Cir_Forward.Primer -- -
Cir_Reverse.Primer -- -
580 600 620 640
Genomic(MLOC_36687) CACCTCCCAACTCCTTTTCACTGTTGATCTTATTTCATACTTATGTGCTAATGTCATCATTTTTTATTTGCGTAATAGGC 640
cDNA2(MLOC_36687) ---C 197
CircularRNA -- -
Cir_Forward.Primer -- -
Cir_Reverse.Primer -- -
660 680 700 720
Genomic(MLOC_36687) AGCTCATGACGCTTTTGTTGAGAGCAGTGGTGCTGTGAACACAATTTCTGCATCTCTTATGAAGATAGCAAATGACATAC 720
cDNA2(MLOC_36687) AGCTCATGACGCTTTTGTTGAGAGCAGTGGTGCTGTGAACACAATTTCTGCATCTCTTATGAAGATAGCAAATGACATAC 277
CircularRNA -- -
Cir_Forward.Primer -- -
Cir_Reverse.Primer -- -
740 760 780 800
Genomic(MLOC_36687) GCTTGCTGGGAAGGTCAGTACAGTTTTCTATTATCTTTTTTCATAATCATTATTTTCCTAATGTTTTGAGGAGCTGCACT 800
cDNA2(MLOC_36687) GCTTGCTGGGAAG--- 290
CircularRNA -- -
Cir_Forward.Primer -- -
Cir_Reverse.Primer -- -
820 840 860 880
Genomic(MLOC_36687) TATAACATTTCCAATATGATCACAGTGGCCCTCGTTGTGGACTTGGTGAACTTATCCTACCAGAAAATGAGCCTGGGAGC 880
cDNA2(MLOC_36687) -------------------------TGGCCCTCGTTGTGGACTTGGTGAACTTATCCTACCAGAAAATGAGCCTGGGAGC 345
CircularRNA -- -
Cir_Forward.Primer -- -
Cir_Reverse.Primer -- -
900 920 940 960
Genomic(MLOC_36687) AGCATTATGCCTGTAAGCAGTATATCACTATTAATTTGATTGTTCAGCATGTTCGTCGATTACAGCTTTGTTGTCTCCAC 960
cDNA2(MLOC_36687) AGCATTATGCCTG--- 358
CircularRNA -- -
Cir_Forward.Primer -- -
Cir_Reverse.Primer -- -
980 1.000 1.020 1.040
Genomic(MLOC_36687) TGGTATGTAACTGCTTCTTTCTTTCAGGGAAAGGTCAATCCTACCCAGTGTGAAGCTCTGACCATGGTTTGTGCTCAGGT 1040
cDNA2(MLOC_36687) ----------------------------GAAAGGTCAATCCTACCCAGTGTGAAGCTCTGACCATGGTTTGTGCT----- 405
CircularRNA -- -
Cir_Forward.Primer -- -
Cir_Reverse.Primer -- -
1.060 1.080 1.100 1.120
Genomic(MLOC_36687) ATGCTACTCGTACCTTATTTTTCCATTTGAGAGCATCTCTTCCAGATCCTCTAAAGTCCCGTCCCGCAAATTTTGTTTAC 1120
cDNA2(MLOC_36687) -- 405
CircularRNA -- -
Cir_Forward.Primer -- -
Cir_Reverse.Primer -- -

```
                                                        1.140                    1.160                    1.180                    1.200
Genomic(MLOC_36687)  GGTCCGCCTGTAAAAAAAATACGGTACTGCGAAGCCTCCGGCGGAACAGGTCCTGTAAACTCGTCCCGCAATTTTTTTTC 1200
cDNA2(MLOC_36687)    -------------------------------------------------------------------------------- 405
CircularRNA          -------------------------------------------------------------------------------- -
Cir_Forward.Primer   -------------------------------------------------------------------------------- -
Cir_Reverse.Primer   -------------------------------------------------------------------------------- -

                                                        1.220                    1.240                    1.260                    1.280
Genomic(MLOC_36687)  CGTGCCGGAGGCCTCACTGTACCGCAAAAAAATTCATAGGGCAAATATTGACAAGTGCTTGAATTCATATAGATGCACAA 1280
cDNA2(MLOC_36687)    -------------------------------------------------------------------------------- 405
CircularRNA          -------------------------------------------------------------------------------- -
Cir_Forward.Primer   -------------------------------------------------------------------------------- -
Cir_Reverse.Primer   -------------------------------------------------------------------------------- -

                                                        1.300                    1.320                    1.340                    1.360
Genomic(MLOC_36687)  GATTTATATATACATAGTCACACTAGTTCATCACATCTAAAATCATGAGCACCATACGAGGTAGCAAATTTAGCCATACA 1360
cDNA2(MLOC_36687)    -------------------------------------------------------------------------------- 405
CircularRNA          -------------------------------------------------------------------------------- -
Cir_Forward.Primer   -------------------------------------------------------------------------------- -
Cir_Reverse.Primer   -------------------------------------------------------------------------------- -

                                                        1.380                    1.400                    1.420                    1.440
Genomic(MLOC_36687)  AATGCACGATCAAACAAATAAAACATCAGCAAATGGCCACCGGTCATGAAGAAATGAAGCAACATCATCAATGTTGTGAA 1440
cDNA2(MLOC_36687)    -------------------------------------------------------------------------------- 405
CircularRNA          -------------------------------------------------------------------------------- -
Cir_Forward.Primer   -------------------------------------------------------------------------------- -
Cir_Reverse.Primer   -------------------------------------------------------------------------------- -

                                                        1.460                    1.480                    1.500                    1.520
Genomic(MLOC_36687)  GATCGTCGCCACCACCACTGGCCGTAGCATCTCCATCACCATTATCCACATTGACCGGAGAATTAGCACCACCATCTTCA 1520
cDNA2(MLOC_36687)    -------------------------------------------------------------------------------- 405
CircularRNA          -------------------------------------------------------------------------------- -
Cir_Forward.Primer   -------------------------------------------------------------------------------- -
Cir_Reverse.Primer   -------------------------------------------------------------------------------- -

                                                        1.540                    1.560                    1.580                    1.600
Genomic(MLOC_36687)  ACTTGCATCGGAGAATGAGCACCACCGTCACCATGAACACCGTTTGCCGAAGCACCACCGCCACCTCCACCACCATTGCC 1600
cDNA2(MLOC_36687)    -------------------------------------------------------------------------------- 405
CircularRNA          -------------------------------------------------------------------------------- -
Cir_Forward.Primer   -------------------------------------------------------------------------------- -
Cir_Reverse.Primer   -------------------------------------------------------------------------------- -

                                                        1.620                    1.640                    1.660                    1.680
Genomic(MLOC_36687)  AAAAGCTTCACCACCACCACCACCACCCCCAATGTCGAAGCCAACACCACCTCCAAACCCAACACCACCACCATTGCCCA 1680
cDNA2(MLOC_36687)    -------------------------------------------------------------------------------- 405
CircularRNA          -------------------------------------------------------------------------------- -
Cir_Forward.Primer   -------------------------------------------------------------------------------- -
Cir_Reverse.Primer   -------------------------------------------------------------------------------- -

                                                        1.700                    1.720                    1.740                    1.760
Genomic(MLOC_36687)  AGCCATCACCACCTCCAAACCCAACACCACCACCATTGCCCAAGCCATCACCACCTCCAAACCCTCCACCACCAATGATG 1760
cDNA2(MLOC_36687)    -------------------------------------------------------------------------------- 405
CircularRNA          -------------------------------------------------------------------------------- -
Cir_Forward.Primer   -------------------------------------------------------------------------------- -
Cir_Reverse.Primer   -------------------------------------------------------------------------------- -

                                                        1.780                    1.800                    1.820                    1.840
Genomic(MLOC_36687)  AAGCCCTGGAAGCAAAACCCCCTCCAAAGCCCACACCACCACGTACATTTGCCCAACCCAACGGCAGATTTTGAAGCTCA 1840
cDNA2(MLOC_36687)    -------------------------------------------------------------------------------- 405
CircularRNA          -------------------------------------------------------------------------------- -
Cir_Forward.Primer   -------------------------------------------------------------------------------- -
Cir_Reverse.Primer   -------------------------------------------------------------------------------- -

                                                        1.860                    1.880                    1.900                    1.920
Genomic(MLOC_36687)  TCTTTCTTGGGCACATGATCCTCCATTTTCTTCTCAATTCCCAACTTTTTTGTCATTGCATCCATGCCCCCCCGGGGATT 1920
cDNA2(MLOC_36687)    -------------------------------------------------------------------------------- 405
CircularRNA          -------------------------------------------------------------------------------- -
Cir_Forward.Primer   -------------------------------------------------------------------------------- -
Cir_Reverse.Primer   -------------------------------------------------------------------------------- -

                                                        1.940                    1.960                    1.980                    2.000
Genomic(MLOC_36687)  CATCATCATGAACCGGTCCTCCTCAACGGTCCTCTCGTCCCTCCTCTTTTGCTCCTCTAGTGTGATCTTGCGCGCCTCGA 2000
cDNA2(MLOC_36687)    -------------------------------------------------------------------------------- 405
CircularRNA          -------------------------------------------------------------------------------- -
Cir_Forward.Primer   -------------------------------------------------------------------------------- -
Cir_Reverse.Primer   -------------------------------------------------------------------------------- -

                                                        2.020                    2.040                    2.060                    2.080
Genomic(MLOC_36687)  AGGCACACCTTCTCTCCCACTTCTCCTTGTGCTTGTCTTTCTTCTCGGCCATGGCCACTTGAGCATCCCAATGCTTAGTT 2080
cDNA2(MLOC_36687)    -------------------------------------------------------------------------------- 405
CircularRNA          -------------------------------------------------------------------------------- -
Cir_Forward.Primer   -------------------------------------------------------------------------------- -
Cir_Reverse.Primer   -------------------------------------------------------------------------------- -

                                                        2.100                    2.120                    2.140                    2.160
Genomic(MLOC_36687)  AACATGGTTTCTTTCATCTTCGTCATGTCACCAATTTGATCTCTCAAAGTAGTTGCCTCGGCCAAGCTCTTTACTCTTGA 2160
cDNA2(MLOC_36687)    -------------------------------------------------------------------------------- 405
CircularRNA          -------------------------------------------------------------------------------- -
Cir_Forward.Primer   -------------------------------------------------------------------------------- -
Cir_Reverse.Primer   -------------------------------------------------------------------------------- -

                                                        2.180                    2.200                    2.220                    2.240
Genomic(MLOC_36687)  TTTCTCCTTGTTTCCATCCGGTTTGCCCTTGTCTCTTCCTCCTCTTGGTGCATCATCACTATCATCATCCTCATCTTCCA 2240
cDNA2(MLOC_36687)    -------------------------------------------------------------------------------- 405
CircularRNA          -------------------------------------------------------------------------------- -
Cir_Forward.Primer   -------------------------------------------------------------------------------- -
Cir_Reverse.Primer   -------------------------------------------------------------------------------- -
```

```
                    2.260               2.280               2.300               2.320
Genomic(MLOC_36687) ATTTGATAAGAGGACCTTTCTTTGAGTGAGCTTGCTTGTCCCTCACCCTCCACTTCTCATATCCTTGAGCAAGCGCCAAC 2320
cDNA2(MLOC_36687)   -------------------------------------------------------------------------------- 405
CircularRNA         -------------------------------------------------------------------------------- -
Cir_Forward.Primer  -------------------------------------------------------------------------------- -
Cir_Reverse.Primer  -------------------------------------------------------------------------------- -

                    2.340               2.360               2.380               2.400
Genomic(MLOC_36687) AATGATGAAGAGTGAACGGCAGGCCCTTGAAACCCGCGGTTTGCATTAAATATTCATTGGTGATGCGGTCCTACACATGG 2400
cDNA2(MLOC_36687)   -------------------------------------------------------------------------------- 405
CircularRNA         -------------------------------------------------------------------------------- -
Cir_Forward.Primer  -------------------------------------------------------------------------------- -
Cir_Reverse.Primer  -------------------------------------------------------------------------------- -

                    2.420               2.440               2.460               2.480
Genomic(MLOC_36687) GCATTGAGGCAATTATGAACTCAAACTTTTATCAACTCGGACAAAGTCAACAAAATGAAAAAACACTTACATAATCATCA 2480
cDNA2(MLOC_36687)   -------------------------------------------------------------------------------- 405
CircularRNA         -------------------------------------------------------------------------------- -
Cir_Forward.Primer  -------------------------------------------------------------------------------- -
Cir_Reverse.Primer  -------------------------------------------------------------------------------- -

                    2.500               2.520               2.540               2.560
Genomic(MLOC_36687) ATAGCGATGCCACTTGGAGGTGCATTCCGCACTTGCTCCAAGCACCTACTCCAACGGCCACATGTTTTCTTGATCACGTC 2560
cDNA2(MLOC_36687)   -------------------------------------------------------------------------------- 405
CircularRNA         -------------------------------------------------------------------------------- -
Cir_Forward.Primer  -------------------------------------------------------------------------------- -
Cir_Reverse.Primer  -------------------------------------------------------------------------------- -

                    2.580               2.600               2.620               2.640
Genomic(MLOC_36687) CCAATGGCCTTGGATTGACTTTAATGATCGGCCGGAGGGGAACGGCAAGATCCGGTGGTACTTGTCCTTGATCCTTTGCC 2640
cDNA2(MLOC_36687)   -------------------------------------------------------------------------------- 405
CircularRNA         -------------------------------------------------------------------------------- -
Cir_Forward.Primer  -------------------------------------------------------------------------------- -
Cir_Reverse.Primer  -------------------------------------------------------------------------------- -

                    2.660               2.680               2.700               2.720
Genomic(MLOC_36687) AATAACTCCCAATGCTTTGATCTTTGCCGATCATCGGGTCAAGTGATACACTCTCCCATGCTTGAATCAAGCACACATCC 2720
cDNA2(MLOC_36687)   -------------------------------------------------------------------------------- 405
CircularRNA         -------------------------------------------------------------------------------- -
Cir_Forward.Primer  -------------------------------------------------------------------------------- -
Cir_Reverse.Primer  -------------------------------------------------------------------------------- -

                    2.740               2.760               2.780               2.800
Genomic(MLOC_36687) TCCGTCTTCGTGTAGTTGCCGGTCCTCCCCACTCTAGCATCGTACTCGACCTCCTCAATCTCTTCATTTCATCACCATTG 2800
cDNA2(MLOC_36687)   -------------------------------------------------------------------------------- 405
CircularRNA         -------------------------------------------------------------------------------- -
Cir_Forward.Primer  -------------------------------------------------------------------------------- -
Cir_Reverse.Primer  -------------------------------------------------------------------------------- -

                    2.820               2.840               2.860               2.880
Genomic(MLOC_36687) TCATTTGCTTGGTCCACCTCCGCCCTGACCTCCACTTGAGGCGTATCGAAATCGTCAAGGGGAGCACTCAAAACCACCTC 2880
cDNA2(MLOC_36687)   -------------------------------------------------------------------------------- 405
CircularRNA         -------------------------------------------------------------------------------- -
Cir_Forward.Primer  -------------------------------------------------------------------------------- -
Cir_Reverse.Primer  -------------------------------------------------------------------------------- -

                    2.900               2.920               2.940               2.960
Genomic(MLOC_36687) ACTCTCCTCCAACATTTAGATATATGTTGACTGCATGTTTTGGCTATAAAATTCAACGGGCAAAATGTATACAAGTGCTT 2960
cDNA2(MLOC_36687)   -------------------------------------------------------------------------------- 405
CircularRNA         -------------------------------------------------------------------------------- -
Cir_Forward.Primer  -------------------------------------------------------------------------------- -
Cir_Reverse.Primer  -------------------------------------------------------------------------------- -

                    2.980               3.000               3.020               3.040
Genomic(MLOC_36687) CAAAGTCAAACCAAAGATCACCTGAGCGTTTGAAGCACAATATTATTTTTTTACCTCCAGAACTTTCATCGGTCTTGTTG 3040
cDNA2(MLOC_36687)   -------------------------------------------------------------------------------- 405
CircularRNA         -------------------------------------------------------------------------------- -
Cir_Forward.Primer  -------------------------------------------------------------------------------- -
Cir_Reverse.Primer  -------------------------------------------------------------------------------- -

                    3.060               3.080               3.100               3.120
Genomic(MLOC_36687) TAGGCCTGACCGGTCGAGTGGCCGTGATCGGGGAGAATGTGGGCGGCCGGATGAGGAGGAGCCCGGCCTCTTGCCACGAC 3120
cDNA2(MLOC_36687)   -------------------------------------------------------------------------------- 405
CircularRNA         -------------------------------------------------------------------------------- -
Cir_Forward.Primer  -------------------------------------------------------------------------------- -
Cir_Reverse.Primer  -------------------------------------------------------------------------------- -

                    3.140               3.160               3.180               3.200
Genomic(MLOC_36687) CGCCTTCTTCCTCGGGGGCTTCGCCCGCACACACTCTTGCCACAACCTCCTTCCTCGGGGGCTTCGCTCGCACACCGGCC 3200
cDNA2(MLOC_36687)   -------------------------------------------------------------------------------- 405
CircularRNA         -------------------------------------------------------------------------------- -
Cir_Forward.Primer  -------------------------------------------------------------------------------- -
Cir_Reverse.Primer  -------------------------------------------------------------------------------- -

                    3.220               3.240               3.260               3.280
Genomic(MLOC_36687) GGGCCGGTGGAGGCGGTCTTCTTCCTTGTGTCAGCGGCCGCGACGGGAGGAACCGGAGCGAGGGAGGAAGGAGGTGTTGC 3280
cDNA2(MLOC_36687)   -------------------------------------------------------------------------------- 405
CircularRNA         -------------------------------------------------------------------------------- -
Cir_Forward.Primer  -------------------------------------------------------------------------------- -
Cir_Reverse.Primer  -------------------------------------------------------------------------------- -

                    3.300               3.320               3.340               3.360
Genomic(MLOC_36687) ACGGGGTGATACTCCTGCCGCTTCTGCTTGGCGCCGGCGGTGAAAGATGTTGGTGCGGCGCTGGCATGCATGGTCGAGGG 3360
cDNA2(MLOC_36687)   -------------------------------------------------------------------------------- 405
CircularRNA         -------------------------------------------------------------------------------- -
Cir_Forward.Primer  -------------------------------------------------------------------------------- -
Cir_Reverse.Primer  -------------------------------------------------------------------------------- -
```

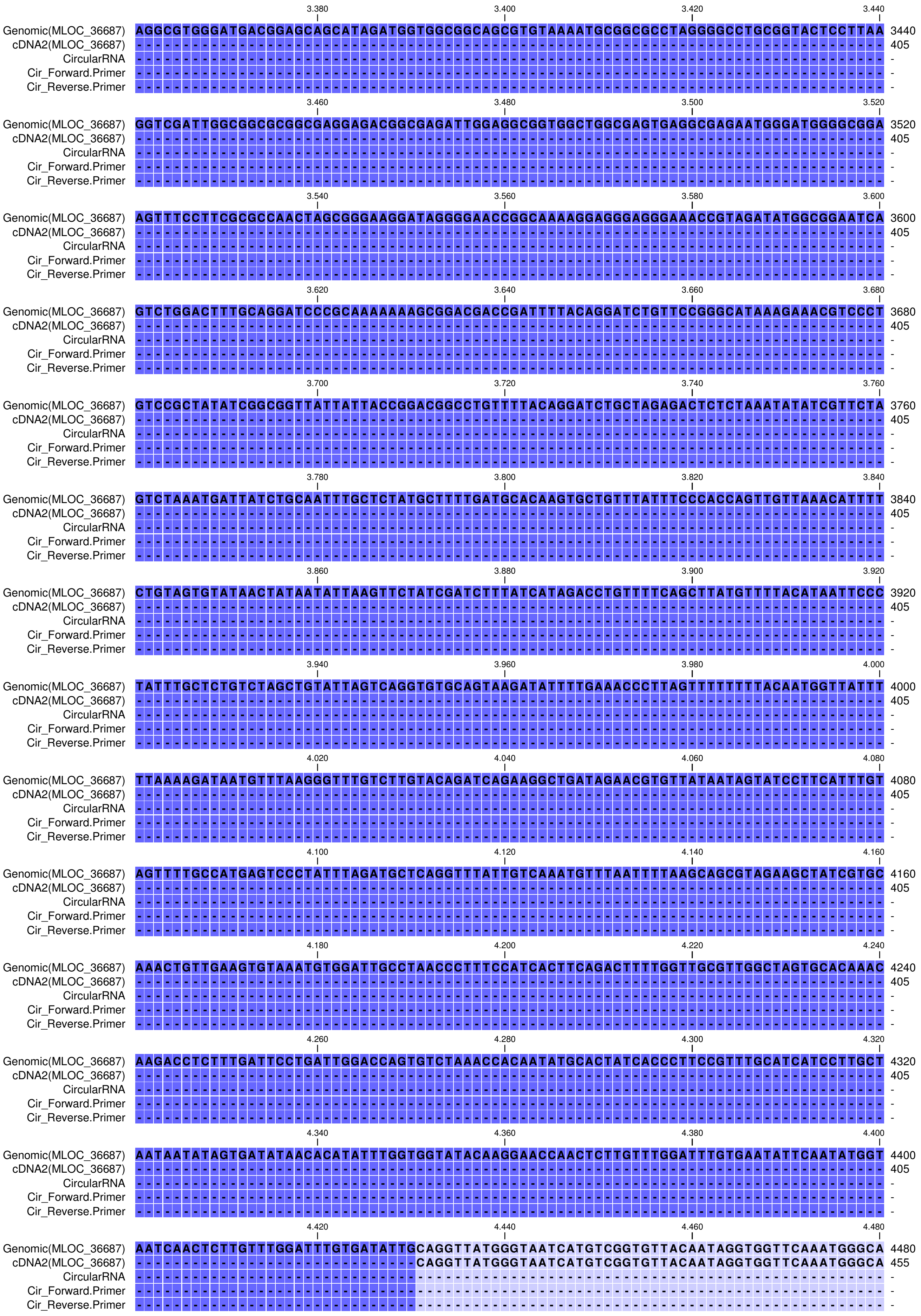
Genomic(MLOC_36687)
cDNA2(MLOC_36687)
CircularRNA
Cir_Forward.Primer
Cir_Reverse.Primer
3.380 3.400 3.420 3.440
AGGCGTGGGATGACGGAGCAGCATAGATGGTGGCGGCAGCGTGTAAAATGCGGCGCCTAGGGGCCTGCGGTACTCCTTAA 3440
405
3.460 3.480 3.500 3.520
GGTCGATTGGCGGCGCGGCGAGGAGACGGCGAGATTGGAGGCGGTGGCTGGCGAGTGAGGCGAGAATGGGATGGGGCGGA 3520
405
3.540 3.560 3.580 3.600
AGTTTCCTTCGCGCCAACTAGCGGGAAGGATAGGGGAACCGGCAAAAGGAGGGAGGGAAACCGTAGATATGGCGGAATCA 3600
405
3.620 3.640 3.660 3.680
GTCTGGACTTTGCAGGATCCCGCAAAAAAAGCGGACGACCGATTTTACAGGATCTGTTCCGGGCATAAAGAAACGTCCCT 3680
405
3.700 3.720 3.740 3.760
GTCCGCTATATCGGCGGTTATTATTACCGGACGGCCTGTTTTACAGGATCTGCTAGAGACTCTCTAAATATATCGTTCTA 3760
405
3.780 3.800 3.820 3.840
GTCTAAATGATTATCTGCAATTTGCTCTATGCTTTTGATGCACAAGTGCTGTTTATTTCCCACCAGTTGTTAAACATTTT 3840
405
3.860 3.880 3.900 3.920
CTGTAGTGTATAACTATAATATTAAGTTCTATCGATCTTTATCATAGACCTGTTTTCAGCTTATGTTTTACATAATTCCC 3920
405
3.940 3.960 3.980 4.000
TATTTGCTCTGTCTAGCTGTATTAGTCAGGTGTGCAGTAAGATATTTTGAAACCCTTAGTTTTTTTTACAATGGTTATTT 4000
405
4.020 4.040 4.060 4.080
TTAAAAGATAATGTTTAAGGGTTTGTCTTGTACAGATCAGAAGGCTGATAGAACGTGTTATAATAGTATCCTTCATTTGT 4080
405
4.100 4.120 4.140 4.160
AGTTTTGCCATGAGTCCCTATTTAGATGCTCAGGTTTATTGTCAAATGTTTAATTTTAAGCAGCGTAGAAGCTATCGTGC 4160
405
4.180 4.200 4.220 4.240
AAACTGTTGAAGTGTAAATGTGGATTGCCTAACCCTTTCCATCACTTCAGACTTTTGGTTGCGTTGGCTAGTGCACAAAC 4240
405
4.260 4.280 4.300 4.320
AAGACCTCTTTGATTCCTGATTGGACCAGTGTCTAAACCACAATATGCACTATCACCCTTCCGTTTGCATCATCCTTGCT 4320
405
4.340 4.360 4.380 4.400
AATAATATAGTGATATAACACATATTTGGTGGTATACAAGGAACCAACTCTTGTTTGGATTTGTGAATATTCAATATGGT 4400
405
4.420 4.440 4.460 4.480
AATCAACTCTTGTTTGGATTTGTGATATTGCAGGTTATGGGTAATCATGTCGGTGTTACAATAGGTGGTTCAAATGGGCA 4480
CAGGTTATGGGTAATCATGTCGGTGTTACAATAGGTGGTTCAAATGGGCA 455

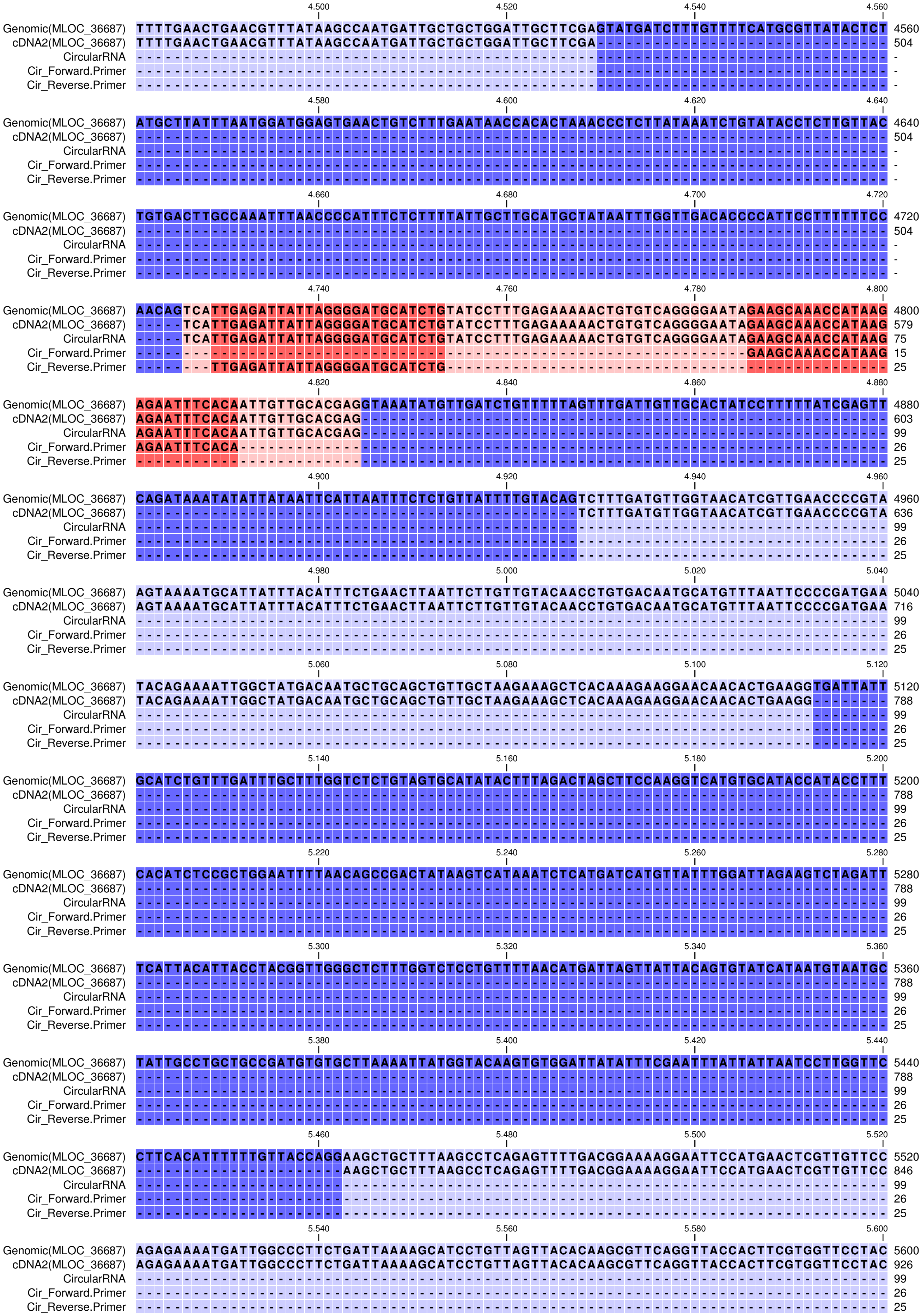

Genomic(MLOC_36687)
cDNA2(MLOC_36687)
CircularRNA
Cir_Forward.Primer
Cir_Reverse.Primer

```
                       5.620               5.640               5.660               5.680
                            |                   |                   |                   |
Genomic(MLOC_36687)  CCCATCTGTAAATAAGGCAAGCCAGATTAAACATCTTCATTTCCCCATGAGGAGAGTTAATTTTGCACAACTAATGATGC 5680
cDNA2(MLOC_36687)    CCCATCTGTAAATAAGGCAAGCCAGATTAAACATCTTCATTTCCCCATGAGGAGAGTTAATTTTGCACAACTAATGATGC 1006
CircularRNA          -------------------------------------------------------------------------------- 99
Cir_Forward.Primer   -------------------------------------------------------------------------------- 26
Cir_Reverse.Primer   -------------------------------------------------------------------------------- 25

                       5.700               5.720               5.740               5.760
                            |                   |                   |                   |
Genomic(MLOC_36687)  CGGAGAAAAACATTTTTTCCTGTTAATCATACAGGAAGTGTGCTTTGATTTATTCGAATAGTTTTTTTTCTTGGATATTA 5760
cDNA2(MLOC_36687)    CGGAGAAAAACATTTTTTCCTGTT-------------------------------------------------------- 1030
CircularRNA          -------------------------------------------------------------------------------- 99
Cir_Forward.Primer   -------------------------------------------------------------------------------- 26
Cir_Reverse.Primer   -------------------------------------------------------------------------------- 25

                       5.780               5.800
                            |                   |
Genomic(MLOC_36687)  TCAGCATGCCAACTTTTACCCAATGCGGCGAATGTTCATGTAATAAGTGCTGCG 5814
cDNA2(MLOC_36687)    ------------------------------------------------------ 1030
CircularRNA          ------------------------------------------------------ 99
Cir_Forward.Primer   ------------------------------------------------------ 26
Cir_Reverse.Primer   ------------------------------------------------------ 25
```

Real-Time PCR for the junction region of fumarase 2 _circular RNA (ID: Ch4:243159573-243159671**)**

By divergent primers ◀▶ on genomic DNA

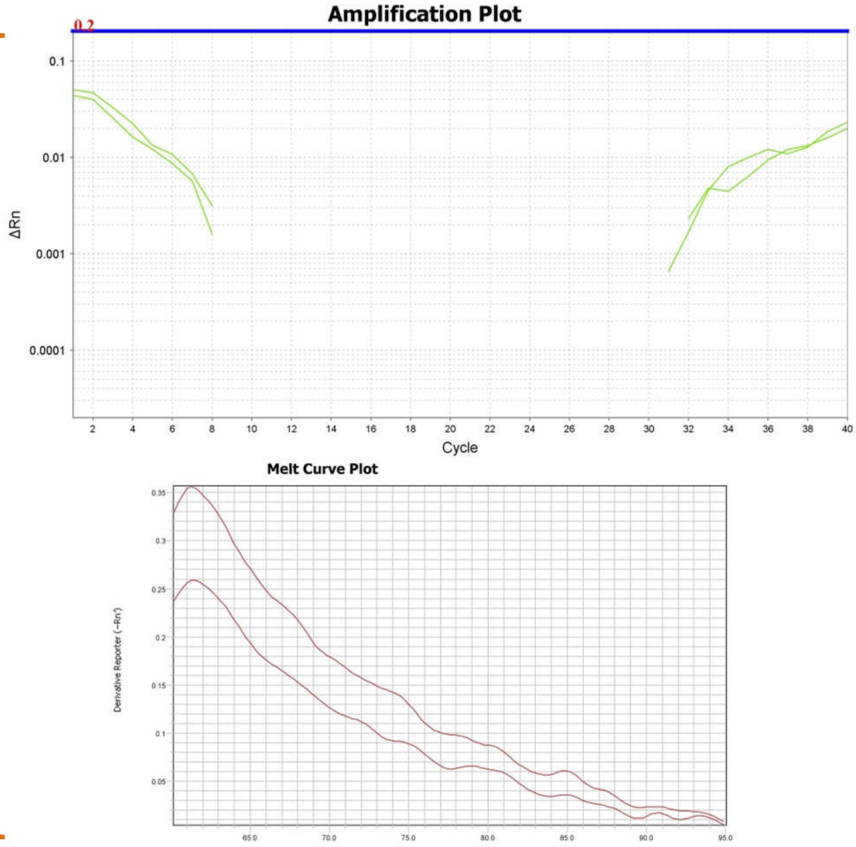

By divergent primers ◀▶& with no template

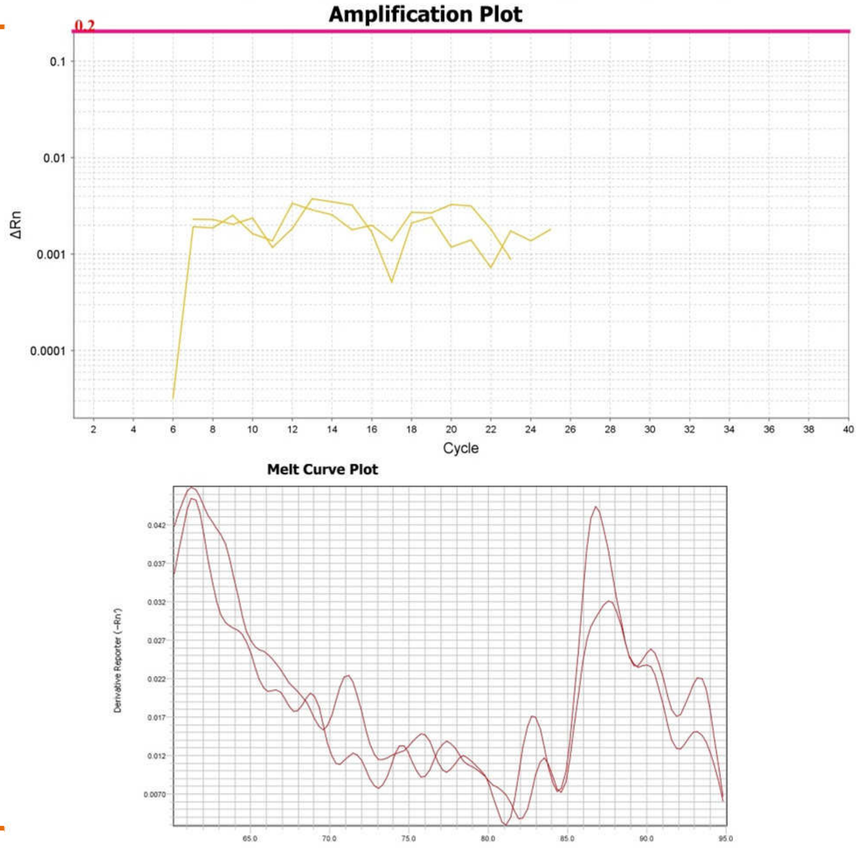

Inositol transporter 2_circular RNA (ID: Ch2:483514445-483514888)

GAAATGATCGTGAGCATGGCGGTGGCCGGCGCCATCATTGGCGCGGCGGTCGGCGGCTGGG
CCAACGACCGGTTCGGGCGGCGAACGTCCATCCTCGTGGCCGACTTGCTCTTTTTTGCGGGCG
CGGTGGTCATGGCTTCGGCGACGGGCCCAGTGCAGCTCGTGGTCGGCCGCGTCTTCGTCGGC
CTAGGCGTCGGCATGGCATCCATGACGGCGCCGCTTTACATCTCCGAGGCGTCGCCGGCGAG
GATCAGGGGCGCGCTTGTGAGTACCAACGGCTTCCTCATCACCGGCGGCCAGTTCTTGTCCTA
CCTTATCAACCTCGCATTCACCAAGGCGCCGGGGACGTGGAGGTGGATGCTCGGAGTCGCCG
GCCTCCCTGCCGTGGTCCAGTTCGTCCTCATG**CTATTCCTCCCCGAATCACCAAGATGGCTTT
ACAGAAAG**

The nucleotides of junction-region are underlined. The nucleotides of junction-region which are supported by the junction-spaning sequencing reads are shown in red. Introns are not shown if the absence is supported by sequencing reads. In the absence of supporting sequencing reads, the intronic nucletides are shown as N.

Structural relationship between the circular RNA and its parental gene

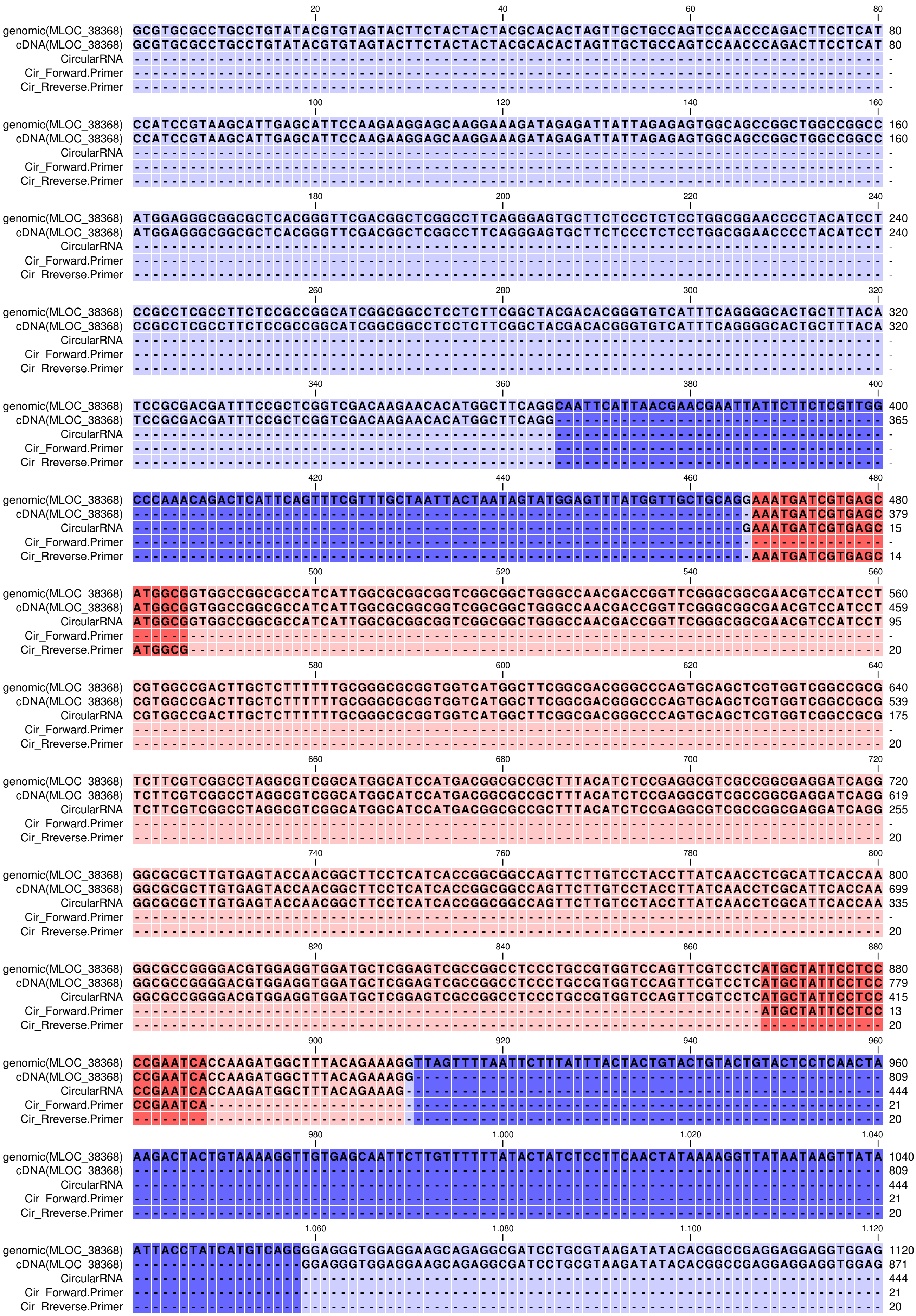
genomic(MLOC_38368)
cDNA(MLOC_38368)
CircularRNA
Cir_Forward.Primer
Cir_Rreverse.Primer

```
                                      1.140                   1.160                   1.180                   1.200
genomic(MLOC_38368)  AGGGAGATGCAGGAGTTGAAGGAGTCGGTGGAAGCGGAGGCGCGGGAGCGGGGCTCGTCGGAGAAGGTAAGCCTGACGGC 1200
cDNA(MLOC_38368)     AGGGAGATGCAGGAGTTGAAGGAGTCGGTGGAAGCGGAGGCGCGGGAGCGGGGCTCGTCGGAGAAGGTAAGCCTGACGGC 951
CircularRNA          -------------------------------------------------------------------------------- 444
Cir_Forward.Primer   -------------------------------------------------------------------------------- 21
Cir_Rreverse.Primer  -------------------------------------------------------------------------------- 20

                                      1.220                   1.240                   1.260                   1.280
genomic(MLOC_38368)  GCTGGTGAAGACGGCAACTGTGCGGCGGGCGCTGGTTGCCGGCGTCGGGCTGCAGGTGTTCCAGCAGCTCGTGGGTATCA 1280
cDNA(MLOC_38368)     GCTGGTGAAGACGGCAACTGTGCGGCGGGCGCTGGTTGCCGGCGTCGGGCTGCAGGTGTTCCAGCAGCTCGTGGGTATCA 1031
CircularRNA          -------------------------------------------------------------------------------- 444
Cir_Forward.Primer   -------------------------------------------------------------------------------- 21
Cir_Rreverse.Primer  -------------------------------------------------------------------------------- 20

                                      1.300                   1.320                   1.340                   1.360
genomic(MLOC_38368)  ACACCGTGATGTACTACAGCCCGAGCATCGTGCAGCTCGCCGGGTTCGCCTCCAACCAAACGGCGCTCGCGCTCTCGCTG 1360
cDNA(MLOC_38368)     ACACCGTGATGTACTACAGCCCGAGCATCGTGCAGCTCGCCGGGTTCGCCTCCAACCAAACGGCGCTCGCGCTCTCGCTG 1111
CircularRNA          -------------------------------------------------------------------------------- 444
Cir_Forward.Primer   -------------------------------------------------------------------------------- 21
Cir_Rreverse.Primer  -------------------------------------------------------------------------------- 20

                                      1.380                   1.400                   1.420                   1.440
genomic(MLOC_38368)  GTCACCTCCGGCCTCAACGCGCTCGGCTCCATCGTTAGCATCTACTTCATCGACCGCACCGGCCGGAGGAAGCTGCTGGT 1440
cDNA(MLOC_38368)     GTCACCTCCGGCCTCAACGCGCTCGGCTCCATCGTTAGCATCTACTTCATCGACCGCACCGGCCGGAGGAAGCTGCTGGT 1191
CircularRNA          -------------------------------------------------------------------------------- 444
Cir_Forward.Primer   -------------------------------------------------------------------------------- 21
Cir_Rreverse.Primer  -------------------------------------------------------------------------------- 20

                                      1.460                   1.480                   1.500                   1.520
genomic(MLOC_38368)  GATAAGCCTCGTCGGCGTCATCGCGTCGCTTGCCCTGCTCTCCGCGGTGTTCCACGAGACCACCTCACACTCGCCCGCCG 1520
cDNA(MLOC_38368)     GATAAGCCTCGTCGGCGTCATCGCGTCGCTTGCCCTGCTCTCCGCGGTGTTCCACGAGACCACCTCACACTCGCCCGCCG 1271
CircularRNA          -------------------------------------------------------------------------------- 444
Cir_Forward.Primer   -------------------------------------------------------------------------------- 21
Cir_Rreverse.Primer  -------------------------------------------------------------------------------- 20

                                      1.540                   1.560                   1.580                   1.600
genomic(MLOC_38368)  TCGGCAGCGCTGAGACCGGCCACTTCGATGGCTCGCTCACATGCCCGGACTACCGGACGACGAGCAGCTCCGGCTGGGAG 1600
cDNA(MLOC_38368)     TCGGCAGCGCTGAGACCGGCCACTTCGATGGCTCGCTCACATGCCCGGACTACCGGACGACGAGCAGCTCCGGCTGGGAG 1351
CircularRNA          -------------------------------------------------------------------------------- 444
Cir_Forward.Primer   -------------------------------------------------------------------------------- 21
Cir_Rreverse.Primer  -------------------------------------------------------------------------------- 20

                                      1.620                   1.640                   1.660                   1.680
genomic(MLOC_38368)  TGGGACTGCACCCGGTGCCTCAAGGCCAGCTCGACGGAGTGCGGCTTCTGCGCGTCCGGCGCCGGCAGCAAGCTGCTCCC 1680
cDNA(MLOC_38368)     TGGGACTGCACCCGGTGCCTCAAGGCCAGCTCGACGGAGTGCGGCTTCTGCGCGTCCGGCGCCGGCAGCAAGCTGCTCCC 1431
CircularRNA          -------------------------------------------------------------------------------- 444
Cir_Forward.Primer   -------------------------------------------------------------------------------- 21
Cir_Rreverse.Primer  -------------------------------------------------------------------------------- 20

                                      1.700                   1.720                   1.740                   1.760
genomic(MLOC_38368)  GGGCGCGTGCCTGCTGTCGAACGCGACGGTGCGCGACGCGTGCCACGGCGAGGGGCGTCTCTGGTACACGCGCGGCTGCC 1760
cDNA(MLOC_38368)     GGGCGCGTGCCTGCTGTCGAACGCGACGGTGCGCGACGCGTGCCACGGCGAGGGGCGTCTCTGGTACACGCGCGGCTGCC 1511
CircularRNA          -------------------------------------------------------------------------------- 444
Cir_Forward.Primer   -------------------------------------------------------------------------------- 21
Cir_Rreverse.Primer  -------------------------------------------------------------------------------- 20

                                      1.780                   1.800                   1.820                   1.840
genomic(MLOC_38368)  CGAGCCGATACGGTTGGCTGGCCATGGTCGGGCTAGCTCTGTACATCTCCTTCTTCTCGCCGGGGATGGGCACGGTGCCC 1840
cDNA(MLOC_38368)     CGAGCCGATACGGTTGGCTGGCCATGGTCGGGCTAGCTCTGTACATCTCCTTCTTCTCGCCGGGGATGGGCACGGTGCCC 1591
CircularRNA          -------------------------------------------------------------------------------- 444
Cir_Forward.Primer   -------------------------------------------------------------------------------- 21
Cir_Rreverse.Primer  -------------------------------------------------------------------------------- 20

                                      1.860                   1.880                   1.900                   1.920
genomic(MLOC_38368)  TGGATCGTCAACTCGGAGATCTACCCGCTGCGGCACCGCGGTGTGTGCGGTGGCGTGGCGGCCACCGCGAACTGGGTGTC 1920
cDNA(MLOC_38368)     TGGATCGTCAACTCGGAGATCTACCCGCTGCGGCACCGCGGTGTGTGCGGTGGCGTGGCGGCCACCGCGAACTGGGTGTC 1671
CircularRNA          -------------------------------------------------------------------------------- 444
Cir_Forward.Primer   -------------------------------------------------------------------------------- 21
Cir_Rreverse.Primer  -------------------------------------------------------------------------------- 20

                                      1.940                   1.960                   1.980                   2.000
genomic(MLOC_38368)  CAACCTGGTAGTGGCGCAGTCCTTCCTGTCGCTGACGGAGGCCATCGGTCCCGCGTGGACATTCCTCATCTTCGGTGTCC 2000
cDNA(MLOC_38368)     CAACCTGGTAGTGGCGCAGTCCTTCCTGTCGCTGACGGAGGCCATCGGTCCCGCGTGGACATTCCTCATCTTCGGTGTCC 1751
CircularRNA          -------------------------------------------------------------------------------- 444
Cir_Forward.Primer   -------------------------------------------------------------------------------- 21
Cir_Rreverse.Primer  -------------------------------------------------------------------------------- 20

                                      2.020                   2.040                   2.060                   2.080
genomic(MLOC_38368)  TGTCCGTGGCAGCGCTCGCGTTCGTGCTCGTCTGCGTGCCGGAGACCAAGGGACTCCCCATCGAGGAGGTGGAGAAGATG 2080
cDNA(MLOC_38368)     TGTCCGTGGCAGCGCTCGCGTTCGTGCTCGTCTGCGTGCCGGAGACCAAGGGACTCCCCATCGAGGAGGTGGAGAAGATG 1831
CircularRNA          -------------------------------------------------------------------------------- 444
Cir_Forward.Primer   -------------------------------------------------------------------------------- 21
Cir_Rreverse.Primer  -------------------------------------------------------------------------------- 20

                                      2.100                   2.120                   2.140                   2.160
genomic(MLOC_38368)  CTGGAGAAGCGGGAGCTCAGGCTCAAGTTCTGGGCACCGCGCGGCCGCGGCAGCAAGAACGACGGTGTGTGAGTGTCATG 2160
cDNA(MLOC_38368)     CTGGAGAAGCGGGAGCTCAGGCTCAAGTTCTGGGCACCGCGCGGCCGCGGCAGCAAGAACGACGGTGTGTGAGTGTCATG 1911
CircularRNA          -------------------------------------------------------------------------------- 444
Cir_Forward.Primer   -------------------------------------------------------------------------------- 21
Cir_Rreverse.Primer  -------------------------------------------------------------------------------- 20

                                      2.180                   2.200                   2.220                   2.240
genomic(MLOC_38368)  TCATCCCTTCCTTCTTTATTTAGCGCGCGAACCAGACAATTTGTGGCGATCCACGTAACTGGAATTCCGATTTGTAGGTT 2240
cDNA(MLOC_38368)     TCATCCCTTCCTTCTTTATTTAGCGCGCGAACCAGACAATTTGTGGCGATCCACGTAACTGGAATTCCGATTTGTAGGTT 1991
CircularRNA          -------------------------------------------------------------------------------- 444
Cir_Forward.Primer   -------------------------------------------------------------------------------- 21
Cir_Rreverse.Primer  -------------------------------------------------------------------------------- 20
```

```
                                                  2.260               2.280               2.300               2.320
                                                  |                   |                   |                   |
genomic(MLOC_38368)   TCTACTACTACCTCTGCAACTAAATACGGAGTATAAAGATGTTTCTGCAGTTTAAATTGTAATTGTATTGTAAAAACGTC 2320
cDNA(MLOC_38368)      TCTACTACTACCTCTGCAACTAAATACGGAGTATAAAGATGTTTCTGCAGTTTAAATTGTAATTGTATTGTAAAAACGTC 2071
CircularRNA           -------------------------------------------------------------------------------- 444
Cir_Forward.Primer    -------------------------------------------------------------------------------- 21
Cir_Rreverse.Primer   -------------------------------------------------------------------------------- 20

                                                  2.340
                                                  |
genomic(MLOC_38368)   TTATACATAGAAAACGGAGGTACGACTATTTTAAAG 2356
cDNA(MLOC_38368)      TTATACATAGAAAACGGAGGTACGACTATTTTAAAG 2107
CircularRNA           ------------------------------------ 444
Cir_Forward.Primer    ------------------------------------ 21
Cir_Rreverse.Primer   ------------------------------------ 20
```

Real-Time PCR for the junction region of inositol transporter 2_circular RNA (ID: Ch2:483514445-483514888)

By divergent primers ◀▶ on genomic DNA

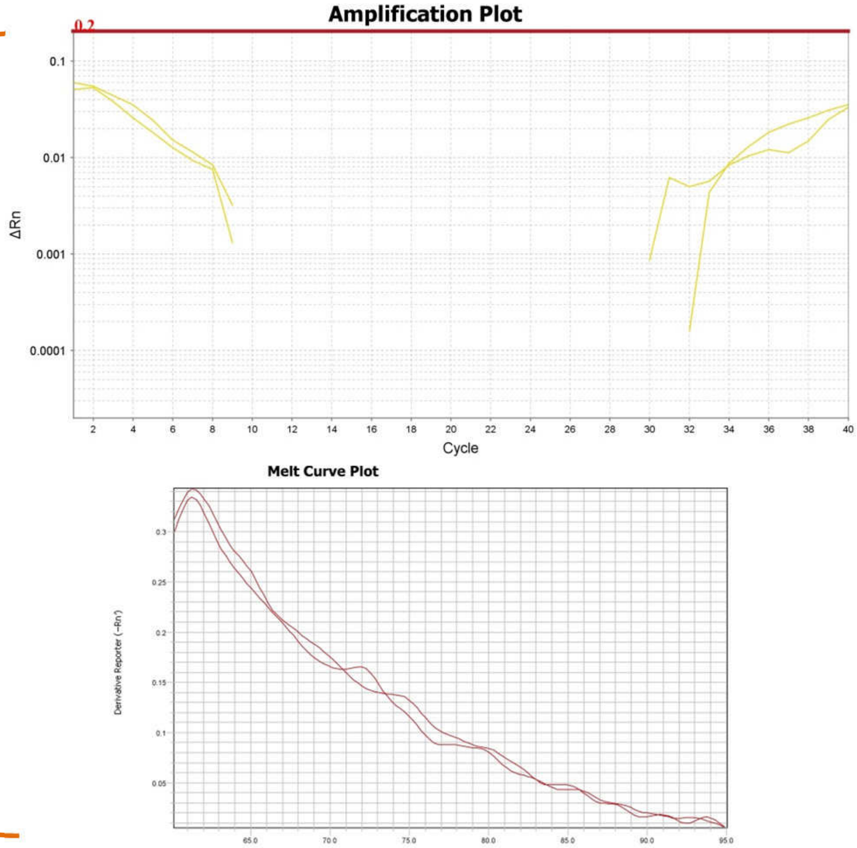

By divergent primers ◀▶ & with no template

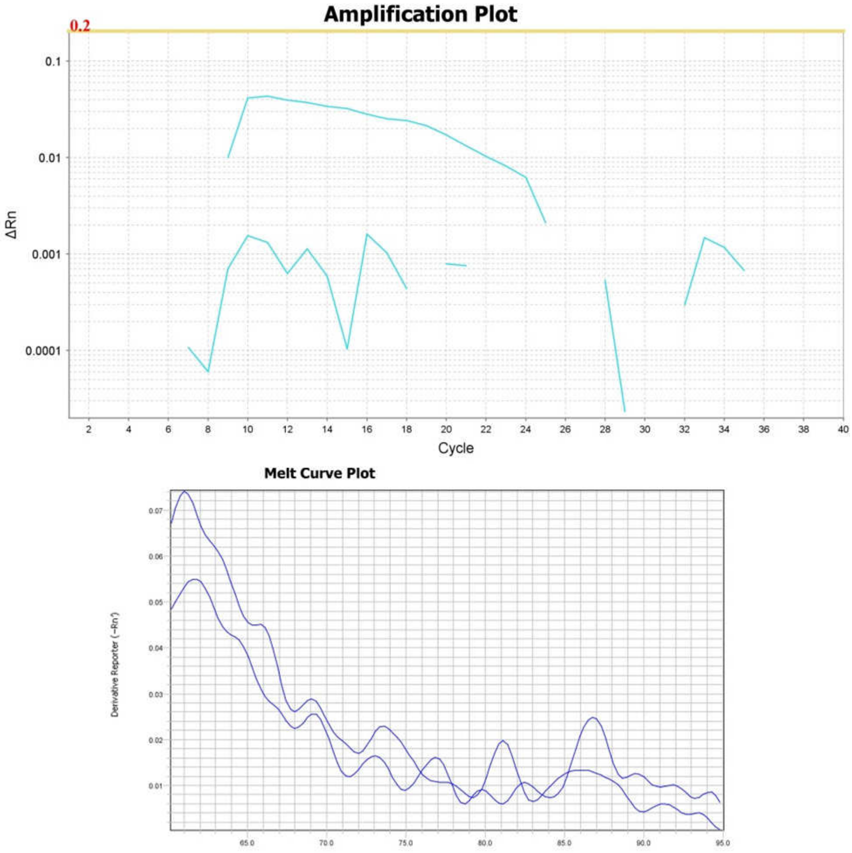

Formin-like protein 20_circular RNA1 (ID: Ch4:491530198-491530553)

<u>**T**</u>**AACTTAGAATAGCAGCTTTCCACCTAGATTCTGCATCAATAGGA**GTACTGTTAGGTTGGTTAG
CAAGCACATCATGACTCACGGGAAGCTGCCGTGAAGGATGTGGTGGAGACTGTGGGGTGG
ACGAGAGCACAGCAGTTTCAGAGTCACCTTGTAGCTTGTGGTCACCTATATGGTCCTGACTTAG
GTATTTGTTCTTTAGGTGTTAGATCTTCGTTTCCATCCATGGTCAGTGATCCATTAGTGGTTGGTA
GTCTGCATGATCCTAGCTAGTAGGAAACTGCATCAGGCAGGGGTAGGGTGGTAAGTTC**TGTTCT**
ACTGTTAGATGACTGGAGTTTACACTCCGGCAT<u>**C**</u>

Formin-like protein 20_circular RNA2 (ID: Ch4:4:491530332-491530553)

<u>**A**</u>**CAGCAGTTTCAGAGTCACCTTGTAGCTTGTGGTCACCTATATGGTCCTGACTTAGG**TATTTGT
TCTTTAGGTGTTAGATCTTCGTTTCCATCCATGGTCAGTGATCCATTAGTGGTTGGTAGTCTGCA
TGATCCTAGCTAGTAGGAAACTGCATCAGGCAGGGGTAGGGTGGTAAGTTCT**GTTCTACTGTTA**
GATGACTGGAGTTTACACTCCGGCATC<u>**T**</u>

The nucleotides of junction-region are underlined. The nucleotides of junction-region which are supported by the junction-spaning sequencing reads are shown in red. Introns are not shown if the absence is supported by sequencing reads. In the absence of supporting sequencing reads, the intronic nucletides are shown as N.

Structural relationship between the circular RNA and its parental gene

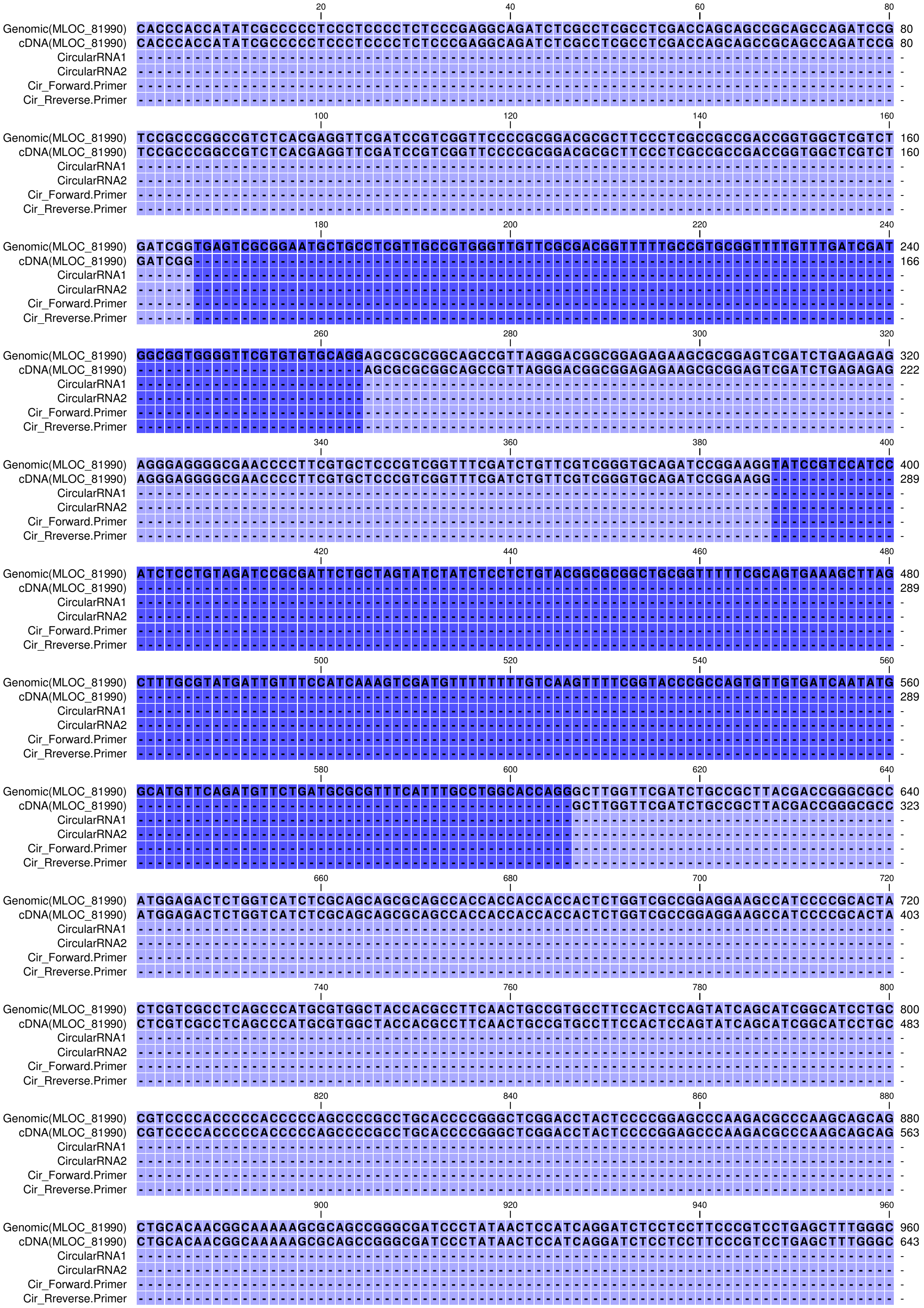

Genomic(MLOC_81990) CACCCACCATATCGCCCCCTCCCTCCCCTCTCCCGAGGCAGATCTCGCCTCGCCTCGACCAGCAGCCGCAGCCAGATCCG 80
cDNA(MLOC_81990) CACCCACCATATCGCCCCCTCCCTCCCCTCTCCCGAGGCAGATCTCGCCTCGCCTCGACCAGCAGCCGCAGCCAGATCCG 80
CircularRNA1
CircularRNA2
Cir_Forward.Primer
Cir_Rreverse.Primer
Genomic(MLOC_81990) TCCGCCCGGCCGTCTCACGAGGTTCGATCCGTCGGTTCCCCGCGGACGCGCTTCCCTCGCCGCCGACCGGTGGCTCGTCT 160
cDNA(MLOC_81990) TCCGCCCGGCCGTCTCACGAGGTTCGATCCGTCGGTTCCCCGCGGACGCGCTTCCCTCGCCGCCGACCGGTGGCTCGTCT 160
Genomic(MLOC_81990) GATCGGTGAGTCGCGGAATGCTGCCTCGTTGCCGTGGGTTGTTCGCGACGGTTTTTGCCGTGCGGTTTTGTTTGATCGAT 240
cDNA(MLOC_81990) GATCGG 166
Genomic(MLOC_81990) GGCGGTGGGGTTCGTGTGTGCAGGAGCGCGCGGCAGCCGTTAGGGACGGCGGAGAGAAGCGCGGAGTCGATCTGAGAGAG 320
cDNA(MLOC_81990) AGCGCGCGGCAGCCGTTAGGGACGGCGGAGAGAAGCGCGGAGTCGATCTGAGAGAG 222
Genomic(MLOC_81990) AGGGAGGGGCGAACCCCTTCGTGCTCCCGTCGGTTTCGATCTGTTCGTCGGGTGCAGATCCGGAAGGTATCCGTCCATCC 400
cDNA(MLOC_81990) AGGGAGGGGCGAACCCCTTCGTGCTCCCGTCGGTTTCGATCTGTTCGTCGGGTGCAGATCCGGAAGG 289
Genomic(MLOC_81990) ATCTCCTGTAGATCCGCGATTCTGCTAGTATCTATCTCCTCTGTACGGCGCGGCTGCGGTTTTTCGCAGTGAAAGCTTAG 480
cDNA(MLOC_81990) 289
Genomic(MLOC_81990) CTTTGCGTATGATTGTTTCCATCAAAGTCGATGTTTTTTTTGTCAAGTTTTCGGTACCCGCCAGTGTTGTGATCAATATG 560
cDNA(MLOC_81990) 289
Genomic(MLOC_81990) GCATGTTCAGATGTTCTGATGCGCGTTTCATTTGCCTGGCACCAGGGCTTGGTTCGATCTGCCGCTTACGACCGGGCGCC 640
cDNA(MLOC_81990) GCTTGGTTCGATCTGCCGCTTACGACCGGGCGCC 323
Genomic(MLOC_81990) ATGGAGACTCTGGTCATCTCGCAGCAGCGCAGCCACCACCACCACCACTCTGGTCGCCGGAGGAAGCCATCCCCGCACTA 720
cDNA(MLOC_81990) ATGGAGACTCTGGTCATCTCGCAGCAGCGCAGCCACCACCACCACCACTCTGGTCGCCGGAGGAAGCCATCCCCGCACTA 403
Genomic(MLOC_81990) CTCGTCGCCTCAGCCCATGCGTGGCTACCACGCCTTCAACTGCCGTGCCTTCCACTCCAGTATCAGCATCGGCATCCTGC 800
cDNA(MLOC_81990) CTCGTCGCCTCAGCCCATGCGTGGCTACCACGCCTTCAACTGCCGTGCCTTCCACTCCAGTATCAGCATCGGCATCCTGC 483
Genomic(MLOC_81990) CGTCCCCACCCCCACCCCCAGCCCCGCCTGCACCCCGGGCTCGGACCTACTCCCCGGAGCCCAAGACGCCCAAGCAGCAG 880
cDNA(MLOC_81990) CGTCCCCACCCCCACCCCCAGCCCCGCCTGCACCCCGGGCTCGGACCTACTCCCCGGAGCCCAAGACGCCCAAGCAGCAG 563
Genomic(MLOC_81990) CTGCACAACGGCAAAAAGCGCAGCCGGGCGATCCCTATAACTCCATCAGGATCTCCTCCTTCCCGTCCTGAGCTTTGGGC 960
cDNA(MLOC_81990) CTGCACAACGGCAAAAAGCGCAGCCGGGCGATCCCTATAACTCCATCAGGATCTCCTCCTTCCCGTCCTGAGCTTTGGGC 643

```
                                                                 980                 1.000               1.020               1.040
Genomic(MLOC_81990)  TGGCCCGGCATACTCCAACTCGCCGCCACCCAGCTCACTGCCGATTCCCAAGTTCTCGCTCCACCAGAAGCGCAGTGTAT 1040
cDNA(MLOC_81990)     TGGCCCGGCATACTCCAACTCGCCGCCACCCAGCTCACTGCCGATTCCCAAGTTCTCGCTCCACCAGAAGCGCAGTGTAT 723
CircularRNA1         -------------------------------------------------------------------------------- -
CircularRNA2         -------------------------------------------------------------------------------- -
Cir_Forward.Primer   -------------------------------------------------------------------------------- -
Cir_Rreverse.Primer  -------------------------------------------------------------------------------- -

                                                                 1.060               1.080               1.100               1.120
Genomic(MLOC_81990)  CACTTGAGTTGCCACCTGCTGACAGGACAGAGCATGTGGAGGTGCTTGTGCATGCCAAGTCAGCACCTTCGACACCCACT 1120
cDNA(MLOC_81990)     CACTTGAGTTGCCACCTGCTGACAGGACAGAGCATGTGGAGGTGCTTGTGCATGCCAAGTCAGCACCTTCGACACCCACT 803
CircularRNA1         -------------------------------------------------------------------------------- -
CircularRNA2         -------------------------------------------------------------------------------- -
Cir_Forward.Primer   -------------------------------------------------------------------------------- -
Cir_Rreverse.Primer  -------------------------------------------------------------------------------- -

                                                                 1.140               1.160               1.180               1.200
Genomic(MLOC_81990)  GCTGGCTCAGGATTTGGCTTCTTTGGTGAGAATGATACTGCCATTGCCACGGAGAATCTGAGGAGGATCCTTAATTTGGA 1200
cDNA(MLOC_81990)     GCTGGCTCAGGATTTGGCTTCTTTGGTGAGAATGATACTGCCATTGCCACGGAGAATCTGAGGAGGATCCTTAATTTGGA 883
CircularRNA1         -------------------------------------------------------------------------------- -
CircularRNA2         -------------------------------------------------------------------------------- -
Cir_Forward.Primer   -------------------------------------------------------------------------------- -
Cir_Rreverse.Primer  -------------------------------------------------------------------------------- -

                                                                 1.220               1.240               1.260               1.280
Genomic(MLOC_81990)  AATCACCGAGGACTGAATGATCGGGGCATCACCGTGTACATAGGTCATCAGCAACTAACCTTGGGTTCTTTGTATAAAGT 1280
cDNA(MLOC_81990)     AATCACCGAGGACTGAATGATCGGGGCATCACCGTGTACATAGGTCATCAGCAACTAACCTTGGGTTCTTTGTATAAAGT 963
CircularRNA1         -------------------------------------------------------------------------------T 1
CircularRNA2         -------------------------------------------------------------------------------- -
Cir_Forward.Primer   -------------------------------------------------------------------------------- -
Cir_Rreverse.Primer  -------------------------------------------------------------------------------- -

                                                                 1.300               1.320               1.340               1.360
Genomic(MLOC_81990)  AACTTAGAATAGCAGCTTTCCACCTAGATTCTGCATCAATAGGAGTACTGTTAGGTTGGTTAGCAAGCACATCATGACTC 1360
cDNA(MLOC_81990)     AACTTAGAATAGCAGCTTTCCACCTAGATTCTGCATCAATAGGAGTACTGTTAGGTTGGTTAGCAAGCACATCATGACTC 1043
CircularRNA1         AACTTAGAATAGCAGCTTTCCACCTAGATTCTGCATCAATAGGAGTACTGTTAGGTTGGTTAGCAAGCACATCATGACTC 81
CircularRNA2         -------------------------------------------------------------------------------- -
Cir_Forward.Primer   -------------------------------------------------------------------------------- -
Cir_Rreverse.Primer  -----AGAATAGCAGCTTTCCACCTAGATTC------------------------------------------------- 26

                                                                 1.380               1.400               1.420               1.440
Genomic(MLOC_81990)  ACGGGAAGCTGCCGTGAAGGATGTGGTGGTGGAGACTGTGGGGTGGACGAGAGCACAGCAGTTTCAGAGTCACCTTGTAG 1440
cDNA(MLOC_81990)     ACGGGAAGCTGCCGTGAAGGATGTGGTGGTGGAGACTGTGGGGTGGACGAGAGCACAGCAGTTTCAGAGTCACCTTGTAG 1123
CircularRNA1         ACGGGAAGCTGCCGTGAAGGATGTGGTGGTGGAGACTGTGGGGTGGACGAGAGCACAGCAGTTTCAGAGTCACCTTGTAG 161
CircularRNA2         ------------------------------------------------------ACAGCAGTTTCAGAGTCACCTTGTAG 26
Cir_Forward.Primer   -------------------------------------------------------------------------------- -
Cir_Rreverse.Primer  -------------------------------------------------------------------------------- 26

                                                                 1.460               1.480               1.500               1.520
Genomic(MLOC_81990)  CTTGTGGTCACCTATATGGTCCTGACTTAGGTATTTGTTCTTTAGGTGTTAGATCTTCGTTTCCATCCATGGTCAGTGAT 1520
cDNA(MLOC_81990)     CTTGTGGTCACCTATATGGTCCTGACTTAGGTATTTGTTCTTTAGGTGTTAGATCTTCGTTTCCATCCATGGTCAGTGAT 1203
CircularRNA1         CTTGTGGTCACCTATATGGTCCTGACTTAGGTATTTGTTCTTTAGGTGTTAGATCTTCGTTTCCATCCATGGTCAGTGAT 241
CircularRNA2         CTTGTGGTCACCTATATGGTCCTGACTTAGGTATTTGTTCTTTAGGTGTTAGATCTTCGTTTCCATCCATGGTCAGTGAT 106
Cir_Forward.Primer   ---------ACCTATATGGTCCTGACTTAGGTATTTGT------------------------------------------ 29
Cir_Rreverse.Primer  -------------------------------------------------------------------------------- 26

                                                                 1.540               1.560               1.580               1.600
Genomic(MLOC_81990)  CCATTAGTGGTTGGTAGTCTGCATGATCCTAGCTAGTAGGAAACTGCATCAGGCAGGGGTAGGGTGGTAAGTTCTGTTCT 1600
cDNA(MLOC_81990)     CCATTAGTGGTTGGTAGTCTGCATGATCCTAGCTAGTAGGAAACTGCATCAGGCAGGGGTAGGGTGGTAAGTTCTGTTCT 1283
CircularRNA1         CCATTAGTGGTTGGTAGTCTGCATGATCCTAGCTAGTAGGAAACTGCATCAGGCAGGGGTAGGGTGGTAAGTTCTGTTCT 321
CircularRNA2         CCATTAGTGGTTGGTAGTCTGCATGATCCTAGCTAGTAGGAAACTGCATCAGGCAGGGGTAGGGTGGTAAGTTCTGTTCT 186
Cir_Forward.Primer   -------------------------------------------------------------------------------- 29
Cir_Rreverse.Primer  -------------------------------------------------------------------------------- 26

                                                                 1.620               1.640               1.660               1.680
Genomic(MLOC_81990)  ACTGTTAGATGACTGGAGTTTACACTCCGGCATCTGTTCCCATGTTATATTCTGTAGGATATCTGCATCTTATATTCTTC 1680
cDNA(MLOC_81990)     ACTGTTAGATGACTGGAGTTTACACTCCGGCATCTGTTCCCATGTTATATTCTGTAGGATATCTGCATCTTATATTCTTC 1363
CircularRNA1         ACTGTTAGATGACTGGAGTTTACACTCCGGCATC---------------------------------------------- 355
CircularRNA2         ACTGTTAGATGACTGGAGTTTACACTCCGGCATCT--------------------------------------------- 221
Cir_Forward.Primer   -------------------------------------------------------------------------------- 29
Cir_Rreverse.Primer  -------------------------------------------------------------------------------- 26

                                                                 1.700               1.720               1.740               1.760
Genomic(MLOC_81990)  CAATACATACAGTCTGCCTCACCATCTTGACACTCTTCTAATCACACCGACTTCCCTGGTGCTCTGCAAAGAACACAAGG 1760
cDNA(MLOC_81990)     CAATACATACAGTCTGCCTCACCATCTTGACACTCTTCTAATCACACCGACTTCCCTGGTGCTCTGCAAAGAACACAAGG 1443
CircularRNA1         -------------------------------------------------------------------------------- 355
CircularRNA2         -------------------------------------------------------------------------------- 221
Cir_Forward.Primer   -------------------------------------------------------------------------------- 29
Cir_Rreverse.Primer  -------------------------------------------------------------------------------- 26

                                                                 1.780               1.800               1.820               1.840
Genomic(MLOC_81990)  ATATCTCCTCACTGGCTCTTCTGTATTTTTTGGAATGAAGAGGCCAGTGTAGGTTCAAGAAGCCCCCCCTTCGACGCCCT 1840
cDNA(MLOC_81990)     ATATCTCCTCACTGGCTCTTCTGTATTTTTTGGAATGAAGAGGCCAGTGTAGGTTCAAGAAGCCCCCCCTTCGACGCCCT 1523
CircularRNA1         -------------------------------------------------------------------------------- 355
CircularRNA2         -------------------------------------------------------------------------------- 221
Cir_Forward.Primer   -------------------------------------------------------------------------------- 29
Cir_Rreverse.Primer  -------------------------------------------------------------------------------- 26

                                                                 1.860               1.880               1.900               1.920
Genomic(MLOC_81990)  TTCTCCCTCATGTGTCTACTAACTTTGATCTGTGCACCTCTCCCCCCACCATAGTTTGTTGCATATGTAGTCAAATCCCC 1920
cDNA(MLOC_81990)     TTCTCCCTCATGTGTCTACTAACTTTGATCTGTGCACCTCTCCCCCCACCATAGTTTGTTGCATATGTAGTCAAATCCCC 1603
CircularRNA1         -------------------------------------------------------------------------------- 355
CircularRNA2         -------------------------------------------------------------------------------- 221
Cir_Forward.Primer   -------------------------------------------------------------------------------- 29
Cir_Rreverse.Primer  -------------------------------------------------------------------------------- 26
```

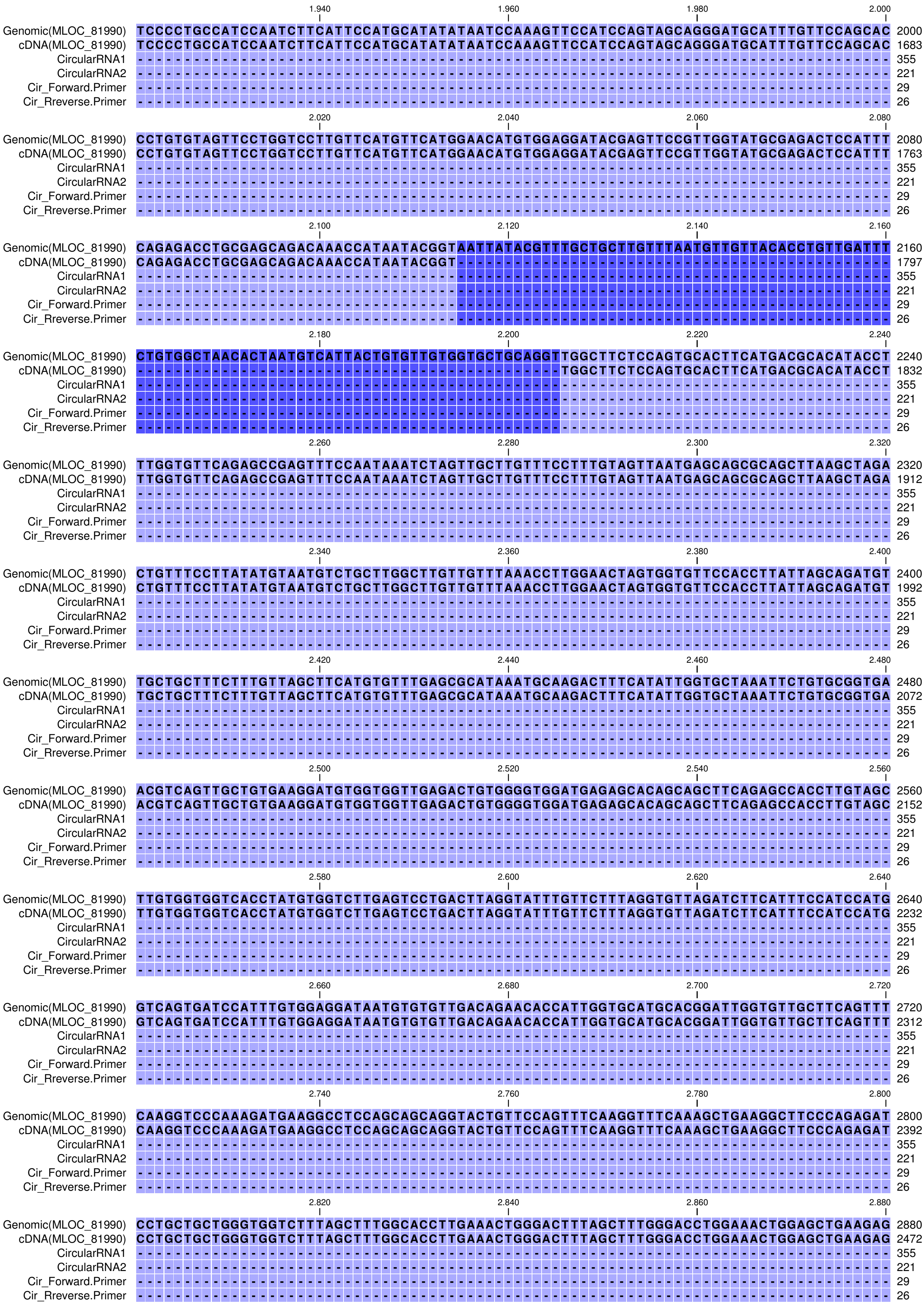
1.940 1.960 1.980 2.000
Genomic(MLOC_81990) TCCCCTGCCATCCAATCTTCATTCCATGCATATATAATCCAAAGTTCCATCCAGTAGCAGGGATGCATTTGTTCCAGCAC 2000
cDNA(MLOC_81990) TCCCCTGCCATCCAATCTTCATTCCATGCATATATAATCCAAAGTTCCATCCAGTAGCAGGGATGCATTTGTTCCAGCAC 1683
CircularRNA1 355
CircularRNA2 221
Cir_Forward.Primer 29
Cir_Rreverse.Primer 26
2.020 2.040 2.060 2.080
Genomic(MLOC_81990) CCTGTGTAGTTCCTGGTCCTTGTTCATGTTCATGGAACATGTGGAGGATACGAGTTCCGTTGGTATGCGAGACTCCATTT 2080
cDNA(MLOC_81990) CCTGTGTAGTTCCTGGTCCTTGTTCATGTTCATGGAACATGTGGAGGATACGAGTTCCGTTGGTATGCGAGACTCCATTT 1763
CircularRNA1 355
CircularRNA2 221
Cir_Forward.Primer 29
Cir_Rreverse.Primer 26
2.100 2.120 2.140 2.160
Genomic(MLOC_81990) CAGAGACCTGCGAGCAGACAAACCATAATACGGTAATTATACGTTTGCTGCTTGTTTAATGTTGTTACACCTGTTGATTT 2160
cDNA(MLOC_81990) CAGAGACCTGCGAGCAGACAAACCATAATACGGT 1797
CircularRNA1 355
CircularRNA2 221
Cir_Forward.Primer 29
Cir_Rreverse.Primer 26
2.180 2.200 2.220 2.240
Genomic(MLOC_81990) CTGTGGCTAACACTAATGTCATTACTGTGTTGTGGTGCTGCAGGTTGGCTTCTCCAGTGCACTTCATGACGCACATACCT 2240
cDNA(MLOC_81990) TGGCTTCTCCAGTGCACTTCATGACGCACATACCT 1832
CircularRNA1 355
CircularRNA2 221
Cir_Forward.Primer 29
Cir_Rreverse.Primer 26
2.260 2.280 2.300 2.320
Genomic(MLOC_81990) TTGGTGTTCAGAGCCGAGTTTCCAATAAATCTAGTTGCTTGTTTCCTTTGTAGTTAATGAGCAGCGCAGCTTAAGCTAGA 2320
cDNA(MLOC_81990) TTGGTGTTCAGAGCCGAGTTTCCAATAAATCTAGTTGCTTGTTTCCTTTGTAGTTAATGAGCAGCGCAGCTTAAGCTAGA 1912
CircularRNA1 355
CircularRNA2 221
Cir_Forward.Primer 29
Cir_Rreverse.Primer 26
2.340 2.360 2.380 2.400
Genomic(MLOC_81990) CTGTTTCCTTATATGTAATGTCTGCTTGGCTTGTTGTTTAAACCTTGGAACTAGTGGTGTTCCACCTTATTAGCAGATGT 2400
cDNA(MLOC_81990) CTGTTTCCTTATATGTAATGTCTGCTTGGCTTGTTGTTTAAACCTTGGAACTAGTGGTGTTCCACCTTATTAGCAGATGT 1992
CircularRNA1 355
CircularRNA2 221
Cir_Forward.Primer 29
Cir_Rreverse.Primer 26
2.420 2.440 2.460 2.480
Genomic(MLOC_81990) TGCTGCTTTCTTTGTTAGCTTCATGTGTTTGAGCGCATAAATGCAAGACTTTCATATTGGTGCTAAATTCTGTGCGGTGA 2480
cDNA(MLOC_81990) TGCTGCTTTCTTTGTTAGCTTCATGTGTTTGAGCGCATAAATGCAAGACTTTCATATTGGTGCTAAATTCTGTGCGGTGA 2072
CircularRNA1 355
CircularRNA2 221
Cir_Forward.Primer 29
Cir_Rreverse.Primer 26
2.500 2.520 2.540 2.560
Genomic(MLOC_81990) ACGTCAGTTGCTGTGAAGGATGTGGTGGTTGAGACTGTGGGGTGGATGAGAGCACAGCAGCTTCAGAGCCACCTTGTAGC 2560
cDNA(MLOC_81990) ACGTCAGTTGCTGTGAAGGATGTGGTGGTTGAGACTGTGGGGTGGATGAGAGCACAGCAGCTTCAGAGCCACCTTGTAGC 2152
CircularRNA1 355
CircularRNA2 221
Cir_Forward.Primer 29
Cir_Rreverse.Primer 26
2.580 2.600 2.620 2.640
Genomic(MLOC_81990) TTGTGGTGGTCACCTATGTGGTCTTGAGTCCTGACTTAGGTATTTGTTCTTTAGGTGTTAGATCTTCATTTCCATCCATG 2640
cDNA(MLOC_81990) TTGTGGTGGTCACCTATGTGGTCTTGAGTCCTGACTTAGGTATTTGTTCTTTAGGTGTTAGATCTTCATTTCCATCCATG 2232
CircularRNA1 355
CircularRNA2 221
Cir_Forward.Primer 29
Cir_Rreverse.Primer 26
2.660 2.680 2.700 2.720
Genomic(MLOC_81990) GTCAGTGATCCATTTGTGGAGGATAATGTGTGTTGACAGAACACCATTGGTGCATGCACGGATTGGTGTTGCTTCAGTTT 2720
cDNA(MLOC_81990) GTCAGTGATCCATTTGTGGAGGATAATGTGTGTTGACAGAACACCATTGGTGCATGCACGGATTGGTGTTGCTTCAGTTT 2312
CircularRNA1 355
CircularRNA2 221
Cir_Forward.Primer 29
Cir_Rreverse.Primer 26
2.740 2.760 2.780 2.800
Genomic(MLOC_81990) CAAGGTCCCAAAGATGAAGGCCTCCAGCAGCAGGTACTGTTCCAGTTTCAAGGTTTCAAAGCTGAAGGCTTCCCAGAGAT 2800
cDNA(MLOC_81990) CAAGGTCCCAAAGATGAAGGCCTCCAGCAGCAGGTACTGTTCCAGTTTCAAGGTTTCAAAGCTGAAGGCTTCCCAGAGAT 2392
CircularRNA1 355
CircularRNA2 221
Cir_Forward.Primer 29
Cir_Rreverse.Primer 26
2.820 2.840 2.860 2.880
Genomic(MLOC_81990) CCTGCTGCTGGGTGGTCTTTAGCTTTGGCACCTTGAAACTGGGACTTTAGCTTTGGGACCTGGAAACTGGAGCTGAAGAG 2880
cDNA(MLOC_81990) CCTGCTGCTGGGTGGTCTTTAGCTTTGGCACCTTGAAACTGGGACTTTAGCTTTGGGACCTGGAAACTGGAGCTGAAGAG 2472
CircularRNA1 355
CircularRNA2 221
Cir_Forward.Primer 29
Cir_Rreverse.Primer 26

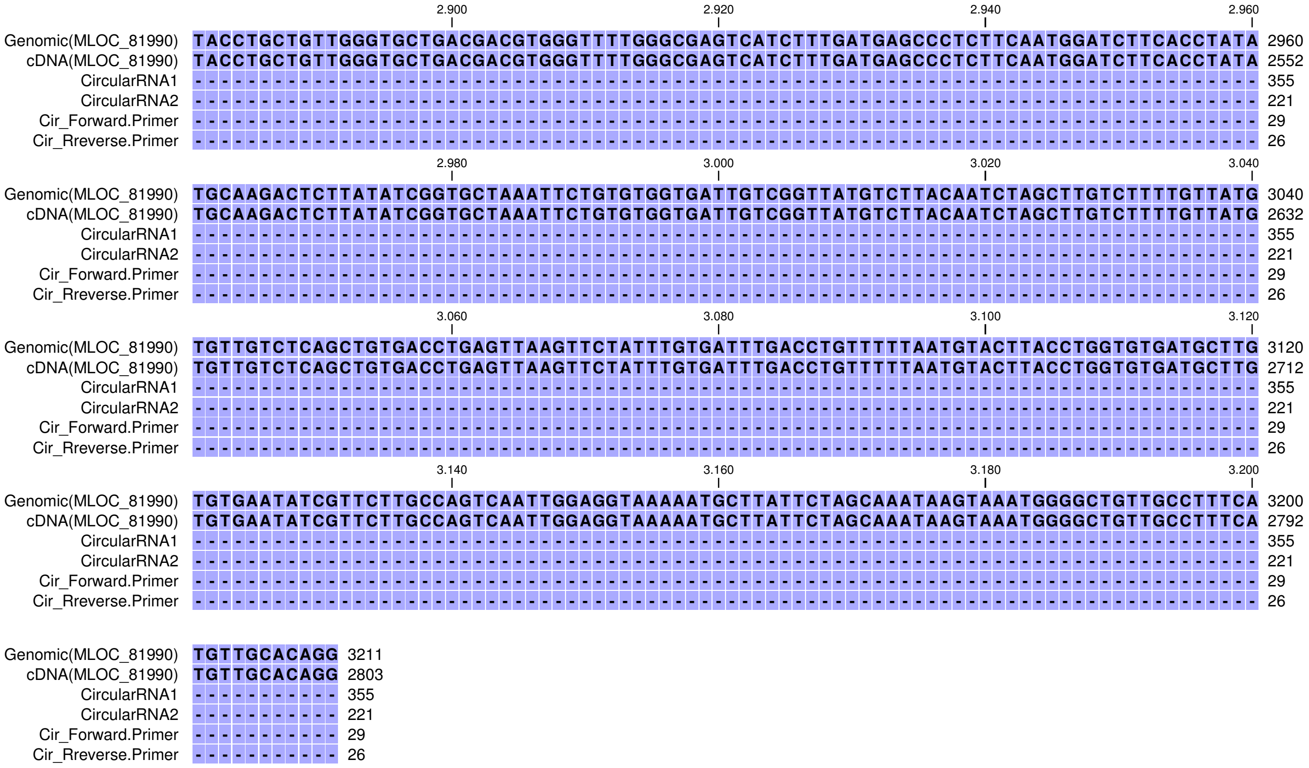

2.900 2.920 2.940 2.960
Genomic(MLOC_81990) TACCTGCTGTTGGGTGCTGACGACGTGGGTTTTGGGCGAGTCATCTTTGATGAGCCCTCTTCAATGGATCTTCACCTATA 2960
cDNA(MLOC_81990) TACCTGCTGTTGGGTGCTGACGACGTGGGTTTTGGGCGAGTCATCTTTGATGAGCCCTCTTCAATGGATCTTCACCTATA 2552
CircularRNA1 355
CircularRNA2 221
Cir_Forward.Primer 29
Cir_Rreverse.Primer 26
2.980 3.000 3.020 3.040
Genomic(MLOC_81990) TGCAAGACTCTTATATCGGTGCTAAATTCTGTGTGGTGATTGTCGGTTATGTCTTACAATCTAGCTTGTCTTTTGTTATG 3040
cDNA(MLOC_81990) TGCAAGACTCTTATATCGGTGCTAAATTCTGTGTGGTGATTGTCGGTTATGTCTTACAATCTAGCTTGTCTTTTGTTATG 2632
CircularRNA1 355
CircularRNA2 221
Cir_Forward.Primer 29
Cir_Rreverse.Primer 26
3.060 3.080 3.100 3.120
Genomic(MLOC_81990) TGTTGTCTCAGCTGTGACCTGAGTTAAGTTCTATTTGTGATTTGACCTGTTTTTAATGTACTTACCTGGTGTGATGCTTG 3120
cDNA(MLOC_81990) TGTTGTCTCAGCTGTGACCTGAGTTAAGTTCTATTTGTGATTTGACCTGTTTTTAATGTACTTACCTGGTGTGATGCTTG 2712
CircularRNA1 355
CircularRNA2 221
Cir_Forward.Primer 29
Cir_Rreverse.Primer 26
3.140 3.160 3.180 3.200
Genomic(MLOC_81990) TGTGAATATCGTTCTTGCCAGTCAATTGGAGGTAAAAATGCTTATTCTAGCAAATAAGTAAATGGGGCTGTTGCCTTTCA 3200
cDNA(MLOC_81990) TGTGAATATCGTTCTTGCCAGTCAATTGGAGGTAAAAATGCTTATTCTAGCAAATAAGTAAATGGGGCTGTTGCCTTTCA 2792
CircularRNA1 355
CircularRNA2 221
Cir_Forward.Primer 29
Cir_Rreverse.Primer 26
Genomic(MLOC_81990) TGTTGCACAGG 3211
cDNA(MLOC_81990) TGTTGCACAGG 2803
CircularRNA1 355
CircularRNA2 221
Cir_Forward.Primer 29
Cir_Rreverse.Primer 26

Real-Time PCR for the junction region of formin-like protein 20_circular RNA1 (ID: Ch4:491530198-491530553)

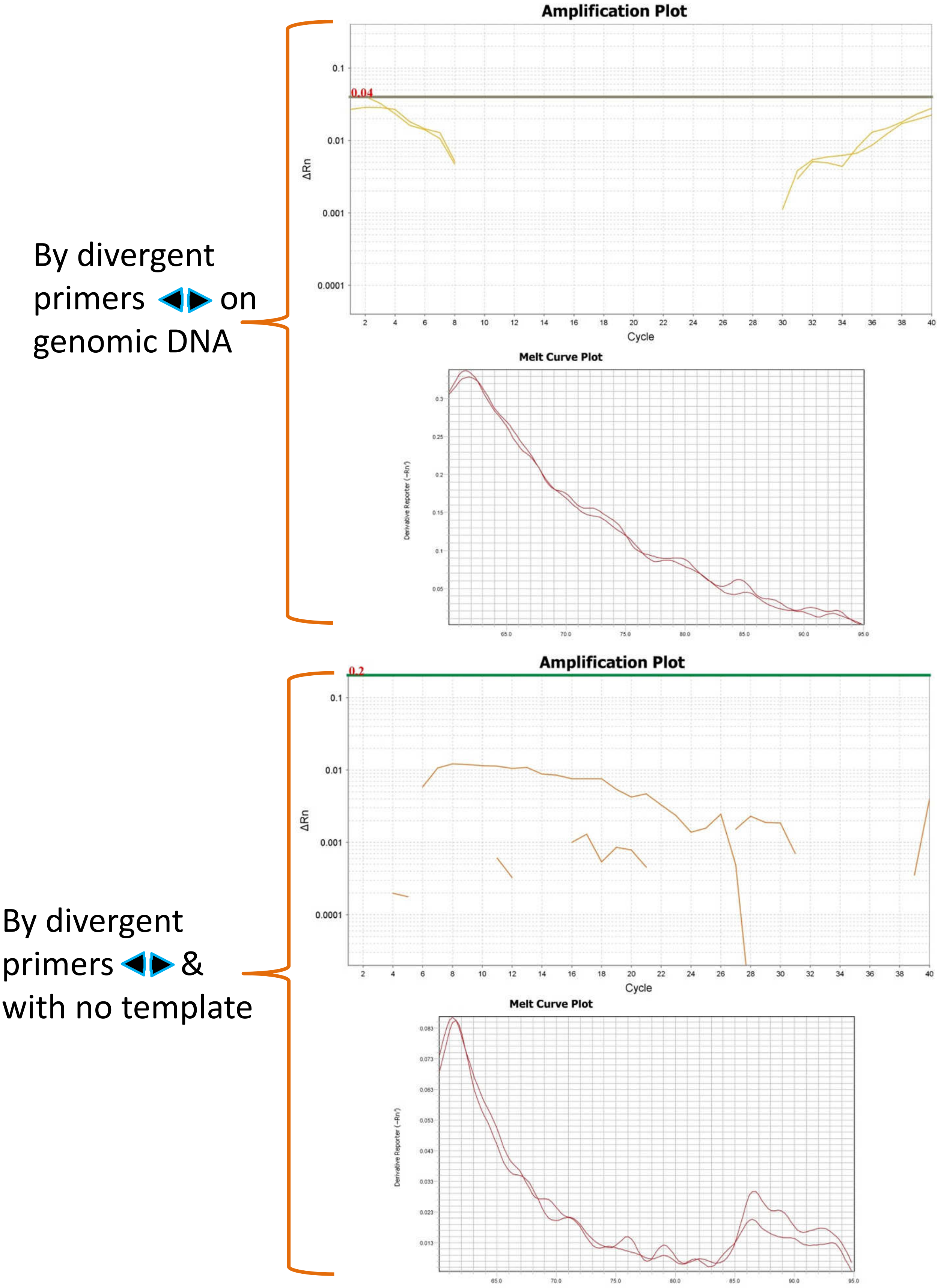

Alpha-mannosidase 1_circular RNA (ID: Ch2:566491430-566492027**)**

GTGAATAGTGGTGAAAAGGACGACAAGATGCAGAGCTTCTTCCTAGCAGAGACGCTCAAGTAC
CTCTATCTGTTGTTCTCCCCTCCGTCAGTCGTATCTTTCGATGAGTGGGTCTTCAACACTGAAGC
TCACCCTTTGAGAATCGTTCCGACACATGGTAGTAACGGTCAGTCAATTGAAACTGCAACGCCA
GTGGTCCGACCATTCGGTAGGAAGCAAGGGAAACAGGGGTAGGCTTGAACAACAGATCAGAGG
ATTTTTGCATCCGCCGTGTGATTGTGACCTGCCTTGTTGAGAATGTTATGTTACGATGGGAATTT
CTTCCTGCAGCGGCAAGGTTGCAAATCACGGTACTAGGCTATTTAGGCAACACAATTGTAGGTA
GAGCATGATGGATAATCTGGGGCATGCTGAACCTTGAGAGTATAACCGAAGATCAGCAGCCCCA
TGAAAGTTTTCACCTTCGAACTTGTCTATGCTCCGTGCATCATTGATTTGCAACACATATGTAACT
TAGTGTCCATATGTTGTTGCTTTCCTGGGGCTCTTCTGGAGT**TGTATCCTTATTGTTTTGATTATA**
TGATATATAAAAAGGGTT

The nucleotides of junction-region are underlined. The nucleotides of junction-region which are supported by the junction-spaning sequencing reads are shown in red. Introns are not shown if the absence is supported by sequencing reads. In the absence of supporting sequencing reads, the intronic nucletides are shown as N.

Structural relationship between the circular RNA and its parental gene

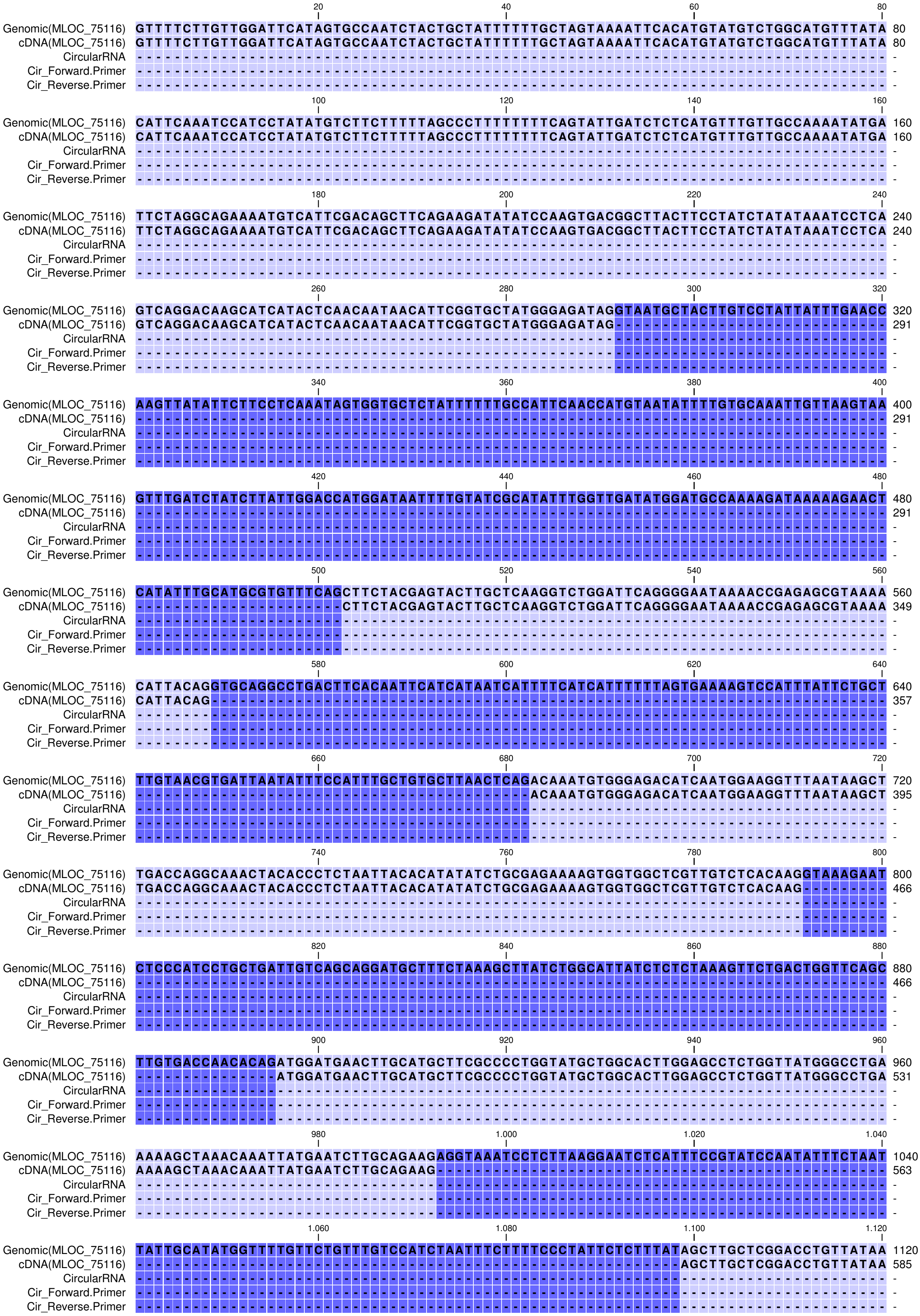
Genomic(MLOC_75116)
cDNA(MLOC_75116)
CircularRNA
Cir_Forward.Primer
Cir_Reverse.Primer

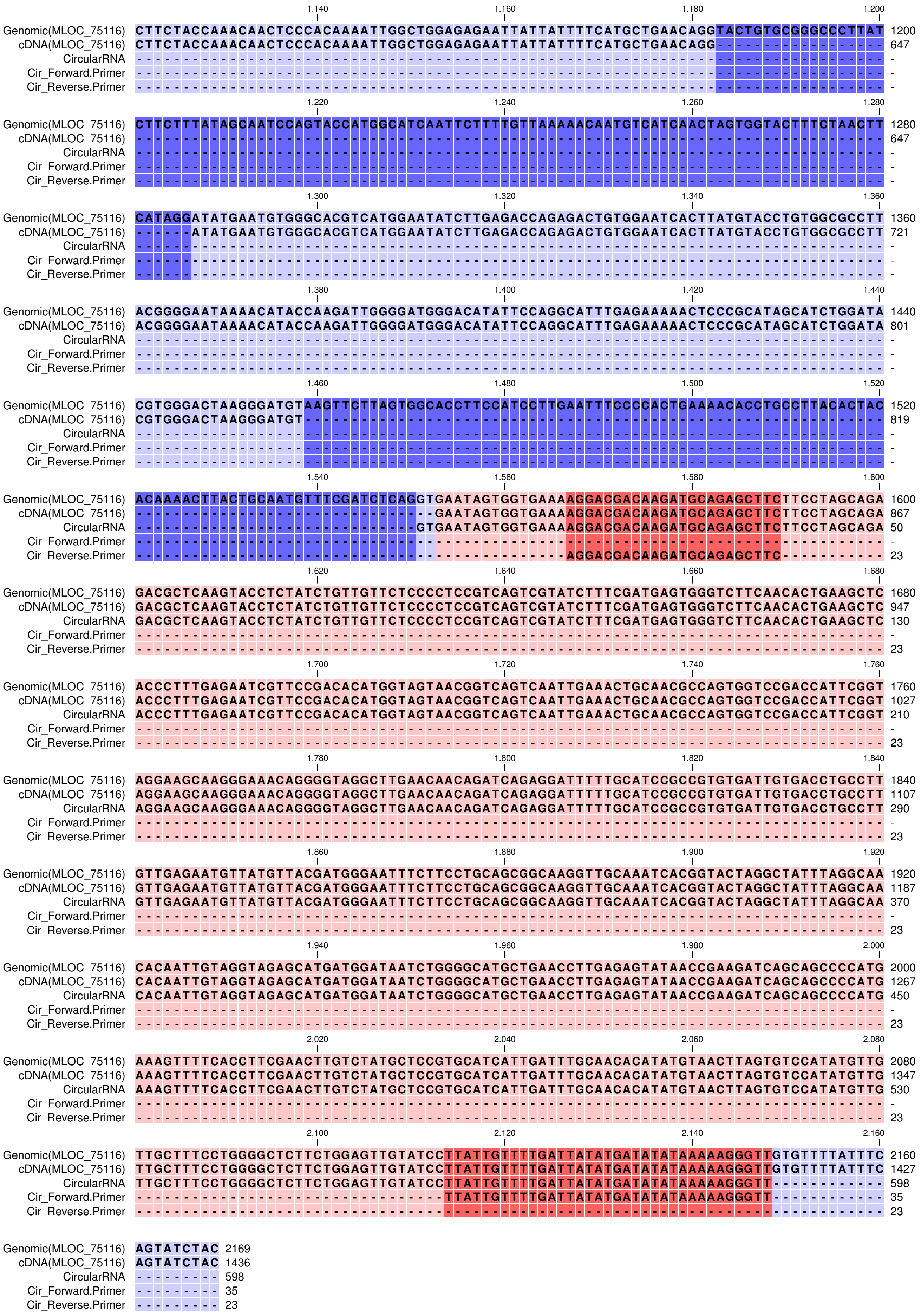
Genomic(MLOC_75116)
cDNA(MLOC_75116)
CircularRNA
Cir_Forward.Primer
Cir_Reverse.Primer

Real-Time PCR for the junction region of alpha-mannosidase 1_circular RNA (ID: Ch2:566491430-566492027)

By divergent primers ◀▶ on genomic DNA

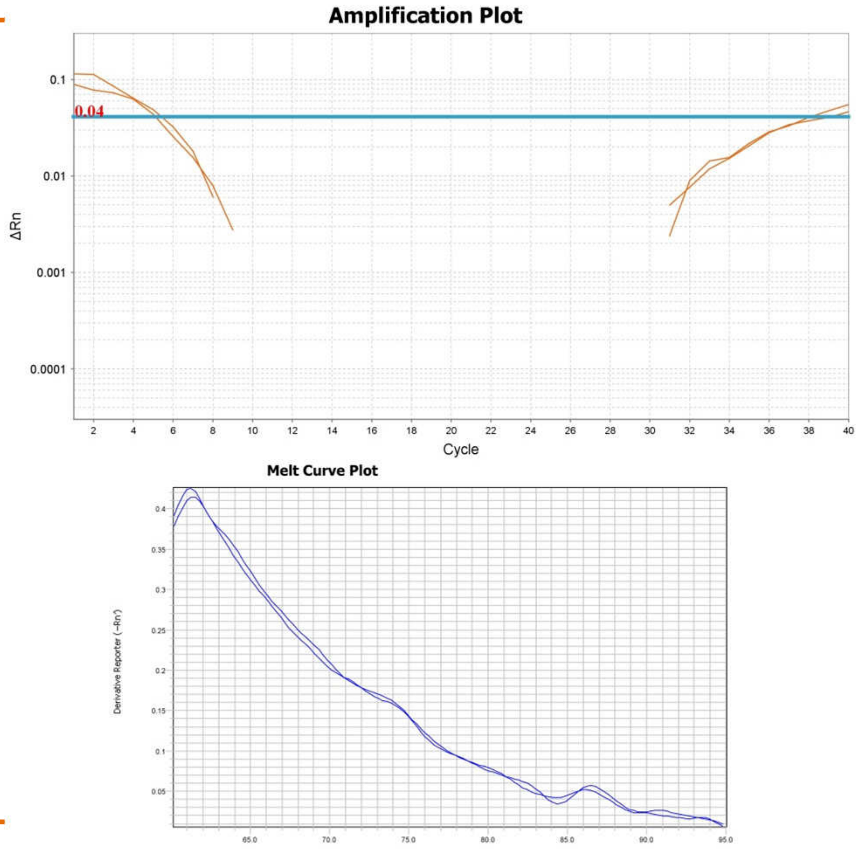

By divergent primers ◀▶ & with no template

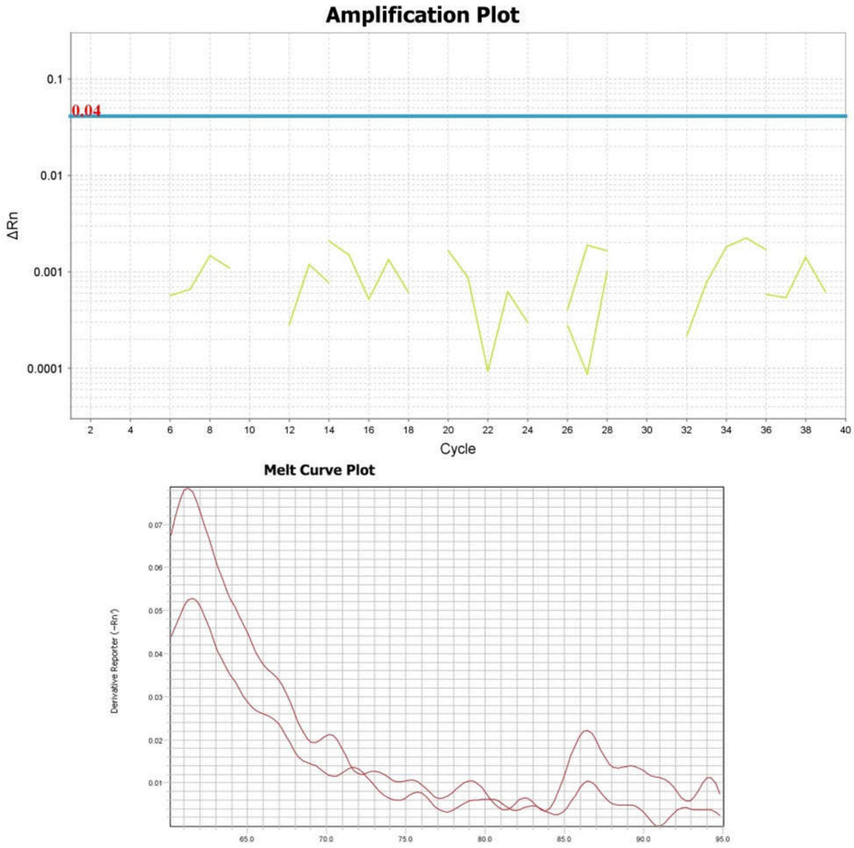

Chromosome segregation protein_circular RNA (ID: Ch5:361207403-361207861)

GAGTGGGTTCATCTAGGCATGTTGCGGGCAAATCCTGCATGTTCGAATCTTACAATAAGCATCC
TCCTTCTCACACGGGCACTATACAAACTGTTGATGGCACAAAACAGCATGTCATTGGAGTTGGC
ACGGTAAAGCGCACTCCAACCATCTTCCTATTCTCAGTTTTACATGTGCCAGCTTTTCCCGTCAA
TTTAGTCTCTTTCTGTGCTCTCATTGATCAAATAGACTGTCGTGTGATCCTTGACAAATTCGGTCA
CGAGATTCAGGAGCGACAAACGAGGCAGGCGGTTGGGACTGGCACCAGACGTAGGGGGCTCT
CGTATATGGACCAGGGGGTGCAGCCAGACTTGATTTGTGCTGCGACCATGGAGGACAAGGAGA
AGCAGGCGATGATCCATCACTGTAGGAGGGGACATGTC**TCGTTTGATAAGATAAGTAGAATATT**
TCCGGATGTT

The nucleotides of junction-region are underlined. The nucleotides of junction-region which are supported by the junction-spaning sequencing reads are shown in red. Introns are not shown if the absence is supported by sequencing reads. In the absence of supporting sequencing reads, the intronic nucletides are shown as N.

Structural relationship between the circular RNA and its parental gene

```
                    20        40        60        80
Genomic             CCATCGTATCAAAGCAAATGGACGAAGTGGCACCAAGTCTCCGGAGTCCTATGTGACAAGAGTGCCACAAAACCTAAAAG 80
cDNA(predicted)     -------------------------------------------------------------------------------- -
CircularRNA         -------------------------------------------------------------------------------- -
Cir_Forward.Primer  -------------------------------------------------------------------------------- -
Cir_Reverse.Primer  -------------------------------------------------------------------------------- -

                    100       120       140       160
Genomic             GTTCTATAGGACGACGATTCAACATACGATGTTGTATGTCGCAAAATGTTGGCCAACTAAAGGCACCATATCCAACAAT 160
cDNA(predicted)     -------------------------------------------------------------------------------- -
CircularRNA         -------------------------------------------------------------------------------- -
Cir_Forward.Primer  -------------------------------------------------------------------------------- -
Cir_Reverse.Primer  -------------------------------------------------------------------------------- -

                    180       200       220       240
Genomic             TAGGTGAAGAGGGATGTGCATGCTAAAATGGATATGTGGCCACACAAGAAAGGATCAGAGCAAATGATGATAGACGTGAT 240
cDNA(predicted)     -------------------------------------------------------------------------------- -
CircularRNA         -------------------------------------------------------------------------------- -
Cir_Forward.Primer  -------------------------------------------------------------------------------- -
Cir_Reverse.Primer  -------------------------------------------------------------------------------- -

                    260       280       300       320
Genomic             AGAGTTGAGGTACCACCAATTGAAGAGAAGCTTGTCCAACATCGTCTGAGATGGTTTAGGCATATACAACGCGGGCATCC 320
cDNA(predicted)     -------------------------------------------------------------------------------- -
CircularRNA         -------------------------------------------------------------------------------- -
Cir_Forward.Primer  -------------------------------------------------------------------------------- -
Cir_Reverse.Primer  -------------------------------------------------------------------------------- -

                    340       360       380       400
Genomic             AGAGGAGTTGGTGCATAGCGGAAGATTAAAGTGTGTTGATAATGTCAAGACATGTCGGGATAGATCAAACTCGGCATGGG 400
cDNA(predicted)     -------------------------------------------------------------------------------- -
CircularRNA         -------------------------------------------------------------------------------- -
Cir_Forward.Primer  -------------------------------------------------------------------------------- -
Cir_Reverse.Primer  -------------------------------------------------------------------------------- -

                    420       440       460       480
Genomic             AGGAATCCGCAAAGAGACCTAAAGGATTGGAATATATCATCAAAGAACTAGTCATAAACAAGGATGTGTGGAAGTTAGCT 480
cDNA(predicted)     -------------------------------------------------------------------------------- -
CircularRNA         -------------------------------------------------------------------------------- -
Cir_Forward.Primer  -------------------------------------------------------------------------------- -
Cir_Reverse.Primer  -------------------------------------------------------------------------------- -

                    500       520       540       560
Genomic             ACCCATGTGCCAGAACCATGACTAGGTTACGAGATCTTATAACCATTACGAGATCTTATGGGTTTCAGCTCTAGCCTGCC 560
cDNA(predicted)     -------------------------------------------------------------------------------- -
CircularRNA         -------------------------------------------------------------------------------- -
Cir_Forward.Primer  -------------------------------------------------------------------------------- -
Cir_Reverse.Primer  -------------------------------------------------------------------------------- -

                    580       600       620       640
Genomic             CCTATTTGTTTGAGACTGAAAAGCCTTGTTGTCGGAGTACCCATGGAGATGCGCTGAAAGTGGCCATACTTTACATGACC 640
cDNA(predicted)     -------------------------------------------------------------------------------- -
CircularRNA         -------------------------------------------------------------------------------- -
Cir_Forward.Primer  -------------------------------------------------------------------------------- -
Cir_Reverse.Primer  -------------------------------------------------------------------------------- -

                    660       680       700       720
Genomic             AGTATAGCCGGCTCCTAACAGCAAGCTGATAGCCACCTAATTCCTGATAACGATTCACTATTCTATAGATCAAATTATTG 720
cDNA(predicted)     -------------------------------------------------------------------------------- -
CircularRNA         -------------------------------------------------------------------------------- -
Cir_Forward.Primer  -------------------------------------------------------------------------------- -
Cir_Reverse.Primer  -------------------------------------------------------------------------------- -

                    740       760       780       800
Genomic             AGACATGGTACATGCCCAGGGACACCCACACTAAGTCAAAACGACTCGTGCTTATACAATGATATACCAAATTACCAACC 800
cDNA(predicted)     -------------------------------------------------------------------------------- -
CircularRNA         -------------------------------------------------------------------------------- -
Cir_Forward.Primer  -------------------------------------------------------------------------------- -
Cir_Reverse.Primer  -------------------------------------------------------------------------------- -

                    820       840       860       880
Genomic             CAGATGTCAAGCAGCAAACTGCTAAATTTTGCAGCAGTCTCTAAAATTATTGAGACATGGTACATGCCCAGGGACATCCG 880
cDNA(predicted)     -------------------------------------------------------------------------------- -
CircularRNA         -------------------------------------------------------------------------------- -
Cir_Forward.Primer  -------------------------------------------------------------------------------- -
Cir_Reverse.Primer  -------------------------------------------------------------------------------- -

                    900       920       940       960
Genomic             TTCTTTTTTGCTCGATCAAGTCTTTGCAGCATTAACCAGAGGGGATATACAAGTACTAGCGCTGAGATGCACTAACTTTA 960
cDNA(predicted)     -------------------------------------------------------------------------------- -
CircularRNA         -------------------------------------------------------------------------------- -
Cir_Forward.Primer  -------------------------------------------------------------------------------- -
Cir_Reverse.Primer  -------------------------------------------------------------------------------- -

                    980       1.000     1.020     1.040
Genomic             TGGATGCTTTTAGCCAATCCTAACTGATTCAATCTAGAGAACTGTTGAACCGGTATGGGCCCAGCCCATCCACTTATCTC 1040
cDNA(predicted)     -------------------------------------------------------------------------------- -
CircularRNA         -------------------------------------------------------------------------------- -
Cir_Forward.Primer  -------------------------------------------------------------------------------- -
Cir_Reverse.Primer  -------------------------------------------------------------------------------- -

                    1.060     1.080     1.100     1.120
Genomic             TTAGGGCCCAAGCCCATATAAAGGAACTGACCTAGAAGGAGGCTTCAAGCCATCCCGTTCCCAGCTCAGCCGCCACACAC 1120
cDNA(predicted)     -------------------------------------------------------------------------------- -
CircularRNA         -------------------------------------------------------------------------------- -
Cir_Forward.Primer  -------------------------------------------------------------------------------- -
Cir_Reverse.Primer  -------------------------------------------------------------------------------- -
```

```
                                           1.140               1.160               1.180               1.200
Genomic             ATACACACAGTCTGCAGGTACTCCCCTCTCACGATTCAACATGGTATCAGACGCCAGGAACCTGCTTGAGGGCTGCTGTA 1200
cDNA(predicted)     ----------------------------------------ATGGTATCAGACGCCAGGAACCTGCTTGAGGGCTGCTGTA 40
CircularRNA         -------------------------------------------------------------------------------- -
Cir_Forward.Primer  -------------------------------------------------------------------------------- -
Cir_Reverse.Primer  -------------------------------------------------------------------------------- -
                                           1.220               1.240               1.260               1.280
Genomic             CGGCTGCATCTGCTGGAGTTGTGGGCTGCTGCTGTGCGCTGGTGCTTGCTGTTTTTGCTGCTGTGCAGAATAAGTGTGAC 1280
cDNA(predicted)     CGGCTGCATCTGCTGGAGTTGTGGGCTGCTGCTGTGCGCTGGTGCTTGCTGTTTTTGCTGCTGTGCAGAATAAGTGTGAC 120
CircularRNA         -------------------------------------------------------------------------------- -
Cir_Forward.Primer  -------------------------------------------------------------------------------- -
Cir_Reverse.Primer  -------------------------------------------------------------------------------- -
                                           1.300               1.320               1.340               1.360
Genomic             TGGTACGGCTGGAGCTGCTGGCTGTGGACCTGTTGCTGAATGGGAAGGGAAGAGAAGAGCTGCTGCAGAGGAGGCTGTAG 1360
cDNA(predicted)     T------------------------------------------------------------------------------- 121
CircularRNA         -------------------------------------------------------------------------------- -
Cir_Forward.Primer  -------------------------------------------------------------------------------- -
Cir_Reverse.Primer  -------------------------------------------------------------------------------- -
                                           1.380               1.400               1.420               1.440
Genomic             CTGCCTGAAGCTGTGTTTGCTGCTGCTGTAGGTGGACGAGGTGGTGTGTGTTGGGAGCTGCTGCTGCTGTTTGTAGAGGA 1440
cDNA(predicted)     ------------------------------GGTGGACGAGGTGGTGTGTGTTGGGAGCTGCTGCTGCTGTTTGTAGAGGA 171
CircularRNA         -------------------------------------------------------------------------------- -
Cir_Forward.Primer  -------------------------------------------------------------------------------- -
Cir_Reverse.Primer  -------------------------------------------------------------------------------- -
                                           1.460               1.480               1.500               1.520
Genomic             GAAGGGCTTGAGGGGGAGAGCCTCACGCTCAAGGAGAGGCTCTTTCTGATGGAGCAGGAGGTCGGCAGGGAGTGCCGTCT 1520
cDNA(predicted)     GAAGGGCTTGAGGGGGAGAGCCTCACGCTCAAGGAGAGGCTCTTTCTGATGGAGCAGGAGGTCGGCAGGGAGTGCCGTCT 251
CircularRNA         -------------------------------------------------------------------------------- -
Cir_Forward.Primer  -------------------------------------------------------------------------------- -
Cir_Reverse.Primer  -------------------------------------------------------------------------------- -
                                           1.540               1.560               1.580               1.600
Genomic             TGTGACCATCGAGGCGCATTTTGCAGGGGCCAATGGTGCTGCTGGCAGCACCAAGAATGCTGTTGTTCTGAAGGAGATGG 1600
cDNA(predicted)     TGTGACCATCGAGGCGCATTTTGCAGGGGCCAATGGTGCTGCTGGCAGCACCAAGAATGCTGTTGTTCTGAAGGAGATGG 331
CircularRNA         -------------------------------------------------------------------------------- -
Cir_Forward.Primer  -------------------------------------------------------------------------------- -
Cir_Reverse.Primer  -------------------------------------------------------------------------------- -
                                           1.620               1.640               1.660               1.680
Genomic             GTGATACTATAGCTGCGGCTGGTGCTCACTATGCAGTCAAGGAGTCTCTAGGGAATGATGACATGAGAAGGGGTGATGTG 1680
cDNA(predicted)     GTGATACTATAGCTGCGGCTGGTGCTCACTATGCAGTCAAGGAGTCTCTAGGGAATGATGACATGAGAAGGGGTGATGTG 411
CircularRNA         -------------------------------------------------------------------------------- -
Cir_Forward.Primer  -------------------------------------------------------------------------------- -
Cir_Reverse.Primer  -------------------------------------------------------------------------------- -
                                           1.700               1.720               1.740               1.760
Genomic             GAGCCAGAGAAGAGTGGTGAGGACAGTGCAGAAAAGGACAGTGCAGGGTCAGAGAAGGAATACAACATGACTGTAGCAGA 1760
cDNA(predicted)     GAGCCAGAGAAGAGTGGTGAGGACAGTGCAGAAAAGGACAGTGCAGGGTCAGAGAAGGAATACAACATGACTGTAGCAGA 491
CircularRNA         -------------------------------------------------------------------------------- -
Cir_Forward.Primer  -------------------------------------------------------------------------------- -
Cir_Reverse.Primer  -------------------------------------------------------------------------------- -
                                           1.780               1.800               1.820               1.840
Genomic             GGTTGAGAGGCTCAGCGAGGAACTGGAAAGCCTCAAGAGGAAGGTGGGGGAAATCCAGGCTGGCAAGGAAACAGCCGAGG 1840
cDNA(predicted)     GGTTGAGAGGCTCAGCGAGGAACTGGAAAGCCTCAAGAGGAAGGTGGGGGAAATCCAGGCTGGCAAGGAAACAGCCGAGG 571
CircularRNA         -------------------------------------------------------------------------------- -
Cir_Forward.Primer  -------------------------------------------------------------------------------- -
Cir_Reverse.Primer  -------------------------------------------------------------------------------- -
                                           1.860               1.880               1.900               1.920
Genomic             GGGAGACACGTGAGAAGGATGCTCAGACCGTTAAGTTGAGGGCTGAGTTGGAGGAGCTTCATGCTTTCATATCAAAGCAC 1920
cDNA(predicted)     GGGAGACACGTGAGAAGGATGCTCAGACCGTTAAGTTGAGGGCTGAGTTGGAGGAGCTTCATGCTTTCATATCAAAGCAC 651
CircularRNA         -------------------------------------------------------------------------------- -
Cir_Forward.Primer  -------------------------------------------------------------------------------- -
Cir_Reverse.Primer  -------------------------------------------------------------------------------- -
                                           1.940               1.960               1.980               2.000
Genomic             CAAACTGCAGGCTTGAATCGCTATGGAGATTGTGCTCACTGGACCTCCACTAATGAAGATAGTCCGGAAAAGGCATCTCT 2000
cDNA(predicted)     CAAACTGCAGGCTTGAATCGCTATGGAGATTGTGCTCACTGGACCTCCACTAATGAAGATAGTCCGGAAAAGGCATCTCT 731
CircularRNA         -------------------------------------------------------------------------------- -
Cir_Forward.Primer  -------------------------------------------------------------------------------- -
Cir_Reverse.Primer  -------------------------------------------------------------------------------- -
                                           2.020               2.040               2.060               2.080
Genomic             TGCTGCTAATGAGAATGGTGTAGAGTGGGTTCATCTAGGCATGTTGCGGGCAAATCCTGCATGTTCGAATCTTACAATAA 2080
cDNA(predicted)     TGCTGCTAATGAGAATGGTGTAGAGTGGGTTCATCTAGGCATGTTGCGGGCAAATCCTGCATGTTCGAATCTTACAATAA 811
CircularRNA         ----------------------GAGTGGGTTCATCTAGGCATGTTGCGGGCAAATCCTGCATGTTCGAATCTTACAATAA 58
Cir_Forward.Primer  -------------------------------------------------------------------------------- -
Cir_Reverse.Primer  ------------------------------TCATCTAGGCATGTTGCGGG------------------------------ 20
                                           2.100               2.120               2.140               2.160
Genomic             GCATCCTCCTTCTCACACGGGCACTATACAAACTGTTGATGGCACAAAACAGCATGTCATTGGAGTTGGCACGGTAAAGC 2160
cDNA(predicted)     GCATCCTCCTTCTCACACGGGCACTATACAAACTGTTGATGGCACAAAACAGCATGTCATTGGAGTTGGCACGGTAA--- 888
CircularRNA         GCATCCTCCTTCTCACACGGGCACTATACAAACTGTTGATGGCACAAAACAGCATGTCATTGGAGTTGGCACGGTAAAGC 138
Cir_Forward.Primer  -------------------------------------------------------------------------------- -
Cir_Reverse.Primer  -------------------------------------------------------------------------------- 20
                                           2.180               2.200               2.220               2.240
Genomic             GCACTCCAACCATCTTCCTATTCTCAGTTTTACATGTGCCAGCTTTTCCCGTCAATTTAGTCTCTTTCTGTGCTCTCATT 2240
cDNA(predicted)     -------------------------------------------------------------------------------- 888
CircularRNA         GCACTCCAACCATCTTCCTATTCTCAGTTTTACATGTGCCAGCTTTTCCCGTCAATTTAGTCTCTTTCTGTGCTCTCATT 218
Cir_Forward.Primer  -------------------------------------------------------------------------------- -
Cir_Reverse.Primer  -------------------------------------------------------------------------------- 20
```

```
                                           2.260                        2.280                        2.300                        2.320
Genomic             GATCAAATAGACTGTCGTGTGATCCTTGACAAATTCGGTCACGAGATTCAGGAGCGACAAACGAGGCAGGCGGTTGGGAC 2320
cDNA(predicted)     -------------------------------------------------------------------------------- 888
CircularRNA         GATCAAATAGACTGTCGTGTGATCCTTGACAAATTCGGTCACGAGATTCAGGAGCGACAAACGAGGCAGGCGGTTGGGAC 298
Cir_Forward.Primer  -------------------------------------------------------------------------------- -
Cir_Reverse.Primer  -------------------------------------------------------------------------------- 20

                                           2.340                        2.360                        2.380                        2.400
Genomic             TGGCACCAGACGTAGGGGGCTCTCGTATATGGACCAGGGGGTGCAGCCAGACTTGATTTGTGCTGCGACCATGGAGGACA 2400
cDNA(predicted)     -------------------------------------------------------------------------------- 888
CircularRNA         TGGCACCAGACGTAGGGGGCTCTCGTATATGGACCAGGGGGTGCAGCCAGACTTGATTTGTGCTGCGACCATGGAGGACA 378
Cir_Forward.Primer  --------------------------------------------------ACTTGATTTGTGCTGCGACCAT-------- 22
Cir_Reverse.Primer  -------------------------------------------------------------------------------- 20

                                           2.420                        2.440                        2.460                        2.480
Genomic             AGGAGAAGCAGGCGATGATCCATCACTGTAGGAGGGGACATGTCTCGTTTGATAAGATAAGTAGAATATTTCCGGATGTT 2480
cDNA(predicted)     -------------------------------------------------------------------------------- 888
CircularRNA         AGGAGAAGCAGGCGATGATCCATCACTGTAGGAGGGGACATGTCTCGTTTGATAAGATAAGTAGAATATTTCCGGATGTT 458
Cir_Forward.Primer  -------------------------------------------------------------------------------- 22
Cir_Reverse.Primer  -------------------------------------------------------------------------------- 20

                                           2.500                        2.520                        2.540                        2.560
Genomic             ATGTGTGGAATAGGCAAGGGCAAGCTGACATGTGATGCTTGCGAATATGCAAAACACACAAGAGCTACATATGTGAGTAA 2560
cDNA(predicted)     -------------------------------------------------------------------------------- 888
CircularRNA         -------------------------------------------------------------------------------- 458
Cir_Forward.Primer  -------------------------------------------------------------------------------- 22
Cir_Reverse.Primer  -------------------------------------------------------------------------------- 20
```

Real-Time PCR for the junction region of chromosome segregation protein_circular RNA (ID: Ch5:361207403-361207861)

By divergent primers ◀▶ on genomic DNA

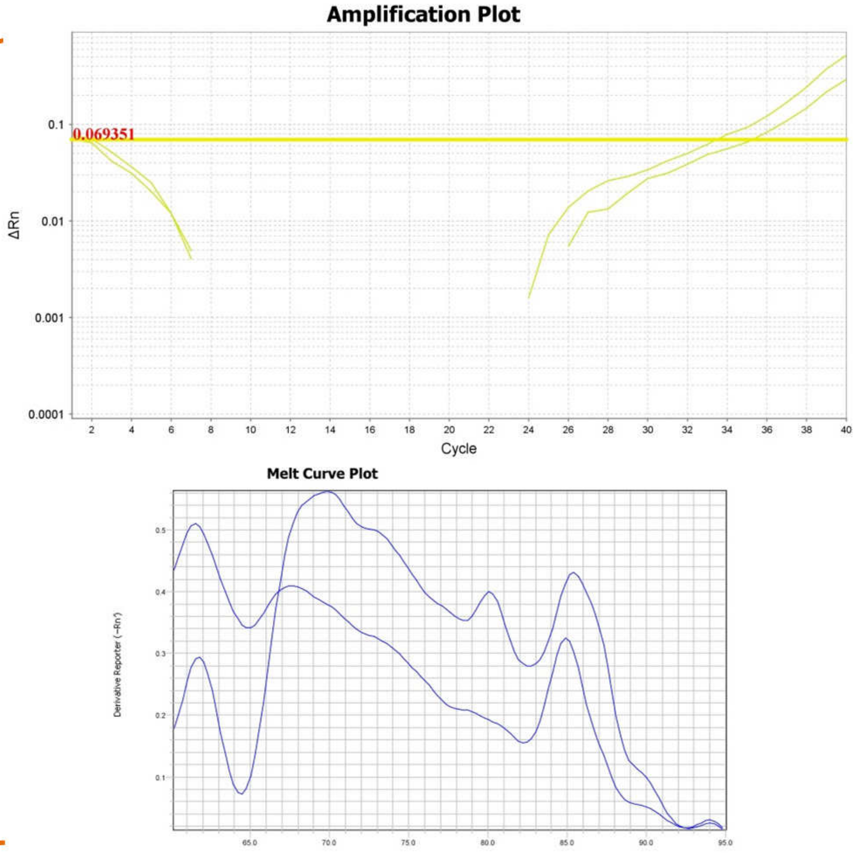

By divergent primers ◀▶ & with no template

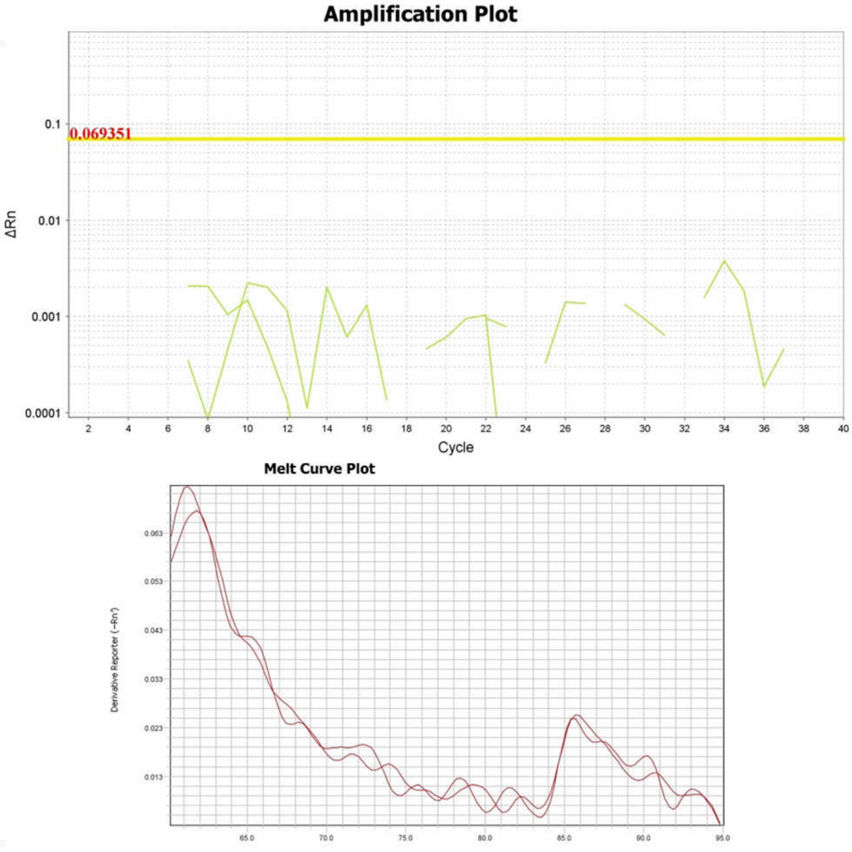

Laccase 12_circular RNA (ID: Ch3:499962355-499962651**)**

<u>C</u>ACCACCGCTATCCTCGAGTACGACTGCGGCTGTACCACAGAGTTGGGCCCCACCATCCCGC
CGGCCTTCCCGACCCTCCCGGCATTCAACGACACCGGCGCCGCCACGGCCTTCTCCGCGGGC
ATCAAGAGCCCACGGAAGGTCGAGATCCCCAGCCCCGTCGACGAGAACCTCTTCTTCACCGTC
GGCCTCGGGCTGTTCAACTGCGAGCCGGGGCAGCTATGCGGCGGGCCGAACAACAACACCCG
CTTCACGGCCAGCAT**GAACAACGTCTCCTTCGTCTTCCCCAAGGCCA<u>C</u>**

The nucleotides of junction-region are underlined. The nucleotides of junction-region which are supported by the junction-spaning sequencing reads are shown in red. Introns are not shown if the absence is supported by sequencing reads. In the absence of supporting sequencing reads, the intronic nucletides are shown as N.

Structural relationship between the circular RNA and its parental gene

↓

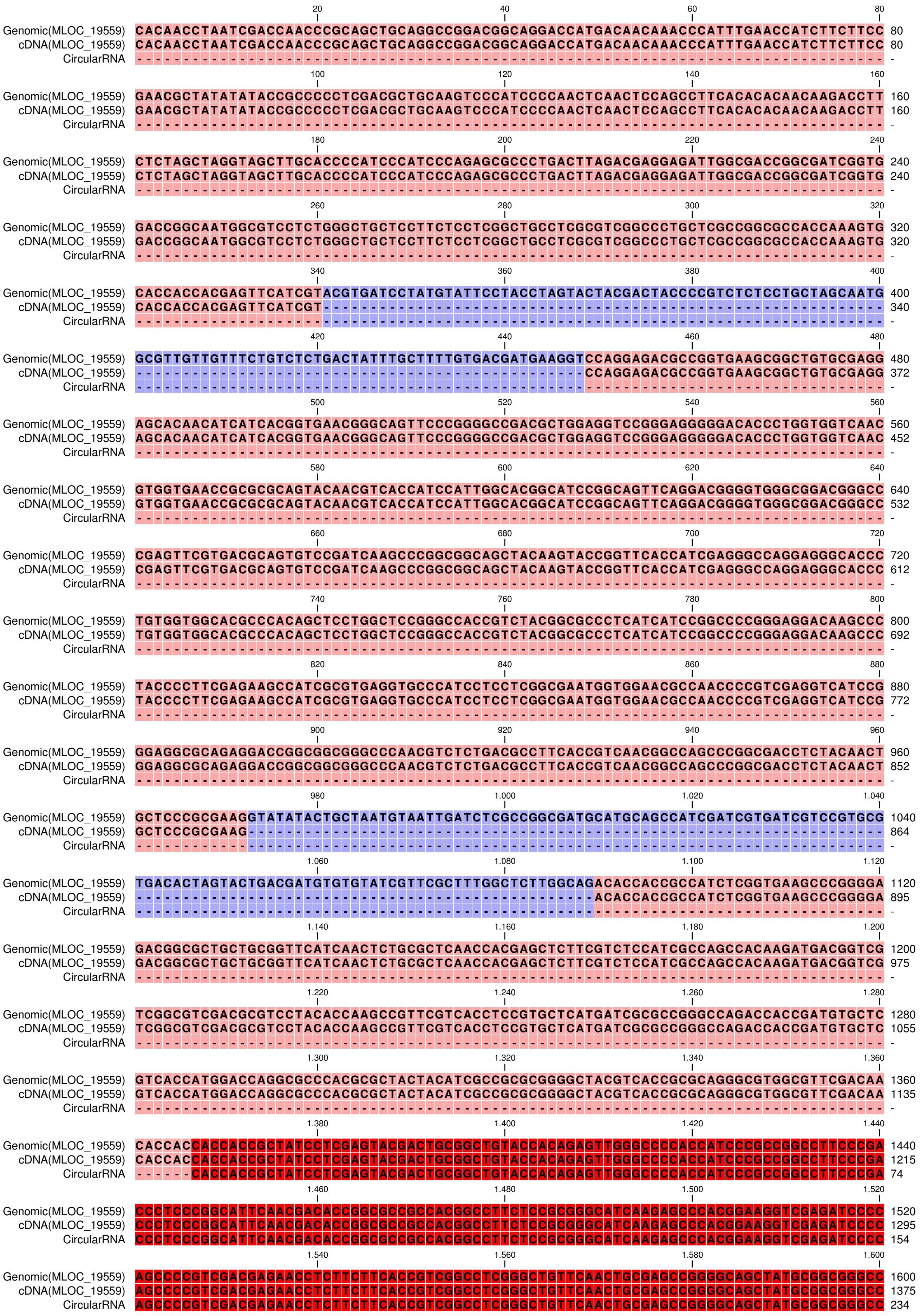
Genomic(MLOC_19559)
cDNA(MLOC_19559)
CircularRNA

```
                                                    1.620               1.640               1.660               1.680
Genomic(MLOC_19559) GAACAACAACACCCGCTTCACGGCCAGCATGAACAACGTCTCCTTCGTCTTCCCCAAGGCCACCTCCCTCCTCCACGCGC 1680
   cDNA(MLOC_19559) GAACAACAACACCCGCTTCACGGCCAGCATGAACAACGTCTCCTTCGTCTTCCCCAAGGCCACCTCCCTCCTCCACGCGC 1455
        CircularRNA GAACAACAACACCCGCTTCACGGCCAGCATGAACAACGTCTCCTTCGTCTTCCCCAAGGCCAC----------------- 297

                                                    1.700               1.720               1.740               1.760
Genomic(MLOC_19559) ACTACTACGACATGCCGGGCGTGTTCACCACTGACTTCCCGGCCTACCCGCCGGTGCAGTTCGACTACACGGCGCAGAAC 1760
   cDNA(MLOC_19559) ACTACTACGACATGCCGGGCGTGTTCACCACTGACTTCCCGGCCTACCCGCCGGTGCAGTTCGACTACACGGCGCAGAAC 1535
        CircularRNA -------------------------------------------------------------------------------- 297

                                                    1.780               1.800               1.820               1.840
Genomic(MLOC_19559) GTCAGCCAAAGCCTGTGGCAGCCGATTCCGGCGACCAAGCTATACAAGCTCAGGTTCGGCTCCGTGGTGCAGGTCGTCCT 1840
   cDNA(MLOC_19559) GTCAGCCAAAGCCTGTGGCAGCCGATTCCGGCGACCAAGCTATACAAGCTCAGGTTCGGCTCCGTGGTGCAGGTCGTCCT 1615
        CircularRNA -------------------------------------------------------------------------------- 297

                                                    1.860               1.880               1.900               1.920
Genomic(MLOC_19559) GCAGGACACCAGCATCGTCACGCCGGAGAACCATCCCATCCACCTCCACGGATACGACTTCTACATCCTCGCCGAGGGCT 1920
   cDNA(MLOC_19559) GCAGGACACCAGCATCGTCACGCCGGAGAACCATCCCATCCACCTCCACGGATACGACTTCTACATCCTCGCCGAGGGCT 1695
        CircularRNA -------------------------------------------------------------------------------- 297

                                                    1.940               1.960               1.980               2.000
Genomic(MLOC_19559) TCGGCAACTACGACGCCGAGAAGGACGCCGACAAGTTCAACCTCGAGAACCCACCTCAGAGGAATACCGTGGCGGTGCCG 2000
   cDNA(MLOC_19559) TCGGCAACTACGACGCCGAGAAGGACGCCGACAAGTTCAACCTCGAGAACCCACCTCAGAGGAATACCGTGGCGGTGCCG 1775
        CircularRNA -------------------------------------------------------------------------------- 297

                                                    2.020               2.040               2.060               2.080
Genomic(MLOC_19559) GTGAACGGCTGGGCGGTCATCCGGTTCCGCGCCGACAACCCGGGGGTGTGGCTCATGCACTGCCATCTCGATGTGCACAT 2080
   cDNA(MLOC_19559) GTGAACGGCTGGGCGGTCATCCGGTTCCGCGCCGACAACCCGGGGGTGTGGCTCATGCACTGCCATCTCGATGTGCACAT 1855
        CircularRNA -------------------------------------------------------------------------------- 297

                                                    2.100               2.120               2.140               2.160
Genomic(MLOC_19559) CACCTGGGGCCTGGCAATGGCGTTTCTGGTGGAGGACGGATATGGCGAGCTTCAGTCCCTGGAGGCGCCTCCAGTTGATC 2160
   cDNA(MLOC_19559) CACCTGGGGCCTGGCAATGGCGTTTCTGGTGGAGGACGGATATGGCGAGCTTCAGTCCCTGGAGGCGCCTCCAGTTGATC 1935
        CircularRNA -------------------------------------------------------------------------------- 297

                                                    2.180               2.200               2.220               2.240
Genomic(MLOC_19559) TTCCAATGTGCTAATCACCAGCAAAAAGATACGATCGATATTCAAGGCTGCCCACGTTCTATTATCGGTTCCTGCCATTT 2240
   cDNA(MLOC_19559) TTCCAATGTGCTAATCACCAGCAAAAAGATACGATCGATATTCAAGGCTGCCCACGTTCTATTATCGGTTCCTGCCATTT 2015
        CircularRNA -------------------------------------------------------------------------------- 297

                                                    2.260               2.280               2.300               2.320
Genomic(MLOC_19559) AGGGGGGGTATTTGTGATCTTTATTTTATACTTCTAGGGGGGTTGATTTTCCATCATGCTTTGTAACTTTCCCCTGTAAA 2320
   cDNA(MLOC_19559) AGGGGGGGTATTTGTGATCTTTATTTTATACTTCTAGGGGGGTTGATTTTCCATCATGCTTTGTAACTTTCCCCTGTAAA 2095
        CircularRNA -------------------------------------------------------------------------------- 297

                                                    2.340
Genomic(MLOC_19559) TTTGCGTATTTTCAGTAATTCATCAGCTTATTA 2353
   cDNA(MLOC_19559) TTTGCGTATTTTCAGTAATTCATCAGCTTATTA 2128
        CircularRNA --------------------------------- 297
```

ARID/BRIGHT DNA-binding protein_circular RNA (ID: Ch2:532048153-532048410**)**

GTGACATCATGCAAACTGTGGCGTCAAGTGGGAGAGTCATTCAAACCTCCGAAGTATGAGTTC
AACCGATTGTCTTCTGCAATAATCTCTTCCTGGCTTTTTCATTTGATAAATCTCTATTGTAATTGTG
CAATGGTGGAGTTGAAATGTAATTGACATGTGCTGTTCTTTCTCTACTCTCTCCTATCACGTAAC
CAACATATTTTATGCA**GGACATGCACAACCGTTTCATGGACCTTCCGTAACTTCTATGAGAAG**

The nucleotides of junction-region are underlined. The nucleotides of junction-region which are supported by the junction-spaning sequencing reads are shown in red. Introns are not shown if the absence is supported by sequencing reads. In the absence of supporting sequencing reads, the intronic nucletides are shown as N.

Structural relationship between the circular RNA and its parental gene

```
                                                   20                  40                  60                  80
                                                    |                   |                   |                   |
Genomic      TTTTAGGACTAGCTTGACACCCCTCGTAGTTATAGGACCTCTGATGCGTTTTACTCATCTTTTCTCTATGCTTTTGGTAC 80
CircularRNA  -------------------------------------------------------------------------------- -

                                                  100                 120                 140                 160
                                                    |                   |                   |                   |
Genomic      CTTTGTTATGCTACCATACTCATGTAATCCACACGTCTACTCATCTGCCGAACTTGTTTGTACCCTATGCCTTTATATCA 160
CircularRNA  -------------------------------------------------------------------------------- -

                                                  180                 200                 220                 240
                                                    |                   |                   |                   |
Genomic      GATCCTGTTAATTGGATGCACCTTACACAAAGTATGAAGCGTCATTTTTAATATGATACCTTTCTCTCTTCCATATGCTG 240
CircularRNA  -------------------------------------------------------------------------------- -

                                                  260                 280                 300                 320
                                                    |                   |                   |                   |
Genomic      TTTACAATCCTTGTTCAACTATTTAATTGATTATCTACTAATGTTTTCTCTATGGACAATGACTCAAGTTCTATCACCAA 320
CircularRNA  -------------------------------------------------------------------------------- -

                                                  340                 360                 380                 400
                                                    |                   |                   |                   |
Genomic      CATAGAAGTAGGGACAGACTCCATTTAGTTTGTTGGTATTAGAAATACGACCCAAAACCTCGTGCTTACTGTCCTACCTG 400
CircularRNA  -------------------------------------------------------------------------------- -

                                                  420                 440                 460                 480
                                                    |                   |                   |                   |
Genomic      GCTCATGCAGCCAATGAGGCATTCCTTTTCCCATGATCTTGCCTGGCATTATTATCATATTTTGTGTTTTCAGTAAGTGA 480
CircularRNA  -------------------------------------------------------------------------------- -

                                                  500                 520                 540                 560
                                                    |                   |                   |                   |
Genomic      GTTGTAAAGTACTTCCTCAGCTCAGAATTAGTTGCCACTCAAACGGATGTATCTAGTACTGAAAATACGTCTAGATACAT 560
CircularRNA  -------------------------------------------------------------------------------- -

                                                  580                 600                 620                 640
                                                    |                   |                   |                   |
Genomic      CCGTTTGAGCGTCAACTAATTCCGGACGGAGGGAGTATATGATTTAAGAAAAGCAATGGTAAGAATCACATCAGAGGGCA 640
CircularRNA  -------------------------------------------------------------------------------- -

                                                  660                 680                 700                 720
                                                    |                   |                   |                   |
Genomic      AAAGGTTAACCATATTGAAGAAATCAACATAACTAATAAGGATTTCTTGCAAGCCAAGATTTCTGTTTTTTGGAAAGACA 720
CircularRNA  -------------------------------------------------------------------------------- -

                                                  740                 760                 780                 800
                                                    |                   |                   |                   |
Genomic      AAACACCAGCCTGAATGATAGCTTGACCATGTGCAGTGGAAGCAATGGCACTAATCATAGTTTTTAATGATGATGAAATT 800
CircularRNA  -------------------------------------------------------------------------------- -

                                                  820                 840                 860                 880
                                                    |                   |                   |                   |
Genomic      CTCTATGAGCATATATGCATACACAATTAGCAAACACCTAGTTGCTTAAGAGGAAAGTCAAGCTTTGCCCTTCACTTAAT 880
CircularRNA  -------------------------------------------------------------------------------- -

                                                  900                 920                 940                 960
                                                    |                   |                   |                   |
Genomic      ATCTCCTTTTCTCTCCAGAATGAGGGTAAGGTACATTTAGACTGTCCGATATTTCTTTGTTTGACTACAGAAGTATAGAT 960
CircularRNA  -------------------------------------------------------------------------------- -

                                                  980               1.000               1.020               1.040
                                                    |                   |                   |                   |
Genomic      GTGTTTGCTTGTCATTTTGTAACTTTGCCTGTAACTTTGTATAACATTTAGCCAATGTGCGGTTGCATTCTTTCTTATAT 1040
CircularRNA  -------------------------------------------------------------------------------- -

                                                1.060               1.080               1.100               1.120
                                                    |                   |                   |                   |
Genomic      GTTGCATTAACAGGTGACATCATGCAAACTGTGGCGTCAAGTGGGAGAGTCATTCAAACCTCCGAAGTATGAGTTCAACC 1120
CircularRNA  -------------GTGACATCATGCAAACTGTGGCGTCAAGTGGGAGAGTCATTCAAACCTCCGAAGTATGAGTTCAACC 67

                                                1.140               1.160               1.180               1.200
                                                    |                   |                   |                   |
Genomic      GATTGTCTTCTGCAATAATCTCTTCCTGGCTTTTTCATTTGATAAATCTCTATTGTAATTGTGCAATGGTGGAGTTGAAA 1200
CircularRNA  GATTGTCTTCTGCAATAATCTCTTCCTGGCTTTTTCATTTGATAAATCTCTATTGTAATTGTGCAATGGTGGAGTTGAAA 147

                                                1.220               1.240               1.260               1.280
                                                    |                   |                   |                   |
Genomic      TGTAATTGACATGTGCTGTTCTTTCTCTACTCTCTCCTATCACGTAACCAACATATTTTATGCAGGACATGCACAACCGT 1280
CircularRNA  TGTAATTGACATGTGCTGTTCTTTCTCTACTCTCTCCTATCACGTAACCAACATATTTTATGCAGGACATGCACAACCGT 227

                                                1.300               1.320               1.340               1.360
                                                    |                   |                   |                   |
Genomic      TTCATGGACCTTCCGTAACTTCTATGAGAAGGTACTTCTCTATATTCTCAGAGATGATCATGTCATTGTTTATAACTCCA 1360
CircularRNA  TTCATGGACCTTCCGTAACTTCTATGAGAAG------------------------------------------------- 258

                                                1.380               1.400               1.420               1.440
                                                    |                   |                   |                   |
Genomic      CACTTGCTCTGCTCTTGTGGTTGAATCCTGTCATGTGGTATGAATGCCATGGTAGTTGCTTGAGCAAGACCTTTTGCACT 1440
CircularRNA  -------------------------------------------------------------------------------- 258

                                                1.460               1.480               1.500               1.520
                                                    |                   |                   |                   |
Genomic      TACTCAGATTAACTCGTTGAAAGTTTTACCAAGTTCATAACATAGAACTATGCCATAAGTCCAATCTCCAGAACTGGCAA 1520
CircularRNA  -------------------------------------------------------------------------------- 258

                                                1.540               1.560               1.580               1.600
                                                    |                   |                   |                   |
Genomic      TTCTGGTTCAAATTTAATACTACTCCCTCCGTCCGGAATTACTTGTCACACAAATGTATAAAAATGGATGTACCTAAAAC 1600
CircularRNA  -------------------------------------------------------------------------------- 258

                                                1.620               1.640               1.660               1.680
                                                    |                   |                   |                   |
Genomic      TAAAATACATCTAGATACATTCATTCCTATGACAAGTATTTCCGGACGGAGGGAGTAAATAANNNNNNNNNNNNNNNNNN 1680
CircularRNA  -------------------------------------------------------------------------------- 258
```

Ubiquitin-conjugating enzyme 11_circular RNA (ID: Ch3:473869516-473869731)

GTTCTGCTTTCAATCTGCTCGCTGCTTACCGACCCTAACCCGGACGACCCTCTCGTCCCTGAG
ATTGCCCACATGTACAAGACGGATCGGTCTAAGTACGAGACGACAGCCCGCAGCTGGACGCAG
AAGTATGCCATGGGATGATACGGAGCCCATGCTCGTATCCACTGCTT**GCATGCAGAAGATTGT**
GTTGCTGTCCCGGGAAACTATCTCTGG

The nucleotides of junction-region are underlined. The nucleotides of junction-region which are supported by the junction-spaning sequencing reads are shown in red. Introns are not shown if the absence is supported by sequencing reads. In the absence of supporting sequencing reads, the intronic nucletides are shown as N.

Structural relationship between the circular RNA and its parental gene

```
                                                 20                  40                  60                  80
Genomic          GGTGTGATGGAACAAATATGTTAAGGTTAACGAATCATTACTTAATCGAAGCTTCGTGTTCTAGATTACTTTGGACAAAT 80
cDNA(predicted)  -------------------------------------------------------------------------------- -
CircularRNA      -------------------------------------------------------------------------------- -

                                                100                 120                 140                 160
Genomic          ATATACGAGTAAGTAGTGAAGTTACTTCAGCGCCAGGCTAAATACTTTGAAGCAATAAACCCGTATTGTTGTATCTTTTG 160
cDNA(predicted)  -------------------------------------------------------------------------------- -
CircularRNA      -------------------------------------------------------------------------------- -

                                                180                 200                 220                 240
Genomic          CTTTTTATTATTCTTATGCTGCCAACTTATTGCAGTATGTGGTGAACGAAAATATGTTTAAACTCTAGAGTTAGTAACTT 240
cDNA(predicted)  -------------------------------------------------------------------------------- -
CircularRNA      -------------------------------------------------------------------------------- -

                                                260                 280                 300                 320
Genomic          ATTCTTGAGTCTCATAAACATGCAAATGTAACTCAAAGCAGGAGCTTATCATGGCTTCAAAACGTATCCTGAAGGAACTC 320
cDNA(predicted)  --------------------------------------------------ATGGCTTCAAAACGTATCCTGAAGGAACTC 30
CircularRNA      -------------------------------------------------------------------------------- -

                                                340                 360                 380                 400
Genomic          AAGGACTTGCAGAAAGATCCTCCGACATCATGCAGTGCAGGTACGAGCCAGATATATAATTCACTGATGACTAATATCCA 400
cDNA(predicted)  AAGGACTTGCAGAAAGATCCTCCGACATCATGCAGTGCAGGT-------------------------------------- 72
CircularRNA      -------------------------------------------------------------------------------- -

                                                420                 440                 460                 480
Genomic          TGTTGTGTGTATCTTAGCAGCGTTGATATGCTATGGTCATGTGAACTATCACCGGAAGTTTTTATGGCGTCGGCCCATTG 480
cDNA(predicted)  -------------------------------------------------------------------------------- 72
CircularRNA      -------------------------------------------------------------------------------- -

                                                500                 520                 540                 560
Genomic          ATAAATTGGATTATTTCCTAATATACTTTTCATTTTGCTCAGTCACTTATCCAGGAAATTGAACTATAACACACAGTTTC 560
cDNA(predicted)  -------------------------------------------------------------------------------- 72
CircularRNA      -------------------------------------------------------------------------------- -

                                                580                 600                 620                 640
Genomic          ATTCACTGTTCTTGCCCAAAGAGATTGTATCACTATGCCAGTTTCTGTCTGTCAGTGTCCTTTTACGGTTGACTAGCTAA 640
cDNA(predicted)  -------------------------------------------------------------------------------- 72
CircularRNA      -------------------------------------------------------------------------------- -

                                                660                 680                 700                 720
Genomic          ATTGATGTTTTTATTTAAGAAAGGTATCTTGCATAGAGTCTTCCTAAGTACTCTGTATATTCCAACTTAGTAACTGATGA 720
cDNA(predicted)  -------------------------------------------------------------------------------- 72
CircularRNA      -------------------------------------------------------------------------------- -

                                                740                 760                 780                 800
Genomic          ACAGTCATCAACAACCACTACAATTGACCATCAGTTCTATAATCAGGATTATGTCGTGCTGCTGCTTGGTGCTTACTCCC 800
cDNA(predicted)  -------------------------------------------------------------------------------- 72
CircularRNA      -------------------------------------------------------------------------------- -

                                                820                 840                 860                 880
Genomic          TTGTTTGCACAAATGATTGTAGTGATTTATTATAGGTTCCTTTTTATTTAGGTCCTTCTGGCGAGGATATGTTCCATTGG 880
cDNA(predicted)  -----------------------------------------------------CCTTCTGGCGAGGATATGTTCCATTGG 99
CircularRNA      -------------------------------------------------------------------------------- -

                                                900                 920                 940                 960
Genomic          CAGGCAACCATCATGGGCCCTCCTGATAGTCCCTATGCTGGAGGTGTTTTCTTGGTGAATATCCATTTCCCCCCGGACTA 960
cDNA(predicted)  CAGGCAACCATCATGGGCCCTCCTGATAGTCCCTATGCTGGAGGTGTTTTCTTGGTGAATATCCATTTCCCCCCGGACTA 179
CircularRNA      -------------------------------------------------------------------------------- -

                                                980               1.000               1.020               1.040
Genomic          CCCCTTCAAGCCTCCGAAGGTTAGTGCTGAACAATGTTGAGCCAAACAAATTTCCTTGCATCTTTGCCATCTTTATTGGA 1040
cDNA(predicted)  CCCCTTCAAGCCTCCGAAGGT----------------------------------------------------------- 200
CircularRNA      -------------------------------------------------------------------------------- -

                                              1.060               1.080               1.100               1.120
Genomic          ACATGAATCTTGTCAAACTGTCAGTATGTCTACCCTCTTCATGTTCCATTGATGTTTCAGTGTTAGCTTGTCTGATTTAT 1120
cDNA(predicted)  -------------------------------------------------------------------------------- 200
CircularRNA      -------------------------------------------------------------------------------- -

                                              1.140               1.160               1.180               1.200
Genomic          GTTCATCCCTACATTTTGACATTAAGGACCAACTATATCTTTTTATATCTCAATGTTTTTACAGAAATCGTTATTATATC 1200
cDNA(predicted)  -------------------------------------------------------------------------------- 200
CircularRNA      -------------------------------------------------------------------------------- -

                                              1.220               1.240               1.260               1.280
Genomic          ACCTCTTCTTTTTTAGAAAAATATTCTGTTTCTGAATCTATCTTTTCTTTTATCACCCCATTCTATGACAGGGTCAACTT 1280
cDNA(predicted)  -------------------------------------------------------------------------------- 200
CircularRNA      -------------------------------------------------------------------------------- -

                                              1.300               1.320               1.340               1.360
Genomic          TGTGGCCCTACTTGTTTTTTTAATCTTCTCTTTTCCTCCTTGTACTGTTCTAAAGTGTCTCTTCCTTCAGGTATCGTTCA 1360
cDNA(predicted)  ------------------------------------------------------------------------ATCGTTCA 208
CircularRNA      -------------------------------------------------------------------------------- -

                                              1.380               1.400               1.420               1.440
Genomic          AGACAAAGGTCTTCCATCCGAACATCAATAGCAATGGAAGCATATGCCTCGACATTCTGAAGGAGCAATGGAGTCCTGCT 1440
cDNA(predicted)  AGACAAAGGTCTTCCATCCGAACATCAATAGCAATGGAAGCATATGCCTCGACATTCTGAAGGAGCAATGGAGTCCTGCT 288
CircularRNA      -------------------------------------------------------------------------------- -

                                              1.460               1.480               1.500               1.520
Genomic          TTGACGATCTCTAAGGTGCTCTCAAAGCATTTCATTCCTTTACATCTTCTCTGTTGTGCTGTATATACAATAGTAAATGA 1520
cDNA(predicted)  TTGACGATCTCTAAGG---------------------------------------------------------------- 304
CircularRNA      -------------------------------------------------------------------------------- -

                                              1.540               1.560               1.580               1.600
Genomic          GTTATTTCCATGCCCTTGCACTCTCGATGAGCTACAGTTGTTTGTATTGCCTCATTACACAATTTATAATCTCAACTTAC 1600
cDNA(predicted)  -------------------------------------------------------------------------------- 304
CircularRNA      -------------------------------------------------------------------------------- -
```

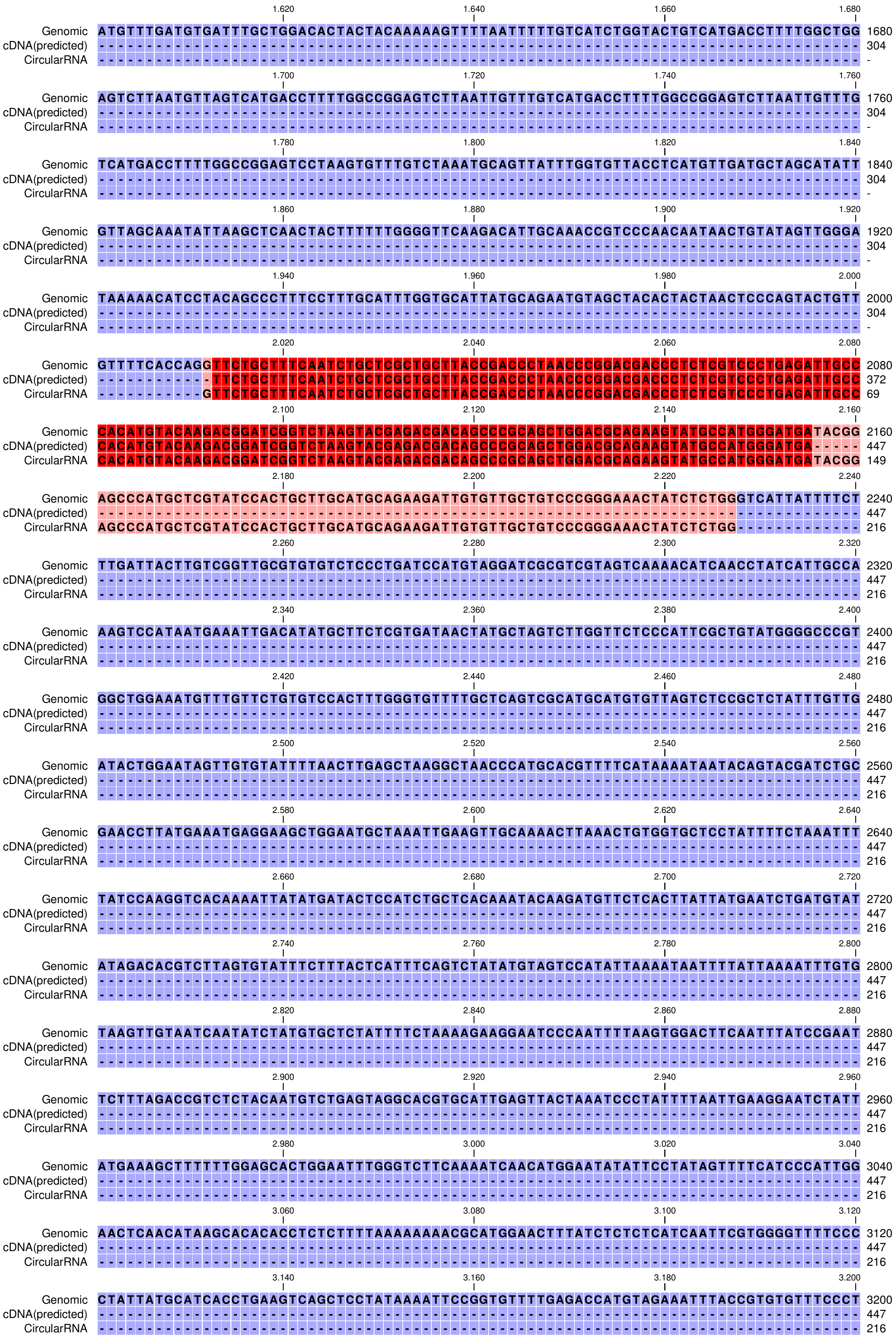
Genomic
cDNA(predicted)
CircularRNA
1.620
1.640
1.660
1.680
1.700
1.720
1.740
1.760
1.780
1.800
1.820
1.840
1.860
1.880
1.900
1.920
1.940
1.960
1.980
2.000
2.020
2.040
2.060
2.080
2.100
2.120
2.140
2.160
2.180
2.200
2.220
2.240
2.260
2.280
2.300
2.320
2.340
2.360
2.380
2.400
2.420
2.440
2.460
2.480
2.500
2.520
2.540
2.560
2.580
2.600
2.620
2.640
2.660
2.680
2.700
2.720
2.740
2.760
2.780
2.800
2.820
2.840
2.860
2.880
2.900
2.920
2.940
2.960
2.980
3.000
3.020
3.040
3.060
3.080
3.100
3.120
3.140
3.160
3.180
3.200

```
                  3.220               3.240               3.260               3.280
Genomic           ATCCCTATTCATATGTTTTCAGTTTCATGTTCCTGCAACTTTTGTTTGGAACAGACTGCATACCAAGGCCGAATGCTGAA 3280
cDNA(predicted)   -------------------------------------------------------------------------------- 447
CircularRNA       -------------------------------------------------------------------------------- 216

                  3.300               3.320               3.340               3.360
Genomic           ACCAAGTCTAGGTCGATTGTGGGGTTCGCTAAAATCATATGTATTATTAAAAAACATGAAAGTTTGGCATATCAATCGGC 3360
cDNA(predicted)   -------------------------------------------------------------------------------- 447
CircularRNA       -------------------------------------------------------------------------------- 216

                  3.380               3.400               3.420               3.440
Genomic           CTATTAGCTCAGCTGGTTAGAGCGTCGTGCTAATAACGCGAAGGTCGCAGGTTCGAGACCTGCATGGGCCAATATTTTTT 3440
cDNA(predicted)   -------------------------------------------------------------------------------- 447
CircularRNA       -------------------------------------------------------------------------------- 216

                  3.460               3.480               3.500               3.520
Genomic           TAGATAAAAAGTCGTTGCGAATTTTTGTTGTTAAATTCCATTTCTTTTACAACAGTGCTAAATTCTGTTTTGCCCGCATG 3520
cDNA(predicted)   -------------------------------------------------------------------------------- 447
CircularRNA       -------------------------------------------------------------------------------- 216

                  3.540               3.560               3.580               3.600
Genomic           CACACTCCTCTCCTGATGTTCGCCTAGTGTGACAGTTAGGCCATGTTCGGATACTCTCCGCTCCTCGATCGCAGCTCCCG 3600
cDNA(predicted)   -------------------------------------------------------------------------------- 447
CircularRNA       -------------------------------------------------------------------------------- 216

                  3.620               3.640               3.660               3.680
Genomic           GAGTGGAAAGAGCGTCAACACAATTGCAGGGAGCAGCTCAAACCCAGCTCCTCGCGCGGAGCGGACTTATGATGCGGGAG 3680
cDNA(predicted)   -------------------------------------------------------------------------------- 447
CircularRNA       -------------------------------------------------------------------------------- 216

                  3.700               3.720               3.740               3.760
Genomic           AGCTTCCGAACAGGCACTTAGATTTGCCGCTGTTTTTTTCTTCTTTTATGATTTTGGCATGCCTTCAGCACACAAGTACA 3760
cDNA(predicted)   -------------------------------------------------------------------------------- 447
CircularRNA       -------------------------------------------------------------------------------- 216

                  3.780               3.800               3.820               3.840
Genomic           CAAACCATTGTTCTCCTAGCTCTACCCAGCACGCACCGTGTTGAACAGAAGCAAAAACAAGTTATCGCTCTCGTGTTCCT 3840
cDNA(predicted)   -------------------------------------------------------------------------------- 447
CircularRNA       -------------------------------------------------------------------------------- 216

                  3.860               3.880               3.900               3.920
Genomic           GAAAATAACAGTTCACAAAACCATGACCGCACAGGCTATTTATCATCTCTTTTCTGATTGCTAAGTTCTGTTCAAGTGTT 3920
cDNA(predicted)   -------------------------------------------------------------------------------- 447
CircularRNA       -------------------------------------------------------------------------------- 216

                  3.940               3.960               3.980               4.000
Genomic           TGTTGAATCCAATCGCACAGGCTTCTGGCTTCTGTATTGTTGTCTGAATCTGTTTCTTTACGTTGAAGTCCCCGTGTTCG 4000
cDNA(predicted)   -------------------------------------------------------------------------------- 447
CircularRNA       -------------------------------------------------------------------------------- 216

                  4.020               4.040               4.060               4.080
Genomic           ACTTCTGGTGGTGGTGGGAAACATCTCCCCGTTTGGGTTGAGGATACCGCAAACTTGAACGACCATAACAGTGTTCATAA 4080
cDNA(predicted)   -------------------------------------------------------------------------------- 447
CircularRNA       -------------------------------------------------------------------------------- 216

                  4.100               4.120               4.140               4.160
Genomic           ACCAAAAGAATTACCAATGAGAGAACGGCGGTGGTTCTGAACAAAAAAAAAGATACTCCCTCCATCCTATAATATAAGAG 4160
cDNA(predicted)   -------------------------------------------------------------------------------- 447
CircularRNA       -------------------------------------------------------------------------------- 216

                  4.180               4.200               4.220
Genomic           CGTTTTTGACACCACGCATTTGTTGAGCATATTTTTTGAGGAGGATAAAACAACTCAGAGATTTGTTGGTAGTACCG 4237
cDNA(predicted)   ----------------------------------------------------------------------------- 447
CircularRNA       ----------------------------------------------------------------------------- 216
```

Probable microtubule-stabilizing protein_circular RNA

(ID: Ch4:330511991-330512323)

CGACGCCCAAGTGAACACAATGAATCCAAGCGAAAACAGGATGAGTATGATGAGGAAGGCAA
TAGCTACCAAGAAGATGAAATCAATGATGGCGAGGGTGATACTCTTGACTTGGCTAATGAGTTCA
CAGATGTGCATCTTGACTTGGATGATGAGTTTGCAGATGAAGATAATACAACTGCAGAAATGGAA
CCAGTTATACTTGGATTCGATCAGGGTGTACAGGTTGAAATTCCCGTGAATAATGAGCTTGAATT
AAGTTCTGTGAAGAGCACTGAACTGGAAGTTGGTG**TACACTTGGGCGTCGTGAAGCAAGAGTT**
GAGATGTGGTTCA

The nucleotides of junction-region are underlined. The nucleotides of junction-region which are supported by the junction-spaning sequencing reads are shown in red. Introns are not shown if the absence is supported by sequencing reads. In the absence of supporting sequencing reads, the intronic nucletides are shown as N.

Structural relationship between the circular RNA and its parental gene

```
                                   20                  40                  60                  80
Genomic(MLOC_11124)  AGCGTTTGGAGGGCGCGCCCGCCACTACTGGCGGTGGGGTCGCCTCAGCCCCGCCCCGATCCGGCGGGGCCGTGGCTGGG 80
cDNA(MLOC_11124)     AGCGTTTGGAGGGCGCGCCCGCCACTACTGGCGGTGGGGTCGCCTCAGCCCCGCCCCGATCCGGCGGGGCCGTGGCTGGG 80
CircularRNA          -------------------------------------------------------------------------------- -

                                  100                 120                 140                 160
Genomic(MLOC_11124)  TGGACGAAGCCTGCTCCGGCATCCGAGAAGCAACTGGGCTCGATACCGGCTCCTAGTGGCGTTGCCAGGCTCCCATCGTA 160
cDNA(MLOC_11124)     TGGACGAAGCCTGCTCCGGCATCCGAGAAGCAACTGGGCTCGATACCGGCTCCTAGTGGCGTTGCCAGGCTCCCATCGTA 160
CircularRNA          -------------------------------------------------------------------------------- -

                                  180                 200                 220                 240
Genomic(MLOC_11124)  TGGATTCCAGGAGAAAGCTGTCGTCCTGCGGGGCGAGGACTTCCCTTCTCTTAAAGCAGCGGTGGCGCCACCACCTGCGC 240
cDNA(MLOC_11124)     TGGATTCCAGGAGAAAGCTGTCGTCCTGCGGGGCGAGGACTTCCCTTCTCTTAAAGCAGCGGTGGCGCCACCACCTGCGC 240
CircularRNA          -------------------------------------------------------------------------------- -

                                  260                 280                 300                 320
Genomic(MLOC_11124)  CACCCGTGCAGCATCGCCAGAAAGACGTTGATGGTGCTCAAGCCGCCATGCCGGAGACACAGCCGATGCCCCTGGGCATG 320
cDNA(MLOC_11124)     CACCCGTGCAGCATCGCCAGAAAGACGTTGATGGTGCTCAAGCCGCCATGCCGGAGACACAGCCGATGCCCCTGGGCATG 320
CircularRNA          -------------------------------------------------------------------------------- -

                                  340                 360                 380                 400
Genomic(MLOC_11124)  CGACCGCATGTGATGCCCTCGCGTGGTGCTGAGCCCTTGGCTTCTGCCGGCGTCACAGGTACTGGCCTCCATGGTTCAGC 400
cDNA(MLOC_11124)     CGACCGCATGTGATGCCCTCGCGTGGTGCTGAGCCCTTGGCTTCTGCCGGCGTCACAGGTACTGGCCTCCATGGTTCAGC 400
CircularRNA          -------------------------------------------------------------------------------- -

                                  420                 440                 460                 480
Genomic(MLOC_11124)  GGAGAAGGCCCAAACTCATGATTTGGGGCCGCTGCCACTGGTGCGGCTAAGGTATGATGCTGATTGGGCTGATGATGAAC 480
cDNA(MLOC_11124)     GGAGAAGGCCCAAACTCATGATTTGGGGCCGCTGCCACTGGTGCGGCTAAGGTATGATGCTGATTGGGCTGATGATGAAC 480
CircularRNA          -------------------------------------------------------------------------------- -

                                  500                 520                 540                 560
Genomic(MLOC_11124)  GTGATACAGGGCTGAGTCTCCCAGATCGCGACAGTAAAGAGAGGGGATTTGGTAGGATTGAGACCATGGTTCCAGGGTGC 560
cDNA(MLOC_11124)     GTGATACAGGGCTGAGTCTCCCAGATCGCGACAGTAAAGAGAGGGGATTTGGTAGGATTGAGACCATGGTTCCAGGGTGC 560
CircularRNA          -------------------------------------------------------------------------------- -

                                  580                 600                 620                 640
Genomic(MLOC_11124)  GACTTCTATGGTGCGACAATGGAGCGCTTAAAAAATGAGTCCTTGGGGAGAGATTACATTGCTCCCAATAAAGAGGGCGT 640
cDNA(MLOC_11124)     GACTTCTATGGTGCGACAATGGAGCGCTTAAAAAATGAGTCCTTGGGGAGAGATTACATTGCTCCCAATAAAGAGGGCGT 640
CircularRNA          -------------------------------------------------------------------------------- -

                                  660                 680                 700                 720
Genomic(MLOC_11124)  GCAAGATGGTTTGTGGCGATCTCCTATGCCAAGCCACAATGTGGAGAGGACAGATGGTCGTCCTCACAGTGCAGGCAAAG 720
cDNA(MLOC_11124)     GCAAGATGGTTTGTGGCGATCTCCTATGCCAAGCCACAATGTGGAGAGGACAGATGGTCGTCCTCACAGTGCAGGCAAAG 720
CircularRNA          -------------------------------------------------------------------------------- -

                                  740                 760                 780                 800
Genomic(MLOC_11124)  GAAGCGGACAGTTACTTTACCATGAAGGCATTACTAACGGTGCCTCCAAGGATTTGTGCAATACTAGTAAGGAGCCTGCT 800
cDNA(MLOC_11124)     GAAGCGGACAGTTACTTTACCATGAAGGCATTACTAACGGTGCCTCCAAGGATTTGTGCAATACTAGTAAGGAGCCTGCT 800
CircularRNA          -------------------------------------------------------------------------------- -

                                  820                 840                 860                 880
Genomic(MLOC_11124)  GTGCGAGCCTATGGACAGATTGGGACGGAACTGCACGGAAGCGCACATATTGGGGAAACTGCAGGTGAATGTTACAATGA 880
cDNA(MLOC_11124)     GTGCGAGCCTATGGACAGATTGGGACGGAACTGCACGGAAGCGCACATATTGGGGAAACTGCAGGTGAATGTTACAATGA 880
CircularRNA          -------------------------------------------------------------------------------- -

                                  900                 920                 940                 960
Genomic(MLOC_11124)  TAATTCTAATAACTGGTACAGAGGGAAGTCTTTCCAGAATAATCCTGTTTCCAAGGTGATGCCATATCTTGGTAATAAGG 960
cDNA(MLOC_11124)     TAATTCTAATAACTGGTACAGAGGGAAGTCTTTCCAGAATAATCCTGTTTCCAAGGTGATGCCATATCTTGGTAATAAGG 960
CircularRNA          -------------------------------------------------------------------------------- -

                                  980               1.000               1.020               1.040
Genomic(MLOC_11124)  GACCTTTAGTTAACGAGCCAGGAGCAAAATTTGGCAGGGATAAGTGGCTCACTGGAGTCCCTGTAAGGCCTTTAGTTGAG 1040
cDNA(MLOC_11124)     GACCTTTAGTTAACGAGCCAGGAGCAAAATTTGGCAGGGATAAGTGGCTCACTGGAGTCCCTGTAAGGCCTTTAGTTGAG 1040
CircularRNA          -------------------------------------------------------------------------------- -

                                1.060               1.080               1.100               1.120
Genomic(MLOC_11124)  CATACCGGTTTTGATAGCATTTCTGCCGTTAGTTTCAGTTCAATAAAGAAGAAAAAAGAAACAACCAAACCATCAGATTT 1120
cDNA(MLOC_11124)     CATACCGGTTTTGATAGCATTTCTGCCGTTAGTTTCAGTTCAATAAAGAAGAAAAAAGAAACAACCAAACCATCAGATTT 1120
CircularRNA          -------------------------------------------------------------------------------- -

                                1.140               1.160               1.180               1.200
Genomic(MLOC_11124)  CCATGATCCAGTAAGGGAGTCATTTGAGGCTGAGCTCGATAGGATCTTGAGGGTACAAGAGCAAGAAAGACAGCGGGTAG 1200
cDNA(MLOC_11124)     CCATGATCCAGTAAGGGAGTCATTTGAGGCTGAGCTCGATAGGATCTTGAGGGTACAAGAGCAAGAAAGACAGCGGGTAG 1200
CircularRNA          -------------------------------------------------------------------------------- -

                                1.220               1.240               1.260               1.280
Genomic(MLOC_11124)  TGGAAGAACAGGCCAGAGTCAGAGAAATCGCTAGGAAACAAGATGAGGAGAGGGAGAACCTGATAAGAGAGGAGGAGGAA 1280
cDNA(MLOC_11124)     TGGAAGAACAGGCCAGAGTCAGAGAAATCGCTAGGAAACAAGATGAGGAGAGGGAGAACCTGATAAGAGAGGAGGAGGAA 1280
CircularRNA          -------------------------------------------------------------------------------- -

                                1.300               1.320               1.340               1.360
Genomic(MLOC_11124)  AGGCGGCGGTTGGTGGAAGAAGAGGCAAGACAGGCTGTTTGGCAAGCCGAGCAAGAGACGCTGGAAGCTTCCAGAAGAGT 1360
cDNA(MLOC_11124)     AGGCGGCGGTTGGTGGAAGAAGAGGCAAGACAGGCTGTTTGGCAAGCCGAGCAAGAGACGCTGGAAGCTTCCAGAAGAGT 1360
CircularRNA          -------------------------------------------------------------------------------- -

                                1.380               1.400               1.420               1.440
Genomic(MLOC_11124)  TGAGGAGCAGAGAATTGCTAGGGAGGAAGAGAAGATGAGGGTTGCTATGGAGGAGGAGCGGCGTAGAGAAGCCGCACGTC 1440
cDNA(MLOC_11124)     TGAGGAGCAGAGAATTGCTAGGGAGGAAGAGAAGATGAGGGTTGCTATGGAGGAGGAGCGGCGTAGAGAAGCCGCACGTC 1440
CircularRNA          -------------------------------------------------------------------------------- -

                                1.460               1.480               1.500               1.520
Genomic(MLOC_11124)  AAAAGCTCCTGGAATTGGAGGCAAGGATTGCTAGACGGCGAGCCGAATCAAACATGAGCAATGGAAATCTTACTTCAGCT 1520
cDNA(MLOC_11124)     AAAAGCTCCTGGAATTGGAGGCAAGGATTGCTAGACGGCGAGCCGAATCAAACATGAGCAATGGAAATCTTACTTCAGCT 1520
CircularRNA          -------------------------------------------------------------------------------- -

                                1.540               1.560               1.580               1.600
Genomic(MLOC_11124)  GCCAATGATGAACAAAGACTTGGAGCCTTGAAAGACAGAGATGTGTCACGGTACACTAATGCTGGTGAAAGACATGCTAT 1600
cDNA(MLOC_11124)     GCCAATGATGAACAAAGACTTGGAGCCTTGAAAGACAGAGATGTGTCACGGTACACTAATGCTGGTGAAAGACATGCTAT 1600
CircularRNA          -------------------------------------------------------------------------------- -
```

```
                    1.620               1.640               1.660               1.680
Genomic(MLOC_11124) TAGCAGACTGGGTGAGCGCATCAATACCTCCATTTCATCGGTGGCTTCCAGTCTCAACCGGTACAGTGATACAGTTCCAA 1680
   cDNA(MLOC_11124) TAGCAGACTGGGTGAGCGCATCAATACCTCCATTTCATCGGTGGCTTCCAGTCTCAACCGGTACAGTGATACAGTTCCAA 1680
        CircularRNA -------------------------------------------------------------------------------- -

                    1.700               1.720               1.740               1.760
Genomic(MLOC_11124) GGGCGCTCAACATTATGGGAGATGGACACTCAGGCTTGGTTGATAGAGAACACGCATACCACAGTGCAAGGGCTGCATTT 1760
   cDNA(MLOC_11124) GGGCGCTCAACATTATGGGAGATGGACACTCAGGCTTGGTTGATAGAGAACACGCATACCACAGTGCAAGGGCTGCATTT 1760
        CircularRNA -------------------------------------------------------------------------------- -

                    1.780               1.800               1.820               1.840
Genomic(MLOC_11124) GAAGACCAAGAAAATATACATTACAGCCCACGGCGTGGCACTTTGGGTACTAAGAGGGAAAGTTTCCCTAAAAAAGATTC 1840
   cDNA(MLOC_11124) GAAGACCAAGAAAATATACATTACAGCCCACGGCGTGGCACTTTGGGTACTAAGAGGGAAAGTTTCCCTAAAAAAGATTC 1840
        CircularRNA -------------------------------------------------------------------------------- -

                    1.860               1.880               1.900               1.920
Genomic(MLOC_11124) TTATGTTGGATTTCGGGCATCGTCAGTCGGGCCATCTTCGAGAGACCAAATCAATGATTCACCATGGGCACTGGAAGATT 1920
   cDNA(MLOC_11124) TTATGTTGGATTTCGGGCATCGTCAGTCGGGCCATCTTCGAGAGACCAAATCAATGATTCACCATGGGCACTGGAAGATT 1920
        CircularRNA -------------------------------------------------------------------------------- -

                    1.940               1.960               1.980               2.000
Genomic(MLOC_11124) ACAGTCAAGGAAGAGTTTCAAGGTGGGATGCACCGACGGAGAATAACTGTTTTGACAAGCAGTCTGAATTTGACACTCAT 2000
   cDNA(MLOC_11124) ACAGTCAAGGAAGAGTTTCAAGGTGGGATGCACCGACGGAGAATAACTGTTTTGACAAGCAGTCTGAATTTGACACTCAT 2000
        CircularRNA -------------------------------------------------------------------------------- -

                    2.020               2.040               2.060               2.080
Genomic(MLOC_11124) TTTTTTAACAGTGATAGGTTTGGAGATGCTGCATGGCTGCCTAGTGGTTCCTACAGAAGCCCCAATGCTCAACAAGGAGG 2080
   cDNA(MLOC_11124) TTTTTTAACAGTGATAGGTTTGGAGATGCTGCATGGCTGCCTAGTGGTTCCTACAGAAGCCCCAATGCTCAACAAGGAGG 2080
        CircularRNA -------------------------------------------------------------------------------- -

                    2.100               2.120               2.140               2.160
Genomic(MLOC_11124) GAGAATGTTCCAGAACTCCGAGGCTCATGATTTCTCGTCTTCTACAAAATCCTGCTATCCTATGCGGCATCCATGTGTAC 2160
   cDNA(MLOC_11124) GAGAATGTTCCAGAACTCCGAGGCTCATGATTTCTCGTCTTCTACAAAATCCTGCTATCCTATGCGGCATCCATGTGTAC 2160
        CircularRNA -------------------------------------------------------------------------------- -

                    2.180               2.200               2.220               2.240
Genomic(MLOC_11124) CCCCACCACATGTTGTGACCTCAATGCATGGAAGTGCAGTTAGTTCTTCTATTCAACGTGCCAATTCATCTTTCATTCAT 2240
   cDNA(MLOC_11124) CCCCACCACATGTTGTGACCTCAATGCATGGAAGTGCAGTTAGTTCTTCTATTCAACGTGCCAATTCATCTTTCATTCAT 2240
        CircularRNA -------------------------------------------------------------------------------- -

                    2.260               2.280               2.300               2.320
Genomic(MLOC_11124) GATTTAATGAGAGAGAGTTCTAGTAGAGATGATGAACAAACTATGCACAGTCAATATGGTAGTGCATACCAAGAAGTATC 2320
   cDNA(MLOC_11124) GATTTAATGAGAGAGAGTTCTAGTAGAGATGATGAACAAACTATGCACAGTCAATATGGTAGTGCATACCAAGAAGTATC 2320
        CircularRNA -------------------------------------------------------------------------------- -

                    2.340               2.360               2.380               2.400
Genomic(MLOC_11124) CCGCCAGCATCGGACGCCTGCTGAAGGCATTGTTGTCAATGAGCAGCAAAATGGGGACAGGGCGAGCCCTGTTTTGGGCT 2400
   cDNA(MLOC_11124) CCGCCAGCATCGGACGCCTGCTGAAGGCATTGTTGTCAATGAGCAGCAAAATGGGGACAGGGCGAGCCCTGTTTTGGGCT 2400
        CircularRNA -------------------------------------------------------------------------------- -

                    2.420               2.440               2.460               2.480
Genomic(MLOC_11124) CACAATCTTCTCTTTCCGTTTCAAGCCCTCCTAGGTCACCTGAACATGTTTCACATGACGAGATGGATGTTTCTTGTGAT 2480
   cDNA(MLOC_11124) CACAATCTTCTCTTTCCGTTTCAAGCCCTCCTAGGTCACCTGAACATGTTTCACATGACGAGATGGATGTTTCTTGTGAT 2480
        CircularRNA -------------------------------------------------------------------------------- -

                    2.500               2.520               2.540               2.560
Genomic(MLOC_11124) TCACCTGCACTGCCAACTTCTGCTGATGGTGATCGTACTGTGGTATCTGATAATGACCAAGTGGCTTCAACTTTAGATGC 2560
   cDNA(MLOC_11124) TCACCTGCACTGCCAACTTCTGCTGATGGTGATCGTACTGTGGTATCTGATAATGACCAAGTGGCTTCAACTTTAGATGC 2560
        CircularRNA -------------------------------------------------------------------------------- -

                    2.580               2.600               2.620               2.640
Genomic(MLOC_11124) AGCAAACATAAGCAGAATCACTACCTCAAGCGCGGCTTATCACATGGAGGATGAGGAATGGCCAAGTGAACACAATGAAT 2640
   cDNA(MLOC_11124) AGCAAACATAAGCAGAATCACTACCTCAAGCGCGGCTTATCACATGGAGGATGAGGAATGGCCAAGTGAACACAATGAAT 2640
        CircularRNA -------------------------------------------------------CGACGCCCAAGTGAACACAATGAAT 25

                    2.660               2.680               2.700               2.720
Genomic(MLOC_11124) CCAAGCGAAAACAGGATGAGTATGATGAGGAAGGCAATAGCTACCAAGAAGATGAAATCAATGATGGCGAGGGTGATACT 2720
   cDNA(MLOC_11124) CCAAGCGAAAACAGGATGAGTATGATGAGGAAGGCAATAGCTACCAAGAAGATGAAATCAATGATGGCGAGGGTGATACT 2720
        CircularRNA CCAAGCGAAAACAGGATGAGTATGATGAGGAAGGCAATAGCTACCAAGAAGATGAAATCAATGATGGCGAGGGTGATACT 105

                    2.740               2.760               2.780               2.800
Genomic(MLOC_11124) CTTGACTTGGCTAATGAGTTCACAGATGTGCATCTTGACTTGGATGATGAGTTTGCAGATGAAGATAATACAACTGCAGA 2800
   cDNA(MLOC_11124) CTTGACTTGGCTAATGAGTTCACAGATGTGCATCTTGACTTGGATGATGAGTTTGCAGATGAAGATAATACAACTGCAGA 2800
        CircularRNA CTTGACTTGGCTAATGAGTTCACAGATGTGCATCTTGACTTGGATGATGAGTTTGCAGATGAAGATAATACAACTGCAGA 185

                    2.820               2.840               2.860               2.880
Genomic(MLOC_11124) AATGGAACCAGTTATACTTGGATTCGATCAGGGTGTACAGGTTGAAATTCCCGTGAATAATGAGCTTGAATTAAGTTCTG 2880
   cDNA(MLOC_11124) AATGGAACCAGTTATACTTGGATTCGATCAGGGTGTACAGGTTGAAATTCCCGTGAATAATGAGCTTGAATTAAGTTCTG 2880
        CircularRNA AATGGAACCAGTTATACTTGGATTCGATCAGGGTGTACAGGTTGAAATTCCCGTGAATAATGAGCTTGAATTAAGTTCTG 265

                    2.900               2.920               2.940               2.960
Genomic(MLOC_11124) TGAAGAGCACTGAACTGGAAGTTGGTGTACACTTGGGCGTCGTGAAGCAAGAGTTGAGATGTGGTTCAGTTGATCCTTAT 2960
   cDNA(MLOC_11124) TGAAGAGCACTGAACTGGAAGTTGGTGTACACTTGGGCGTCGTGAAGCAAGAGTTGAGATGTGGTTCAGTTGATCCTTAT 2960
        CircularRNA TGAAGAGCACTGAACTGGAAGTTGGTGTACACTTGGGCGTCGTGAAGCAAGAGTTGAGATGTGGTTCA------------ 333

                    2.980               3.000               3.020               3.040
Genomic(MLOC_11124) GACATTGTTACGCTTCAAGGCCTTGATCAAACAAATGCCTTGGCCGATGAATCTAATGTTGATCCATCCGGTAGCACAGC 3040
   cDNA(MLOC_11124) GACATTGTTACGCTTCAAGGCCTTGATCAAACAAATGCCTTGGCCGATGAATCTAATGTTGATCCATCCGGTAGCACAGC 3040
        CircularRNA -------------------------------------------------------------------------------- 333

                    3.060               3.080               3.100               3.120
Genomic(MLOC_11124) TGTGTCAAGTTCCAAGTTACCTCAGGCATCTTTTGCCCCCCCTATTGATTCATCAACATCAGCAGTAATTGACCAGAACG 3120
   cDNA(MLOC_11124) TGTGTCAAGTTCCAAGTTACCTCAGGCATCTTTTGCCCCCCCTATTGATTCATCAACATCAGCAGTAATTGACCAGAACG 3120
        CircularRNA -------------------------------------------------------------------------------- 333

                    3.140               3.160               3.180               3.200
Genomic(MLOC_11124) AAGTTCCTGTTAGTCTGGGTTTGTTTTCTGGGCCATCTTTAATACCAACTCCGATTCGAGCCATTCAGATTGGCTCCATA 3200
   cDNA(MLOC_11124) AAGTTCCTGTTAGTCTGGGTTTGTTTTCTGGGCCATCTTTAATACCAACTCCGATTCGAGCCATTCAGATTGGCTCCATA 3200
        CircularRNA -------------------------------------------------------------------------------- 333
```

```
                      3.220               3.240               3.260               3.280
Genomic(MLOC_11124)   CAGATGCCGATCCATCTCCACAATCAGATTAACCCATCCCTGGCTCAACTGCTCCCTTCATCAGCTCCTTTATTCAAGTT 3280
cDNA(MLOC_11124)      CAGATGCCGATCCATCTCCACAATCAGATTAACCCATCCCTGGCTCAACTGCTCCCTTCATCAGCTCCTTTATTCAAGTT 3280
CircularRNA           -------------------------------------------------------------------------------- 333

                      3.300               3.320               3.340               3.360
Genomic(MLOC_11124)   TGGTCAGTTGAGGCATGTCCGCCCTGTTGCCCAGAATGTTCGACAACATTCTCAGGCGGTGCCCTCCATTCAGCCCCCCG 3360
cDNA(MLOC_11124)      TGGTCAGTTGAGGCATGTCCGCCCTGTTGCCCAGAATGTTCGACAACATTCTCAGGCGGTGCCCTCCATTCAGCCCCCCG 3360
CircularRNA           -------------------------------------------------------------------------------- 333

                      3.380               3.400               3.420               3.440
Genomic(MLOC_11124)   CACCAACTCTACATATATCGAAACAGAATGGTTCGAGTGGTATACCTAATGAGATGGATCGAAATGCAAACCAGATTACC 3440
cDNA(MLOC_11124)      CACCAACTCTACATATATCGAAACAGAATGGTTCGAGTGGTATACCTAATGAGATGGATCGAAATGCAAACCAGATTACC 3440
CircularRNA           -------------------------------------------------------------------------------- 333

                      3.460               3.480               3.500               3.520
Genomic(MLOC_11124)   CCAAGAGAATCAAATTTGCATCAGCGCAATGAATCTGAAATCAACTGGATGGCTGATTTAAATGAATTTCAGAGCCGATT 3520
cDNA(MLOC_11124)      CCAAGAGAATCAAATTTGCATCAGCGCAATGAATCTGAAATCAACTGGATGGCTGATTTAAATGAATTTCAGAGCCGATT 3520
CircularRNA           -------------------------------------------------------------------------------- 333

                      3.540               3.560               3.580               3.600
Genomic(MLOC_11124)   AGACAGAACTTCAATTGGAGAAAATGCATCTTTCAGACTTTCAAAAGGTGATTCTCAGAGGAATAATGATATCTCTTCAA 3600
cDNA(MLOC_11124)      AGACAGAACTTCAATTGGAGAAAATGCATCTTTCAGACTTTCAAAAGGTGATTCTCAGAGGAATAATGATATCTCTTCAA 3600
CircularRNA           -------------------------------------------------------------------------------- 333

                      3.620               3.640               3.660               3.680
Genomic(MLOC_11124)   AGCGGAATCACAAATCTTCTTTCAGTAACACAGAATCTTCTCAAGTTGGTTCATATGGGAAAGCCTTGAGTGGCATAAAG 3680
cDNA(MLOC_11124)      AGCGGAATCACAAATCTTCTTTCAGTAACACAGAATCTTCTCAAGTTGGTTCATATGGGAAAGCCTTGAGTGGCATAAAG 3680
CircularRNA           -------------------------------------------------------------------------------- 333

                      3.700               3.720               3.740               3.760
Genomic(MLOC_11124)   GCTCCAGGCGCTGTATCTGGTGGAAGTGGGAGGAGATACGATTATGCTGTTAAAGAATCTAATATGGGATCAACAGGCTC 3760
cDNA(MLOC_11124)      GCTCCAGGCGCTGTATCTGGTGGAAGTGGGAGGAGATACGATTATGCTGTTAAAGAATCTAATATGGGATCAACAGGCTC 3760
CircularRNA           -------------------------------------------------------------------------------- 333

                      3.780               3.800               3.820               3.840
Genomic(MLOC_11124)   ACCTGTTGAACCTTTTCATAAAGACTCCAGAGGATTCCAGAGAAGGTCTCGTAGGAACATAAGAAGAACTGAATTTAGAG 3840
cDNA(MLOC_11124)      ACCTGTTGAACCTTTTCATAAAGACTCCAGAGGATTCCAGAGAAGGTCTCGTAGGAACATAAGAAGAACTGAATTTAGAG 3840
CircularRNA           -------------------------------------------------------------------------------- 333

                      3.860               3.880               3.900               3.920
Genomic(MLOC_11124)   TGCGGGCAAATGTTGAGAAGAACGAAACCCAAGCTTCTGAGTGCCATGATGAGCAGAATGAGAATCCGGTTCCCAATGGA 3920
cDNA(MLOC_11124)      TGCGGGCAAATGTTGAGAAGAACGAAACCCAAGCTTCTGAGTGCCATGATGAGCAGAATGAGAATCCGGTTCCCAATGGA 3920
CircularRNA           -------------------------------------------------------------------------------- 333

                      3.940               3.960               3.980               4.000
Genomic(MLOC_11124)   TTGGCAAGGGAGATTCCGGTGAGAAATGTGAACAGAAAGGAAGGTACAAATGAAACAATTGATATTAATGGAGCAGATTC 4000
cDNA(MLOC_11124)      TTGGCAAGGGAGATTCCGGTGAGAAATGTGAACAGAAAGGAAGGTACAAATGAAACAATTGATATTAATGGAGCAGATTC 4000
CircularRNA           -------------------------------------------------------------------------------- 333

                      4.020               4.040               4.060               4.080
Genomic(MLOC_11124)   TTCATCTACATCTGCTCATTATTATAGTAAAACAGAGAGACTTGCACAGAAGGCTCCATCTTATGACAGGTCTCGTTGTG 4080
cDNA(MLOC_11124)      TTCATCTACATCTGCTCATTATTATAGTAAAACAGAGAGACTTGCACAGAAGGCTCCATCTTATGACAGGTCTCGTTGTG 4080
CircularRNA           -------------------------------------------------------------------------------- 333

                      4.100               4.120               4.140               4.160
Genomic(MLOC_11124)   GGTACAAGAAATCTAGAGCAGGTGGTATCCCCGAGGGAGATGCTAATACCTTGTTACGTGCTGGAGTTGTACGCATCGTT 4160
cDNA(MLOC_11124)      GGTACAAGAAATCTAGAGCAGGTGGTATCCCCGAGGGAGATGCTAATACCTTGTTACGTGCTGGAGTTGTACGCATCGTT 4160
CircularRNA           -------------------------------------------------------------------------------- 333

                      4.180               4.200               4.220               4.240
Genomic(MLOC_11124)   AAGCAGCAAGGCTTTGAAATGCCTGTTGATGCAGATGGTTTTATTGAAGTCAGGTCCAAGAAGCAGATCATGAGTGTCAG 4240
cDNA(MLOC_11124)      AAGCAGCAAGGCTTTGAAATGCCTGTTGATGCAGATGGTTTTATTGAAGTCAGGTCCAAGAAGCAGATCATGAGTGTCAG 4240
CircularRNA           -------------------------------------------------------------------------------- 333

                      4.260               4.280               4.300               4.320
Genomic(MLOC_11124)   GAGAGAGCAGAGGGAGAAAGAAAATAGATTGAAAATAAGGACGGCAAAGGTGCCAAGTTATCATCCTTTACATTGGTGCC 4320
cDNA(MLOC_11124)      GAGAGAGCAGAGGGAGAAAGAAAATAGATTGAAAATAAGGACGGCAAAGG------------------------------ 4290
CircularRNA           -------------------------------------------------------------------------------- 333

                      4.340               4.360               4.380               4.400
Genomic(MLOC_11124)   TTGTATGTATTTGTCTTCCTAAAATTAATGCTTTCGTGTTTGTTGAACCAGGCTCCCCGCAAGCAGCATCAAATGTCGCT 4400
cDNA(MLOC_11124)      ----------------------------------------------------CTCCCCGCAAGCAGCATCAAATGTCGCT 4318
CircularRNA           -------------------------------------------------------------------------------- 333

                      4.420               4.440               4.460               4.480
Genomic(MLOC_11124)   ACACAGTTCTAACAGTTCAAGTATTTGTAAGGGGACAGTTTCTTTGGATGGAGAACCTGCGAAGAAAGTTTCTTTGGGTT 4480
cDNA(MLOC_11124)      ACACAGTTCTAACAGTTCAAGTATTTGTAAGGGGACAGTTTCTTTGGATGGAGAACCTGCGAAGAAAGTTTCTTTGGGTT 4398
CircularRNA           -------------------------------------------------------------------------------- 333

                      4.500               4.520               4.540               4.560
Genomic(MLOC_11124)   CTGTCCTTGCAGTTGAAGGAAGGGTCCTTGACCACGCCGAACCATCATCTTCATTCATGAATGATACAGCTTCAATGACA 4560
cDNA(MLOC_11124)      CTGTCCTTGCAGTTGAAGGAAGGGTCCTTGACCACGCCGAACCATCATCTTCATTCATGAATGATACAGCTTCAATGACA 4478
CircularRNA           -------------------------------------------------------------------------------- 333

                      4.580               4.600               4.620               4.640
Genomic(MLOC_11124)   CCCATAGGGCGGCCACCTTCAGCCAACACAGGACCTCGTACAAACTACTGTGCAATGAAGTATGTAGCACTGTATCTTGG 4640
cDNA(MLOC_11124)      CCCATAGGGCGGCCACCTTCAGCCAACACAGGACCTCGTACAAACTACTGTGCAATGAAG-------------------- 4538
CircularRNA           -------------------------------------------------------------------------------- 333

                      4.660               4.680               4.700               4.720
Genomic(MLOC_11124)   TTATGTTTATGCTATTTAACTAGGATTCTTGTTTATAACCTTGTTCAAAAATTCTTTTGCAGGCCTATCGGAAGCCAGTC 4720
cDNA(MLOC_11124)      ---------------------------------------------------------------CCTATCGGAAGCCAGTC 4555
CircularRNA           -------------------------------------------------------------------------------- 333

                      4.740               4.760               4.780               4.800
Genomic(MLOC_11124)   AACCTCTGACTTGATAACTTCTATTGCCGCAACAAAGCTTGCGGCATGCTTCTCAGAAAGCAATAATAAAACATCGCCAA 4800
cDNA(MLOC_11124)      AACCTCTGACTTGATAACTTCTATTGCCGCAACAAAGCTTGCGGCATGCTTCTCAGAAAGCAATAATAAAACATCGCCAA 4635
CircularRNA           -------------------------------------------------------------------------------- 333
```

```
                                                4.820               4.840
                                                  |                   |
Genomic(MLOC_11124)  TTGGCACTCCATTTAACATGGGCAACTGGGATAGTTCACAAACGAACCAGCAGGTAG  4857
cDNA(MLOC_11124)     TTGGCACTCCATTTAACATGGGCAACTGGGATAGTTCACAAACGAACCAGCAGGTAG  4692
CircularRNA          ---------------------------------------------------------  333
```

RNA-binding (RRM/RBD/RNP motifs) protein_circular RNA

(ID: Ch4:252670767-252671078)

GTTACAGTCCGAGCCCGGTAGGTGGTTATGGAATGAGGATGGATGCAAGGTTTGGGCTTCTAT
CAGGCGGCCGTAGTAGTTATCCTTCTTTTGGTGGTGGTTATGGAATCGGTATGAACTTTGACCC
AGGCATGAACCCAGGTATTGGCGGTAGCTCGAACTTCAACAATAGTGTCCAGTATGGACGGCAG
ATCAATCCATACTACAGTGGTAATTCGGGTAGATACAATAGCAACA**TTAGCTATGGTGGAGTCAA**
CGACAATTCTGGGTCAGTATTCAACTCACTGGCTCGTAATCTGTGGGGTAATTCAG

The nucleotides of junction-region are underlined. The nucleotides of junction-region which are supported by the junction-spaning sequencing reads are shown in red. Introns are not shown if the absence is supported by sequencing reads. In the absence of supporting sequencing reads, the intronic nucletides are shown as N.

Structural relationship between the circular RNA and its parental gene

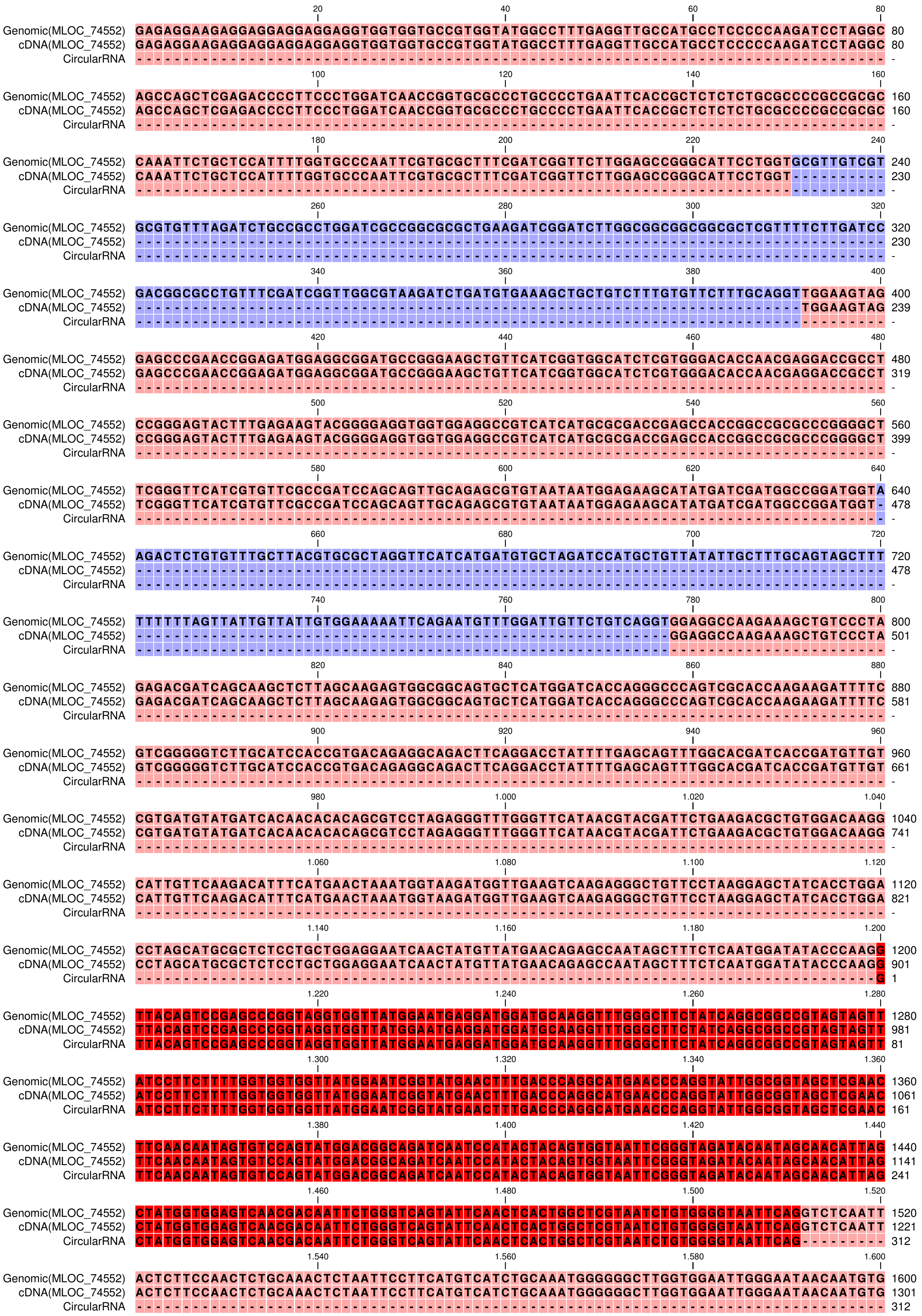

Genomic(MLOC_74552)
cDNA(MLOC_74552)
CircularRNA

```
                    1.620 1.640 1.660 1.680
Genomic(MLOC_74552) AACTGGGGAACCCCTTCTGTGCCTGCTCAAGGTGCTAACGCTGGCTCGGGCTATGGCAGTGGGAACTTTGGTTATGGATC 1680
cDNA(MLOC_74552)    AACTGGGGAACCCCTTCTGTGCCTGCTCAAGGTGCTAACGCTGGCTCGGGCTATGGCAGTGGGAACTTTGGTTATGGATC 1381
CircularRNA         -------------------------------------------------------------------------------- 312

                    1.700 1.720 1.740 1.760
Genomic(MLOC_74552) GACTGAAAACAACTTCAATCTGAGTCCCGGTGCTTATGGAAGGAACACTGGATCAGGTGGCGTCAATGCTTCACTCAACC 1760
cDNA(MLOC_74552)    GACTGAAAACAACTTCAATCTGAGTCCCGGTGCTTATGGAAGGAACACTGGATCAGGTGGCGTCAATGCTTCACTCAACC 1461
CircularRNA         -------------------------------------------------------------------------------- 312

                    1.780 1.800 1.820 1.840
Genomic(MLOC_74552) AGTCAAGCAATGGATATGGGAGGAACTTTGGAGATTCGTCAGCGGCTGGTGGCGGCTCCATATATGGAGACACAACTTGG 1840
cDNA(MLOC_74552)    AGTCAAGCAATGGATATGGGAGGAACTTTGGAGATTCGTCAGCGGCTGGTGGCGGCTCCATATATGGAGACACAACTTGG 1541
CircularRNA         -------------------------------------------------------------------------------- 312

                    1.860 1.880 1.900 1.920
Genomic(MLOC_74552) AGATCTGGATCCGAGCTTGATGGAACCAGCCCATTTGGATATGGGCTGGGGAATGCAGCTTCAGATGTTACAGCAAAGAG 1920
cDNA(MLOC_74552)    AGATCTGGATCCGAGCTTGATGGAACCAGCCCATTTGGATATGGGCTGGGGAATGCAGCTTCAGATGTTACAGCAAAGAG 1621
CircularRNA         -------------------------------------------------------------------------------- 312

                    1.940 1.960 1.980 2.000
Genomic(MLOC_74552) CTCAGCGGGTTACATGGGACATTAACAGATAGAGGTTAGATATTTTCTTCTTTGTATTATCCTTGTGCTTTTCTTTTTTA 2000
cDNA(MLOC_74552)    CTCAGCGGGTTACATGGGACATTAACAGATAGAGG--------------------------------------------- 1656
CircularRNA         -------------------------------------------------------------------------------- 312

                    2.020 2.040 2.060 2.080
Genomic(MLOC_74552) TAGTTTGCAAATGTTGAAGTACTAGTTCATGTACTAGCAGCATCAGATAAAGACCATCAAATTTGAAAGTTTGCACTGAT 2080
cDNA(MLOC_74552)    -------------------------------------------------------------------------------- 1656
CircularRNA         -------------------------------------------------------------------------------- 312

                    2.100 2.120 2.140 2.160
Genomic(MLOC_74552) GGTACTAATGTTTAGACAATGTAGCTTCAAATATGTCGTATCCCCTGCGTCAAATTTCATCCCTAATGCTCAAGTTTCAT 2160
cDNA(MLOC_74552)    -------------------------------------------------------------------------------- 1656
CircularRNA         -------------------------------------------------------------------------------- 312

                    2.180 2.200 2.220 2.240
Genomic(MLOC_74552) ATACAAAATATATTCAGTTGCCTTGGTTCGCCCAGGATAATGCATAGATGTAGTTGGCACATTTGCATGTCCATGAAATC 2240
cDNA(MLOC_74552)    -------------------------------------------------------------------------------- 1656
CircularRNA         -------------------------------------------------------------------------------- 312

                    2.260 2.280 2.300 2.320
Genomic(MLOC_74552) TAGGCAGGCATCGATCATTTGAACCTAGTAGCACCATGATACAGAACTTACTGTCATTATCAACTAAAAAGATGTCTTTA 2320
cDNA(MLOC_74552)    -------------------------------------------------------------------------------- 1656
CircularRNA         -------------------------------------------------------------------------------- 312

                    2.340 2.360 2.380 2.400
Genomic(MLOC_74552) GCATCCATTAAAATTGTTATCTAGTGTCTATCTACTTCATGTCCCAATTGTTACATTAGGCTGATTTTGATGATTCATGA 2400
cDNA(MLOC_74552)    -------------------------------------------------------------------------------- 1656
CircularRNA         -------------------------------------------------------------------------------- 312

                    2.420 2.440 2.460 2.480
Genomic(MLOC_74552) TTGTATATGATGGATATTGCAAATTGTCTGTTCTTCGTGTTCCAGTTGTTACGTGAAAATGATTTTAATGGTTCATGGTT 2480
cDNA(MLOC_74552)    -------------------------------------------------------------------------------- 1656
CircularRNA         -------------------------------------------------------------------------------- 312

                    2.500 2.520 2.540 2.560
Genomic(MLOC_74552) GTCTATGATGGATATTGCAAATTGTTATGAAATGAACACATAAGTTGATTGGATTCGAAAACTTTTGCCATCTTATGGTA 2560
cDNA(MLOC_74552)    -------------------------------------------------------------------------------- 1656
CircularRNA         -------------------------------------------------------------------------------- 312

                    2.580 2.600 2.620 2.640
Genomic(MLOC_74552) TGGATTCATTTTGAACATGCACCTATGAAGATGGTATTAAAGTAGTATGATAAGCTTGCAGTATTAACAAGAGCTAAATC 2640
cDNA(MLOC_74552)    -------------------------------------------------------------------------------- 1656
CircularRNA         -------------------------------------------------------------------------------- 312

                    2.660 2.680 2.700 2.720
Genomic(MLOC_74552) CATTGTTGCCCTCTTTTTCTTTGTGCTACTGTGAACTGTAATATTGTCTAGTCCTAGAAACTTGATTACTGTTTTTCACA 2720
cDNA(MLOC_74552)    -------------------------------------------------------------------------------- 1656
CircularRNA         -------------------------------------------------------------------------------- 312

                    2.740 2.760 2.780 2.800
Genomic(MLOC_74552) TTCAAACATTTCTCATCTGTGAACTTAACCTACACCTCAAATTATTTAATCCACATTGAAATATTGGTCATGCATGAACT 2800
cDNA(MLOC_74552)    -------------------------------------------------------------------------------- 1656
CircularRNA         -------------------------------------------------------------------------------- 312

                    2.820 2.840 2.860 2.880
Genomic(MLOC_74552) GCAGCCATGAGACCATATGAAAGAATTGATGCACCTCATATTTACTTAAACGAAACATATTAGTCCAGTAATACATTCAA 2880
cDNA(MLOC_74552)    -------------------------------------------------------------------------------- 1656
CircularRNA         -------------------------------------------------------------------------------- 312

                    2.900 2.920 2.940 2.960
Genomic(MLOC_74552) CAAGGTGTACGAAAGAATTTATAAGTCGTTTTTTTTTTCAATAGACCAATCTTTGCTTCTTTATATGACTTAAGCTAAAA 2960
cDNA(MLOC_74552)    -------------------------------------------------------------------------------- 1656
CircularRNA         -------------------------------------------------------------------------------- 312

                    2.980 3.000 3.020 3.040
Genomic(MLOC_74552) TATGGTCCAAGGACTTAATTTTAATAGCTGAACCAATTTATATTGTGTATTTCCAAATTTAATTGTTACTGTAATACTTA 3040
cDNA(MLOC_74552)    -------------------------------------------------------------------------------- 1656
CircularRNA         -------------------------------------------------------------------------------- 312

                    3.060 3.080 3.100 3.120
Genomic(MLOC_74552) TGCCATCCATTACCAAGGCATTATGTATAGTAAATCCATTTTGGCTTGCTCATACTATTCAAATATCAAATGATGTAGAT 3120
cDNA(MLOC_74552)    -------------------------------------------------------------------------------- 1656
CircularRNA         -------------------------------------------------------------------------------- 312

                    3.140 3.160 3.180 3.200
Genomic(MLOC_74552) TGTTAAGTATATGCTGGGTAGCATTTGGACAGTTCAAATCTGTGGACATCAATATTTGATTGTTCTGGATGACACTTAAC 3200
cDNA(MLOC_74552)    -------------------------------------------------------------------------------- 1656
CircularRNA         -------------------------------------------------------------------------------- 312
```

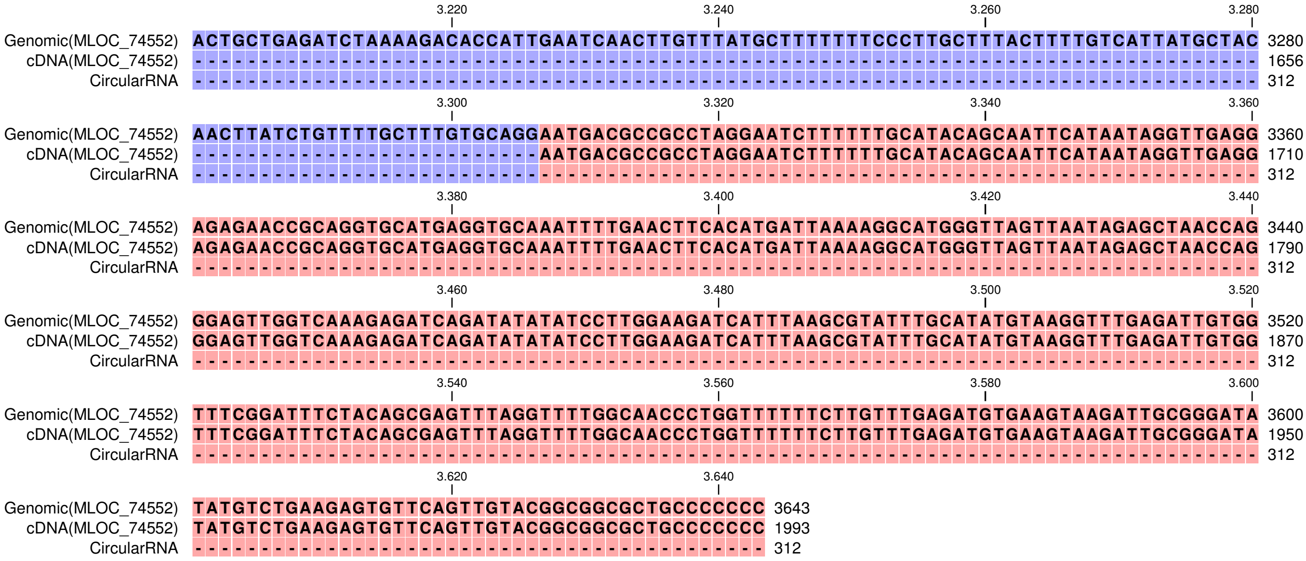
3.220 3.240 3.260 3.280
Genomic(MLOC_74552) ACTGCTGAGATCTAAAAGACACCATTGAATCAACTTGTTTATGCTTTTTTTCCCTTGCTTTACTTTTGTCATTATGCTAC 3280
cDNA(MLOC_74552) -- 1656
CircularRNA -- 312
3.300 3.320 3.340 3.360
Genomic(MLOC_74552) AACTTATCTGTTTTGCTTTGTGCAGGAATGACGCCGCCTAGGAATCTTTTTTGCATACAGCAATTCATAATAGGTTGAGG 3360
cDNA(MLOC_74552) --------------------------AATGACGCCGCCTAGGAATCTTTTTTGCATACAGCAATTCATAATAGGTTGAGG 1710
CircularRNA -- 312
3.380 3.400 3.420 3.440
Genomic(MLOC_74552) AGAGAACCGCAGGTGCATGAGGTGCAAATTTTGAACTTCACATGATTAAAAGGCATGGGTTAGTTAATAGAGCTAACCAG 3440
cDNA(MLOC_74552) AGAGAACCGCAGGTGCATGAGGTGCAAATTTTGAACTTCACATGATTAAAAGGCATGGGTTAGTTAATAGAGCTAACCAG 1790
CircularRNA -- 312
3.460 3.480 3.500 3.520
Genomic(MLOC_74552) GGAGTTGGTCAAAGAGATCAGATATATATCCTTGGAAGATCATTTAAGCGTATTTGCATATGTAAGGTTTGAGATTGTGG 3520
cDNA(MLOC_74552) GGAGTTGGTCAAAGAGATCAGATATATATCCTTGGAAGATCATTTAAGCGTATTTGCATATGTAAGGTTTGAGATTGTGG 1870
CircularRNA -- 312
3.540 3.560 3.580 3.600
Genomic(MLOC_74552) TTTCGGATTTCTACAGCGAGTTTAGGTTTTGGCAACCCTGGTTTTTTCTTGTTTGAGATGTGAAGTAAGATTGCGGGATA 3600
cDNA(MLOC_74552) TTTCGGATTTCTACAGCGAGTTTAGGTTTTGGCAACCCTGGTTTTTTCTTGTTTGAGATGTGAAGTAAGATTGCGGGATA 1950
CircularRNA -- 312
3.620 3.640
Genomic(MLOC_74552) TATGTCTGAAGAGTGTTCAGTTGTACGGCGGCGCTGCCCCCCC 3643
cDNA(MLOC_74552) TATGTCTGAAGAGTGTTCAGTTGTACGGCGGCGCTGCCCCCCC 1993
CircularRNA --- 312

RNA-binding (RRM/RBD/RNP motifs) protein_circular RNA

(ID: Ch6:308455217-308455370)

<u>**A**</u>**CTCGGCCACAGAAGAAGCTCAAAGTGCAGCCCAGAAGTGTTTTACACAAGTGAAGCAAAT**A
ATTCTAGTTTGTCATCACAGTACCGATAATGATGTACATTTTTTGGCGCAT**ATACAACAGTTGAT**
GTCTTTGGCGCATATACAACAGTTGA<u>**T**</u>

The nucleotides of junction-region are underlined. The nucleotides of junction-region which are supported by the junction-spaning sequencing reads are shown in red. Introns are not shown if the absence is supported by sequencing reads. In the absence of supporting sequencing reads, the intronic nucletides are shown as N.

Structural relationship between the circular RNA and its parental gene

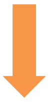

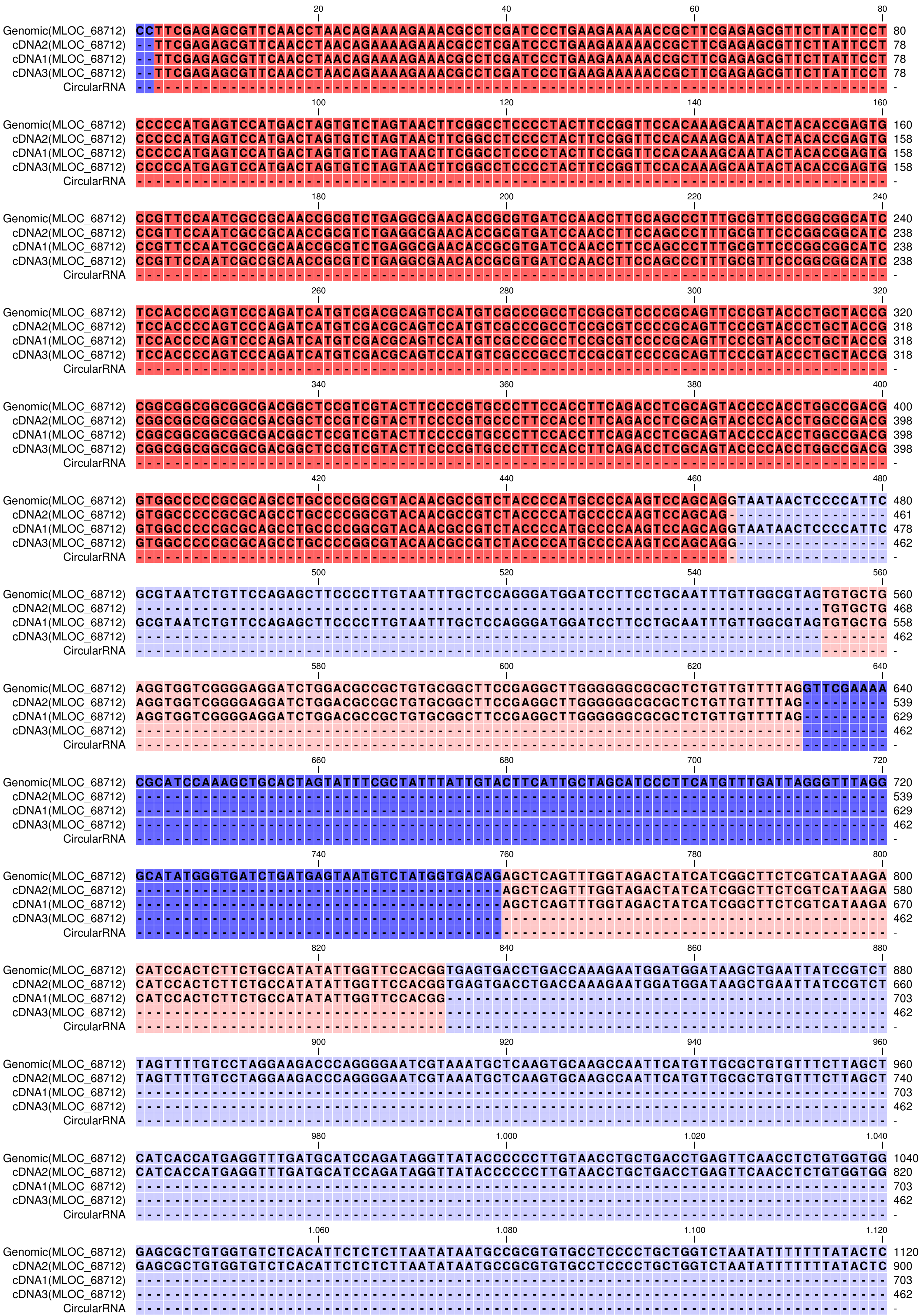
Genomic(MLOC_68712)
cDNA2(MLOC_68712)
cDNA1(MLOC_68712)
cDNA3(MLOC_68712)
CircularRNA

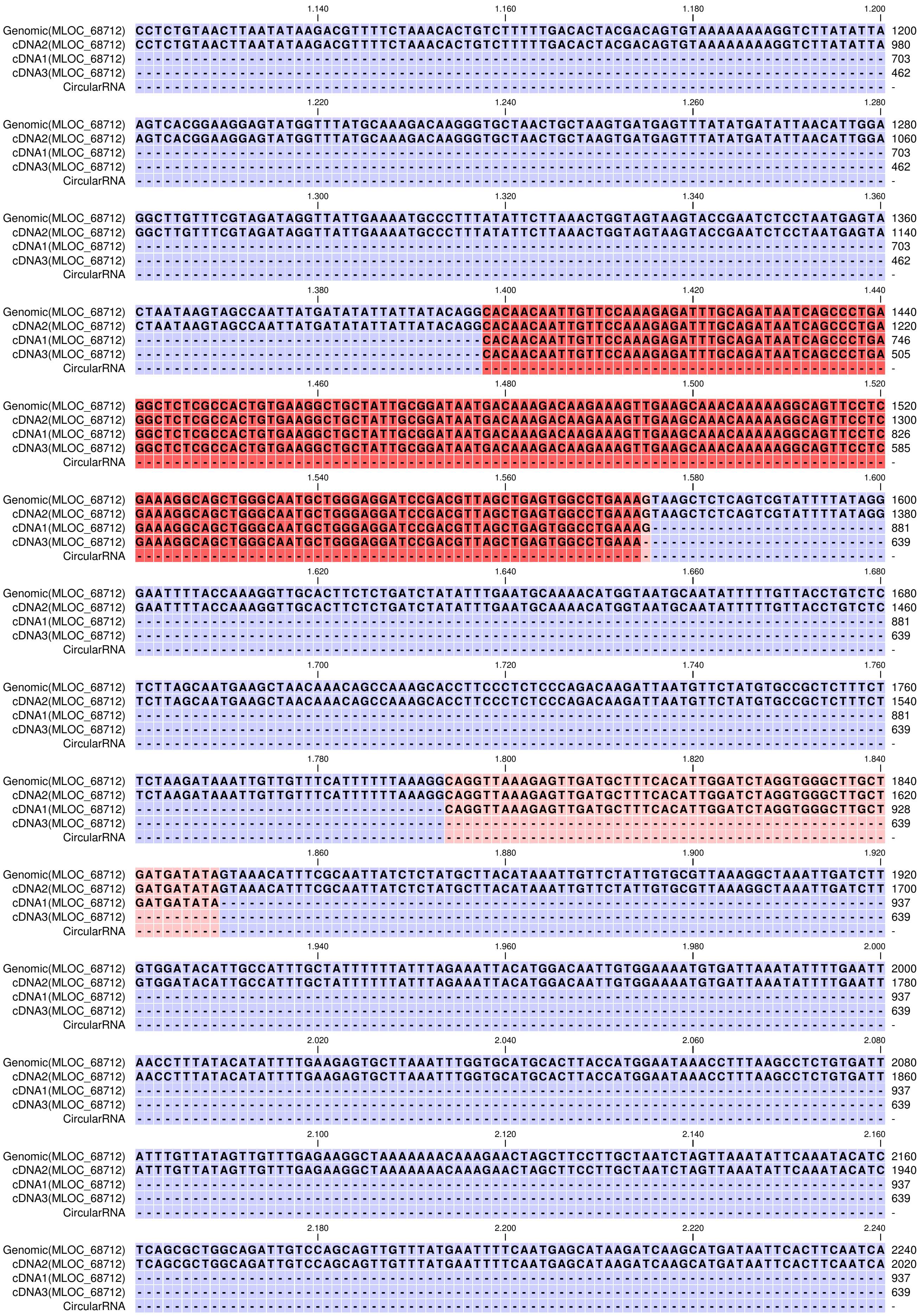

1.140 1.160 1.180 1.200
Genomic(MLOC_68712) CCTCTGTAACTTAATATAAGACGTTTTCTAAACACTGTCTTTTTGACACTACGACAGTGTAAAAAAAAGGTCTTATATTA 1200
cDNA2(MLOC_68712) CCTCTGTAACTTAATATAAGACGTTTTCTAAACACTGTCTTTTTGACACTACGACAGTGTAAAAAAAAGGTCTTATATTA 980
cDNA1(MLOC_68712) 703
cDNA3(MLOC_68712) 462
CircularRNA -
1.220 1.240 1.260 1.280
Genomic(MLOC_68712) AGTCACGGAAGGAGTATGGTTTATGCAAAGACAAGGGTGCTAACTGCTAAGTGATGAGTTTATATGATATTAACATTGGA 1280
cDNA2(MLOC_68712) AGTCACGGAAGGAGTATGGTTTATGCAAAGACAAGGGTGCTAACTGCTAAGTGATGAGTTTATATGATATTAACATTGGA 1060
cDNA1(MLOC_68712) 703
cDNA3(MLOC_68712) 462
CircularRNA -
1.300 1.320 1.340 1.360
Genomic(MLOC_68712) GGCTTGTTTCGTAGATAGGTTATTGAAAATGCCCTTTATATTCTTAAACTGGTAGTAAGTACCGAATCTCCTAATGAGTA 1360
cDNA2(MLOC_68712) GGCTTGTTTCGTAGATAGGTTATTGAAAATGCCCTTTATATTCTTAAACTGGTAGTAAGTACCGAATCTCCTAATGAGTA 1140
cDNA1(MLOC_68712) 703
cDNA3(MLOC_68712) 462
CircularRNA -
1.380 1.400 1.420 1.440
Genomic(MLOC_68712) CTAATAAGTAGCCAATTATGATATATTATTATACAGGCACAACAATTGTTCCAAAGAGATTTGCAGATAATCAGCCCTGA 1440
cDNA2(MLOC_68712) CTAATAAGTAGCCAATTATGATATATTATTATACAGGCACAACAATTGTTCCAAAGAGATTTGCAGATAATCAGCCCTGA 1220
cDNA1(MLOC_68712) CACAACAATTGTTCCAAAGAGATTTGCAGATAATCAGCCCTGA 746
cDNA3(MLOC_68712) CACAACAATTGTTCCAAAGAGATTTGCAGATAATCAGCCCTGA 505
CircularRNA -
1.460 1.480 1.500 1.520
Genomic(MLOC_68712) GGCTCTCGCCACTGTGAAGGCTGCTATTGCGGATAATGACAAAGACAAGAAAGTTGAAGCAAACAAAAAGGCAGTTCCTC 1520
cDNA2(MLOC_68712) GGCTCTCGCCACTGTGAAGGCTGCTATTGCGGATAATGACAAAGACAAGAAAGTTGAAGCAAACAAAAAGGCAGTTCCTC 1300
cDNA1(MLOC_68712) GGCTCTCGCCACTGTGAAGGCTGCTATTGCGGATAATGACAAAGACAAGAAAGTTGAAGCAAACAAAAAGGCAGTTCCTC 826
cDNA3(MLOC_68712) GGCTCTCGCCACTGTGAAGGCTGCTATTGCGGATAATGACAAAGACAAGAAAGTTGAAGCAAACAAAAAGGCAGTTCCTC 585
CircularRNA -
1.540 1.560 1.580 1.600
Genomic(MLOC_68712) GAAAGGCAGCTGGGCAATGCTGGGAGGATCCGACGTTAGCTGAGTGGCCTGAAAGTAAGCTCTCAGTCGTATTTTATAGG 1600
cDNA2(MLOC_68712) GAAAGGCAGCTGGGCAATGCTGGGAGGATCCGACGTTAGCTGAGTGGCCTGAAAGTAAGCTCTCAGTCGTATTTTATAGG 1380
cDNA1(MLOC_68712) GAAAGGCAGCTGGGCAATGCTGGGAGGATCCGACGTTAGCTGAGTGGCCTGAAAG 881
cDNA3(MLOC_68712) GAAAGGCAGCTGGGCAATGCTGGGAGGATCCGACGTTAGCTGAGTGGCCTGAAA 639
CircularRNA -
1.620 1.640 1.660 1.680
Genomic(MLOC_68712) GAATTTTACCAAAGGTTGCACTTCTCTGATCTATATTTGAATGCAAAACATGGTAATGCAATATTTTTGTTACCTGTCTC 1680
cDNA2(MLOC_68712) GAATTTTACCAAAGGTTGCACTTCTCTGATCTATATTTGAATGCAAAACATGGTAATGCAATATTTTTGTTACCTGTCTC 1460
cDNA1(MLOC_68712) 881
cDNA3(MLOC_68712) 639
CircularRNA -
1.700 1.720 1.740 1.760
Genomic(MLOC_68712) TCTTAGCAATGAAGCTAACAAACAGCCAAAGCACCTTCCCTCTCCCAGACAAGATTAATGTTCTATGTGCCGCTCTTTCT 1760
cDNA2(MLOC_68712) TCTTAGCAATGAAGCTAACAAACAGCCAAAGCACCTTCCCTCTCCCAGACAAGATTAATGTTCTATGTGCCGCTCTTTCT 1540
cDNA1(MLOC_68712) 881
cDNA3(MLOC_68712) 639
CircularRNA -
1.780 1.800 1.820 1.840
Genomic(MLOC_68712) TCTAAGATAAATTGTTGTTTCATTTTTTAAAGGCAGGTTAAAGAGTTGATGCTTTCACATTGGATCTAGGTGGGCTTGCT 1840
cDNA2(MLOC_68712) TCTAAGATAAATTGTTGTTTCATTTTTTAAAGGCAGGTTAAAGAGTTGATGCTTTCACATTGGATCTAGGTGGGCTTGCT 1620
cDNA1(MLOC_68712) CAGGTTAAAGAGTTGATGCTTTCACATTGGATCTAGGTGGGCTTGCT 928
cDNA3(MLOC_68712) 639
CircularRNA -
1.860 1.880 1.900 1.920
Genomic(MLOC_68712) GATGATATAGTAAACATTTCGCAATTATCTCTATGCTTACATAAATTGTTCTATTGTGCGTTAAAGGCTAAATTGATCTT 1920
cDNA2(MLOC_68712) GATGATATAGTAAACATTTCGCAATTATCTCTATGCTTACATAAATTGTTCTATTGTGCGTTAAAGGCTAAATTGATCTT 1700
cDNA1(MLOC_68712) GATGATATA 937
cDNA3(MLOC_68712) 639
CircularRNA -
1.940 1.960 1.980 2.000
Genomic(MLOC_68712) GTGGATACATTGCCATTTGCTATTTTTTATTTAGAAATTACATGGACAATTGTGGAAAATGTGATTAAATATTTTGAATT 2000
cDNA2(MLOC_68712) GTGGATACATTGCCATTTGCTATTTTTTATTTAGAAATTACATGGACAATTGTGGAAAATGTGATTAAATATTTTGAATT 1780
cDNA1(MLOC_68712) 937
cDNA3(MLOC_68712) 639
CircularRNA -
2.020 2.040 2.060 2.080
Genomic(MLOC_68712) AACCTTTATACATATTTTGAAGAGTGCTTAAATTTGGTGCATGCACTTACCATGGAATAAACCTTTAAGCCTCTGTGATT 2080
cDNA2(MLOC_68712) AACCTTTATACATATTTTGAAGAGTGCTTAAATTTGGTGCATGCACTTACCATGGAATAAACCTTTAAGCCTCTGTGATT 1860
cDNA1(MLOC_68712) 937
cDNA3(MLOC_68712) 639
CircularRNA -
2.100 2.120 2.140 2.160
Genomic(MLOC_68712) ATTTGTTATAGTTGTTTGAGAAGGCTAAAAAAACAAAGAACTAGCTTCCTTGCTAATCTAGTTAAATATTCAAATACATC 2160
cDNA2(MLOC_68712) ATTTGTTATAGTTGTTTGAGAAGGCTAAAAAAACAAAGAACTAGCTTCCTTGCTAATCTAGTTAAATATTCAAATACATC 1940
cDNA1(MLOC_68712) 937
cDNA3(MLOC_68712) 639
CircularRNA -
2.180 2.200 2.220 2.240
Genomic(MLOC_68712) TCAGCGCTGGCAGATTGTCCAGCAGTTGTTTATGAATTTTCAATGAGCATAAGATCAAGCATGATAATTCACTTCAATCA 2240
cDNA2(MLOC_68712) TCAGCGCTGGCAGATTGTCCAGCAGTTGTTTATGAATTTTCAATGAGCATAAGATCAAGCATGATAATTCACTTCAATCA 2020
cDNA1(MLOC_68712) 937
cDNA3(MLOC_68712) 639
CircularRNA -

```
                                2.260               2.280               2.300               2.320
Genomic(MLOC_68712) TAGTTGGTATTTGGCAAGATAAAAAGTGCTCGTTCGGATGAAATTCCATCAAACTATCAGTTTCCTAGAGTCTTTATCCC 2320
  cDNA2(MLOC_68712) TAGTTGGTATTTGGCAAGATAAAAAGTGCTCGTTCGGATGAAATTCCATCAAACTATCAGTTTCCTAGAGTCTTTATCCC 2100
  cDNA1(MLOC_68712) -------------------------------------------------------------------------------- 937
  cDNA3(MLOC_68712) -------------------------------------------------------------------------------- 639
        CircularRNA -------------------------------------------------------------------------------- -

                                2.340               2.360               2.380               2.400
Genomic(MLOC_68712) TAATACCCACAATTTAGCCGCTATGGTAGATAGATAACCTTTCTATCTCGGTTAACACCTCTAAGCCTCGCTGGCCATAT 2400
  cDNA2(MLOC_68712) TAATACCCACAATTTAGCCGCTATGGTAGATAGATAACCTTTCTATCTCGGTTAACACCTCTAAGCCTCGCTGGCCATAT 2180
  cDNA1(MLOC_68712) -------------------------------------------------------------------------------- 937
  cDNA3(MLOC_68712) -------------------------------------------------------------------------------- 639
        CircularRNA -------------------------------------------------------------------------------- -

                                2.420               2.440               2.460               2.480
Genomic(MLOC_68712) CTCTTAGCATCACCTCTAGCTGCCAAGGGCTATAAATTTTAATATCTGATCTGAAGTTGATATACATAAACTAATTAAAG 2480
  cDNA2(MLOC_68712) CTCTTAGCATCACCTCTAGCTGCCAAGGGCTATAAATTTTAATATCTGATCTGAAGTTGATATACATAAACTAATTAAAG 2260
  cDNA1(MLOC_68712) -------------------------------------------------------------------------------- 937
  cDNA3(MLOC_68712) -------------------------------------------------------------------------------- 639
        CircularRNA -------------------------------------------------------------------------------- -

                                2.500               2.520               2.540               2.560
Genomic(MLOC_68712) TTAATTAGCTTATTATTGGTACTTGTGATGTCTTTGAATGCAACTTAGGATTTATATGCTGTGTTAGTGCAGGTGTCTCG 2560
  cDNA2(MLOC_68712) TTAATTAGCTTATTATTGGTACTTGTGATGTCTTTGAATGCAACTTAGGATTTATATGCTGTGTTAGTGCAGGTGTCTCG 2340
  cDNA1(MLOC_68712) -------------------------------------------------------------------------------- 937
  cDNA3(MLOC_68712) -------------------------------------------------------------------------------- 639
        CircularRNA -------------------------------------------------------------------------------- -

                                2.580               2.600               2.620               2.640
Genomic(MLOC_68712) TCTTCTGCTTGTACTCATTATTAGTAGTTGTATCATTTAGTTGCTCTCCATTGTATGGGTATCTCTGTACCTTCCATATT 2640
  cDNA2(MLOC_68712) TCTTCTGCTTGTACTCATTATTAGTAGTTGTATCATTTAGTTGCTCTCCATTGTATGGGTATCTCTGTACCTTCCATATT 2420
  cDNA1(MLOC_68712) -------------------------------------------------------------------------------- 937
  cDNA3(MLOC_68712) -------------------------------------------------------------------------------- 639
        CircularRNA -------------------------------------------------------------------------------- -

                                2.660               2.680               2.700               2.720
Genomic(MLOC_68712) TACAATTGTCACTTCCCTCCGTGAACAGATGACTTCCGTTTGTTTTGTGGTGATCTTGGAAATGAAGTGAATGATGATCT 2720
  cDNA2(MLOC_68712) TACAATTGTCACTTCCCTCCGTGAACAGATGACTTCCGTTTGTTTTGTGGTGATCTTGGAAATGAAGTGAATGATGATCT 2500
  cDNA1(MLOC_68712) ----------------------------ATGACTTCCGTTTGTTTTGTGGTGATCTTGGAAATGAAGTGAATGATGATCT 989
  cDNA3(MLOC_68712) ----------------------------ATGACTTCCGTTTGTTTTGTGGTGATCTTGGAAATGAAGTGAATGATGATCT 691
        CircularRNA -------------------------------------------------------------------------------- -

                                2.740               2.760               2.780               2.800
Genomic(MLOC_68712) TCTGACCAAGGCTTTTTCAAAATATCCATCCTTCAACATGGCTAGGGTGAGTCTTGCCTCTTCTACCCATGACATCAATT 2800
  cDNA2(MLOC_68712) TCTGACCAAGGCTTTTTCAAAATATCCATCCTTCAACATGGCTAGGGT-------------------------------- 2548
  cDNA1(MLOC_68712) TCTGACCAAGGCTTTTTCAAAATATCCATCCTTCAACATGGCTAGGGT-------------------------------- 1037
  cDNA3(MLOC_68712) TCTGACCAAGGCTTTTTCAAAATATCCATCCTTCAACATGGCTAGGGT-------------------------------- 739
        CircularRNA -------------------------------------------------------------------------------- -

                                2.820               2.840               2.860               2.880
Genomic(MLOC_68712) CTATCTCCCTTTCTTTTTAGTGATCTGATTGTAAACACATGATGCATTAGGTTATACGGGATAAATCTAGCGGTAAGACT 2880
  cDNA2(MLOC_68712) ----------------------------------------------------TATACGGGATAAATCTAGCGGTAAGACT 2576
  cDNA1(MLOC_68712) ----------------------------------------------------TATACGGGATAAATCTAGCGGTAAGACT 1065
  cDNA3(MLOC_68712) ----------------------------------------------------TATACGGGATAAATCTAGCGGTAAGACT 767
        CircularRNA -------------------------------------------------------------------------------- -

                                2.900               2.920               2.940               2.960
Genomic(MLOC_68712) AAAGGCTACGGGTTTGCTAGTTTTGCCAATGCATCGGATCTTGCGGCAGCACTGAAAGAGATGAACGGTAAATATGTTGT 2960
  cDNA2(MLOC_68712) AAAGGCTACGGGTTTGCTAGTTTTGCCAATGCATCGGATCTTGCGGCAGCACTGAAAGAGATGAACGGTAAATATGTTG- 2655
  cDNA1(MLOC_68712) AAAGGCTACGGGTTTGCTAGTTTTGCCAATGCATCGGATCTTGCGGCAGCACTGAAAGAGATGAACGGTAAATATGTTG- 1144
  cDNA3(MLOC_68712) AAAGGCTACGGGTTTGCTAGTTTTGCCAATGCATCGGATCTTGCGGCAGCACTGAAAGAGATGAACGGTAAATATGTTG- 846
        CircularRNA -------------------------------------------------------------------------------- -

                                2.980               3.000               3.020               3.040
Genomic(MLOC_68712) ATTAGATGGCCTTTTACTATTTGTACTGCAGTCAAACTTATACTGCTAATATCAACCAGGTAAATATGTTGGAAATCGAC 3040
  cDNA2(MLOC_68712) -----------------------------------------------------------------------GAAATCGAC 2664
  cDNA1(MLOC_68712) -----------------------------------------------------------------------GAAATCGAC 1153
  cDNA3(MLOC_68712) -----------------------------------------------------------------------GAAATCGAC 855
        CircularRNA -------------------------------------------------------------------------------- -

                                3.060               3.080               3.100               3.120
Genomic(MLOC_68712) CAATCAAGCTACGGAAGAGCACATGGAAGAATAGAATAGACTACGAAGCTTTGCAGAAGCCTAAGGTTAGTTCCCATGAC 3120
  cDNA2(MLOC_68712) CAATCAAGCTACGGAAGAGCACATGGAAGAATAGAATAGACTACGAAGCTTTGCAGAAGCCTAAG--------------- 2729
  cDNA1(MLOC_68712) CAATCAAGCTACGGAAGAGCACATGGAAGAATAGAATAGACTACGAAGCTTTGCAGAAGCCTAAG--------------- 1218
  cDNA3(MLOC_68712) CAATCAAGCTACGGAAGAGCACATGGAAGAATAGAATAGACTACGAAGCTTTGCAGAAGCCTAAG--------------- 920
        CircularRNA -------------------------------------------------------------------------------- -

                                3.140               3.160               3.180               3.200
Genomic(MLOC_68712) TGTGGCCTACTCTACTACTCTCCTATGCCGGTGCTCATTTAACTTCCTTTTTTTCTGAGGTAACGGTGCGCATTTAACTG 3200
  cDNA2(MLOC_68712) -------------------------------------------------------------------------------- 2729
  cDNA1(MLOC_68712) -------------------------------------------------------------------------------- 1218
  cDNA3(MLOC_68712) -------------------------------------------------------------------------------- 920
        CircularRNA -------------------------------------------------------------------------------- -

                                3.220               3.240               3.260               3.280
Genomic(MLOC_68712) ATCTGTGCCAAATGCCTGCAGACTCGGCCACAGAAGAAGCTCAAAGTGCAGCCCAGAAGTGTTTTACACAAGTGAAGCAA 3280
  cDNA2(MLOC_68712) ---------------------ACTCGGCCACAGAAGAAGCTCAAAGTGCAGCCCAGAAGTGTTTTACACAAGTGAAGCAA 2788
  cDNA1(MLOC_68712) ---------------------ACTCGGCCACAGAAGAAGCTCAAAGTGCAGCCCAGAAGTGTTTTACACAAGTGAAGCAA 1277
  cDNA3(MLOC_68712) ---------------------ACTCGGCCACAGAAGAAGCTCAAAGTGCAGCCCAGAAGTGTTTTACACAAGTGAAGCAA 979
        CircularRNA ---------------------ACTCGGCCACAGAAGAAGCTCAAAGTGCAGCCCAGAAGTGTTTTACACAAGTGAAGCAA 59

                                3.300               3.320               3.340               3.360
Genomic(MLOC_68712) ATAATTCTAGTTTGTCATCACAGTACCGATAATGATGTACATTTTTTGGCGCATATACAACAGTTGATGTCTTTGGCGCA 3360
  cDNA2(MLOC_68712) ATAATTCTAGTTTGTCATCACAGTACCGATAATGATGTACATTTTTTGGCGCATATACAACAGTTGATGTCTTTGGCGCA 2868
  cDNA1(MLOC_68712) ATAATTCTAGTTTGTCATCACAGTACCGATAATGATGTACATTTTTTGGCGCATATACAACAGTTGATGTCTTTGGCGCA 1357
  cDNA3(MLOC_68712) ATAATTCTAGTTTGTCATCACAGTACCGATAATGATGTACATTTTTTGGCGCATATACAACAGTTGATGTCTTTGGCGCA 1059
        CircularRNA ATAATTCTAGTTTGTCATCACAGTACCGATAATGATGTACATTTTTTGGCGCATATACAACAGTTGATGTCTTTGGCGCA 139
```

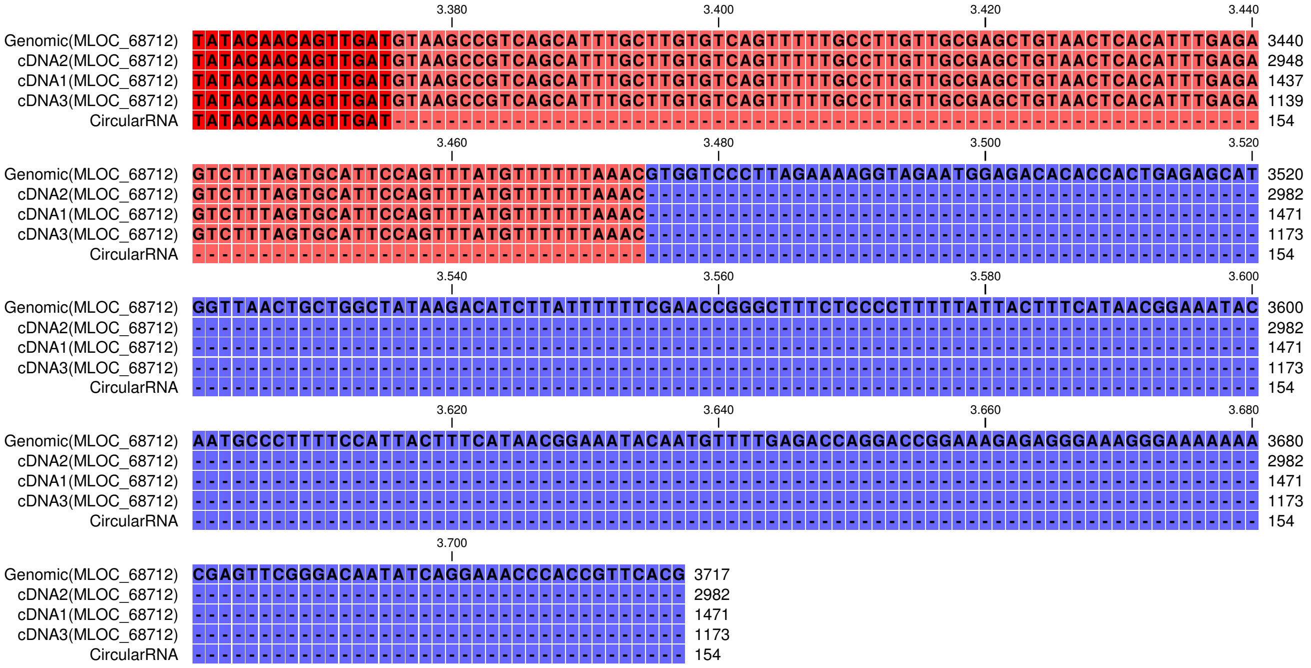
3.380 3.400 3.420 3.440
Genomic(MLOC_68712) TATACAACAGTTGATGTAAGCCGTCAGCATTTGCTTGTGTCAGTTTTTGCCTTGTTGCGAGCTGTAACTCACATTTGAGA 3440
cDNA2(MLOC_68712) TATACAACAGTTGATGTAAGCCGTCAGCATTTGCTTGTGTCAGTTTTTGCCTTGTTGCGAGCTGTAACTCACATTTGAGA 2948
cDNA1(MLOC_68712) TATACAACAGTTGATGTAAGCCGTCAGCATTTGCTTGTGTCAGTTTTTGCCTTGTTGCGAGCTGTAACTCACATTTGAGA 1437
cDNA3(MLOC_68712) TATACAACAGTTGATGTAAGCCGTCAGCATTTGCTTGTGTCAGTTTTTGCCTTGTTGCGAGCTGTAACTCACATTTGAGA 1139
CircularRNA TATACAACAGTTGAT--- 154
3.460 3.480 3.500 3.520
Genomic(MLOC_68712) GTCTTTAGTGCATTCCAGTTTATGTTTTTTAAACGTGGTCCCTTAGAAAAGGTAGAATGGAGACACACCACTGAGAGCAT 3520
cDNA2(MLOC_68712) GTCTTTAGTGCATTCCAGTTTATGTTTTTTAAAC-- 2982
cDNA1(MLOC_68712) GTCTTTAGTGCATTCCAGTTTATGTTTTTTAAAC-- 1471
cDNA3(MLOC_68712) GTCTTTAGTGCATTCCAGTTTATGTTTTTTAAAC-- 1173
CircularRNA -- 154
3.540 3.560 3.580 3.600
Genomic(MLOC_68712) GGTTAACTGCTGGCTATAAGACATCTTATTTTTTCGAACCGGGCTTTCTCCCCTTTTTATTACTTTCATAACGGAAATAC 3600
cDNA2(MLOC_68712) -- 2982
cDNA1(MLOC_68712) -- 1471
cDNA3(MLOC_68712) -- 1173
CircularRNA -- 154
3.620 3.640 3.660 3.680
Genomic(MLOC_68712) AATGCCCTTTTCCATTACTTTCATAACGGAAATACAATGTTTTGAGACCAGGACCGGAAAGAGAGGGAAAGGGAAAAAAA 3680
cDNA2(MLOC_68712) -- 2982
cDNA1(MLOC_68712) -- 1471
cDNA3(MLOC_68712) -- 1173
CircularRNA -- 154
3.700
Genomic(MLOC_68712) CGAGTTCGGGACAATATCAGGAAACCCACCGTTCACG 3717
cDNA2(MLOC_68712) ------------------------------------- 2982
cDNA1(MLOC_68712) ------------------------------------- 1471
cDNA3(MLOC_68712) ------------------------------------- 1173
CircularRNA ------------------------------------- 154

RNA-binding (RRM/RBD/RNP motifs) protein_circular RNA

(ID: Ch6:260427394-260427518)

<u>G</u>AAGTGGAGGAGGTCGACGAGGAGGGCCACCCCGGGAGGAGGGGACGGCGAGATGTGGGC
GATGGTGGTGGCTATGGTGACGCCGG**TGGGGATGACGGGAGGGCTGGGGGCGGTGATTCGT
CGG<u>G</u>**

The nucleotides of junction-region are underlined. The nucleotides of junction-region which are supported by the junction-spaning sequencing reads are shown in red. Introns are not shown if the absence is supported by sequencing reads. In the absence of supporting sequencing reads, the intronic nucletides are shown as N.

Structural relationship between the circular RNA and its parental gene

```
                                                                 20                  40                  60                  80
Genomic(MLOC_7493)  CGACTTTATAAACGCCTCCGGAGCCAGTTCCGCGTTGGACTTGACCTCGCGCCGCCTCCGTTTTTCCTCTCCCCACCTCC  80
 cDNA1(MLOC_7493)   --------------------------------------------------------------------------------  -
 cDNA2(MLOC_7493)   -------ATAAACGCCTCCGGAGCCAGTTCCGCGTTGGACTTGACCTCGCGCCGCCTCCGTTTTTCCTCTCCCCACCTCC  73
      CircularRNA   --------------------------------------------------------------------------------  -

                                                                100                 120                 140                 160
Genomic(MLOC_7493)  TCCCTGTAAACCCGTACCAGAAGCAGCGCTCCTAGGGTTTGCTCCTGCCCCAAACCCTACCACCAGATTGCCCCGATTCC  160
 cDNA1(MLOC_7493)   --------------------------------------------------------------------------------  -
 cDNA2(MLOC_7493)   TCCCTGTAAACCCGTACCAGAAGCAGCGCTCCTAGGGTTTGCTCCTGCCCCAAACCCTACCACCAGATTGCCCCGATTCC  153
      CircularRNA   --------------------------------------------------------------------------------  -

                                                                180                 200                 220                 240
Genomic(MLOC_7493)  GGCAGAGGTAATCCCATCTGCTCCTCGCGCCCTCTCCCCGGCCGCGGGCTAGTAGGTGTATTTCTTTTCCGCTTCGTGTG  240
 cDNA1(MLOC_7493)   --------------------------------------------------------------------------------  -
 cDNA2(MLOC_7493)   GGCAGAGG------------------------------------------------------------------------  161
      CircularRNA   --------------------------------------------------------------------------------  -

                                                                260                 280                 300                 320
Genomic(MLOC_7493)  GGTGCTCAGATTCCGCTGGTTGTTGCAGGCGGCGGCTGGGGGCTGGTGGCATGGCGGGGTATCCGGAGGACAACCAGCAC  320
 cDNA1(MLOC_7493)   --------------------------------------------------------------------------------  -
 cDNA2(MLOC_7493)   -----------------------------CGGCGGCTGGGGGCTGGTGGCATGGCGGGGTATCCGGAGGACAACCAGCAC  212
      CircularRNA   --------------------------------------------------------------------------------  -

                                                                340                 360                 380                 400
Genomic(MLOC_7493)  GCCATGAACGGGTACGAGGATGAGGTCGAGGAAGTGGAGGAGGTCGACGAGGAGGGCCACCCCGGGAGGAGGGGACGGCG  400
 cDNA1(MLOC_7493)   --------------------------------------------------------------------------------  -
 cDNA2(MLOC_7493)   GCCATGAACGGGTACGAGGATGAGGTCGAGGAAGTGGAGGAGGTCGACGAGGAGGGCCACCCCGGGAGGAGGGGACGGCG  292
      CircularRNA   ------------------------------GAAGTGGAGGAGGTCGACGAGGAGGGCCACCCCGGGAGGAGGGGACGGCG  50

                                                                420                 440                 460                 480
Genomic(MLOC_7493)  AGATGTGGGCGATGGTGGTGGCTATGGTGACGCCGGTGGGGATGACGGGAGGGCTGGGGGCGGTGATTCGTCGGGGTAGG  480
 cDNA1(MLOC_7493)   --------------------------------------------------------------------------------  -
 cDNA2(MLOC_7493)   AGATGTGGGCGATGGTGGTGGCTATGGTGACGCCGGTGGGGATGACGGGAGGGCTGGGGGCGGTGATTCGTCGGGG----  368
      CircularRNA   AGATGTGGGCGATGGTGGTGGCTATGGTGACGCCGGTGGGGATGACGGGAGGGCTGGGGGCGGTGATTCGTCGGG-----  125

                                                                500                 520                 540                 560
Genomic(MLOC_7493)  TGATTTGAACTATTTTTTCTGTTTGTCTTGGATTCGCGGAGTGTTGGTGTACTGATGTTTGGAGTCTTTGCAGGAAGATT  560
 cDNA1(MLOC_7493)   --------------------------------------------------------------------------------  -
 cDNA2(MLOC_7493)   --------------------------------------------------------------------------AAGATT  374
      CircularRNA   --------------------------------------------------------------------------------  125

                                                                580                 600                 620                 640
Genomic(MLOC_7493)  TTCGTCGGAGGCGTTGCATGGGAGACAACTGAAGGTATAAGACGGCTCTTGTGTTTTCTTTCTACTTCACTCATAGTTGC  640
 cDNA1(MLOC_7493)   --------------------------------------------------------------------------------  -
 cDNA2(MLOC_7493)   TTCGTCGGAGGCGTTGCATGGGAGACAACTGAAG----------------------------------------------  408
      CircularRNA   --------------------------------------------------------------------------------  125

                                                                660                 680                 700                 720
Genomic(MLOC_7493)  TATTGTCTAGGGATGTTACATTCGTTCAAATGCCAATCTTCAATTGTCCAGCCTTTGTTAGATGCATCATCTTGATAGAA  720
 cDNA1(MLOC_7493)   --------------------------------------------------------------------------------  -
 cDNA2(MLOC_7493)   --------------------------------------------------------------------------------  408
      CircularRNA   --------------------------------------------------------------------------------  125

                                                                740                 760                 780                 800
Genomic(MLOC_7493)  AATCTGGAGAGGAAATTAGCTATAGTAGAAGTATTGTTCTTTGAATACACTGCCACAGATTTTCTAATGGCAGATGTTAA  800
 cDNA1(MLOC_7493)   --------------------------------------------------------------------------------  -
 cDNA2(MLOC_7493)   --------------------------------------------------------------------------------  408
      CircularRNA   --------------------------------------------------------------------------------  125

                                                                820                 840                 860                 880
Genomic(MLOC_7493)  GACATTCAGAAGTGGTCAATACCTGTAGTGCAGTTTGAGGATTTGTGTAGCTATGATAGATTGATCTTATTCATATGTTT  880
 cDNA1(MLOC_7493)   --------------------------------------------------------------------------------  -
 cDNA2(MLOC_7493)   --------------------------------------------------------------------------------  408
      CircularRNA   --------------------------------------------------------------------------------  125

                                                                900                 920                 940                 960
Genomic(MLOC_7493)  TGTGTTCTTTTTTCGGGGTTGCAAGTTTTGGTAATATCGTAGGTAATGCGGATTACTTCTATAATGTGTGCGTGTGCAAT  960
 cDNA1(MLOC_7493)   --------------------------------------------------------------------------------  -
 cDNA2(MLOC_7493)   --------------------------------------------------------------------------------  408
      CircularRNA   --------------------------------------------------------------------------------  125

                                                                980                 1.000               1.020               1.040
Genomic(MLOC_7493)  GTGTACCTAATCAATCTTTTATCAATCAGCTAGCGGGAAAAAGTGATGTCTTGGGTACATGTGTTGTAAAACTTTCATGA  1040
 cDNA1(MLOC_7493)   --------------------------------------------------------------------------------  -
 cDNA2(MLOC_7493)   --------------------------------------------------------------------------------  408
      CircularRNA   --------------------------------------------------------------------------------  125

                                                                1.060               1.080               1.100               1.120
Genomic(MLOC_7493)  GCATATTGGTCCAGTTGTTTGTACTTTGAAACAGTTCTGCTTCTTAACATCTCCTGAAGGATAGCTGTTTATACTTTGTT  1120
 cDNA1(MLOC_7493)   --------------------------------------------------------------------------------  -
 cDNA2(MLOC_7493)   --------------------------------------------------------------------------------  408
      CircularRNA   --------------------------------------------------------------------------------  125

                                                                1.140               1.160               1.180               1.200
Genomic(MLOC_7493)  ATTCACTGCTGCTTTCAGGTTCAGTATAGTTTGGTTATTGCATTAGTTTGTAGTCTCATCCAAACAGAAGGTTCCATTAT  1200
 cDNA1(MLOC_7493)   --------------------------------------------------------------------------------  -
 cDNA2(MLOC_7493)   --------------------------------------------------------------------------------  408
      CircularRNA   --------------------------------------------------------------------------------  125

                                                                1.220               1.240               1.260               1.280
Genomic(MLOC_7493)  TTGTCTTCCATTGTCAAATATACCAGCTCGGTCACTATTGCTTGAGGGGTTAGTCCTAGTTTTTGCAAACTACTCCCTCC  1280
 cDNA1(MLOC_7493)   --------------------------------------------------------------------------------  -
 cDNA2(MLOC_7493)   --------------------------------------------------------------------------------  408
      CircularRNA   --------------------------------------------------------------------------------  125
```

```
                                                   1.300               1.320               1.340               1.360
Genomic(MLOC_7493)  GTTCCTAAATATAAGTCGTTCTAGAGATTCCACTACGGACTACATACGGAGCAAAATGTGTGAATCTAGACTCTAAATTA 1360
cDNA1(MLOC_7493)    -------------------------------------------------------------------------------- -
cDNA2(MLOC_7493)    -------------------------------------------------------------------------------- 408
CircularRNA         -------------------------------------------------------------------------------- 125

                                                   1.380               1.400               1.420               1.440
Genomic(MLOC_7493)  TGTCTATATACATCACTATGTAGTCCATAGTGAAATCTCTAAAAAGACTTATGTTTTGGAATGGAGGGAGTAATACTGTA 1440
cDNA1(MLOC_7493)    -------------------------------------------------------------------------------- -
cDNA2(MLOC_7493)    -------------------------------------------------------------------------------- 408
CircularRNA         -------------------------------------------------------------------------------- 125

                                                   1.460               1.480               1.500               1.520
Genomic(MLOC_7493)  CCACTTACAAATCATGTCTATCTTATTTTTAATTTCGGTAAATATAGGAATGAAGTTATGTTATACAGAAGATAATTTTT 1520
cDNA1(MLOC_7493)    -------------------------------------------------------------------------------- -
cDNA2(MLOC_7493)    -------------------------------------------------------------------------------- 408
CircularRNA         -------------------------------------------------------------------------------- 125

                                                   1.540               1.560               1.580               1.600
Genomic(MLOC_7493)  TTTGCGGTCAGTGCATTTAACCGTATCTATGTGTATCTTGCATTTAGTTTATCTTGTTATCGATAGCATACATTTATTTT 1600
cDNA1(MLOC_7493)    -------------------------------------------------------------------------------- -
cDNA2(MLOC_7493)    -------------------------------------------------------------------------------- 408
CircularRNA         -------------------------------------------------------------------------------- 125

                                                   1.620               1.640               1.660               1.680
Genomic(MLOC_7493)  GATAAAAGATACTCGGGCAAGAACAACTCTGTTTCATCTTTGCATCTCCTTTATTTCTTCTTTATTAGTTATGTTAGAAT 1680
cDNA1(MLOC_7493)    GATAAAAGATACTCGGGCAAGAACAACTCTGTTTCATCTTTGCATCTCCTTTATTTCTTCTTTATTAGTTATGTTAGAAT 80
cDNA2(MLOC_7493)    -------------------------------------------------------------------------------- 408
CircularRNA         -------------------------------------------------------------------------------- 125

                                                   1.700               1.720               1.740               1.760
Genomic(MLOC_7493)  GTTTCACTCTTGTATGTTGCACATCGGTCTCCTTTGAATTCAGTAATATTCATTTGTTTCATGCATTAATATACTTGTAG 1760
cDNA1(MLOC_7493)    GTTTCACTCTTGTATGTTGCACATCGGTCTCCTTTGAATTCAGTAATATTCATTTGTTTCATGCATTAATATACTTGTAG 160
cDNA2(MLOC_7493)    -------------------------------------------------------------------------------- 408
CircularRNA         -------------------------------------------------------------------------------- 125

                                                   1.780               1.800               1.820               1.840
Genomic(MLOC_7493)  TTCTTAAAGGAAAGTAGGCATGAGAATGAGTTGACTTTCTTTTAGTAATGTTTGTGTATCTGTTGGTGTTCTTGGTTTCT 1840
cDNA1(MLOC_7493)    TTCTTAAAGGAAAGTAGGCATGAGAATGAGTTGACTTTCTTTTAGTAATGTTTGTGTATCTGTTGGTGTTCTTGGTTTCT 240
cDNA2(MLOC_7493)    -------------------------------------------------------------------------------- 408
CircularRNA         -------------------------------------------------------------------------------- 125

                                                   1.860               1.880               1.900               1.920
Genomic(MLOC_7493)  GAGTATGTTAGCTGTTGTTGCTGCTTTTTATATGAAATGTTTCTATCGTGTAAAAAACACTCAGCTATTTCTTCAATATT 1920
cDNA1(MLOC_7493)    GAGTATGTTAGCTGTTGTTGCTGCTTTTTATATGAAATGTTTCTATCGTGTAAAAAACACTCAGCTATTTCTTCAATATT 320
cDNA2(MLOC_7493)    -------------------------------------------------------------------------------- 408
CircularRNA         -------------------------------------------------------------------------------- 125

                                                   1.940               1.960               1.980               2.000
Genomic(MLOC_7493)  CCCTGGTTTCTTTTTAGCAATCCAGTTAATTTTCTTTCGCCAATTGACAATTGTGTATGGTTTATTTGTTTGTATGTAGA 2000
cDNA1(MLOC_7493)    CCCTGGTTTCTTTTTAGCAATCCAGTTAATTTTCTTTCGCCAATTGACAATTGTGTATGGTTTATTTGTTTGTATGTAGA 400
cDNA2(MLOC_7493)    -------------------------------------------------------------------------------A 409
CircularRNA         -------------------------------------------------------------------------------- 125

                                                   2.020               2.040               2.060               2.080
Genomic(MLOC_7493)  ATCATTCTCCAAGCATTTTGGGAAGTATGGGGCTATAACTGATTCTGTAATTATGAAGGACAAGCATACTAAGATGCCTC 2080
cDNA1(MLOC_7493)    ATCATTCTCCAAGCATTTTGGGAAGTATGGGGCTATAACTGATTCTGTAATTATGAAGGACAAGCATACTAAGATGCCTC 480
cDNA2(MLOC_7493)    ATCATTCTCCAAGCATTTTGGGAAGTATGGGGCTATAACTGATTCTGTAATTATGAAGGACAAGCATACTAAGATGCCTC 489
CircularRNA         -------------------------------------------------------------------------------- 125

                                                   2.100               2.120               2.140               2.160
Genomic(MLOC_7493)  GTGGATTTGGATTTGTTACATTTTCTGATCCATCTGTAATAGACAAGGTTTTGGAGGATGAACACAATATAGATGGAAGA 2160
cDNA1(MLOC_7493)    GTGGATTTGGATTTGTTACATTTTCTGATCCATCTGTAATAGACAAGGTTTTGGAGGATGAACACAATATAGATGGAAGA 560
cDNA2(MLOC_7493)    GTGGATTTGGATTTGTTACATTTTCTGATCCATCTGTAATAGACAAGGTTTTGGAGGATGAACACAATATAGATGGAAGA 569
CircularRNA         -------------------------------------------------------------------------------- 125

                                                   2.180               2.200               2.220               2.240
Genomic(MLOC_7493)  ACGGTAGGATATCTTAATCTAGATTGGCCGTTATGTGGGACCTCATTGTAAAGTTTATTCTTAGTTAATAGTTGCACAGT 2240
cDNA1(MLOC_7493)    ACGGTAGGATATCTTAATCTAGATTGGCCGTTATGTGGGACCTCATTGTAAAGTTTATTCTTAGTTAATAGTTGCACAGT 640
cDNA2(MLOC_7493)    ACGGT--------------------------------------------------------------------------- 574
CircularRNA         -------------------------------------------------------------------------------- 125

                                                   2.260               2.280               2.300               2.320
Genomic(MLOC_7493)  TGATTTGAAGCGTTCAGCTTCTCTGTATCTCTCTCAACTTATGAACTATCCAGGTTGAAGTCAAAAGGACAGTCCCGAGG 2320
cDNA1(MLOC_7493)    TGATTTGAAGCGTTCAGCTTCTCTGTATCTCTCTCAACTTATGAACTATCCAGGTTGAAGTCAAAAGGACAGTCCCGAGG 720
cDNA2(MLOC_7493)    -------------------------------------------------------TGAAGTCAAAAGGACAGTCCCGAGG 599
CircularRNA         -------------------------------------------------------------------------------- 125

                                                   2.340               2.360               2.380               2.400
Genomic(MLOC_7493)  GAAGAAATGTCCTCAAAAGATGGCCCCAAGACAAGAAAGATCTTTGTTGGTGGGCTACCATCAACACTAACTGAAGGTAT 2400
cDNA1(MLOC_7493)    GAAGAAATGTCCTCAAAAGATGGCCCCAAGACAAGAAAGATCTTTGTTGGTGGGCTACCATCAACACTAACTGAAGGTAT 800
cDNA2(MLOC_7493)    GAAGAAATGTCCTCAAAAGATGGCCCCAAGACAAGAAAGATCTTTGTTGGTGGGCTACCATCAACACTAACTGAAG---- 675
CircularRNA         -------------------------------------------------------------------------------- 125

                                                   2.420               2.440               2.460               2.480
Genomic(MLOC_7493)  TAGTTGGTATTAGCTGCAAATGACCAACCTTGTACCTATTTTGGTTATGGTAAAACTTACAAGCTTTTAATTTCAGATGA 2480
cDNA1(MLOC_7493)    TAGTTGGTATTAGCTGCAAATGACCAACCTTGTACCTATTTTGGTTATGGTAAAACTTACAAGCTTTTAATTTCAGATGA 880
cDNA2(MLOC_7493)    ----------------------------------------------------------------------------ATGA 679
CircularRNA         -------------------------------------------------------------------------------- 125

                                                   2.500               2.520               2.540               2.560
Genomic(MLOC_7493)  TTTGAGGGATCACTTTTCCTCATATGGCAACGTGGTTGAACATCAGATAATGGTGGATCACAGCACTGGCCGTTCGAGAG 2560
cDNA1(MLOC_7493)    TTTGAGGGATCACTTTTCCTCATATGGCAACGTGGTTGAACATCAGATAATGGTGGATCACAGCACTGGCCGTTCGAGAG 960
cDNA2(MLOC_7493)    TTTGAGGGATCACTTTTCCTCATATGGCAACGTGGTTGAACATCAGATAATGGTGGATCACAGCACTGGCCGTTCGAGAG 759
CircularRNA         -------------------------------------------------------------------------------- 125
```

```
                                        2.580               2.600               2.620               2.640
Genomic(MLOC_7493) GTTTTGGTTTTGTCACTTTTGAAAGTGAGGATTCTGTCGAAAGGGTCATATCCGAGGGAAGAATGCGTGATCTTGGTGGG 2640
  cDNA1(MLOC_7493) GTTTTGGTTTTGTCACTTTTGAAAGTGAGGATTCTGTCGAAAGGGTCATATCCGAGGGAAGAATGCGTGATCTTGGTGGG 1040
  cDNA2(MLOC_7493) GTTTTGGTTTTGTCACTTTTGAAAGTGAGGATTCTGTCGAAAGGGTCATATCCGAGGGAAGAATGCGTGATCTTGGTGGG 839
       CircularRNA -------------------------------------------------------------------------------- 125

                                        2.660               2.680               2.700               2.720
Genomic(MLOC_7493) AAGCAGGTCTGTATCTTCTCCTGTTTCCAAACCTTTTTATTTAAGTAGTTCTTCCTGAGTTCCATTATCTTATAGGAAAT 2720
  cDNA1(MLOC_7493) AAGCAGGTCTGTATCTTCTCCTGTTTCCAAACCTTTTTATTTAAGTAGTTCTTCCTGAGTTCCATTATCTTATAGGAAAT 1120
  cDNA2(MLOC_7493) AAGCAGGT------------------------------------------------------------------------ 847
       CircularRNA -------------------------------------------------------------------------------- 125

                                        2.740               2.760               2.780               2.800
Genomic(MLOC_7493) ATATAAGGTAAGGATAGTAGAGATAAACGTTCATGAGGATTTCTGCACTTAACTGCTCAGTTATGAGATTTGCTCCAAAG 2800
  cDNA1(MLOC_7493) ATATAAGGTAAGGATAGTAGAGATAAACGTTCATGAGGATTTCTGCACTTAACTGCTCAGTTATGAGATTTGCTCCAAAG 1200
  cDNA2(MLOC_7493) -------------------------------------------------------------------------------- 847
       CircularRNA -------------------------------------------------------------------------------- 125

                                        2.820               2.840               2.860               2.880
Genomic(MLOC_7493) TGGTGAATGGAATCCCTTCTAAAAGCTTCTTATTGTGGTTTTATCTTCTTGTGGTTATTGCTGCTACTATATTGGACATG 2880
  cDNA1(MLOC_7493) TGGTGAATGGAATCCCTTCTAAAAGCTTCTTATTGTGGTTTTATCTTCTTGTGGTTATTGCTGCTACTATATTGGACATG 1280
  cDNA2(MLOC_7493) -------------------------------------------------------------------------------- 847
       CircularRNA -------------------------------------------------------------------------------- 125

                                        2.900               2.920               2.940               2.960
Genomic(MLOC_7493) TCCTTCTAATGTTTAGAGTTCTTTATGCATTGCTGGTATTTTCTTGCTGAGCCAGATGGATTGGAAACTTTTATCTCAGA 2960
  cDNA1(MLOC_7493) TCCTTCTAATGTTTAGAGTTCTTTATGCATTGCTGGTATTTTCTTGCTGAGCCAGATGGATTGGAAACTTTTATCTCAGA 1360
  cDNA2(MLOC_7493) -------------------------------------------------------------------------------- 847
       CircularRNA -------------------------------------------------------------------------------- 125

                                        2.980               3.000               3.020               3.040
Genomic(MLOC_7493) TGCACCACTTACATATAGAAGATTATTTGTTTTACATCAAATTGAGCTGCGAGCTTGATATTGCTTTTAGTTGACAAATC 3040
  cDNA1(MLOC_7493) TGCACCACTTACATATAGAAGATTATTTGTTTTACATCAAATTGAGCTGCGAGCTTGATATTGCTTTTAGTTGACAAATC 1440
  cDNA2(MLOC_7493) -------------------------------------------------------------------------------- 847
       CircularRNA -------------------------------------------------------------------------------- 125

                                        3.060               3.080               3.100               3.120
Genomic(MLOC_7493) ATAGTAAATTTCTTCACAGGTTGAAATAAAGAAGGCTGAACCAAAGAAACATGGTGGTGATCACAGTAGCAATGGGAGAT 3120
  cDNA1(MLOC_7493) ATAGTAAATTTCTTCACAGGTTGAAATAAAGAAGGCTGAACCAAAGAAACATGGTGGTGATCACAGTAGCAATGGGAGAT 1520
  cDNA2(MLOC_7493) ---------------------TGAAATAAAGAAGGCTGAACCAAAGAAACATGGTGGTGATCACAGTAGCAATGGGAGAT 906
       CircularRNA -------------------------------------------------------------------------------- 125

                                        3.140               3.160               3.180               3.200
Genomic(MLOC_7493) CTAGTCACTCTGGTGGTGGTGGTTACCGTAGTTCTTACCGTAGTGGTGGAGCTGCTGCTGGTGGTGGCAGTAGCAGCGCT 3200
  cDNA1(MLOC_7493) CTAGTCACTCTGGTGGTGGTGGTTACCGTAGTTCTTACCGTAGTGGTGGAGCTGCTGCTGGTGGTGGCAGTAGCAGCGCT 1600
  cDNA2(MLOC_7493) CTAGTCACTCTGGTGGTGGTGGTTACCGTAGTTCTTACCGTAGTGGTGGAGCTGCTGCTGGTGGTGGCAGTAGCAGCGCT 986
       CircularRNA -------------------------------------------------------------------------------- 125

                                        3.220               3.240               3.260               3.280
Genomic(MLOC_7493) GGTGGCGGCGGCTATGGCTATGGTGCTGGTGGTCATCGGTCTGCTGCTGCAGGAAGTTATTATGATAGCACGGGATATGG 3280
  cDNA1(MLOC_7493) GGTGGCGGCGGCTATGGCTATGGTGCTGGTGGTCATCGGTCTGCTGCTGCAGGAAGTTATTATGATAGCACGGGATATGG 1680
  cDNA2(MLOC_7493) GGTGGCGGCGGCTATGGCTATGGTGCTGGTGGTCATCGGTCTGCTGCTGCAGGAAGTTATTATGATAGCACGGGATATGG 1066
       CircularRNA -------------------------------------------------------------------------------- 125

                                        3.300               3.320               3.340               3.360
Genomic(MLOC_7493) TTATGGTAGAGGAGGCTATGGTGCCGCCGCTGCTGCCGCTGCCTATGGAGGCAATGCTGGATATGGGTCTGGTTTTGGTG 3360
  cDNA1(MLOC_7493) TTATGGTAGAGGAGGCTATGGTGCCGCCGCTGCTGCCGCTGCCTATGGAGGCAATGCTGGATATGGGTCTGGTTTTGGTG 1760
  cDNA2(MLOC_7493) TTATGGTAGAGGAGGCTATGGTGCCGCCGCTGCTGCCGCTGCCTATGGAGGCAATGCTGGATATGGGTCTGGTTTTGGTG 1146
       CircularRNA -------------------------------------------------------------------------------- 125

                                        3.380               3.400               3.420               3.440
Genomic(MLOC_7493) GTGGCTATGGAGGCTCTATGTACGGTGGTGCTTATGGTGCATACGGACCATATGGTGGTGGTGCCTATGGTGCGGGTGCT 3440
  cDNA1(MLOC_7493) GTGGCTATGGAGGCTCTATGTACGGTGGTGCTTATGGTGCATACGGACCATATGGTGGTGGTGCCTATGGTGCGGGTGCT 1840
  cDNA2(MLOC_7493) GTGGCTATGGAGGCTCTATGTACGGTGGTGCTTATGGTGCATACGGACCATATGGTGGTGGTGCCTATGGTGCGGGTGCT 1226
       CircularRNA -------------------------------------------------------------------------------- 125

                                        3.460               3.480               3.500               3.520
Genomic(MLOC_7493) TATGGAGGCGGTGCCTATGGTGGGGGTGCTTATGGAGGTGGTGCCTATGGCGGCGGCGCTCCTGGTGGCTACGGTGCTGG 3520
  cDNA1(MLOC_7493) TATGGAGGCGGTGCCTATGGTGGGGGTGCTTATGGAGGTGGTGCCTATGGCGGCGGCGCTCCTGGTGGCTACGGTGCTGG 1920
  cDNA2(MLOC_7493) TATGGAGGCGGTGCCTATGGTGGGGGTGCTTATGGAGGTGGTGCCTATGGCGGCGGCGCTCCTGGTGGCTACGGTGCTGG 1306
       CircularRNA -------------------------------------------------------------------------------- 125

                                        3.540               3.560               3.580               3.600
Genomic(MLOC_7493) TGGATATGGTAGTTATGGGGGAGCAGGAGCAGGGGCAGGAGCAGGGGGTGCTGGTGGTGGTGGTGGTGGAAGTGCAGGTG 3600
  cDNA1(MLOC_7493) TGGATATGGTAGTTATGGGGGAGCAGGAGCAGGGGCAGGAGCAGGGGGTGCTGGTGGTGGTGGTGGTGGAAGTGCAGGTG 2000
  cDNA2(MLOC_7493) TGGATATGGTAGTTATGGGGGAGCAGGAGCAGGGGCAGGAGCAGGGGGTGCTGGTGGTGGTGGTGGTGGAAGTGCAGGTG 1386
       CircularRNA -------------------------------------------------------------------------------- 125

                                        3.620               3.640               3.660               3.680
Genomic(MLOC_7493) GTCGGGGCTCTAGCAGGTACCATCCCTATGGGAAATGAGTGCAAACCCGTGTGGTTTTTTCTAGGATGCAATAAGCGCAC 3680
  cDNA1(MLOC_7493) GTCGGGGCTCTAGCAGGTACCATCCCTATGGGAAATGAGTGCAAACCCGTGTGGTTTTTTCTAGGATGCAATAAGCGCAC 2080
  cDNA2(MLOC_7493) GTCGGGGCTCTAGCAGGTACCATCCCTATGGGAAATGAGTGCAAACCCGTGTGGTTTTTTCTAGGATGCAATAAGCGCAC 1466
       CircularRNA -------------------------------------------------------------------------------- 125

                                        3.700               3.720               3.740               3.760
Genomic(MLOC_7493) TCATCTCCATGGGATCTCCTAGAAGTTGGGCATTCATCTTGGGTTACCTAAACAGAGGCATCAGAACATTTTTGGTTTTG 3760
  cDNA1(MLOC_7493) TCATCTCCATGGGATCTCCTAGAAGTTGGGCATTCATCTTGGGTTACCTAAACAGAGGCATCAGAACATTTTTGGTTTTG 2160
  cDNA2(MLOC_7493) TCATCTCCATGGGATCTCCTAGAAGTTGGGCATTCATCTTGGGTTACCTAAACAGAGGCATCAGAACATTTTTGGTTTTG 1546
       CircularRNA -------------------------------------------------------------------------------- 125

                                        3.780               3.800               3.820               3.840
Genomic(MLOC_7493) TTGAATTAAAGTCGTAACCAGAGACAAGTGTAATCTTTGTTGCTGAGCCTGCCCGTAGTTTGGTGTAGCGGCTACACATT 3840
  cDNA1(MLOC_7493) TTGAATTAAAGTCGTAACCAGAGACAAGTGTAATCTTTGTTGCTGAGCCTGCCCGTAGTTTGGTGTAGCGGCTACACATT 2240
  cDNA2(MLOC_7493) TTGAATTAAAGTCGTAACCAGAGACAAGTGTAATCTTTGTTGCTGAGCCTGCCCGTAGTTTGGTGTAGCGGCTACACATT 1626
       CircularRNA -------------------------------------------------------------------------------- 125
```

```
                                  3.860               3.880               3.900               3.920
Genomic(MLOC_7493) AAACTGTGTTGTATTCCTTAGTTTCCATACGCAGAGCGTTAGAAAACCCTGAAAACCAAGCCTTTCGTGGTAGGAACAAT 3920
  cDNA1(MLOC_7493) AAACTGTGTTGTATTCCTTAGTTTCCATACGCAGAGCGTTAGAAAACCCTGAAAACCAAGCCTTTCGTGGTAGGAACAAT 2320
  cDNA2(MLOC_7493) AAACTGTGTTGTATTCCTTAGTTTCCATACGCAGAGCGTTAGAAAACCCTGAAAACCAAGCCTTTCGTGGTAGGAACAAT 1706
       CircularRNA -------------------------------------------------------------------------------- 125
                                  3.940               3.960               3.980               4.000
Genomic(MLOC_7493) ATTGGTGTGATAGAACTATATTTGTGCTGTAAGAGATTGTCTTTGCAAGCTAGCTGTTGCCATCTGAGATTATATGTAGG 4000
  cDNA1(MLOC_7493) ATTGGTGTGATAGAACTATATTTGTGCTGTAAGAGATTGTCTTTGCAAGCTAGCTGTTGCCATCTGAGATTATATGTAGG 2400
  cDNA2(MLOC_7493) ATTGGTGTGATAGAACTATATTTGTGCTGTAAGAGATTGTCTTTGCAAGCTAGCTGTTGCCATCTGAGATTATATGTAGG 1786
       CircularRNA -------------------------------------------------------------------------------- 125
                                  4.020               4.040               4.060               4.080
Genomic(MLOC_7493) GGAGCCTTTAAACTGTTTGGTTTATGCATCTGGCGGACTGTTTGATGCATTTTGACATCGTAGGTTATGTATAGTCATTG 4080
  cDNA1(MLOC_7493) GGAGCCTTTAAACTGTTTGGTTTATGCATCTGGCGGACTGTTTGATGCATTTTGACATCGTAGGTTATGTATAGTCATTG 2480
  cDNA2(MLOC_7493) GGAGCCTTTAAACTGTTTGGTTTATGC----------------------------------------------------- 1813
       CircularRNA -------------------------------------------------------------------------------- 125
                                  4.100               4.120               4.140               4.160
Genomic(MLOC_7493) CTAATGCTCATGGAACCCAGATGCGTTCTGGGTGTAAAAGAACATGATTTTATGACTTGTGGCACTGAAGTTTTTTGCTA 4160
  cDNA1(MLOC_7493) CTAATGCTCATGGAACCCAGATGCGTTCTGGGTGTAAAAGAACATGATTTTATGACTTGTGGCACTGAAGTTTTTTGCTA 2560
  cDNA2(MLOC_7493) -------------------------------------------------------------------------------- 1813
       CircularRNA -------------------------------------------------------------------------------- 125
                                  4.180               4.200               4.220               4.240
Genomic(MLOC_7493) CCAGTGTTTGCAAATTTAATCATCATTGCTATATATCGTCTGGTAACATTTGTCCCTGTTCCGTCGACATGTTTTCACTC 4240
  cDNA1(MLOC_7493) CCAGTGTTTGCAAATTTAATCATCATTGCTATATATCGTCTGGTAACATTTGTCCCTGTTCCGTCGACATGTTTTCACTC 2640
  cDNA2(MLOC_7493) -------------------------------------------------------------------------------- 1813
       CircularRNA -------------------------------------------------------------------------------- 125
                                  4.260               4.280               4.300               4.320
Genomic(MLOC_7493) TGGAAACGTCTGACGAGTTAAAAGCCCTAAATGAAAGAGCATTCCCAATGGCCGACCCAAACGTCTGGGGACCTGTCCTC 4320
  cDNA1(MLOC_7493) TGGAAACGTCTGACGAGTTAAAAGCCCTAAATGAAAGAGCATTCCCAATGGCCGACCCAAACGTCTGGGGACCTGTCCTC 2720
  cDNA2(MLOC_7493) -------------------------------------------------------------------------------- 1813
       CircularRNA -------------------------------------------------------------------------------- 125
                                  4.340               4.360               4.380               4.400
Genomic(MLOC_7493) GAACGGCGGCATAGGGGTAGGCCGTTCAACCTTTTTATAGTTTTTAAATAACTAGCAAAAAGGCACGCGTTGCAATGGGA 4400
  cDNA1(MLOC_7493) GAACGGCGGCATAGGGGTAGGCCGTTCAACCTTTTTATAGTTTTTAAATAACTAGCAAAAAGGCACGCGTTGCAATGGGA 2800
  cDNA2(MLOC_7493) -------------------------------------------------------------------------------- 1813
       CircularRNA -------------------------------------------------------------------------------- 125
                                  4.420               4.440               4.460               4.480
Genomic(MLOC_7493) GAAAAAAAATCATAATCTTTAATTGTGGTAATCACATTATGTTCTTAAAATTTTAGGACATTTCTTGAGATGCATAAACA 4480
  cDNA1(MLOC_7493) GAAAAAAAATCATAATCTTTAATTGTGGTAATCACATTATGTTCTTAAAATTTTAGGACATTTCTTGAGATGCATAAACA 2880
  cDNA2(MLOC_7493) -------------------------------------------------------------------------------- 1813
       CircularRNA -------------------------------------------------------------------------------- 125
                                  4.500               4.520               4.540               4.560
Genomic(MLOC_7493) TTATTTGAATTGTGATGAAAATGACAAAGTATGTTTTGTTAATCAATGATAGCATATGGATAAGAACATTATTTTTATCT 4560
  cDNA1(MLOC_7493) TTATTTGAATTGTGATGAAAATGACAAAGTATGTTTTGTTAATCAATGATAGCATATGGATAAGAACATTATTTTTATCT 2960
  cDNA2(MLOC_7493) -------------------------------------------------------------------------------- 1813
       CircularRNA -------------------------------------------------------------------------------- 125
                                  4.580               4.600               4.620               4.640
Genomic(MLOC_7493) TGCTGATATATGTGTTTGTATTTGTAATCCAAGTGAAGTCTTAATTTGGTTGCAAACATATTAAACAACTTTATCGAAAA 4640
  cDNA1(MLOC_7493) TGCTGATATATGTGTTTGTATTTGTAATCCAAGTGAAGTCTTAATTTGGTTGCAAACATATTAAACAACTTTATCGAAAA 3040
  cDNA2(MLOC_7493) -------------------------------------------------------------------------------- 1813
       CircularRNA -------------------------------------------------------------------------------- 125
                                  4.660               4.680               4.700               4.720
Genomic(MLOC_7493) CAATCAATTTGTAAGTACATGTCTACCAAATGTTACATTTCATATATATGAAAGATCGAGCTAAGCCATTTGTTTACATA 4720
  cDNA1(MLOC_7493) CAATCAATTTGTAAGTACATGTCTACCAAATGTTACATTTCATATATATGAAAGATCGAGCTAAGCCATTTGTTTACATA 3120
  cDNA2(MLOC_7493) -------------------------------------------------------------------------------- 1813
       CircularRNA -------------------------------------------------------------------------------- 125
                                  4.740               4.760               4.780
Genomic(MLOC_7493) AAGCTTGTATAAATATCATTGTATATTTTTTATACACTAGTACAAATGACCGTGCGTTACATCGGGC 4787
  cDNA1(MLOC_7493) AAGCTTGTATAAATATCATTGTATATTTTTTATACACTAGTACAAATGACCGTGCGTTACATCGGGC 3187
  cDNA2(MLOC_7493) ------------------------------------------------------------------- 1813
       CircularRNA ------------------------------------------------------------------- 125
```

Ribosomal protein L30/L7_circular RNA (ID: Ch5:524054971-524055497)

CTGAAGAGCGTGAGGGAGTTGATCTACAAGAGGGGTTATGGAAAGCTCAACAAGCAAAGGAT
TCCTCTGACTAACAACAAGGTCATCGAGGAGGGTCTTGGAAAGCACAACATCATCTGCATTGAA
GACGTTGTCCATGAGATCTTGACGGTTGGCCCTCACTTCAAGGAGGCCAACAACTTCCTGTGGC
CATTCAAGCTCAAGGCGCCGCTGGGAGGCCTCAAGAAGAAGAGGAACCACTACGTCGAGGGTG
GTGACGCCGGCAACCGTGAGGACTACATCAACCAGCTCGTCAGGAGGATGAACTAGGTTGATT
GATCGATGGCGCCTTGTGTTTGGAAGATGTTTTGTTTGTTAAGCCCGCTTTACATTAGGAAGTGG
ATGGTGGATGCAGTTTTGATA**TTTCGGAACCATGATGCATGTTTGGAAGAAT**

The nucleotides of junction-region are underlined. The nucleotides of junction-region which are supported by the junction-spaning sequencing reads are shown in red. Introns are not shown if the absence is supported by sequencing reads. In the absence of supporting sequencing reads, the intronic nucletides are shown as N.

Structural relationship between the circular RNA and its parental gene

```
                      20                  40                  60                  80
Genomic(MLOC_17150)   CTCGCCTCCTCCTCCCTCTGCTGGAAGCTCGTCGAGGTAAGCGCACGCCCTCCCGATCCAACCAGCCGCGGCGAACGCAT 80
cDNA(MLOC_17150)      CTCGCCTCCTCCTCCCTCTGCTGGAAGCTCGTCGAG-------------------------------------------- 36
CircularRNA           -------------------------------------------------------------------------------- -

                      100                 120                 140                 160
Genomic(MLOC_17150)   TTGCGCCCGCAGCTCGCACGGATCTGACCGTGCAGATGTCGCAGATGTCGTCCGAGGCGGCGAAGGTGGTGGTGCCGGAG 160
cDNA(MLOC_17150)      --------------------------------------------ATGTCGTCCGAGGCGGCGAAGGTGGTGGTGCCGGAG 72
CircularRNA           -------------------------------------------------------------------------------- -

                      180                 200                 220                 240
Genomic(MLOC_17150)   TCGGTGCTCCTCAAGAGGAAGCGGGAGGAGCTCTGGGCCGCCGAGAAGAAGACCAAGGCCGTCGAGGAGAAGAAGAAGTC 240
cDNA(MLOC_17150)      TCGGTGCTCCTCAAGAGGAAGCGGGAGGAGCTCTGGGCCGCCGAGAAGAAGACCAAGGCCGTCGAGGAGAAGAAGAAGTC 152
CircularRNA           -------------------------------------------------------------------------------- -

                      260                 280                 300                 320
Genomic(MLOC_17150)   GACGGAGAACCGCAAGGTCATCTTCGCCCGCGCCAAGCAGTACGCCGAGGAGTACGATGCACAGGTCAGATCGCCGCTCC 320
cDNA(MLOC_17150)      GACGGAGAACCGCAAGGTCATCTTCGCCCGCGCCAAGCAGTACGCCGAGGAGTACGATGCACAGG--------------- 217
CircularRNA           -------------------------------------------------------------------------------- -

                      340                 360                 380                 400
Genomic(MLOC_17150)   CCGCTCCCCGCTCCATCCCTTGCCCCTTTAGCGGTCGTTTGCTCATGCGTTCGTGGTTAGATTTTAGCGCGAGAAGATGC 400
cDNA(MLOC_17150)      -------------------------------------------------------------------------------- 217
CircularRNA           -------------------------------------------------------------------------------- -

                      420                 440                 460                 480
Genomic(MLOC_17150)   GTTGTGCCGGATCTGTCATGGATTCGTTTGTTTGTGGTGGATTTCGGATTGTGCTCGACGTGTTTTCTGCTAATACTTGA 480
cDNA(MLOC_17150)      -------------------------------------------------------------------------------- 217
CircularRNA           -------------------------------------------------------------------------------- -

                      500                 520                 540                 560
Genomic(MLOC_17150)   TCGTCATACACTTGCATAGATGTAAACATTCGCTCTCAGTGATGCCGCAGTAACCTAGAAATGCCATTCAGTTCGTAGTT 560
cDNA(MLOC_17150)      -------------------------------------------------------------------------------- 217
CircularRNA           -------------------------------------------------------------------------------- -

                      580                 600                 620                 640
Genomic(MLOC_17150)   CGTGGCAATTCTGTTGTGTGTTGATGATGTATGTGCATGTCCCTTGCCATTGTCTGTATATATAATGACCTAGGCCAGAT 640
cDNA(MLOC_17150)      -------------------------------------------------------------------------------- 217
CircularRNA           -------------------------------------------------------------------------------- -

                      660                 680                 700                 720
Genomic(MLOC_17150)   TTGAATGGTTTTGCAGCAAAATGAAATCAAACCTGAACCATTTAAAGGTGATTTAGGCGTTGTATTACTGTGTTGAATAT 720
cDNA(MLOC_17150)      -------------------------------------------------------------------------------- 217
CircularRNA           -------------------------------------------------------------------------------- -

                      740                 760                 780                 800
Genomic(MLOC_17150)   AACTGTTGGGTTTAAGTTGTTGCAAGATTCATTCTAATTTCTTGCCCGGCCGGTATCATTGGAATGTTACCTTCTCCTTT 800
cDNA(MLOC_17150)      -------------------------------------------------------------------------------- 217
CircularRNA           -------------------------------------------------------------------------------- -

                      820                 840                 860                 880
Genomic(MLOC_17150)   CTGCTTGATGGAACCTTTAGTTGTGCTTCTAATAAAGTTTTCTGCTAATGGCTTAACTTTGGGAAATATATGTACTTAAT 880
cDNA(MLOC_17150)      -------------------------------------------------------------------------------- 217
CircularRNA           -------------------------------------------------------------------------------- -

                      900                 920                 940                 960
Genomic(MLOC_17150)   GCTGGTTTTGCTTGCAGGACAAGGAGTTGGTTCAGCTTAAGCGTGAGGCCCGGATGAAGGGTGGGTTCTATGTCAGTCCT 960
cDNA(MLOC_17150)      ------------------ACAAGGAGTTGGTTCAGCTTAAGCGTGAGGCCCGGATGAAGGGTGGGTTCTATGTCAGTCCT 279
CircularRNA           -------------------------------------------------------------------------------- -

                      980                 1.000               1.020               1.040
Genomic(MLOC_17150)   GAGGCTAAGTTGCTGTTTGTTGTCCGAATCAGAGGGTAACTCGCACCACCTTGCCTCCCTGTATGTCATTGTATGTGTGT 1040
cDNA(MLOC_17150)      GAGGCTAAGTTGCTGTTTGTTGTCCGAATCAGAGG--------------------------------------------- 314
CircularRNA           -------------------------------------------------------------------------------- -

                      1.060               1.080               1.100               1.120
Genomic(MLOC_17150)   TGTGGTCATTTCTGGCTTAAGTTGACATAATTTTTGTTAATGTCTGGCTGCAGTATCAATGCCATGCACCCAAAAACCAA 1120
cDNA(MLOC_17150)      -----------------------------------------------------TATCAATGCCATGCACCCAAAAACCAA 341
CircularRNA           -------------------------------------------------------------------------------- -

                      1.140               1.160               1.180               1.200
Genomic(MLOC_17150)   GAAGATCCTCCAGCTTTTGCGTTTGAGACAGGTGGGTTGGCCAATTTGTTGATTGTGTCAAGTTTGAACTTGATTCATGC 1200
cDNA(MLOC_17150)      GAAGATCCTCCAGCTTTTGCGTTTGAGACAG------------------------------------------------- 372
CircularRNA           -------------------------------------------------------------------------------- -

                      1.220               1.240               1.260               1.280
Genomic(MLOC_17150)   GGCCATCTCTTGTTTGGATTGATTGCTACATGTGTTCATTGTGCCAATTGTTTGATTGTGCAGATATTCAATGGAGTGTT 1280
cDNA(MLOC_17150)      ---------------------------------------------------------------ATATTCAATGGAGTGTT 389
CircularRNA           -------------------------------------------------------------------------------- -

                      1.300               1.320               1.340               1.360
Genomic(MLOC_17150)   CCTCAAGGTTAACAAGGCCACCATCAACATGCTCCGCAGGGTCGAGCCATATGTTGCATATGGGTATGTTACAAGTGTTC 1360
cDNA(MLOC_17150)      CCTCAAGGTTAACAAGGCCACCATCAACATGCTCCGCAGGGTCGAGCCATATGTTGCATATGGGTA-------------- 455
CircularRNA           -------------------------------------------------------------------------------- -

                      1.380               1.400               1.420               1.440
Genomic(MLOC_17150)   AATTTTTACTTGATACATGCCTTGAACATTTGATGATTGTAATTGCTGTTATCATTATCCAACTTGTCTTGCTTGAACAA 1440
cDNA(MLOC_17150)      -------------------------------------------------------------------------------- 455
CircularRNA           -------------------------------------------------------------------------------- -

                      1.460               1.480               1.500               1.520
Genomic(MLOC_17150)   CTTTACAATGTGTGTAACACCATGTTAGAGCTTTTGACTGTTATGTTTTTATCCACTCCTGGAGGCTGGAAATTTGTAAC 1520
cDNA(MLOC_17150)      -------------------------------------------------------------------------------- 455
CircularRNA           -------------------------------------------------------------------------------- -

                      1.540               1.560               1.580               1.600
Genomic(MLOC_17150)   TTCATGAGTACTTAGTACAGTGACTTCATGAGTACTTAGTATCTGTTTGTAGCTAAACGTATAGAATCATCATCTTTGTA 1600
cDNA(MLOC_17150)      -------------------------------------------------------------------------------- 455
CircularRNA           -------------------------------------------------------------------------------- -
```

```
                        1.620               1.640               1.660               1.680
Genomic(MLOC_17150) GGTACCCGAACCTGAAGAGCGTGAGGGAGTTGATCTACAAGAGGGGTTATGGAAAGCTCAACAAGCAAAGGATTCCTCTG 1680
cDNA(MLOC_17150)    ----CCCGAACCTGAAGAGCGTGAGGGAGTTGATCTACAAGAGGGGTTATGGAAAGCTCAACAAGCAAAGGATTCCTCTG 531
CircularRNA         -----------CTGAAGAGCGTGAGGGAGTTGATCTACAAGAGGGGTTATGGAAAGCTCAACAAGCAAAGGATTCCTCTG 69

                        1.700               1.720               1.740               1.760
Genomic(MLOC_17150) ACTAACAACAAGGTCATCGAGGAGGTGAGCATTCCATTGCCCAACATATGGATCTCTACTTTGCATCTCCAAATGCTACC 1760
cDNA(MLOC_17150)    ACTAACAACAAGGTCATCGAGGAGG------------------------------------------------------- 556
CircularRNA         ACTAACAACAAGGTCATCGAGGAGG------------------------------------------------------- 94

                        1.780               1.800               1.820               1.840
Genomic(MLOC_17150) TGGGTTGATCCTGACCATTTTGTTGTCTTATTAACCAGGGTCTTGGAAAGCACAACATCATCTGCATTGAAGACGTTGTC 1840
cDNA(MLOC_17150)    ---------------------------------------GTCTTGGAAAGCACAACATCATCTGCATTGAAGACGTTGTC 597
CircularRNA         ---------------------------------------GTCTTGGAAAGCACAACATCATCTGCATTGAAGACGTTGTC 135

                        1.860               1.880               1.900               1.920
Genomic(MLOC_17150) CATGAGATCTTGACGGTTGGCCCTCACTTCAAGGAGGCCAACAACTTCCTGTGGCCATTCAAGCTCAAGGCGCCGCTGGG 1920
cDNA(MLOC_17150)    CATGAGATCTTGACGGTTGGCCCTCACTTCAAGGAGGCCAACAACTTCCTGTGGCCATTCAAGCTCAAGGCGCCGCTGGG 677
CircularRNA         CATGAGATCTTGACGGTTGGCCCTCACTTCAAGGAGGCCAACAACTTCCTGTGGCCATTCAAGCTCAAGGCGCCGCTGGG 215

                        1.940               1.960               1.980               2.000
Genomic(MLOC_17150) AGGCCTCAAGAAGAAGAGGAACCACTACGTCGAGGGTGGTGACGCCGGCAACCGTGAGGACTACATCAACCAGCTCGTCA 2000
cDNA(MLOC_17150)    AGGCCTCAAGAAGAAGAGGAACCACTACGTCGAGGGTGGTGACGCCGGCAACCGTGAGGACTACATCAACCAGCTCGTCA 757
CircularRNA         AGGCCTCAAGAAGAAGAGGAACCACTACGTCGAGGGTGGTGACGCCGGCAACCGTGAGGACTACATCAACCAGCTCGTCA 295

                        2.020               2.040               2.060               2.080
Genomic(MLOC_17150) GGAGGATGAACTAGGTTGATTGATCGATGGCGCCTTGTGTTTGGAAGATGTTTTGTTTGTTAAGCCCGCTTTACATTAGG 2080
cDNA(MLOC_17150)    GGAGGATGAACTAGGTTGATTGATCGATGGCGCCTTGTGTTTGGAAGATGTTTTGTTTGTTAAGCCCGCTTTACATTAGG 837
CircularRNA         GGAGGATGAACTAGGTTGATTGATCGATGGCGCCTTGTGTTTGGAAGATGTTTTGTTTGTTAAGCCCGCTTTACATTAGG 375

                        2.100               2.120               2.140               2.160
Genomic(MLOC_17150) AAGTGGATGGTGGATGCAGTTTTGATATTTCGGAACCATGATGCATGTTTGGAAGAATCTGACTCTGTATGTGGATGTTA 2160
cDNA(MLOC_17150)    AAGTGGATGGTGGATGCAGTTTTGATATTTCGGAACCATGATGCATGTTTGGAAGAATCTGACTCTGTATGTGGATGTTA 917
CircularRNA         AAGTGGATGGTGGATGCAGTTTTGATATTTCGGAACCATGATGCATGTTTGGAAGAAT---------------------- 433

                        2.180               2.200               2.220               2.240
Genomic(MLOC_17150) TGAGACGTATTTAATCTCCTCCTATGTTCTGAGAATACGTTTCTCGTTGTTGTCAGCCTAGTATGGCTGCCTTTGCTATG 2240
cDNA(MLOC_17150)    TGAGACGTATTTAATCTCCTCCTATGTTCTGAGAATACGTTTCTCGTTGTTGTCAGCCTAGTATGGCTGCCTTTGC---- 993
CircularRNA         -------------------------------------------------------------------------------- 433

                        2.260               2.280               2.300               2.320
Genomic(MLOC_17150) TCGCGGTTTTTGTTATTTCTTGTGAGCATAGTGTATTTCGAGTGATTCAGCTCAATACTTATCACTGGACGGGGCGCATA 2320
cDNA(MLOC_17150)    -------------------------------------------------------------------------------- 993
CircularRNA         -------------------------------------------------------------------------------- 433

                        2.340               2.360               2.380               2.400
Genomic(MLOC_17150) AATAGATTGATTTAGGAAGTGCTACCTCCGTTCCTAAATGCTCCCTTCGTCCCAAATTCTTGTCTAAGACAGAATTTTGG 2400
cDNA(MLOC_17150)    -------------------------------------------------------------------------------- 993
CircularRNA         -------------------------------------------------------------------------------- 433

                        2.420               2.440               2.460               2.480
Genomic(MLOC_17150) CACGGAGGAGTGTAGATACATTCATTTTTGTGACAAATAGGAGTATTTAGCTATGAAGGGAGGTACACACCCAGGGTCGG 2480
cDNA(MLOC_17150)    -------------------------------------------------------------------------------- 993
CircularRNA         -------------------------------------------------------------------------------- 433

                        2.500               2.520               2.540               2.560
Genomic(MLOC_17150) ATGAACCAACAACATCCAGGCAGTGGGAAAATGACAGCCTCCACATGATTCATGCTTCCACCAATAATAAATGTCCATGT 2560
cDNA(MLOC_17150)    -------------------------------------------------------------------------------- 993
CircularRNA         -------------------------------------------------------------------------------- 433

                        2.580               2.600               2.620               2.640
Genomic(MLOC_17150) TCCATAGAAGAAGTAATAAAAAAAATGTCCATGTGGTAGCTATATACCTAATGAGAGATTGATGTAGCATGTGCATGTTC 2640
cDNA(MLOC_17150)    -------------------------------------------------------------------------------- 993
CircularRNA         -------------------------------------------------------------------------------- 433

                        2.660               2.680               2.700               2.720
Genomic(MLOC_17150) ACAGTGGTAACAGTACATGTATGCTATGGAGACTATATATGATGCATCATCGAGCACTTGACTCGTCATCCCCATTGAAA 2720
cDNA(MLOC_17150)    -------------------------------------------------------------------------------- 993
CircularRNA         -------------------------------------------------------------------------------- 433

                        2.740               2.760               2.780               2.800
Genomic(MLOC_17150) TCCTCTAAAAAACTAGGATGATCAGTTGGCTAGTAGGTCAAACAACAACCAACCAATGAGCATAATCGGATTAGGAGGTA 2800
cDNA(MLOC_17150)    -------------------------------------------------------------------------------- 993
CircularRNA         -------------------------------------------------------------------------------- 433
```

Transducin/WD40 repeat-like protein_circular RNA (ID: Ch5:484562574-484563329)

GATAAGAAACCAGCTGTGAGGTGGGTGAAGAATGAGAAATTCGACGGAATCACATTGATTCAT
CATAGGGCTGCATCAACTGTGGAGTGGCATTCAAAGGGAGACTATTTCACGACAGTTGTGCCAA
CTGNNN…NNNGTGAATCAAGGGCTGTGCTGTTGCATCAGCTCTCCAAGAAGCATTCTCACCAT
CCTTTCCGTAAATTGCCGGGACTCCCTGTTGCAGCCGTATTCCATCCAACTCAAAAGATGTTCTT
TGTTGCCACTAAGAAGTTTGTTCAGGTTTATGATCTCCAAAAGGCAGAGGTAGTCAAAAAGCTGG
AGTCA**GGTCTCCGGGAAATTTCCTCTATCTCCATCCATCCTGGTGGT**

The nucleotides of junction-region are underlined. The nucleotides of junction-region which are supported by the junction-spaning sequencing reads are shown in red. Introns are not shown if the absence is supported by sequencing reads. In the absence of supporting sequencing reads, the intronic nucletides are shown as N.

Structural relationship between the circular RNA and its parental gene

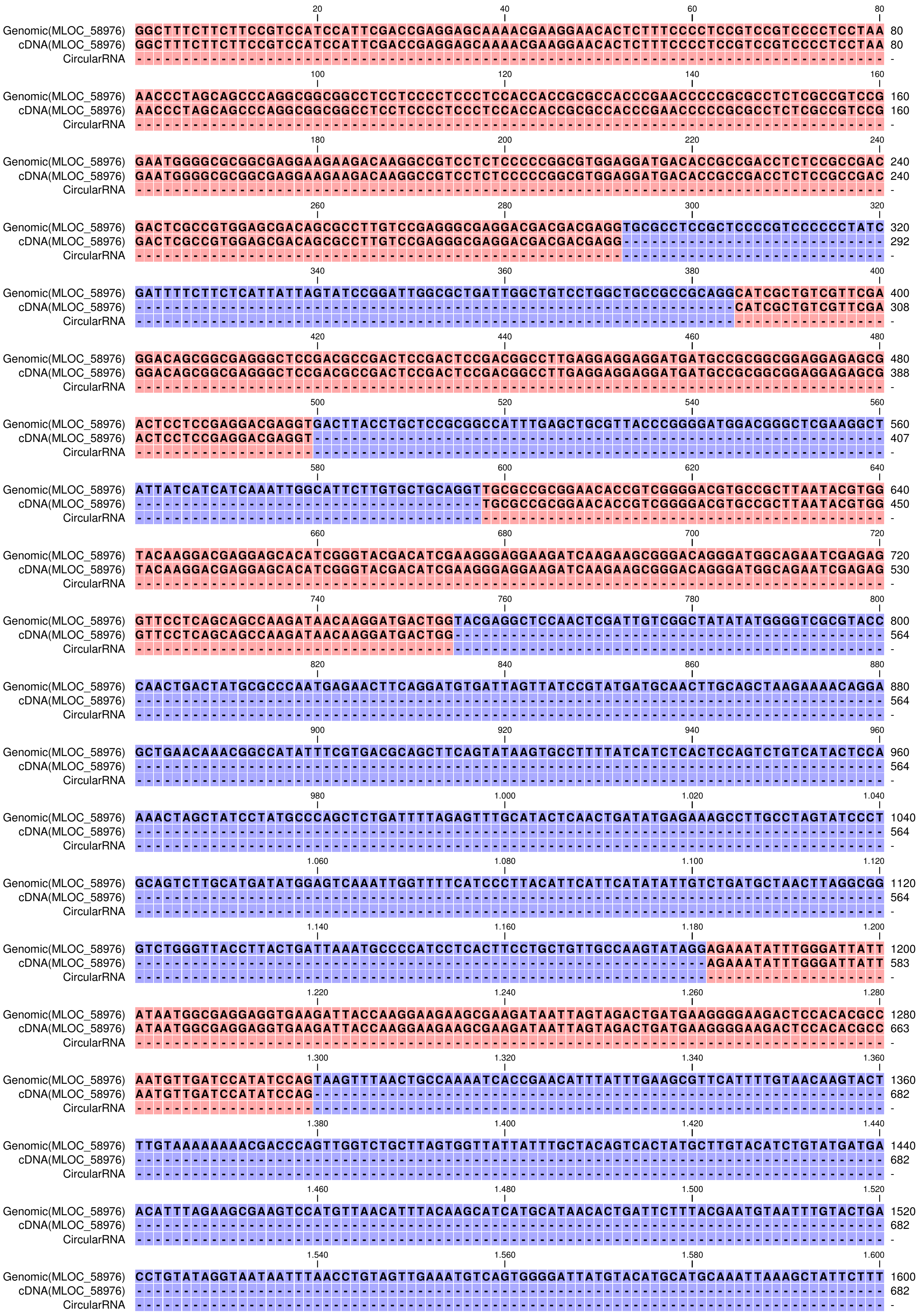
Genomic(MLOC_58976) GGCTTTCTTCTTCCGTCCATCCATTCGACCGAGGAGCAAAACGAAGGAACACTCTTTCCCCTCCGTCCGTCCCCTCCTAA 80
cDNA(MLOC_58976) GGCTTTCTTCTTCCGTCCATCCATTCGACCGAGGAGCAAAACGAAGGAACACTCTTTCCCCTCCGTCCGTCCCCTCCTAA 80
CircularRNA -
Genomic(MLOC_58976) AACCCTAGCAGCCCAGGCGGCGGCCTCCTCCCCTCCCTCCACCACCGCGCCACCCGAACCCCCGCGCCTCTCGCCGTCCG 160
cDNA(MLOC_58976) AACCCTAGCAGCCCAGGCGGCGGCCTCCTCCCCTCCCTCCACCACCGCGCCACCCGAACCCCCGCGCCTCTCGCCGTCCG 160
CircularRNA -
Genomic(MLOC_58976) GAATGGGGCGCGGCGAGGAAGAAGACAAGGCCGTCCTCTCCCCCGGCGTGGAGGATGACACCGCCGACCTCTCCGCCGAC 240
cDNA(MLOC_58976) GAATGGGGCGCGGCGAGGAAGAAGACAAGGCCGTCCTCTCCCCCGGCGTGGAGGATGACACCGCCGACCTCTCCGCCGAC 240
CircularRNA -
Genomic(MLOC_58976) GACTCGCCGTGGAGCGACAGCGCCTTGTCCGAGGGCGAGGACGACGACGAGGTGCGCCTCCGCTCCCCGTCCCCCCTATC 320
cDNA(MLOC_58976) GACTCGCCGTGGAGCGACAGCGCCTTGTCCGAGGGCGAGGACGACGACGAGG---------------------------- 292
CircularRNA -
Genomic(MLOC_58976) GATTTTCTTCTCATTATTAGTATCCGGATTGGCGCTGATTGGCTGTCCTGGCTGCCGCCGCAGGCATCGCTGTCGTTCGA 400
cDNA(MLOC_58976) --CATCGCTGTCGTTCGA 308
CircularRNA -
Genomic(MLOC_58976) GGACAGCGGCGAGGGCTCCGACGCCGACTCCGACTCCGACGGCCTTGAGGAGGAGGATGATGCCGCGGCGGAGGAGAGCG 480
cDNA(MLOC_58976) GGACAGCGGCGAGGGCTCCGACGCCGACTCCGACTCCGACGGCCTTGAGGAGGAGGATGATGCCGCGGCGGAGGAGAGCG 388
CircularRNA -
Genomic(MLOC_58976) ACTCCTCCGAGGACGAGGTGACTTACCTGCTCCGCGGCCATTTGAGCTGCGTTACCCGGGGATGGACGGGCTCGAAGGCT 560
cDNA(MLOC_58976) ACTCCTCCGAGGACGAGGT--- 407
CircularRNA -
Genomic(MLOC_58976) ATTATCATCATCAAATTGGCATTCTTGTGCTGCAGGTTGCGCCGCGGAACACCGTCGGGGACGTGCCGCTTAATACGTGG 640
cDNA(MLOC_58976) -------------------------------------TGCGCCGCGGAACACCGTCGGGGACGTGCCGCTTAATACGTGG 450
CircularRNA -
Genomic(MLOC_58976) TACAAGGACGAGGAGCACATCGGGTACGACATCGAAGGGAGGAAGATCAAGAAGCGGGACAGGGATGGCAGAATCGAGAG 720
cDNA(MLOC_58976) TACAAGGACGAGGAGCACATCGGGTACGACATCGAAGGGAGGAAGATCAAGAAGCGGGACAGGGATGGCAGAATCGAGAG 530
CircularRNA -
Genomic(MLOC_58976) GTTCCTCAGCAGCCAAGATAACAAGGATGACTGGTACGAGGCTCCAACTCGATTGTCGGCTATATATGGGGTCGCGTACC 800
cDNA(MLOC_58976) GTTCCTCAGCAGCCAAGATAACAAGGATGACTGG-- 564
CircularRNA -
Genomic(MLOC_58976) CAACTGACTATGCGCCCAATGAGAACTTCAGGATGTGATTAGTTATCCGTATGATGCAACTTGCAGCTAAGAAAACAGGA 880
cDNA(MLOC_58976) 564
CircularRNA -
Genomic(MLOC_58976) GCTGAACAAACGGCCATATTTCGTGACGCAGCTTCAGTATAAGTGCCTTTTATCATCTCACTCCAGTCTGTCATACTCCA 960
cDNA(MLOC_58976) 564
CircularRNA -
Genomic(MLOC_58976) AAACTAGCTATCCTATGCCCAGCTCTGATTTTAGAGTTTGCATACTCAACTGATATGAGAAAGCCTTGCCTAGTATCCCT 1040
cDNA(MLOC_58976) 564
CircularRNA -
Genomic(MLOC_58976) GCAGTCTTGCATGATATGGAGTCAAATTGGTTTTCATCCCTTACATTCATTCATATATTGTCTGATGCTAACTTAGGCGG 1120
cDNA(MLOC_58976) 564
CircularRNA -
Genomic(MLOC_58976) GTCTGGGTTACCTTACTGATTAAATGCCCCATCCTCACTTCCTGCTGTTGCCAAGTATAGGAGAAATATTTGGGATTATT 1200
cDNA(MLOC_58976) ---AGAAATATTTGGGATTATT 583
CircularRNA -
Genomic(MLOC_58976) ATAATGGCGAGGAGGTGAAGATTACCAAGGAAGAAGCGAAGATAATTAGTAGACTGATGAAGGGGAAGACTCCACACGCC 1280
cDNA(MLOC_58976) ATAATGGCGAGGAGGTGAAGATTACCAAGGAAGAAGCGAAGATAATTAGTAGACTGATGAAGGGGAAGACTCCACACGCC 663
CircularRNA -
Genomic(MLOC_58976) AATGTTGATCCATATCCAGTAAGTTTAACTGCCAAAATCACCGAACATTTATTTGAAGCGTTCATTTTGTAACAAGTACT 1360
cDNA(MLOC_58976) AATGTTGATCCATATCCAG--- 682
CircularRNA -
Genomic(MLOC_58976) TTGTAAAAAAAACGACCCAGTTGGTCTGCTTAGTGGTTATTATTTGCTACAGTCACTATGCTTGTACATCTGTATGATGA 1440
cDNA(MLOC_58976) 682
CircularRNA -
Genomic(MLOC_58976) ACATTTAGAAGCGAAGTCCATGTTAACATTTACAAGCATCATGCATAACACTGATTCTTTACGAATGTAATTTGTACTGA 1520
cDNA(MLOC_58976) 682
CircularRNA -
Genomic(MLOC_58976) CCTGTATAGGTAATAATTTAACCTGTAGTTGAAATGTCAGTGGGGATTATGTACATGCATGCAAATTAAAGCTATTCTTT 1600
cDNA(MLOC_58976) 682
CircularRNA -

```
                     1.620               1.640               1.660               1.680
Genomic(MLOC_58976)  ACAGTTCATGGTTTCTTTCATTTTCTCTACATAATGTTCAACACAGACCGAGTAAACTTCTCATGGCACTTTGCTTACTG 1680
cDNA(MLOC_58976)     -------------------------------------------------------------------------------- 682
CircularRNA          -------------------------------------------------------------------------------- -

                     1.700               1.720               1.740               1.760
Genomic(MLOC_58976)  ATTTGAATATGCTGATCATTGAAGTTATTTCTTTTACAATTTATGGTTTCCTTCCATTTTCTACACATGTAACTATTATG 1760
cDNA(MLOC_58976)     -------------------------------------------------------------------------------- 682
CircularRNA          -------------------------------------------------------------------------------- -

                     1.780               1.800               1.820               1.840
Genomic(MLOC_58976)  CTCAACATCGGCTGAGTAAACTTTTCTCGTAGGATTATGTTGACTGGTTTGAATATGCTGATAAAGGCCATCCACTTTCT 1840
cDNA(MLOC_58976)     ---------------------------------ATTATGTTGACTGGTTTGAATATGCTGATAAAGGCCATCCACTTTCT 729
CircularRNA          -------------------------------------------------------------------------------- -

                     1.860               1.880               1.900               1.920
Genomic(MLOC_58976)  AACGCTCCTGAGCCAAAGAGGCGATTTGTGCCCTCAAAGTGGGAGCAAAAGAAGGTATATCTCCAGTTTCCTTATCTTTT 1920
cDNA(MLOC_58976)     AACGCTCCTGAGCCAAAGAGGCGATTTGTGCCCTCAAAGTGGGAGCAAAAGAAGGT------------------------ 785
CircularRNA          -------------------------------------------------------------------------------- -

                     1.940               1.960               1.980               2.000
Genomic(MLOC_58976)  ATTTTATGCTAGTGCTTTTGATAAAATCTACATGCTCTTTAATTTCTAATAAGATAGAACTAACTGTCTGGATGTCTTGT 2000
cDNA(MLOC_58976)     -------------------------------------------------------------------------------- 785
CircularRNA          -------------------------------------------------------------------------------- -

                     2.020               2.040               2.060               2.080
Genomic(MLOC_58976)  TAATCTGTTTGTTCGAGATCTGAAGTATCTTGCTTAAGAAAAGAAATACCCTATTGAAATATGTTTTATACAGAATGGTC 2080
cDNA(MLOC_58976)     -------------------------------------------------------------------------------- 785
CircularRNA          -------------------------------------------------------------------------------- -

                     2.100               2.120               2.140               2.160
Genomic(MLOC_58976)  TAAGAAGTTTGTGAGACAGTGGACTGATGATTGTTGGCAGGCCATGGGTGCCTGCTTTAATAACCCAGTTCATGATTGTG 2160
cDNA(MLOC_58976)     -------------------------------------------------------------------------------- 785
CircularRNA          -------------------------------------------------------------------------------- -

                     2.180               2.200               2.220               2.240
Genomic(MLOC_58976)  AGGTGTTTCTAGCTTTTATATTTTTGCTGTCTGCCTGCGAGTGTTTACTGTTTAGATTTTAGATTGCGCTTTTCTAATTT 2240
cDNA(MLOC_58976)     -------------------------------------------------------------------------------- 785
CircularRNA          -------------------------------------------------------------------------------- -

                     2.260               2.280               2.300               2.320
Genomic(MLOC_58976)  ATAGCATGTTGCAGCAAGGGATTGATAATGTTAGTTTAACTTCCACTGTTGTTCGGTTGCCTAGATTTTTGTGGGCAACC 2320
cDNA(MLOC_58976)     -------------------------------------------------------------------------------- 785
CircularRNA          -------------------------------------------------------------------------------- -

                     2.340               2.360               2.380               2.400
Genomic(MLOC_58976)  ACTGAATAAGACCATGTGATATTAGTACTCCATAAACAGACTTAGTAGCCATATGGCTAATTTTTAGGTACCATCTGAAA 2400
cDNA(MLOC_58976)     -------------------------------------------------------------------------------- 785
CircularRNA          -------------------------------------------------------------------------------- -

                     2.420               2.440               2.460               2.480
Genomic(MLOC_58976)  CTGCTTTGGATTCTTGTTGTATATGCTTTCTTGTTGGCAAGAATTTTAGATTATATGCTGTTAATACGGATCAAATAACC 2480
cDNA(MLOC_58976)     -------------------------------------------------------------------------------- 785
CircularRNA          -------------------------------------------------------------------------------- -

                     2.500               2.520               2.540               2.560
Genomic(MLOC_58976)  TCAATTTCCAGAGGATTTATTTGTTAGTTTCTTTCTGAAACTGCTTTGGAATTGCATTTGTGCTATCACGTTTCTTGCCT 2560
cDNA(MLOC_58976)     -------------------------------------------------------------------------------- 785
CircularRNA          -------------------------------------------------------------------------------- -

                     2.580               2.600               2.620               2.640
Genomic(MLOC_58976)  GCCATTTATAACTAATAATTAGTTGGCATCGCTATTTACTATATCTTTTACCTCATTGTTTTTCTTTATGGCAGGTTGTT 2640
cDNA(MLOC_58976)     ----------------------------------------------------------------------------TGTT 789
CircularRNA          -------------------------------------------------------------------------------- -

                     2.660               2.680               2.700               2.720
Genomic(MLOC_58976)  AAACTTGTTAGAGCCATTCGTAAAGGATGGATCAAGTTTGACAAGCCAAAGGATGAACCAAACTATTATCTCTTGTGGGG 2720
cDNA(MLOC_58976)     AAACTTGTTAGAGCCATTCGTAAAGGATGGATCAAGTTTGACAAGCCAAAGGATGAACCAAACTATTATCTCTTGTGGGG 869
CircularRNA          -------------------------------------------------------------------------------- -

                     2.740               2.760               2.780               2.800
Genomic(MLOC_58976)  AGATGACACTGATACAGCAGACAATAAGCGGCAAGGCTTGAGCTACATTCCTGCACCTAAACCAAATTTGCCAGGTACCA 2800
cDNA(MLOC_58976)     AGATGACACTGATACAGCAGACAATAAGCGGCAAGGCTTGAGCTACATTCCTGCACCTAAACCAAATTTGCCAGGT---- 945
CircularRNA          -------------------------------------------------------------------------------- -

                     2.820               2.840               2.860               2.880
Genomic(MLOC_58976)  TGCTATCATTTTGCAAGTCCTGTCATGCTTATTTGAAAAGCTATCCAGCAATCTATTTGACCTGATGACCTCCCATTCTG 2880
cDNA(MLOC_58976)     -------------------------------------------------------------------------------- 945
CircularRNA          -------------------------------------------------------------------------------- -

                     2.900               2.920               2.940               2.960
Genomic(MLOC_58976)  TTGGAATGATGTGAAACAGTTTTGCACTGGGCATTTCTGTTCCTTAGTTGATGTTCTATTCATTCTGTATGTTGCTATTA 2960
cDNA(MLOC_58976)     -------------------------------------------------------------------------------- 945
CircularRNA          -------------------------------------------------------------------------------- -

                     2.980               3.000               3.020               3.040
Genomic(MLOC_58976)  ATTTTTTCTATTCAGGTCATGAGGAGTCGTATAATCCTTCTGTTGAATATATTCCCACACAAGAGGAGATAGATAAATAC 3040
cDNA(MLOC_58976)     -----------------CATGAGGAGTCGTATAATCCTTCTGTTGAATATATTCCCACACAAGAGGAGATAGATAAATAC 1008
CircularRNA          -------------------------------------------------------------------------------- -

                     3.060               3.080               3.100               3.120
Genomic(MLOC_58976)  CAGCTTATGTATGAGGAGGACCGGCCAAAATTCATCCCACGACGGTATAATTTTTTATTGTCATTATGATTTTTCTTATG 3120
cDNA(MLOC_58976)     CAGCTTATGTATGAGGAGGACCGGCCAAAATTCATCCCACGACG------------------------------------ 1052
CircularRNA          -------------------------------------------------------------------------------- -

                     3.140               3.160               3.180               3.200
Genomic(MLOC_58976)  CATGGTCTGATTCGTCTGAGGAAATTTAGTTATGTGGCCCCTTTATTTTGTGTCGAGTATGCAGATTCGAATCTCTTAGA 3200
cDNA(MLOC_58976)     ----------------------------------------------------------------ATTCGAATCTCTTAGA 1068
CircularRNA          -------------------------------------------------------------------------------- -
```

```
                          3.220               3.240               3.260               3.280
Genomic(MLOC_58976)  AGTGTGCCTGCCTACGAGAAGGCACTTAGGGAAGGATTTGATCGGTGCCTAGATCTTTATCTATGTCCTAGAACTCGCAA 3280
cDNA(MLOC_58976)     AGTGTGCCTGCCTACGAGAAGGCACTTAGGGAAGGATTTGATCGGTGCCTAGATCTTTATCTATGTCCTAGAACTCGCAA 1148
CircularRNA          -------------------------------------------------------------------------------- -

                          3.300               3.320               3.340               3.360
Genomic(MLOC_58976)  GAAGCGTGTAAGTCAAGGATCTATCTTCTTTTCATAACTATATATAAGGGCCTTTATTGCCTGGATTCTAGTTCATCATT 3360
cDNA(MLOC_58976)     GAAGCGT------------------------------------------------------------------------- 1155
CircularRNA          -------------------------------------------------------------------------------- -

                          3.380               3.400               3.420               3.440
Genomic(MLOC_58976)  AAACATCGTGATCTTCTTTTCAATTCATGATGATTATTTGACAATACCACAGATAAACATTGATCCTGAGTCACTCAGGC 3440
cDNA(MLOC_58976)     ----------------------------------------------------ATAAACATTGATCCTGAGTCACTCAGGC 1183
CircularRNA          -------------------------------------------------------------------------------- -

                          3.460               3.480               3.500               3.520
Genomic(MLOC_58976)  CAAAGTTACCTAGTAAAAAGGATCTGAGGCCATATCCACGAACTTGTTACCTTGAGTTCAAGGGCCATGGTGGTCCAGTG 3520
cDNA(MLOC_58976)     CAAAGTTACCTAGTAAAAAGGATCTGAGGCCATATCCACGAACTTGTTACCTTGAGTTCAAGGGCCATGGTGGTCCAGTG 1263
CircularRNA          -------------------------------------------------------------------------------- -

                          3.540               3.560               3.580               3.600
Genomic(MLOC_58976)  ACGTCGCTGTCAGTTGAAACAACAGGCCAGTGGATAGCATCAGGTATAGACTGTAGATATTTATTTTCTATGTTCTTCCT 3600
cDNA(MLOC_58976)     ACGTCGCTGTCAGTTGAAACAACAGGCCAGTGGATAGCATC--------------------------------------- 1304
CircularRNA          -------------------------------------------------------------------------------- -

                          3.620               3.640               3.660               3.680
Genomic(MLOC_58976)  GAATATATATGATAATGTTCTGATAGTTCAAATGGTAATGAACTCAATGTTATTAGTCAATTTGCTTTGAACTCCGATGT 3680
cDNA(MLOC_58976)     -------------------------------------------------------------------------------- 1304
CircularRNA          -------------------------------------------------------------------------------- -

                          3.700               3.720               3.740               3.760
Genomic(MLOC_58976)  TTGGTCAGACCATTTCATAGAAGACACTTGGCACTTTCAATGTAACTAACTTTATTAGTATGCATGATATACAGAAGTAC 3760
cDNA(MLOC_58976)     -------------------------------------------------------------------------------- 1304
CircularRNA          -------------------------------------------------------------------------------- -

                          3.780               3.800               3.820               3.840
Genomic(MLOC_58976)  TCCCTCCGTCCGGAATTACTTGTCACCAAAATGGAAAGAAATGGATGTATCTAGAATTAAAATATGTCTAGATGCATCCA 3840
cDNA(MLOC_58976)     -------------------------------------------------------------------------------- 1304
CircularRNA          -------------------------------------------------------------------------------- -

                          3.860               3.880               3.900               3.920
Genomic(MLOC_58976)  TTTCTATGACAAGTAATTCCGGACGGAGGGAGTAGTAAATGACAGTGCTGTAAAACATAACTGTTGTGCCAAATATCATT 3920
cDNA(MLOC_58976)     -------------------------------------------------------------------------------- 1304
CircularRNA          -------------------------------------------------------------------------------- -

                          3.940               3.960               3.980               4.000
Genomic(MLOC_58976)  GATTTAATCGCATGATTCCATAAGAGGGTCTTATGTTTGTACAATGATGAGGAATTGGACAAGATAAATCCGTGAACAGG 4000
cDNA(MLOC_58976)     -------------------------------------------------------------------------------- 1304
CircularRNA          -------------------------------------------------------------------------------- -

                          4.020               4.040               4.060               4.080
Genomic(MLOC_58976)  CTCTTTTGCTCCATGAAATGATCTGATCAACTTGAGCATGGCACTGTCTTATATTACAGTTCTTCTTTGCTGAAGGTTCA 4080
cDNA(MLOC_58976)     -------------------------------------------------------------------------AGGTTCA 1311
CircularRNA          -------------------------------------------------------------------------------- -

                          4.100               4.120               4.140               4.160
Genomic(MLOC_58976)  TCTGATGGAACAATTCGTGTTTGGGAGGTTGAAACTGGTCGCTGTCTTAAGGTTTTCAATGTTGGTGCTGATGTCCATCA 4160
cDNA(MLOC_58976)     TCTGATGGAACAATTCGTGTTTGGGAGGTTGAAACTGGTCGCTGTCTTAAGGTTTTCAATGTTGGTGCTGATGTCCATCA 1391
CircularRNA          -------------------------------------------------------------------------------- -

                          4.180               4.200               4.220               4.240
Genomic(MLOC_58976)  TATTTCGTGGAATCCTTCACCTGAGAGGCCCATTCTGGCTGCTATTGTGTATGCCTTCGCTATTCACAAGTATACTTCAT 4240
cDNA(MLOC_58976)     TATTTCGTGGAATCCTTCACCTGAGAGGCCCATTCTGGCTGCTATTGT-------------------------------- 1439
CircularRNA          -------------------------------------------------------------------------------- -

                          4.260               4.280               4.300               4.320
Genomic(MLOC_58976)  TTTTTGGCGTTACACTTTTTCATCATTCATCTTATTTTGTGCAGTGGCCGTGATCTTCTACTTCTTAACGCTGAGGTGGG 4320
cDNA(MLOC_58976)     --------------------------------------------TGGCCGTGATCTTCTACTTCTTAACGCTGAGGTGGG 1475
CircularRNA          -------------------------------------------------------------------------------- -

                          4.340               4.360               4.380               4.400
Genomic(MLOC_58976)  GGATGAGGAAACTCAAGTGAGGACAAAACAGCTCCTCCATGTCGAGGAACAGACCCCTGAAGATGATGGTAATAGTAGTT 4400
cDNA(MLOC_58976)     GGATGAGGAAACTCAAGTGAGGACAAAACAGCTCCTCCATGTCGAGGAACAGACCCCTGAAGATGATGGT---------- 1545
CircularRNA          -------------------------------------------------------------------------------- -

                          4.420               4.440               4.460               4.480
Genomic(MLOC_58976)  GTCACTCAAACAATTCTTCCACCTACCTTATTCACTGTGTTCTTAACTATTTAATTATATTGCAGGTGATAAGAAACCAG 4480
cDNA(MLOC_58976)     -------------------------------------------------------------------GATAAGAAACCAG 1558
CircularRNA          -------------------------------------------------------------------GATAAGAAACCAG 13

                          4.500               4.520               4.540               4.560
Genomic(MLOC_58976)  CTGTGAGGTGGGTGAAGAATGAGAAATTCGACGGAATCACATTGATTCATCATAGGGTATAATACCTGAGGGTTACTTAT 4560
cDNA(MLOC_58976)     CTGTGAGGTGGGTGAAGAATGAGAAATTCGACGGAATCACATTGATTCATCATAGGG----------------------- 1615
CircularRNA          CTGTGAGGTGGGTGAAGAATGAGAAATTCGACGGAATCACATTGATTCATCATAGGG----------------------- 70

                          4.580               4.600               4.620               4.640
Genomic(MLOC_58976)  CATTCTCCTAAATGCGTAGGTTATTGTTCGCATGCTTACATCTGTTCTATTGTTCAGGCTGCATCAACTGTGGAGTGGCA 4640
cDNA(MLOC_58976)     ----------------------------------------------------------CTGCATCAACTGTGGAGTGGCA 1637
CircularRNA          ----------------------------------------------------------CTGCATCAACTGTGGAGTGGCA 92

                          4.660               4.680               4.700               4.720
Genomic(MLOC_58976)  TTCAAAGGGAGACTATTTCACGACAGTTGTGCCAACTGATATCCTGTTTGCTTTGTTTAATTTATAAGTGATGCTTGAAC 4720
cDNA(MLOC_58976)     TTCAAAGGGAGACTATTTCACGACAGTTGTGCCAACTG------------------------------------------ 1675
CircularRNA          TTCAAAGGGAGACTATTTCACGACAGTTGTGCCAACTGNNNNNNNNNNNNNNNNNNNNNNNNNNNNNNNNNNNNNNNNNN 172

                          4.740               4.760               4.780               4.800
Genomic(MLOC_58976)  ACAATAACCAATATGTTTAAATCTTGGAGCTGCCATGCAACATTCTAACTATATCAAGATAATCATTTGCGATGGTTCTA 4800
cDNA(MLOC_58976)     -------------------------------------------------------------------------------- 1675
CircularRNA          NNNNNNNNNNNNNNNNNNNNNNNNNNNNNNNNNNNNNNNNNNNNNNNNNNNNNNNNNNNNNNNNNNNNNNNNNNNNNNNN 252
```

```
                                         4.820                         4.840                         4.860                         4.880
Genomic(MLOC_58976) TAGTGTGTGCTGCAGTAAAATTGGCTTTTCTTTCTTTGTTTCAGGGCTATGTGCTTTTCTTTCTTTATTGGGTGTCACTC 4880
   cDNA(MLOC_58976) -------------------------------------------------------------------------------- 1675
        CircularRNA NNNNNNNNNNNNNNNNNNNNNNNNNNNNNNNNNNNNNNNNNNNNNNNNNNNNNNNNNNNNNNNNNNNNNNNNNNNNNNNN 332
                                         4.900                         4.920                         4.940                         4.960
Genomic(MLOC_58976) TTTCAATTCATAGTTACTGTAGATATGCATCCCCGCCAACATATAGTGTGTACACAGATTCTTCACATTTTGAATATCTA 4960
   cDNA(MLOC_58976) -------------------------------------------------------------------------------- 1675
        CircularRNA NNNNNNNNNNNNNNNNNNNNNNNNNNNNNNNNNNNNNNNNNNNNNNNNNNNNNNNNNNNNNNNNNNNNNNNNNNNNNNNN 412
                                         4.980                         5.000                         5.020                         5.040
Genomic(MLOC_58976) TTCTTCTGTTTGTCTTCCTTAATTCCTCTGTACGTGAATCAAGGGCTGTGCTGTTGCATCAGCTCTCCAAGAAGCATTCT 5040
   cDNA(MLOC_58976) ---------------------------------GTGAATCAAGGGCTGTGCTGTTGCATCAGCTCTCCAAGAAGCATTCT 1722
        CircularRNA NNNNNNNNNNNNNNNNNNNNNNNNNNNNNNNNNGTGAATCAAGGGCTGTGCTGTTGCATCAGCTCTCCAAGAAGCATTCT 492
                                         5.060                         5.080                         5.100                         5.120
Genomic(MLOC_58976) CACCATCCTTTCCGTAAATTGCCGGGACTCCCTGTTGCAGCCGTATTCCATCCAACTCAAAAGATGTTCTTTGTTGCCAC 5120
   cDNA(MLOC_58976) CACCATCCTTTCCGTAAATTGCCGGGACTCCCTGTTGCAGCCGTATTCCATCCAACTCAAAAGATGTTCTTTGTTGCCAC 1802
        CircularRNA CACCATCCTTTCCGTAAATTGCCGGGACTCCCTGTTGCAGCCGTATTCCATCCAACTCAAAAGATGTTCTTTGTTGCCAC 572
                                         5.140                         5.160                         5.180                         5.200
Genomic(MLOC_58976) TAAGAAGTTTGTTCAGGTTTATGATCTCCAAAAGGCAGAGGTAGTCAAAAAGCTGGAGTCAGGTCTCCGGGAAATTTCCT 5200
   cDNA(MLOC_58976) TAAGAAGTTTGTTCAGGTTTATGATCTCCAAAAGGCAGAGGTAGTCAAAAAGCTGGAGTCAGGTCTCCGGGAAATTTCCT 1882
        CircularRNA TAAGAAGTTTGTTCAGGTTTATGATCTCCAAAAGGCAGAGGTAGTCAAAAAGCTGGAGTCAGGTCTCCGGGAAATTTCCT 652
                                         5.220                         5.240                         5.260                         5.280
Genomic(MLOC_58976) CTATCTCCATCCATCCTGGTGGTACGTATAAAATCAACTTGATTACTAATTTACCAGTTGATAAATCTTGAGTTGCTTCA 5280
   cDNA(MLOC_58976) CTATCTCCATCCATCCTGGTGGT--------------------------------------------------------- 1905
        CircularRNA CTATCTCCATCCATCCTGGTGGT--------------------------------------------------------- 675
                                         5.300                         5.320                         5.340                         5.360
Genomic(MLOC_58976) TATTGCCTTGCTATTCAAAGCGCTGTGAACCAGTTGTTAAATTAATGGTAGTGCGACACTGTTTGTTGAATTGCCTTATT 5360
   cDNA(MLOC_58976) -------------------------------------------------------------------------------- 1905
        CircularRNA -------------------------------------------------------------------------------- 675
                                         5.380                         5.400                         5.420                         5.440
Genomic(MLOC_58976) AATGGCATTGCAATTCTTTCGTGCAGGTGATAATGTTATTGTGGGAAGTAAAGATGGCAAATTATGTTGGTTTGACACTG 5440
   cDNA(MLOC_58976) ----------------------------GATAATGTTATTGTGGGAAGTAAAGATGGCAAATTATGTTGGTTTGACACTG 1957
        CircularRNA -------------------------------------------------------------------------------- 675
                                         5.460                         5.480                         5.500                         5.520
Genomic(MLOC_58976) ATCTATCTACAAGACCATACAAGATGTTAAAGTACGACTTCTGTAAATTGCTAGTGTTAGCGTTTTAGGCAGTTGGTTGC 5520
   cDNA(MLOC_58976) ATCTATCTACAAGACCATACAAGATGTTAAAG------------------------------------------------ 1989
        CircularRNA -------------------------------------------------------------------------------- 675
                                         5.540                         5.560                         5.580                         5.600
Genomic(MLOC_58976) TACTCCCCCCGTCCTGAATTACTTCTTTTAGTGTTAGGTACATCCGTATCTAGAAAAATCTAAGACAAGTAATTTGGGAT 5600
   cDNA(MLOC_58976) -------------------------------------------------------------------------------- 1989
        CircularRNA -------------------------------------------------------------------------------- 675
                                         5.620                         5.640                         5.660                         5.680
Genomic(MLOC_58976) GGAAGTAATATTATTTTAGCTTTTCTTTGTTCAAATTTGGTTTGGCCAACTTAACACTTTCTATGTACTTCCTCGGTTCC 5680
   cDNA(MLOC_58976) -------------------------------------------------------------------------------- 1989
        CircularRNA -------------------------------------------------------------------------------- 675
                                         5.700                         5.720                         5.740                         5.760
Genomic(MLOC_58976) ATAAGTCTTATTGTGGTTTCAGTTGAAAATTTGAACTAAAACCACAACAGGAATTATGGAACGGAGGGAGTACCAATTAG 5760
   cDNA(MLOC_58976) -------------------------------------------------------------------------------- 1989
        CircularRNA -------------------------------------------------------------------------------- 675
                                         5.780                         5.800                         5.820                         5.840
Genomic(MLOC_58976) TAAGTACTTTAATAACAATGCACAGTATGGGCCACATGCTTTTCTATCTGATATGGCCACCTATTTTAATCAAGAAACAG 5840
   cDNA(MLOC_58976) -------------------------------------------------------------------------------- 1989
        CircularRNA -------------------------------------------------------------------------------- 675
                                         5.860                         5.880                         5.900                         5.920
Genomic(MLOC_58976) TACATGCCACATGCTTTGCTATCTGATGCGGCCACAGATGATTCCTTTATGGTAGCCTTCAAAGTCATGCCGACCTTTTC 5920
   cDNA(MLOC_58976) -------------------------------------------------------------------------------- 1989
        CircularRNA -------------------------------------------------------------------------------- 675
                                         5.940                         5.960                         5.980                         6.000
Genomic(MLOC_58976) CTTCTCGACATCACCGCTATGCGCAAGTGTTTATGATTTACACTGCTTAGTCTAGTTTTTTTTGAATGGTTTGTACTGTA 6000
   cDNA(MLOC_58976) -------------------------------------------------------------------------------- 1989
        CircularRNA -------------------------------------------------------------------------------- 675
                                         6.020                         6.040                         6.060                         6.080
Genomic(MLOC_58976) AAAAAATTCATGTTTTTTCCGTTTCTAGTTACTGCCTTGTTTTAAAAAAAAAACCATTAGATACAATTGTTTTTTTGGAT 6080
   cDNA(MLOC_58976) -------------------------------------------------------------------------------- 1989
        CircularRNA -------------------------------------------------------------------------------- 675
                                         6.100                         6.120                         6.140                         6.160
Genomic(MLOC_58976) AGTACCCACTAGTTATATTTATACTGTTCTGTATGAGTTTATGTTTTGTATTTTATTTATTTCAGGAACCACAAGAAGGA 6160
   cDNA(MLOC_58976) ------------------------------------------------------------------AACCACAAGAAGGA 2003
        CircularRNA -------------------------------------------------------------------------------- 675
                                         6.180                         6.200                         6.220                         6.240
Genomic(MLOC_58976) TATAACCAGTGTTACTTTCCACCGGAGATACCCTCTTTTTGCCTCGTCATCGGAGGATTGTACAGCTCATGTGTTCCATG 6240
   cDNA(MLOC_58976) TATAACCAGTGTTACTTTCCACCGGAGATACCCTCTTTTTGCCTCGTCATCGGAGGATTGTACAGCTCATGTGTTCCATG 2083
        CircularRNA -------------------------------------------------------------------------------- 675
                                         6.260                         6.280                         6.300                         6.320
Genomic(MLOC_58976) GGATGGTTTATGCTGATCTCAACCAGAACCCACTTATTGTACCATTGGAGATTCTTCGTGGCCATTTGAGTTCAGATAGA 6320
   cDNA(MLOC_58976) GGATGGTTTATGCTGATCTCAACCAGAACCCACTTATTGTACCATTGGAGATTCTTCGTGGCCATTTGAGTTCAGATAGA 2163
        CircularRNA -------------------------------------------------------------------------------- 675
                                         6.340                         6.360                         6.380                         6.400
Genomic(MLOC_58976) AGAGGTATTTTGTCATACAACATGTGGTTTCTATGATTTGCATTATTTTACATCTTTTGTTGATTCTTATTTGCACTCAT 6400
   cDNA(MLOC_58976) AGAGG--------------------------------------------------------------------------- 2168
        CircularRNA -------------------------------------------------------------------------------- 675
```

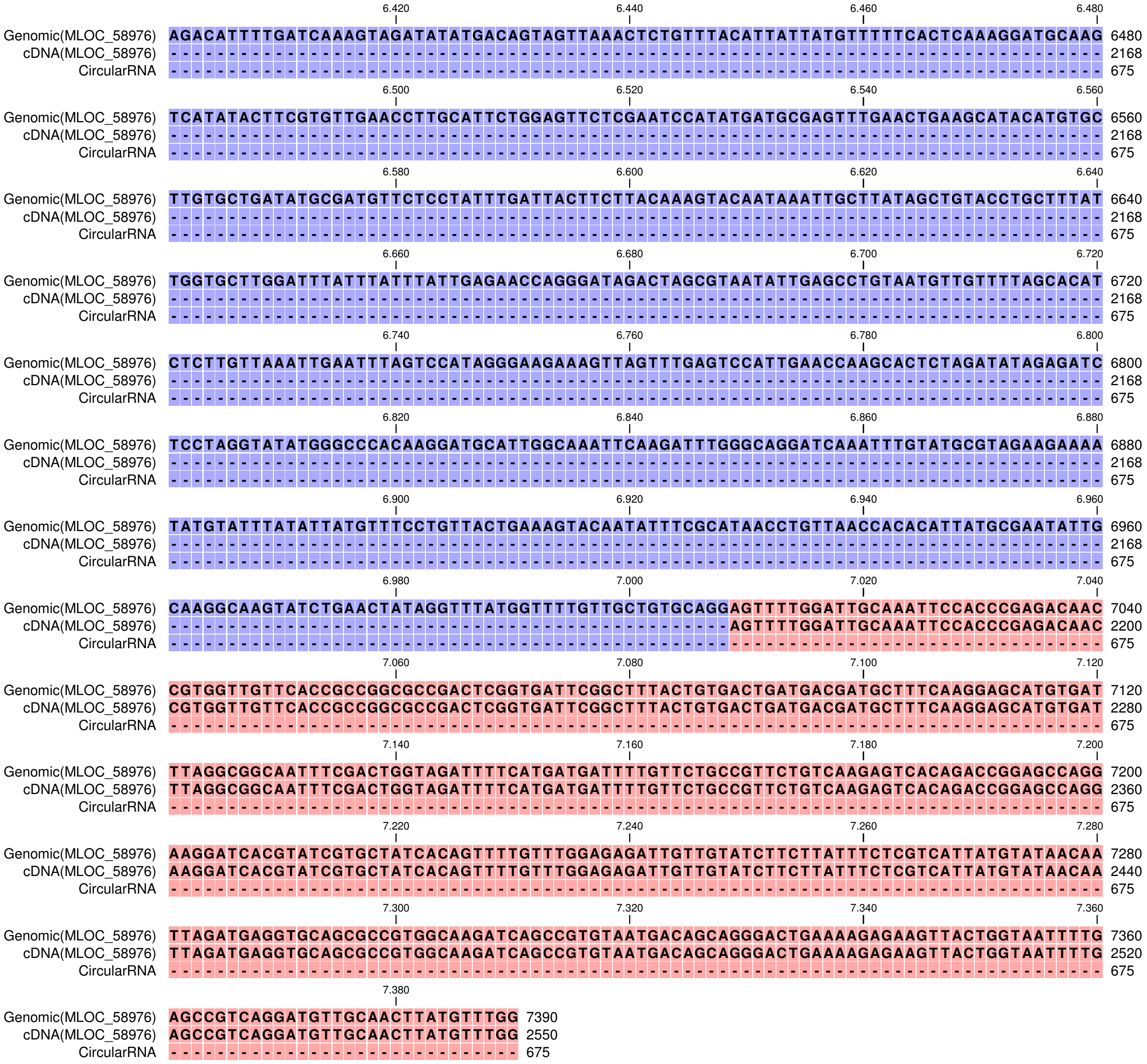
6.420 6.440 6.460 6.480
Genomic(MLOC_58976) AGACATTTTGATCAAAGTAGATATATGACAGTAGTTAAACTCTGTTTACATTATTATGTTTTTCACTCAAAGGATGCAAG 6480
cDNA(MLOC_58976) 2168
CircularRNA 675
6.500 6.520 6.540 6.560
Genomic(MLOC_58976) TCATATACTTCGTGTTGAACCTTGCATTCTGGAGTTCTCGAATCCATATGATGCGAGTTTGAACTGAAGCATACATGTGC 6560
cDNA(MLOC_58976) 2168
CircularRNA 675
6.580 6.600 6.620 6.640
Genomic(MLOC_58976) TTGTGCTGATATGCGATGTTCTCCTATTTGATTACTTCTTACAAAGTACAATAAATTGCTTATAGCTGTACCTGCTTTAT 6640
cDNA(MLOC_58976) 2168
CircularRNA 675
6.660 6.680 6.700 6.720
Genomic(MLOC_58976) TGGTGCTTGGATTTATTTATTTATTGAGAACCAGGGATAGACTAGCGTAATATTGAGCCTGTAATGTTGTTTTAGCACAT 6720
cDNA(MLOC_58976) 2168
CircularRNA 675
6.740 6.760 6.780 6.800
Genomic(MLOC_58976) CTCTTGTTAAATTGAATTTAGTCCATAGGGAAGAAAGTTAGTTTGAGTCCATTGAACCAAGCACTCTAGATATAGAGATC 6800
cDNA(MLOC_58976) 2168
CircularRNA 675
6.820 6.840 6.860 6.880
Genomic(MLOC_58976) TCCTAGGTATATGGGCCCACAAGGATGCATTGGCAAATTCAAGATTTGGGCAGGATCAAATTTGTATGCGTAGAAGAAAA 6880
cDNA(MLOC_58976) 2168
CircularRNA 675
6.900 6.920 6.940 6.960
Genomic(MLOC_58976) TATGTATTTATATTATGTTTCCTGTTACTGAAAGTACAATATTTCGCATAACCTGTTAACCACACATTATGCGAATATTG 6960
cDNA(MLOC_58976) 2168
CircularRNA 675
6.980 7.000 7.020 7.040
Genomic(MLOC_58976) CAAGGCAAGTATCTGAACTATAGGTTTATGGTTTTGTTGCTGTGCAGGAGTTTTGGATTGCAAATTCCACCCGAGACAAC 7040
cDNA(MLOC_58976) AGTTTTGGATTGCAAATTCCACCCGAGACAAC 2200
CircularRNA 675
7.060 7.080 7.100 7.120
Genomic(MLOC_58976) CGTGGTTGTTCACCGCCGGCGCCGACTCGGTGATTCGGCTTTACTGTGACTGATGACGATGCTTTCAAGGAGCATGTGAT 7120
cDNA(MLOC_58976) CGTGGTTGTTCACCGCCGGCGCCGACTCGGTGATTCGGCTTTACTGTGACTGATGACGATGCTTTCAAGGAGCATGTGAT 2280
CircularRNA 675
7.140 7.160 7.180 7.200
Genomic(MLOC_58976) TTAGGCGGCAATTTCGACTGGTAGATTTTCATGATGATTTTGTTCTGCCGTTCTGTCAAGAGTCACAGACCGGAGCCAGG 7200
cDNA(MLOC_58976) TTAGGCGGCAATTTCGACTGGTAGATTTTCATGATGATTTTGTTCTGCCGTTCTGTCAAGAGTCACAGACCGGAGCCAGG 2360
CircularRNA 675
7.220 7.240 7.260 7.280
Genomic(MLOC_58976) AAGGATCACGTATCGTGCTATCACAGTTTTGTTTGGAGAGATTGTTGTATCTTCTTATTTCTCGTCATTATGTATAACAA 7280
cDNA(MLOC_58976) AAGGATCACGTATCGTGCTATCACAGTTTTGTTTGGAGAGATTGTTGTATCTTCTTATTTCTCGTCATTATGTATAACAA 2440
CircularRNA 675
7.300 7.320 7.340 7.360
Genomic(MLOC_58976) TTAGATGAGGTGCAGCGCCGTGGCAAGATCAGCCGTGTAATGACAGCAGGGACTGAAAAGAGAAGTTACTGGTAATTTTG 7360
cDNA(MLOC_58976) TTAGATGAGGTGCAGCGCCGTGGCAAGATCAGCCGTGTAATGACAGCAGGGACTGAAAAGAGAAGTTACTGGTAATTTTG 2520
CircularRNA 675
7.380
Genomic(MLOC_58976) AGCCGTCAGGATGTTGCAACTTATGTTTGG 7390
cDNA(MLOC_58976) AGCCGTCAGGATGTTGCAACTTATGTTTGG 2550
CircularRNA 675

Abscisic acid-responsive (TB2/DP1, HVA22) protein_circular RNA
(ID: Ch6:20988426-20990992)

<u>G</u>ATTTTACTTGCAATGCTTACTGTTTTCGAAAGAATTGGGGATAATTTCATATCTTGGTNNN…N
NNTGCCAATGTATAGTGAAGCAAAGCTGGCTTTTATCGTGTACTTGTGGTATCCCAAGACACAG
GTTTGGTTTCCTGCTTTTTCTGTTGCCGTAACATGTTTATGTCTTGTCGCTTTTCTGTAATAAAGG
ATTATTCCAGGGGACTTCTTATGTCTACGAATCGTTCTTCAAGCCGTATATTGCAAAACACGAAT
CTGAGATTGATCGTAATCTTCTTGAGTTGAGGACAAGGGCTAGTGATATGGCTGTTATTTACTTC
CACAAGGTTGCAGACTACAGCCAGTCAAGGTTCCATGAAATCTTGCAATATATCGCTTCTCAATC
ACAAGGATCTCGTCCTCAGGNNN…NNNCACAGCAGCAGCAGCAGCGTCCACCACCTCCACGT
ACTCGGATGGCGAACCCTGCACCACCACCTGTTCCTGCACCGACGGCACCACCTATGCCACCA
CAGCCCGCCCAAGTTCCTCCTAC**TCCACCAAGGATGCAGGTGCAAGATAAGGGACCAGTTCC**
<u>A</u>

The nucleotides of junction-region are underlined. The nucleotides of junction-region which are supported by the junction-spaning sequencing reads are shown in red. Introns are not shown if the absence is supported by sequencing reads. In the absence of supporting sequencing reads, the intronic nucletides are shown as N.

Structural relationship between the circular RNA and its parental gene

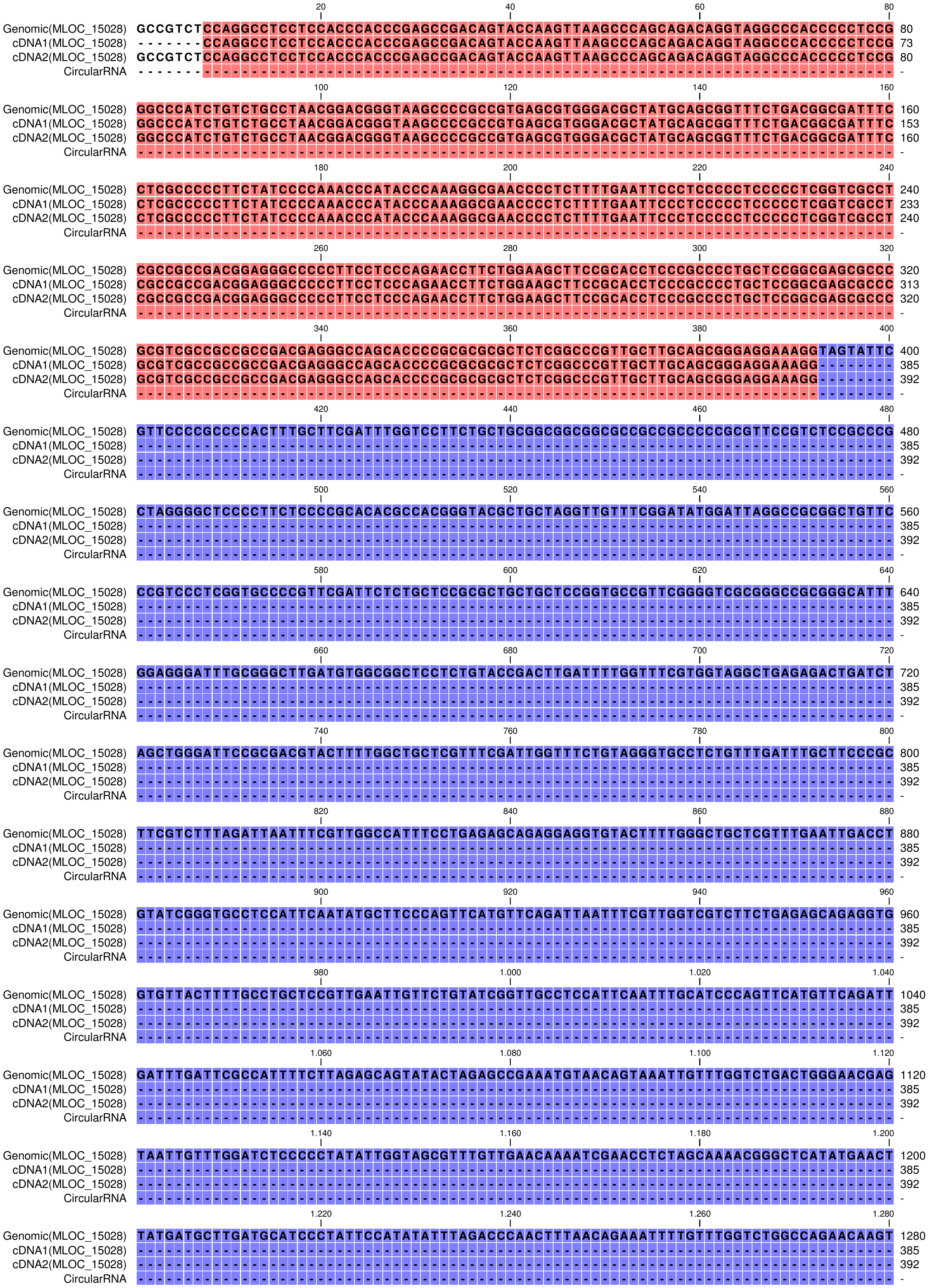

Genomic(MLOC_15028) GCCGTCTCCAGGCCTCCTCCACCCACCCGAGCCGACAGTACCAAGTTAAGCCCAGCAGACAGGTAGGCCCACCCCCTCCG 80
cDNA1(MLOC_15028) -------CCAGGCCTCCTCCACCCACCCGAGCCGACAGTACCAAGTTAAGCCCAGCAGACAGGTAGGCCCACCCCCTCCG 73
cDNA2(MLOC_15028) GCCGTCTCCAGGCCTCCTCCACCCACCCGAGCCGACAGTACCAAGTTAAGCCCAGCAGACAGGTAGGCCCACCCCCTCCG 80
CircularRNA
Genomic(MLOC_15028) GGCCCATCTGTCTGCCTAACGGACGGGTAAGCCCCGCCGTGAGCGTGGGACGCTATGCAGCGGTTTCTGACGGCGATTTC 160
cDNA1(MLOC_15028) GGCCCATCTGTCTGCCTAACGGACGGGTAAGCCCCGCCGTGAGCGTGGGACGCTATGCAGCGGTTTCTGACGGCGATTTC 153
cDNA2(MLOC_15028) GGCCCATCTGTCTGCCTAACGGACGGGTAAGCCCCGCCGTGAGCGTGGGACGCTATGCAGCGGTTTCTGACGGCGATTTC 160
Genomic(MLOC_15028) CTCGCCCCCTTCTATCCCCAAACCCATACCCAAAGGCGAACCCCTCTTTTGAATTCCCTCCCCCTCCCCCTCGGTCGCCT 240
cDNA1(MLOC_15028) CTCGCCCCCTTCTATCCCCAAACCCATACCCAAAGGCGAACCCCTCTTTTGAATTCCCTCCCCCTCCCCCTCGGTCGCCT 233
cDNA2(MLOC_15028) CTCGCCCCCTTCTATCCCCAAACCCATACCCAAAGGCGAACCCCTCTTTTGAATTCCCTCCCCCTCCCCCTCGGTCGCCT 240
Genomic(MLOC_15028) CGCCGCCGACGGAGGGCCCCCTTCCTCCCAGAACCTTCTGGAAGCTTCCGCACCTCCCGCCCCTGCTCCGGCGAGCGCCC 320
cDNA1(MLOC_15028) CGCCGCCGACGGAGGGCCCCCTTCCTCCCAGAACCTTCTGGAAGCTTCCGCACCTCCCGCCCCTGCTCCGGCGAGCGCCC 313
cDNA2(MLOC_15028) CGCCGCCGACGGAGGGCCCCCTTCCTCCCAGAACCTTCTGGAAGCTTCCGCACCTCCCGCCCCTGCTCCGGCGAGCGCCC 320
Genomic(MLOC_15028) GCGTCGCCGCCGCCGACGAGGGCCAGCACCCCGCGCGCGCTCTCGGCCCGTTGCTTGCAGCGGGAGGAAAGGTAGTATTC 400
cDNA1(MLOC_15028) GCGTCGCCGCCGCCGACGAGGGCCAGCACCCCGCGCGCGCTCTCGGCCCGTTGCTTGCAGCGGGAGGAAAGG 385
cDNA2(MLOC_15028) GCGTCGCCGCCGCCGACGAGGGCCAGCACCCCGCGCGCGCTCTCGGCCCGTTGCTTGCAGCGGGAGGAAAGG 392
Genomic(MLOC_15028) GTTCCCCGCCCCACTTTGCTTCGATTTGGTCCTTCTGCTGCGGCGGCGGCGCCGCCGCCCCCGCGTTCCGTCTCCGCCCG 480
Genomic(MLOC_15028) CTAGGGGCTCCCCTTCTCCCCGCACACGCCACGGGTACGCTGCTAGGTTGTTTCGGATATGGATTAGGCCGCGGCTGTTC 560
Genomic(MLOC_15028) CCGTCCCTCGGTGCCCCGTTCGATTCTCTGCTCCGCGCTGCTGCTCCGGTGCCGTTCGGGGTCGCGGGCCGCGGGCATTT 640
Genomic(MLOC_15028) GGAGGGATTTGCGGGCTTGATGTGGCGGCTCCTCTGTACCGACTTGATTTTGGTTTCGTGGTAGGCTGAGAGACTGATCT 720
Genomic(MLOC_15028) AGCTGGGATTCCGCGACGTACTTTTGGCTGCTCGTTTCGATTGGTTTCTGTAGGGTGCCTCTGTTTGATTTGCTTCCCGC 800
Genomic(MLOC_15028) TTCGTCTTTAGATTAATTTCGTTGGCCATTTCCTGAGAGCAGAGGAGGTGTACTTTTGGGCTGCTCGTTTGAATTGACCT 880
Genomic(MLOC_15028) GTATCGGGTGCCTCCATTCAATATGCTTCCCAGTTCATGTTCAGATTAATTTCGTTGGTCGTCTTCTGAGAGCAGAGGTG 960
Genomic(MLOC_15028) GTGTTACTTTTGCCTGCTCCGTTGAATTGTTCTGTATCGGTTGCCTCCATTCAATTTGCATCCCAGTTCATGTTCAGATT 1040
Genomic(MLOC_15028) GATTTGATTCGCCATTTTCTTAGAGCAGTATACTAGAGCCGAAATGTAACAGTAAATTGTTTGGTCTGACTGGGAACGAG 1120
Genomic(MLOC_15028) TAATTGTTTGGATCTCCCCCTATATTGGTAGCGTTTGTTGAACAAAATCGAACCTCTAGCAAAACGGGCTCATATGAACT 1200
Genomic(MLOC_15028) TATGATGCTTGATGCATCCCTATTCCATATATTTAGACCCAACTTTAACAGAAATTTTGTTTGGTCTGGCCAGAACAAGT 1280

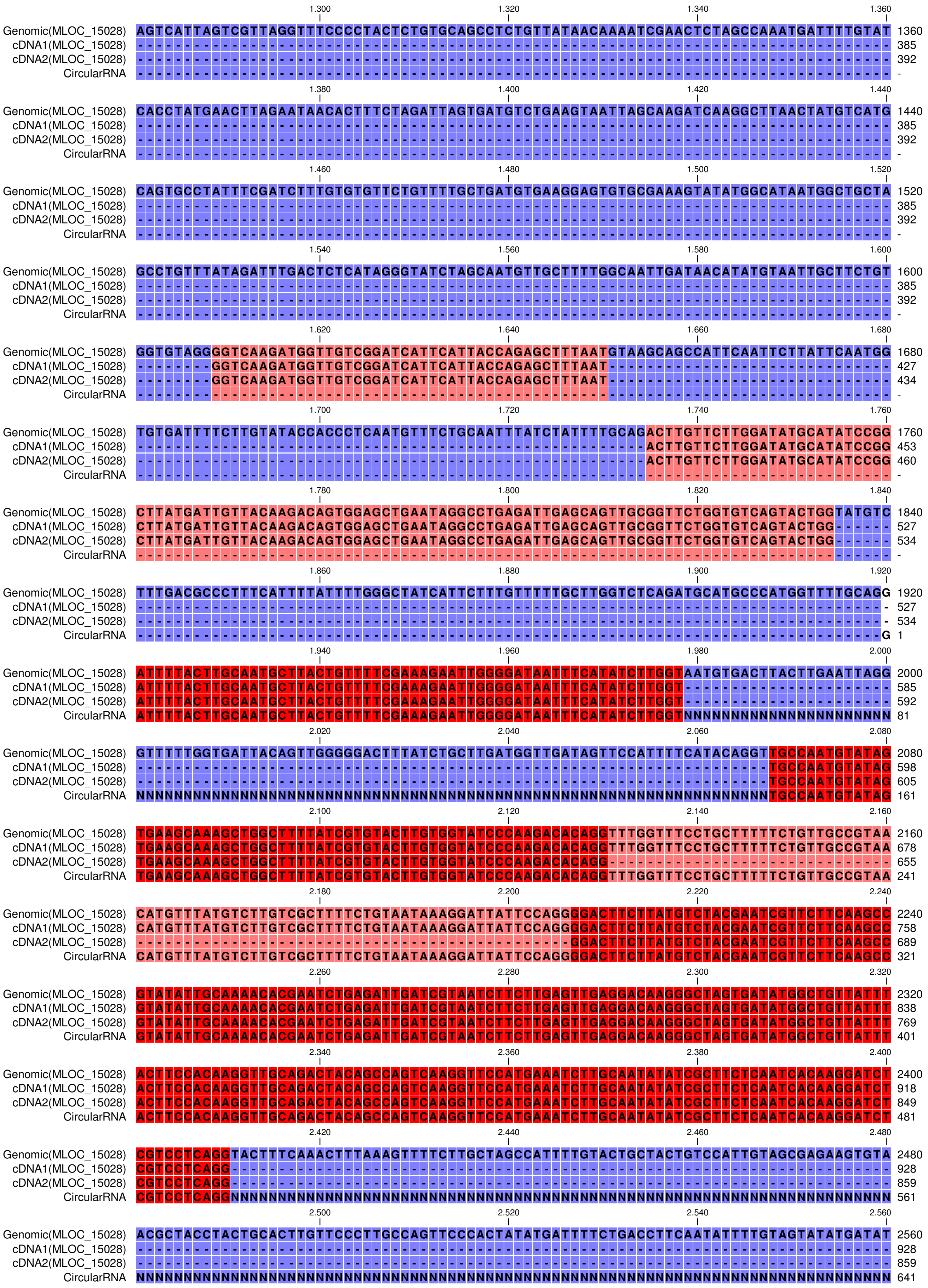
Genomic(MLOC_15028)
cDNA1(MLOC_15028)
cDNA2(MLOC_15028)
CircularRNA

```
                                                  2.580               2.600               2.620               2.640
Genomic(MLOC_15028)  AATCGGGGGGCTTCTTTCATTCTGTTAGTGCTATTTAGGTTTTCTTCTATTTGAGCCCAATGTTGTAGGCTAGATATCCT 2640
  cDNA1(MLOC_15028)  -------------------------------------------------------------------------------- 928
  cDNA2(MLOC_15028)  -------------------------------------------------------------------------------- 859
        CircularRNA  NNNNNNNNNNNNNNNNNNNNNNNNNNNNNNNNNNNNNNNNNNNNNNNNNNNNNNNNNNNNNNNNNNNNNNNNNNNNNNNN 721

                                                  2.660               2.680               2.700               2.720
Genomic(MLOC_15028)  GCCTTAACATAGAATATGATATAATCTGGGGCCTTCTTTCATAATGGTAGTGCTATCTTAGGTTTTCTTCTATTTGAGCC 2720
  cDNA1(MLOC_15028)  -------------------------------------------------------------------------------- 928
  cDNA2(MLOC_15028)  -------------------------------------------------------------------------------- 859
        CircularRNA  NNNNNNNNNNNNNNNNNNNNNNNNNNNNNNNNNNNNNNNNNNNNNNNNNNNNNNNNNNNNNNNNNNNNNNNNNNNNNNNN 801

                                                  2.740               2.760               2.780               2.800
Genomic(MLOC_15028)  TAATGTTGTAGGCTGGATATCCTGCCTTAACATAGAATTTTAAATGCTAGGACAGGAATTTCTTCACATGTTGAATTGAT 2800
  cDNA1(MLOC_15028)  -------------------------------------------------------------------------------- 928
  cDNA2(MLOC_15028)  -------------------------------------------------------------------------------- 859
        CircularRNA  NNNNNNNNNNNNNNNNNNNNNNNNNNNNNNNNNNNNNNNNNNNNNNNNNNNNNNNNNNNNNNNNNNNNNNNNNNNNNNNN 881

                                                  2.820               2.840               2.860               2.880
Genomic(MLOC_15028)  AACGATCCATGTATTACATCCTACCACCACATTTTTTCTTGTTAAAATGATCTTTAATTTACATGCCCTGATTTCTAGTG 2880
  cDNA1(MLOC_15028)  -------------------------------------------------------------------------------- 928
  cDNA2(MLOC_15028)  -------------------------------------------------------------------------------- 859
        CircularRNA  NNNNNNNNNNNNNNNNNNNNNNNNNNNNNNNNNNNNNNNNNNNNNNNNNNNNNNNNNNNNNNNNNNNNNNNNNNNNNNNN 961

                                                  2.900               2.920               2.940               2.960
Genomic(MLOC_15028)  GAAGTTAGAAAAGTATGAATAAAACCCTGTTCTGTGTTCCTTTTCACAGCACGTATTTTTACATTTCGCATCTCGTCTAC 2960
  cDNA1(MLOC_15028)  -------------------------------------------------------------------------------- 928
  cDNA2(MLOC_15028)  -------------------------------------------------------------------------------- 859
        CircularRNA  NNNNNNNNNNNNNNNNNNNNNNNNNNNNNNNNNNNNNNNNNNNNNNNNNNNNNNNNNNNNNNNNNNNNNNNNNNNNNNNN 1041

                                                  2.980               3.000               3.020               3.040
Genomic(MLOC_15028)  AAGTGGTGCTTTTATTGCTAACGGGATATTAATTATTTTTTGTAGTTAGTAGTTATAGTTGGACCCTACCACGGACCCTG 3040
  cDNA1(MLOC_15028)  -------------------------------------------------------------------------------- 928
  cDNA2(MLOC_15028)  -------------------------------------------------------------------------------- 859
        CircularRNA  NNNNNNNNNNNNNNNNNNNNNNNNNNNNNNNNNNNNNNNNNNNNNNNNNNNNNNNNNNNNNNNNNNNNNNNNNNNNNNNN 1121

                                                  3.060               3.080               3.100               3.120
Genomic(MLOC_15028)  TGCATGCGGAAGCTACGTGCACTGGGCTGCCCTTTTTTAGTAGTTGTAGTTTTTTTATTATTTTTGAGATAGCTCGCCAT 3120
  cDNA1(MLOC_15028)  -------------------------------------------------------------------------------- 928
  cDNA2(MLOC_15028)  -------------------------------------------------------------------------------- 859
        CircularRNA  NNNNNNNNNNNNNNNNNNNNNNNNNNNNNNNNNNNNNNNNNNNNNNNNNNNNNNNNNNNNNNNNNNNNNNNNNNNNNNNN 1201

                                                  3.140               3.160               3.180               3.200
Genomic(MLOC_15028)  CCATGCTGAATGCTATTTTTAATAGTTGTAGTTGGAGTTAAATAATACCGTGTTTGGGTAAAGGAAATATAATATGAAGC 3200
  cDNA1(MLOC_15028)  -------------------------------------------------------------------------------- 928
  cDNA2(MLOC_15028)  -------------------------------------------------------------------------------- 859
        CircularRNA  NNNNNNNNNNNNNNNNNNNNNNNNNNNNNNNNNNNNNNNNNNNNNNNNNNNNNNNNNNNNNNNNNNNNNNNNNNNNNNNN 1281

                                                  3.220               3.240               3.260               3.280
Genomic(MLOC_15028)  TCGGAGATCATATGCACCAAAATTTTGGCATATGCATCTACCCTTCATTTATAATAGGGGGATTATGCATCTATTTTTTC 3280
  cDNA1(MLOC_15028)  -------------------------------------------------------------------------------- 928
  cDNA2(MLOC_15028)  -------------------------------------------------------------------------------- 859
        CircularRNA  NNNNNNNNNNNNNNNNNNNNNNNNNNNNNNNNNNNNNNNNNNNNNNNNNNNNNNNNNNNNNNNNNNNNNNNNNNNNNNNN 1361

                                                  3.300               3.320               3.340               3.360
Genomic(MLOC_15028)  CTAGTTCTAAGAATTTATGTGAACGTGTGAATGATCGATACATCTGGGAAATTGTCTCTGGTCTTCTAGGAGCACTACTA 3360
  cDNA1(MLOC_15028)  -------------------------------------------------------------------------------- 928
  cDNA2(MLOC_15028)  -------------------------------------------------------------------------------- 859
        CircularRNA  NNNNNNNNNNNNNNNNNNNNNNNNNNNNNNNNNNNNNNNNNNNNNNNNNNNNNNNNNNNNNNNNNNNNNNNNNNNNNNNN 1441

                                                  3.380               3.400               3.420               3.440
Genomic(MLOC_15028)  CTAAAGATTAATGCCACACCATATTCTGAAATTGTAGAAATATCTCCACCTAGTGTTCAGTTGAATATTCTTCCTGGTTT 3440
  cDNA1(MLOC_15028)  -------------------------------------------------------------------------------- 928
  cDNA2(MLOC_15028)  -------------------------------------------------------------------------------- 859
        CircularRNA  NNNNNNNNNNNNNNNNNNNNNNNNNNNNNNNNNNNNNNNNNNNNNNNNNNNNNNNNNNNNNNNNNNNNNNNNNNNNNNNN 1521

                                                  3.460               3.480               3.500               3.520
Genomic(MLOC_15028)  GCACCCTTTGGAAGAATTTGTGTGTATGTCCTGGTTGCACCCTTTGCGTCGCAAAAGTTCTGTTATGTCATCAACTCATC 3520
  cDNA1(MLOC_15028)  -------------------------------------------------------------------------------- 928
  cDNA2(MLOC_15028)  -------------------------------------------------------------------------------- 859
        CircularRNA  NNNNNNNNNNNNNNNNNNNNNNNNNNNNNNNNNNNNNNNNNNNNNNNNNNNNNNNNNNNNNNNNNNNNNNNNNNNNNNNN 1601

                                                  3.540               3.560               3.580               3.600
Genomic(MLOC_15028)  ACCATCTAGTTGCATTATAGCACTATGCCTTTTTCTTTCCAGCAATTGTACCTCGATTTATCAGTATTCTGTAATCTCTG 3600
  cDNA1(MLOC_15028)  -------------------------------------------------------------------------------- 928
  cDNA2(MLOC_15028)  -------------------------------------------------------------------------------- 859
        CircularRNA  NNNNNNNNNNNNNNNNNNNNNNNNNNNNNNNNNNNNNNNNNNNNNNNNNNNNNNNNNNNNNNNNNNNNNNNNNNNNNNNN 1681

                                                  3.620               3.640               3.660               3.680
Genomic(MLOC_15028)  TTGGTACTTGGTAGGAAATGGCATTTGTTTCTCTGGTTTCTTTTACAGCTTAGGAGCCTGAGTTGTGGTTAATGGTGGCA 3680
  cDNA1(MLOC_15028)  -------------------------------------------------------------------------------- 928
  cDNA2(MLOC_15028)  -------------------------------------------------------------------------------- 859
        CircularRNA  NNNNNNNNNNNNNNNNNNNNNNNNNNNNNNNNNNNNNNNNNNNNNNNNNNNNNNNNNNNNNNNNNNNNNNNNNNNNNNNN 1761

                                                  3.700               3.720               3.740               3.760
Genomic(MLOC_15028)  TTTGCGTGGAGAAAGTTAGCTAGCAATGATTTTGTAGTATCATGTCATTCATTTACCATACTTCTGTACCGATTTTCTTG 3760
  cDNA1(MLOC_15028)  -------------------------------------------------------------------------------- 928
  cDNA2(MLOC_15028)  -------------------------------------------------------------------------------- 859
        CircularRNA  NNNNNNNNNNNNNNNNNNNNNNNNNNNNNNNNNNNNNNNNNNNNNNNNNNNNNNNNNNNNNNNNNNNNNNNNNNNNNNNN 1841

                                                  3.780               3.800               3.820               3.840
Genomic(MLOC_15028)  CAAGAGTTATCACAATATGATTATACATTTGGGCACACAGACAAAAGCAAACTTGTCCAGAATTACCTTGCCATTCTTAA 3840
  cDNA1(MLOC_15028)  -------------------------------------------------------------------------------- 928
  cDNA2(MLOC_15028)  -------------------------------------------------------------------------------- 859
        CircularRNA  NNNNNNNNNNNNNNNNNNNNNNNNNNNNNNNNNNNNNNNNNNNNNNNNNNNNNNNNNNNNNNNNNNNNNNNNNNNNNNNN 1921
```

```
                                  3.860               3.880               3.900               3.920
Genomic(MLOC_15028)  TGCTTTACAGATTTTTGTAGAGACAACATGGATATTTGGTCAAATAGACGACTTGCAATTTAATTTTGGTCCATGCCCCT 3920
 cDNA1(MLOC_15028)  -------------------------------------------------------------------------------- 928
 cDNA2(MLOC_15028)  -------------------------------------------------------------------------------- 859
       CircularRNA  NNNNNNNNNNNNNNNNNNNNNNNNNNNNNNNNNNNNNNNNNNNNNNNNNNNNNNNNNNNNNNNNNNNNNNNNNNNNNNNN 2001

                                  3.940               3.960               3.980               4.000
Genomic(MLOC_15028)  TGTCTTCTAGTGAAGTTTTATCTATAGTTCCTTATATAGTTTGTATCATCTTCAATGAATAACACTTCCATTCTTGTTAC 4000
 cDNA1(MLOC_15028)  -------------------------------------------------------------------------------- 928
 cDNA2(MLOC_15028)  -------------------------------------------------------------------------------- 859
       CircularRNA  NNNNNNNNNNNNNNNNNNNNNNNNNNNNNNNNNNNNNNNNNNNNNNNNNNNNNNNNNNNNNNNNNNNNNNNNNNNNNNNN 2081

                                  4.020               4.040               4.060               4.080
Genomic(MLOC_15028)  ACTGTTTGTTAGAGTTAGTGACTAGAGATCACATACAAATATATAATCTCCATATTTGAATGTGATAACCGCTGCACCAC 4080
 cDNA1(MLOC_15028)  -------------------------------------------------------------------------------- 928
 cDNA2(MLOC_15028)  -------------------------------------------------------------------------------- 859
       CircularRNA  NNNNNNNNNNNNNNNNNNNNNNNNNNNNNNNNNNNNNNNNNNNNNNNNNNNNNNNNNNNNNNNNNNNNNNNNNNNNNNNN 2161

                                  4.100               4.120               4.140               4.160
Genomic(MLOC_15028)  TCCATCATACCATTTATTGCATCATTGTATTAACAAAGGGGGAGATGTAGGCGTTCATGTACAGAAGTTGTTCTGTGTGT 4160
 cDNA1(MLOC_15028)  -------------------------------------------------------------------------------- 928
 cDNA2(MLOC_15028)  -------------------------------------------------------------------------------- 859
       CircularRNA  NNNNNNNNNNNNNNNNNNNNNNNNNNNNNNNNNNNNNNNNNNNNNNNNNNNNNNNNNNNNNNNNNNNNNNNNNNNNNNNN 2241

                                  4.180               4.200               4.220               4.240
Genomic(MLOC_15028)  ATTTGTCGAGAGTGCAGTATGGGCTTGTGTTTACCAGCTGTAGCATTTAGAGTCAGATGGTTCTTTTGAACTCATTAACA 4240
 cDNA1(MLOC_15028)  -------------------------------------------------------------------------------- 928
 cDNA2(MLOC_15028)  -------------------------------------------------------------------------------- 859
       CircularRNA  NNNNNNNNNNNNNNNNNNNNNNNNNNNNNNNNNNNNNNNNNNNNNNNNNNNNNNNNNNNNNNNNNNNNNNNNNNNNNNNN 2321

                                  4.260               4.280               4.300               4.320
Genomic(MLOC_15028)  TATCTAGTATTGTGTGCCGTGCGTATGCCAGTGTCAGATGTATATGTCTCATGTGCTGACTTTACTGTCTCTGTCTCTGT 4320
 cDNA1(MLOC_15028)  -------------------------------------------------------------------------------- 928
 cDNA2(MLOC_15028)  -------------------------------------------------------------------------------- 859
       CircularRNA  NNNNNNNNNNNNNNNNNNNNNNNNNNNNNNNNNNNNNNNNNNNNNNNNNNNNNNNNNNNNNNNNNNNNNNNNNNNNNNNN 2401

                                  4.340               4.360               4.380               4.400
Genomic(MLOC_15028)  GCAGGCACAGCAGCAGCAGCAGCGTCCACCACCTCCACGTACTCGGATGGCGAACCCTGCACCACCACCTGTTCCTGCAC 4400
 cDNA1(MLOC_15028)  -----CACAGCAGCAGCAGCAGCGTCCACCACCTCCACGTACTCGGATGGCGAACCCTGCACCACCACCTGTTCCTGCAC 1003
 cDNA2(MLOC_15028)  -----CACAGCAGCAGCAGCAGCGTCCACCACCTCCACGTACTCGGATGGCGAACCCTGCACCACCACCTGTTCCTGCAC 934
       CircularRNA  NNNNNCACAGCAGCAGCAGCAGCGTCCACCACCTCCACGTACTCGGATGGCGAACCCTGCACCACCACCTGTTCCTGCAC 2481

                                  4.420               4.440               4.460               4.480
Genomic(MLOC_15028)  CGACGGCACCACCTATGCCACCACAGCCCGCCCAAGTTCCTCCTACTCCACCAAGGATGCAGGTGCAAGATAAGGGACCA 4480
 cDNA1(MLOC_15028)  CGACGGCACCACCTATGCCACCACAGCCCGCCCAAGTTCCTCCTACTCCACCAAGGATGCAGGTGCAAGATAAGGGACCA 1083
 cDNA2(MLOC_15028)  CGACGGCACCACCTATGCCACCACAGCCCGCCCAAGTTCCTCCTACTCCACCAAGGATGCAGGTGCAAGATAAGGGACCA 1014
       CircularRNA  CGACGGCACCACCTATGCCACCACAGCCCGCCCAAGTTCCTCCTACTCCACCAAGGATGCAGGTGCAAGATAAGGGACCA 2561

                                  4.500               4.520               4.540               4.560
Genomic(MLOC_15028)  GTTCCAGTTGCCCCTCCTGGTGCAGTGCCTCCTGTGCAGCAACAAACACCTTCTGGTCCTGGCCCTGTCGCCGGCAACAG 4560
 cDNA1(MLOC_15028)  GTTCCAGTTGCCCCTCCTGGTGCAGTGCCTCCTGTGCAGCAACAAACACCTTCTGGTCCTGGCCCTGTCGCCGGCAACAG 1163
 cDNA2(MLOC_15028)  GTTCCAGTTGCCCCTCCTGGTGCAGTGCCTCCTGTGCAGCAACAAACACCTTCTGGTCCTGGCCCTGTCGCCGGCAACAG 1094
       CircularRNA  GTTCCA-------------------------------------------------------------------------- 2567

                                  4.580               4.600               4.620               4.640
Genomic(MLOC_15028)  CCCCCCAAACAGTGAAGACATGCTGGTTGATCAAGCTGGGCCATCGACAAGCAACATGCCACCACCCCGCCCAACAATGC 4640
 cDNA1(MLOC_15028)  CCCCCCAAACAGTGAAGACATGCTGGTTGATCAAGCTGGGCCATCGACAAGCAACATGCCACCACCCCGCCCAACAATGC 1243
 cDNA2(MLOC_15028)  CCCCCCAAACAGTGAAGACATGCTGGTTGATCAAGCTGGGCCATCGACAAGCAACATGCCACCACCCCGCCCAACAATGC 1174
       CircularRNA  -------------------------------------------------------------------------------- 2567

                                  4.660               4.680               4.700               4.720
Genomic(MLOC_15028)  CTGAAGACGAGGAGACGCTCATCCATGAGGCTATTCGGTTGACTCGAGGCCGGCTCAGGAGGCGCATGGGTGGATCTGGA 4720
 cDNA1(MLOC_15028)  CTGAAGACGAGGAGACGCTCATCCATGAGGCTATTCGGTTGACTCGAGGCCGGCTCAGGAGGCGCATGGGTGGATCTGGA 1323
 cDNA2(MLOC_15028)  CTGAAGACGAGGAGACGCTCATCCATGAGGCTATTCGGTTGACTCGAGGCCGGCTCAGGAGGCGCATGGGTGGATCTGGA 1254
       CircularRNA  -------------------------------------------------------------------------------- 2567

                                  4.740               4.760               4.780               4.800
Genomic(MLOC_15028)  CCTCCATCCAATTAGCTCTTCTCGCACTTATGATGATGGCAGGGGAAATGCCGGGTTGGTCTGTCAGGCATGGCTGAATT 4800
 cDNA1(MLOC_15028)  CCTCCATCCAATTAGCTCTTCTCGCACTTATGATGATGGCAGGGGAAATGCCGGGTTGGTCTGTCAGGCATGGCTGAATT 1403
 cDNA2(MLOC_15028)  CCTCCATCCAATTAGCTCTTCTCGCACTTATGATGATGGCAGGGGAAATGCCGGGTTGGTCTGTCAGGCATGGCTGAATT 1334
       CircularRNA  -------------------------------------------------------------------------------- 2567

                                  4.820               4.840               4.860               4.880
Genomic(MLOC_15028)  ACCTCGCACAGTTGCGGATGCAAGCTCACGTCCACCTTGTAAAACGATGAACCCTGGTTCCTGTTGTTTCGCAGCCCAGA 4880
 cDNA1(MLOC_15028)  ACCTCGCACAGTTGCGGATGCAAGCTCACGTCCACCTTGTAAAACGATGAACCCTGGTTCCTGTTGTTTCGCAGCCCAGA 1483
 cDNA2(MLOC_15028)  ACCTCGCACAGTTGCGGATGCAAGCTCACGTCCACCTTGTAAAACGATGAACCCTGGTTCCTGTTGTTTCGCAGCCCAGA 1414
       CircularRNA  -------------------------------------------------------------------------------- 2567

                                  4.900               4.920               4.940               4.960
Genomic(MLOC_15028)  TTGTGTATATAAACCCACCCCACACCAGGGGTATGAACGCTGGGATGTCAGTGGACCACCAGTTTAGCTAGGCGGTAGCA 4960
 cDNA1(MLOC_15028)  TTGTGTATATAAACCCACCCCACACCAGGGGTATGAACGCTGGGATGTCAGTGGACCACCAGTTTAGCTAGGCGGTAGCA 1563
 cDNA2(MLOC_15028)  TTGTGTATATAAACCCACCCCACACCAGGGGTATGAACGCTGGGATGTCAGTGGACCACCAGTTTAGCTAGGCGGTAGCA 1494
       CircularRNA  -------------------------------------------------------------------------------- 2567

                                  4.980               5.000               5.020               5.040
Genomic(MLOC_15028)  GAAGATGGTTAAGATGCTTGGCGAAATCGGTTCCAACTTTGTTCTGTTGAAATTGGTTGGGTAGTGACTTCATCTGTGCT 5040
 cDNA1(MLOC_15028)  GAAGATGGTTAAGATGCTTGGCGAAATCGGTTCCAACTTTGTTCTGTTGAAATTGGTTGGGTAGTGACTTCATCTGTGCT 1643
 cDNA2(MLOC_15028)  GAAGATGGTTAAGATGCTTGGCGAAATCGGTTCCAACTTTGTTCTGTTGAAATTGGTTGGGTAGTGACTTCATCTGTGCT 1574
       CircularRNA  -------------------------------------------------------------------------------- 2567

                                  5.060               5.080               5.100               5.120
Genomic(MLOC_15028)  GGAAAGTGGTTCAAAGGTGAAGTTGAACAATCTCTGGCTGTACTGGAAAGATAGAACCTTGGCCTGTCTGGCATTAGCAC 5120
 cDNA1(MLOC_15028)  GGAAAGTGGTTCAAAGGTGAAGTTGAACAATCTCTGGCTGTACTGGAAAGATAGA------------------------- 1698
 cDNA2(MLOC_15028)  GGAAAGTGGTTCAAAGGTGAAGTTGAACAATCTCTGGCTGTACTGGAAAGATAGAACCTTGGCCTGTCTGGCATTAGCAC 1654
       CircularRNA  -------------------------------------------------------------------------------- 2567
```

```
                                                        5.140                                 5.160
                                                          |                                     |
Genomic(MLOC_15028) GTTCTGTTGCTGCTACATTTATTTAGCTTCTTCTCGATTTCGC 5163
  cDNA1(MLOC_15028) ------------------------------------------- 1698
  cDNA2(MLOC_15028) GTTCTGTTGCTGCTACATTTATTTAGCTTCTTCTCGATTTCGC 1697
        CircularRNA ------------------------------------------- 2567
```

Far upstream element-binding protein 2 _circular RNA
(ID: Ch6:245908278-245908734)

CAAATCCTTCTGCAGGTCATTCCTTTACATTTGCCTCCTGGTGATACTTCAACTGAAAGAACACT
GTATATTGATGGTACTGCAGAGCAAATTGAAATAGCAAAGCAGCTTGTGAGTGAGGTTACCAGT
GAGGTCAGCTTTCTTTCTCTTTTCATGCCACTTCCTAATTGCTGCCACTGTTTGTTATTTATCTGT
GTTGTTAAAATCATGTGGGTTTGATTACAGCTTTTTGTATGTTACTGTGTTTTTGTCTCGATTTATA
TTTCTCTATTTGACCTCTGTTTTCCATTGTTTGCTTTTGACAATCTTCTGTGTATTTAGTTATGATA
TACCCTATATTCTTGTCAAATATACATGTCCAATTTTTTTATCTCAATGGGTTGTCTTGTGAAACAA
GTTGGTCATGGAGCA**CCAGCAGTTTGGCTTTGACATGATTGTATACTTTATACAATTCTGT**

The nucleotides of junction-region are underlined. The nucleotides of junction-region which are supported by the junction-spaning sequencing reads are shown in red. Introns are not shown if the absence is supported by sequencing reads. In the absence of supporting sequencing reads, the intronic nucletides are shown as N.

Structural relationship between the circular RNA and its parental gene

```
                                        20                  40                  60                  80
Genomic(MLOC_60294) AATGTAGCATATAAATACGCATCCCTGCAGCACTCTAGGGTTCTCCGTTTTGCGCCGTCTTATCCCTCTGCTCCCCCACA 80
   cDNA(MLOC_60294) ----------------------------------------------GTTTTGCGCCGTCTTATCCCTCTGCTCCCCCACA 34
        CircularRNA -------------------------------------------------------------------------------- -

                                       100                 120                 140                 160
Genomic(MLOC_60294) ACAGCGCGCTTCTCGCCACCACCACCCTAACCCTAGTTCCCCCGCCGCCATGGCCGACGACCACTACTCCTCCAAGCGCA 160
   cDNA(MLOC_60294) ACAGCGCGCTTCTCGCCACCACCACCCTAACCCTAGTTCCCCCGCCGCCATGGCCGACGACCACTACTCCTCCAAGCGCA 114
        CircularRNA -------------------------------------------------------------------------------- -

                                       180                 200                 220                 240
Genomic(MLOC_60294) AGTACGACGACTCCCCGCCGCCGCGCCGCACGGGATTCTCCTCCGGCCCACCGCCTGCCTCGCCGCCGGTTGCCGGTGGC 240
   cDNA(MLOC_60294) AGTACGACGACTCCCCGCCGCCGCGCCGCACGGGATTCTCCTCCGGCCCACCGCCTGCCTCGCCGCCGGTTGCCGGTGGC 194
        CircularRNA -------------------------------------------------------------------------------- -

                                       260                 280                 300                 320
Genomic(MLOC_60294) CCGGTGCCATCTTCGTACAATACTGTGCCGCCGCCTCCCGACGAGATCCAGCTCGCGAAGCAACGCGCGCAGGAGATCGC 320
   cDNA(MLOC_60294) CCGGTGCCATCTTCGTACAATACTGTGCCGCCGCCTCCCGACGAGATCCAGCTCGCGAAGCAACGCGCGCAGGAGATCGC 274
        CircularRNA -------------------------------------------------------------------------------- -

                                       340                 360                 380                 400
Genomic(MLOC_60294) AGCTAGGCTCTTCAGCGCCGCTGAGGCGAAGCGTCCCCGTATAGACAACGGCGACGACGACGTTGGCACCGGTGGCGGAG 400
   cDNA(MLOC_60294) AGCTAGGCTCTTCAGCGCCGCTGAGGCGAAGCGTCCCCGTATAGACAACGGCGACGACGACGTTGGCACCGGTGGCGGAG 354
        CircularRNA -------------------------------------------------------------------------------- -

                                       420                 440                 460                 480
Genomic(MLOC_60294) GAGGTTCCCTGGGGGGCGGTGGCCGTATCGGTGGCGGCGGCCTCGGATTCTCGTCCTCAGCCGGTGGTGGTGAGTATCTT 480
   cDNA(MLOC_60294) GAGGTTCCCTGGGGGGCGGTGGCCGTATCGGTGGCGGCGGCCTCGGATTCTCGTCCTCAGCCGGTGGTGG---------- 424
        CircularRNA -------------------------------------------------------------------------------- -

                                       500                 520                 540                 560
Genomic(MLOC_60294) GGCTATATCTCATGAAACACGTTTTGGATCGGGAGATCTGTTTTCTGTGTGCTTGTGGTCGCGGCTGCTGTTATGCTGTT 560
   cDNA(MLOC_60294) -------------------------------------------------------------------------------- 424
        CircularRNA -------------------------------------------------------------------------------- -

                                       580                 600                 620                 640
Genomic(MLOC_60294) TATTTAGTTTGTTCCTTAGCTGCTAAGTACTGCTATGCGATCCATGCGATTTAGCGATACGTTTAGGAAGCAGTGTAAGA 640
   cDNA(MLOC_60294) -------------------------------------------------------------------------------- 424
        CircularRNA -------------------------------------------------------------------------------- -

                                       660                 680                 700                 720
Genomic(MLOC_60294) AAATTGCCTCCCGAGGCGATCTTAACCGTGCTACAATCTAAGCAACAACAGAACGCCTAGCGATTTCGTTATCCTTGCTA 720
   cDNA(MLOC_60294) -------------------------------------------------------------------------------- 424
        CircularRNA -------------------------------------------------------------------------------- -

                                       740                 760                 780                 800
Genomic(MLOC_60294) GGAAGTTTAGATCTTCGTAGGTTAAAATATTATGACGGGTTTATCAGGATGACTAAAAGTTTGTTAATAATTCTGGCAAA 800
   cDNA(MLOC_60294) -------------------------------------------------------------------------------- 424
        CircularRNA -------------------------------------------------------------------------------- -

                                       820                 840                 860                 880
Genomic(MLOC_60294) TATATCTTGTGTAGATCAGTAGTATCCATAGTTTGATTCTGCTATATCATGTAATATTTTAATCAGTGTCTCGAGGAACT 880
   cDNA(MLOC_60294) -------------------------------------------------------------------------------- 424
        CircularRNA -------------------------------------------------------------------------------- -

                                       900                 920                 940                 960
Genomic(MLOC_60294) AGCACCTCTCTGCTAGGTGCAGCAATGATATGCGTTGAAACTTCTGTAGTAGCCATTGCCACTAAATGACAATAGTTTAT 960
   cDNA(MLOC_60294) -------------------------------------------------------------------------------- 424
        CircularRNA -------------------------------------------------------------------------------- -

                                       980               1.000               1.020               1.040
Genomic(MLOC_60294) GTGGTTTCCTTTTTCTCATTTTTTTCATGTGAATGCAAAAACGGCAATATACTGCTTTCATAATTCTTTATAGTCACAAA 1040
   cDNA(MLOC_60294) -------------------------------------------------------------------------------- 424
        CircularRNA -------------------------------------------------------------------------------- -

                                     1.060               1.080               1.100               1.120
Genomic(MLOC_60294) GCCAAATATTATCTCATGAAGTACATAAATGATGTGATTTCCTTTAATATTTCCAGGCAGCTCTGAACGTTATTAGTTTG 1120
   cDNA(MLOC_60294) -------------------------------------------------------------------------------- 424
        CircularRNA -------------------------------------------------------------------------------- -

                                     1.140               1.160               1.180               1.200
Genomic(MLOC_60294) TTACTTATGTACTGACATCATTTTATTGTACTGTTTTATGTTTTTCACGTGGATGTTATTCTTCTCAGTTAGCAAATACG 1200
   cDNA(MLOC_60294) -------------------------------------------------------------------------------- 424
        CircularRNA -------------------------------------------------------------------------------- -

                                     1.220               1.240               1.260               1.280
Genomic(MLOC_60294) CAGCTATCAGGAATATAATGAGTCGTTGTTTTGTTTCCAGGCCATGGTTCTTCCCTCCCTCCCTTATCTTCTCAAGGAAA 1280
   cDNA(MLOC_60294) -----------------------------------------CCATGGTTCTTCCCTCCCTCCCTTATCTTCTCAAGGAAA 463
        CircularRNA -------------------------------------------------------------------------------- -

                                     1.300               1.320               1.340               1.360
Genomic(MLOC_60294) CTCGCATCAGTATTCTTCATATGGTGGTGGATACCAGAGTGGTAGTACAACAAAAAAGATTGATATCCCAAATGGAAGGG 1360
   cDNA(MLOC_60294) CTCGCATCAGTATTCTTCATATGGTGGTGGATACCAGAGTGGTAGTACAACAAAAAAGATTGATATCCCAAATGGAAGGG 543
        CircularRNA -------------------------------------------------------------------------------- -

                                     1.380               1.400               1.420               1.440
Genomic(MLOC_60294) TATGCTACTTGGCAGTTTACTCTTCTGGTCTTTGGATTCATGAAGTTGATGGAAGGTTGCATGAAGTGCAGTTTTTTTGT 1440
   cDNA(MLOC_60294) T------------------------------------------------------------------------------- 544
        CircularRNA -------------------------------------------------------------------------------- -

                                     1.460               1.480               1.500               1.520
Genomic(MLOC_60294) CTGCTAACTGATAACGTTCCTTGATTTTTAGGTTGGTGTTATCATTGGCAAAGCTGGAGAAACTATAAAGCATCTCCAAG 1520
   cDNA(MLOC_60294) ---------------------------------TGGTGTTATCATTGGCAAAGCTGGAGAAACTATAAAGCATCTCCAAG 591
        CircularRNA -------------------------------------------------------------------------------- -

                                     1.540               1.560               1.580               1.600
Genomic(MLOC_60294) CTCAGTCAGGGGCAAAGATCCAAGTAACAAGGGACATGGATGTTCAACCTGGCTCACAGACAAGATCGGTTGATATTTCG 1600
   cDNA(MLOC_60294) CTCAGTCAGGGGCAAAGATCCAAGTAACAAGGGACATGGATGTTCAACCTGGCTCACAGACAAGATCGGTTGATATTTCG 671
        CircularRNA -------------------------------------------------------------------------------- -
```

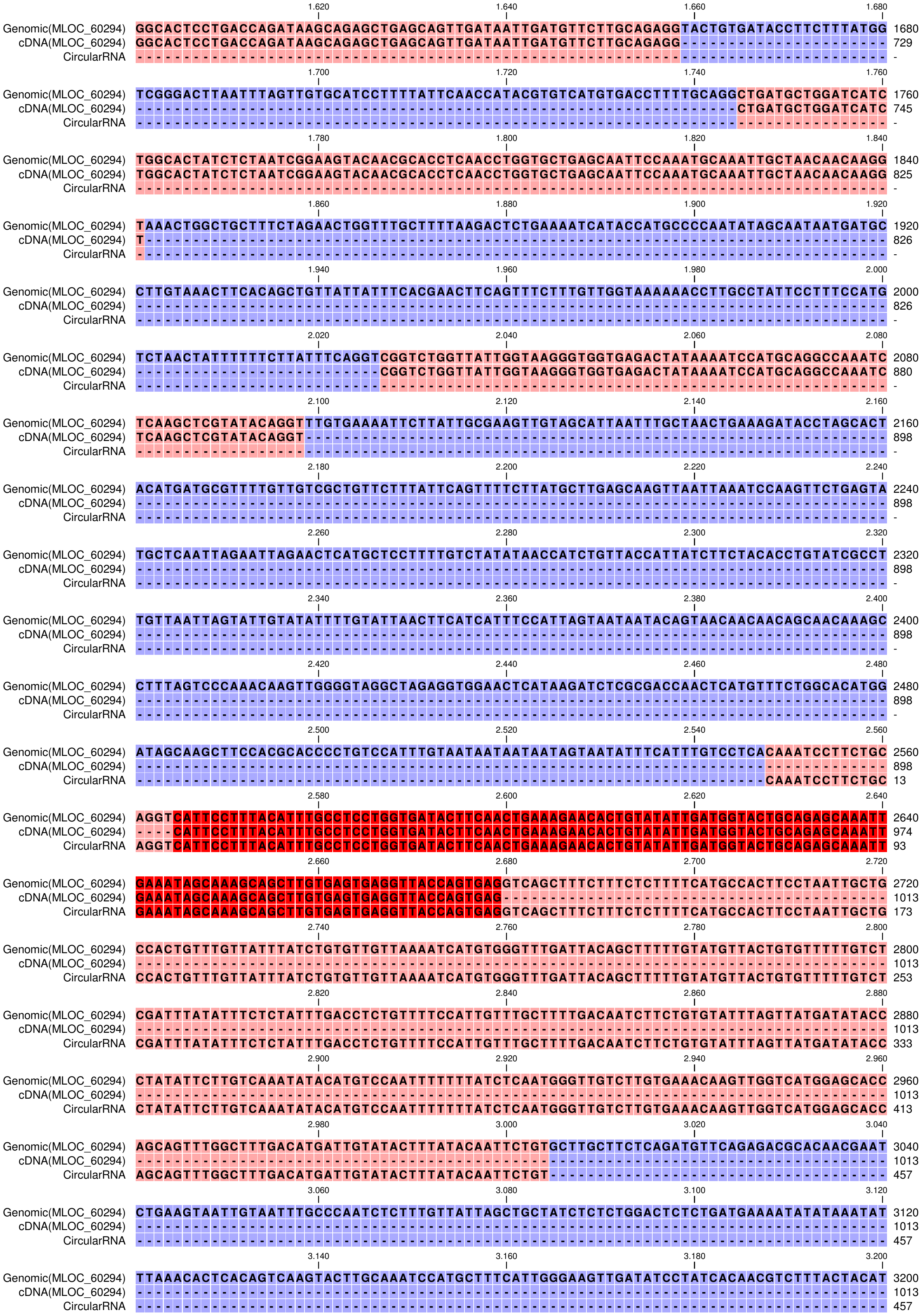

1.620 1.640 1.660 1.680
Genomic(MLOC_60294) GGCACTCCTGACCAGATAAGCAGAGCTGAGCAGTTGATAATTGATGTTCTTGCAGAGGTACTGTGATACCTTCTTTATGG 1680
cDNA(MLOC_60294) GGCACTCCTGACCAGATAAGCAGAGCTGAGCAGTTGATAATTGATGTTCTTGCAGAGG---------------------- 729
CircularRNA -- -
1.700 1.720 1.740 1.760
Genomic(MLOC_60294) TCGGGACTTAATTTAGTTGTGCATCCTTTTATTCAACCATACGTGTCATGTGACCTTTTGCAGGCTGATGCTGGATCATC 1760
cDNA(MLOC_60294) --CTGATGCTGGATCATC 745
CircularRNA -- -
1.780 1.800 1.820 1.840
Genomic(MLOC_60294) TGGCACTATCTCTAATCGGAAGTACAACGCACCTCAACCTGGTGCTGAGCAATTCCAAATGCAAATTGCTAACAACAAGG 1840
cDNA(MLOC_60294) TGGCACTATCTCTAATCGGAAGTACAACGCACCTCAACCTGGTGCTGAGCAATTCCAAATGCAAATTGCTAACAACAAGG 825
CircularRNA -- -
1.860 1.880 1.900 1.920
Genomic(MLOC_60294) TAAACTGGCTGCTTTCTAGAACTGGTTTGCTTTTAAGACTCTGAAAATCATACCATGCCCCAATATAGCAATAATGATGC 1920
cDNA(MLOC_60294) T--- 826
CircularRNA -- -
1.940 1.960 1.980 2.000
Genomic(MLOC_60294) CTTGTAAACTTCACAGCTGTTATTATTTCACGAACTTCAGTTTCTTTGTTGGTAAAAAACCTTGCCTATTCCTTTCCATG 2000
cDNA(MLOC_60294) -- 826
CircularRNA -- -
2.020 2.040 2.060 2.080
Genomic(MLOC_60294) TCTAACTATTTTTTCTTATTTCAGGTCGGTCTGGTTATTGGTAAGGGTGGTGAGACTATAAAATCCATGCAGGCCAAATC 2080
cDNA(MLOC_60294) --------------------------CGGTCTGGTTATTGGTAAGGGTGGTGAGACTATAAAATCCATGCAGGCCAAATC 880
CircularRNA -- -
2.100 2.120 2.140 2.160
Genomic(MLOC_60294) TCAAGCTCGTATACAGGTTTGTGAAAATTCTTATTGCGAAGTTGTAGCATTAATTTGCTAACTGAAAGATACCTAGCACT 2160
cDNA(MLOC_60294) TCAAGCTCGTATACAGGT-- 898
CircularRNA -- -
2.180 2.200 2.220 2.240
Genomic(MLOC_60294) ACATGATGCGTTTTGTTGTCGCTGTTCTTTATTCAGTTTTCTTATGCTTGAGCAAGTTAATTAAATCCAAGTTCTGAGTA 2240
cDNA(MLOC_60294) -- 898
CircularRNA -- -
2.260 2.280 2.300 2.320
Genomic(MLOC_60294) TGCTCAATTAGAATTAGAACTCATGCTCCTTTTGTCTATATAACCATCTGTTACCATTATCTTCTACACCTGTATCGCCT 2320
cDNA(MLOC_60294) -- 898
CircularRNA -- -
2.340 2.360 2.380 2.400
Genomic(MLOC_60294) TGTTAATTAGTATTGTATATTTTGTATTAACTTCATCATTTCCATTAGTAATAATACAGTAACAACAACAGCAACAAAGC 2400
cDNA(MLOC_60294) -- 898
CircularRNA -- -
2.420 2.440 2.460 2.480
Genomic(MLOC_60294) CTTTAGTCCCAAACAAGTTGGGGTAGGCTAGAGGTGGAACTCATAAGATCTCGCGACCAACTCATGTTTCTGGCACATGG 2480
cDNA(MLOC_60294) -- 898
CircularRNA -- -
2.500 2.520 2.540 2.560
Genomic(MLOC_60294) ATAGCAAGCTTCCACGCACCCCTGTCCATTTGTAATAATAATAATAGTAATATTTCATTTGTCCTCACAAATCCTTCTGC 2560
cDNA(MLOC_60294) -- 898
CircularRNA ---CAAATCCTTCTGC 13
2.580 2.600 2.620 2.640
Genomic(MLOC_60294) AGGTCATTCCTTTACATTTGCCTCCTGGTGATACTTCAACTGAAAGAACACTGTATATTGATGGTACTGCAGAGCAAATT 2640
cDNA(MLOC_60294) ----CATTCCTTTACATTTGCCTCCTGGTGATACTTCAACTGAAAGAACACTGTATATTGATGGTACTGCAGAGCAAATT 974
CircularRNA AGGTCATTCCTTTACATTTGCCTCCTGGTGATACTTCAACTGAAAGAACACTGTATATTGATGGTACTGCAGAGCAAATT 93
2.660 2.680 2.700 2.720
Genomic(MLOC_60294) GAAATAGCAAAGCAGCTTGTGAGTGAGGTTACCAGTGAGGTCAGCTTTCTTTCTCTTTTCATGCCACTTCCTAATTGCTG 2720
cDNA(MLOC_60294) GAAATAGCAAAGCAGCTTGTGAGTGAGGTTACCAGTGAG--- 1013
CircularRNA GAAATAGCAAAGCAGCTTGTGAGTGAGGTTACCAGTGAGGTCAGCTTTCTTTCTCTTTTCATGCCACTTCCTAATTGCTG 173
2.740 2.760 2.780 2.800
Genomic(MLOC_60294) CCACTGTTTGTTATTTATCTGTGTTGTTAAAATCATGTGGGTTTGATTACAGCTTTTTGTATGTTACTGTGTTTTTGTCT 2800
cDNA(MLOC_60294) -- 1013
CircularRNA CCACTGTTTGTTATTTATCTGTGTTGTTAAAATCATGTGGGTTTGATTACAGCTTTTTGTATGTTACTGTGTTTTTGTCT 253
2.820 2.840 2.860 2.880
Genomic(MLOC_60294) CGATTTATATTTCTCTATTTGACCTCTGTTTTCCATTGTTTGCTTTTGACAATCTTCTGTGTATTTAGTTATGATATACC 2880
cDNA(MLOC_60294) -- 1013
CircularRNA CGATTTATATTTCTCTATTTGACCTCTGTTTTCCATTGTTTGCTTTTGACAATCTTCTGTGTATTTAGTTATGATATACC 333
2.900 2.920 2.940 2.960
Genomic(MLOC_60294) CTATATTCTTGTCAAATATACATGTCCAATTTTTTTATCTCAATGGGTTGTCTTGTGAAACAAGTTGGTCATGGAGCACC 2960
cDNA(MLOC_60294) -- 1013
CircularRNA CTATATTCTTGTCAAATATACATGTCCAATTTTTTTATCTCAATGGGTTGTCTTGTGAAACAAGTTGGTCATGGAGCACC 413
2.980 3.000 3.020 3.040
Genomic(MLOC_60294) AGCAGTTTGGCTTTGACATGATTGTATACTTTATACAATTCTGTGCTTGCTTCTCAGATGTTCAGAGACGCACAACGAAT 3040
cDNA(MLOC_60294) -- 1013
CircularRNA AGCAGTTTGGCTTTGACATGATTGTATACTTTATACAATTCTGT------------------------------------ 457
3.060 3.080 3.100 3.120
Genomic(MLOC_60294) CTGAAGTAATTGTAATTTGCCCAATCTCTTTGTTATTAGCTGCTATCTCTCTGGACTCTCTGATGAAAATATATAAATAT 3120
cDNA(MLOC_60294) -- 1013
CircularRNA -- 457
3.140 3.160 3.180 3.200
Genomic(MLOC_60294) TTAAACACTCACAGTCAAGTACTTGCAAATCCATGCTTTCATTGGGAAGTTGATATCCTATCACAACGTCTTTACTACAT 3200
cDNA(MLOC_60294) -- 1013
CircularRNA -- 457

```
                    3.220               3.240               3.260               3.280
Genomic(MLOC_60294) ATCATACTGGTGCTTGTGGTGCTATCATATGTCCCTGGTTACACTGTGTTTATTATCTTGACATAAGGCCAAATTAGTGT 3280
cDNA(MLOC_60294)    -------------------------------------------------------------------------------- 1013
CircularRNA         -------------------------------------------------------------------------------- 457

                    3.300               3.320               3.340               3.360
Genomic(MLOC_60294) CTGTAAATAGTTCTGAGGTCTGCACACATCAATCCTGGTGGCCAGGTTTGTAACTACCTGATGTTAATATACTTGATGCT 3360
cDNA(MLOC_60294)    -------------------------------------------------------------------------------- 1013
CircularRNA         -------------------------------------------------------------------------------- 457

                    3.380               3.400               3.420               3.440
Genomic(MLOC_60294) CATGAATGAAAGATAAACTCTGTTTCGGTATAGAAGGTCCATGTGTAGCATTGGAGCACCATTAGAATCCAAATGCAAAT 3440
cDNA(MLOC_60294)    -------------------------------------------------------------------------------- 1013
CircularRNA         -------------------------------------------------------------------------------- 457

                    3.460               3.480               3.500               3.520
Genomic(MLOC_60294) TCAATGGTATATACTTTCTGAACAATATAATACATACATCCCGGAACACGCCTGGTCCTTCTATATTGAAACGGAAATAA 3520
cDNA(MLOC_60294)    -------------------------------------------------------------------------------- 1013
CircularRNA         -------------------------------------------------------------------------------- 457

                    3.540               3.560               3.580               3.600
Genomic(MLOC_60294) CTGCTTCGTATTTGGATTATATAATCGGATTTCATGTATCTGGTCTTGTGATCTTGTCTGTTTAAATTTGCATTTGTTAT 3600
cDNA(MLOC_60294)    -------------------------------------------------------------------------------- 1013
CircularRNA         -------------------------------------------------------------------------------- 457

                    3.620               3.640               3.660               3.680
Genomic(MLOC_60294) TTCTGTACATCTTTGGGATAGTTGGTGCAATCCTCTATACAGGTGACGAGTTTGCATTGATATTTTTGCTCGGTCATGGG 3680
cDNA(MLOC_60294)    -------------------------------------------------------------------------------- 1013
CircularRNA         -------------------------------------------------------------------------------- 457

                    3.700               3.720               3.740               3.760
Genomic(MLOC_60294) GTAGATATCATTCTTACATTCTTCCTTCAATTCTTGATATCCCATTTTATCTGTTGCTAGCACCACCAACTGGGAGCAAC 3760
cDNA(MLOC_60294)    -------------------------------------------------------------------------------- 1013
CircularRNA         -------------------------------------------------------------------------------- 457

                    3.780               3.800               3.820               3.840
Genomic(MLOC_60294) ATGTAACAGATAACAATGTTATAATTTATTGACTAGGCAAGAAAGTTTGTATATATTATGTGTTGTTTTGAAAGATTATG 3840
cDNA(MLOC_60294)    -------------------------------------------------------------------------------- 1013
CircularRNA         -------------------------------------------------------------------------------- 457

                    3.860               3.880               3.900               3.920
Genomic(MLOC_60294) CAGGATACCTTGCATTAATCTATTGTCTTTGGTTTGCCCGTATATATTTTCTCTCTGTTGATTTTTCAGGTTACCATGAC 3920
cDNA(MLOC_60294)    -------------------------------------------------------------------------------- 1013
CircularRNA         -------------------------------------------------------------------------------- 457

                    3.940               3.960               3.980               4.000
Genomic(MLOC_60294) CAAGCTTCCTTAAAACTTTGCTTAAATTCTGTTTGACATTCTTTTGTTCACTTGTACTCCCTCCGTCTCAAAATAAGTTT 4000
cDNA(MLOC_60294)    -------------------------------------------------------------------------------- 1013
CircularRNA         -------------------------------------------------------------------------------- 457

                    4.020               4.040               4.060               4.080
Genomic(MLOC_60294) TTTAAGCTTAGTACAACTTCGTACAACAGCTAGTACAAAGTTGAGGCAGTTATTTTGGGACAGCGGAAGTAATATATTCT 4080
cDNA(MLOC_60294)    -------------------------------------------------------------------------------- 1013
CircularRNA         -------------------------------------------------------------------------------- 457

                    4.100               4.120               4.140               4.160
Genomic(MLOC_60294) TTTGTTCACTTGTACTCCCTCCGTCTCAAAATAAGTTTTTTAAGCTTAGTACAACTTCGTACAACGGCTAGTACAAAGTT 4160
cDNA(MLOC_60294)    -------------------------------------------------------------------------------- 1013
CircularRNA         -------------------------------------------------------------------------------- 457

                    4.180               4.200               4.220               4.240
Genomic(MLOC_60294) GAGGCAGTTATTTTGGGACAGCGGAAGTAATATATTCTTTGTTGTAGGAGACATGTCTGAAAGAACTGCTGTTGTGTATG 4240
cDNA(MLOC_60294)    -------------------------------------------------------------------------------- 1013
CircularRNA         -------------------------------------------------------------------------------- 457

                    4.260               4.280               4.300               4.320
Genomic(MLOC_60294) TTCTGTCATTTAGCAATATGCGTATATGTTCCACATAATAAGGATCACTGTCACCATCGAGTATTACTGATTTATCAACT 4320
cDNA(MLOC_60294)    -------------------------------------------------------------------------------- 1013
CircularRNA         -------------------------------------------------------------------------------- 457

                    4.340               4.360               4.380               4.400
Genomic(MLOC_60294) TCTATAAAGTGCAAAACATTTATAAACTGCAAGGCAATCCTTTATTGCATGAATATTCAATGTATGTATGTGTTTGTCTA 4400
cDNA(MLOC_60294)    -------------------------------------------------------------------------------- 1013
CircularRNA         -------------------------------------------------------------------------------- 457

                    4.420               4.440               4.460               4.480
Genomic(MLOC_60294) GCTGCCCAATCTTCATTAGCTCCCATAGTTTTGCAAATGGGAGACGCTAGATGGGCATCAGCAATGTTGCTTGAGAATCT 4480
cDNA(MLOC_60294)    -------------------------------------------------------------------------------- 1013
CircularRNA         -------------------------------------------------------------------------------- 457

                    4.500               4.520               4.540               4.560
Genomic(MLOC_60294) TGCTCGGTGAGTTGACCCCTCCATTTTTTCTTGGTGGTTCTGGAATTTCTGTATATTACGTTGCTTTGTTTTTGGATATG 4560
cDNA(MLOC_60294)    -------------------------------------------------------------------------------- 1013
CircularRNA         -------------------------------------------------------------------------------- 457

                    4.580               4.600               4.620               4.640
Genomic(MLOC_60294) TTTTGTCTGGTCTGCACTTGTTCTGCATGGTCCATTGCATGTTTTATCCTATACATATACAGATGTGCAAACTATTTTTG 4640
cDNA(MLOC_60294)    -------------------------------------------------------------------------------- 1013
CircularRNA         -------------------------------------------------------------------------------- 457

                    4.660               4.680               4.700               4.720
Genomic(MLOC_60294) CAGAATCGTGCCAGAAATCAAATGTCAGGTGGCTATTCTCAGCAGGGCTACCGCCCTCCTCGTCCTCAGGCCAACTGGGG 4720
cDNA(MLOC_60294)    ---AATCGTGCCAGAAATCAAATGTCAGGTGGCTATTCTCAGCAGGGCTACCGCCCTCCTCGTCCTCAGGCCAACTGGGG 1090
CircularRNA         -------------------------------------------------------------------------------- 457

                    4.740               4.760               4.780               4.800
Genomic(MLOC_60294) CGCGCCTGGTGCACCAACAACACAACAGCCTGGTTACGGTTACATGCAGCCTGGAGCTTATCCTGGTGCACCACCACAGT 4800
cDNA(MLOC_60294)    CGCGCCTGGTGCACCAACAACACAACAGCCTGGTTACGGTTACATGCAGCCTGGAGCTTATCCTGGTGCACCACCACAGT 1170
CircularRNA         -------------------------------------------------------------------------------- 457
```

```
                                    4.820                 4.840                 4.860                 4.880
Genomic(MLOC_60294) ATGGCCAGCAACCTTATGGCAGCTACCCTCCAGCATCTGGAGGGTATCAGACAGGGTGGGATCAGTCTTCAAACCAGCAA 4880
   cDNA(MLOC_60294) ATGGCCAGCAACCTTATGGCAGCTACCCTCCAGCATCTGGAGGGTATCAGACAGGGTGGGATCAGTCTTCAAACCAGCAA 1250
        CircularRNA -------------------------------------------------------------------------------- 457

                                    4.900                 4.920                 4.940                 4.960
Genomic(MLOC_60294) TCACAGCAGCCCCCCCCTGGCACTGGCTATGACTACTATAACCAACAACAGCAACCACAACAGCAACAATCTGCCACTGG 4960
   cDNA(MLOC_60294) TCACAGCAGCCCCCCCCTGGCACTGGCTATGACTACTATAACCAACAACAGCAACCACAACAGCAACAATCTGCCACTGG 1330
        CircularRNA -------------------------------------------------------------------------------- 457

                                    4.980                 5.000                 5.020                 5.040
Genomic(MLOC_60294) AACTGCTGCACCTGCTGATGCTAACAACTACAATTACAGCCATCCTCCTGCTAGTTATGCTTCACAAGGGTATGGTGATT 5040
   cDNA(MLOC_60294) AACTGCTGCACCTGCTGATGCTAACAACTACAATTACAGCCATCCTCCTGCTAGTTATGCTTCACAAGGGTATGGTGATT 1410
        CircularRNA -------------------------------------------------------------------------------- 457

                                    5.060                 5.080                 5.100                 5.120
Genomic(MLOC_60294) CTACCTACTCCCAGCAGAGTGGCGGGCAGCAAGCTTATGACTACTCTGGTTACCAGACCCAAGGGCAGCAGCAGTCTTAC 5120
   cDNA(MLOC_60294) CTACCTACTCCCAGCAGAGTGGCGGGCAGCAAGCTTATGACTACTCTGGTTACCAGACCCAAGGGCAGCAGCAGTCTTAC 1490
        CircularRNA -------------------------------------------------------------------------------- 457

                                    5.140                 5.160                 5.180                 5.200
Genomic(MLOC_60294) TCACAGCAGCCTGGATATGATCAGCAGAGCTATGGGGCATCTGGCTATGGATCAGCTGCTAACTCAACTCAGGATGGCAC 5200
   cDNA(MLOC_60294) TCACAGCAGCCTGGATATGATCAGCAGAGCTATGGGGCATCTGGCTATGGATCAGCTGCTAACTCAACTCAGGATGGCAC 1570
        CircularRNA -------------------------------------------------------------------------------- 457

                                    5.220                 5.240                 5.260                 5.280
Genomic(MLOC_60294) TGCACCGAGCTATGGTGGTCCAGGTGGCGCTGGTCAAGCATCTCCAGGGCAGCAAGCTTCAACTCCTTCCACCGGAGGCC 5280
   cDNA(MLOC_60294) TGCACCGAGCTATGGTGGTCCAGGTGGCGCTGGTCAAGCATCTCCAGGGCAGCAAGCTTCAACTCCTTCCACCGGAGGCC 1650
        CircularRNA -------------------------------------------------------------------------------- 457

                                    5.300                 5.320                 5.340                 5.360
Genomic(MLOC_60294) AACCGGGTTATCCTAGCCAACCACCTGCTAGTGCTGCTGCATCAAGCTACCCGGCGCAAGGTTCTGCCCCTCCATCTGGA 5360
   cDNA(MLOC_60294) AACCGGGTTATCCTAGCCAACCACCTGCTAGTGCTGCTGCATCAAGCTACCCGGCGCAAGGTTCTGCCCCTCCATCTGGA 1730
        CircularRNA -------------------------------------------------------------------------------- 457

                                    5.380                 5.400                 5.420                 5.440
Genomic(MLOC_60294) TACGTTGCTCCACAGACACAGCCTGGCTATGGTCCGCAGCCACCACAACAGGGCACGTATGGTCAGGGTGGTTATGGGCA 5440
   cDNA(MLOC_60294) TACGTTGCTCCACAGACACAGCCTGGCTATGGTCCGCAGCCACCACAACAGGGCACGTATGGTCAGGGTGGTTATGGGCA 1810
        CircularRNA -------------------------------------------------------------------------------- 457

                                    5.460                 5.480                 5.500                 5.520
Genomic(MLOC_60294) GCCGCCTCTACAGGCTCAGAAGCTGCCTTCATCTGCTCCTACTTATGGACAGGCACCGCCTGCTCAGGCTGGATATGGGC 5520
   cDNA(MLOC_60294) GCCGCCTCTACAGGCTCAGAAGCTGCCTTCATCTGCTCCTACTTATGGACAGGCACCGCCTGCTCAGGCTGGATATGGGC 1890
        CircularRNA -------------------------------------------------------------------------------- 457

                                    5.540                 5.560                 5.580                 5.600
Genomic(MLOC_60294) AGTATGGATACAGTCAGCCAGGCTATGGTGCTCCACCGCCTTACCCTGGTGCACCTACTGCTAGCCAGCCAGGCTATGGC 5600
   cDNA(MLOC_60294) AGTATGGATACAGTCAGCCAGGCTATGGTGCTCCACCGCCTTACCCTGGTGCACCTACTGCTAGCCAGCCAGGCTATGGC 1970
        CircularRNA -------------------------------------------------------------------------------- 457

                                    5.620                 5.640                 5.660                 5.680
Genomic(MLOC_60294) CAGCAGCAGTCATACGGTGATCCTTATGCTACTGGTAGCTATGGGCAGCCTACTGCTTATTCTACTGAAGCCACAGCACC 5680
   cDNA(MLOC_60294) CAGCAGCAGTCATACGGTGATCCTTATGCTACTGGTAGCTATGGGCAGCCTACTGCTTATTCTACTGAAGCCACAGCACC 2050
        CircularRNA -------------------------------------------------------------------------------- 457

                                    5.700                 5.720                 5.740                 5.760
Genomic(MLOC_60294) TGCTGCGTCCCAGGATCAATCTGCCGCCGCACCTGCGCCTACCACAGCAGCTGCCGCTCCTGCTACTGCCAACAGTGGTG 5760
   cDNA(MLOC_60294) TGCTGCGTCCCAGGATCAATCTGCCGCCGCACCTGCGCCTACCACAGCAGCTGCCGCTCCTGCTACTGCCAACAGTGGTG 2130
        CircularRNA -------------------------------------------------------------------------------- 457

                                    5.780                 5.800                 5.820                 5.840
Genomic(MLOC_60294) CCCCCCAAACTTCTCCGAGTTGAGCTACCTGCCTCAGCTGCATCTGAAATCTGGTTTCCTTGGCTCTGCTGCTTGAAGTA 5840
   cDNA(MLOC_60294) CCCCCCAAACTTCTCCGAGTTGAGCTACCTGCCTCAGCTGCATCTGAAATCTGGTTTCCTTGGCTCTGCTGCTTGAAGTA 2210
        CircularRNA -------------------------------------------------------------------------------- 457

                                    5.860                 5.880                 5.900                 5.920
Genomic(MLOC_60294) TAATCCTCAGTTTACCCATGTTATCTTACTGATTGCTTTATTGTGAACTTTATATATTGCTATATTATTATCCTTGTTTT 5920
   cDNA(MLOC_60294) TAATCCTCAGTTTACCCATGTTATCTTACTGATTGCTTTATTGTGAACTTTATATATTGCTATATTATTATCCTTGTTTT 2290
        CircularRNA -------------------------------------------------------------------------------- 457

                                    5.940                 5.960                 5.980                 6.000
Genomic(MLOC_60294) GCTGCTCTCGAACTCGAACTGCTTGTGATCTCTGGTGCAACCTATACTAGGTTTGGATAATCTGAAATGCAACTGTATTC 6000
   cDNA(MLOC_60294) GCTGCTCTCGAACTCGAACTGCTTGTGATCTCTGGTGCAACCTATACTAGGTTTGGATAATCTGAAATGCAACTGTATTC 2370
        CircularRNA -------------------------------------------------------------------------------- 457

                                    6.020                 6.040                 6.060                 6.080
Genomic(MLOC_60294) AATTTTGCTGCTTAACGTGTTGTTGTAGTTATGTGGATGGTATGTATTTGACATCAATTTCTAGCAGCACTTGATTCACT 6080
   cDNA(MLOC_60294) AATTTTGCTGCTTAACGTGTTGTTGTAGTTATGTGGATGGTATGTATTTGACATCAATTTCTAGCAGCACTTGATTCACT 2450
        CircularRNA -------------------------------------------------------------------------------- 457

                                    6.100                 6.120                 6.140                 6.160
Genomic(MLOC_60294) TGTTTTTAAGCTTGAAAACGGCCTCACAATATGAGTTCTCTTTGAAGTTCTCTGTGAAACAAACATAAGTCTGATATTTC 6160
   cDNA(MLOC_60294) TGTTTTTAAGCTTG------------------------------------------------------------------ 2464
        CircularRNA -------------------------------------------------------------------------------- 457

                                    6.180                 6.200                 6.220
Genomic(MLOC_60294) AGGAGGAGCTTCTGTGGCAGAGAGATATGGCTAGATCGTACTACCTTTTTTCTGCTAATATTACAAGGC 6229
   cDNA(MLOC_60294) --------------------------------------------------------------------- 2464
        CircularRNA --------------------------------------------------------------------- 457
```

Sec-independent protein translocase _circular RNA

(ID: Morex_contig_106453:2137-3175)

TTACGAATCCAGGGCAATCAACCACTATCTAAAAACCCAGGTTGGACAACTGCGTGCTGGGG
GATTAGCAGGGGATTTTGTCGTAAAGGAATTACGGAAATGGAAAAGGGACTTCAAAGCTCAGAA
GAGACGGTCCCACTTCTATCAGGGCCTTCTCGGGGTCTTGAGCAATGTTAATTTGCATTGATTG
CAAACAGGGTGCGGGATTCTCTGACGCAAACAAAGAAAAGGTCCCGGAGAAAGGAATCAGTCT
CTTCAGTCCGGCGCATGCCTCAAATTCATTTTTCCTTTGAATTACTCATTTTTTTGAATTTCGCAC
CGGAAACTTTTCTAGGAGAAGTTCGAATCCGTTCCGTTCGGATATTGATCGGTCTTGGTTTGACA
TGGTTTACGCGTTACTGGTTCCCGGAAGAGTCAATTTCTCCATTAGCTAAACCCTTTATTACCCT
ACCTTTGGACTCGTATTTTGTTTGTACACAATCAACGGAGGCCCCCCGACATATGTTGCAACGT
CTTCAATAGCATGCTCTTACTTCGTCTTTCCCTTAATAAGTCATCAAATTTGGTGCTTTTCGATCC
CCAGTTGCTATGGGGAACAAAGGCAGAAATACAATAGAATCCTCCATTTAAGTGGTTCTCGCTTC
TCCTTGTTCCTGCTCCTAACTCCTCCCCGGGTAGTTCCCAATGTTTGGCACTTTCCATACTTCGT
GGGTGCAACATCAACAAATTCGCTCATGATCAAGTTACAACCTAAGATCTATGACTATATTATGTT
AACTGTTCGTATTTTGTTCATTCCATCGGTATGCTCCCAGGTACCTGTAATTGTGATCTGTTTGCC
AGAACCAAGGGGTCTTTCTGTGGAAACCTTCACGAGCAATCGTCGTTTTTTGATGGTTTTTCCGC
TTTTCACAGCTGCTCTTTCCACACCCCCGGATATCTGGTGCCAAACCGTCGCCCCTTTCCTTATA
TATTCGATAATCGAGTTTGCTATC**TTTGTGGCATTGATTGTACAAGTTCGTGAAGAGGGCTGGA
CGA**

The nucleotides of junction-region are underlined. The nucleotides of junction-region which are supported by the junction-spaning sequencing reads are shown in red. Introns are not shown if the absence is supported by sequencing reads. In the absence of supporting sequencing reads, the intronic nucleotides are shown as N.

Structural relationship between the circular RNA and its parental gene

```
                   20                  40                  60                  80
Genomic(MLOC_816) CTCATTTCGAATTTCATCTTAGTATCGGTAATGCCCCGCCCAAAAGTATTTCATCGCAATCACACGCTAGCTTGGTTGGA 80
cDNA(MLOC_816)    CTCATTTCGAATTTCATCTTAGTATCGGTAATGCCCCGCCCAAAAGTATTTCATCGCAATCACACGCTAGCTTGGTTGGA 80
CircularRNA       -------------------------------------------------------------------------------- -

                   100                 120                 140                 160
Genomic(MLOC_816) AAAGCTCGTTTGCACCCACTAACTAATACCTTCTCGTCAGTATTGAATTCTCCATGCTTTCGCCTTGCTTTGTAGTCATT 160
cDNA(MLOC_816)    AAAGCTCGTTTGCACCCACTAACTAATACCTTCTCGTCAGTATTGAATTCTCCATGCTTTCGCCTTGCTTTGTAGTCATT 160
CircularRNA       -------------------------------------------------------------------------------- -

                   180                 200                 220                 240
Genomic(MLOC_816) TTCTATGAGTAATCGGTCCGAACTCAATTTCAAGAAAAAACTCTATCTCGCCTGTGGCCAAAAGTAGTGAAAATGGTATT 240
cDNA(MLOC_816)    TTCTATGAGTAATCGGTCCGAACTCAATTTCAAGAAAAAACTCTATCTCGCCTGTGGCCAAAAGTAGTGAAAATGGTATT 240
CircularRNA       -------------------------------------------------------------------------------- -

                   260                 280                 300                 320
Genomic(MLOC_816) TAGCTTAGTCAATATACAACACAACTGCTGATTCTTCGCGAGATGAAAACCCTTTGTCCTCCGTCTGCAGGGGCAATTCC 320
cDNA(MLOC_816)    TAGCTTAGTCAATATACAACACAACTGCTGATTCTTCGCGAGATGAAAACCCTTTGTCCTCCGTCTGCAGGGGCAATTCC 320
CircularRNA       -------------------------------------------------------------------------------- -

                   340                 360                 380                 400
Genomic(MLOC_816) TGAGAAAAGTGCTACTAGCCCTTGTTTTGGTCATGTCCACAGGCCTTTCCATTCGTCTTTATTCGTCTTTCTTTCGGCAA 400
cDNA(MLOC_816)    TGAGAAAAGTGCTACTAGCCCTTGTTTTGGTCATGTCCACAGGCCTTTCCATTCGTCTTTATTCGTCTTTCTTTCGGCAA 400
CircularRNA       -------------------------------------------------------------------------------- -

                   420                 440                 460                 480
Genomic(MLOC_816) TCTATGCATAATTCTACTTCTTGAACCACCGAATCCGCTTTTGAAAATGGATAACTAGCTCCATTCTGCGTCAAGTACTA 480
cDNA(MLOC_816)    TCTATGCATAATTCTACTTCTTGAACCACCGAATCCGCTTTTGAAAATGGATAACTAGCTCCATTCTGCGTCAAGTACTA 480
CircularRNA       -------------------------------------------------------------------------------- -

                   500                 520                 540                 560
Genomic(MLOC_816) AGGATGGGCCCAAAGCACACTTTTGAAAGGCATCGGTCACCTTAAAAACCCAAAAAGTAGGAAGTCTAGCGTGCTTCTTT 560
cDNA(MLOC_816)    AGGATGGGCCCAAAGCACACTTTTGAAAGGCATCGGTCACCTTAAAAACCCAAAAAGTAGGAAGTCTAGCGTGCTTCTTT 560
CircularRNA       -------------------------------------------------------------------------------- -

                   580                 600                 620                 640
Genomic(MLOC_816) AGTGCTGCTCTTCGATCAGATGCTTTCTCATTGAACGAGGCATATTAAAGTGAAAGAAGTTCCAGGTCGAGTCCCGAGAA 640
cDNA(MLOC_816)    AGTGCTGCTCTTCGATCAGATGCTTTCTCATTGAACGAGGCATATTAAAGTGAAAGAAGTTCCAGGTCGAGTCCCGAGAA 640
CircularRNA       -------------------------------------------------------------------------------- -

                   660                 680                 700                 720
Genomic(MLOC_816) GGCGATAGTTGATTTTGATCATGAAGTTGGCACTGGACTTTGGAATTGATTCTGGACCCCCACCTGCTCAGGACGCCCCG 720
cDNA(MLOC_816)    GGCGATAGTTGATTTTGATCATGAAGTTGGCACTGGACTTTGGAATTGATTCTGGACCCCCACCTGCTCAGGACGCCCCG 720
CircularRNA       -------------------------------------------------------------------------------- -

                   740                 760                 780                 800
Genomic(MLOC_816) CCTGCTCAGGACGATCCCATTACGGATTTTCTGGAGGGGGACACGCTTGACCCAGCGCAAAAACGGGAATTCAGAAATAT 800
cDNA(MLOC_816)    CCTGCTCAGGACGATCCCATTACGGATTTTCTGGAGGGGGACACGCTTGACCCAGCGCAAAAACGGGAATTCAGAAATAT 800
CircularRNA       -------------------------------------------------------------------------------- -

                   820                 840                 860                 880
Genomic(MLOC_816) TCTGAATTTCTTGAAGACCAAAAGCAGAACGAACCTTCAAAATGAGCGAATCGTAATAAAAAGATGTTGGGGGGGCGAGG 880
cDNA(MLOC_816)    TCTGAATTTCTTGAAGACCAAAAGCAGAACGAACCTTCAAAATGAGCGAATCGTAATAAAAAGATGTTGGGGGGGCGAGG 880
CircularRNA       -------------------------------------------------------------------------------- -

                   900                 920                 940                 960
Genomic(MLOC_816) GTAGTAGTTACGAATCCAGGGCAATCAACCACTATCTAAAAACCCAGGTTGGACAACTGCGTGCTGGGGGATTAGCAGGG 960
cDNA(MLOC_816)    GTAGTAGTTACGAATCCAGGGCAATCAACCACTATCTAAAAACCCAGGTTGGACAACTGCGTGCTGGGGGATTAGCAGGG 960
CircularRNA       -------TTACGAATCCAGGGCAATCAACCACTATCTAAAAACCCAGGTTGGACAACTGCGTGCTGGGGGATTAGCAGGG 73

                   980                 1.000               1.020               1.040
Genomic(MLOC_816) GATTTTGTCGTAAAGGAATTACGGAAATGGAAAAGGGACTTCAAAGCTCAGAAGAGACGGTCCCACTTCTATCAGGGCCT 1040
cDNA(MLOC_816)    GATTTTGTCGTAAAGGAATTACGGAAATGGAAAAGGGACTTCAAAGCTCAGAAGAGACGGTCCCACTTCTATCAGGGCCT 1040
CircularRNA       GATTTTGTCGTAAAGGAATTACGGAAATGGAAAAGGGACTTCAAAGCTCAGAAGAGACGGTCCCACTTCTATCAGGGCCT 153

                   1.060               1.080               1.100               1.120
Genomic(MLOC_816) TCTCGGGGTCTTGAGCAATGTTAATTTGCATTGATTGCAAACAGGGTGCGGGATTCTCTGACGCAAACAAAGAAAAGGTC 1120
cDNA(MLOC_816)    TCTCGGGGTCTTGAGCAATGTTAATTTGCATTGATTGCAAACAGGGTGCGGGATTCTCTGACGCAAACAAAGAAAAGGTC 1120
CircularRNA       TCTCGGGGTCTTGAGCAATGTTAATTTGCATTGATTGCAAACAGGGTGCGGGATTCTCTGACGCAAACAAAGAAAAGGTC 233

                   1.140               1.160               1.180               1.200
Genomic(MLOC_816) CCGGAGAAAGGAATCAGTCTCTTCAGTCCGGCGCATGCCTCAAATTCATTTTTCCTTTGAATTACTCATTTTTTTGAATT 1200
cDNA(MLOC_816)    CCGGAGAAAGGAATCAGTCTCTTCAGTCCGGCGCATGCCTCAAATTCATTTTTCCTTTGAATTACTCATTTTTTTGAATT 1200
CircularRNA       CCGGAGAAAGGAATCAGTCTCTTCAGTCCGGCGCATGCCTCAAATTCATTTTTCCTTTGAATTACTCATTTTTTTGAATT 313

                   1.220               1.240               1.260               1.280
Genomic(MLOC_816) TCGCACCGGAAACTTTTCTAGGAGAAGTTCGAATCCGTTCCGTTCGGATATTGATCGGTCTTGGTTTGACATGGTTTACG 1280
cDNA(MLOC_816)    TCGCACCGGAAACTTTTCTAGGAGAAGTTCGAATCCGTTCCGTTCGGATATTGATCGGTCTTGGTTTGACATGGTTTACG 1280
CircularRNA       TCGCACCGGAAACTTTTCTAGGAGAAGTTCGAATCCGTTCCGTTCGGATATTGATCGGTCTTGGTTTGACATGGTTTACG 393

                   1.300               1.320               1.340               1.360
Genomic(MLOC_816) CGTTACTGGTTCCCGGAAGAGTCAATTTCTCCATTAGCTAAACCCTTTATTACCCTACCTTTGGACTCGTATTTTGTTTG 1360
cDNA(MLOC_816)    CGTTACTGGTTCCCGGAAGAGTCAATTTCTCCATTAGCTAAACCCTTTATTACCCTACCTTTGGACTCGTATTTTGTTTG 1360
CircularRNA       CGTTACTGGTTCCCGGAAGAGTCAATTTCTCCATTAGCTAAACCCTTTATTACCCTACCTTTGGACTCGTATTTTGTTTG 473

                   1.380               1.400               1.420               1.440
Genomic(MLOC_816) TACACAATCAACGGAGGCCCCCCCGACATATGTTGCAACGTCTTCAATAGCATGCTCTTACTTCGTCTTTCCCTTAATAA 1440
cDNA(MLOC_816)    TACACAATCAACGGAGGCCCCCCCGACATATGTTGCAACGTCTTCAATAGCATGCTCTTACTTCGTCTTTCCCTTAATAA 1440
CircularRNA       TACACAATCAACGGAGGCCCCCCCGACATATGTTGCAACGTCTTCAATAGCATGCTCTTACTTCGTCTTTCCCTTAATAA 553

                   1.460               1.480               1.500               1.520
Genomic(MLOC_816) GTCATCAAATTTGGTGCTTTTCGATCCCCAGTTGCTATGGGGAACAAAGGCAGAAATACAATAGAATCCTCCATTTAAGT 1520
cDNA(MLOC_816)    GTCATCAAATTTGGTGCTTTTCGATCCCCAGTTGCTATGGGGAACAAAGGCAGAAATACAATAGAATCCTCCATTTAAGT 1520
CircularRNA       GTCATCAAATTTGGTGCTTTTCGATCCCCAGTTGCTATGGGGAACAAAGGCAGAAATACAATAGAATCCTCCATTTAAGT 633

                   1.540               1.560               1.580               1.600
Genomic(MLOC_816) GGTTCTCGCTTCTCCTTGTTCCTGCTCCTAACTCCTCCCCGGGTAGTTCCCAATGTTTGGCACTTTCCATACTTCGTGGG 1600
cDNA(MLOC_816)    GGTTCTCGCTTCTCCTTGTTCCTGCTCCTAACTCCTCCCCGGGTAGTTCCCAATGTTTGGCACTTTCCATACTTCGTGGG 1600
CircularRNA       GGTTCTCGCTTCTCCTTGTTCCTGCTCCTAACTCCTCCCCGGGTAGTTCCCAATGTTTGGCACTTTCCATACTTCGTGGG 713
```

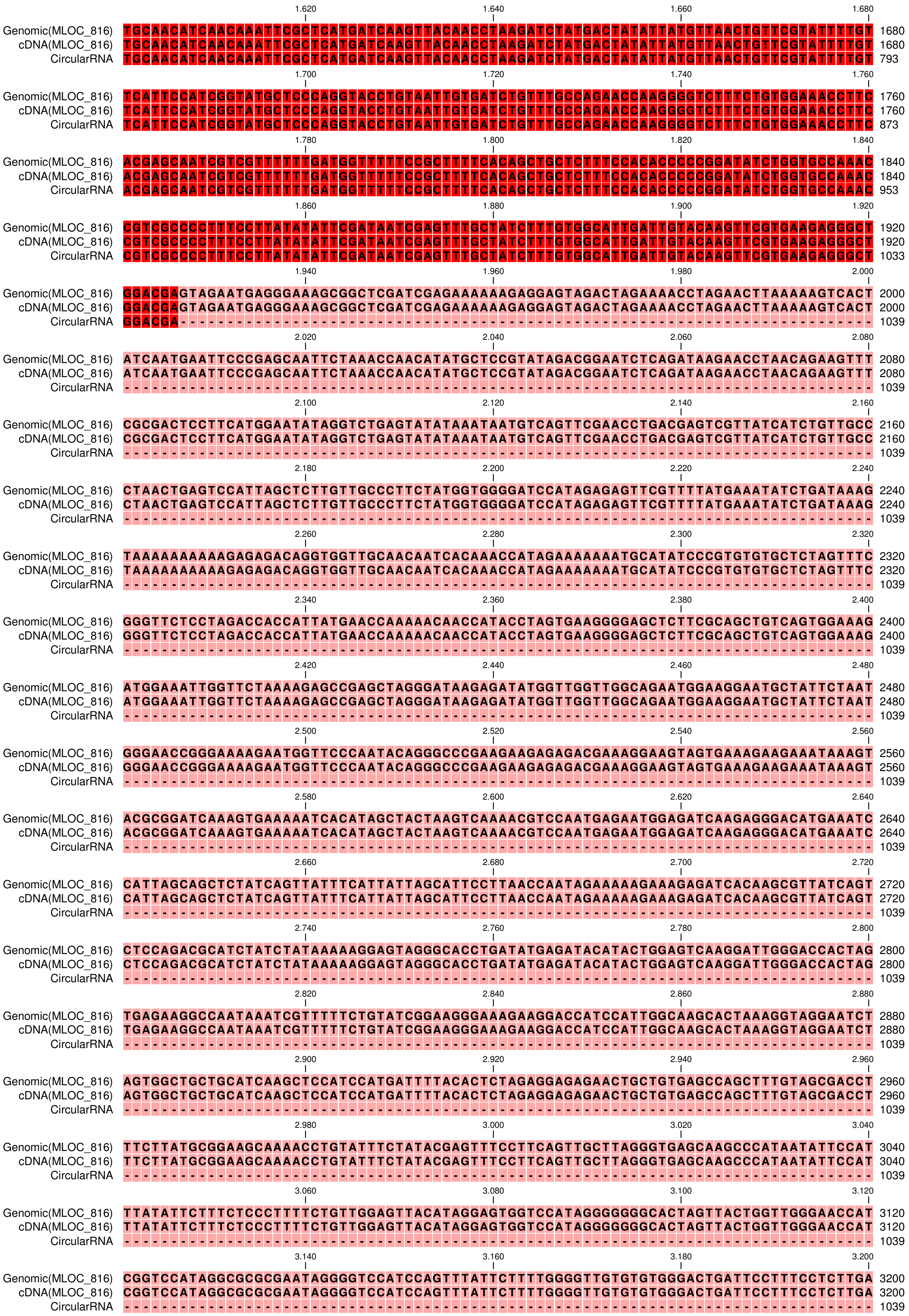
Genomic(MLOC_816)
cDNA(MLOC_816)
CircularRNA
1.620
3.200
1680
1680
793
3200
3200
1039

```
                                         3.220                3.240                3.260                3.280
                                           |                    |                    |                    |
Genomic(MLOC_816) TGTGGAAAAATAGGCTCTTAGGGCATATGCTGCCTTTACCGGAAAAAGAAAGTCAACGGACTTCATGGACGAGGAGGTGG 3280
   cDNA(MLOC_816) TGTGGAAAAATAGGCTCTTAGGGCATATGCTGCCTTTACCGGAAAAAGAAAGTCAACGGACTTCATGGACGAGGAGGTGG 3280
      CircularRNA -------------------------------------------------------------------------------- 1039

                                         3.300                3.320                3.340                3.360
                                           |                    |                    |                    |
Genomic(MLOC_816) AGGATGAATGGTATGGCTTGCTCACTTGCTTAAAAACTTATTTTCCAAAATAAGGAGAAAGAATATAAATCTCCGAATGG 3360
   cDNA(MLOC_816) AGGATGAATGGTATGGCTTGCTCACTTGCTTAAAAACTTATTTTCCAAAATAAGGAGAAAGAATATAAATCTCCGAATGG 3360
      CircularRNA -------------------------------------------------------------------------------- 1039

                                         3.380                3.400                3.420                3.440
                                           |                    |                    |                    |
Genomic(MLOC_816) AGAAAAGCATATTCTAGTATTTGCCCATAAGCTTATTCCAAATAGGAGTGCTACATAAGCATATGCTAAAGGGGGAGGAA 3440
   cDNA(MLOC_816) AGAAAAGCATATTCTAGTATTTGCCCATAAGCTTATTCCAAATAGGAGTGCTACATAAGCATATGCTAAAGGGGGAGGAA 3440
      CircularRNA -------------------------------------------------------------------------------- 1039

                                         3.460                3.480                3.500                3.520
                                           |                    |                    |                    |
Genomic(MLOC_816) TCCATTTATTATTTATTTCTTAGCCTGGGCCCAACTATGTACAGAAATCTATCTATTATTGCCTGCATCGACAGCAAAGT 3520
   cDNA(MLOC_816) TCCATTTATTATTTATTTCTTAGCCTGGGCCCAACTATGTACAGAAATCTATCTATTATTGCCTGCATCGACAGCAAAGT 3520
      CircularRNA -------------------------------------------------------------------------------- 1039

                                         3.540                3.560
                                           |                    |
Genomic(MLOC_816) AAACTCTCGCTGATGATAGAAGAACAACTCTTAGGGCAGTTGCAAGGAAACTATGAG 3577
   cDNA(MLOC_816) AAACTCTCGCTGATGATAGAAGAACAACTCTTAGGGCAGTTGCAAGGAAACTATGAG 3577
      CircularRNA --------------------------------------------------------- 1039
```

Ubiquitin-specific protease 17_circular RNA (ID: Ch7:307483097-307485269)

CTCATTCAAATGCCAGATATCCCACTGGAGAAAAGGTCACAAAGAGGAATGTCATCCACCAAA
TGCTAATGCTAAGCCAGATGACGGAGCTCCAATATTGGTTGCAAAGGAAGGAAGTCTTGAACAC
CGAAGAGCTTATGAAGAGAATGTAGTGCCTGGTGTACAACCAGTGGCTGAAATTAGCGTACCGG
TTGCGTCTGAGTCTGAAACTTCTGGCACAAACCATGTTGTGAAGAGCTCAAATGATAAAAGGAAA
GACACGCTTTTCAAAGAAGTATCTGCTACCACAGAGATCCCTGAAAAGGGCGATTCCGACAATG
TGGTCAAACTTCGTCAAATTACCCCTCAAGTAGCATCCTTTGACAGTACAAGGACAGAATCAAAT
ACCAAATCTACAAATTTTGTTGAAAACAGCTCTTACATGAAGGACTCAGATGAGATATTGGTTCGT
AAATCTCAAGCTTCTGCACCAAAGATGAATGGCCTTACCAGTTCCATCCATGAAGATATATCTGT
TCATTGCAAGGACCATGGTAACACTGAGGATGCTGATGTTAGTGAGGATTGCTCTCGAATTAATC
ATATTAGAAAACCTGCAGATAGGAGCAATTCCCAAACAGTTGCAACTGCCGTACCAGCACCCAA
AAGTTCTAGGACTTCTCTTTGCGTGGAAGTTGAACCGTGCAAAATAAAAGCTACTGGCAAAGAAA
AGTCTGAAGGCTCGAAATTAGTATCATCAGTATTAGCTGTTGATAAGGTTTCTTCCATCCATGGT
GGCCGTCCTGTAGCACCTAATCCATCAAAAAGGGCTGATAATCCAACAGATAGAGGAGGTTCAG
CAGCAAATAACCTTGCGACATCTCTGAAGAAAATTGTCAGGCAACAAACAACACCAAAAGTTGTG
AGGCATTATCCTTCAGAGTTGNNN…NNNACACTTTTTCCATATGATGTTTTCATCAAGCTCTACG
ACAAGGTTGAATTGCGCCCTTTTGGTCTTCATAACCTTGGCAACAGNNN…NNNCTGCTATGCAA
ATGTTGTTCTTCAGTGCTTGATGTTTACTCGACCACTTACAACGTATCTTCTGGGAGGGTTTCATT
CGAAAAATTGTNNN…NNNTCCAAAAAGGCATGGTGTTTCATGTGTGAATTTGAAAGACTTATTGT
GGAGGGCAAGCAGGGCAAGACTGCTTTATCACCAACTGGGATACTGTCCCATTTGCACGAGATT
GGAAGTAGCT**TTGGTCCTGGTAAACAAGAAGATGCTCATGAGTTCCTTCG**

The nucleotides of junction-region are underlined. The nucleotides of junction-region which are supported by the junction-spaning sequencing reads are shown in red. Introns are not shown if the absence is supported by sequencing reads. In the absence of supporting sequencing reads, the intronic nucletides are shown as N.

Structural relationship between the circular RNA and its parental gene

```
                 20                  40                  60                  80
Genomic          TCATTTTGCTCCGTATGTAGCCTTTTAGTGAAATCTCCAAAAAGACTTATATTTAGGAACGGAGGGAGTACATGAATAGT 80
cDNA(predicted)  -------------------------------------------------------------------------------- -
CircularRNA      -------------------------------------------------------------------------------- -

                 100                 120                 140                 160
Genomic          AAACATGCAAACATGCTATGTGTCGAAAATCCTAAACTGCGGTTGAATATTGTGCGTGGTGTATAAAAATCTAATCGTGT 160
cDNA(predicted)  -------------------------------------------------------------------------------- -
CircularRNA      -------------------------------------------------------------------------------- -

                 180                 200                 220                 240
Genomic          TACTTGTTCAACCTTGTGACACCTTTCCTTTTGTAGCTCATTCAAATGCCAGATATCCCACTGGAGAAAAGGTCACAAAG 240
cDNA(predicted)  ------------------------------------CTCATTCAAATGCCAGATATCCCACTGGAGAAAAGGTCACAAAG 44
CircularRNA      ------------------------------------CTCATTCAAATGCCAGATATCCCACTGGAGAAAAGGTCACAAAG 44

                 260                 280                 300                 320
Genomic          AGGAATGTCATCCACCAAATGCTAATGCTAAGCCAGATGACGGAGCTCCAATATTGGTTGCAAAGGAAGGAAGTCTTGAA 320
cDNA(predicted)  AGGAATGTCATCCACCAAATGCTAATGCTAAGCCAGATGACGGAGCTCCAATATTGGTTGCAAAGGAAGGAAGTCTTGAA 124
CircularRNA      AGGAATGTCATCCACCAAATGCTAATGCTAAGCCAGATGACGGAGCTCCAATATTGGTTGCAAAGGAAGGAAGTCTTGAA 124

                 340                 360                 380                 400
Genomic          CACCGAAGAGCTTATGAAGAGAATGTAGTGCCTGGTGTACAACCAGTGGCTGAAATTAGCGTACCGGTTGCGTCTGAGTC 400
cDNA(predicted)  CACCGAAGAGCTTATGAAGAGAATGTAGTGCCTGGTGTACAACCAGTGGCTGAAATTAGCGTACCGGTTGCGTCTGAGTC 204
CircularRNA      CACCGAAGAGCTTATGAAGAGAATGTAGTGCCTGGTGTACAACCAGTGGCTGAAATTAGCGTACCGGTTGCGTCTGAGTC 204

                 420                 440                 460                 480
Genomic          TGAAACTTCTGGCACAAACCATGTTGTGAAGAGCTCAAATGATAAAAGGAAAGACACGCTTTTCAAAGAAGTATCTGCTA 480
cDNA(predicted)  TGAAACTTCTGGCACAAACCATGTTGTGAAGAGCTCAAATGATAAAAGGAAAGACACGCTTTTCAAAGAAGTATCTGCTA 284
CircularRNA      TGAAACTTCTGGCACAAACCATGTTGTGAAGAGCTCAAATGATAAAAGGAAAGACACGCTTTTCAAAGAAGTATCTGCTA 284

                 500                 520                 540                 560
Genomic          CCACAGAGATCCCTGAAAAGGGCGATTCCGACAATGTGGTCAAACTTCGTCAAATTACCCCTCAAGTAGCATCCTTTGAC 560
cDNA(predicted)  CCACAGAGATCCCTGAAAAGGGCGATTCCGACAATGTGGTCAAACTTCGTCAAATTACCCCTCAAGTAGCATCCTTTGAC 364
CircularRNA      CCACAGAGATCCCTGAAAAGGGCGATTCCGACAATGTGGTCAAACTTCGTCAAATTACCCCTCAAGTAGCATCCTTTGAC 364

                 580                 600                 620                 640
Genomic          AGTACAAGGACAGAATCAAATACCAAATCTACAAATTTTGTTGAAAACAGCTCTTACATGAAGGACTCAGATGAGATATT 640
cDNA(predicted)  AGTACAAGGACAGAATCAAATACCAAATCTACAAATTTTGTTGAAAACAGCTCTTACATGAAGGACTCAGATGAGATATT 444
CircularRNA      AGTACAAGGACAGAATCAAATACCAAATCTACAAATTTTGTTGAAAACAGCTCTTACATGAAGGACTCAGATGAGATATT 444

                 660                 680                 700                 720
Genomic          GGTTCGTAAATCTCAAGCTTCTGCACCAAAGATGAATGGCCTTACCAGTTCCATCCATGAAGATATATCTGTTCATTGCA 720
cDNA(predicted)  GGTTCGTAAATCTCAAGCTTCTGCACCAAAGATGAATGGCCTTACCAGTTCCATCCATGAAGATATATCTGTTCATTGCA 524
CircularRNA      GGTTCGTAAATCTCAAGCTTCTGCACCAAAGATGAATGGCCTTACCAGTTCCATCCATGAAGATATATCTGTTCATTGCA 524

                 740                 760                 780                 800
Genomic          AGGACCATGGTAACACTGAGGATGCTGATGTTAGTGAGGATTGCTCTCGAATTAATCATATTAGAAAACCTGCAGATAGG 800
cDNA(predicted)  AGGACCATGGTAACACTGAGGATGCTGATGTTAGTGAGGATTGCTCTCGAATTAATCATATTAGAAAACCTGCAGATAGG 604
CircularRNA      AGGACCATGGTAACACTGAGGATGCTGATGTTAGTGAGGATTGCTCTCGAATTAATCATATTAGAAAACCTGCAGATAGG 604

                 820                 840                 860                 880
Genomic          AGCAATTCCCAAACAGTTGCAACTGCCGTACCAGCACCCAAAAGTTCTAGGACTTCTCTTTGCGTGGAAGTTGAACCGTG 880
cDNA(predicted)  AGCAATTCCCAAACAGTTGCAACTGCCGTACCAGCACCCAAAAGTTCTAGGACTTCTCTTTGCGTGGAAGTTGAACCGTG 684
CircularRNA      AGCAATTCCCAAACAGTTGCAACTGCCGTACCAGCACCCAAAAGTTCTAGGACTTCTCTTTGCGTGGAAGTTGAACCGTG 684

                 900                 920                 940                 960
Genomic          CAAAATAAAAGCTACTGGCAAAGAAAAGTCTGAAGGCTCGAAATTAGTATCATCAGTATTAGCTGTTGATAAGGTTTCTT 960
cDNA(predicted)  CAAAATAAAAGCTACTGGCAAAGAAAAGTCTGAAGGCTCGAAATTAGTATCATCAGTATTAGCTGTTGATAAGGTTTCTT 764
CircularRNA      CAAAATAAAAGCTACTGGCAAAGAAAAGTCTGAAGGCTCGAAATTAGTATCATCAGTATTAGCTGTTGATAAGGTTTCTT 764

                 980                 1.000               1.020               1.040
Genomic          CCATCCATGGTGGCCGTCCTGTAGCACCTAATCCATCAAAAAGGGCTGATAATCCAACAGATAGAGGAGGTTCAGCAGCA 1040
cDNA(predicted)  CCATCCATGGTGGCCGTCCTGTAGCACCTAATCCATCAAAAAGGGCTGATAATCCAACAGATAGAGGAGGTTCAGCAGCA 844
CircularRNA      CCATCCATGGTGGCCGTCCTGTAGCACCTAATCCATCAAAAAGGGCTGATAATCCAACAGATAGAGGAGGTTCAGCAGCA 844

                 1.060               1.080               1.100               1.120
Genomic          AATAACCTTGCGACATCTCTGAAGAAAATTGTCAGGCAACAAACAACACCAAAAGTTGTGAGGCATTATCCTTCAGAGTT 1120
cDNA(predicted)  AATAACCTTGCGACATCTCTGAAGAAAATTGTCAGGCAACAAACAACACCAAAAGTTGTGAGGCATTATCCTTCAGAGTT 924
CircularRNA      AATAACCTTGCGACATCTCTGAAGAAAATTGTCAGGCAACAAACAACACCAAAAGTTGTGAGGCATTATCCTTCAGAGTT 924

                 1.140               1.160               1.180               1.200
Genomic          GGTAAATATAACTGAATGACTCTGCAGTTAGTCATTTATTTTCTTTTACTTGCTACTAACTTCTGCTTTTCAGACACTTT 1200
cDNA(predicted)  G------------------------------------------------------------------------ACACTTT 932
CircularRNA      GNNNNNNNNNNNNNNNNNNNNNNNNNNNNNNNNNNNNNNNNNNNNNNNNNNNNNNNNNNNNNNNNNNNNNNNNACACTTT 1004

                 1.220               1.240               1.260               1.280
Genomic          TTCCATATGATGTTTTCATCAAGCTCTACGACAAGGTTGAATTGCGCCCTTTTGGTCTTCATAACCTTGGCAACAGGTAA 1280
cDNA(predicted)  TTCCATATGATGTTTTCATCAAGCTCTACGACAAGGTTGAATTGCGCCCTTTTGGTCTTCATAACCTTGGCAACAG---- 1008
CircularRNA      TTCCATATGATGTTTTCATCAAGCTCTACGACAAGGTTGAATTGCGCCCTTTTGGTCTTCATAACCTTGGCAACAGNNNN 1084

                 1.300               1.320               1.340               1.360
Genomic          ACATTGAATTACCGTTTCTGAAATGTAATATGTTGAGAGATTAACTGCATGCTTTGTATTTATTTGAAGTCCATCCCATA 1360
cDNA(predicted)  -------------------------------------------------------------------------------- 1008
CircularRNA      NNNNNNNNNNNNNNNNNNNNNNNNNNNNNNNNNNNNNNNNNNNNNNNNNNNNNNNNNNNNNNNNNNNNNNNNNNNNNNNN 1164

                 1.380               1.400               1.420               1.440
Genomic          TTTTTTTGTGTAGGCATCTCTAGCTTTGTAATCTGTTTGCAAGTTAGTTTTTACTTCTGTTGGAGTCTTGTTGACATATA 1440
cDNA(predicted)  -------------------------------------------------------------------------------- 1008
CircularRNA      NNNNNNNNNNNNNNNNNNNNNNNNNNNNNNNNNNNNNNNNNNNNNNNNNNNNNNNNNNNNNNNNNNNNNNNNNNNNNNNN 1244

                 1.460               1.480               1.500               1.520
Genomic          AGTAGATCTTATCTGGTTTGTTTACCTAGGGCCATCATTAATTTAATGAGCTAAAGATGGACTTTGTTTCCCACCCACAA 1520
cDNA(predicted)  -------------------------------------------------------------------------------- 1008
CircularRNA      NNNNNNNNNNNNNNNNNNNNNNNNNNNNNNNNNNNNNNNNNNNNNNNNNNNNNNNNNNNNNNNNNNNNNNNNNNNNNNNN 1324

                 1.540               1.560               1.580               1.600
Genomic          AAATATCTGAAGTGATCAGACAAATATGCTCCAGCTTTATTGATCCAGCATGCTTTGATAGTGTTTAATGGCATGATAGT 1600
cDNA(predicted)  -------------------------------------------------------------------------------- 1008
CircularRNA      NNNNNNNNNNNNNNNNNNNNNNNNNNNNNNNNNNNNNNNNNNNNNNNNNNNNNNNNNNNNNNNNNNNNNNNNNNNNNNNN 1404
```

```
                                      1.620                         1.640                         1.660                         1.680
Genomic           GTTACTCTACTACTTTTTGTCCTGCTGTTGATGACTTACCAGTACATGGAACTTTAGGCCTTTGGTTCACAGGCTAGGGA 1680
cDNA(predicted)   -------------------------------------------------------------------------------- 1008
CircularRNA       NNNNNNNNNNNNNNNNNNNNNNNNNNNNNNNNNNNNNNNNNNNNNNNNNNNNNNNNNNNNNNNNNNNNNNNNNNNNNNNN 1484

                                      1.700                         1.720                         1.740                         1.760
Genomic           TATCACAGGAATAGAAAAATCATAGGAAGTGAGATGACATCTATCTCAATTCCTGTAGGGAAAGAGATGTCATTTGATGC 1760
cDNA(predicted)   -------------------------------------------------------------------------------- 1008
CircularRNA       NNNNNNNNNNNNNNNNNNNNNNNNNNNNNNNNNNNNNNNNNNNNNNNNNNNNNNNNNNNNNNNNNNNNNNNNNNNNNNNN 1564

                                      1.780                         1.800                         1.820                         1.840
Genomic           ATAGGATAGAAATTTTTCCATTGAGTCAGTTAATGTTTTTTTTCCTTTGAAATGTGAAGGATTGGTTCCTATTCTACACA 1840
cDNA(predicted)   -------------------------------------------------------------------------------- 1008
CircularRNA       NNNNNNNNNNNNNNNNNNNNNNNNNNNNNNNNNNNNNNNNNNNNNNNNNNNNNNNNNNNNNNNNNNNNNNNNNNNNNNNN 1644

                                      1.860                         1.880                         1.900                         1.920
Genomic           GGAATAGGAATCTTTTCCTATAAACCAAAGGGCTCCAAAGGAAATTTTCCTTTTTCCCAAAATTCTTGTAAACCAAAGGA 1920
cDNA(predicted)   -------------------------------------------------------------------------------- 1008
CircularRNA       NNNNNNNNNNNNNNNNNNNNNNNNNNNNNNNNNNNNNNNNNNNNNNNNNNNNNNNNNNNNNNNNNNNNNNNNNNNNNNNN 1724

                                      1.940                         1.960                         1.980                         2.000
Genomic           GGCCTATGTTAAACAGGATGTTGTCGCCGTAGTTGAGATATTTTGTTTAAGTTGTTATGTGCCGCCCTGTCTAACCTGTT 2000
cDNA(predicted)   -------------------------------------------------------------------------------- 1008
CircularRNA       NNNNNNNNNNNNNNNNNNNNNNNNNNNNNNNNNNNNNNNNNNNNNNNNNNNNNNNNNNNNNNNNNNNNNNNNNNNNNNNN 1804

                                      2.020                         2.040                         2.060                         2.080
Genomic           TTTAACCTGTTTTCTTATTATTTTTCTCTTGCAGCTGCTATGCAAATGTTGTTCTTCAGTGCTTGATGTTTACTCGACCA 2080
cDNA(predicted)   ----------------------------------CTGCTATGCAAATGTTGTTCTTCAGTGCTTGATGTTTACTCGACCA 1054
CircularRNA       NNNNNNNNNNNNNNNNNNNNNNNNNNNNNNNNNNCTGCTATGCAAATGTTGTTCTTCAGTGCTTGATGTTTACTCGACCA 1884

                                      2.100                         2.120                         2.140                         2.160
Genomic           CTTACAACGTATCTTCTGGGAGGGTTTCATTCGAAAAATTGTAAGTGCCTACCAAGTTTCTTATGTCATTCTTTTTCTCC 2160
cDNA(predicted)   CTTACAACGTATCTTCTGGGAGGGTTTCATTCGAAAAATTGT-------------------------------------- 1096
CircularRNA       CTTACAACGTATCTTCTGGGAGGGTTTCATTCGAAAAATTGTNNNNNNNNNNNNNNNNNNNNNNNNNNNNNNNNNNNNNN 1964

                                      2.180                         2.200                         2.220                         2.240
Genomic           ATGTCGCACTTGTTTGATTAACTGCATAGTATCTTGTGTTTTCTAGGTTCCAAAAAGGCATGGTGTTTCATGTGTGAATT 2240
cDNA(predicted)   ------------------------------------------------TCCAAAAAGGCATGGTGTTTCATGTGTGAATT 1128
CircularRNA       NNNNNNNNNNNNNNNNNNNNNNNNNNNNNNNNNNNNNNNNNNNNNNNNTCCAAAAAGGCATGGTGTTTCATGTGTGAATT 2044

                                      2.260                         2.280                         2.300                         2.320
Genomic           TGAAAGACTTATTGTGGAGGGCAAGCAGGGCAAGACTGCTTTATCACCAACTGGGATACTGTCCCATTTGCACGAGATTG 2320
cDNA(predicted)   TGAAAGACTTATTGTGGAGGGCAAGCAGGGCAAGACTGCTTTATCACCAACTGGGATACTGTCCCATTTGCACGAGATTG 1208
CircularRNA       TGAAAGACTTATTGTGGAGGGCAAGCAGGGCAAGACTGCTTTATCACCAACTGGGATACTGTCCCATTTGCACGAGATTG 2124

                                      2.340                         2.360                         2.380                         2.400
Genomic           GAAGTAGCTTTGGTCCTGGTAAACAAGAAGATGCTCATGAGTTCCTTCGGTAGGAAACCGATCATTGCATTTTTTCATCT 2400
cDNA(predicted)   GAAGTAGCTTTGGTCCTGGTAAA--------------------------------------------------------- 1231
CircularRNA       GAAGTAGCTTTGGTCCTGGTAAACAAGAAGATGCTCATGAGTTCCTTCG------------------------------- 2173

                                      2.420                         2.440                         2.460                         2.480
Genomic           ATGTTAGTTATTTACAGTTGGCACTTTTAGAAACTTAAGTTGGAGTTCACAGAGAAGCCATGAATATTTATGTTTCAATT 2480
cDNA(predicted)   -------------------------------------------------------------------------------- 1231
CircularRNA       -------------------------------------------------------------------------------- 2173

                                      2.500                         2.520                         2.540                         2.560
Genomic           TTTTCAATATAAGGTTTCCACAATAATATGGCTCTTAAGTTGAATCTGCTGATGCTATACTTCAATTGACCAATTATGAC 2560
cDNA(predicted)   -------------------------------------------------------------------------------- 1231
CircularRNA       -------------------------------------------------------------------------------- 2173

                                      2.580                         2.600                         2.620                         2.640
Genomic           GTGTTGGTTGAAGGTAAAATTACCGACATGCTCTGCGTGACACTTTACTAATGGTGGATGAGCTATTTTGCGATCATAGA 2640
cDNA(predicted)   ------------------ATTACCGACATGCTCTGCGTGACACTTTACTAA----------------------------- 1264
CircularRNA       -------------------------------------------------------------------------------- 2173

                                      2.660                         2.680                         2.700                         2.720
Genomic           TGTTGATGAGAGGTGTACCAACGGCTCATCTCTGTGTGTCGTATGTTGTGGCGGCGCTCTACTCTAGACATCGACCAACC 2720
cDNA(predicted)   -------------------------------------------------------------------------------- 1264
CircularRNA       -------------------------------------------------------------------------------- 2173

                                      2.740                         2.760                         2.780                         2.800
Genomic           CTCCTCTTCTAGTCACTTACACGAGCTGGGCCTTATGTGCCTAAGTGGTGATGGGCCATGCCCATACGTTCAAAACTCCC 2800
cDNA(predicted)   -------------------------------------------------------------------------------- 1264
CircularRNA       -------------------------------------------------------------------------------- 2173
```

Probable KH domain-containing splicing factor_circular RNA (ID: Ch7:457516955-457517152**)**

CCCTTCAAACACCCCATGCGTCTGGAGGATGGCCAACTTTCTCCCCTGCTATGGCACAATCCC
AGAGACCTTCACAAGGTGCACCGAGTTTTATGCCAATGAGACCCCCTATTTCTGTCTCACCAG**TT**
CTATCGGCACCTTCTCAGTCAAATATGTCCACCAGCTATGGAACTCAGAATCCTCCAAGAGCA
AACTTCA

The nucleotides of junction-region are underlined. The nucleotides of junction-region which are supported by the junction-spaning sequencing reads are shown in red. Introns are not shown if the absence is supported by sequencing reads. In the absence of supporting sequencing reads, the intronic nucletides are shown as N.

Structural relationship between the circular RNA and its parental gene

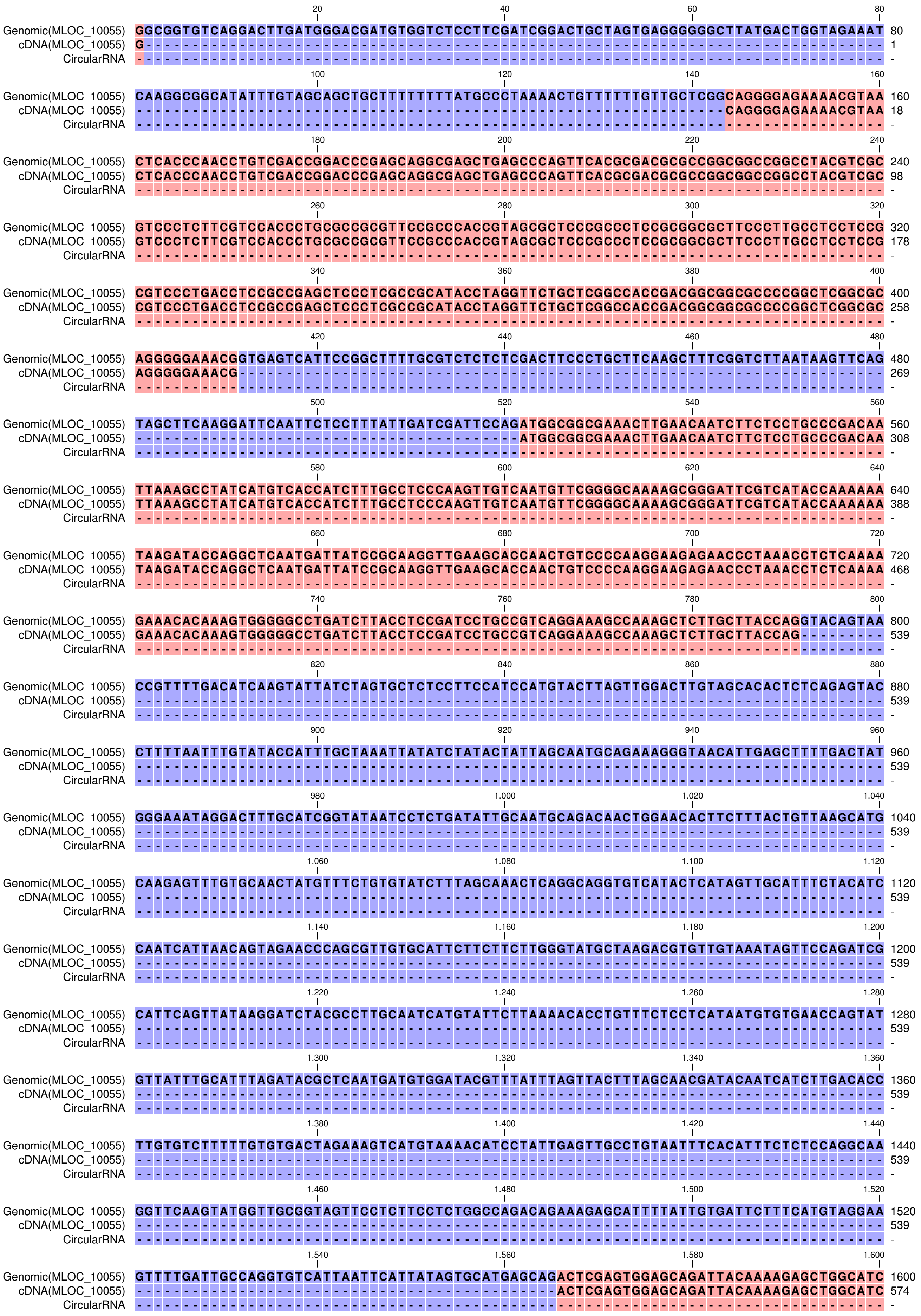
Genomic(MLOC_10055)
cDNA(MLOC_10055)
CircularRNA

```
                     1.620               1.640               1.660               1.680
Genomic(MLOC_10055)  TGGAGCTCTAGTGATAGGAGGAAACGAAGGCTCATTGTTTACGGCAAAGGGTTCAAGTTCTGATGGTGCTGAAAATCCAA 1680
cDNA(MLOC_10055)     TGGAGCTCTAGTGATAGGAGGAAACGAAGGCTCATTGTTTACGGCAAAGGGTTCAAGTTCTGATGGTGCTGAAAATCCAA 654
CircularRNA          -------------------------------------------------------------------------------- -

                     1.700               1.720               1.740               1.760
Genomic(MLOC_10055)  AAGAGAATGAGGTATTCATTATGTGACCACCATGGTGTTTCCCCCTTTCTATTGTTCTCAACATTTGGATCTAGTCCTTT 1760
cDNA(MLOC_10055)     AAGAGAATGAGG-------------------------------------------------------------------- 666
CircularRNA          -------------------------------------------------------------------------------- -

                     1.780               1.800               1.820               1.840
Genomic(MLOC_10055)  AGTATGTATTTTCGTAACTAGTAACCTCAGGTGCATGCTTTATAGAGGAAATGAGTTATACAATCAAATTATGATGTCAT 1840
cDNA(MLOC_10055)     -------------------------------------------------------------------------------- 666
CircularRNA          -------------------------------------------------------------------------------- -

                     1.860               1.880               1.900               1.920
Genomic(MLOC_10055)  TTGATATGCTTTTGAAAGCATGCTGATTGATGGTTATGGTTATGTCAAAACGATTGTAAATTCTCTTTTGATAACGTCTT 1920
cDNA(MLOC_10055)     -------------------------------------------------------------------------------- 666
CircularRNA          -------------------------------------------------------------------------------- -

                     1.940               1.960               1.980               2.000
Genomic(MLOC_10055)  CCTTTTTTCCTTTCGCGGCAGGGGAAGATTAAACTCTTGGAGCTTGAAAAACGAGAAATTATTGGTGAGAGAAATTCTTC 2000
cDNA(MLOC_10055)     ----------------------GGAAGATTAAACTCTTGGAGCTTGAAAAACGAGAAATTATTGG--------------- 709
CircularRNA          -------------------------------------------------------------------------------- -

                     2.020               2.040               2.060               2.080
Genomic(MLOC_10055)  TCTTACTGTGTGTCTTTCACTTCATATTTTTCTGAATAAAGAACTACTGCTGTGTCAGTGCATCCTGTCTCTGATGGAAG 2080
cDNA(MLOC_10055)     -------------------------------------------------------------------------------- 709
CircularRNA          -------------------------------------------------------------------------------- -

                     2.100               2.120               2.140               2.160
Genomic(MLOC_10055)  GTTGCTTCATCAAGGGGGCATACCATGTTCAATATTGCTTAAGCATATTAATTATGCAGGGACTGAATGCCTCTGTATTA 2160
cDNA(MLOC_10055)     -------------------------------------------------------------------------------- 709
CircularRNA          -------------------------------------------------------------------------------- -

                     2.180               2.200               2.220               2.240
Genomic(MLOC_10055)  GACTGTTAATCATGCTTTGTTGTATTATGTCTTAAAGAAGTGTGTTATTAACTTGACTGTGTATTCATTGTAGGAGAAAT 2240
cDNA(MLOC_10055)     --------------------------------------------------------------------------AGAAAT 715
CircularRNA          -------------------------------------------------------------------------------- -

                     2.260               2.280               2.300               2.320
Genomic(MLOC_10055)  ACTCCAACTAAATCCAGCATACAAGGCTCCTGATGATTACAAGCCATTACTTAAGGAGACAAAAATCCCTCTTCCTGTAC 2320
cDNA(MLOC_10055)     ACTCCAACTAAATCCAGCATACAAGGCTCCTGATGATTACAAGCCATTACTTAAGGAGACAAAAATCCCTCTTCCT---- 791
CircularRNA          -------------------------------------------------------------------------------- -

                     2.340               2.360               2.380               2.400
Genomic(MLOC_10055)  GTAAATGCACTGCTGTTTAATGCTTATTGATCAGTTTTCAGTTTGCACTTGTACCAGTAGTTGTTTCACTTGTTTGTAGT 2400
cDNA(MLOC_10055)     -------------------------------------------------------------------------------- 791
CircularRNA          -------------------------------------------------------------------------------- -

                     2.420               2.440               2.460               2.480
Genomic(MLOC_10055)  GGTGTCCATCTTCCATTCATATCTGAAGGTTCTCATGTCAGCTTTGTAGTGCCATCTCCTAGATTAACAATATCTTCTTA 2480
cDNA(MLOC_10055)     -------------------------------------------------------------------------------- 791
CircularRNA          -------------------------------------------------------------------------------- -

                     2.500               2.520               2.540               2.560
Genomic(MLOC_10055)  AATAAAATCTAAATCTGTGTGGATAACAAATTTGAGACTGCCTTCTCTCTGTATAAAATGTATTGCATTTACCTTGTGCT 2560
cDNA(MLOC_10055)     -------------------------------------------------------------------------------- 791
CircularRNA          -------------------------------------------------------------------------------- -

                     2.580               2.600               2.620               2.640
Genomic(MLOC_10055)  ATTGGCTATTGTAGACAGAAGCACATCCAGGACAAAACATCATTGGGGTTCTTATAGGACCTGAGAGAAACACCCAGAAG 2640
cDNA(MLOC_10055)     --------------ACAGAAGCACATCCAGGACAAAACATCATTGGGGTTCTTATAGGACCTGAGAGAAACACCCAGAAG 857
CircularRNA          -------------------------------------------------------------------------------- -

                     2.660               2.680               2.700               2.720
Genomic(MLOC_10055)  CGGCTACAGGAGGTATTAATGGGTTATTCTTGGAAACCTTCATTTTAATTGGTTGAAACATCCACGAGAATACTATTATT 2720
cDNA(MLOC_10055)     CGGCTACAGGAGG------------------------------------------------------------------- 870
CircularRNA          -------------------------------------------------------------------------------- -

                     2.740               2.760               2.780               2.800
Genomic(MLOC_10055)  TCTTAGAGTGTACTGTTACATCATTTATCATATGCCAGGAAACTGGAGCTAAAATACGAGTTTACGGGACTAAGAAAGGC 2800
cDNA(MLOC_10055)     ---------------------------------------AAACTGGAGCTAAAATACGAGTTTACGGGACTAAGAAAGGC 911
CircularRNA          -------------------------------------------------------------------------------- -

                     2.820               2.840               2.860               2.880
Genomic(MLOC_10055)  AGTGGTGAAAAGGTAAACTTGTTTCCTTGATTCCTTTTAACTTATTATATTTCTGTTCTCCATGTGAGTGAGAGCAGTAT 2880
cDNA(MLOC_10055)     AGTGGTGAAAAGG------------------------------------------------------------------- 924
CircularRNA          -------------------------------------------------------------------------------- -

                     2.900               2.920               2.940               2.960
Genomic(MLOC_10055)  GTCATACCCTCGTACTGTACAATGATGGAGAAACTATTATGACTTGTTTCAAACTTCTTAAATGTGTTGTTTGTGCTTTG 2960
cDNA(MLOC_10055)     -------------------------------------------------------------------------------- 924
CircularRNA          -------------------------------------------------------------------------------- -

                     2.980               3.000               3.020               3.040
Genomic(MLOC_10055)  GTATAGCTTTGTATTTGGTGCTGTTTCGTATACATTAAAATCTTTAATGCGAAGTATTCAGTTGGACATGCAATCCTTTG 3040
cDNA(MLOC_10055)     -------------------------------------------------------------------------------- 924
CircularRNA          -------------------------------------------------------------------------------- -

                     3.060               3.080               3.100               3.120
Genomic(MLOC_10055)  AGCGTAGATCTAGTAAACTGTATTGAGGTGAAACAAAACAAAAATGTTGGGCCTTTGTTAATTTTTTGCTCTCGTTCTTT 3120
cDNA(MLOC_10055)     -------------------------------------------------------------------------------- 924
CircularRNA          -------------------------------------------------------------------------------- -

                     3.140               3.160               3.180               3.200
Genomic(MLOC_10055)  GTTGTATCGCCTAATCGAAAATGTTAGTTAGGATCGTTTCCATGGCAGATCTTTAGGCATACTTTCTGTTTAAGTTTTAC 3200
cDNA(MLOC_10055)     -------------------------------------------------------------------------------- 924
CircularRNA          -------------------------------------------------------------------------------- -
```

```
                          3.220                3.240                3.260                3.280
Genomic(MLOC_10055)  CTGGACCGTGATAACTTTGATGCTCAAGTTCTAAGCACATGTTTTCCTTGATTATTCACTTTTTATTAGCAAATAATTCT 3280
cDNA(MLOC_10055)     -------------------------------------------------------------------------------- 924
CircularRNA          -------------------------------------------------------------------------------- -

                          3.300                3.320                3.340                3.360
Genomic(MLOC_10055)  GATAGCGATTTCAATACACCTACAACGACTGAAAACCTAAATTTGTTATTCATGCTCAGGCTGAGGTTCGCCAGCCAGAT 3360
cDNA(MLOC_10055)     ------------------------------------------------------------CTGAGGTTCGCCAGCCAGAT 944
CircularRNA          -------------------------------------------------------------------------------- -

                          3.380                3.400                3.420                3.440
Genomic(MLOC_10055)  GTACATGAAGCACAAGCTGCTTATGAGGACATATACATACATGTGTCAGCTGACTCTTATGACAAAGTTGATGCTGCAGT 3440
cDNA(MLOC_10055)     GTACATGAAGCACAAGCTGCTTATGAGGACATATACATACATGTGTCAGCTGACTCTTATGACAAAGTTGATGCTGCAGT 1024
CircularRNA          -------------------------------------------------------------------------------- -

                          3.460                3.480                3.500                3.520
Genomic(MLOC_10055)  TGCGTTGATTGAGATGCTTCTTACTCCTGTCTCAGTAAGTTCAGAAGCTTGGTCTTATATTTCTACTTGTAATATGCTTT 3520
cDNA(MLOC_10055)     TGCGTTGATTGAGATGCTTCTTACTCCTGTCTCAGTAA------------------------------------------ 1062
CircularRNA          -------------------------------------------------------------------------------- -

                          3.540                3.560                3.580                3.600
Genomic(MLOC_10055)  CAATTGACCACCAAATCATTTCAACGTTGTCAGGTAAATTCAACAGATACTTCAGAAACAGCTGTTGTTTCTTCGGCAGT 3600
cDNA(MLOC_10055)     -------------------------------------ATTCAACAGATACTTCAGAAACAGCTGTTGTTTCTTCGGCAGT 1105
CircularRNA          -------------------------------------------------------------------------------- -

                          3.620                3.640                3.660                3.680
Genomic(MLOC_10055)  TACCTCTAGTGGTGTAAACCCAGCTGATATGCAACAGGGGCAGAGCACCACTTCTCAGCCTGGCTTATTTCAGTACCAAT 3680
cDNA(MLOC_10055)     TACCTCTAGTGGTGTAAACCCAGCTGATATGCAACAGGGGCAGAGCACCACTTCTCAGCCTGGCTTATTTCAGTACCAAT 1185
CircularRNA          -------------------------------------------------------------------------------- -

                          3.700                3.720                3.740                3.760
Genomic(MLOC_10055)  CACACAGTTCTCATTGGCTTTCCATTTCTCAAACCAGTGCTCCATCAATTCCTTCCTCAGGGCCTACACCGAGCCCATTA 3760
cDNA(MLOC_10055)     CACACAGTTCTCATTGGCTTTCCATTTCTCAAACCAGTGCTCCATCAATTCCTTCCTCAGGGCCTACACCGAGCCCATTA 1265
CircularRNA          -------------------------------------------------------------------------------- -

                          3.780                3.800                3.820                3.840
Genomic(MLOC_10055)  CCCAACAATTCATTGCAGCTGCAACCTCCTGTTGGTTCTTTCAGCATGCCACCATATACAGGACAACCTCCTCACATGAA 3840
cDNA(MLOC_10055)     CCCAACAATTCATTGCAGCTGCAACCTCCTGTTGGTTCTTTCAGCATGCCACCATATACAGGACAACCTCCTCACATGAA 1345
CircularRNA          -------------------------------------------------------------------------------- -

                          3.860                3.880                3.900                3.920
Genomic(MLOC_10055)  TTTTATGCCAAGAAACACACCGCCTTTTCATGGATTTCAGCCATCAATGCCAAACAATCAACAATCTTCACAGCAGTTCC 3920
cDNA(MLOC_10055)     TTTTATGCCAAGAAACACACCGCCTTTTCATGGATTTCAGCCATCAATGCCAAACAATCAACAATCTTCACAGCAGTTCC 1425
CircularRNA          -------------------------------------------------------------------------------- -

                          3.940                3.960                3.980                4.000
Genomic(MLOC_10055)  AAGCCAACCCCTCTATTGAACCATCTTTTGGTCAACCAGCTGGAAATGCTTACAACACACAACCATTGCCATCTTCTGCG 4000
cDNA(MLOC_10055)     AAGCCAACCCCTCTATTGAACCATCTTTTGGTCAACCAGCTGGAAATGCTTACAACACACAACCATTGCCATCTTCTGCG 1505
CircularRNA          -------------------------------------------------------------------------------- -

                          4.020                4.040                4.060                4.080
Genomic(MLOC_10055)  GCCCAACTGCCTGTTAGACCCCTTCAAACACCCCATGCGTCTGGAGGATGGCCAACTTTCTCCCCTGCTATGGCACAATC 4080
cDNA(MLOC_10055)     GCCCAACTGCCTGTTAGACCCCTTCAAACACCCCATGCGTCTGGAGGATGGCCAACTTTCTCCCCTGCTATGGCACAATC 1585
CircularRNA          -------------------CCCTTCAAACACCCCATGCGTCTGGAGGATGGCCAACTTTCTCCCCTGCTATGGCACAATC 61

                          4.100                4.120                4.140                4.160
Genomic(MLOC_10055)  CCAGAGACCTTCACAAGGTGCACCGAGTTTTATGCCAATGAGACCCCCTATTTCTGTCTCACCAGTTCTATCGGCACCTT 4160
cDNA(MLOC_10055)     CCAGAGACCTTCACAAGGTGCACCGAGTTTTATGCCAATGAGACCCCCTATTTCTGTCTCACCAGTTCTATCGGCACCTT 1665
CircularRNA          CCAGAGACCTTCACAAGGTGCACCGAGTTTTATGCCAATGAGACCCCCTATTTCTGTCTCACCAGTTCTATCGGCACCTT 141

                          4.180                4.200                4.220                4.240
Genomic(MLOC_10055)  CTCAGTCAAATATGTCCACCAGCTATGGAACTCAGAATCCTCCAAGAGCAAACTTCACTCCTCCAGCAACATTACATTCC 4240
cDNA(MLOC_10055)     CTCAGTCAAATATGTCCACCAGCTATGGAACTCAGAATCCTCCAAGAGCAAACTTCACTCCTCCAGCAACATTACATTCC 1745
CircularRNA          CTCAGTCAAATATGTCCACCAGCTATGGAACTCAGAATCCTCCAAGAGCAAACTTCA----------------------- 198

                          4.260                4.280                4.300                4.320
Genomic(MLOC_10055)  AGGCCTCCTGGTGGCCCCCAGTCTTTTCCTTCAGTTTCATCTCATGGTCCCACGTTGGTGCAAGTACTATCATCTCCTGT 4320
cDNA(MLOC_10055)     AGGCCTCCTGGTGGCCCCCAGTCTTTTCCTTCAGTTTCATCTCATGGTCCCACGTTGGTGCAAGTACTATCATCTCCTGT 1825
CircularRNA          -------------------------------------------------------------------------------- 198

                          4.340                4.360                4.380                4.400
Genomic(MLOC_10055)  TGGGGCGCCACCGCCGCAAACTTACCCACCATCGATGCAAATGCGTCCTCCCATGTCAACACCTCCTCAAATGAGAGGCA 4400
cDNA(MLOC_10055)     TGGGGCGCCACCGCCGCAAACTTACCCACCATCGATGCAAATGCGTCCTCCCATGTCAACACCTCCTCAAATGAGAGGCA 1905
CircularRNA          -------------------------------------------------------------------------------- 198

                          4.420                4.440                4.460                4.480
Genomic(MLOC_10055)  CACTTTCACCTTTCCCTCAAGCTGGACCAACACCTGGCAATGCACAAGTGGCACCTTCATCGCGCCCACCTGCTGGTATG 4480
cDNA(MLOC_10055)     CACTTTCACCTTTCCCTCAAGCTGGACCAACACCTGGCAATGCACAAGTGGCACCTTCATCGCGCCCACCTGCTGGTATG 1985
CircularRNA          -------------------------------------------------------------------------------- 198

                          4.500                4.520                4.540                4.560
Genomic(MLOC_10055)  CACGGGTTGTCTTTCTCAAGTTCAGCAAACACAGGCTACAATCAGACATCAATAGCTGCTTTCAGACCTCCACGTCCAGC 4560
cDNA(MLOC_10055)     CACGGGTTGTCTTTCTCAAGTTCAGCAAACACAGGCTACAATCAGACATCAATAGCTGCTTTCAGACCTCCACGTCCAGC 2065
CircularRNA          -------------------------------------------------------------------------------- 198

                          4.580                4.600                4.620                4.640
Genomic(MLOC_10055)  CACAGGTGATTTTACCTTTAGGCCTCACGTGCCACCTTCGGCTGAACATACAGCTTCAGCGGGCCAAATGGGAGCTCAAG 4640
cDNA(MLOC_10055)     CACAGGTGATTTTACCTTTAGGCCTCACGTGCCACCTTCGGCTGAACATACAGCTTCAGCGGGCCAAATGGGAGCTCAAG 2145
CircularRNA          -------------------------------------------------------------------------------- 198

                          4.660                4.680                4.700                4.720
Genomic(MLOC_10055)  CTAACTCTCCATTTGGTTTACCCCAAGCATCGCCGTTCCGTCCTGCCAATCATGGCCCCATTTCCCCAGTTCAAGGTTTC 4720
cDNA(MLOC_10055)     CTAACTCTCCATTTGGTTTACCCCAAGCATCGCCGTTCCGTCCTGCCAATCATGGCCCCATTTCCCCAGTTCAAGGTTTC 2225
CircularRNA          -------------------------------------------------------------------------------- 198

                          4.740                4.760                4.780                4.800
Genomic(MLOC_10055)  CAAAGGCCTCCAGATGGAAGTCATATGAGTCAGGCTCGGATGCATGCTCCACCTCCACACTTCCACGGAACCTTCCGTGG 4800
cDNA(MLOC_10055)     CAAAGGCCTCCAGATGGAAGTCATATGAGTCAGGCTCGGATGCATGCTCCACCTCCACACTTCCACGGAACCTTCCGTGG 2305
CircularRNA          -------------------------------------------------------------------------------- 198
```

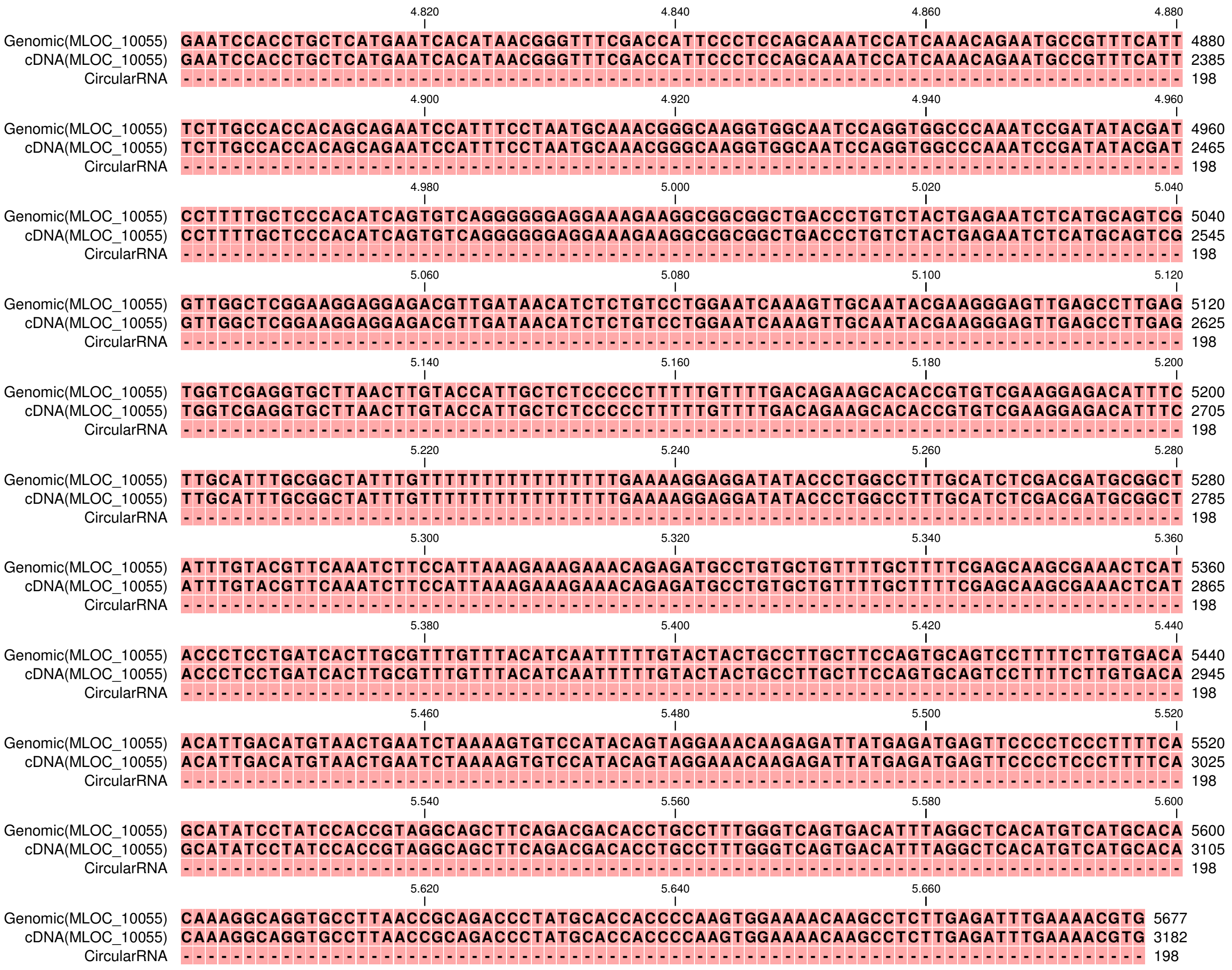
Genomic(MLOC_10055) GAATCCACCTGCTCATGAATCACATAACGGGTTTCGACCATTCCCTCCAGCAAATCCATCAAACAGAATGCCGTTTCATT 4880
cDNA(MLOC_10055) GAATCCACCTGCTCATGAATCACATAACGGGTTTCGACCATTCCCTCCAGCAAATCCATCAAACAGAATGCCGTTTCATT 2385
CircularRNA 198
Genomic(MLOC_10055) TCTTGCCACCACAGCAGAATCCATTTCCTAATGCAAACGGGCAAGGTGGCAATCCAGGTGGCCCAAATCCGATATACGAT 4960
cDNA(MLOC_10055) TCTTGCCACCACAGCAGAATCCATTTCCTAATGCAAACGGGCAAGGTGGCAATCCAGGTGGCCCAAATCCGATATACGAT 2465
CircularRNA 198
Genomic(MLOC_10055) CCTTTTGCTCCCACATCAGTGTCAGGGGGGAGGAAAGAAGGCGGCGGCTGACCCTGTCTACTGAGAATCTCATGCAGTCG 5040
cDNA(MLOC_10055) CCTTTTGCTCCCACATCAGTGTCAGGGGGGAGGAAAGAAGGCGGCGGCTGACCCTGTCTACTGAGAATCTCATGCAGTCG 2545
CircularRNA 198
Genomic(MLOC_10055) GTTGGCTCGGAAGGAGGAGACGTTGATAACATCTCTGTCCTGGAATCAAAGTTGCAATACGAAGGGAGTTGAGCCTTGAG 5120
cDNA(MLOC_10055) GTTGGCTCGGAAGGAGGAGACGTTGATAACATCTCTGTCCTGGAATCAAAGTTGCAATACGAAGGGAGTTGAGCCTTGAG 2625
CircularRNA 198
Genomic(MLOC_10055) TGGTCGAGGTGCTTAACTTGTACCATTGCTCTCCCCCTTTTTGTTTTGACAGAAGCACACCGTGTCGAAGGAGACATTTC 5200
cDNA(MLOC_10055) TGGTCGAGGTGCTTAACTTGTACCATTGCTCTCCCCCTTTTTGTTTTGACAGAAGCACACCGTGTCGAAGGAGACATTTC 2705
CircularRNA 198
Genomic(MLOC_10055) TTGCATTTGCGGCTATTTGTTTTTTTTTTTTTTTTTGAAAAGGAGGATATACCCTGGCCTTTGCATCTCGACGATGCGGCT 5280
cDNA(MLOC_10055) TTGCATTTGCGGCTATTTGTTTTTTTTTTTTTTTTTGAAAAGGAGGATATACCCTGGCCTTTGCATCTCGACGATGCGGCT 2785
CircularRNA 198
Genomic(MLOC_10055) ATTTGTACGTTCAAATCTTCCATTAAAGAAAGAAACAGAGATGCCTGTGCTGTTTTGCTTTTCGAGCAAGCGAAACTCAT 5360
cDNA(MLOC_10055) ATTTGTACGTTCAAATCTTCCATTAAAGAAAGAAACAGAGATGCCTGTGCTGTTTTGCTTTTCGAGCAAGCGAAACTCAT 2865
CircularRNA 198
Genomic(MLOC_10055) ACCCTCCTGATCACTTGCGTTTGTTTACATCAATTTTTGTACTACTGCCTTGCTTCCAGTGCAGTCCTTTTCTTGTGACA 5440
cDNA(MLOC_10055) ACCCTCCTGATCACTTGCGTTTGTTTACATCAATTTTTGTACTACTGCCTTGCTTCCAGTGCAGTCCTTTTCTTGTGACA 2945
CircularRNA 198
Genomic(MLOC_10055) ACATTGACATGTAACTGAATCTAAAAGTGTCCATACAGTAGGAAACAAGAGATTATGAGATGAGTTCCCCTCCCTTTTCA 5520
cDNA(MLOC_10055) ACATTGACATGTAACTGAATCTAAAAGTGTCCATACAGTAGGAAACAAGAGATTATGAGATGAGTTCCCCTCCCTTTTCA 3025
CircularRNA 198
Genomic(MLOC_10055) GCATATCCTATCCACCGTAGGCAGCTTCAGACGACACCTGCCTTTGGGTCAGTGACATTTAGGCTCACATGTCATGCACA 5600
cDNA(MLOC_10055) GCATATCCTATCCACCGTAGGCAGCTTCAGACGACACCTGCCTTTGGGTCAGTGACATTTAGGCTCACATGTCATGCACA 3105
CircularRNA 198
Genomic(MLOC_10055) CAAAGGCAGGTGCCTTAACCGCAGACCCTATGCACCACCCCAAGTGGAAAACAAGCCTCTTGAGATTTGAAAACGTG 5677
cDNA(MLOC_10055) CAAAGGCAGGTGCCTTAACCGCAGACCCTATGCACCACCCCAAGTGGAAAACAAGCCTCTTGAGATTTGAAAACGTG 3182
CircularRNA 198

Ribosomal protein L6_circular RNA (ID: Ch6:245896672-245897042)

GGGAGCTGTTTGAGACTGAGAAGGAGGCAACCAAGAACCTGCCTGACTTCAAAAAGGATGAC
CAGAAGGCCATTGATGCTGAGTTGATCAAGGCTATCGATGCTGTCCCTGACCTTAAGAACTATCT
TGGTGCCCGGTTCTCTCTCAGGGACGGTGACAAGCCCCATGAGATGACCTTCTAAGTTGGTAGA
AGTCCTGCGTTTTGAATGTGTTTTGCCTAGCTTGTTGCTGAAATTTGTA**CCCTGTTACTTGACTTC**
GTATGTCTGTTGAAGTGAGAAG

The nucleotides of junction-region are underlined. The nucleotides of junction-region which are supported by the junction-spaning sequencing reads are shown in red. Introns are not shown if the absence is supported by sequencing reads. In the absence of supporting sequencing reads, the intronic nucleotides are shown as N.

Structural relationship between the circular RNA and its parental gene

```
                                                 20                  40                  60                  80
Genomic(MLOC_63134) CCATCGACCTCCACGCCACCGCACACATATAAGCATGCGACCAAACCGCATCGTCCATTCTTCCCTGCCGCCGCCTCCTC 80
  cDNA1(MLOC_63134) CCATCGACCTCCACGCCACCGCACACATATAAGCATGCGACCAAACCGCATCGTCCATTCTTCCCTGCCGCCGCCTCCTC 80
  cDNA2(MLOC_63134) CCATCGACCTCCACGCCACCGCACACATATAAGCATGCGACCAAACCGCATCGTCCATTCTTCCCTGCCGCCGCCTCCTC 80
        CircularRNA -------------------------------------------------------------------------------- -

                                                100                 120                 140                 160
Genomic(MLOC_63134) CCTTCTTCAGCCGAAGCGCCCACACGCCTCGACCCAAGGGATCCCAATGGCGCCGACGTCCAAGATGGCGCTGGGCATCA 160
  cDNA1(MLOC_63134) CCTTCTTCAGCCGAAGCGCCCACACGCCTCGACCCAAGGGATCCCAATGGCGCCGACGTCCAAGATGGCGCTGGGCATCA 160
  cDNA2(MLOC_63134) CCTTCTTCAGCCGAAGCGCCCACACGCCTCGACCCAAGGGATCCCAATGGCGCCGACGTCCAAGATGGCGCTGGGCATCA 160
        CircularRNA -------------------------------------------------------------------------------- -

                                                180                 200                 220                 240
Genomic(MLOC_63134) AGCGCGCGTCGCGGTCGCACTCCTACCACCGCCGCGGGCTCTGGGCCATCAAGGCCAAGAACGGCGGAGCCTTCCCAAAG 240
  cDNA1(MLOC_63134) AGCGCGCGTCGCGGTCGCACTCCTACCACCGCCGCGGGCTCTGGGCCATCAAGGCCAAGAACGGCGGAGCCTTCCCAAAG 240
  cDNA2(MLOC_63134) AGCGCGCGTCGCGGTCGCACTCCTACCACCGCCGCGGGCTCTGGGCCATCAAGGCCAAGAACGGCGGAGCCTTCCCAAAG 240
        CircularRNA -------------------------------------------------------------------------------- -

                                                260                 280                 300                 320
Genomic(MLOC_63134) GCCGAGAAGCCAGCCGCCGTCGCCGAGCCCAAGTTCTACCCCGCCGACGACGTCAAGCCCCGCACCGCCAGCACCCGCAA 320
  cDNA1(MLOC_63134) GCCGAGAAGCCAGCCGCCGTCGCCGAGCCCAAGTTCTACCCCGCCGACGACGTCAAGCCCCGCACCGCCAGCACCCGCAA 320
  cDNA2(MLOC_63134) GCCGAGAAGCCAGCCGCCGTCGCCGAGCCCAAGTTCTACCCCGCCGACGACGTCAAGCCCCGCACCGCCAGCACCCGCAA 320
        CircularRNA -------------------------------------------------------------------------------- -

                                                340                 360                 380                 400
Genomic(MLOC_63134) GCCCAAACCTACCAAGCTCAGGTCCGTGCCGCTACCGCCTTGCAGCTCTCTCTGTCCCTCTCCGGGATTTAGGTTGAGAT 400
  cDNA1(MLOC_63134) GCCCAAACCTACCAAGCTCAGGTC-------------------------------------------------------- 344
  cDNA2(MLOC_63134) GCCCAAACCTACCAAGCTCAGGTC-------------------------------------------------------- 344
        CircularRNA -------------------------------------------------------------------------------- -

                                                420                 440                 460                 480
Genomic(MLOC_63134) CGATTTGGTTGTCGCAGGTCGACCATCACGCCCGGCACGGTGCTGATCCTGCTCGCTGGGAGGTACATGGGGAAGCGCGT 480
  cDNA1(MLOC_63134) --------------------GACCATCACGCCCGGCACGGTGCTGATCCTGCTCGCTGGGAGGTACATGGGGAAGCGCGT 404
  cDNA2(MLOC_63134) --------------------GACCATCACGCCCGGCACGGTGCTGATCCTGCTCGCTGGGAGGTACATGGGGAAGCGCGT 404
        CircularRNA -------------------------------------------------------------------------------- -

                                                500                 520                 540                 560
Genomic(MLOC_63134) GGTGTTCCTCAAGCAGCTCAAGTCTGGCCTGCTCCTCATCACTGGTAAATCTCGATGGCTCCTGCGCTATGATACTGAGA 560
  cDNA1(MLOC_63134) GGTGTTCCTCAAGCAGCTCAAGTCTGGCCTGCTCCTCATCACTGG----------------------------------- 449
  cDNA2(MLOC_63134) GGTGTTCCTCAAGCAGCTCAAGTCTGGCCTGCTCCTCATCACTGG----------------------------------- 449
        CircularRNA -------------------------------------------------------------------------------- -

                                                580                 600                 620                 640
Genomic(MLOC_63134) TGCACCCACTCCTCATAACCCAGTAGCGTGGGTCTTCGCATTGTTGGTTATTGAAATTATAGGTTTGGTTACTCGAAGTA 640
  cDNA1(MLOC_63134) -------------------------------------------------------------------------------- 449
  cDNA2(MLOC_63134) -------------------------------------------------------------------------------- 449
        CircularRNA -------------------------------------------------------------------------------- -

                                                660                 680                 700                 720
Genomic(MLOC_63134) GCTATTCGTCGATTGATGCATTGTGGATGCCTTGGCTATGTAGGTTCTTGTTTGTTTGGTCGTCTAGCCTGTTGGTTGCG 720
  cDNA1(MLOC_63134) -------------------------------------------------------------------------------- 449
  cDNA2(MLOC_63134) -------------------------------------------------------------------------------- 449
        CircularRNA -------------------------------------------------------------------------------- -

                                                740                 760                 780                 800
Genomic(MLOC_63134) TAAACCATACTTGGACTATAACTACCATTTGTGGTTTGCTGTTATCACCGACGTGCTTTTAATCGTGTTGTTAATGCCTA 800
  cDNA1(MLOC_63134) -------------------------------------------------------------------------------- 449
  cDNA2(MLOC_63134) -------------------------------------------------------------------------------- 449
        CircularRNA -------------------------------------------------------------------------------- -

                                                820                 840                 860                 880
Genomic(MLOC_63134) GAAATTTGAATATGCAACTAAACTAAAATGCCAACGAGTGCTTTATTTTGTTAACTGTTTGTATTTGTTAGCATCTGTTG 880
  cDNA1(MLOC_63134) -------------------------------------------------------------------------------- 449
  cDNA2(MLOC_63134) -------------------------------------------------------------------------------- 449
        CircularRNA -------------------------------------------------------------------------------- -

                                                900                 920                 940                 960
Genomic(MLOC_63134) GTGTCATAGTTATTGGTTCCCATAGGATGTTTGTAACTACAAAAGTTTTGAATTAAATTCGAGGCAGACATCTTTTGGAT 960
  cDNA1(MLOC_63134) -------------------------------------------------------------------------------- 449
  cDNA2(MLOC_63134) -------------------------------------------------------------------------------- 449
        CircularRNA -------------------------------------------------------------------------------- -

                                                980               1.000               1.020               1.040
Genomic(MLOC_63134) TCTGGTTCTGGTTCTGTATTCTACCTTGAATTAGTCGTCTTTGAGAATTCCCTCCTTGCATGTTTATCTACCCCAGTTAA 1040
  cDNA1(MLOC_63134) -------------------------------------------------------------------------------- 449
  cDNA2(MLOC_63134) -------------------------------------------------------------------------------- 449
        CircularRNA -------------------------------------------------------------------------------- -

                                              1.060               1.080               1.100               1.120
Genomic(MLOC_63134) TCTGGCAAACTGGGAATTTCACATCCCTAATAATTCTAAATGCAACAATATATTCTTTTGAGGTATTTGCTTATTTCATG 1120
  cDNA1(MLOC_63134) -------------------------------------------------------------------------------- 449
  cDNA2(MLOC_63134) -------------------------------------------------------------------------------- 449
        CircularRNA -------------------------------------------------------------------------------- -

                                              1.140               1.160               1.180               1.200
Genomic(MLOC_63134) TTCTTACAAGTTATTTTGTATGCCTATTTTAACGCTGATGTTTATTTTCAGGACCTTTCAAGATCAATGGTGTCCCAGTT 1200
  cDNA1(MLOC_63134) ----------------------------------------------------ACCTTTCAAGATCAATGGTGTCCCAGTT 477
  cDNA2(MLOC_63134) ----------------------------------------------------ACCTTTCAAGATCAATGGTGTCCCAGTT 477
        CircularRNA -------------------------------------------------------------------------------- -

                                              1.220               1.240               1.260               1.280
Genomic(MLOC_63134) CGCCGGGTGAACCAGGCTTATGTCATTGCCACATCCACAAAGGTTGACATCTCTGGTGTTAAGGTGGAGAAGTTTGATGA 1280
  cDNA1(MLOC_63134) CGCCGGGTGAACCAGGCTTATGTCATTGCCACATCCACAAAGGTTGACATCTCTGGTGTTAAGGTGGAGAAGTTTGATGA 557
  cDNA2(MLOC_63134) CGCCGGGTGAACCAGGCTTATGTCATTGCCACATCCACAAAGGTTGACATCTCTGGTGTTAAGGTGGAGAAGTTTGATGA 557
        CircularRNA -------------------------------------------------------------------------------- -
```

```
                                    1.300               1.320               1.340               1.360
Genomic(MLOC_63134) CAAGTACTTTGCTAGGGACAAGAAGGCCAGGGCAAAGAAGACTGAAGGGGAGCTGTTTGAGACTGAGAAGGAGGTACATC 1360
  cDNA1(MLOC_63134) CAAGTACTTTGCTAGGGACAAGAAGGCCAGGGCAAAGAAGACTGAAGGGGAGCTGTTTGAGACTGAGAAGGAGG------ 631
  cDNA2(MLOC_63134) CAAGTACTTTGCTAGGGACAAGAAGGCCAGGGCAAAGAAGACTGAAGGGGAGCTGTTTGAGACTGAGAAGGAGG------ 631
        CircularRNA -----------------------------------------------GGGAGCTGTTTGAGACTGAGAAGGAGG------ 27

                                    1.380               1.400               1.420               1.440
Genomic(MLOC_63134) CATGAACCATTGGACGATCAGCTTATGTTCATGTCATTCTGTTCCCCCCAAACTTAGTTCTGTCTCTTGTCTGTTTAACC 1440
  cDNA1(MLOC_63134) -------------------------------------------------------------------------------- 631
  cDNA2(MLOC_63134) -------------------------------------------------------------------------------- 631
        CircularRNA -------------------------------------------------------------------------------- 27

                                    1.460               1.480               1.500               1.520
Genomic(MLOC_63134) TGCAGGCAACCAAGAACCTGCCTGACTTCAAAAAGGATGACCAGAAGGCCATTGATGCTGAGTTGATCAAGGCTATCGAT 1520
  cDNA1(MLOC_63134) ------CAACCAAGAACCTGCCTGACTTCAAAAAGGATGACCAGAAGGCCATTGATGCTGAGTTGATCAAGGCTATCGAT 705
  cDNA2(MLOC_63134) ------CAACCAAGAACCTGCCTGACTTCAAAAAGGATGACCAGAAGGCCATTGATGCTGAGTTGATCAAGGCTATCGAT 705
        CircularRNA ------CAACCAAGAACCTGCCTGACTTCAAAAAGGATGACCAGAAGGCCATTGATGCTGAGTTGATCAAGGCTATCGAT 101

                                    1.540               1.560               1.580               1.600
Genomic(MLOC_63134) GCTGTCCCTGACCTTAAGAACTATCTTGGTGCCCGGTTCTCTCTCAGGGACGGTGACAAGCCCCATGAGATGACCTTCTA 1600
  cDNA1(MLOC_63134) GCTGTCCCTGACCTTAAGAACTATCTTGGTGCCCGGTTCTCTCTCAGGGACGGTGACAAGCCCCATGAGATGACCTTCTA 785
  cDNA2(MLOC_63134) GCTGTCCCTGACCTTAAGAACTATCTTGGTGCCCGGTTCTCTCTCAGGGACGGTGACAAGCCCCATGAGATGACCTTCTA 785
        CircularRNA GCTGTCCCTGACCTTAAGAACTATCTTGGTGCCCGGTTCTCTCTCAGGGACGGTGACAAGCCCCATGAGATGACCTTCTA 181

                                    1.620               1.640               1.660               1.680
Genomic(MLOC_63134) AGTTGGTAGAAGTCCTGCGTTTTGAATGTGTTTTGCCTAGCTTGTTGCTGAAATTTGTACCCTGTTACTTGACTTCGTAT 1680
  cDNA1(MLOC_63134) AGTTGGTAGAAGTCCTGCGTTTTGAATGTGTTTTGCCTAGCTTGTTGCTGAAATTTGTACCCTGTTACTTGACTTCGTAT 865
  cDNA2(MLOC_63134) AGTTGGTAGAAGTCCTGCGTTTTGAATGTGTTTTGCCTAGCTTGTTGCTGAAATTTGTACCCTGTTACTTGACTTCGTAT 865
        CircularRNA AGTTGGTAGAAGTCCTGCGTTTTGAATGTGTTTTGCCTAGCTTGTTGCTGAAATTTGTACCCTGTTACTTGACTTCGTAT 261

                                    1.700               1.720               1.740               1.760
Genomic(MLOC_63134) GTCTGTTGAAGTGAGAAGGTTAAGAGAATCGTTATCTGCTTTTGCAGGTTTTCTTTGCGTTTGCTTAATGTAACACTTCG 1760
  cDNA1(MLOC_63134) GTCTGTTGAAGTGAGAAGGT------------------------------------------------------------ 885
  cDNA2(MLOC_63134) GTCTGTTGAAGTGAGAAGGTTAAGAGAATCGTTATCTGCTTTTGCAGGTTTTCTTTGCGTTTGCTTAATGTAACACTTCG 945
        CircularRNA GTCTGTTGAAGTGAGAAG-------------------------------------------------------------- 279

                                    1.780               1.800
Genomic(MLOC_63134) TTGTGCGTTATTTGTCCTCATTTTCCTTCCATCTGCTGGATGTTTTTGCAGGTGATCAC 1819
  cDNA1(MLOC_63134) -----------------------------------------------------GATCAC 891
  cDNA2(MLOC_63134) TTGTGCGTTATTTGTCCTCATTTTCCTTCCATCTGCTGGATGTTTTTGCAGGTGATCAC 1004
        CircularRNA ----------------------------------------------------------- 279
```

Glycyl-tRNA synthetase 2_circular RNA1 (ID: Ch6:268555508-268556210)

GTCCTATTGGAGTTTATCTACTGCAAATGTTCTGCTGCGATTTAGCTCGGAGCGCTGCCTCGAG
TGCTTAGTTTCGTCTTGCCGAGGTTTCATTTTCCTCTTCCAACCTTATTTTCTTGTGCTTGTATTAT
ATTCAGTCTTTTAATCAATTCTAACTGCGTGTGTTCCGTGCGTTGTGTTCTTTCTTACCTGTTGTT
GCTCTCTCCCTTCTCCTTTTCACTCATTTCTTTGATTTAACGACATAATATCTCATCTTTGTTCGTG
TAGATTCATTTTTGTATCTACCTATGAGGCGGTTGATGAAAATCCAAGATACTGCAGTGGCAGAT
TAATATATGTGTAACAAATTATGCTTGATAATCTGCCCAAAATTTTCATGACATTTAGTCTCGGTC
CTTGATTATTTTCTTTCGAAGGAGGGTTCCAGGAGTACTGGGCCTCCGTCGGATGCAGGAGTAC
TGGGCCTCCGTCGGATGCGCCGTCATGCAGTGCAGCAACACCAAATCACCACCCTTTTCCTCTC
TTCTACTCCTACTTGTATCCGCCAGCTATGTAGATTCACCAGCAGCAGCATCAATCAATGGCTGA
TAGATATAATGACGGTTCTTGATTTTCCCAATCAATCCATCGAGCAGGTCGGCGCCGGGACGAT
GAACCCCCTCACCTT**CATCCGGGTGCTGGGCCGCGAGCCGTGGAACGTCGC**

Glycyl-tRNA synthetase 2_circular RNA2 (ID: Ch6:268555508-268556362)

GTCCTATTGGAGTTTATCTACTGCAAATGTTCTGCTGCGATTTAGCTCGGAGCGCTGCCTCGAG
TGCTTAGTTTCGTCTTGCCGAGGTTTCATTTTCCTCTTCCAACCTTATTTTCTTGTGCTTGTATTAT
ATTCAGTCTTTTAATCAATTCTAACTGCGTGTGTTCCGTGCGTTGTGTTCTTTCTTACCTGTTGTT
GCTCTCTCCCTTCTCCTTTTCACTCATTTCTTTGATTTAACGACATAATATCTCATCTTTGTTCGTG
TAGATTCATTTTTGTATCTACCTATGAGGCGGTTGATGAAAATCCAAGATACTGCAGTGGCAGAT
TAATATATGTGTAACAAATTATGCTTGATAATCTGCCCAAAATTTTCATGACATTTAGTCTCGGTC
CTTGATTATTTTCTTTCGAAGGAGGGTTCCAGGAGTACTGGGCCTCCGTCGGATGCAGGAGTAC
TGGGCCTCCGTCGGATGCGCCGTCATGCAGTGCAGCAACACCAAATCACCACCCTTTTCCTCTC
TTCTACTCCTACTTGTATCCGCCAGCTATGTAGATTCACCAGCAGCAGCATCAATCAATGGCTGA
TAGATATAATGACGGTTCTTGATTTTCCCAATCAATCCATCGAGCAGGTCGGCGCCGGGACGAT
GAACCCCCTCACCTTCATCCGGGTGCTGGGCCGCGAGCCGTGGAACGTCGCGTGAGTGACCC
GACCCTGCATCTTATCCATTCAANNN...NNN**CCGGTGCAGCTCGCTAGGATGCTCAGGCGATA**
G

The nucleotides of junction-region are underlined. The nucleotides of junction-region which are supported by the junction-spaning sequencing reads are shown in red. Introns are not shown if the absence is supported by sequencing reads. In the absence of supporting sequencing reads, the intronic nucletides are shown as N.

Structural relationship between the circular RNA and its parental gene

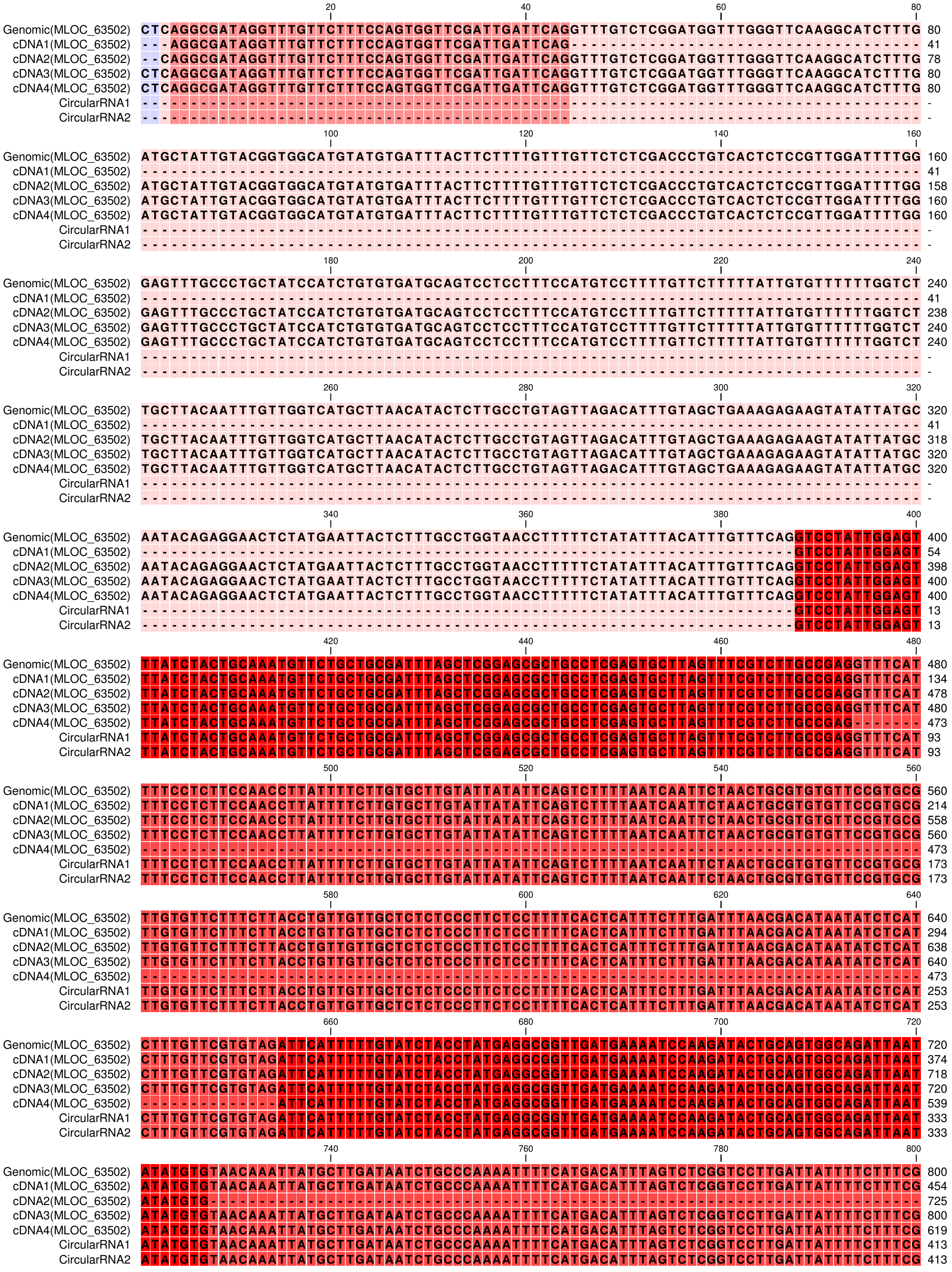

20 40 60 80
Genomic(MLOC_63502) CTCAGGCGATAGGTTTGTTCTTTCCAGTGGTTCGATTGATTCAGGTTTGTCTCGGATGGTTTGGGTTCAAGGCATCTTTG 80
cDNA1(MLOC_63502) ---AGGCGATAGGTTTGTTCTTTCCAGTGGTTCGATTGATTCAG------------------------------------ 41
cDNA2(MLOC_63502) --CAGGCGATAGGTTTGTTCTTTCCAGTGGTTCGATTGATTCAGGTTTGTCTCGGATGGTTTGGGTTCAAGGCATCTTTG 78
cDNA3(MLOC_63502) CTCAGGCGATAGGTTTGTTCTTTCCAGTGGTTCGATTGATTCAGGTTTGTCTCGGATGGTTTGGGTTCAAGGCATCTTTG 80
cDNA4(MLOC_63502) CTCAGGCGATAGGTTTGTTCTTTCCAGTGGTTCGATTGATTCAGGTTTGTCTCGGATGGTTTGGGTTCAAGGCATCTTTG 80
CircularRNA1 -- -
CircularRNA2 -- -
100 120 140 160
Genomic(MLOC_63502) ATGCTATTGTACGGTGGCATGTATGTGATTTACTTCTTTTGTTTGTTCTCTCGACCCTGTCACTCTCCGTTGGATTTTGG 160
cDNA1(MLOC_63502) -- 41
cDNA2(MLOC_63502) ATGCTATTGTACGGTGGCATGTATGTGATTTACTTCTTTTGTTTGTTCTCTCGACCCTGTCACTCTCCGTTGGATTTTGG 158
cDNA3(MLOC_63502) ATGCTATTGTACGGTGGCATGTATGTGATTTACTTCTTTTGTTTGTTCTCTCGACCCTGTCACTCTCCGTTGGATTTTGG 160
cDNA4(MLOC_63502) ATGCTATTGTACGGTGGCATGTATGTGATTTACTTCTTTTGTTTGTTCTCTCGACCCTGTCACTCTCCGTTGGATTTTGG 160
CircularRNA1 -- -
CircularRNA2 -- -
180 200 220 240
Genomic(MLOC_63502) GAGTTTGCCCTGCTATCCATCTGTGTGATGCAGTCCTCCTTTCCATGTCCTTTTGTTCTTTTTATTGTGTTTTTTGGTCT 240
cDNA1(MLOC_63502) -- 41
cDNA2(MLOC_63502) GAGTTTGCCCTGCTATCCATCTGTGTGATGCAGTCCTCCTTTCCATGTCCTTTTGTTCTTTTTATTGTGTTTTTTGGTCT 238
cDNA3(MLOC_63502) GAGTTTGCCCTGCTATCCATCTGTGTGATGCAGTCCTCCTTTCCATGTCCTTTTGTTCTTTTTATTGTGTTTTTTGGTCT 240
cDNA4(MLOC_63502) GAGTTTGCCCTGCTATCCATCTGTGTGATGCAGTCCTCCTTTCCATGTCCTTTTGTTCTTTTTATTGTGTTTTTTGGTCT 240
CircularRNA1 -- -
CircularRNA2 -- -
260 280 300 320
Genomic(MLOC_63502) TGCTTACAATTTGTTGGTCATGCTTAACATACTCTTGCCTGTAGTTAGACATTTGTAGCTGAAAGAGAAGTATATTATGC 320
cDNA1(MLOC_63502) -- 41
cDNA2(MLOC_63502) TGCTTACAATTTGTTGGTCATGCTTAACATACTCTTGCCTGTAGTTAGACATTTGTAGCTGAAAGAGAAGTATATTATGC 318
cDNA3(MLOC_63502) TGCTTACAATTTGTTGGTCATGCTTAACATACTCTTGCCTGTAGTTAGACATTTGTAGCTGAAAGAGAAGTATATTATGC 320
cDNA4(MLOC_63502) TGCTTACAATTTGTTGGTCATGCTTAACATACTCTTGCCTGTAGTTAGACATTTGTAGCTGAAAGAGAAGTATATTATGC 320
CircularRNA1 -- -
CircularRNA2 -- -
340 360 380 400
Genomic(MLOC_63502) AATACAGAGGAACTCTATGAATTACTCTTTGCCTGGTAACCTTTTTCTATATTTACATTTGTTTCAGGTCCTATTGGAGT 400
cDNA1(MLOC_63502) ---GTCCTATTGGAGT 54
cDNA2(MLOC_63502) AATACAGAGGAACTCTATGAATTACTCTTTGCCTGGTAACCTTTTTCTATATTTACATTTGTTTCAGGTCCTATTGGAGT 398
cDNA3(MLOC_63502) AATACAGAGGAACTCTATGAATTACTCTTTGCCTGGTAACCTTTTTCTATATTTACATTTGTTTCAGGTCCTATTGGAGT 400
cDNA4(MLOC_63502) AATACAGAGGAACTCTATGAATTACTCTTTGCCTGGTAACCTTTTTCTATATTTACATTTGTTTCAGGTCCTATTGGAGT 400
CircularRNA1 ---GTCCTATTGGAGT 13
CircularRNA2 ---GTCCTATTGGAGT 13
420 440 460 480
Genomic(MLOC_63502) TTATCTACTGCAAATGTTCTGCTGCGATTTAGCTCGGAGCGCTGCCTCGAGTGCTTAGTTTCGTCTTGCCGAGGTTTCAT 480
cDNA1(MLOC_63502) TTATCTACTGCAAATGTTCTGCTGCGATTTAGCTCGGAGCGCTGCCTCGAGTGCTTAGTTTCGTCTTGCCGAGGTTTCAT 134
cDNA2(MLOC_63502) TTATCTACTGCAAATGTTCTGCTGCGATTTAGCTCGGAGCGCTGCCTCGAGTGCTTAGTTTCGTCTTGCCGAGGTTTCAT 478
cDNA3(MLOC_63502) TTATCTACTGCAAATGTTCTGCTGCGATTTAGCTCGGAGCGCTGCCTCGAGTGCTTAGTTTCGTCTTGCCGAGGTTTCAT 480
cDNA4(MLOC_63502) TTATCTACTGCAAATGTTCTGCTGCGATTTAGCTCGGAGCGCTGCCTCGAGTGCTTAGTTTCGTCTTGCCGAG------- 473
CircularRNA1 TTATCTACTGCAAATGTTCTGCTGCGATTTAGCTCGGAGCGCTGCCTCGAGTGCTTAGTTTCGTCTTGCCGAGGTTTCAT 93
CircularRNA2 TTATCTACTGCAAATGTTCTGCTGCGATTTAGCTCGGAGCGCTGCCTCGAGTGCTTAGTTTCGTCTTGCCGAGGTTTCAT 93
500 520 540 560
Genomic(MLOC_63502) TTTCCTCTTCCAACCTTATTTTCTTGTGCTTGTATTATATTCAGTCTTTTAATCAATTCTAACTGCGTGTGTTCCGTGCG 560
cDNA1(MLOC_63502) TTTCCTCTTCCAACCTTATTTTCTTGTGCTTGTATTATATTCAGTCTTTTAATCAATTCTAACTGCGTGTGTTCCGTGCG 214
cDNA2(MLOC_63502) TTTCCTCTTCCAACCTTATTTTCTTGTGCTTGTATTATATTCAGTCTTTTAATCAATTCTAACTGCGTGTGTTCCGTGCG 558
cDNA3(MLOC_63502) TTTCCTCTTCCAACCTTATTTTCTTGTGCTTGTATTATATTCAGTCTTTTAATCAATTCTAACTGCGTGTGTTCCGTGCG 560
cDNA4(MLOC_63502) -- 473
CircularRNA1 TTTCCTCTTCCAACCTTATTTTCTTGTGCTTGTATTATATTCAGTCTTTTAATCAATTCTAACTGCGTGTGTTCCGTGCG 173
CircularRNA2 TTTCCTCTTCCAACCTTATTTTCTTGTGCTTGTATTATATTCAGTCTTTTAATCAATTCTAACTGCGTGTGTTCCGTGCG 173
580 600 620 640
Genomic(MLOC_63502) TTGTGTTCTTTCTTACCTGTTGTTGCTCTCTCCCTTCTCCTTTTCACTCATTTCTTTGATTTAACGACATAATATCTCAT 640
cDNA1(MLOC_63502) TTGTGTTCTTTCTTACCTGTTGTTGCTCTCTCCCTTCTCCTTTTCACTCATTTCTTTGATTTAACGACATAATATCTCAT 294
cDNA2(MLOC_63502) TTGTGTTCTTTCTTACCTGTTGTTGCTCTCTCCCTTCTCCTTTTCACTCATTTCTTTGATTTAACGACATAATATCTCAT 638
cDNA3(MLOC_63502) TTGTGTTCTTTCTTACCTGTTGTTGCTCTCTCCCTTCTCCTTTTCACTCATTTCTTTGATTTAACGACATAATATCTCAT 640
cDNA4(MLOC_63502) -- 473
CircularRNA1 TTGTGTTCTTTCTTACCTGTTGTTGCTCTCTCCCTTCTCCTTTTCACTCATTTCTTTGATTTAACGACATAATATCTCAT 253
CircularRNA2 TTGTGTTCTTTCTTACCTGTTGTTGCTCTCTCCCTTCTCCTTTTCACTCATTTCTTTGATTTAACGACATAATATCTCAT 253
660 680 700 720
Genomic(MLOC_63502) CTTTGTTCGTGTAGATTCATTTTTGTATCTACCTATGAGGCGGTTGATGAAAATCCAAGATACTGCAGTGGCAGATTAAT 720
cDNA1(MLOC_63502) CTTTGTTCGTGTAGATTCATTTTTGTATCTACCTATGAGGCGGTTGATGAAAATCCAAGATACTGCAGTGGCAGATTAAT 374
cDNA2(MLOC_63502) CTTTGTTCGTGTAGATTCATTTTTGTATCTACCTATGAGGCGGTTGATGAAAATCCAAGATACTGCAGTGGCAGATTAAT 718
cDNA3(MLOC_63502) CTTTGTTCGTGTAGATTCATTTTTGTATCTACCTATGAGGCGGTTGATGAAAATCCAAGATACTGCAGTGGCAGATTAAT 720
cDNA4(MLOC_63502) --------------ATTCATTTTTGTATCTACCTATGAGGCGGTTGATGAAAATCCAAGATACTGCAGTGGCAGATTAAT 539
CircularRNA1 CTTTGTTCGTGTAGATTCATTTTTGTATCTACCTATGAGGCGGTTGATGAAAATCCAAGATACTGCAGTGGCAGATTAAT 333
CircularRNA2 CTTTGTTCGTGTAGATTCATTTTTGTATCTACCTATGAGGCGGTTGATGAAAATCCAAGATACTGCAGTGGCAGATTAAT 333
740 760 780 800
Genomic(MLOC_63502) ATATGTGTAACAAATTATGCTTGATAATCTGCCCAAAATTTTCATGACATTTAGTCTCGGTCCTTGATTATTTTCTTTCG 800
cDNA1(MLOC_63502) ATATGTGTAACAAATTATGCTTGATAATCTGCCCAAAATTTTCATGACATTTAGTCTCGGTCCTTGATTATTTTCTTTCG 454
cDNA2(MLOC_63502) ATATGTG--- 725
cDNA3(MLOC_63502) ATATGTGTAACAAATTATGCTTGATAATCTGCCCAAAATTTTCATGACATTTAGTCTCGGTCCTTGATTATTTTCTTTCG 800
cDNA4(MLOC_63502) ATATGTGTAACAAATTATGCTTGATAATCTGCCCAAAATTTTCATGACATTTAGTCTCGGTCCTTGATTATTTTCTTTCG 619
CircularRNA1 ATATGTGTAACAAATTATGCTTGATAATCTGCCCAAAATTTTCATGACATTTAGTCTCGGTCCTTGATTATTTTCTTTCG 413
CircularRNA2 ATATGTGTAACAAATTATGCTTGATAATCTGCCCAAAATTTTCATGACATTTAGTCTCGGTCCTTGATTATTTTCTTTCG 413

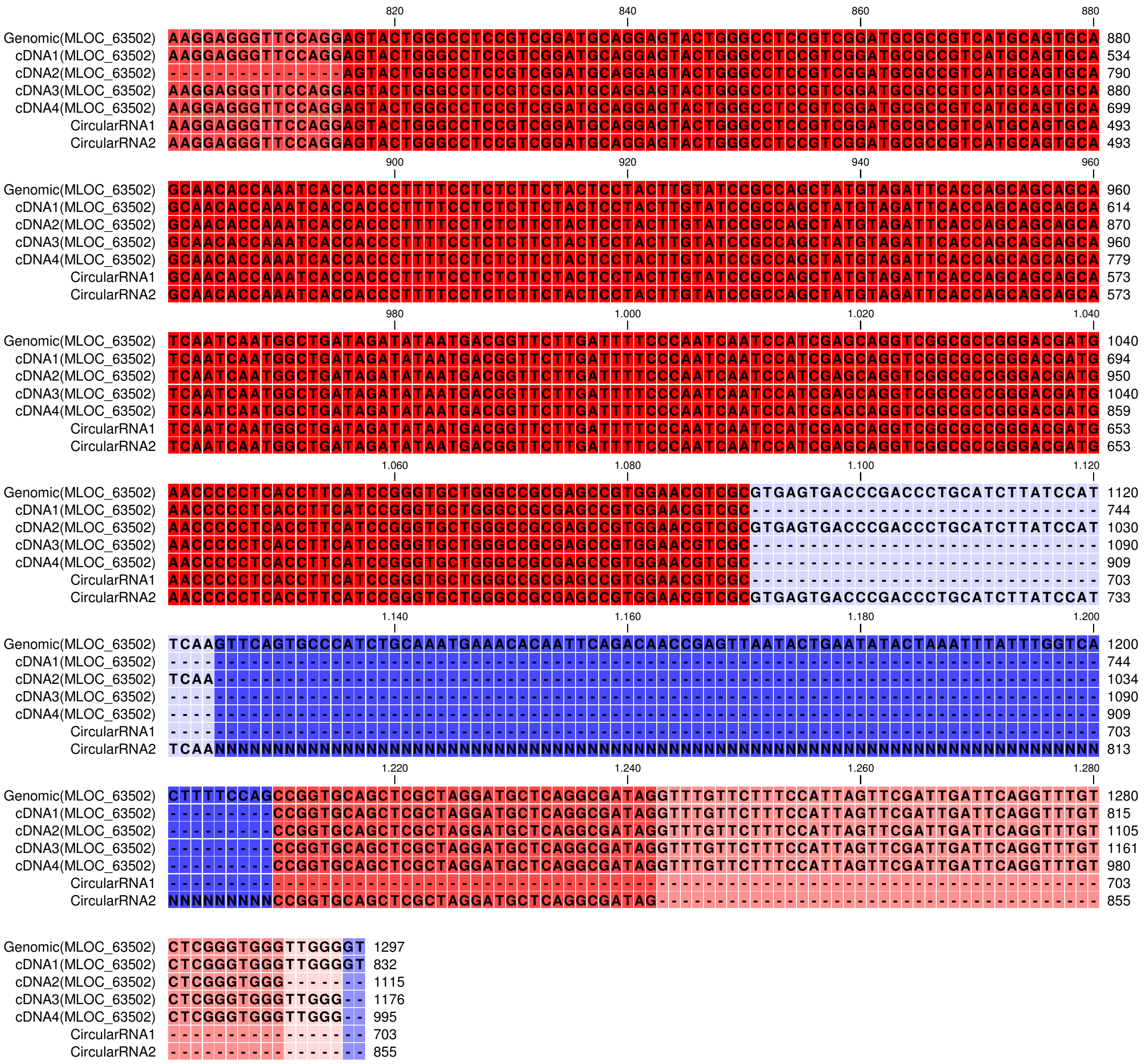

820 840 860 880
Genomic(MLOC_63502) AAGGAGGGTTCCAGGAGTACTGGGCCTCCGTCGGATGCAGGAGTACTGGGCCTCCGTCGGATGCGCCGTCATGCAGTGCA 880
cDNA1(MLOC_63502) AAGGAGGGTTCCAGGAGTACTGGGCCTCCGTCGGATGCAGGAGTACTGGGCCTCCGTCGGATGCGCCGTCATGCAGTGCA 534
cDNA2(MLOC_63502) ---------------AGTACTGGGCCTCCGTCGGATGCAGGAGTACTGGGCCTCCGTCGGATGCGCCGTCATGCAGTGCA 790
cDNA3(MLOC_63502) AAGGAGGGTTCCAGGAGTACTGGGCCTCCGTCGGATGCAGGAGTACTGGGCCTCCGTCGGATGCGCCGTCATGCAGTGCA 880
cDNA4(MLOC_63502) AAGGAGGGTTCCAGGAGTACTGGGCCTCCGTCGGATGCAGGAGTACTGGGCCTCCGTCGGATGCGCCGTCATGCAGTGCA 699
CircularRNA1 AAGGAGGGTTCCAGGAGTACTGGGCCTCCGTCGGATGCAGGAGTACTGGGCCTCCGTCGGATGCGCCGTCATGCAGTGCA 493
CircularRNA2 AAGGAGGGTTCCAGGAGTACTGGGCCTCCGTCGGATGCAGGAGTACTGGGCCTCCGTCGGATGCGCCGTCATGCAGTGCA 493
900 920 940 960
Genomic(MLOC_63502) GCAACACCAAATCACCACCCTTTTCCTCTCTTCTACTCCTACTTGTATCCGCCAGCTATGTAGATTCACCAGCAGCAGCA 960
cDNA1(MLOC_63502) GCAACACCAAATCACCACCCTTTTCCTCTCTTCTACTCCTACTTGTATCCGCCAGCTATGTAGATTCACCAGCAGCAGCA 614
cDNA2(MLOC_63502) GCAACACCAAATCACCACCCTTTTCCTCTCTTCTACTCCTACTTGTATCCGCCAGCTATGTAGATTCACCAGCAGCAGCA 870
cDNA3(MLOC_63502) GCAACACCAAATCACCACCCTTTTCCTCTCTTCTACTCCTACTTGTATCCGCCAGCTATGTAGATTCACCAGCAGCAGCA 960
cDNA4(MLOC_63502) GCAACACCAAATCACCACCCTTTTCCTCTCTTCTACTCCTACTTGTATCCGCCAGCTATGTAGATTCACCAGCAGCAGCA 779
CircularRNA1 GCAACACCAAATCACCACCCTTTTCCTCTCTTCTACTCCTACTTGTATCCGCCAGCTATGTAGATTCACCAGCAGCAGCA 573
CircularRNA2 GCAACACCAAATCACCACCCTTTTCCTCTCTTCTACTCCTACTTGTATCCGCCAGCTATGTAGATTCACCAGCAGCAGCA 573
980 1.000 1.020 1.040
Genomic(MLOC_63502) TCAATCAATGGCTGATAGATATAATGACGGTTCTTGATTTTCCCAATCAATCCATCGAGCAGGTCGGCGCCGGGACGATG 1040
cDNA1(MLOC_63502) TCAATCAATGGCTGATAGATATAATGACGGTTCTTGATTTTCCCAATCAATCCATCGAGCAGGTCGGCGCCGGGACGATG 694
cDNA2(MLOC_63502) TCAATCAATGGCTGATAGATATAATGACGGTTCTTGATTTTCCCAATCAATCCATCGAGCAGGTCGGCGCCGGGACGATG 950
cDNA3(MLOC_63502) TCAATCAATGGCTGATAGATATAATGACGGTTCTTGATTTTCCCAATCAATCCATCGAGCAGGTCGGCGCCGGGACGATG 1040
cDNA4(MLOC_63502) TCAATCAATGGCTGATAGATATAATGACGGTTCTTGATTTTCCCAATCAATCCATCGAGCAGGTCGGCGCCGGGACGATG 859
CircularRNA1 TCAATCAATGGCTGATAGATATAATGACGGTTCTTGATTTTCCCAATCAATCCATCGAGCAGGTCGGCGCCGGGACGATG 653
CircularRNA2 TCAATCAATGGCTGATAGATATAATGACGGTTCTTGATTTTCCCAATCAATCCATCGAGCAGGTCGGCGCCGGGACGATG 653
1.060 1.080 1.100 1.120
Genomic(MLOC_63502) AACCCCCTCACCTTCATCCGGGTGCTGGGCCGCGAGCCGTGGAACGTCGCGTGAGTGACCCGACCCTGCATCTTATCCAT 1120
cDNA1(MLOC_63502) AACCCCCTCACCTTCATCCGGGTGCTGGGCCGCGAGCCGTGGAACGTCGC------------------------------ 744
cDNA2(MLOC_63502) AACCCCCTCACCTTCATCCGGGTGCTGGGCCGCGAGCCGTGGAACGTCGCGTGAGTGACCCGACCCTGCATCTTATCCAT 1030
cDNA3(MLOC_63502) AACCCCCTCACCTTCATCCGGGTGCTGGGCCGCGAGCCGTGGAACGTCGC------------------------------ 1090
cDNA4(MLOC_63502) AACCCCCTCACCTTCATCCGGGTGCTGGGCCGCGAGCCGTGGAACGTCGC------------------------------ 909
CircularRNA1 AACCCCCTCACCTTCATCCGGGTGCTGGGCCGCGAGCCGTGGAACGTCGC------------------------------ 703
CircularRNA2 AACCCCCTCACCTTCATCCGGGTGCTGGGCCGCGAGCCGTGGAACGTCGCGTGAGTGACCCGACCCTGCATCTTATCCAT 733
1.140 1.160 1.180 1.200
Genomic(MLOC_63502) TCAAGTTCAGTGCCCATCTGCAAATGAAACACAATTCAGACAACCGAGTTAATACTGAATATACTAAATTTATTTGGTCA 1200
cDNA1(MLOC_63502) -- 744
cDNA2(MLOC_63502) TCAA-- 1034
cDNA3(MLOC_63502) -- 1090
cDNA4(MLOC_63502) -- 909
CircularRNA1 -- 703
CircularRNA2 TCAANNNNNNNNNNNNNNNNNNNNNNNNNNNNNNNNNNNNNNNNNNNNNNNNNNNNNNNNNNNNNNNNNNNNNNNNNNNN 813
1.220 1.240 1.260 1.280
Genomic(MLOC_63502) CTTTTCCAGCCGGTGCAGCTCGCTAGGATGCTCAGGCGATAGGTTTGTTCTTTCCATTAGTTCGATTGATTCAGGTTTGT 1280
cDNA1(MLOC_63502) ---------CCGGTGCAGCTCGCTAGGATGCTCAGGCGATAGGTTTGTTCTTTCCATTAGTTCGATTGATTCAGGTTTGT 815
cDNA2(MLOC_63502) ---------CCGGTGCAGCTCGCTAGGATGCTCAGGCGATAGGTTTGTTCTTTCCATTAGTTCGATTGATTCAGGTTTGT 1105
cDNA3(MLOC_63502) ---------CCGGTGCAGCTCGCTAGGATGCTCAGGCGATAGGTTTGTTCTTTCCATTAGTTCGATTGATTCAGGTTTGT 1161
cDNA4(MLOC_63502) ---------CCGGTGCAGCTCGCTAGGATGCTCAGGCGATAGGTTTGTTCTTTCCATTAGTTCGATTGATTCAGGTTTGT 980
CircularRNA1 -- 703
CircularRNA2 NNNNNNNNNCCGGTGCAGCTCGCTAGGATGCTCAGGCGATAG-------------------------------------- 855
Genomic(MLOC_63502) CTCGGGTGGGTTGGGGT 1297
cDNA1(MLOC_63502) CTCGGGTGGGTTGGGGT 832
cDNA2(MLOC_63502) CTCGGGTGGG------- 1115
cDNA3(MLOC_63502) CTCGGGTGGGTTGGG-- 1176
cDNA4(MLOC_63502) CTCGGGTGGGTTGGG-- 995
CircularRNA1 ----------------- 703
CircularRNA2 ----------------- 855

Kinesin-related protein 11-like_circular RNA (ID: Ch1:128008822-128014221)

<u>**G**</u>**TGATAAATGATCTGCTTGATCCTACTGGACAAAATCTG**CGTGTGAGGGAGGATGCACAGGNNN…NNN
GAACTTATGTAGAAGGAATAAAAGAAGAAGTTGTACTTTCTCCAGGACATGCTCTCTCTTTCATAGCTGCTGGCGAAGNN
N…NNNAGCATCGGCATGTTGGTTCAAACAACTTCAACTTATTTAGCAGCCGAAGTCATACTATTTTCACTATGNNN…NN
NATGATTGAAAGCAGTGATCGTGGTGACGAGTACGACGGAGCCATGTACTCACAGCTTNNN…NNNAATTTGATTGATC
TAGCTGGCTCAGAGAGTTCTAAGACAGAGACTACAGGGTTAAGAAGAAGGGAAGGATCTTACATTAACAAGAGTCTTTT
AACCCTCGGAACAGTNNN…NNNCATCGGGAAGCTCAGTGAAGGGAGAGCAACACATATACCCTATCGAGATTCTAAGT
TGACTCGTCTGCTGCAGTCATCGCTGAGTGGCCATGGTCACGTCTCANNN…NNNCTAATTTGCACGATTACCCCAGCATC
GAGTAACATGGAGGAAACCCATAATACATTGAAATTTGCAAGTCGAGCAAAACGTGTTGAAATTTATGCCTCTCGTAACC
GGNNN…NNNTTAGTTGATGAGAAGTCTTTGATCAAGAAGTATCAGAGAGAAATATCATCTCTAAAGCAAGAGCTTGAT
GAGTTTAGGAGAGGAATGCTTGGGGGTGCTAGTCAGGAAGAAATAATGATTTTACGGCAACAGNNN…NNNTTGGAGG
AGGGCCAGGTAAAAATGCAATCTCGTCTTGAGGAGGAAGAGGATGCCAAAGCTGCTCTGATGAGCAGAATACAACGGTT
GACTAAGTTAATACTTGTTTCCACGAAGACCAATATTCCTGCTTTGACAGATTCGTCTCGTCTTCAGCGCCAAAATTCTGTT
AGTGAGGAAGATNNN…NNNAAGTTAAGCTCTTCGCAAGATGGTACTACAGTAGTCCAAAATGACAGTACATCAAAGGA
CACCTTATCATCTGCTTTGCCTGATCCGTTGGATGAGATCAATGGACTAAGGTCTGCTAGCGGAGATCCTTCTTCAGTAAC
TGGTTCAGGACAAGATTCAACGCAGGTNNN…NNNGGGAATCACGGAATCAGATCATTTAGATCTATTGATTGAGCAAG
TTAAGATGCTTGCTGGGGAGATTGCGTTTGGTACCAGTTCACTGAAAAGACTAATTG**AGCAATCCATAGAAGACC**
CTGAAGGGACAAAAAATCA<u>**A**</u>

The nucleotides of junction-region are underlined. The nucleotides of junction-region which are supported by the junction-spaning sequencing reads are shown in red. Introns are not shown if the absence is supported by sequencing reads. In the absence of supporting sequencing reads, the intronic nucletides are shown as N.

Structural relationship between the circular RNA and its parental gene

```
                          20                  40                  60                  80
Genomic(MLOC_10504) GTTGCCGACGGTCTGGTGCGTCAATGGATGCGCCTATGCTGATTGCTGGCCGTGACAGAGAAGTCGAAGCTGTGGGCGGC 80
   cDNA(MLOC_10504) GTTGCCGACGGTCTGGTGCGTCAATGGATGCGCCTATGCTGATTGCTGGCCGTGACAGAGAAGTCGAAGCTGTGGGCGGC 80
        CircularRNA -------------------------------------------------------------------------------- -

                         100                 120                 140                 160
Genomic(MLOC_10504) AGGGCCATTGTAGCAACGATGACTCCATGAGGGCGGTTTTTAGCGGGCGGTGCTCTCCGGATATTGCGCTGGACTAGGTC 160
   cDNA(MLOC_10504) AGGGCCATTGTAGCAACGATGACTCCATGAGGGCGGTTTTTAGCGGGCGGTGCTCTCCGGATATTGCGCTGGACTAGGTC 160
        CircularRNA -------------------------------------------------------------------------------- -

                         180                 200                 220                 240
Genomic(MLOC_10504) GGTGCGAGGCTTGCAACTTTCTTTGTGAAGCTCGTTGCCAAAATCGGAGCTGTCTTGTCCTCGATGCTACGGTCGACTGT 240
   cDNA(MLOC_10504) GGTGCGAGGCTTGCAACTTTCTTTGTGAAGCTCGTTGCCAAAATCGGAGCTGTCTTGTCCTCGATGCTACGGTCGACTGT 240
        CircularRNA -------------------------------------------------------------------------------- -

                         260                 280                 300                 320
Genomic(MLOC_10504) TTGGCGTTTGTGGGCAGGTCGTCCGCGTGGCGCGTGCCCCCATGTGGGGTTTTTAATCTCGGTTTTCCTCTAATTAACCG 320
   cDNA(MLOC_10504) TTGGCGTTTGTGGGCAGGTCGTCCGCGTGGCGCGTGCCCCCATGTGGGGTTTTTAATCTCGGTTTTCCTCTAATTAACCG 320
        CircularRNA -------------------------------------------------------------------------------- -

                         340                 360                 380                 400
Genomic(MLOC_10504) GGCAACTCTCGTCTTCTTAATCAATGAAAATGGCAAATCTTTTGCCTCGTTTAAAAAAAAAGACACTATGATTTCCGAAT 400
   cDNA(MLOC_10504) GGCAACTCTCGTCTTCTTAATCAATGAAAATGGCAAATCTTTTGCCTCGTTTAAAAAAAAAGACACTATGATTTCCGAAT 400
        CircularRNA -------------------------------------------------------------------------------- -

                         420                 440                 460                 480
Genomic(MLOC_10504) ATTGACTATTGAGCTTCTTGCCTTTCATGAAGATCAAGTTAAAAGTTACAAGTCACACTCCATCACAGATTATCTGTGTC 480
   cDNA(MLOC_10504) ATTGACTATTGAGCTTCTTGCCTTTCATGAAGATCAAGTTAAAAGTTACAAGTCACACTCCATCACAGATTATCTGTGTC 480
        CircularRNA -------------------------------------------------------------------------------- -

                         500                 520                 540                 560
Genomic(MLOC_10504) CAGAATGCACTTTCATCTAGTACTATCTGGATGGCTTAAATCTGTATTTTTATTGCAGGTGATAAATGATCTGCTTGATC 560
   cDNA(MLOC_10504) CAGAATGCACTTTCATCTAGTACTATCTGGATGGCTTAAATCTGTATTTTTATTGCAGGTGATAAATGATCTGCTTGATC 560
        CircularRNA ----------------------------------------------------------GTGATAAATGATCTGCTTGATC 22

                         580                 600                 620                 640
Genomic(MLOC_10504) CTACTGGACAAAATCTGCGTGTGAGGGAGGATGCACAGGTTTAATTCTCTAAAATTGTTACACTATGTTTATTGTCCTAC 640
   cDNA(MLOC_10504) CTACTGGACAAAATCTGCGTGTGAGGGAGGATGCACAGG----------------------------------------- 599
        CircularRNA CTACTGGACAAAATCTGCGTGTGAGGGAGGATGCACAGGNNNNNNNNNNNNNNNNNNNNNNNNNNNNNNNNNNNNNNNNN 102

                         660                 680                 700                 720
Genomic(MLOC_10504) AATTATTACACTATGCTTATTGTCCTGCTTCTGTATCTGTGCTGTAATTCTGTATTAATTGTTTTGCATCTCCTCCTAAA 720
   cDNA(MLOC_10504) -------------------------------------------------------------------------------- 599
        CircularRNA NNNNNNNNNNNNNNNNNNNNNNNNNNNNNNNNNNNNNNNNNNNNNNNNNNNNNNNNNNNNNNNNNNNNNNNNNNNNNNNN 182

                         740                 760                 780                 800
Genomic(MLOC_10504) CCTAATTTTGAGTACTTTTTTGGTAATTATTTTCTTAGATTTCTGCTTTTTTGTCACTAGTATGAATCTGCTTTTCATTT 800
   cDNA(MLOC_10504) -------------------------------------------------------------------------------- 599
        CircularRNA NNNNNNNNNNNNNNNNNNNNNNNNNNNNNNNNNNNNNNNNNNNNNNNNNNNNNNNNNNNNNNNNNNNNNNNNNNNNNNNN 262

                         820                 840                 860                 880
Genomic(MLOC_10504) TTTAAAGTTCTGGCCGTCATCCCATCCAGTTAACAAACTGTGAAGATATAGTAATATTGTTAGTTTAGCTTTCATGGGTT 880
   cDNA(MLOC_10504) -------------------------------------------------------------------------------- 599
        CircularRNA NNNNNNNNNNNNNNNNNNNNNNNNNNNNNNNNNNNNNNNNNNNNNNNNNNNNNNNNNNNNNNNNNNNNNNNNNNNNNNNN 342

                         900                 920                 940                 960
Genomic(MLOC_10504) TGATATGGATATTACTCGTACTTGCTACACATTATCTTTCTGCCCCTCCTTTTACCTTGTTTCTTCTTTAAAGTTCTGGC 960
   cDNA(MLOC_10504) -------------------------------------------------------------------------------- 599
        CircularRNA NNNNNNNNNNNNNNNNNNNNNNNNNNNNNNNNNNNNNNNNNNNNNNNNNNNNNNNNNNNNNNNNNNNNNNNNNNNNNNNN 422

                         980               1.000               1.020               1.040
Genomic(MLOC_10504) CATCATTCCATCCAGTTAACAAACTGTGAAGATATTGTAATATTGTTAGTTTAGCTTTCATGGGTTTGATATGGATATTG 1040
   cDNA(MLOC_10504) -------------------------------------------------------------------------------- 599
        CircularRNA NNNNNNNNNNNNNNNNNNNNNNNNNNNNNNNNNNNNNNNNNNNNNNNNNNNNNNNNNNNNNNNNNNNNNNNNNNNNNNNN 502

                       1.060               1.080               1.100               1.120
Genomic(MLOC_10504) CTCGTACTTGCTACACATTATCTTTCTTTAAACCTTGTTGTTTCCTCATGAAATGATGTCTAGATTAATGGTTCCAGTAG 1120
   cDNA(MLOC_10504) -------------------------------------------------------------------------------- 599
        CircularRNA NNNNNNNNNNNNNNNNNNNNNNNNNNNNNNNNNNNNNNNNNNNNNNNNNNNNNNNNNNNNNNNNNNNNNNNNNNNNNNNN 582

                       1.140               1.160               1.180               1.200
Genomic(MLOC_10504) AATGCCATCTATTTGATTTACAAGTTATCAAAATGATAAGCAGTTTTTGTGGCCTTCTTTTGGTAATGAAATTGTTACAT 1200
   cDNA(MLOC_10504) -------------------------------------------------------------------------------- 599
        CircularRNA NNNNNNNNNNNNNNNNNNNNNNNNNNNNNNNNNNNNNNNNNNNNNNNNNNNNNNNNNNNNNNNNNNNNNNNNNNNNNNNN 662

                       1.220               1.240               1.260               1.280
Genomic(MLOC_10504) GTCAAGTGAGCACGTAAACTATGTCTGGAGTGCCATTTGGGTCTGAGAGTGATTGACATGTAGTTCTTGTTTGTGTAGTC 1280
   cDNA(MLOC_10504) -------------------------------------------------------------------------------- 599
        CircularRNA NNNNNNNNNNNNNNNNNNNNNNNNNNNNNNNNNNNNNNNNNNNNNNNNNNNNNNNNNNNNNNNNNNNNNNNNNNNNNNNN 742

                       1.300               1.320               1.340               1.360
Genomic(MLOC_10504) AGCGACTCAGTGTTGTTTTTTTCTTGTTCATCTACGATGCAAGCTGTATATATGCGATAACGCTGATGTAGCTTCATGCC 1360
   cDNA(MLOC_10504) -------------------------------------------------------------------------------- 599
        CircularRNA NNNNNNNNNNNNNNNNNNNNNNNNNNNNNNNNNNNNNNNNNNNNNNNNNNNNNNNNNNNNNNNNNNNNNNNNNNNNNNNN 822

                       1.380               1.400               1.420               1.440
Genomic(MLOC_10504) TTTATCTACTGCAGTGATGTCTCTAGTTTTAAGCTTCCGCTGATCTTCTTCCTTTTGCTACAGGGAACTTATGTAGAAGG 1440
   cDNA(MLOC_10504) ----------------------------------------------------------------GAACTTATGTAGAAGG 615
        CircularRNA NNNNNNNNNNNNNNNNNNNNNNNNNNNNNNNNNNNNNNNNNNNNNNNNNNNNNNNNNNNNNNNNGAACTTATGTAGAAGG 902

                       1.460               1.480               1.500               1.520
Genomic(MLOC_10504) AATAAAAGAAGAAGTTGTACTTTCTCCAGGACATGCTCTCTCTTTCATAGCTGCTGGCGAAGGTGATTATACTATATTGC 1520
   cDNA(MLOC_10504) AATAAAAGAAGAAGTTGTACTTTCTCCAGGACATGCTCTCTCTTTCATAGCTGCTGGCGAAG------------------ 677
        CircularRNA AATAAAAGAAGAAGTTGTACTTTCTCCAGGACATGCTCTCTCTTTCATAGCTGCTGGCGAAGNNNNNNNNNNNNNNNNNN 982

                       1.540               1.560               1.580               1.600
Genomic(MLOC_10504) TTTCCCCACTACTAGAGTGTTACTCGCAAGATGGTAGAGATCACCGATTGGTATGGATATGCACCAGGAAGATGTTGTTT 1600
   cDNA(MLOC_10504) -------------------------------------------------------------------------------- 677
        CircularRNA NNNNNNNNNNNNNNNNNNNNNNNNNNNNNNNNNNNNNNNNNNNNNNNNNNNNNNNNNNNNNNNNNNNNNNNNNNNNNNNN 1062
```

```
                         1.620                1.640                1.660                1.680
Genomic(MLOC_10504) CTGTGAGACATGTGCTGTTAGCCATGCAGTATCTCTATAATCCATTAGTCTGCTTGTGAACTGTATGATTTTCTTCTCTT 1680
   cDNA(MLOC_10504) -------------------------------------------------------------------------------- 677
        CircularRNA NNNNNNNNNNNNNNNNNNNNNNNNNNNNNNNNNNNNNNNNNNNNNNNNNNNNNNNNNNNNNNNNNNNNNNNNNNNNNNNN 1142
                         1.700                1.720                1.740                1.760
Genomic(MLOC_10504) GTAGAACCAATAACTAGCCACTGATTTTCTATGATTGCTTTTGTTTAACGGATCTGCAGTTTCTCTGTGAGAAATTGTTG 1760
   cDNA(MLOC_10504) -------------------------------------------------------------------------------- 677
        CircularRNA NNNNNNNNNNNNNNNNNNNNNNNNNNNNNNNNNNNNNNNNNNNNNNNNNNNNNNNNNNNNNNNNNNNNNNNNNNNNNNNN 1222
                         1.780                1.800                1.820                1.840
Genomic(MLOC_10504) TCTCTCAATCAGTTATGCTGCATATTATCAAGACTACTGTTGTCCAATAATAATAATTGTACAGCTTTAAATTTAGACCT 1840
   cDNA(MLOC_10504) -------------------------------------------------------------------------------- 677
        CircularRNA NNNNNNNNNNNNNNNNNNNNNNNNNNNNNNNNNNNNNNNNNNNNNNNNNNNNNNNNNNNNNNNNNNNNNNNNNNNNNNNN 1302
                         1.860                1.880                1.900                1.920
Genomic(MLOC_10504) AAAGTTGCACGTAGTTTCCAAAATATGCAACTAATCGACTGACCGTCAGATAAAGAGTCAACGTCTAAGATTGCCGACTT 1920
   cDNA(MLOC_10504) -------------------------------------------------------------------------------- 677
        CircularRNA NNNNNNNNNNNNNNNNNNNNNNNNNNNNNNNNNNNNNNNNNNNNNNNNNNNNNNNNNNNNNNNNNNNNNNNNNNNNNNNN 1382
                         1.940                1.960                1.980                2.000
Genomic(MLOC_10504) AAAAACAACAATTTCTCAGTCTACTGAGAAATAAACTCTTCCATTACTACCTCCTTTACAAACTAGTAGATATTTTTCAC 2000
   cDNA(MLOC_10504) -------------------------------------------------------------------------------- 677
        CircularRNA NNNNNNNNNNNNNNNNNNNNNNNNNNNNNNNNNNNNNNNNNNNNNNNNNNNNNNNNNNNNNNNNNNNNNNNNNNNNNNNN 1462
                         2.020                2.040                2.060                2.080
Genomic(MLOC_10504) TTCTAGCCATTAGCACACGTATTCAGGAATGATTTATGACCATAGAGTATCTTTTATCAGCTTATCTAGTGACTCCTCTC 2080
   cDNA(MLOC_10504) -------------------------------------------------------------------------------- 677
        CircularRNA NNNNNNNNNNNNNNNNNNNNNNNNNNNNNNNNNNNNNNNNNNNNNNNNNNNNNNNNNNNNNNNNNNNNNNNNNNNNNNNN 1542
                         2.100                2.120                2.140                2.160
Genomic(MLOC_10504) CCATGATCTATTCTCAACGTTGGTCTCTCTTATATTCCTAATCACAATATGATTAATGCATAGATACTTTAGTTTGGGAA 2160
   cDNA(MLOC_10504) -------------------------------------------------------------------------------- 677
        CircularRNA NNNNNNNNNNNNNNNNNNNNNNNNNNNNNNNNNNNNNNNNNNNNNNNNNNNNNNNNNNNNNNNNNNNNNNNNNNNNNNNN 1622
                         2.180                2.200                2.220                2.240
Genomic(MLOC_10504) CCTAAAACATCATCTATTCGGTAAAAAAAAAGGTTTAAGCATCAGCTAATATTTTTTCTAGACGACATATGATTTTACTC 2240
   cDNA(MLOC_10504) -------------------------------------------------------------------------------- 677
        CircularRNA NNNNNNNNNNNNNNNNNNNNNNNNNNNNNNNNNNNNNNNNNNNNNNNNNNNNNNNNNNNNNNNNNNNNNNNNNNNNNNNN 1702
                         2.260                2.280                2.300                2.320
Genomic(MLOC_10504) ACAGTTGTAATTATTCAGTTTGCTAACTCAATTTTGGTGCTGATTTCTGAATTACTCTCACTTTTTGTCTTACCCGATTG 2320
   cDNA(MLOC_10504) -------------------------------------------------------------------------------- 677
        CircularRNA NNNNNNNNNNNNNNNNNNNNNNNNNNNNNNNNNNNNNNNNNNNNNNNNNNNNNNNNNNNNNNNNNNNNNNNNNNNNNNNN 1782
                         2.340                2.360                2.380                2.400
Genomic(MLOC_10504) TTTCACAGTTTTTTCTATTTTGTTGACGTTTTAATCGATGATCTATAATTTTAGTCTTATCAGCTAATATTTTTTCTAAA 2400
   cDNA(MLOC_10504) -------------------------------------------------------------------------------- 677
        CircularRNA NNNNNNNNNNNNNNNNNNNNNNNNNNNNNNNNNNNNNNNNNNNNNNNNNNNNNNNNNNNNNNNNNNNNNNNNNNNNNNNN 1862
                         2.420                2.440                2.460                2.480
Genomic(MLOC_10504) CGACATATGATTTTACTCACAGTTGTAATTATTTATTATTCAGTTTGCTAACTCAATTTTGGTGCTGATTTCTGAATTAC 2480
   cDNA(MLOC_10504) -------------------------------------------------------------------------------- 677
        CircularRNA NNNNNNNNNNNNNNNNNNNNNNNNNNNNNNNNNNNNNNNNNNNNNNNNNNNNNNNNNNNNNNNNNNNNNNNNNNNNNNNN 1942
                         2.500                2.520                2.540                2.560
Genomic(MLOC_10504) TCACACTTTTTGTCTTACCCGAGTGTTCCACAGTTTTTTCTATTTTGTTGATGTTTTAATCAATGATCTATAATTTTAGT 2560
   cDNA(MLOC_10504) -------------------------------------------------------------------------------- 677
        CircularRNA NNNNNNNNNNNNNNNNNNNNNNNNNNNNNNNNNNNNNNNNNNNNNNNNNNNNNNNNNNNNNNNNNNNNNNNNNNNNNNNN 2022
                         2.580                2.600                2.620                2.640
Genomic(MLOC_10504) CTTATCAGAGTCAAACTAATGCTACTACTTAAAATGGCAGAGCATCGGCATGTTGGTTCAAACAACTTCAACTTATTTAG 2640
   cDNA(MLOC_10504) ----------------------------------------AGCATCGGCATGTTGGTTCAAACAACTTCAACTTATTTAG 717
        CircularRNA NNNNNNNNNNNNNNNNNNNNNNNNNNNNNNNNNNNNNNNNAGCATCGGCATGTTGGTTCAAACAACTTCAACTTATTTAG 2102
                         2.660                2.680                2.700                2.720
Genomic(MLOC_10504) CAGCCGAAGTCATACTATTTTCACTATGGTGAGCTTACAAGTTTTGTCAGTATTTAAGGCATTTAATCAATTGTGAATAT 2720
   cDNA(MLOC_10504) CAGCCGAAGTCATACTATTTTCACTATG---------------------------------------------------- 745
        CircularRNA CAGCCGAAGTCATACTATTTTCACTATGNNNNNNNNNNNNNNNNNNNNNNNNNNNNNNNNNNNNNNNNNNNNNNNNNNNN 2182
                         2.740                2.760                2.780                2.800
Genomic(MLOC_10504) TGCCTTATTGTTCTTGTATGGTGGATGCTCTTGGGTTTCTTGATAATCCTGCATCATGTTTCTGAATTTCACATGTGATA 2800
   cDNA(MLOC_10504) -------------------------------------------------------------------------------- 745
        CircularRNA NNNNNNNNNNNNNNNNNNNNNNNNNNNNNNNNNNNNNNNNNNNNNNNNNNNNNNNNNNNNNNNNNNNNNNNNNNNNNNNN 2262
                         2.820                2.840                2.860                2.880
Genomic(MLOC_10504) TGTTGTATTTTTTTATTTGGGCCCATAATATTTGTGCGCTCTTCTGCAGATGATTGAAAGCAGTGATCGTGGTGACGAGT 2880
   cDNA(MLOC_10504) -------------------------------------------------ATGATTGAAAGCAGTGATCGTGGTGACGAGT 776
        CircularRNA NNNNNNNNNNNNNNNNNNNNNNNNNNNNNNNNNNNNNNNNNNNNNNNNNATGATTGAAAGCAGTGATCGTGGTGACGAGT 2342
                         2.900                2.920                2.940                2.960
Genomic(MLOC_10504) ACGACGGAGCCATGTACTCACAGCTTGTATGTTGTTTTTACCCATAAGCAAGATTTCATTTTTCAATCACTACGCTTTGA 2960
   cDNA(MLOC_10504) ACGACGGAGCCATGTACTCACAGCTT------------------------------------------------------ 802
        CircularRNA ACGACGGAGCCATGTACTCACAGCTTNNNNNNNNNNNNNNNNNNNNNNNNNNNNNNNNNNNNNNNNNNNNNNNNNNNNN 2422
                         2.980                3.000                3.020                3.040
Genomic(MLOC_10504) CGCTTTCTAGTCTTACTTTTTAGTGCTTCTTCTCTGTAGAATTTGATTGATCTAGCTGGCTCAGAGAGTTCTAAGACAGA 3040
   cDNA(MLOC_10504) ---------------------------------------AATTTGATTGATCTAGCTGGCTCAGAGAGTTCTAAGACAGA 843
        CircularRNA NNNNNNNNNNNNNNNNNNNNNNNNNNNNNNNNNNNNNNNAATTTGATTGATCTAGCTGGCTCAGAGAGTTCTAAGACAGA 2502
                         3.060                3.080                3.100                3.120
Genomic(MLOC_10504) GACTACAGGGTTAAGAAGAAGGGAAGGATCTTACATTAACAAGAGTCTTTTAACCCTCGGAACAGTAAGCTTTTGCTTCT 3120
   cDNA(MLOC_10504) GACTACAGGGTTAAGAAGAAGGGAAGGATCTTACATTAACAAGAGTCTTTTAACCCTCGGAACAGT-------------- 909
        CircularRNA GACTACAGGGTTAAGAAGAAGGGAAGGATCTTACATTAACAAGAGTCTTTTAACCCTCGGAACAGTNNNNNNNNNNNNNN 2582
                         3.140                3.160                3.180                3.200
Genomic(MLOC_10504) AATTACTTGTCACAACTTGGGGTTATTGTTTTATGTCTATCGTCGTAAATTTGAACTTGAAACACATCTCGTTAGGATCC 3200
   cDNA(MLOC_10504) -------------------------------------------------------------------------------- 909
        CircularRNA NNNNNNNNNNNNNNNNNNNNNNNNNNNNNNNNNNNNNNNNNNNNNNNNNNNNNNNNNNNNNNNNNNNNNNNNNNNNNNNN 2662
```

```
                        3.220               3.240               3.260               3.280
Genomic(MLOC_10504)  TCTTTTAGAACTTTAGTTCTACCTACTTCAGGGAGCACACCTGAACTTATATTGGAAATCATCTGGTAATTAGTTGGGGC 3280
cDNA(MLOC_10504)     -------------------------------------------------------------------------------- 909
CircularRNA          NNNNNNNNNNNNNNNNNNNNNNNNNNNNNNNNNNNNNNNNNNNNNNNNNNNNNNNNNNNNNNNNNNNNNNNNNNNNNNNN 2742

                        3.300               3.320               3.340               3.360
Genomic(MLOC_10504)  TGTTGAATATGTGGCAATGTCTTTTGTACATCTAATCAACTTTCAGGGCCACTTATGTTTCTGGTATCCTTAATGTAATC 3360
cDNA(MLOC_10504)     -------------------------------------------------------------------------------- 909
CircularRNA          NNNNNNNNNNNNNNNNNNNNNNNNNNNNNNNNNNNNNNNNNNNNNNNNNNNNNNNNNNNNNNNNNNNNNNNNNNNNNNNN 2822

                        3.380               3.400               3.420               3.440
Genomic(MLOC_10504)  ATCGATCACTTCTAAACTTCACTTTCTAATTCAAAGGTCATCGGGAAGCTCAGTGAAGGGAGAGCAACACATATACCCTA 3440
cDNA(MLOC_10504)     --------------------------------------CATCGGGAAGCTCAGTGAAGGGAGAGCAACACATATACCCTA 951
CircularRNA          NNNNNNNNNNNNNNNNNNNNNNNNNNNNNNNNNNNNNNCATCGGGAAGCTCAGTGAAGGGAGAGCAACACATATACCCTA 2902

                        3.460               3.480               3.500               3.520
Genomic(MLOC_10504)  TCGAGATTCTAAGTTGACTCGTCTGCTGCAGTCATCGCTGAGTGGCCATGGTCACGTCTCAGTAAGTATCTAGCCGATCT 3520
cDNA(MLOC_10504)     TCGAGATTCTAAGTTGACTCGTCTGCTGCAGTCATCGCTGAGTGGCCATGGTCACGTCTCA------------------- 1012
CircularRNA          TCGAGATTCTAAGTTGACTCGTCTGCTGCAGTCATCGCTGAGTGGCCATGGTCACGTCTCANNNNNNNNNNNNNNNNNNN 2982

                        3.540               3.560               3.580               3.600
Genomic(MLOC_10504)  AGACCTTCCTGCTTTGTAACTCATTACATGTGCTGGTGGAGGCTATCTGTGCAGTGTGAAGCGAAACTTGATTATCAATT 3600
cDNA(MLOC_10504)     -------------------------------------------------------------------------------- 1012
CircularRNA          NNNNNNNNNNNNNNNNNNNNNNNNNNNNNNNNNNNNNNNNNNNNNNNNNNNNNNNNNNNNNNNNNNNNNNNNNNNNNNNN 3062

                        3.620               3.640               3.660               3.680
Genomic(MLOC_10504)  TGCCAGCTAATGTACCAACTTTATGCAGCTAATTTGCACGATTACCCCAGCATCGAGTAACATGGAGGAAACCCATAATA 3680
cDNA(MLOC_10504)     ----------------------------CTAATTTGCACGATTACCCCAGCATCGAGTAACATGGAGGAAACCCATAATA 1064
CircularRNA          NNNNNNNNNNNNNNNNNNNNNNNNNNNNCTAATTTGCACGATTACCCCAGCATCGAGTAACATGGAGGAAACCCATAATA 3142

                        3.700               3.720               3.740               3.760
Genomic(MLOC_10504)  CATTGAAATTTGCAAGTCGAGCAAAACGTGTTGAAATTTATGCCTCTCGTAACCGGNNNNNNNNNNNNNNNNNNNNNNNN 3760
cDNA(MLOC_10504)     CATTGAAATTTGCAAGTCGAGCAAAACGTGTTGAAATTTATGCCTCTCGTAACCGG------------------------ 1120
CircularRNA          CATTGAAATTTGCAAGTCGAGCAAAACGTGTTGAAATTTATGCCTCTCGTAACCGGGTAAATGTTCTCAGCATGCAATTC 3222

                        3.780               3.800               3.820               3.840
Genomic(MLOC_10504)  NNNNNNNNNNNNNNNNNNNNNNNNNNNNNNNNNNNNNNNNNNNNNNNNNNNNNNNNNNNNNNTTAGTTGATGAGAAGTCT 3840
cDNA(MLOC_10504)     --------------------------------------------------------------TTAGTTGATGAGAAGTCT 1138
CircularRNA          TCGCTTCCCCTGACACCATTATTTGAATAATTGATTAACATTTCATTATTTTCTCTGTCCAGTTAGTTGATGAGAAGTCT 3302

                        3.860               3.880               3.900               3.920
Genomic(MLOC_10504)  TTGATCAAGAAGTATCAGAGAGAAATATCATCTCTAAAGCAAGAGCTTGATGAGTTTAGGAGAGGAATGCTTGGGGGTGC 3920
cDNA(MLOC_10504)     TTGATCAAGAAGTATCAGAGAGAAATATCATCTCTAAAGCAAGAGCTTGATGAGTTTAGGAGAGGAATGCTTGGGGGTGC 1218
CircularRNA          TTGATCAAGAAGTATCAGAGAGAAATATCATCTCTAAAGCAAGAGCTTGATGAGTTTAGGAGAGGAATGCTTGGGGGTGC 3382

                        3.940               3.960               3.980               4.000
Genomic(MLOC_10504)  TAGTCAGGAAGAAATAATGATTTTACGGCAACAGGTCTGCTTCATTTGCTTTTGCCAGATAGAACTAAGGAGTGAAATGG 4000
cDNA(MLOC_10504)     TAGTCAGGAAGAAATAATGATTTTACGGCAACAG---------------------------------------------- 1252
CircularRNA          TAGTCAGGAAGAAATAATGATTTTACGGCAACAGNNNNNNNNNNNNNNNNNNNNNNNNNNNNNNNNNNNNNNNNNNNNNN 3462

                        4.020               4.040               4.060               4.080
Genomic(MLOC_10504)  TTTATATCTATGCAAATGCTTGATTTTTTTTCTGTTCTATATATGAATACTTATAATGATTTCATATTGCTAAGTTGGTA 4080
cDNA(MLOC_10504)     -------------------------------------------------------------------------------- 1252
CircularRNA          NNNNNNNNNNNNNNNNNNNNNNNNNNNNNNNNNNNNNNNNNNNNNNNNNNNNNNNNNNNNNNNNNNNNNNNNNNNNNNNN 3542

                        4.100               4.120               4.140               4.160
Genomic(MLOC_10504)  AATGCTTAAAAAATGACTCTTCTTTTACCTTGTTTAGTTGGAGGAGGGCCAGGTAAAAATGCAATCTCGTCTTGAGGAGG 4160
cDNA(MLOC_10504)     -------------------------------------TTGGAGGAGGGCCAGGTAAAAATGCAATCTCGTCTTGAGGAGG 1295
CircularRNA          NNNNNNNNNNNNNNNNNNNNNNNNNNNNNNNNNNNNNTTGGAGGAGGGCCAGGTAAAAATGCAATCTCGTCTTGAGGAGG 3622

                        4.180               4.200               4.220               4.240
Genomic(MLOC_10504)  AAGAGGATGCCAAAGCTGCTCTGATGAGCAGAATACAACGGTTGACTAAGTTAATACTTGTTTCCACGAAGACCAATATT 4240
cDNA(MLOC_10504)     AAGAGGATGCCAAAGCTGCTCTGATGAGCAGAATACAACGGTTGACTAAGTTAATACTTGTTTCCACGAAGACCAATATT 1375
CircularRNA          AAGAGGATGCCAAAGCTGCTCTGATGAGCAGAATACAACGGTTGACTAAGTTAATACTTGTTTCCACGAAGACCAATATT 3702

                        4.260               4.280               4.300               4.320
Genomic(MLOC_10504)  CCTGCTTTGACAGATTCGTCTCGTCTTCAGCGCCAAAATTCTGTTAGTGAGGAAGATGTAGGTGCTCAATATTGATCTAA 4320
cDNA(MLOC_10504)     CCTGCTTTGACAGATTCGTCTCGTCTTCAGCGCCAAAATTCTGTTAGTGAGGAAGAT----------------------- 1432
CircularRNA          CCTGCTTTGACAGATTCGTCTCGTCTTCAGCGCCAAAATTCTGTTAGTGAGGAAGATNNNNNNNNNNNNNNNNNNNNNNN 3782

                        4.340               4.360               4.380               4.400
Genomic(MLOC_10504)  TTGTGCATTTGCTGCTAAAATTTAAGGTTTTATGATTCTATAAAGTTATAAGTCATGCTTTTTATACTTATAAAGTGCAC 4400
cDNA(MLOC_10504)     -------------------------------------------------------------------------------- 1432
CircularRNA          NNNNNNNNNNNNNNNNNNNNNNNNNNNNNNNNNNNNNNNNNNNNNNNNNNNNNNNNNNNNNNNNNNNNNNNNNNNNNNNN 3862

                        4.420               4.440               4.460               4.480
Genomic(MLOC_10504)  TAAATCTATGTATTTCAGAAGTTAAGCTCTTCGCAAGATGGTACTACAGTAGTCCAAAATGACAGTACATCAAAGGACAC 4480
cDNA(MLOC_10504)     ------------------AAGTTAAGCTCTTCGCAAGATGGTACTACAGTAGTCCAAAATGACAGTACATCAAAGGACAC 1494
CircularRNA          NNNNNNNNNNNNNNNNNNAAGTTAAGCTCTTCGCAAGATGGTACTACAGTAGTCCAAAATGACAGTACATCAAAGGACAC 3942

                        4.500               4.520               4.540               4.560
Genomic(MLOC_10504)  CTTATCATCTGCTTTGCCTGATCCGTTGGATGAGATCAATGGACTAAGGTCTGCTAGCGGAGATCCTTCTTCAGTAACTG 4560
cDNA(MLOC_10504)     CTTATCATCTGCTTTGCCTGATCCGTTGGATGAGATCAATGGACTAAGGTCTGCTAGCGGAGATCCTTCTTCAGTAACTG 1574
CircularRNA          CTTATCATCTGCTTTGCCTGATCCGTTGGATGAGATCAATGGACTAAGGTCTGCTAGCGGAGATCCTTCTTCAGTAACTG 4022

                        4.580               4.600               4.620               4.640
Genomic(MLOC_10504)  GTTCAGGACAAGATTCAACGCAGGTATTTGGATTTGCATTCTACATCTTTTAAGTGACCGGTGCCTTAATCCTAAGGTTT 4640
cDNA(MLOC_10504)     GTTCAGGACAAGATTCAACGCAGGT------------------------------------------------------- 1599
CircularRNA          GTTCAGGACAAGATTCAACGCAGGTNNNNNNNNNNNNNNNNNNNNNNNNNNNNNNNNNNNNNNNNNNNNNNNNNNNNNNN 4102

                        4.660               4.680               4.700               4.720
Genomic(MLOC_10504)  ATTGCTATCATCTCGCCCTTCTTGTTTTTTTTTTGTCAGTAGCACCAGTATGCATGCACAATTTCTTGTGGAAAGTATGT 4720
cDNA(MLOC_10504)     -------------------------------------------------------------------------------- 1599
CircularRNA          NNNNNNNNNNNNNNNNNNNNNNNNNNNNNNNNNNNNNNNNNNNNNNNNNNNNNNNNNNNNNNNNNNNNNNNNNNNNNNNN 4182

                        4.740               4.760               4.780               4.800
Genomic(MLOC_10504)  CTTATCACTTGTTCGGCTCAAAACAACATTTCGGAAACTACAATATCAGAATTCCAAAACCTATTTGGCCAGATTGCTGG 4800
cDNA(MLOC_10504)     -------------------------------------------------------------------------------- 1599
CircularRNA          NNNNNNNNNNNNNNNNNNNNNNNNNNNNNNNNNNNNNNNNNNNNNNNNNNNNNNNNNNNNNNNNNNNNNNNNNNNNNNNN 4262
```

```
                                4.820               4.840               4.860               4.880
Genomic(MLOC_10504)  TTTTCCCTAAAAACATTGCGTGCAATTAGTGTGGGTAACACTCACAGCTTACTTCCCATTGCGGTCATGCATTGTTTCTT 4880
cDNA(MLOC_10504)     -------------------------------------------------------------------------------- 1599
CircularRNA          NNNNNNNNNNNNNNNNNNNNNNNNNNNNNNNNNNNNNNNNNNNNNNNNNNNNNNNNNNNNNNNNNNNNNNNNNNNNNNNN 4342

                                4.900               4.920               4.940               4.960
Genomic(MLOC_10504)  TTCAGTCATGGCTGGGATTTTTTTCTTTTTGTGAAAAGTCATGGCTGGGAGTTGTGTTCTCTAGTTGACTGTATGCTCGC 4960
cDNA(MLOC_10504)     -------------------------------------------------------------------------------- 1599
CircularRNA          NNNNNNNNNNNNNNNNNNNNNNNNNNNNNNNNNNNNNNNNNNNNNNNNNNNNNNNNNNNNNNNNNNNNNNNNNNNNNNNN 4422

                                4.980               5.000               5.020               5.040
Genomic(MLOC_10504)  CGCCACAAATATTTTTGTCCTGAGCTCCATTCATTCTTTTCCCAGAATAGAAGGCATCTGCGATACAATACTGCGATATG 5040
cDNA(MLOC_10504)     -------------------------------------------------------------------------------- 1599
CircularRNA          NNNNNNNNNNNNNNNNNNNNNNNNNNNNNNNNNNNNNNNNNNNNNNNNNNNNNNNNNNNNNNNNNNNNNNNNNNNNNNNN 4502

                                5.060               5.080               5.100               5.120
Genomic(MLOC_10504)  CTCATATTATATTTCCGTAAAGCAAGACTAGATCGATTAATCTGTATTTTTGCTAGATAAAAGTAGAAGATACATGTTTA 5120
cDNA(MLOC_10504)     -------------------------------------------------------------------------------- 1599
CircularRNA          NNNNNNNNNNNNNNNNNNNNNNNNNNNNNNNNNNNNNNNNNNNNNNNNNNNNNNNNNNNNNNNNNNNNNNNNNNNNNNNN 4582

                                5.140               5.160               5.180               5.200
Genomic(MLOC_10504)  TTTGTATTTTTGAAAATTAAGTATTTAGTGAGCCTATAAGTGAGCAAGGAGGTGATATTACCATATGAAAATGGAAAACC 5200
cDNA(MLOC_10504)     -------------------------------------------------------------------------------- 1599
CircularRNA          NNNNNNNNNNNNNNNNNNNNNNNNNNNNNNNNNNNNNNNNNNNNNNNNNNNNNNNNNNNNNNNNNNNNNNNNNNNNNNNN 4662

                                5.220               5.240               5.260               5.280
Genomic(MLOC_10504)  TAAACTAAGATCGAGAGCTTCTGTTTGCCATGCCCTAAAACCAAATGCAAACACGAAAATCAGATCTGGGTACTGGACAG 5280
cDNA(MLOC_10504)     -------------------------------------------------------------------------------- 1599
CircularRNA          NNNNNNNNNNNNNNNNNNNNNNNNNNNNNNNNNNNNNNNNNNNNNNNNNNNNNNNNNNNNNNNNNNNNNNNNNNNNNNNN 4742

                                5.300               5.320               5.340               5.360
Genomic(MLOC_10504)  TGGACTCTAGAATGGAATAGGTTCTAGAGAGAGGAGGCGGCAGCAGCTAGAGGGATGGAGGAGGTGGCTGTGGCAGGAAC 5360
cDNA(MLOC_10504)     -------------------------------------------------------------------------------- 1599
CircularRNA          NNNNNNNNNNNNNNNNNNNNNNNNNNNNNNNNNNNNNNNNNNNNNNNNNNNNNNNNNNNNNNNNNNNNNNNNNNNNNNNN 4822

                                5.380               5.400               5.420               5.440
Genomic(MLOC_10504)  AACAGTTTAGTGCGGAGGAAGCGACATTGTGTGGTGGAAGCAGCAGCCTTTGGTGGAGGAGGGAAAGGAAGAGGCAGCGA 5440
cDNA(MLOC_10504)     -------------------------------------------------------------------------------- 1599
CircularRNA          NNNNNNNNNNNNNNNNNNNNNNNNNNNNNNNNNNNNNNNNNNNNNNNNNNNNNNNNNNNNNNNNNNNNNNNNNNNNNNNN 4902

                                5.460               5.480               5.500               5.520
Genomic(MLOC_10504)  CAGCCGATAGGAGTGAGGAGGGGAAGGAAGAGGCAGTGACAGCCGATAGAAATGAGGAGCGGAAGGAGGGGGAAATAGAA 5520
cDNA(MLOC_10504)     -------------------------------------------------------------------------------- 1599
CircularRNA          NNNNNNNNNNNNNNNNNNNNNNNNNNNNNNNNNNNNNNNNNNNNNNNNNNNNNNNNNNNNNNNNNNNNNNNNNNNNNNNN 4982

                                5.540               5.560               5.580               5.600
Genomic(MLOC_10504)  CGGGAGGGCTGCCAGAGGAGCCAAGGACTGAGTGGAGACTTGTCGTGGATCTTTATTTTGCACTCTCTGATGGAACATAT 5600
cDNA(MLOC_10504)     -------------------------------------------------------------------------------- 1599
CircularRNA          NNNNNNNNNNNNNNNNNNNNNNNNNNNNNNNNNNNNNNNNNNNNNNNNNNNNNNNNNNNNNNNNNNNNNNNNNNNNNNNN 5062

                                5.620               5.640               5.660               5.680
Genomic(MLOC_10504)  CCAACAGTCTATATTAACAGTTCATCGTGGTTTCTTCCCTAACAAAAAGTTGTTCCTGGCTTCAAGAGCTCTGATGCTAG 5680
cDNA(MLOC_10504)     -------------------------------------------------------------------------------- 1599
CircularRNA          NNNNNNNNNNNNNNNNNNNNNNNNNNNNNNNNNNNNNNNNNNNNNNNNNNNNNNNNNNNNNNNNNNNNNNNNNNNNNNNN 5142

                                5.700               5.720               5.740               5.760
Genomic(MLOC_10504)  ACATGCAATAGACAGTATAATGTTTATTTTATTATGTATTTTTTTAGAATCCATTTGATACCGGATTTAGCTGAATTTAT 5760
cDNA(MLOC_10504)     -------------------------------------------------------------------------------- 1599
CircularRNA          NNNNNNNNNNNNNNNNNNNNNNNNNNNNNNNNNNNNNNNNNNNNNNNNNNNNNNNNNNNNNNNNNNNNNNNNNNNNNNNN 5222

                                5.780               5.800               5.820               5.840
Genomic(MLOC_10504)  GATTATTGATTATGGCTTAATGTATTTTGTGTATCAGGTGGGAATCACGGAATCAGATCATTTAGATCTATTGATTGAGC 5840
cDNA(MLOC_10504)     ---------------------------------------GGGAATCACGGAATCAGATCATTTAGATCTATTGATTGAGC 1640
CircularRNA          NNNNNNNNNNNNNNNNNNNNNNNNNNNNNNNNNNNNNNNGGGAATCACGGAATCAGATCATTTAGATCTATTGATTGAGC 5302

                                5.860               5.880               5.900               5.920
Genomic(MLOC_10504)  AAGTTAAGATGCTTGCTGGGGAGATTGCGTTTGGTACCAGTTCACTGAAAAGACTAATTGAGCAATCCATAGAAGACCCT 5920
cDNA(MLOC_10504)     AAGTTAAGATGCTTGCTGGGGAGATTGCGTTTGGTACCAGTTCACTGAAAA----------------------------- 1691
CircularRNA          AAGTTAAGATGCTTGCTGGGGAGATTGCGTTTGGTACCAGTTCACTGAAAAGACTAATTGAGCAATCCATAGAAGACCCT 5382

                                5.940
Genomic(MLOC_10504)  GAAGGGACAAAAAATCAAGTAGG 5943
cDNA(MLOC_10504)     ----------------------- 1691
CircularRNA          GAAGGGACAAAAAATCAA----- 5400
```

SAD1/UNC-84 domain protein 2_circular RNA (ID: Ch1:60689841-60690191)

CAAAAACCTACACCTGAGCTTCTTACTGAAGGTGGAGTTAATGGGGTCTCAAATGATAAGGTTT
CTAGCAAGAAGGATATTGGTCATACAAGCCGTGGAGAGTCTGTTATCGACAAGCCAAAATATTC
GTCTGAGGCAAAAAAAGATGCATTTCCTCCATCACCTTCTGCTGAACATCGAAAGAAAATTACTA
CTAAACACAGAAAAACCAAGTGGGAAACTGCACTAAGTGTGCTGATGAAGCTTTGCCTTCTTTTT
ACGGCTTTTGCTTGGATGGGGCAGGTGTTATGGGGATGGCA**AAATGGGGATCTATCATTTACA**
GCACTTGATATGGAGAGCAGACTTTCCAAG

The nucleotides of junction-region are underlined. The nucleotides of junction-region which are supported by the junction-spaning sequencing reads are shown in red. Introns are not shown if the absence is supported by sequencing reads. In the absence of supporting sequencing reads, the intronic nucletides are shown as N.

Structural relationship between the circular RNA and its parental gene

```
                      20                  40                  60                  80
Genomic(MLOC_74926)  GCCCTCCTCCTCCCCGCAGCCCTCCCGCGCCGACGGCGGCGGGCCACCGCAGGCCAGCCCCACCGGCGCGGTTCGGTTAC 80
cDNA(MLOC_74926)     GCCCTCCTCCTCCCCGCAGCCCTCCCGCGCCGACGGCGGCGGGCCACCGCAGGCCAGCCCCACCGGCGCGGTTCGGTTAC 80
CircularRNA          -------------------------------------------------------------------------------- -

                      100                 120                 140                 160
Genomic(MLOC_74926)  CCCCTTCGTCGGTAAGGAACCAACAAACCCTAGCTCGATTGCGTGCGAGTCGTTTTTTCCTTCCTCCTCGGGCATCTCCC 160
cDNA(MLOC_74926)     CCCCTTCGTCGGT------------------------------------------------------------------- 93
CircularRNA          -------------------------------------------------------------------------------- -

                      180                 200                 220                 240
Genomic(MLOC_74926)  GTTGCCGCTGCAGCGAGCAACCAAGATCTGAACCGTTTAGTCAGCCGGTTTGTTTGCTCCATGGATGGCGCCCTCTGATC 240
cDNA(MLOC_74926)     -------------------------------------------------------------------------------- 93
CircularRNA          -------------------------------------------------------------------------------- -

                      260                 280                 300                 320
Genomic(MLOC_74926)  CCCAGCCCGATGTGCTCGATTTAGGTCGTTCTCCCTCTTCCTTCTGTCTGGGTTGACCCGTGGTAGTGGTTTAGGGTTTT 320
cDNA(MLOC_74926)     -------------------------------------------------------------------------------- 93
CircularRNA          -------------------------------------------------------------------------------- -

                      340                 360                 380                 400
Genomic(MLOC_74926)  CTCTTTTTTCCTTGGTGATGTATGTAATTGCTTGAACTTGGGGAATTAGAACCGTTGGGAAGTTGCAGGCTTGCTTTCGG 400
cDNA(MLOC_74926)     -------------------------------------------------------------------------------- 93
CircularRNA          -------------------------------------------------------------------------------- -

                      420                 440                 460                 480
Genomic(MLOC_74926)  TTAATTGTTTTTGCCCCTTTCACTGTGGCAGCTGAATATACAATTCGAGATTTGGTTCGAAATGTTGTGTAGGATACCAC 480
cDNA(MLOC_74926)     -------------------------------------------------------------------------------- 93
CircularRNA          -------------------------------------------------------------------------------- -

                      500                 520                 540                 560
Genomic(MLOC_74926)  AGTTTACTCCTTGTCTTCACATTTTACTGCTTGGCTTTATATTTTTGGTCGGCGATGGTAAATGGCCGTACTAGTAGTTA 560
cDNA(MLOC_74926)     -------------------------------------------------------------------------------- 93
CircularRNA          -------------------------------------------------------------------------------- -

                      580                 600                 620                 640
Genomic(MLOC_74926)  AATAACAGTATGGATTCTTTCATTTCTTCGAAATCTGCAGACTCTCCTGTACCTTTATGTCCGATAGTGTATTCTTTTCC 640
cDNA(MLOC_74926)     -------------------------------------------------------------------------------- 93
CircularRNA          -------------------------------------------------------------------------------- -

                      660                 680                 700                 720
Genomic(MLOC_74926)  TACTTACGACGGGCTCTTTGCTGCCCAGCTTTAATTGCTGGATTGCAAAGGAATAAATAATGTATCCGGAGGCTGTTATA 720
cDNA(MLOC_74926)     -------------------------------------------------------------------------------- 93
CircularRNA          -------------------------------------------------------------------------------- -

                      740                 760                 780                 800
Genomic(MLOC_74926)  TGTTACAGTTCATCTTATGCATGTGAACTCCACCTTTTGTTTTAGGCTTACTGTATATAGATTATCCATCCATTACCCTG 800
cDNA(MLOC_74926)     -------------------------------------------------------------------------------- 93
CircularRNA          -------------------------------------------------------------------------------- -

                      820                 840                 860                 880
Genomic(MLOC_74926)  CACAGATAAGTTACCAACAAAAATAAATATGGGAAGGCCGGCTATAAGATAAATTCCTTGGATGTATCCTTCATGTTACC 880
cDNA(MLOC_74926)     -------------------------------------------------------------------------------- 93
CircularRNA          -------------------------------------------------------------------------------- -

                      900                 920                 940                 960
Genomic(MLOC_74926)  CTGCACAGATTTATTGCCAAAAAATAATCATGTAACAGCACTCATTTTCTGGTGCCTTGTTCCATTTGCATTGTTATTTC 960
cDNA(MLOC_74926)     -------------------------------------------------------------------------------- 93
CircularRNA          -------------------------------------------------------------------------------- -

                      980                 1.000               1.020               1.040
Genomic(MLOC_74926)  CAGTTTGTAATCTAGCTATGTTCTATTGTAGGTTAAGGGACAACGATGTCGGCTTCAACTGCTGCAATACCGGCCGCAAA 1040
cDNA(MLOC_74926)     ---------------------------------TAAGGGACAACGATGTCGGCTTCAACTGCTGCAATACCGGCCGCAAA 140
CircularRNA          -------------------------------------------------------------------------------- -

                      1.060               1.080               1.100               1.120
Genomic(MLOC_74926)  TACAAATGGGAACCATGCACTAAGTGTGGATTCACATTCCAGCCAAGATGTTAGGAGGCGAACAGTTGTTGTTGCAAAGC 1120
cDNA(MLOC_74926)     TACAAATGGGAACCATGCACTAAGTGTGGATTCACATTCCAGCCAAGATGTTAGGAGGCGAACAGTTGTTGTTGCAAAGC 220
CircularRNA          -------------------------------------------------------------------------------C 1

                      1.140               1.160               1.180               1.200
Genomic(MLOC_74926)  AAAAACCTACACCTGAGCTTCTTACTGAAGGTGGAGTTAATGGGGTCTCAAATGATAAGGTTTCTAGCAAGAAGGATATT 1200
cDNA(MLOC_74926)     AAAAACCTACACCTGAGCTTCTTACTGAAGGTGGAGTTAATGGGGTCTCAAATGATAAGGTTTCTAGCAAGAAGGATATT 300
CircularRNA          AAAAACCTACACCTGAGCTTCTTACTGAAGGTGGAGTTAATGGGGTCTCAAATGATAAGGTTTCTAGCAAGAAGGATATT 81

                      1.220               1.240               1.260               1.280
Genomic(MLOC_74926)  GGTCATACAAGCCGTGGAGAGTCTGTTATCGACAAGCCAAAATATTCGTCTGAGGCAAAAAAAGATGCATTTCCTCCATC 1280
cDNA(MLOC_74926)     GGTCATACAAGCCGTGGAGAGTCTGTTATCGACAAGCCAAAATATTCGTCTGAGGCAAAAAAAGATGCATTTCCTCCATC 380
CircularRNA          GGTCATACAAGCCGTGGAGAGTCTGTTATCGACAAGCCAAAATATTCGTCTGAGGCAAAAAAAGATGCATTTCCTCCATC 161

                      1.300               1.320               1.340               1.360
Genomic(MLOC_74926)  ACCTTCTGCTGAACATCGAAAGAAAATTACTACTAAACACAGAAAAACCAAGTGGGAAACTGCACTAAGTGTGCTGATGA 1360
cDNA(MLOC_74926)     ACCTTCTGCTGAACATCGAAAGAAAATTACTACTAAACACAGAAAAACCAAGTGGGAAACTGCACTAAGTGTGCTGATGA 460
CircularRNA          ACCTTCTGCTGAACATCGAAAGAAAATTACTACTAAACACAGAAAAACCAAGTGGGAAACTGCACTAAGTGTGCTGATGA 241

                      1.380               1.400               1.420               1.440
Genomic(MLOC_74926)  AGCTTTGCCTTCTTTTTACGGCTTTTGCTTGGATGGGGCAGGTGTTATGGGGATGGCAAAATGGGGATCTATCATTTACA 1440
cDNA(MLOC_74926)     AGCTTTGCCTTCTTTTTACGGCTTTTGCTTGGATGGGGCAGGTGTTATGGGGATGGCAAAATGGGGATCTATCATTTACA 540
CircularRNA          AGCTTTGCCTTCTTTTTACGGCTTTTGCTTGGATGGGGCAGGTGTTATGGGGATGGCAAAATGGGGATCTATCATTTACA 321

                      1.460               1.480               1.500               1.520
Genomic(MLOC_74926)  GCACTTGATATGGAGAGCAGACTTTCCAAGGTAGAGGCGTTCAAGAAGACGACTAGGATGCTCCAGGTGCAGCTGGACAT 1520
cDNA(MLOC_74926)     GCACTTGATATGGAGAGCAGACTTTCCAAGGTAGAGGCGTTCAAGAAGACGACTAGGATGCTCCAGGTGCAGCTGGACAT 620
CircularRNA          GCACTTGATATGGAGAGCAGACTTTCCAAG-------------------------------------------------- 351

                      1.540               1.560               1.580               1.600
Genomic(MLOC_74926)  TTTAGATAAGAAGCTAGGAGATGAGATTGGCAAAGCAAAAAGGGACATCACAAAGCAATTTGATGACAAAGGTAAGAAGT 1600
cDNA(MLOC_74926)     TTTAGATAAGAAGCTAGGAGATGAGATTGGCAAAGCAAAAAGGGACATCACAAAGCAATTTGATGACAAAGGTAAGAAGT 700
CircularRNA          -------------------------------------------------------------------------------- 351
```

```
                        1.620               1.640               1.660               1.680
Genomic(MLOC_74926) TAGAGACAAAGATGAAGACATTGGAAGGCAAGGCTGACATATTAGATAAGTCACTTACTGAACTGAGAGACATGGGTTTT 1680
   cDNA(MLOC_74926) TAGAGACAAAGATGAAGACATTGGAAGGCAAGGCTGACATATTAGATAAGTCACTTACTGAACTGAGAGACATGGGTTTT 780
         CircularRNA -------------------------------------------------------------------------------- 351

                        1.700               1.720               1.740               1.760
Genomic(MLOC_74926) GTCTCTAAGAAAGAATTCGATGAGATTCTGACTCAGTTGAAGAGAAAGAAGGGTTTGGATGGCACTGGGAGTGATATAAC 1760
   cDNA(MLOC_74926) GTCTCTAAGAAAGAATTCGATGAGATTCTGACTCAGTTGAAGAGAAAGAAGGGTTTGGATGGCACTGGGAGTGATATAAC 860
         CircularRNA -------------------------------------------------------------------------------- 351

                        1.780               1.800               1.820               1.840
Genomic(MLOC_74926) CTTGGACGATGTAAGAATATTTGCCAAAGAGATTGTGGAGATGGAGATAGCTAGGCATGCAGCAGATGGCCTTGGAATGG 1840
   cDNA(MLOC_74926) CTTGGACGATGTAAGAATATTTGCCAAAGAGATTGTGGAGATGGAGATAGCTAGGCATGCAGCAGATGGCCTTGGAATGG 940
         CircularRNA -------------------------------------------------------------------------------- 351

                        1.860               1.880               1.900               1.920
Genomic(MLOC_74926) TGGATTATGCATTAGGGTCTGGTGGTGGCAAAGTGGTTAAGCATTCGGGAGCTTTCAAGAAAGCTAAGAGCATTCTTCCT 1920
   cDNA(MLOC_74926) TGGATTATGCATTAGGGTCTGGTGGTGGCAAAGTGGTTAAGCATTCGGGAGCTTTCAAGAAAGCTAAGAGCATTCTTCCT 1020
         CircularRNA -------------------------------------------------------------------------------- 351

                        1.940               1.960               1.980               2.000
Genomic(MLOC_74926) CGTCGTAGTGAAAGTCACAAAATGCTGGAGCCAAGTTTTGGGCAGCCAGGAGAATGTTTTGCACTGGAAGGAAGTGTTGG 2000
   cDNA(MLOC_74926) CGTCGTAGTGAAAGTCACAAAATGCTGGAGCCAAGTTTTGGGCAGCCAGGAGAATGTTTTGCACTGGAAGGAAGTGTTGG 1100
         CircularRNA -------------------------------------------------------------------------------- 351

                        2.020               2.040               2.060               2.080
Genomic(MLOC_74926) ATTCGTGGAAATCAGGCTCAGGACAGGAATTGTTCCTGAGGCAGTCACTCTAGAGCATGTCGACAAGGTATGGCTATTCA 2080
   cDNA(MLOC_74926) ATTCGTGGAAATCAGGCTCAGGACAGGAATTGTTCCTGAGGCAGTCACTCTAGAGCATGTCGACAAG------------- 1167
         CircularRNA -------------------------------------------------------------------------------- 351

                        2.100               2.120               2.140               2.160
Genomic(MLOC_74926) TTGTATTACCATAATTCAATATTACTTTATTTATTTATTTGGCATCTCTTTGTCCAACCCATACGGCGGCTCATTATTTT 2160
   cDNA(MLOC_74926) -------------------------------------------------------------------------------- 1167
         CircularRNA -------------------------------------------------------------------------------- 351

                        2.180               2.200               2.220               2.240
Genomic(MLOC_74926) CCATGCTACAAGCCTTTTCTTTTGTCAACATATCTGATTTGAGATAGCTGACCACATATGCCATGTCGGTGCGTGCTCTG 2240
   cDNA(MLOC_74926) -------------------------------------------------------------------------------- 1167
         CircularRNA -------------------------------------------------------------------------------- 351

                        2.260               2.280               2.300               2.320
Genomic(MLOC_74926) ATTGATCAGCTTCACCTTTGATGCCATTACTATCTATTACTAGGAACTTGCAACTACATGGCATGCAGCTCTATCTGTTA 2320
   cDNA(MLOC_74926) -------------------------------------------------------------------------------- 1167
         CircularRNA -------------------------------------------------------------------------------- 351

                        2.340               2.360               2.380               2.400
Genomic(MLOC_74926) GCGGTATTGGACTTCATTTGTTCTTCCCTCAATCAGGCTTTATTGGGCCATGATAGAAAGAAATCTTTGTGAAAGCAACG 2400
   cDNA(MLOC_74926) -------------------------------------------------------------------------------- 1167
         CircularRNA -------------------------------------------------------------------------------- 351

                        2.420               2.440               2.460               2.480
Genomic(MLOC_74926) CATGTTCTTCATTTTGCTGTGTCCCAGTTGACATGACCGGTTCTTCCATTTTGTTTGTTTACGTTATGATCTTATCATTT 2480
   cDNA(MLOC_74926) -------------------------------------------------------------------------------- 1167
         CircularRNA -------------------------------------------------------------------------------- 351

                        2.500               2.520               2.540               2.560
Genomic(MLOC_74926) AGCAATCATGCCCTGCATGTCCTTTGCTCCCTCGAATCACTTCTCTGCTTAATTCTAGTGTACTACTACTAATCTTATGC 2560
   cDNA(MLOC_74926) -------------------------------------------------------------------------------- 1167
         CircularRNA -------------------------------------------------------------------------------- 351

                        2.580               2.600               2.620               2.640
Genomic(MLOC_74926) GGTTCATAACTGGCCTGTGCAGAGTGTGGCCTTCGACCGCTCCAGCGCTCCCAAGGATTTCCAGGTCAGCGGGTGGTACC 2640
   cDNA(MLOC_74926) ----------------------AGTGTGGCCTTCGACCGCTCCAGCGCTCCCAAGGATTTCCAGGTCAGCGGGTGGTACC 1225
         CircularRNA -------------------------------------------------------------------------------- 351

                        2.660               2.680               2.700               2.720
Genomic(MLOC_74926) AAGGAGCAGACGACGATTCAGACAAGCAACCACGCATGCCGACAAGCCTAGGAGAGTTCACTTACGACCTTGAGAAGAGC 2720
   cDNA(MLOC_74926) AAGGAGCAGACGACGATTCAGACAAGCAACCACGCATGCCGACAAGCCTAGGAGAGTTCACTTACGACCTTGAGAAGAGC 1305
         CircularRNA -------------------------------------------------------------------------------- 351

                        2.740               2.760               2.780               2.800
Genomic(MLOC_74926) AACGCCCAGACCTTCCAGCTAGACAGGACCACCGCCGACGCGTCGGTGGTCAACATGGTCCGGCTCGACTTCTCCTCGAA 2800
   cDNA(MLOC_74926) AACGCCCAGACCTTCCAGCTAGACAGGACCACCGCCGACGCGTCGGTGGTCAACATGGTCCGGCTCGACTTCTCCTCGAA 1385
         CircularRNA -------------------------------------------------------------------------------- 351

                        2.820               2.840               2.860               2.880
Genomic(MLOC_74926) CCACGGGCAGCCTGAGCTGACGTGCATCTACCGCTTCAGGGTGCACGGCAGCGAGCCTGGCTCTCTCGGCACCGCAGCCT 2880
   cDNA(MLOC_74926) CCACGGGCAGCCTGAGCTGACGTGCATCTACCGCTTCAGGGTGCACGGCAGCGAGCCTGGCTCTCTCGGCACCGCAGCCT 1465
         CircularRNA -------------------------------------------------------------------------------- 351

                        2.900               2.920               2.940               2.960
Genomic(MLOC_74926) CGTGAGGCCTGCCTGTTCTAGTAGTTCTGTAGTTGTGTGACGGTCTTTCCTCTTTGGCTTTGAAGACTTGAAGCCCCTGT 2960
   cDNA(MLOC_74926) CGTGAGGCCTGCCTGTTCTAGTAGTTCTGTAGTTGTGTGACGGTCTTTCCTCTTTGGCTTTGAAGACTTGAAGCCCCTGT 1545
         CircularRNA -------------------------------------------------------------------------------- 351

                        2.980               3.000               3.020               3.040
Genomic(MLOC_74926) CAAACGGTAGGTTCAAGTTGATTCTAATCTGTAGCATAATGGTGCCACTATGGACACACCTGACTTGCTGTTCGTATTCC 3040
   cDNA(MLOC_74926) CAAACGGTAGGTTCAAGTTGATTCTAATCTGTAGCATAATGGTGCCACTATGGACACACCTGACTTGCTGTTCGTATTCC 1625
         CircularRNA -------------------------------------------------------------------------------- 351

                        3.060               3.080               3.100               3.120
Genomic(MLOC_74926) TAGCTAGAGGTGCTAATTGTTTGTAGTATCTAGCAACTGTGATTGCTCTCTGTACTGTGGCAGTATTTGTAATGTAAAAT 3120
   cDNA(MLOC_74926) TAGCTAGAGGTGCTAATTGTTTGTAGTATCTAGCAACTGTGATTGCTCTCTGTACTGTGGCAGTATTTGTAATGTAAAAT 1705
         CircularRNA -------------------------------------------------------------------------------- 351

                        3.140               3.160               3.180               3.200
Genomic(MLOC_74926) TGTTAATTATGGTGTGAAGTGTCCTCTTGTATTTGGAGCTTTTATGTGCCGTGAGACTGAGCCATATCTCAACTACGTAA 3200
   cDNA(MLOC_74926) TGTTAATTATGGTGTGAAGTGTCCTCTTGTATTTGGAGCTTTTATGTGCCGTGAGACTGAGCCATATCTCAACTACGTAA 1785
         CircularRNA -------------------------------------------------------------------------------- 351
```

3.220

Genomic(MLOC_74926)	TACTATGAAAAGTGTGCGTTTGGTTC	3226
cDNA(MLOC_74926)	TACTATGAAAAGTGTGCGTTTGGTTC	1811
CircularRNA	--------------------------	351

Probable beta-1-4-glucosyltransferase_circular RNA (ID: Ch4:71095325-71097097)

CACCATCCCATTGAGCCATGAGATCTGAACCAATGAATTCAGATAACATCTACCATGTTTACGG
ATCACTACATTTCCTGTTGGAACAATGCGATATCCCTTTTTCAGGAACCATCTGAAAACGGGCTT
CATGCTTTTCTGAATTATTCAGCGGCACGCATTACATGCTCGCTGTAAGCTTCTAAAAAAATTTAT
ATTTTTGTTGCTAAATGTTCTCTCTTACTAAACTAGCTTCAGGAAAAACAACAGGTTTGCTATCCA
TTTGATCTCGTTTGTTTGAATAGCAGAATTGCTACCTAAATTCTCAAAAGAAAATTGTGCTTTCAC
AGTAATACATATCTGTGATGCGTACTGCATATTGTATGGTACGCTATCCACTTATTCAGGGTNNN
...NNNTAGATATCATCTCTGCTCTAGCCAGGAGAACTTTGACCAGGACAACATTTTACAAAGGTN
NN...NNNTTGGTACTAATTTCTCGAAAAATCGTGCTTTTTGCAGCACCATCCTGTTGTGCCATGA
GATCTGAACCAATGAATTCAGATAACATCTACTATGTTTACGGATCACTACATGTTCCATTGGAAC
AATGTGAGGTNNN...NNNTTGCTACTAAATTCGCAAAAAAAATCGTGCTTTTGCATCACCATCCTG
TGGAACCACGAGACCTGATCCAATGAATTTACTGAACGTTTTCTATGTTTTAAAAATTCTGGATCA
CTACATGTTCTACTGGAACAAGNNN...NNNAGGGAAAGGCAAGGCCCCTGAAGTCCCAATCCCT
ATGAACAATGCTGGAGAAGCGTAGACAAAGAA**AAAGGGGTGCTTAGCTGTGTTGCATCCGCCA**
TTGTTCCTTCCAG

The nucleotides of junction-region are underlined. The nucleotides of junction-region which are supported by the junction-spaning sequencing reads are shown in red. Introns are not shown if the absence is supported by sequencing reads. In the absence of supporting sequencing reads, the intronic nucletides are shown as N.

Structural relationship between the circular RNA and its parental gene

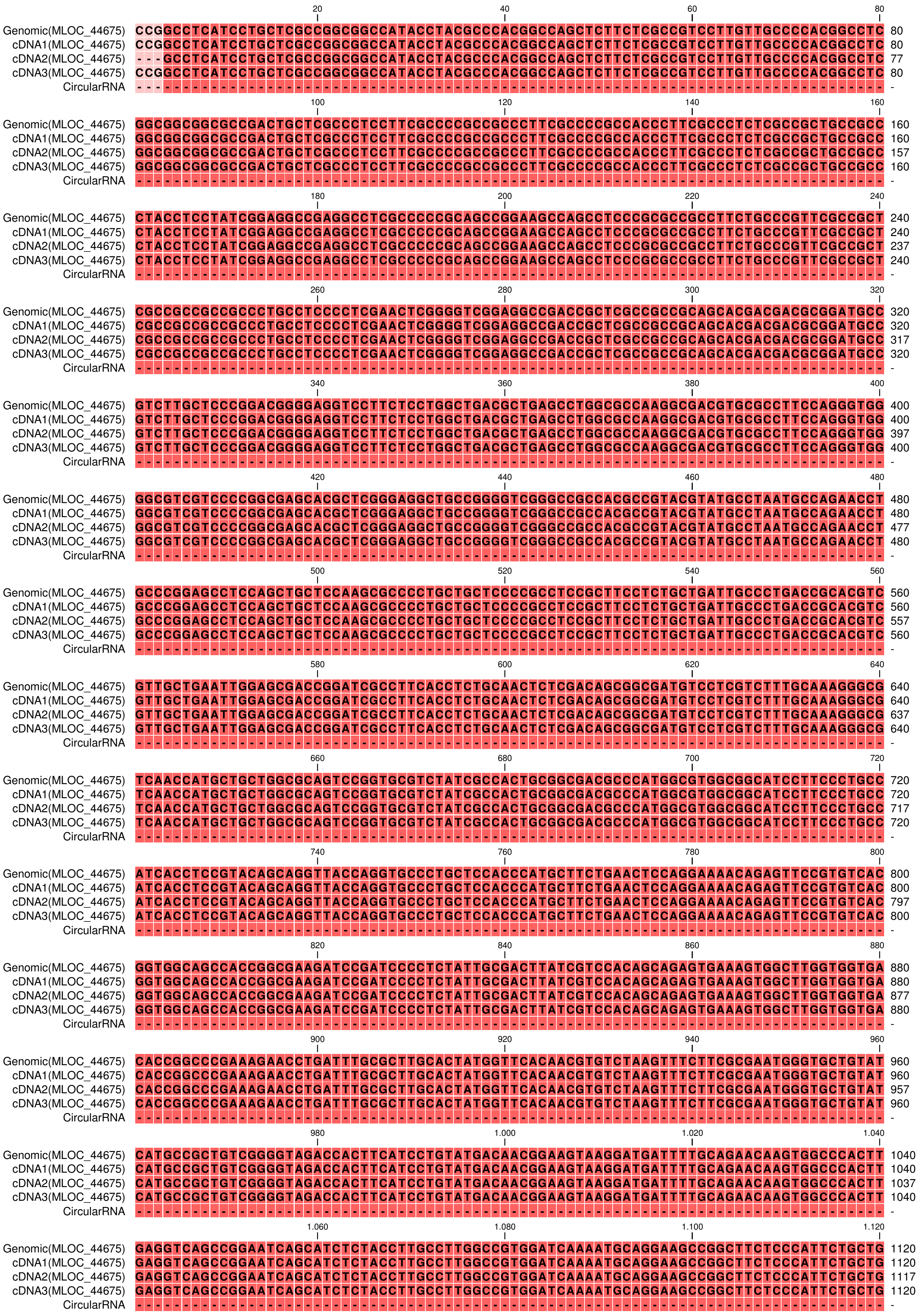
Genomic(MLOC_44675)
cDNA1(MLOC_44675)
cDNA2(MLOC_44675)
cDNA3(MLOC_44675)
CircularRNA

```
                                   1.140               1.160               1.180               1.200
Genomic(MLOC_44675) CAACGCACCAGAGTTCTTGCAAGTGGGTGGCATTTATAGATGTCGACGAATTCATTTTCTCGCCCAACTGGAAAGGATCC 1200
  cDNA1(MLOC_44675) CAACGCACCAGAGTTCTTGCAAGTGGGTGGCATTTATAGATGTCGACGAATTCATTTTCTCGCCCAACTGGAAAGGATCC 1200
  cDNA2(MLOC_44675) CAACGCACCAGAGTTCTTGCAAGTGGGTGGCATTTATAGATGTCGACGAATTCATTTTCTCGCCCAACTGGAAAGGATCC 1197
  cDNA3(MLOC_44675) CAACGCACCAGAGTTCTTGCAAGTGGGTGGCATTTATAGATGTCGACGAATTCATTTTCTCGCCCAACTGGAAAGGATCC 1200
        CircularRNA -------------------------------------------------------------------------------- -

                                   1.220               1.240               1.260               1.280
Genomic(MLOC_44675) GAGAAACCGTCAAAATCAATGCTTCAAGCTATTGTTCCGGTTGATCCAGACGTCGGGCAGGTGTATCTGCCGTGCTTTGA 1280
  cDNA1(MLOC_44675) GAGAAACCGTCAAAATCAATGCTTCAAGCTATTGTTCCGGTTGATCCAGACGTCGGGCAGGTGTATCTGCCGTGCTTTGA 1280
  cDNA2(MLOC_44675) GAGAAACCGTCAAAATCAATGCTTCAAGCTATTGTTCCGGTTGATCCAGACGTCGGGCAGGTGTATCTGCCGTGCTTTGA 1277
  cDNA3(MLOC_44675) GAGAAACCGTCAAAATCAATGCTTCAAGCTATTGTTCCGGTTGATCCAGACGTCGGGCAGGTGTATCTGCCGTGCTTTGA 1280
        CircularRNA -------------------------------------------------------------------------------- -

                                   1.300               1.320               1.340               1.360
Genomic(MLOC_44675) TTTTGCCCCCTCTGGCCAAACATCACACCCACAGGAGGGTGTCATCCAAGGATACACATGCCGCCTGAAGAAGATTCTCA 1360
  cDNA1(MLOC_44675) TTTTGCCCCCTCTGGCCAAACATCACACCCACAGGAGGGTGTCATCCAAGGATACACATGCCGCCTGAAGAAGATTCTCA 1360
  cDNA2(MLOC_44675) TTTTGCCCCCTCTGGCCAAACATCACACCCACAGGAGGGTGTCATCCAAGGATACACATGCCGCCTGAAGAAGATTCTCA 1357
  cDNA3(MLOC_44675) TTTTGCCCCCTCTGGCCAAACATCACACCCACAGGAGGGTGTCATCCAAGGATACACATGCCGCCTGAAGAAGATTCTCA 1360
        CircularRNA -------------------------------------------------------------------------------- -

                                   1.380               1.400               1.420               1.440
Genomic(MLOC_44675) GGCACAAATCGCTGGTTCTTCTTGATGCGGTGGACCATTCTTTGGAGAACGCAATTCACCACTTCACGCTCAAGGCTGGC 1440
  cDNA1(MLOC_44675) GGCACAAATCGCTGGTTCTTCTTGATGCGGTGGACCATTCTTTGGAGAACGCAATTCACCACTTCACGCTCAAGGCTGGC 1440
  cDNA2(MLOC_44675) GGCACAAATCGCTGGTTCTTCTTGATGCGGTGGACCATTCTTTGGAGAACGCAATTCACCACTTCACGCTCAAGGCTGGC 1437
  cDNA3(MLOC_44675) GGCACAAATCGCTGGTTCTTCTTGATGCGGTGGACCATTCTTTGGAGAACGCAATTCACCACTTCACGCTCAAGGCTGGC 1440
        CircularRNA -------------------------------------------------------------------------------- -

                                   1.460               1.480               1.500               1.520
Genomic(MLOC_44675) TTCAGAAGCATATGGAACATGCAGGCGCGTGTCAACCATTATAAATACCAGGCATGGAGTGAGTTCAAGTATAAGTTCAA 1520
  cDNA1(MLOC_44675) TTCAGAAGCATATGGAACATGCAGGCGCGTGTCAACCATTATAAATACCAGGCATGGAGTGAGTTCAAGTATAAGTTCAA 1520
  cDNA2(MLOC_44675) TTCAGAAGCATATGGAACATGCAGGCGCGTGTCAACCATTATAAATACCAGGCATGGAGTGAGTTCAAGTATAAGTTCAA 1517
  cDNA3(MLOC_44675) TTCAGAAGCATATGGAACATGCAGGCGCGTGTCAACCATTATAAATACCAGGCATGGAGTGAGTTCAAGTATAAGTTCAA 1520
        CircularRNA -------------------------------------------------------------------------------- -

                                   1.540               1.560               1.580               1.600
Genomic(MLOC_44675) ACGACGAGTATCCGCCTACGTGGCTGACTGGAGAGATCCGATCAACCTTGAGTCTGCTGACCGGGCCCCCGGCTTAGGAG 1600
  cDNA1(MLOC_44675) ACGACGAGTATCCGCCTACGTGGCTGACTGGAGAGATCCGATCAACCTTGAGTCTGCTGACCGGGCCCCCGGCTTAGGAG 1600
  cDNA2(MLOC_44675) ACGACGAGTATCCGCCTACGTGGCTGACTGGAGAGATCCGATCAACCTTGAGTCTGCTGACCGGGCCCCCGGCTTAGGAG 1597
  cDNA3(MLOC_44675) ACGACGAGTATCCGCCTACGTGGCTGACTGGAGAGATCCGATCAACCTTGAGTCTGCTGACCGGGCCCCCGGCTTAGGAG 1600
        CircularRNA -------------------------------------------------------------------------------- -

                                   1.620               1.640               1.660               1.680
Genomic(MLOC_44675) TTGATGGTGTTGAACCGGTTGGTTGGGCACAAAGGTACTGTGAGGTCAAGGATTACCTTCTTCAGGAATTGAGTGCAAGA 1680
  cDNA1(MLOC_44675) TTGATGGTGTTGAACCGGTTGGTTGGGCACAAAGGTACTGTGAGGTCAAGGATTACCTTCTTCAGGAATTGAGTGCAAGA 1680
  cDNA2(MLOC_44675) TTGATGGTGTTGAACCGGTTGGTTGGGCACAAAGGTACTGTGAGGTCAAGGATTACCTTCTTCAGGAATTGAGTGCAAGA 1677
  cDNA3(MLOC_44675) TTGATGGTGTTGAACCGGTTGGTTGGGCACAAAGGTACTGTGAGGTCAAGGATTACCTTCTTCAGGAATTGAGTGCAAGA 1680
        CircularRNA -------------------------------------------------------------------------------- -

                                   1.700               1.720               1.740               1.760
Genomic(MLOC_44675) TGGTTTGGCACTGGACTAGGAAGTCCGGGATCTCAGGATACTTAGGCCTGGCTTTATCTGTAACCTGCGGGTGTCTAGAA 1760
  cDNA1(MLOC_44675) TGGTTTGGCACTGGACTAGGAAGTCCGGGATCTCAGGATACTTAGGCCTGGCTTTATCTGTAACCTGCGGGTGTCTAGAA 1760
  cDNA2(MLOC_44675) TGGTTTGGCACTGGACTAGGAAGTCCGGGATCTCAGGATACTTAGGCCTGGCTTTATCTGTAACCTGCGGGTGTCTAGAA 1757
  cDNA3(MLOC_44675) TGGTTTGGCACTGGACTAGGAAGTCCGGGATCTCAGGATACTTAGGCCTGGCTTTATCTGTAACCTGCGGGTGTCTAGAA 1760
        CircularRNA -------------------------------------------------------------------------------- -

                                   1.780               1.800               1.820               1.840
Genomic(MLOC_44675) GGATCTAGAGGCATTAGAGATGGAATTGTAATCTAGTTTAAACCTGTCTCCTTGCCTCCCAAGATCAATCTAGAAAACCG 1840
  cDNA1(MLOC_44675) GGATCTAGAGGCATTAGAGATGGAATTGTAATCTAGTTTAAACCTGTCTCCTTGCCTCCCAAGATCAATCTAGAAAACCG 1840
  cDNA2(MLOC_44675) GGATCTAGAGGCATTAGAGATGGAATTGTAATCTAGTTTAAACCTGTCTCCTTGCCTCCCAAGATCAATCTAGAAAACCG 1837
  cDNA3(MLOC_44675) GGATCTAGAGGCATTAGAGATGGAATTGTAATCTAGTTTAAACCTGTCTCCTTGCCTCCCAAGATCAATCTAGAAAACCG 1840
        CircularRNA -------------------------------------------------------------------------------- -

                                   1.860               1.880               1.900               1.920
Genomic(MLOC_44675) ACCTGTACATCGCTTGCCACGTTCGGCACCAATAAATAAACATCAGGCGGCCTCCAATGGAGGTAGAGACGCTTCCACCA 1920
  cDNA1(MLOC_44675) ACCTGTACATCGCTTGCCACGTTCGGCACCAATAAATAAACATCAGGCGGCCTCCAATGGAG------------------ 1902
  cDNA2(MLOC_44675) ACCTGTACATCGCTTGCCACGTTCGGCACCAATAAATAAACATCAGGCGGCCTCCAATGGAGGTAGAGACGCTTCCACCA 1917
  cDNA3(MLOC_44675) ACCTGTACATCGCTTGCCACGTTCGGCACCAATAAATAAACATCAGGCGGCCTCCAATGGAGGTAGAGACGCTTCCACCA 1920
        CircularRNA -------------------------------------------------------------------------------- -

                                   1.940               1.960               1.980               2.000
Genomic(MLOC_44675) TTCTCACATAGTATCACGAGCTATCTTCCTCTTTTGGCAATTCATCTAGATTGATCTGATCATCGTTAGCTCTTCCTCCT 2000
  cDNA1(MLOC_44675) -------------------------------------------------------------------------------- 1902
  cDNA2(MLOC_44675) TTCTCACATAGTATCACGAGCTATCTTCCTCTTTTGGCAATTCATCTAGATTGATCTGATCATCGTTAGCTCTTCCTCCT 1997
  cDNA3(MLOC_44675) TTCTCACATAGTATCACGAGCTATCTTCCTCTTTTGGCAATTCATCTAGATTGATCTGATCATCGTTAGCTCTTCCTCCT 2000
        CircularRNA -------------------------------------------------------------------------------- -

                                   2.020               2.040               2.060               2.080
Genomic(MLOC_44675) GCTGCAGCCCTTCATCCACACCGCCACCGCCATCTCTGAGAATTCTTGTCCAGCACTGAAAAATCCATTCATAAGGTTCA 2080
  cDNA1(MLOC_44675) -------CCCTTCATCCACACCGCCACCGCCATCTCTGAGAATTCTTGTCCAGCACTGAAAAATCCATTCATAAGGTTCA 1975
  cDNA2(MLOC_44675) GCTGCAGCCCTTCATCCACACCGCCACCGCCATCTCTGAGAATTCTTGTCCAGCACTGAAAAATCCATTCATAAGGTTCA 2077
  cDNA3(MLOC_44675) GCTGCAGCCCTTCATCCACACCGCCACCGCCATCTCTGAGAATTCTTGTCCAGCACTGAAAAATCCATTCATAAGGTTCA 2080
        CircularRNA -------------------------------------------------------------------------------- -

                                   2.100               2.120               2.140               2.160
Genomic(MLOC_44675) TAGGATGGCAGAATAAGTTTGCGGAAGGTATTTTTTTGGCACTACTGGTTTTTAAAGTTTGCTGATCTAGAGCTTCTATC 2160
  cDNA1(MLOC_44675) TAGGATGGCAGAATAAGTTTGCGGAAGGTATTTTTTTGGCACTACTGGTTTTTAAAGTTTGCTGATCTAGAGCTTCTATC 2055
  cDNA2(MLOC_44675) TAGGATGGCAGAATAAGTTTGCGGAAGGTATTTTTTTGGCACTACTGGTTTTTAAAGTTTGCTGATCTAGAGCTTCTATC 2157
  cDNA3(MLOC_44675) TAGGATGGCAGAATAAGTTTGCGGAAGGTATTTTTTTGGCACTACTGGTTTTTAAAGTTTGCTGATCTAGAGCTTCTATC 2160
        CircularRNA -------------------------------------------------------------------------------- -

                                   2.180               2.200               2.220               2.240
Genomic(MLOC_44675) GCCTCTTCAGCAATCTAGTGGGTTTAACCTTTAGCTGAAGCATCCTTGCAAATACTGATTCCTATTAGTACATATACCTG 2240
  cDNA1(MLOC_44675) GCCTCTTCAGCAATCTAGTGGGTTTAACCTTTAGCTGAAGCATCCTTGCAAATACTGATTCCTATTAGTACATATACCTG 2135
  cDNA2(MLOC_44675) GCCTCTTCAGCAATCTAGTGGGTTTAACCTTTAGCTGAAGCATCCTTGCAAATACTGATTCCTATTAGTACATATACCTG 2237
  cDNA3(MLOC_44675) GCCTCTTCAGCAATCTAGTGGGTTTAACCTTTAGCTGAAGCATCCTTGCAAATACTGATTCCTATTAGTACATATACCTG 2240
        CircularRNA -------------------------------------------------------------------------------- -
```

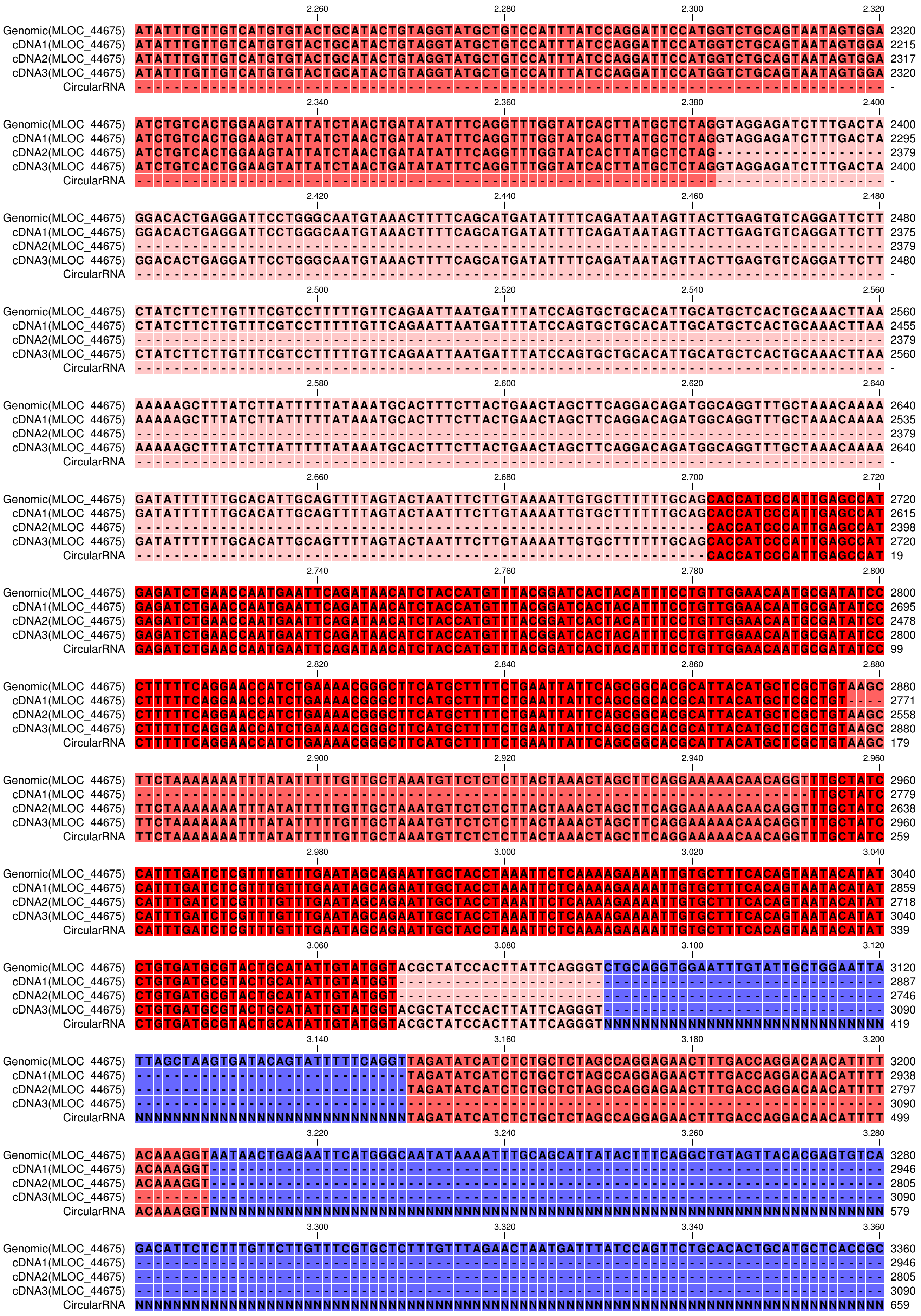

Genomic(MLOC_44675)
cDNA1(MLOC_44675)
cDNA2(MLOC_44675)
cDNA3(MLOC_44675)
CircularRNA

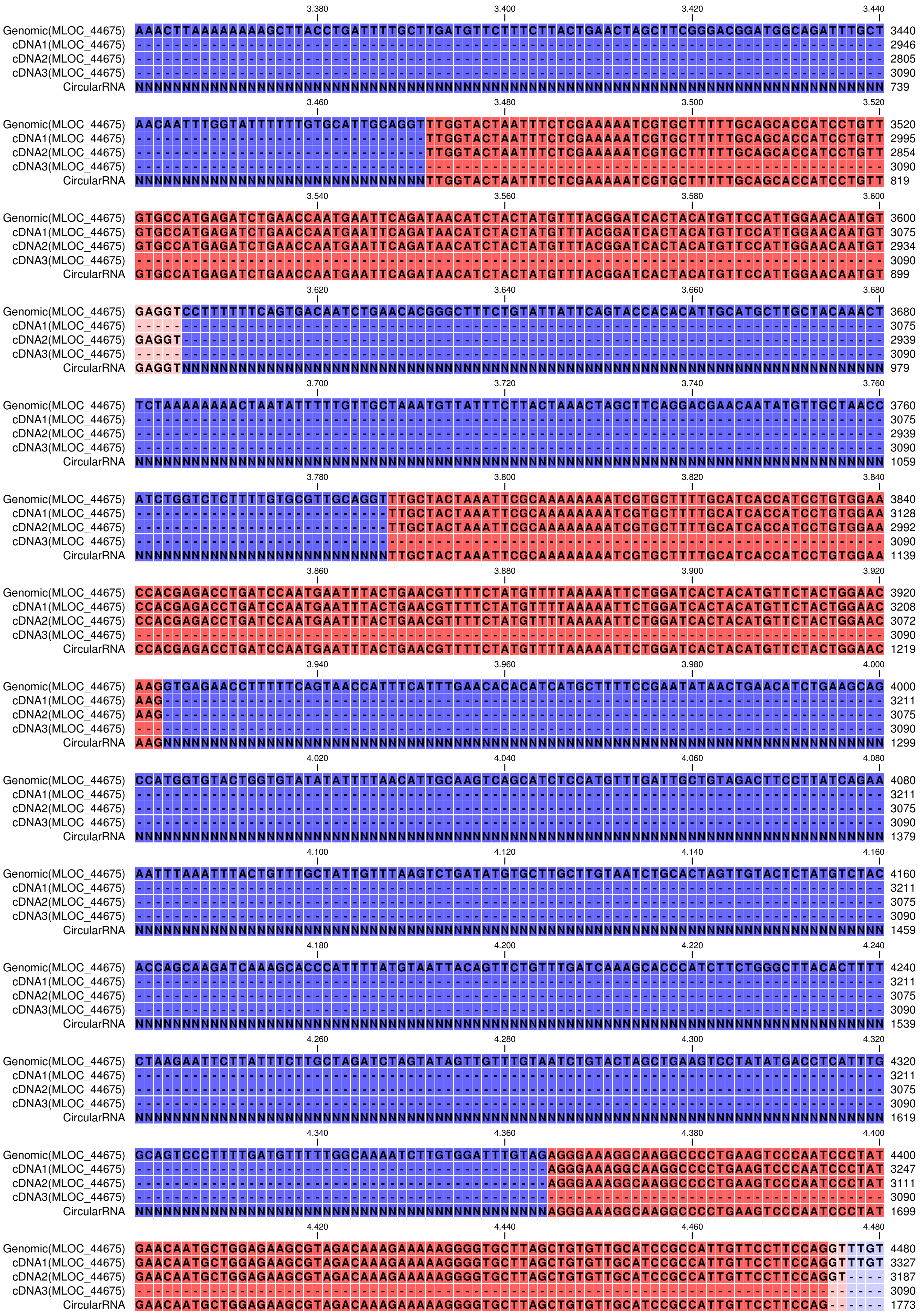
Genomic(MLOC_44675)
cDNA1(MLOC_44675)
cDNA2(MLOC_44675)
cDNA3(MLOC_44675)
CircularRNA

```
                   4.500               4.520               4.540               4.560
Genomic(MLOC_44675) CCCAGGGTTCTTACTGTAAGTTTGCCCCAATTTTTGGTTGTTTCTTTGTCTTGCTCTTCTCTTGCGTGTCCATGCTGTAA 4560
cDNA1(MLOC_44675)   CCCAGGGTTCTTACTGTAAGTTTGCCCCAATTTTTGGTTGTTTCTTTGTCTTGCTCTTCTCTTGCGTGTCCATGCTGTAA 3407
cDNA2(MLOC_44675)   -------------------------------------------------------------------------------- 3187
cDNA3(MLOC_44675)   -------------------------------------------------------------------------------- 3090
CircularRNA         -------------------------------------------------------------------------------- 1773

                   4.580               4.600               4.620               4.640
Genomic(MLOC_44675) GTTTTGGCCCAATTAGGGCCTGATTGGATTGTCGTTTTTCACAGTTTTACACCTGTAAAAGATACAGACCTGAATCGGTC 4640
cDNA1(MLOC_44675)   GTTTTGGCCCAATTAGGGCCTGATTGGATTGTCGTTTTTCACAGTTTTACACCTGTAAAAGATACAGACCTGAATCGGTC 3487
cDNA2(MLOC_44675)   -------------------------------------------------------------------------------- 3187
cDNA3(MLOC_44675)   -------------------------------------------------------------------------------- 3090
CircularRNA         -------------------------------------------------------------------------------- 1773

                   4.660               4.680               4.700               4.720
Genomic(MLOC_44675) GTTTTCTTTTCCGGTTGGAAAGCACATATGGCTGATGATTTACACCTGTAGAAGTAATGGTTGTTTTTCTCAACTTGCCC 4720
cDNA1(MLOC_44675)   GTTTTCTTTTCCGGTTGGAAAGCACATATGGCTGATGATTTACACCTGTAGAAGTAATGGTTGTTTTTCTCAACTTGCCC 3567
cDNA2(MLOC_44675)   -------------------------------------------------------------------------------- 3187
cDNA3(MLOC_44675)   -------------------------------------------------------------------------------- 3090
CircularRNA         -------------------------------------------------------------------------------- 1773

                   4.740               4.760               4.780               4.800
Genomic(MLOC_44675) GCAAGTTCTGGTGTCACGGTCGTTTTGTTGCATTGCTTTTTCATGTAGCTGGGTTGTACTGCCTTCATTTTTTAAATATA 4800
cDNA1(MLOC_44675)   GCAAGTTCTGGTGTCACGGTCGTTTTGTTGCATTGCTTTTTCATGTAGCTGGG--------------------------- 3620
cDNA2(MLOC_44675)   -------------------------------------------------------------------------------- 3187
cDNA3(MLOC_44675)   -------------------------------------------------------------------------------- 3090
CircularRNA         -------------------------------------------------------------------------------- 1773

                   4.820               4.840               4.860               4.880
Genomic(MLOC_44675) AGCCTTTTTAGAGATTTCAATACGAACTATATACATATGTATAGACATATTTTTAGAGTGTAGATCACACATTTTGCTCC 4880
cDNA1(MLOC_44675)   -------------------------------------------------------------------------------- 3620
cDNA2(MLOC_44675)   -------------------------------------------------------------------------------- 3187
cDNA3(MLOC_44675)   -------------------------------------------------------------------------------- 3090
CircularRNA         -------------------------------------------------------------------------------- 1773

                   4.900               4.920               4.940               4.960
Genomic(MLOC_44675) GTATGTAGTCTATTAAAATATCTGAAAAGTCTTATATTTAGGATAGGAACGGAGGGAGTAGTCGTGAGACGTCAGGTGAG 4960
cDNA1(MLOC_44675)   -------------------------------------------------------------------------------- 3620
cDNA2(MLOC_44675)   -------------------------------------------------------------------------------- 3187
cDNA3(MLOC_44675)   -------------------------------------------------------------------------------- 3090
CircularRNA         -------------------------------------------------------------------------------- 1773

                   4.980               5.000               5.020               5.040
Genomic(MLOC_44675) AACGTTTGGCTTTGAAAATCATCTTACATCTAGATGAGGCACAAGGACCTTCAAAAGAGACTCAAGAGAAAAGTTGTCAG 5040
cDNA1(MLOC_44675)   -------------------------------------------------------------------------------- 3620
cDNA2(MLOC_44675)   -------------------------------------------------------------------------------- 3187
cDNA3(MLOC_44675)   -------------------------------------------------------------------------------- 3090
CircularRNA         -------------------------------------------------------------------------------- 1773

                   5.060               5.080               5.100               5.120
Genomic(MLOC_44675) GCTGGCCGTGTTGGAGCAAGCTAGGAAAAGACAAAACATGAGGATTACAAATATCAAAGAGGGGGACGCCAACACCCGTT 5120
cDNA1(MLOC_44675)   -------------------------------------------------------------------------------- 3620
cDNA2(MLOC_44675)   -------------------------------------------------------------------------------- 3187
cDNA3(MLOC_44675)   -------------------------------------------------------------------------------- 3090
CircularRNA         -------------------------------------------------------------------------------- 1773

                   5.140               5.160               5.180               5.200
Genomic(MLOC_44675) TCTTCCATCTTCAAATCAACGGGAGACGACGGAAAAACCTTATCCATAGGCTTAATCATGGGAGTGGCTGGGTGACTAGC 5200
cDNA1(MLOC_44675)   -------------------------------------------------------------------------------- 3620
cDNA2(MLOC_44675)   -------------------------------------------------------------------------------- 3187
cDNA3(MLOC_44675)   -------------------------------------------------------------------------------- 3090
CircularRNA         -------------------------------------------------------------------------------- 1773

                   5.220               5.240               5.260               5.280
Genomic(MLOC_44675) CAAAACCATAAAAACAACATTGTCCAGGCACATTTTGCCAACACTATGAAAAAGGGCTCGCCGCGCCCCAAGACTATCAA 5280
cDNA1(MLOC_44675)   -------------------------------------------------------------------------------- 3620
cDNA2(MLOC_44675)   -------------------------------------------------------------------------------- 3187
cDNA3(MLOC_44675)   -------------------------------------------------------------------------------- 3090
CircularRNA         -------------------------------------------------------------------------------- 1773

                   5.300               5.320               5.340               5.360
Genomic(MLOC_44675) CTGGGCTGCTCTTCCCACTTCGGGCCACGACCTTTCCGACCTTTGGGAGCCCATAACGGAGGACGAGGTCAAGGATGCTA 5360
cDNA1(MLOC_44675)   -------------------------------------------------------------------------------- 3620
cDNA2(MLOC_44675)   -------------------------------------------------------------------------------- 3187
cDNA3(MLOC_44675)   -------------------------------------------------------------------------------- 3090
CircularRNA         -------------------------------------------------------------------------------- 1773

                   5.380               5.400               5.420               5.440
Genomic(MLOC_44675) TATCCGCTCTCCCCGGGGACAAGGTCTCGGGACCTGATGGTTTCACCGGGAAATTTTACAAGGCCTGTTGGGACATTATC 5440
cDNA1(MLOC_44675)   -------------------------------------------------------------------------------- 3620
cDNA2(MLOC_44675)   -------------------------------------------------------------------------------- 3187
cDNA3(MLOC_44675)   -------------------------------------------------------------------------------- 3090
CircularRNA         -------------------------------------------------------------------------------- 1773

                   5.460               5.480               5.500               5.520
Genomic(MLOC_44675) AAGGATGATATCATACTCGTGATCAACAAGTTCTCTAGTCTGCATGCAAAAAACTTTCATTGGCTCAACTCGGCAAACAT 5520
cDNA1(MLOC_44675)   -------------------------------------------------------------------------------- 3620
cDNA2(MLOC_44675)   -------------------------------------------------------------------------------- 3187
cDNA3(MLOC_44675)   -------------------------------------------------------------------------------- 3090
CircularRNA         -------------------------------------------------------------------------------- 1773

                   5.540               5.560               5.580               5.600
Genomic(MLOC_44675) CGCCCTCATCCCTAAGAAAGATGGCGCCAAGAACATTTCAGACTTGAGACCCATCAGCCTTATACACGTTGTTGCAAAGC 5600
cDNA1(MLOC_44675)   -------------------------------------------------------------------------------- 3620
cDNA2(MLOC_44675)   -------------------------------------------------------------------------------- 3187
cDNA3(MLOC_44675)   -------------------------------------------------------------------------------- 3090
CircularRNA         -------------------------------------------------------------------------------- 1773
```

```
                    5.620               5.640               5.660               5.680
Genomic(MLOC_44675) TCATTGTCAAAATCCTTGTGACTAGGCTGGCCCGTCACATGAACGGTCTCGTCTCCATTGCGGAAAGCGCTTTCATCAAA 5680
cDNA1(MLOC_44675)   -------------------------------------------------------------------------------- 3620
cDNA2(MLOC_44675)   -------------------------------------------------------------------------------- 3187
cDNA3(MLOC_44675)   -------------------------------------------------------------------------------- 3090
CircularRNA         -------------------------------------------------------------------------------- 1773

                    5.700               5.720               5.740               5.760
Genomic(MLOC_44675) ACATGGACTATTCATGAAGACTTCATGTATGTTCAAAACTTGGCTAGGAAATTTCACCACAAAGAAAACCCCAATGCTCC 5760
cDNA1(MLOC_44675)   -------------------------------------------------------------------------------- 3620
cDNA2(MLOC_44675)   -------------------------------------------------------------------------------- 3187
cDNA3(MLOC_44675)   -------------------------------------------------------------------------------- 3090
CircularRNA         -------------------------------------------------------------------------------- 1773

                    5.780               5.800               5.820               5.840
Genomic(MLOC_44675) TCTTCAAGCTTGATATCAAGAAGGCTTTTGACTCGGTCAAATGAGACTATATTATTGAGCTGCTAACCCACCATGGCTTC 5840
cDNA1(MLOC_44675)   -------------------------------------------------------------------------------- 3620
cDNA2(MLOC_44675)   -------------------------------------------------------------------------------- 3187
cDNA3(MLOC_44675)   -------------------------------------------------------------------------------- 3090
CircularRNA         -------------------------------------------------------------------------------- 1773

                    5.860               5.880               5.900               5.920
Genomic(MLOC_44675) TCGAGTCACTTTCATGGATGGGTGTCGGCCCTACTTTCCTCGGCCTCCTCCAGAGTTTTGCTCAATGGAATTGCTGGTGA 5920
cDNA1(MLOC_44675)   -------------------------------------------------------------------------------- 3620
cDNA2(MLOC_44675)   -------------------------------------------------------------------------------- 3187
cDNA3(MLOC_44675)   -------------------------------------------------------------------------------- 3090
CircularRNA         -------------------------------------------------------------------------------- 1773

                    5.940               5.960               5.980               6.000
Genomic(MLOC_44675) CCTGATCAAGCATGTATGTGGCCTCCGACATGGCAACCCTCTCTCACCGTTGCTCTTTGTGCTCGCCATTGACCCACTGC 6000
cDNA1(MLOC_44675)   -------------------------------------------------------------------------------- 3620
cDNA2(MLOC_44675)   -------------------------------------------------------------------------------- 3187
cDNA3(MLOC_44675)   -------------------------------------------------------------------------------- 3090
CircularRNA         -------------------------------------------------------------------------------- 1773

                    6.020               6.040               6.060               6.080
Genomic(MLOC_44675) ATCACATCTTGGGCAAGGCCACTTCCCAGGGGTTGCTACACTCACCCGACAACCGCTTCACGGGCATTAGAGCCTCATTA 6080
cDNA1(MLOC_44675)   -------------------------------------------------------------------------------- 3620
cDNA2(MLOC_44675)   -------------------------------------------------------------------------------- 3187
cDNA3(MLOC_44675)   -------------------------------------------------------------------------------- 3090
CircularRNA         -------------------------------------------------------------------------------- 1773

                    6.100               6.120               6.140               6.160
Genomic(MLOC_44675) TATGCTGACGACACAATGGTTTTTTGCGCCCCTCTCAAGGAGGATGTCAGTTTCATTGCCAACATGCTCTCCTCCTTCGG 6160
cDNA1(MLOC_44675)   -------------------------------------------------------------------------------- 3620
cDNA2(MLOC_44675)   -------------------------------------------------------------------------------- 3187
cDNA3(MLOC_44675)   -------------------------------------------------------------------------------- 3090
CircularRNA         -------------------------------------------------------------------------------- 1773

                    6.180               6.200               6.220               6.240
Genomic(MLOC_44675) TGATTCCACTGGGCTCGTCACTAACTGTACAAAGCGCTTAGTCGCCCCATCCAGAGCGCTAATGTGGACCTCGACAACAG 6240
cDNA1(MLOC_44675)   -------------------------------------------------------------------------------- 3620
cDNA2(MLOC_44675)   -------------------------------------------------------------------------------- 3187
cDNA3(MLOC_44675)   -------------------------------------------------------------------------------- 3090
CircularRNA         -------------------------------------------------------------------------------- 1773

                    6.260               6.280               6.300               6.320
Genomic(MLOC_44675) TACGGTCATCATTCCCGCCAGTACGGTCATCATTCCCGCCAGTACGGTCATCATTCCCAATTGCTACTTAGGCCTCCCAC 6320
cDNA1(MLOC_44675)   -------------------------------------------------------------------------------- 3620
cDNA2(MLOC_44675)   -------------------------------------------------------------------------------- 3187
cDNA3(MLOC_44675)   -------------------------------------------------------------------------------- 3090
CircularRNA         -------------------------------------------------------------------------------- 1773

                    6.340               6.360               6.380               6.400
Genomic(MLOC_44675) TTGCGATTTTACGACTCAAGAAGATACATTTGCAACACCTCGAAGACAAGGTGGCCAAGAAACTTGCACCTTGGAGAGGA 6400
cDNA1(MLOC_44675)   -------------------------------------------------------------------------------- 3620
cDNA2(MLOC_44675)   -------------------------------------------------------------------------------- 3187
cDNA3(MLOC_44675)   -------------------------------------------------------------------------------- 3090
CircularRNA         -------------------------------------------------------------------------------- 1773

                    6.420               6.440               6.460               6.480
Genomic(MLOC_44675) AGACATGTGGCTATTGCGGGCCGAATGGCCTTGGTCAAAGCGGTTCTCATGGCGGTGGCCATTTATCACATCACTCTGAT 6480
cDNA1(MLOC_44675)   -------------------------------------------------------------------------------- 3620
cDNA2(MLOC_44675)   -------------------------------------------------------------------------------- 3187
cDNA3(MLOC_44675)   -------------------------------------------------------------------------------- 3090
CircularRNA         -------------------------------------------------------------------------------- 1773

                    6.500               6.520               6.540               6.560
Genomic(MLOC_44675) GGACCTTCCGGTGGAGGTGCTCCAAAAATTTGGTAGTCTGAGGAGAGCCTACCTTTGGGCTGGTACAAACACCGTGTCCG 6560
cDNA1(MLOC_44675)   -------------------------------------------------------------------------------- 3620
cDNA2(MLOC_44675)   -------------------------------------------------------------------------------- 3187
cDNA3(MLOC_44675)   -------------------------------------------------------------------------------- 3090
CircularRNA         -------------------------------------------------------------------------------- 1773

                    6.580               6.600               6.620               6.640
Genomic(MLOC_44675) GAGGCAAATGCAAAATTAACTGGCAGCACGTATGTAGGCCCAAAGAGTTTGGGGATTGGTAATCCTCAATCTGCAAAAGT 6640
cDNA1(MLOC_44675)   -------------------------------------------------------------------------------- 3620
cDNA2(MLOC_44675)   -------------------------------------------------------------------------------- 3187
cDNA3(MLOC_44675)   -------------------------------------------------------------------------------- 3090
CircularRNA         -------------------------------------------------------------------------------- 1773

                    6.660               6.680               6.700               6.720
Genomic(MLOC_44675) TCGCCACCACGCTCCTACTTAGATGGCTTTGGTTGGAGTGGGATTACCCGCCAAGGGCGTGGTACGGTATTGCGGTCCCA 6720
cDNA1(MLOC_44675)   -------------------------------------------------------------------------------- 3620
cDNA2(MLOC_44675)   -------------------------------------------------------------------------------- 3187
cDNA3(MLOC_44675)   -------------------------------------------------------------------------------- 3090
CircularRNA         -------------------------------------------------------------------------------- 1773
```

```
                                                                    6.740                       6.760                       6.780                       6.800
Genomic(MLOC_44675)  TGCAACATGAAGGATCGTGGTTTTTTCAGGACCGCCACTAAGGTTACCCTCGGAGACGACTCCAAGGCCAAGTTCTGGGA  6800
cDNA1(MLOC_44675)    --------------------------------------------------------------------------------  3620
cDNA2(MLOC_44675)    --------------------------------------------------------------------------------  3187
cDNA3(MLOC_44675)    --------------------------------------------------------------------------------  3090
CircularRNA          --------------------------------------------------------------------------------  1773

                                                                    6.820                       6.840                       6.860                       6.880
Genomic(MLOC_44675)  ATCATCTTGGTTAGATGCCATTGACCCCAAAGACATCGTCTTAGAAATCTTCGAGCTCTCAAGGAACAAGTGCGGTACCG  6880
cDNA1(MLOC_44675)    --------------------------------------------------------------------------------  3620
cDNA2(MLOC_44675)    --------------------------------------------------------------------------------  3187
cDNA3(MLOC_44675)    --------------------------------------------------------------------------------  3090
CircularRNA          --------------------------------------------------------------------------------  1773

                                                                    6.900                       6.920                       6.940                       6.960
Genomic(MLOC_44675)  TAGCCTCAGCCCTTAGGGATGACCACTGGATAACCTTGATTGACACAAGAGCAGGTTTATCGCTCGTCCGATTCGAACAA  6960
cDNA1(MLOC_44675)    --------------------------------------------------------------------------------  3620
cDNA2(MLOC_44675)    --------------------------------------------------------------------------------  3187
cDNA3(MLOC_44675)    --------------------------------------------------------------------------------  3090
CircularRNA          --------------------------------------------------------------------------------  1773

                                                                    6.980                       7.000                       7.020                       7.040
Genomic(MLOC_44675)  TTTGTAGCTCTTTGGAACAAGCTCCTACACATCACTATCCAACCTGGTGTGCAGGACACCATCCACTGGAAGCTCTCCAG  7040
cDNA1(MLOC_44675)    --------------------------------------------------------------------------------  3620
cDNA2(MLOC_44675)    --------------------------------------------------------------------------------  3187
cDNA3(MLOC_44675)    --------------------------------------------------------------------------------  3090
CircularRNA          --------------------------------------------------------------------------------  1773

                                                                    7.060                       7.080                       7.100                       7.120
Genomic(MLOC_44675)  CGATGGGGCCTACACTGCCAAGACGTCCTACTTAGCGAAATTTGGCGGCCTTGTTCGCTCCAACTTGCAAACTATTGTCT  7120
cDNA1(MLOC_44675)    --------------------------------------------------------------------------------  3620
cDNA2(MLOC_44675)    --------------------------------------------------------------------------------  3187
cDNA3(MLOC_44675)    --------------------------------------------------------------------------------  3090
CircularRNA          --------------------------------------------------------------------------------  1773

                                                                    7.140                       7.160                       7.180                       7.200
Genomic(MLOC_44675)  GGAGGCTTGGGCCCCTCCCAAGTGTATTTTTTGCATGGTTGGTCACGCAAAATTGGATCTGGACGACGGACAGACTCCAA  7200
cDNA1(MLOC_44675)    --------------------------------------------------------------------------------  3620
cDNA2(MLOC_44675)    --------------------------------------------------------------------------------  3187
cDNA3(MLOC_44675)    --------------------------------------------------------------------------------  3090
CircularRNA          --------------------------------------------------------------------------------  1773

                                                                    7.220                       7.240                       7.260                       7.280
Genomic(MLOC_44675)  CGCCGTGGATGGCCTAACTGTAATTGTTGCCCTCTTTGCAATCAGGTGCAAGAGTCCACGACTCACCTCCTATCAAGTGA  7280
cDNA1(MLOC_44675)    --------------------------------------------------------------------------------  3620
cDNA2(MLOC_44675)    -----------------------------------------------GCAAGAGTCCACGACTCACCTCCTATCAAGTGA  3220
cDNA3(MLOC_44675)    --------------------------------------------------------------------------------  3090
CircularRNA          --------------------------------------------------------------------------------  1773

                                                                    7.300                       7.320                       7.340                       7.360
Genomic(MLOC_44675)  CGCTTCTCCATTCGAGTCTGGAATGAGGTCCTGACGTGGATGTACCTTGATGTCGATACCGCCCTTTGGTTGAACTACGA  7360
cDNA1(MLOC_44675)    --------------------------------------------------------------------------------  3620
cDNA2(MLOC_44675)    CGCTTCTCCATTCGAGTCTGGAATGAGGTCCTGACGTGGATGTACCTTGATGTCGATACCGCCCTTTGGTTGAACTACGA  3300
cDNA3(MLOC_44675)    --------------------------------------------------------------------------------  3090
CircularRNA          --------------------------------------------------------------------------------  1773

                                                                    7.380                       7.400                       7.420                       7.440
Genomic(MLOC_44675)  CACAGTGAGATTATGGTGGGATGGGGTTATCACCAAGAGCATGCTGCCAAAGAAAGCCTTGGCCTCCTTACTCCATCTTG  7440
cDNA1(MLOC_44675)    --------------------------------------------------------------------------------  3620
cDNA2(MLOC_44675)    CACAGTGAGATTATGGTGGGATGGGGTTATCACCAAGAGCATGCTGCCAAAGAAAGCCTTGGCCTCCTTACTCCATCTTG  3380
cDNA3(MLOC_44675)    --------------------------------------------------------------------------------  3090
CircularRNA          --------------------------------------------------------------------------------  1773

                                                                    7.460                       7.480                       7.500                       7.520
Genomic(MLOC_44675)  TTAGCTGGGAGAGTTGGAAGGAGTGAAATGCCCCGAGTGTTCAGATCCAAGGCGGCGCCTGTGGTGGTGATCATGCGTAC  7520
cDNA1(MLOC_44675)    --------------------------------------------------------------------------------  3620
cDNA2(MLOC_44675)    TTAGCTGGGAGAGTTGGAAGGAGTGAAATGCCCCGAGTGTTCAGATCCAAGGCGGCGCCTGTGGTGGTGATCATGCGTAC  3460
cDNA3(MLOC_44675)    --------------------------------------------------------------------------------  3090
CircularRNA          --------------------------------------------------------------------------------  1773

                                                                    7.540                       7.560                       7.580                       7.600
Genomic(MLOC_44675)  TATTAAAGACGAGGCGTCCATCTGCGCCCTGGCGGGTGCTAAGCATTTGTGTAATTTGATGCCGCGAGAGTAGTCCGTTG  7600
cDNA1(MLOC_44675)    --------------------------------------------------------------------------------  3620
cDNA2(MLOC_44675)    TATTAAAGACGAGGCGTCCATCTGCGCCCTGGCGGGTGCTAAGCATTTGTGTAATTTGATGCCGCGAGAGTAGTCCGTTG  3540
cDNA3(MLOC_44675)    --------------------------------------------------------------------------------  3090
CircularRNA          --------------------------------------------------------------------------------  1773

                                                                    7.620                       7.640                       7.660
Genomic(MLOC_44675)  TAATATTCATGCTTGGCGGGACTGTGATGCCAAAAACCTTCTCTTCTCCTTATTCCATGAAATTGGCAAGTC  7672
cDNA1(MLOC_44675)    ------------------------------------------------------------------------  3620
cDNA2(MLOC_44675)    TAATATTCATGCTTGGCGGGACTGTGATGCCAAAAACCTTCTCTTCTCCTTATTCCATGAAATTGGCAAGTC  3612
cDNA3(MLOC_44675)    ------------------------------------------------------------------------  3090
CircularRNA          ------------------------------------------------------------------------  1773
```

Unknown_circular RNA (ID: Ch7:4237959-4238849)

GTCAGGTTAATGAGGATGTCGAGAGCAGATTCCAGCATTTGGCAAGTTCAGTACAAAATGATGT
TATGCAGTTAAAGGCGACGACCAGAGAGGACGAGGGAGGCACGCACACCCAAGACGTCGTTGC
CGTCGTTCCACGGATGACGAGGAAGAGGGCAAGGGAGGCATGCGCATCGGAGACTGCGTTGC
CATCGTTGCAGCACACTCGACTCACGCGTAATGGGAGCAAGGGGGAGGCGCCGACCAGTGGTT
GCTCCGGTAAGTGGAAGACTGTATTAGCACCATAAATCTTTTTAGTCCCACATTGCATTGGTAGT
AGTAAACACCAAATTGATTACCTAGAGACCTGTAGCTGTACAGTGATAGAGGTGTAGGCATAAAT
GTCTACGGTTACAGAGTGAAAAAACCCGAACTCACATGTATATATATATATATATCCTTCTGATTT
TAAGATGACCCAAGTGATTTAACACTCTTCCAGAAAATATCTTACAATTAGATTCTCTAGTTTGTG
ATTAGGTCCCACTGCTTGAAGCTCTAGACAAATATTTGGATAAAGCTGTCAAATTTTCATGATATT
CAGAAGTATATGTTCTGGTGAAATGAAATGCCCAATTGATGGTGTTTCGACAGATCAGGTTAATA
AGGATGTCGAGAGCAGATTCCGGCATTTGGCAAGTTCAGTACAAAATGATGTTATGCAGTTAGA
GGCAGCGACTGGAGAGGACGGGGGAGGCGCGCGCATCCAAGACCTCGTTACCATTGTTACAC
GGATGACGAGGAGGGCGGCGAGGGAGGCATGCGCATCAGAGACCGCATTGGTGTCATTGCAA
CGTATTCGACTCA**CACGCGGCGGAAGCAGGGGGGAGGCGCCGACCAGTGGCCGCTCCG**

The nucleotides of junction-region are underlined. The nucleotides of junction-region which are supported by the junction-spaning sequencing reads are shown in red. Introns are not shown if the absence is supported by sequencing reads. In the absence of supporting sequencing reads, the intronic nucletides are shown as N.

Structural relationship between the circular RNA and its parental gene

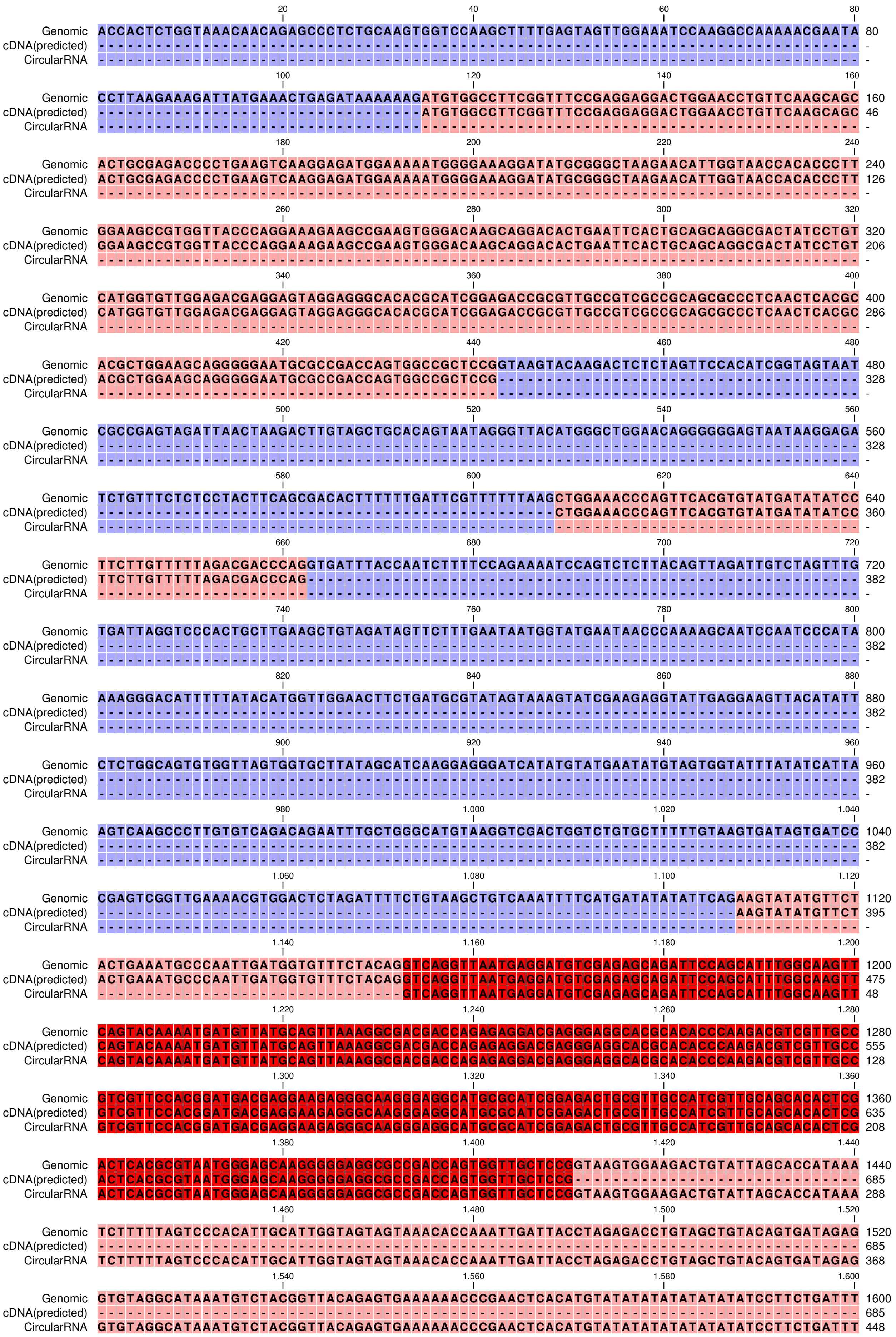

20 40 60 80
Genomic ACCACTCTGGTAAACAACAGAGCCCTCTGCAAGTGGTCCAAGCTTTTGAGTAGTTGGAAATCCAAGGCCAAAAACGAATA 80
cDNA(predicted) -- -
CircularRNA -- -
100 120 140 160
Genomic CCTTAAGAAAGATTATGAAACTGAGATAAAAAAGATGTGGCCTTCGGTTTCCGAGGAGGACTGGAACCTGTTCAAGCAGC 160
cDNA(predicted) ----------------------------------ATGTGGCCTTCGGTTTCCGAGGAGGACTGGAACCTGTTCAAGCAGC 46
CircularRNA -- -
180 200 220 240
Genomic ACTGCGAGACCCCTGAAGTCAAGGAGATGGAAAAATGGGGAAAGGATATGCGGGCTAAGAACATTGGTAACCACACCCTT 240
cDNA(predicted) ACTGCGAGACCCCTGAAGTCAAGGAGATGGAAAAATGGGGAAAGGATATGCGGGCTAAGAACATTGGTAACCACACCCTT 126
CircularRNA -- -
260 280 300 320
Genomic GGAAGCCGTGGTTACCCAGGAAAGAAGCCGAAGTGGGACAAGCAGGACACTGAATTCACTGCAGCAGGCGACTATCCTGT 320
cDNA(predicted) GGAAGCCGTGGTTACCCAGGAAAGAAGCCGAAGTGGGACAAGCAGGACACTGAATTCACTGCAGCAGGCGACTATCCTGT 206
CircularRNA -- -
340 360 380 400
Genomic CATGGTGTTGGAGACGAGGAGTAGGAGGGCACACGCATCGGAGACCGCGTTGCCGTCGCCGCAGCGCCCTCAACTCACGC 400
cDNA(predicted) CATGGTGTTGGAGACGAGGAGTAGGAGGGCACACGCATCGGAGACCGCGTTGCCGTCGCCGCAGCGCCCTCAACTCACGC 286
CircularRNA -- -
420 440 460 480
Genomic ACGCTGGAAGCAGGGGGAATGCGCCGACCAGTGGCCGCTCCGGTAAGTACAAGACTCTCTAGTTCCACATCGGTAGTAAT 480
cDNA(predicted) ACGCTGGAAGCAGGGGGAATGCGCCGACCAGTGGCCGCTCCG-------------------------------------- 328
CircularRNA -- -
500 520 540 560
Genomic CGCCGAGTAGATTAACTAAGACTTGTAGCTGCACAGTAATAGGGTTACATGGGCTGGAACAGGGGGGAGTAATAAGGAGA 560
cDNA(predicted) -- 328
CircularRNA -- -
580 600 620 640
Genomic TCTGTTTCTCTCCTACTTCAGCGACACTTTTTTGATTCGTTTTTTAAGCTGGAAACCCAGTTCACGTGTATGATATATCC 640
cDNA(predicted) --CTGGAAACCCAGTTCACGTGTATGATATATCC 360
CircularRNA -- -
660 680 700 720
Genomic TTCTTGTTTTTAGACGACCCAGGTGATTTACCAATCTTTTCCAGAAAATCCAGTCTCTTACAGTTAGATTGTCTAGTTTG 720
cDNA(predicted) TTCTTGTTTTTAGACGACCCAG-- 382
CircularRNA -- -
740 760 780 800
Genomic TGATTAGGTCCCACTGCTTGAAGCTGTAGATAGTTCTTTGAATAATGGTATGAATAACCCAAAAGCAATCCAATCCCATA 800
cDNA(predicted) -- 382
CircularRNA -- -
820 840 860 880
Genomic AAAGGGACATTTTTATACATGGTTGGAACTTCTGATGCGTATAGTAAAGTATCGAAGAGGTATTGAGGAAGTTACATATT 880
cDNA(predicted) -- 382
CircularRNA -- -
900 920 940 960
Genomic CTCTGGCAGTGTGGTTAGTGGTGCTTATAGCATCAAGGAGGGATCATATGTATGAATATGTAGTGGTATTTATATCATTA 960
cDNA(predicted) -- 382
CircularRNA -- -
980 1.000 1.020 1.040
Genomic AGTCAAGCCCTTGTGTCAGACAGAATTTGCTGGGCATGTAAGGTCGACTGGTCTGTGCTTTTTGTAAGTGATAGTGATCC 1040
cDNA(predicted) -- 382
CircularRNA -- -
1.060 1.080 1.100 1.120
Genomic CGAGTCGGTTGAAAACGTGGACTCTAGATTTTCTGTAAGCTGTCAAATTTTCATGATATATATTCAGAAGTATATGTTCT 1120
cDNA(predicted) ---AAGTATATGTTCT 395
CircularRNA -- -
1.140 1.160 1.180 1.200
Genomic ACTGAAATGCCCAATTGATGGTGTTTCTACAGGTCAGGTTAATGAGGATGTCGAGAGCAGATTCCAGCATTTGGCAAGTT 1200
cDNA(predicted) ACTGAAATGCCCAATTGATGGTGTTTCTACAGGTCAGGTTAATGAGGATGTCGAGAGCAGATTCCAGCATTTGGCAAGTT 475
CircularRNA --------------------------------GTCAGGTTAATGAGGATGTCGAGAGCAGATTCCAGCATTTGGCAAGTT 48
1.220 1.240 1.260 1.280
Genomic CAGTACAAAATGATGTTATGCAGTTAAAGGCGACGACCAGAGAGGACGAGGGAGGCACGCACACCCAAGACGTCGTTGCC 1280
cDNA(predicted) CAGTACAAAATGATGTTATGCAGTTAAAGGCGACGACCAGAGAGGACGAGGGAGGCACGCACACCCAAGACGTCGTTGCC 555
CircularRNA CAGTACAAAATGATGTTATGCAGTTAAAGGCGACGACCAGAGAGGACGAGGGAGGCACGCACACCCAAGACGTCGTTGCC 128
1.300 1.320 1.340 1.360
Genomic GTCGTTCCACGGATGACGAGGAAGAGGGCAAGGGAGGCATGCGCATCGGAGACTGCGTTGCCATCGTTGCAGCACACTCG 1360
cDNA(predicted) GTCGTTCCACGGATGACGAGGAAGAGGGCAAGGGAGGCATGCGCATCGGAGACTGCGTTGCCATCGTTGCAGCACACTCG 635
CircularRNA GTCGTTCCACGGATGACGAGGAAGAGGGCAAGGGAGGCATGCGCATCGGAGACTGCGTTGCCATCGTTGCAGCACACTCG 208
1.380 1.400 1.420 1.440
Genomic ACTCACGCGTAATGGGAGCAAGGGGGAGGCGCCGACCAGTGGTTGCTCCGGTAAGTGGAAGACTGTATTAGCACCATAAA 1440
cDNA(predicted) ACTCACGCGTAATGGGAGCAAGGGGGAGGCGCCGACCAGTGGTTGCTCCG------------------------------ 685
CircularRNA ACTCACGCGTAATGGGAGCAAGGGGGAGGCGCCGACCAGTGGTTGCTCCGGTAAGTGGAAGACTGTATTAGCACCATAAA 288
1.460 1.480 1.500 1.520
Genomic TCTTTTTAGTCCCACATTGCATTGGTAGTAGTAAACACCAAATTGATTACCTAGAGACCTGTAGCTGTACAGTGATAGAG 1520
cDNA(predicted) -- 685
CircularRNA TCTTTTTAGTCCCACATTGCATTGGTAGTAGTAAACACCAAATTGATTACCTAGAGACCTGTAGCTGTACAGTGATAGAG 368
1.540 1.560 1.580 1.600
Genomic GTGTAGGCATAAATGTCTACGGTTACAGAGTGAAAAAACCCGAACTCACATGTATATATATATATATATCCTTCTGATTT 1600
cDNA(predicted) -- 685
CircularRNA GTGTAGGCATAAATGTCTACGGTTACAGAGTGAAAAAACCCGAACTCACATGTATATATATATATATATCCTTCTGATTT 448

```
                 1.620               1.640               1.660               1.680
Genomic          TAAGATGACCCAAGTGATTTAACACTCTTCCAGAAAATATCTTACAATTAGATTCTCTAGTTTGTGATTAGGTCCCACTG 1680
cDNA(predicted)  -------------------------------------------------------------------------------- 685
CircularRNA      TAAGATGACCCAAGTGATTTAACACTCTTCCAGAAAATATCTTACAATTAGATTCTCTAGTTTGTGATTAGGTCCCACTG 528

                 1.700               1.720               1.740               1.760
Genomic          CTTGAAGCTCTAGACAAATATTTGGATAAAGCTGTCAAATTTTCATGATATTCAGAAGTATATGTTCTGGTGAAATGAAA 1760
cDNA(predicted)  -------------------------------------------------------------------------------- 685
CircularRNA      CTTGAAGCTCTAGACAAATATTTGGATAAAGCTGTCAAATTTTCATGATATTCAGAAGTATATGTTCTGGTGAAATGAAA 608

                 1.780               1.800               1.820               1.840
Genomic          TGCCCAATTGATGGTGTTTCGACAGATCAGGTTAATAAGGATGTCGAGAGCAGATTCCGGCATTTGGCAAGTTCAGTACA 1840
cDNA(predicted)  -------------------------------------------------------------------------------- 685
CircularRNA      TGCCCAATTGATGGTGTTTCGACAGATCAGGTTAATAAGGATGTCGAGAGCAGATTCCGGCATTTGGCAAGTTCAGTACA 688

                 1.860               1.880               1.900               1.920
Genomic          AAATGATGTTATGCAGTTAGAGGCAGCGACTGGAGAGGACGGGGGAGGCGCGCGCATCCAAGACCTCGTTACCATTGTTA 1920
cDNA(predicted)  -------------------------------------------------------------------------------- 685
CircularRNA      AAATGATGTTATGCAGTTAGAGGCAGCGACTGGAGAGGACGGGGGAGGCGCGCGCATCCAAGACCTCGTTACCATTGTTA 768

                 1.940               1.960               1.980               2.000
Genomic          CACGGATGACGAGGAGGGCGGCGAGGGAGGCATGCGCATCAGAGACCGCATTGGTGTCATTGCAACGTATTCGACTCACA 2000
cDNA(predicted)  -------------------------------------------------------------------------------- 685
CircularRNA      CACGGATGACGAGGAGGGCGGCGAGGGAGGCATGCGCATCAGAGACCGCATTGGTGTCATTGCAACGTATTCGACTCACA 848

                 2.020               2.040               2.060               2.080
Genomic          CGCGGCGGAAGCAGGGGGGAGGCGCCGACCAGTGGCCGCTCCGGTAAATGGAAGACTGTACTACCATCGTAAAACTTTTT 2080
cDNA(predicted)  -------------------------------------------------------------------------------- 685
CircularRNA      CGCGGCGGAAGCAGGGGGGAGGCGCCGACCAGTGGCCGCTCCG------------------------------------- 891

                 2.100               2.120               2.140               2.160
Genomic          AGTCCCGCATCACATTGGTAGTAGTAATCACTAAATAGATTACCTAGAGACCTGTAGCTGTACAGTGGTAGAGGTATAGG 2160
cDNA(predicted)  -------------------------------------------------------------------------------- 685
CircularRNA      -------------------------------------------------------------------------------- 891

                 2.180               2.200               2.220               2.240
Genomic          CATAGATATCTAGGGTTACAGGGTGACAAAAAACCAACTCACATGGAGGCATCACTGGATCGCAAGTCCCCCCTAATTCG 2240
cDNA(predicted)  -------------------------------------------------------------------------------- 685
CircularRNA      -------------------------------------------------------------------------------- 891

                 2.260               2.280               2.300               2.320
Genomic          TACTTTTGGTGAAAAGGGCCATCATAACTGGTGCTAGTTATTTCCCCAGGAGTTATCCAGAAGATATCTTACAATGGGAT 2320
cDNA(predicted)  -------------------------------------------------------------------------------- 685
CircularRNA      -------------------------------------------------------------------------------- 891

                 2.340               2.360               2.380               2.400
Genomic          TATCTAGTTCGTGATTAGGTCCGACTGCTTGAAGCTATAGATAAATCTTCAACTAATGATATGGACATGACAAGTCTTAG 2400
cDNA(predicted)  -------------------------------------------------------------------------------- 685
CircularRNA      -------------------------------------------------------------------------------- 891

                 2.420               2.440               2.460               2.480
Genomic          TAGCCTACTACTGAAACTAGATGAAATAACATCCTTATAGCCCAAAGCCCAACTAATTGCTAGCTGTCCCAGCTGCGACA 2480
cDNA(predicted)  -------------------------------------------------------------------------------- 685
CircularRNA      -------------------------------------------------------------------------------- 891

                 2.500               2.520               2.540               2.560
Genomic          CCTGCAAAGGATTATATGGTTGGGTAAGCAATTGATAACCCAAGGACAATCCAATCCGATAACAGGGGAATTTTTAGTTC 2560
cDNA(predicted)  -------------------------------------------------------------------------------- 685
CircularRNA      -------------------------------------------------------------------------------- 891

                 2.580               2.600               2.620               2.640
Genomic          TAGTTCCTAGCAAATATTTAGTTCCTAGAAATTTTCAGTAATTATGGATTTGATAGATATTTTTTTAGTAATTCTATAGC 2640
cDNA(predicted)  -------------------------------------------------------------------------------- 685
CircularRNA      -------------------------------------------------------------------------------- 891

                 2.660               2.680               2.700               2.720
Genomic          ATTTCTATATGTAGTTACATTTTGTGCTTAATTATTTTATTTAACAATTTACAGATATGTCTGGAAACGACTCCGACGCA 2720
cDNA(predicted)  ------------------------------------------------------ATATGTCTGGAAACGACTCCGACGCA 711
CircularRNA      -------------------------------------------------------------------------------- 891

                 2.740               2.760               2.780               2.800
Genomic          GCCTCTTCTTCACGGACGATAAGCGCGGGCGATGAAGCTGTAGAACCGTCACCACGTCATGGCGATCCCGACGCTTTGGG 2800
cDNA(predicted)  GCCTCTTCTTCACGGACGATAAGCGCGGGCGATGAAGCTGTAGAACCGTCACCACGTCATGGCGATCCCGACGCTTTGGG 791
CircularRNA      -------------------------------------------------------------------------------- 891

                 2.820               2.840               2.860               2.880
Genomic          CACATTCATGGATGGGCAGGACGACTACACATCCACTGTTCATGATGAGTCAGGCACCGCCACAAATCCTGCAGATGCGA 2880
cDNA(predicted)  CACATTCATGGATGGGCAGGACGACTACACATCCACTGTTCATGATGAGTCAGGCACCGCCACAAATCCTGCAGATGCGA 871
CircularRNA      -------------------------------------------------------------------------------- 891

                 2.900               2.920               2.940               2.960
Genomic          CCGATCGTGACGAAGGGACCGGCCAAGGGGACCAGGAAGCTGGACAACCTAAAAAGCAACGCAAACCTAGGCGCCGAAAC 2960
cDNA(predicted)  CCGATCGTGACGAAGGGACCGGCCAAGGGGACCAGGAAGCTGGACAACCTAAAAAGCAACGCAAACCTAGGCGCCGAAAC 951
CircularRNA      -------------------------------------------------------------------------------- 891

                 2.980               3.000               3.020               3.040
Genomic          ATGCTTGGCACCAATAGGATTGTTATCAACCGAGTGTCTGAGGCGGGTCTACCTCTTAGTCCCAAGAAGGCCGAACGAGG 3040
cDNA(predicted)  ATGCTTGGCACCAATAGGATTGTTATCAACCGAGTGTCTGAGGCGGGTCTACCTCTTAGTCCCAAGAAGGCCGAACGAGG 1031
CircularRNA      -------------------------------------------------------------------------------- 891

                 3.060               3.080               3.100               3.120
Genomic          TTACAGTAATGGCCTAGGCTGCATCCTTCGTGAAACCGTGAGCATTAATGAAACCAATCTCAGGTCGAAAGCCAACGAGA 3120
cDNA(predicted)  TTACAGTAATGGCCTAGGCTGCATCCTTCGTGAAACCGTGAGCATTAATGAAACCAATCTCAGGTCGAAAGCCAACGAGA 1111
CircularRNA      -------------------------------------------------------------------------------- 891

                 3.140               3.160               3.180               3.200
Genomic          ATCTGCGAGCACTCCTCATATCGAAGTTGCACACTCATTACAAGTTCCCGGATGAGTCCCTAGACGAGACTACTCCGGTA 3200
cDNA(predicted)  ATCTGCGAGCACTCCTCATATCGAAGTTGCACACTCATTACAAGTTCCCGGATGAGTCCCTAGACGAGACTACTCCGGTA 1191
CircularRNA      -------------------------------------------------------------------------------- 891
```

```
                                   3.220               3.240               3.260               3.280
Genomic          AATAACAGAGCCCTCTGCAAGTGGTCCAAGGTTTTGAGTACATGGAAATCCAAAGCCAAAAGCGAATACCTTGAGAAAGA 3280
cDNA(predicted)  AATAACAGAGCCCTCTGCAAGTGGTCCAAGGTTTTGAGTACATGGAAATCCAAAGCCAAAAGCGAATACCTTGAGAAAGA 1271
CircularRNA      -------------------------------------------------------------------------------- 891

                                   3.300               3.320               3.340               3.360
Genomic          TTACATAACTGAGATAAAAAAGAAGTGGCCTTCGGTTTCTGAGGAGGACTGGAACCTGTTCAAGCAGCACTGCAAGACCC 3360
cDNA(predicted)  TTACATAACTGAGATAAAAAAGAAGTGGCCTTCGGTTTCTGAGGAGGACTGGAACCTGTTCAAGCAGCACTGCAAGACCC 1351
CircularRNA      -------------------------------------------------------------------------------- 891

                                   3.380               3.400               3.420               3.440
Genomic          CTGAAGTCAAGGAGATGGAAAAATGGGGGAAGGATATGCGGGCAAGGAACATTGGCAACCACACCCTTGGAAGCCGTGGT 3440
cDNA(predicted)  CTGAAGTCAAGGAGATGGAAAAATGGGGGAAGGATATGCGGGCAAGGAACATTGGCAACCACACCCTTGGAAGCCGTGGT 1431
CircularRNA      -------------------------------------------------------------------------------- 891

                                   3.460               3.480               3.500               3.520
Genomic          TACCCAGGGAAGAAGCCAAAGTGGGACAAGCAGGATGCTGAGTTTGCTGCAGCAGGCATACCAAACCCCTTCAAGGAATT 3520
cDNA(predicted)  TACCCAGGGAAGAAGCCAAAGTGGGACAAGCAGGATGCTGAGTTTGCTGCAGCAGGCATACCAAACCCCTTCAAGGAATT 1511
CircularRNA      -------------------------------------------------------------------------------- 891

                                   3.540               3.560               3.580               3.600
Genomic          TGAAAACCCGCGTGAAAATGATTACATCAGGAGCCGGTGCAAATATGATGAAGAGACTAAGACATGGGTCTTGGACGAGA 3600
cDNA(predicted)  TGAAAACCCGCGTGAAAATGATTACATCAGGAGC---------------------------------------------- 1545
CircularRNA      -------------------------------------------------------------------------------- 891

                                   3.620               3.640               3.660               3.680
Genomic          AAACGATGAAAGTCAAGGAACTCCTGGTAATTGATTAACCTGCTTATTTTTTGTTATGCTAATTAATTAGATTTCTTTCC 3680
cDNA(predicted)  -------------------------------------------------------------------------------- 1545
CircularRNA      -------------------------------------------------------------------------------- 891

                                   3.700               3.720               3.740               3.760
Genomic          AAGTAGTCACACTTCTAAATGGTTACCTCCCTATTGTAGAGGCAGTATCATGTGGAATCTCAAAGCTCCCAGGAGTCGGA 3760
cDNA(predicted)  -------------------------------------------------------------------------------- 1545
CircularRNA      -------------------------------------------------------------------------------- 891
```

Unknown_circular RNA (ID: Ch2:476367671-476368169)

<u>G</u>CATAGGGATCAAGGTCCAAGACAGTGGAGAACTGCAGATCCCTATGCTGTTAGGAGGAGAA
TAGTTGTTCATCCTCGCTTTCACAACAAGGAGCAACAAGATTTTTACGAGATAGTGTTATTGGAC
AAGAGTTTTGCAGTCATTGACATGAGATATGTGGACTGGGAGTACATCAAGGAACATGAGGATTT
CTTCTTTCATGTGAGAGAGAACTTCAGGGCAATTGATACTGAAGATTTTGTTGGCAAAGAGATGA
CTGCCTGGAATGATTAGATGATCATGCAATTCTACTCCACTACACATTTTTATCCAAATGGTAGGA
TTGCATGGATGACTGAAGATCACGGGTATGAATCAACTGTGAAGAAGTGGGCTACGATCATTGG
TGCTCCTAAAGCCCAAGAGGGGACTTGGATGTGTACTCTGAGCCTAAAATGAATCACAACACTA
TGGCT**AATATGTACAATTCAGTGCCCCATCAGTATTTGAAGACTCAC<u>A</u>**

The nucleotides of junction-region are underlined. The nucleotides of junction-region which are supported by the junction-spaning sequencing reads are shown in red. Introns are not shown if the absence is supported by sequencing reads. In the absence of supporting sequencing reads, the intronic nucletides are shown as N.

Structural relationship between the circular RNA and its parental gene

↓

```
                 20                  40                  60                  80
Genomic          GGACTCAGGCACTAGCCCTGAGAGGACAGCTTCGGACCCAGATACTCCAAGCTCCCGTTATATGCCCAAGGCATCTATGA 80
cDNA(predicted)  -------------------------------------------------------------------------------- -
CircularRNA      -------------------------------------------------------------------------------- -

                 100                 120                 140                 160
Genomic          GGTCAAGGAAGAAGCTTAATTCTGATGAGGAGGATGCTGACTTCATTCCAGAAGAAAGTATTCCCCAAAAGAAAAAGGAG 160
cDNA(predicted)  ---------------------------------ATGCTGACTTCATTCCAGAAGAAAGTATTCCCCAAAAGAAAAAGGAG 47
CircularRNA      -------------------------------------------------------------------------------- -

                 180                 200                 220                 240
Genomic          AGTCACAGTCAAGAAAACTGTGAGAAAGGAGTATGCCGGGACTGATTCTATGAGAGCTCCAGGGACGGCTCAGCAAGGGA 240
cDNA(predicted)  AGTCACAGTCAAGAAAACTGTGAGAAAGGAGTATGCCGGGACTGATTCTATGAGAGCTCCAGGGACGGCTCAGCAAGGGA 127
CircularRNA      -------------------------------------------------------------------------------- -

                 260                 280                 300                 320
Genomic          TGAAACTCACCCTTGAAAACCCATATTCCAAGCAGGCTAAGAAGCCAGGGCAGAAGAGAGCCAGGAAGAGAGTTGTGCAC 320
cDNA(predicted)  TGAAACTCACCCTTGAAAACCCATATTCCAAGCAGGCTAAGAAGCCAGGGCAGAAGAGAGCCAGGAAGAGAGTTGTGCAC 207
CircularRNA      -------------------------------------------------------------------------------- -

                 340                 360                 380                 400
Genomic          GTTGTTGGAAGGCCAACTTCCATGTATGAAGATCCAGCAGAAGCTGCTAAGGAAGAAGAGGAAGAGGAAACTGTCAATCC 400
cDNA(predicted)  GTTGTTGGAAGGCCAACTTCCATGTATGAAGATCCAGCAGAAGCTGCTAAGGAAGAAGAGGAAGAGGAAACTGTCAATCC 287
CircularRNA      -------------------------------------------------------------------------------- -

                 420                 440                 460                 480
Genomic          ACCTCCTTCCAATAAGCTTTCGGCAGATGCTATGCCAAGCAGGTCTGCATCCAAGACAAAGTCTTCTGCTCCTAAGTCTA 480
cDNA(predicted)  ACCTCCTTCCAATAAGCTTTCGGCAGATGCTATGCCAAGCAGGTCTGCATCCAAGACAAAGTCTTCTGCTCCTAAGTCTA 367
CircularRNA      -------------------------------------------------------------------------------- -

                 500                 520                 540                 560
Genomic          AGAAGCTAGTAGCTCCCAAGAACACTACTAGGGATATACTTGCAGCAGAGAAGAACAAGGGCTCTGCATCCAGATATGTT 560
cDNA(predicted)  AGAAGCTAGTAGCTCCCAAGAACACTACTAGGGATATACTTGCAGCAGAGAAGAACAAGGGCTCTGCATCCAGATATGTT 447
CircularRNA      -------------------------------------------------------------------------------- -

                 580                 600                 620                 640
Genomic          GTTGCGGAGGAGGAAAAAGATATTTCTGTGCTGAGGAAGTTGAGAGCTCATTTGCCACTGCACAATGATGCTCATTCAGT 640
cDNA(predicted)  GTTGCGGAGGAGGAAAAAGATATTTCTGTGCTGAGGAAGTTGAGAGCTCATTTGCCACTGCACAATGATGCTCATTCAGT 527
CircularRNA      -------------------------------------------------------------------------------- -

                 660                 680                 700                 720
Genomic          TGTTGAGGACATAAAGAAGAGAAGGGATCAAGGTCCAAGACAGTGGAGAACTGCAGATCCCTATGCTGTTAGGAGGAGAA 720
cDNA(predicted)  TGTTGAGGACATAAAGAAGAGAAGGGATCAAGGTCCAAGACAGTGGAGAACTGCAGATCCCTATGCTGTTAGGAGGAGAA 607
CircularRNA      ------------------GCATAGGGATCAAGGTCCAAGACAGTGGAGAACTGCAGATCCCTATGCTGTTAGGAGGAGAA 62

                 740                 760                 780                 800
Genomic          TAGTTGTTCATCCTCGCTTTCACAACAAGGAGCAACAAGATTTTTACGAGATAGTGTTATTGGACAAGAGTTTTGCAGTC 800
cDNA(predicted)  TAGTTGTTCATCCTCGCTTTCACAACAAGGAGCAACAAGATTTTTACGAGATAGTGTTATTGGACAAGAGTTTTGCAGTC 687
CircularRNA      TAGTTGTTCATCCTCGCTTTCACAACAAGGAGCAACAAGATTTTTACGAGATAGTGTTATTGGACAAGAGTTTTGCAGTC 142

                 820                 840                 860                 880
Genomic          ATTGACATGAGATATGTGGACTGGGAGTACATCAAGGAACATGAGGATTTCTTCTTTCATGTGAGAGAGAACTTCAGGGC 880
cDNA(predicted)  ATTGACATGAGATATGTGGACTGGGAGTACATCAAGGAACATGAGGATTTCTTCTTTCAT-------------------- 747
CircularRNA      ATTGACATGAGATATGTGGACTGGGAGTACATCAAGGAACATGAGGATTTCTTCTTTCATGTGAGAGAGAACTTCAGGGC 222

                 900                 920                 940                 960
Genomic          AATTGATACTGAAGATTTTGTTGGCAAAGAGATGACTGCCTGGAATGATTAGATGATCATGCAATTCTACTCCACTACAC 960
cDNA(predicted)  -------------------------------------------------------------------------------- 747
CircularRNA      AATTGATACTGAAGATTTTGTTGGCAAAGAGATGACTGCCTGGAATGATTAGATGATCATGCAATTCTACTCCACTACAC 302

                 980                 1.000               1.020               1.040
Genomic          ATTTTTATCCAAATGGTAGGATTGCATGGATGACTGAAGATCACGGGTATGAATCAACTGTGAAGAAGTGGGCTACGATC 1040
cDNA(predicted)  -------------------------------------------------------------------------------- 747
CircularRNA      ATTTTTATCCAAATGGTAGGATTGCATGGATGACTGAAGATCACGGGTATGAATCAACTGTGAAGAAGTGGGCTACGATC 382

                 1.060               1.080               1.100               1.120
Genomic          ATTGGTGCTCCTAAAGCCCAAGAGGGGACTTGGATGTGTACTCTGAGCCTAAAATGAATCACAACACTATGGCTAATATG 1120
cDNA(predicted)  -------------------------------------------------------------------------------- 747
CircularRNA      ATTGGTGCTCCTAAAGCCCAAGAGGGGACTTGGATGTGTACTCTGAGCCTAAAATGAATCACAACACTATGGCTAATATG 462

                 1.140               1.160               1.180               1.200
Genomic          TACAATTCAGTGCCCCATCAGTATTTGAAGACTCACAAGTTTGGCTCCGTCTACTTTCTACAAGCAAGGATACCCACTAC 1200
cDNA(predicted)  -------------------------------------------------------------------------------- 747
CircularRNA      TACAATTCAGTGCCCCATCAGTATTTGAAGACTCACA------------------------------------------- 499

                 1.220               1.240               1.260               1.280
Genomic          CAACAACATTATGAGGTACACCCTGATGCTCAAGTCAGGAAATGAAACGATGATCAGAGGGTATTCCATCAATATGTTGC 1280
cDNA(predicted)  -------------------------------------------------------------------------------- 747
CircularRNA      -------------------------------------------------------------------------------- 499

                 1.300               1.320               1.340               1.360
Genomic          ACCACGTTGATTCTCACACAAGGATTAGAGTGATGGATCTGGTAGTTGAGACGGTCAGGAGGACAGCTGCTGATCAGAAG 1360
cDNA(predicted)  -------------------------------------------------------------------------------- 747
CircularRNA      -------------------------------------------------------------------------------- 499

                 1.380               1.400               1.420               1.440
Genomic          AGATCATGTGGATATGCTCCGTACATTCAGATGCTCATCAATGCCAGATTGGGAAGCATACATATCAACTTGATCATCAC 1440
cDNA(predicted)  -------------------------------------------------------------------------------- 747
CircularRNA      -------------------------------------------------------------------------------- 499

                 1.460               1.480               1.500               1.520
Genomic          ACCTGTCACTTCAGCCAAAGTTCGAAGACAATGAGGCAATGATGGACCCCAACCATCCCAGCTCAACTACAGCTCGTCAG 1520
cDNA(predicted)  --------------CCAAAGTTCGAAGACAATGAGGCAATGATGGACCCCAACCATCCCAGCTCAACTACAGCTCGTCAG 813
CircularRNA      -------------------------------------------------------------------------------- 499

                 1.540               1.560               1.580               1.600
Genomic          CAAGCAAAGGCAGAGGCAGCCAGAGCAGCTCCAACACCACAGTTGAGATCCAGAGAGGAGCATATGTCATTTCTTGTCAA 1600
cDNA(predicted)  CAAGCAAAGGCAGAGGCAGCCAGAGCAGCTCCAACACCACAGTTGAGATCCAGAGAGGAGCATATGTCATTTCTTGTCAA 893
CircularRNA      -------------------------------------------------------------------------------- 499
```

```
                                     1.620               1.640               1.660               1.680
         Genomic  CACCATCCAAGGTATGGAAAAGAATATTTCTGAGATTCTGCTGAACCAGAAGAGCCTTGAGAGGATCGTGGAGACTAAAT 1680
cDNA(predicted)   CACCATCCAAGGTATGGAAAAGAATATTTCTGAGATTCTGCTGAACCAGAAGAGCCTTGAGAGGATCGTGGAGACTAAAT 973
     CircularRNA  -------------------------------------------------------------------------------- 499

                                     1.700               1.720               1.740               1.760
         Genomic  CCCATGACCTGGATATCAAAGTAATGGAGTTGGCTACCACAATTGAGCAGCTCAAACATGAAGTTGATGCAATGCACACA 1760
cDNA(predicted)   CCCATGACCTGGATATCAAAGTAATGGAGTTGGCTACCACAATTGAGCAGCTCAAACATGAAGTTGATGCAATGCACACA 1053
     CircularRNA  -------------------------------------------------------------------------------- 499

                                     1.780               1.800               1.820               1.840
         Genomic  CCTCGTTCTAGCAATGATGATGATGATACGTCTCTTCCTACAACTACTCAATTCAGGACTCATACTAGGTCTGCAACTAT 1840
cDNA(predicted)   CCTCGTTCTAGCAATGATGATGATGATACGTCTCTTCCTACAACTACTCAATTCAGGACTCATACTAG------------ 1121
     CircularRNA  -------------------------------------------------------------------------------- 499

                                     1.860               1.880               1.900               1.920
         Genomic  GCCAGTTTCGGAAGCTAGACCTACTTCGTCAGCCCATGCATCAGCTCCACCAGCTCCTGCACNNNNNNNNNNNACACCTC 1920
cDNA(predicted)   -------------------------------------------------------------------------------- 1121
     CircularRNA  -------------------------------------------------------------------------------- 499

                                     1.940               1.960               1.980               2.000
         Genomic  CACCTCAGAGGACATCCGCCGAAGCCTTCGCCGACACTCTCATGTCGACTCCATCAACTGCTACCGGAGGAGCTCGAGCC 2000
cDNA(predicted)   --------AGGACATCCGCCGAAGCCTTCGCCGACACTCTCATGTCGACTCCATCAACTGCTACCGGAGGAGCTCGAGCC 1193
     CircularRNA  -------------------------------------------------------------------------------- 499

                                     2.020
         Genomic  CAGAGCACGTTTAGTTCCAGACCTTTGAACTTTTTG 2036
cDNA(predicted)   CAGAGCACGTTTAGTTCCAGACCTTTGA-------- 1221
     CircularRNA  ------------------------------------ 499
```

Probable long non-coding RNA_circular RNA (ID: Ch2:605441109-605441443)

<u>C</u>AGACATTGTTTACCAGTAGACTTCTGGTGATGTTTGGACTGTTCGGTTAAAATCAAGGCCATC
AGCAAGAACATAGAGGGAAAACAAGCATAAACTTGGATTGGAAGGGAATGATTTTTATCATTTTT
CTAGAGACCAAATTGTTCAATTCAACTCACTATACAGAGGATAAAATCACTGTTCTGACCCTTTTA
GCAATCGTTGGCACAAAATGAACTCGACATGAAAGTATTTCACGATCTGACCCTTTTCGCAACAC
CACAGTCCGTGGCGTTTCTGCTTAACATAGAAACGCG**ACCTAGATCGGCGTATGTAAGTGCCG**
CTGCAACGCTA<u>G</u>

The nucleotides of junction-region are underlined. The nucleotides of junction-region which are supported by the junction-spaning sequencing reads are shown in red. Introns are not shown if the absence is supported by sequencing reads. In the absence of supporting sequencing reads, the intronic nucletides are shown as N.

Structural relationship between the circular RNA and its parental gene

```
                                                                                20                  40                  60                  80
Genomic      CATCGTGTTTTTATCATTTTTCCAGAACCCAAATTGTTCAATCAAACCCGCTATACAAAGCAAATAAAATCAGACATTGT 80
CircularRNA  -------------------------------------------------------------------------------- -

                                                                               100                 120                 140                 160
Genomic      TTACCAGTAGACTTCTGGTGATGTTTGGATTGTTTGGTTAAAATCAAGGCCATCATGTTTTTTAACATATTTCCAGAACC 160
CircularRNA  -------------------------------------------------------------------------------- -

                                                                               180                 200                 220                 240
Genomic      CAAATTGTTCAATCAAATCCGCTATACAAAGCAAACAAAACCAGACATTGTTTACCAGTAGACTTCTGGTGATGTTTGGA 240
CircularRNA  -----------------------------------------CAGACATTGTTTACCAGTAGACTTCTGGTGATGTTTGGA 39

                                                                               260                 280                 300                 320
Genomic      CTGTTTGGTTAAAATCAAGGCCATCAGCAAGAACATAGAGGGAAAACAAGCATAAACTTGGATTGGAAGGGAATGATTTT 320
CircularRNA  CTGTTCGGTTAAAATCAAGGCCATCAGCAAGAACATAGAGGGAAAACAAGCATAAACTTGGATTGGAAGGGAATGATTTT 119

                                                                               340                 360                 380                 400
Genomic      TATCCTTTTTCTAGAGACCAAATTGTTCAATTCAACTCACTATACAGAGGATAAAATCACTGTTCTGACCCTTTTAGCAA 400
CircularRNA  TATCATTTTTCTAGAGACCAAATTGTTCAATTCAACTCACTATACAGAGGATAAAATCACTGTTCTGACCCTTTTAGCAA 199

                                                                               420                 440                 460                 480
Genomic      TCGTTGGCACAAAATGAACTCGACATGAAAGTATTTCACGATCTGACCCTTTTCGCAACACCACAGTCCGTGGCGTTTCT 480
CircularRNA  TCGTTGGCACAAAATGAACTCGACATGAAAGTATTTCACGATCTGACCCTTTTCGCAACACCACAGTCCGTGGCGTTTCT 279

                                                                               500                 520                 540                 560
Genomic      GCTTAACATAGAAACGCGACCTAGATCGGCGTATGTAAGTGCCGCTGCAACGCTAGCTGGCTCGGCGTTTCTAAGTTAAG 560
CircularRNA  GCTTAACATAGAAACGCGACCTAGATCGGCGTATGTAAGTGCCGCTGCAACGCTAG------------------------ 335

                                                                               580                 600                 620                 640
Genomic      CGGAAACGCCACGGACTGTGGCGTTGCGAAAAGGGTCAAATCATGAAATACTTTCATGTCGAGTTCATTTTGTGCCAACG 640
CircularRNA  -------------------------------------------------------------------------------- 335

                                                                               660                 680                 700                 720
Genomic      ATTGCCAAAAGGGTCAGAACAGTGATTTTATCCCTATACAGAGCCAACAAAACCCAACATTGTTTATCAGTAGACTTCTG 720
CircularRNA  -------------------------------------------------------------------------------- 335
```

Unknown_circular RNA (ID: Ch3:100501939-100502294**)**

<u>**T**</u>**GAGAGAACACACAGTGACGCAGATGATTGGGCGACTGCTGCAGGT**GGACGAACACCATCGT
CGTGGATGATATGGGGCATGAGAGGAGTAGCTTGGACAGTGCCCGCGTTAGGGACGACACGG
CAAGTGTTGAGAAATGTATCCAAGAGCAAATGTTGTATTACCAACAACCGCGACAAGATAGGAC
GTACCTGATGCACGCTTTGTCCGGTGTCGTTGCCAAGCTGTGAGAGGAGTTCATAGCCACACTC
TACATCTATTAGAGTACTGCCACCAACCCTTCATGGGCCAGTCGTGCACTGATGCGCTCAATC**A**
CAAGGCGGTGGTGGTCCGCGGCGTGTCGTCCACATCAAA<u>**C**</u>

The nucleotides of junction-region are underlined. The nucleotides of junction-region which are supported by the junction-spaning sequencing reads are shown in red. Introns are not shown if the absence is supported by sequencing reads. In the absence of supporting sequencing reads, the intronic nucletides are shown as N.

Structural relationship between the circular RNA and its parental gene

↓

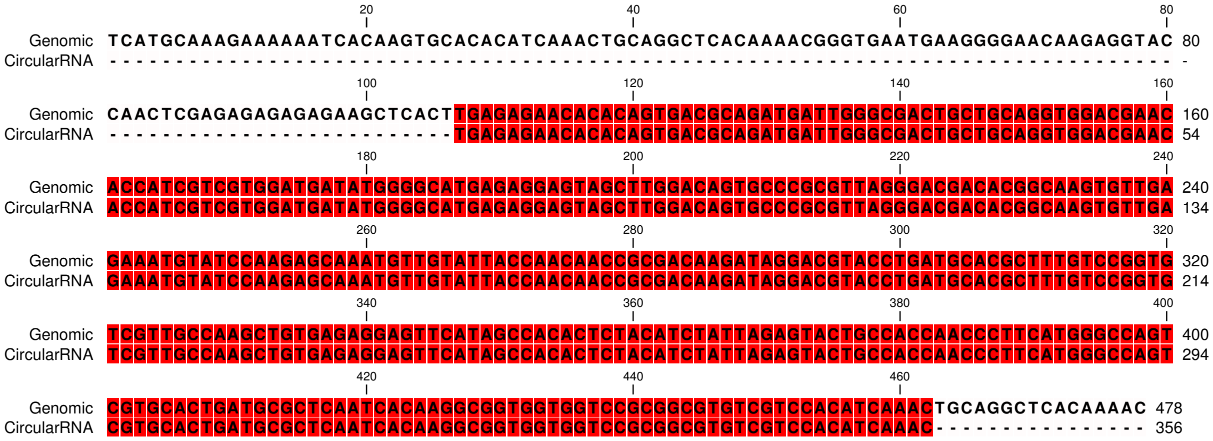
20 40 60 80
Genomic TCATGCAAAGAAAAAATCACAAGTGCACACATCAAACTGCAGGCTCACAAAACGGGTGAATGAAGGGGAACAAGAGGTAC 80
CircularRNA -- -
100 120 140 160
Genomic CAACTCGAGAGAGAGAGAAGCTCACTTGAGAGAACACACAGTGACGCAGATGATTGGGCGACTGCTGCAGGTGGACGAAC 160
CircularRNA --------------------------TGAGAGAACACACAGTGACGCAGATGATTGGGCGACTGCTGCAGGTGGACGAAC 54
180 200 220 240
Genomic ACCATCGTCGTGGATGATATGGGGCATGAGAGGAGTAGCTTGGACAGTGCCCGCGTTAGGGACGACACGGCAAGTGTTGA 240
CircularRNA ACCATCGTCGTGGATGATATGGGGCATGAGAGGAGTAGCTTGGACAGTGCCCGCGTTAGGGACGACACGGCAAGTGTTGA 134
260 280 300 320
Genomic GAAATGTATCCAAGAGCAAATGTTGTATTACCAACAACCGCGACAAGATAGGACGTACCTGATGCACGCTTTGTCCGGTG 320
CircularRNA GAAATGTATCCAAGAGCAAATGTTGTATTACCAACAACCGCGACAAGATAGGACGTACCTGATGCACGCTTTGTCCGGTG 214
340 360 380 400
Genomic TCGTTGCCAAGCTGTGAGAGGAGTTCATAGCCACACTCTACATCTATTAGAGTACTGCCACCAACCCTTCATGGGCCAGT 400
CircularRNA TCGTTGCCAAGCTGTGAGAGGAGTTCATAGCCACACTCTACATCTATTAGAGTACTGCCACCAACCCTTCATGGGCCAGT 294
420 440 460
Genomic CGTGCACTGATGCGCTCAATCACAAGGCGGTGGTGGTCCGCGGCGTGTCGTCCACATCAAACTGCAGGCTCACAAAAC 478
CircularRNA CGTGCACTGATGCGCTCAATCACAAGGCGGTGGTGGTCCGCGGCGTGTCGTCCACATCAAAC---------------- 356

Probable long non-coding RNA_circular RNA (ID: Ch4:247058951-247059280**)**

<u>A</u>GGAGAGGAGATCAGATGTATGGAAACAAAAGAAAAGGCTGCAAGTCATGGTTGTTTATACGA
GAGGAGATGGTGGACCTGCAGACACCTTTGATTTCGTTAAATCCTGTGGAGGGAAGGGGAGATT
ATGGATGGATGGAGCTTGGTTGAAATTGGGTTGCTGTTGCATTGGCATTTAAGACTCAAGAGAAT
GTTCCTTCCCAAAGAAGAGGTCGGGCGTTCTCGTTGGCGACTCTAGCGGTTGAATTCATCAGCT
TATCTAGATTAGCTAGTGTTGGTGTTGCTATTT**ATGGATTTGTGGTGAAGAGGAACAACCATCC
TGGTTGCTG<u>C</u>**

The nucleotides of junction-region are underlined. The nucleotides of junction-region which are supported by the junction-spaning sequencing reads are shown in red. Introns are not shown if the absence is supported by sequencing reads. In the absence of supporting sequencing reads, the intronic nucletides are shown as N.

Structural relationship between the circular RNA and its parental gene

↓

```
                   20                  40                  60                  80
Genomic            TTAATAAATGAGGTTATATATGATACTAGTTTATGATACTATGCATTGTGAAGGTAGTATCATACAATAGTATCATATCC 80
cDNA(AK355915)     -------------------------------------------------------------------------------- -
CircularRNA        -------------------------------------------------------------------------------- -
                   100                 120                 140                 160
Genomic            ATGATACTACTATATGATACTCCCCACTATGACCAGCCTAATTAGTCACTATGTCACATACATGCGGGACCCACTGGTTA 160
cDNA(AK355915)     -------------------------------------------------------------------------------- -
CircularRNA        -------------------------------------------------------------------------------- -
                   180                 200                 220                 240
Genomic            GATATTACTTGGACCACCCCCTTTGACACGTGACATATTCCCGACGCAAGGCTGACCTAGTACTATTTCCTGGAATGAAA 240
cDNA(AK355915)     -------------------------------------------------------------------------------- -
CircularRNA        -------------------------------------------------------------------------------- -
                   260                 280                 300                 320
Genomic            ATAAATTATAAATGCTTTATTATTTTCAGAAAAGTGTTTAAAACTTATAAAATTCATATAATAATATATAACTCAGATTG 320
cDNA(AK355915)     -------------------------------------------------------------------------------- -
CircularRNA        -------------------------------------------------------------------------------- -
                   340                 360                 380                 400
Genomic            AAATAATTTATATATGAAAAATGAACCGAAAAATGCAAGGAATATGTTTATGGCATTTTCATGCATGATAGAACAACTTA 400
cDNA(AK355915)     -------------------------------------------------------------------------------- -
CircularRNA        -------------------------------------------------------------------------------- -
                   420                 440                 460                 480
Genomic            TTTCTTGTGTTTAGGAGAAATCATATAAATGACATTTAAATAACCACATATGAAGTTTGAATTTGAATCTTTGGTTCAAA 480
cDNA(AK355915)     -------------------------------------------------------------------------------- -
CircularRNA        -------------------------------------------------------------------------------- -
                   500                 520                 540                 560
Genomic            CAAACTTCATTTAACATGCATTAGATAAATCAACAACATGTTTCCACATCATTTTCATGCATCAAAGTGTTGCATTTCCG 560
cDNA(AK355915)     -------------------------------------------------------------------------------- -
CircularRNA        -------------------------------------------------------------------------------- -
                   580                 600                 620                 640
Genomic            TGAATTAAATGTTGTTCCTTCTTTGTTTGCTGGTGTTGTTCCCTCTCGGTAGTTATTATTCCGATGTTGAGATTGATGTT 640
cDNA(AK355915)     -------------------------------------------------------------------------------- -
CircularRNA        -------------------------------------------------------------------------------- -
                   660                 680                 700                 720
Genomic            GCTGATGAAGAACAGTTAATCTTCAGAAGTGCCAGGCAAGCAACCCTCTTAAGCATACTGATATAAACTCACTCTCTCGC 720
cDNA(AK355915)     -------------------------------------------------------------------------------- -
CircularRNA        -------------------------------------------------------------------------------- -
                   740                 760                 780                 800
Genomic            TCCTCATCTCTTTTACTGCATTAGGAGAATAACAATTCAACTATTACATGTTCTCGATAGTTGAACCCATTCGCATGAAA 800
cDNA(AK355915)     -------------------------------------------------------------------------------- -
CircularRNA        -------------------------------------------------------------------------------- -
                   820                 840                 860                 880
Genomic            TGTAATTGTCACAATAAATAGTCGAAAGAACTTAGCATGAGTAGCAACTACTTGAGCCTTGTTACGCTTACTCATCAATG 880
cDNA(AK355915)     -------------------------------------------------------------------------------- -
CircularRNA        -------------------------------------------------------------------------------- -
                   900                 920                 940                 960
Genomic            CTTCCTTACTATGCGTGATATGCTTATAGTTTTGTCAGGTCTGATTCATCTAGGCGATGAATAAGAATGGATGTGTTCAT 960
cDNA(AK355915)     -------------------------------------------------------------------------------- -
CircularRNA        -------------------------------------------------------------------------------- -
                   980                 1.000               1.020               1.040
Genomic            GTCCTATTGCGTGAGAGATAAGTGTGTGAATCCGATTTGATAAAGGTAACGATGAGAGGCCATGTAGGTGTATATGGTGG 1040
cDNA(AK355915)     -------------------------------------------------------------------------------- -
CircularRNA        -------------------------------------------------------------------------------- -
                   1.060               1.080               1.100               1.120
Genomic            GCTGTCTCATTGGGGTCGTCCTTAAGAACTGAGTTATGTGTATATTACCCAAAATGAGATACTACCACACATTAGGATAC 1120
cDNA(AK355915)     -------------------------------------------------------------------------------- -
CircularRNA        -------------------------------------------------------------------------------- -
                   1.140               1.160               1.180               1.200
Genomic            TTAATTGACCCTCTCGGCTTATTAATTGCTTTGATCTCTATCCACCAGTTACAACTAGTTTCTGGTGTTCGTAGGAAAAG 1200
cDNA(AK355915)     -------------------------------------------------------------------------------- -
CircularRNA        -------------------------------------------------------------------------------- -
                   1.220               1.240               1.260               1.280
Genomic            TGTTGGCGGTCGTGTCTAGTGCTGCCCAACGGGGTTGGCTTCAGTACGGTAGATGCATAATGGCATAGTGTACCAAGTGG 1280
cDNA(AK355915)     -------------------------------------------------------------------------------- -
CircularRNA        -------------------------------------------------------------------------------- -
                   1.300               1.320               1.340               1.360
Genomic            CACCCAATGGTGGGCTTGTGGAACCTTGCATACATTTTTGGGCCCTTATAGAAAACTTCGTCCGGACTTCCTTGCGGGTT 1360
cDNA(AK355915)     -------------------------------------------------------------------------------- -
CircularRNA        -------------------------------------------------------------------------------- -
                   1.380               1.400               1.420               1.440
Genomic            CAATCTCCGATAGGCGATTAACATGGACTAGAGTCTTGTGTGGTTAACGACTGTGGACGGCACCCATGCAAGGCTTTCGA 1440
cDNA(AK355915)     -------------------------------------------------------------------------------- -
CircularRNA        -------------------------------------------------------------------------------- -
                   1.460               1.480               1.500               1.520
Genomic            TTGAAGGTTGACGATTACATGATGTGCATGTGGTGGTAAGTGGTGAGATTGTGTGTGTGAAGAAGTATACCCCTACAAGG 1520
cDNA(AK355915)     -------------------------------------------------------------------------------- -
CircularRNA        -------------------------------------------------------------------------------- -
                   1.540               1.560               1.580               1.600
Genomic            TTAACTAATTTATTCGAATAGCCATGTCCGCATATATGGACTACTTGGAAAGCCTATATGGTTCAGAGATAACTTGAATT 1600
cDNA(AK355915)     -------------------------------------------------------------------------------- -
CircularRNA        -------------------------------------------------------------------------------- -
```

```
Genomic          GGATACTCTAAGATGCGCAAGACAAGTGTGAGTGCTATGGATGTCCTTCTCCTTGGGAGACGGGAGAGGATCTGTAGTTA 1680
cDNA(AK355915)   -------------------------------------------------------------------------------- -
CircularRNA      -------------------------------------------------------------------------------- -

Genomic          TCTATTGATATGGTGAATATGTAGACTTGAGTGCGCTCTCTCACCCCAATGGAAGTACTTGTAGTCCTAGTACAGGTTAG 1760
cDNA(AK355915)   -------------------------------------------------------------------------------- -
CircularRNA      -------------------------------------------------------------------------------- -

Genomic          CGAGGATTCAAAGCTGTCTTGCTGTAGTAAACCCCACAATCCCCTTGTTGGTGATGATGCATATGTAGATAGTTCTCATG 1840
cDNA(AK355915)   -------------------------------------------------------------------------------- -
CircularRNA      -------------------------------------------------------------------------------- -

Genomic          TAAATCTTGCTCAGTACCTTTGTACTCATGGTTGCTTTATTTTGTTTTTGTAGAGGAGACCCCAGTCTCAGTAGTGGTTC 1920
cDNA(AK355915)   -------------------------------------------------------------------------------- -
CircularRNA      -------------------------------------------------------------------------------- -

Genomic          CTACGATGGGTTTGATGTTGACCGGATTAGCTTGAGAATCCTAGATAGAGGTCTAGCTCCTTCGATATATGGTCATTGTA 2000
cDNA(AK355915)   -------------------------------------------------------------------------------- -
CircularRNA      -------------------------------------------------------------------------------- -

Genomic          GATTTTGACGCTCCTCTAGCCACTTTTAGTAGATCTATGTATCCTGACATCACTACAGGAATCATGTAATTTGCCGTCTG 2080
cDNA(AK355915)   -------------------------------------------------------------------------------- -
CircularRNA      -------------------------------------------------------------------------------- -

Genomic          TAGTGTTTTTGCCTGTCAGGTTTTCGTCGGGACAGACGACAAAGAGCATTTTTATCGTCAACTGCTGACGGTAAAGAATG 2160
cDNA(AK355915)   -------------------------------------------------------------------------------- -
CircularRNA      -------------------------------------------------------------------------------- -

Genomic          ACTGGCGGTGAAAACACACTTTGCCGTATTCCTTTTTATTATAGACGGCAACATATATCTTTGTCGTCTGCATTTTTCAC 2240
cDNA(AK355915)   -------------------------------------------------------------------------------- -
CircularRNA      -------------------------------------------------------------------------------- -

Genomic          TATAGACGGCAAAATTGTTCTTTGCCGTCTGTAATGAAAAATACAGACGGCAAAGACATATTTTGCCGTCTGTAATAAAA 2320
cDNA(AK355915)   -------------------------------------------------------------------------------- -
CircularRNA      -------------------------------------------------------------------------------- -

Genomic          GGCAGGCAGCAAAGTGTGTTTTTTGCCGTCTTTGATAAAAACGCAGTCGGCAAAAAAAGAAGCACACCACGCGGATCGAT 2400
cDNA(AK355915)   -------------------------------------------------------------------------------- -
CircularRNA      -------------------------------------------------------------------------------- -

Genomic          CAGGTGGATCGTTGACGTGGCGCAATCCCAATCATTCATTTGATGTTGGACGGGTCTAAGCGGGCTGAAGTTTTGTCGTC 2480
cDNA(AK355915)   -------------------------------------------------------------------------------- -
CircularRNA      -------------------------------------------------------------------------------- -

Genomic          TTCCCTTGCCAAGCTAAGGACAAATTGCAGTGTCCTCACCATGCTCTCCCGCATCTCTCGGCTCGTCCGCCTCCTCACTC 2560
cDNA(AK355915)   ----------------------------------------------------------AGGCTCGTCCGCCTCCTCACTC 22
CircularRNA      -------------------------------------------------------------------------------- -

Genomic          GTGTGATCCAATCCCCATCCACCCACCGTCGCCACAAGGCTCCGCCGGCCACCCACCCTTTCCCCCTCCCCTCCCCCTCC 2640
cDNA(AK355915)   GTGTGATCCAATCCCCATCCACCCACCGTCGCCACAAGGCTCCGCCGGCCACCCACCCTTTCCCCCTCCCCTCACCCTCC 102
CircularRNA      -------------------------------------------------------------------------------- -

Genomic          CCCTCCCCTCGCCTTCCTCCCTACCCAGGCCGCAGATCCAATAGTCCATGTTCTTTTCCCTTCCTCCTTCCTTCTCTGTT 2720
cDNA(AK355915)   CCCTCCCCTCGCCTTCCTCCCTACCCAGGCCGCAGATCCAATAGTCCATGTTCTTTTCCCTTCCTCCTTCCTTCTCTGTT 182
CircularRNA      -------------------------------------------------------------------------------- -

Genomic          AGGGTTAGGGTTTGCGGTCGGAGGGCCCGGCCGGCCGGTGATGGACGCCGGCGACGGAGGTCTCCTCTCCACGGATCAGT 2800
cDNA(AK355915)   AGGGTTAGGGTTTGCGGTCGGAGGGCCCGGCCGGCCGGTGGTGGACGCCGGCGACGGAGGTCTCCTCTCCACGGATCAGT 262
CircularRNA      -------------------------------------------------------------------------------- -

Genomic          GATGGAGTGCGTGCTTATTTTGCTCTCAATGGATCAAGGTGCCTTCTCACTCCCATCCCTCCTCTCCCTAGGATTCGATT 2880
cDNA(AK355915)   GATGGAGTGCGTGCTTATTTTGCTCTCAATGGATCAAGGTGCCTTCTCACTCCCATCCCTCCTCTCCCTAGGATTCGATT 342
CircularRNA      -------------------------------------------------------------------------------- -

Genomic          CGTTATCCTCACGCCCTGTTCCTCTTTTCATGTTCGTGCTGCAAGCATATCCACTGGATCCATGCGGTGTTGGAGGAGAT 2960
cDNA(AK355915)   CGTTATCCTCACGCCCTGTTCCTCTTTTCATGTTCGTGCTGCAAGCATATCCACTGGATCCATGCGGTGTTGGAGGAGAT 422
CircularRNA      -------------------------------------------------------------------------------- -

Genomic          GATGCGGTTCGTGTACTCCCTTCTGTGTGTATGTGTAGTTGGTAAGCCACCCTGGCTCTCCCCTCTCTTCCCATTTGCTC 3040
cDNA(AK355915)   GATGCGGTTCGTGTACTCCCTTCTGTGTGTATGTGTAGTTGGT------------------------------------- 465
CircularRNA      -------------------------------------------------------------------------------- -

Genomic          TATCTCTCTCTGACCCGTGTCTCATCTTGTCTTTGTAGGTGCAATAGCTAGAGTGGCCAGAGGAGGTGGAGGAGCTGGCC 3120
cDNA(AK355915)   ----------------------------------------GCAATAGCTAGAGTGGCCAGAGGAGGTGGAGGAGCTGGCC 505
CircularRNA      -------------------------------------------------------------------------------- -

Genomic          AGCACCCATCGCCTGGACATAAGGTCAGCTTCCCCTCTCCCCCATGGCCCCATCGACCCCCATCACACATCTGAATATAT 3200
cDNA(AK355915)   AGCACCCATCGCCTGGACATAAG--------------------------------------------------------- 528
CircularRNA      -------------------------------------------------------------------------------- -
```

```
                                       3.220                3.240                3.260                3.280
Genomic            TCACCTGCCTGCACTTCCATGACTAAAAGTCTAAAACTGAACCAAATTGTGCGTGCGTAAGTCAAGTCAAGTCAACACAT 3280
cDNA(AK355915)     -------------------------------------------------------------------------------- 528
CircularRNA        -------------------------------------------------------------------------------- -

                                       3.300                3.320                3.340                3.360
Genomic            GGTTGTATTATAACCAATGATTTAGGGCTACTGCCATAGGTTCGCTTTCCTAATTGCAGACGTACATGAATAAACAGCTC 3360
cDNA(AK355915)     -------------------------------------------------------------------------------- 528
CircularRNA        -------------------------------------------------------------------------------- -

                                       3.380                3.400                3.420                3.440
Genomic            CATCACTATGATATGGTAATAGTTTGAGTGNNNNGACAAATATTTTATCATGGTTAAATTTGATGTTCCTGGCATAACAG 3440
cDNA(AK355915)     -------------------------------------------------------------------------------- 528
CircularRNA        -------------------------------------------------------------------------------- -

                                       3.460                3.480                3.500                3.520
Genomic            ATCCGCTCATCTACTTGACGTCATCGGTTACATCAACAGAAAAATTGTTACTCCGATATGGATACATCATGTGTCATATA 3520
cDNA(AK355915)     -------------------------------------------------------------------------------- 528
CircularRNA        -------------------------------------------------------------------------------- -

                                       3.540                3.560                3.580                3.600
Genomic            CCAGTAAAAAATGTATCTCTAAGTAAGTATATTTGTCCAAAGTATACATGTGAAGTTATCATGCAGTTGATGCAAAATGG 3600
cDNA(AK355915)     -------------------------------------------------------------------------------- 528
CircularRNA        -------------------------------------------------------------------------------- -

                                       3.620                3.640                3.660                3.680
Genomic            GAAAAAAATAAAGCACTCCATTTTTTATGCAAATGGGAAAATAATTATATCCATTTGAGCTCAAGGGCTCTGGATTTTTC 3680
cDNA(AK355915)     -------------------------------------------------------------------------------- 528
CircularRNA        -------------------------------------------------------------------------------- -

                                       3.700                3.720                3.740                3.760
Genomic            ACTTACATGCTTGACTACTTTTTGTTGTCACTATCTAGCGACGAATGAGATAAATAAAAACATTGCTACCTTACTTACCT 3760
cDNA(AK355915)     -------------------------------------------------------------------------------- 528
CircularRNA        -------------------------------------------------------------------------------- -

                                       3.780                3.800                3.820                3.840
Genomic            ATTGACTTGCTTGCTTTGTGCCACAACATTTCAGTNNNNCCTACTGAGTTATATTTGACTTGCTGCTAGTAGTAACCAGG 3840
cDNA(AK355915)     -------------------------------------------------------------------------------- 528
CircularRNA        -------------------------------------------------------------------------------- -

                                       3.860                3.880                3.900                3.920
Genomic            AGTATTTGTATTATTCAATACTAGTATTCAGTGTGAAGAAATTCTACTCAGTGTTGGCCTACTACTATGGTTTGCCGGTA 3920
cDNA(AK355915)     -------------------------------------------------------------------------------- 528
CircularRNA        -------------------------------------------------------------------------------- -

                                       3.940                3.960                3.980                4.000
Genomic            ATTAAAGATCTATCTAGGTACTTTCTGCGTGGGTCATGCATGCTGTGTACTTTCTTTATTAGTATTACCAAATGCCATTA 4000
cDNA(AK355915)     -------------------------------------------------------------------------------- 528
CircularRNA        -------------------------------------------------------------------------------- -

                                       4.020                4.040                4.060                4.080
Genomic            TTCATAACAGCCCCCTCGTAGGCAACTTATGCCTTTTTTGTCTAGCTCTTTGGTGCAGTTTTATGTTTTTTAACGGCTTG 4080
cDNA(AK355915)     -------------------------------------------------------------------------------- 528
CircularRNA        -------------------------------------------------------------------------------- -

                                       4.100                4.120                4.140                4.160
Genomic            AAATAACATGGTTTAGCTTGTATTTCGTGTGGTTAATTACATTTATCTGGGAGAATAAGGTCCATGGAAAAAACTGGGGG 4160
cDNA(AK355915)     -------------------------------------------------------------------------------- 528
CircularRNA        -------------------------------------------------------------------------------- -

                                       4.180                4.200                4.220                4.240
Genomic            TTTACAACATCTTGAGTCTCTATATTCGTTTACATACATTTTGAGTTAAATCTGAGAGTTCTTGCATGGGACACAGTTTC 4240
cDNA(AK355915)     -------------------------------------------------------------------------------- 528
CircularRNA        -------------------------------------------------------------------------------- -

                                       4.260                4.280                4.300                4.320
Genomic            CGGCATTTTATTTACCTTTCCTTCTTCCCCTCCATTATTTATTTATGTGGAATCTATTCTATGTTAAGATGTAGCATCTT 4320
cDNA(AK355915)     -------------------------------------------------------------------------------- 528
CircularRNA        -------------------------------------------------------------------------------- -

                                       4.340                4.360                4.380                4.400
Genomic            TAGATTGCATATGAGCCAGTTAGGTTTTTTGACTGCACACAAATCCGCTGCCACCTCCTCTCTGCTTCCTCGGCTGCAGC 4400
cDNA(AK355915)     -------------------------------------------------------------------------------- 528
CircularRNA        -------------------------------------------------------------------------------- -

                                       4.420                4.440                4.460                4.480
Genomic            GTCGCCGCTCCTCCTGTGTCTGGGCTGGACGGTAGCTGCCGGCCGGAGGGTTGCGCACTTCTTCGGCACACACACACAAT 4480
cDNA(AK355915)     -------------------------------------------------------------------------------- 528
CircularRNA        -------------------------------------------------------------------------------- -

                                       4.500                4.520                4.540                4.560
Genomic            GGTCGACTGGCTTCGCCAAGGTACGTTCATGTGCGCTGGCACAGGCCGAGCTTGCATGGAATGACGACCCGCCGCACGCC 4560
cDNA(AK355915)     -------------------------------------------------------------------------------- 528
CircularRNA        -------------------------------------------------------------------------------- -

                                       4.580                4.600                4.620                4.640
Genomic            CTGTATTGATCAGGTCGGTGTACTGGTAGTTTCATGTTTATCCATTATCCTTTGTGTGAATCTCAAAGGATATTTGTGTT 4640
cDNA(AK355915)     -------------------------------------------------------------------------------- 528
CircularRNA        -------------------------------------------------------------------------------- -

                                       4.660                4.680                4.700                4.720
Genomic            CTACTGAAAAACAGTCAGATGTTAAGCTCTGAAACTTTGAAACTTTGAGAGGGGTGTTTATATTTACCTAATCAGAATTT 4720
cDNA(AK355915)     -------------------------------------------------------------------------------- 528
CircularRNA        -------------------------------------------------------------------------------- -

                                       4.740                4.760                4.780                4.800
Genomic            TGCAATGGTAGCCTGCTAGATCACTTGCTAAAATAGTTATGTGCAATCTAAACTTTGAGAGGGGTGTCTATATTTTAGTT 4800
cDNA(AK355915)     -------------------------------------------------------------------------------- 528
CircularRNA        -------------------------------------------------------------------------------- -
```

```
                     4.820               4.840               4.860               4.880
Genomic         AATCAACATTTTCTATGTTTAGTTCATACAAGTCTATTGTGGTGTGGGTGACTCATGATTTATGAATGCAAATCAGTAAC 4880
cDNA(AK355915)  -------------------------------------------------------------------------------- 528
CircularRNA     -------------------------------------------------------------------------------- -

                     4.900               4.920               4.940               4.960
Genomic         CACAATTTTGTTTTATTGTCTTCTCATTCCGATAGTGTATTTACCAGTCTTGAATGATCATTAACCTGTTTTTTGTCATC 4960
cDNA(AK355915)  -------------------------------------------------------------------------------- 528
CircularRNA     -------------------------------------------------------------------------------- -

                     4.980               5.000               5.020               5.040
Genomic         TGGTGTTTATCTAATTTACTGATACAAAAATGAGTAATTGTGATGAATCTCTTACCCTAATTATTGTTTCTTCTGACTAG 5040
cDNA(AK355915)  -------------------------------------------------------------------------------- 528
CircularRNA     -------------------------------------------------------------------------------- -

                     5.060               5.080               5.100               5.120
Genomic         CTACCAGTGGAGCCGGATGTGGTCGCCTACTTTGTCGCACAACTCCTTTGACCATCACGTACAACCCAACCTGGGTGCTG 5120
cDNA(AK355915)  -------TGGAGCCGGATGTGGTCGCCTACTTTGTCGCACAACTCCTTTGACCATCACGTACAACCCAACCTGGGTGCTG 601
CircularRNA     -------------------------------------------------------------------------------- -

                     5.140               5.160               5.180               5.200
Genomic         CAGGTTGGTCTCTCTCTCTCTCAGATTTTCAAGGTCAATTCTCTTCACAACATTTACCTCTCATGGTAGTTCCTTCTCTA 5200
cDNA(AK355915)  CAGG---------------------------------------------------------------------------- 605
CircularRNA     -------------------------------------------------------------------------------- -

                     5.220               5.240               5.260               5.280
Genomic         TTTAATAGTTATCTATCTACTCCCTCCGCCCGGAATTACTTGAGGCCCAAACACATAGTGCTAGGTACATTTGTTTGAGC 5280
cDNA(AK355915)  -------------------------------------------------------------------------------- 605
CircularRNA     -------------------------------------------------------------------------------- -

                     5.300               5.320               5.340               5.360
Genomic         ATCAAGTAATTCCGGAAGGAGGTGTAGTACTTGTGTTATCTAGCATCTACTGGTGTTGTCAGTTTGGTACTTTGCTTATT 5360
cDNA(AK355915)  -------------------------------------------------------------------------------- 605
CircularRNA     -------------------------------------------------------------------------------- -

                     5.380               5.400               5.420               5.440
Genomic         TCTGGGATGTGGACTGTCGTAATTCTGAACCATCGTTACTAGTTACTTGTGGAATCGTAAACCGTTCATCTTCCGATAAG 5440
cDNA(AK355915)  -------------------------------------------------------------------------------- 605
CircularRNA     -------------------------------------------------------------------------------- -

                     5.460               5.480               5.500               5.520
Genomic         AAAAAAATGCTTGCACTCTTCACAGGAGAAGATAACTCTCTAACTCTAGAACAGGAGATTTCTCACTTTTTAGGCATCAC 5520
cDNA(AK355915)  -------------------------------------------------------------------------------- 605
CircularRNA     -------------------------------------------------------------------------------- -

                     5.540               5.560               5.580               5.600
Genomic         CTTTGTTTGTTTATTGTTGTTGACTGGAGAGGCTTGACTCGGTTCTTTGTACAGGAGAGGAGATCAGATGTATGGAAACA 5600
cDNA(AK355915)  -------------------------------------------------------AGAGGAGATCAGATGTATGGAAACA 630
CircularRNA     ----------------------------------------------------AGGAGAGGAGATCAGATGTATGGAAACA 28

                     5.620               5.640               5.660               5.680
Genomic         AAAGAAAAGGCTGCAAGTCATGGTTGTTTATACGAGAGGAGATGGTGGACCTGCAGACACCTTTGATTTCGTTAAATCCT 5680
cDNA(AK355915)  AAAGAAAAGGCTGCAAGTCATGGTTGTTTATACGAGAGGAGATGGTGGACCTGCAGACACCTTTGATTTCGTTAAATCCT 710
CircularRNA     AAAGAAAAGGCTGCAAGTCATGGTTGTTTATACGAGAGGAGATGGTGGACCTGCAGACACCTTTGATTTCGTTAAATCCT 108

                     5.700               5.720               5.740               5.760
Genomic         GTGGAGGGAAGGGGAGATTATGGATGGATGGAGCTTGGTTGAAATTGGGTTGCTGTTGCATTGGCATTTAAGACTCAAGA 5760
cDNA(AK355915)  GTGGAGGGAAGGGGAGATTATGGATGGATGGAGCTTGGTTGAAATTGGGTTGCTGTTGCATTGGCATTTAAGACTCAAGA 790
CircularRNA     GTGGAGGGAAGGGGAGATTATGGATGGATGGAGCTTGGTTGAAATTGGGTTGCTGTTGCATTGGCATTTAAGACTCAAGA 188

                     5.780               5.800               5.820               5.840
Genomic         GAATGTTCCTTCCCAAAGAAGAGGTCGGGCGTTCTCGTTGGCGACTCTAGCGGTTGAATTCATCAGCTTATCTAGATTAG 5840
cDNA(AK355915)  GAATGTTCCTTCCCAAAGAAGAGGTCGGGCGTTCTCGTTGGCGACTCTAGCGGTTGAATTCATCAGCTTATCTAGATTAG 870
CircularRNA     GAATGTTCCTTCCCAAAGAAGAGGTCGGGCGTTCTCGTTGGCGACTCTAGCGGTTGAATTCATCAGCTTATCTAGATTAG 268

                     5.860               5.880               5.900               5.920
Genomic         CTAGTGTTGGTGTTGCTATTTATGGATTTGTGGTGAAGAGGAACAACCATCCTGGTTGCTGCCTGCACGTGTTTCAAGTT 5920
cDNA(AK355915)  CTAGTGTTGGTGTTGCTATTTATGGATTTGTGGTGAAGAGGAACAACCATCCTGGTTGCTGCCTGCACGTGTTTCAAGTT 950
CircularRNA     CTAGTGTTGGTGTTGCTATTTATGGATTTGTGGTGAAGAGGAACAACCATCCTGGTTGCTGC------------------ 330

                     5.940               5.960               5.980               6.000
Genomic         TTGTTGTTGATTAACTTGGTAGTCATTGCTTCAATGATATCTTTATCAAGTTAATTTATGAACATACATGTATTTCTTCC 6000
cDNA(AK355915)  TTGTTGTTGATTAACTTGGTAGTCATTGCTTCAATGATATCTTTATCAAGTTAATTTATGAACATACATGTATTTCTTCC 1030
CircularRNA     -------------------------------------------------------------------------------- 330

                     6.020               6.040               6.060               6.080
Genomic         AAATCTTCATATGTGAAAATTTTCATGTCTGTTGCTACTTATTATTTTGATAATTTTGATCTATATCTGATATACACTAG 6080
cDNA(AK355915)  AAATCTTCATATGTGAAAATTTTCATGTCTGTTGCTACTTATTATTTTGATAATTTTGATCTATATCTGATATAC----- 1105
CircularRNA     -------------------------------------------------------------------------------- 330

                     6.100               6.120               6.140
Genomic         CAAAAGTGCCCGTGGGAGAAAAAAAACATCACACATTTTTAATCTTTTTATAATCATTTT 6140
cDNA(AK355915)  ------------------------------------------------------------ 1105
CircularRNA     ------------------------------------------------------------ 330
```

Probable aminopeptidase_circular RNA (ID: Ch5:286201637-286202872)

AGCATGAGGTGGATCGCAATGAGTTCCTAAAGCTGTGCAAGAAGGTGGAGTACACCATCAGG
GCTTGGTATCTCCTCCAGTTTGATGATTTAATGCAACTTTACTCGCTATTTGACCCGGTTAGTGG
TGAGAAGAGGCTGGAACAGCAGAATCTGACACCCGAGGAGATCGATACTCTTGAATTCAATTTC
ATGACATATCTTTTCCAGGTTGGATGCAGTAGGTTACATATATATCCTGTCCTGACACATGAAGA
CCTCCCTTGGTTTTGTATGTGTTGTTTTATTCAATCATCACAACAAACAAAAAAACTGAGTGAAAT
ATTATTTTTGAAACGCCTATTCAAAAATAACATGTGATTGCAAGTAAACACTGAGGTCACGCTAAT
ATTATGCAGATAAAACCAGTCATGAGATGTGAAGTCTGAATATCAGGCATTCATGGCCTCGCTAT
TGTTTTTCCATCAAAAACTACCTAGGGAATCCCATAGCAGTTTACAAAACAATCTTGGGGTGTCC
TGTTGCTAGAACTTCTTGCTTCGGTACTTCTTCCCTGGCTTGTTTGTTTAATGCTGCATCGAAATG
TATGGTTTTATCAGCAGTCTCCAGCTTCTGTTATTGGAAAATTTGTTCCATATCCTTTTTGTGTTG
TTTTGGGATTGAAATTAGTTTGACTTTCTGGGTTTCCTCAGTAAAGCTCGAAAAGGAAATTGTGTT
GTTAGTAGGCTGACATATTAACTTTCATAATTTGAACTAGAAAAGAGAACAAAATGGTAATTTGAT
GGTGTGACATGCCTAATTGTCATCATAGTAACAATGATTTTGCAACATTTACTGTTTAGTTTGTGC
GACAGCTTAAAGTTGTAACCATTTGGATCTACTGTATCGAGCAGATAATGGAAAAGAGCAACTTC
AAGTTGTTATCTGATGAAGAGTATGATGTTGCGCAGTCTGGAAAATATCTTTTGAACCTTCCCAT
CAAAGTTGATGAATCTAAGGTTCAAAAAATTATAAGCTTCTCAAGTTAGCATTAGAAAAGTACATG
TCCACTGCACTGAATTTTTCTCTTCAAATGTAGCTGGACAAGAAGTTGTTGACAACAT**ACTTTAA
AGAACACCCACATGATAATCTGCCTACATTTGCTGATAAG**

The nucleotides of junction-region are underlined. The nucleotides of junction-region which are supported by the junction-spaning sequencing reads are shown in red. Introns are not shown if the absence is supported by sequencing reads. In the absence of supporting sequencing reads, the intronic nucletides are shown as N.

Structural relationship between the circular RNA and its parental gene

```
                        20                  40                  60                  80
Genomic      CTGAAGATCTTATTGTATTGATTGTGCTGCAAGCGTTCTTTTCTACTCTTTAATGGATTAGATGAAAAGATATCAACACT 80
CircularRNA  -------------------------------------------------------------------------------- -

                       100                 120                 140                 160
Genomic      GAGGTTTCCCCTTAATTCACTCCATTTGTTCTAATATATGCTTAGAGCATGAGGTGGATCGCAATGAGTTCCTAAAGCTG 160
CircularRNA  ---------------------------------------------AGCATGAGGTGGATCGCAATGAGTTCCTAAAGCTG 35

                       180                 200                 220                 240
Genomic      TGCAAGAAGGTGGAGTACACCATCAGGGCTTGGTATCTCCTCCAGTTTGATGATTTAATGGTGGGCTCTTTTTGATTGTT 240
CircularRNA  TGCAAGAAGGTGGAGTACACCATCAGGGCTTGGTATCTCCTCCAGTTTGATGATTTAATG-------------------- 95

                       260                 280                 300                 320
Genomic      CCATTTGTTAGGCTTCATATATAACATGCCCAGATTCTTGTATCTCAGTGTGCACTTCTCGATGCAGCAACTTTACTCGC 320
CircularRNA  -------------------------------------------------------------------CAACTTTACTCGC 108

                       340                 360                 380                 400
Genomic      TATTTGACCCGGTTAGTGGTGAGAAGAGGCTGGAACAGCAGAATCTGACACCCGAGGAGATCGATACTCTTGAATTCAAT 400
CircularRNA  TATTTGACCCGGTTAGTGGTGAGAAGAGGCTGGAACAGCAGAATCTGACACCCGAGGAGATCGATACTCTTGAATTCAAT 188

                       420                 440                 460                 480
Genomic      TTCATGACATATCTTTTCCAGGTTGGATGCAGTAGGTTACATATATATCCTGTCCTGACACATGAAGACCTCCCTTGGTT 480
CircularRNA  TTCATGACATATCTTTTCCAGGTTGGATGCAGTAGGTTACATATATATCCTGTCCTGACACATGAAGACCTCCCTTGGTT 268

                       500                 520                 540                 560
Genomic      TTGTATGTGTTGTTTTATTCAATCATCACAACAAACAAAAAAACTGAGTGAAATATTATTTTTGAAACGCCTATTCAAAA 560
CircularRNA  TTGTATGTGTTGTTTTATTCAATCATCACAACAAACAAAAAAACTGAGTGAAATATTATTTTTGAAACGCCTATTCAAAA 348

                       580                 600                 620                 640
Genomic      ATAACATGTGATTGCAAGTAAACACTGAGGTCACGCTAATATTATGCAGATAAAACCAGTCATGAGATGTGAAGTCTGAA 640
CircularRNA  ATAACATGTGATTGCAAGTAAACACTGAGGTCACGCTAATATTATGCAGATAAAACCAGTCATGAGATGTGAAGTCTGAA 428

                       660                 680                 700                 720
Genomic      TATCAGGCATTCATGGCCTCGCTATTGTTTTTCCATCAAAAACTACCTAGGGAATCCCATAGCAGTTTACAAAACAATCT 720
CircularRNA  TATCAGGCATTCATGGCCTCGCTATTGTTTTTCCATCAAAAACTACCTAGGGAATCCCATAGCAGTTTACAAAACAATCT 508

                       740                 760                 780                 800
Genomic      TGGGGTGTCCTGTTGCTAGAACTTCTTGCTTCGGTACTTCTTCCCTGGCTTGTTTGTTTAATGCTGCATCGAAATGTATG 800
CircularRNA  TGGGGTGTCCTGTTGCTAGAACTTCTTGCTTCGGTACTTCTTCCCTGGCTTGTTTGTTTAATGCTGCATCGAAATGTATG 588

                       820                 840                 860                 880
Genomic      GTTTTATCAGCAGTCTCCAGCTTCTGTTATTGGAAAATTTGTTCCATATCCTTTTTGTGTTGTTTTGGGATTGAAATTAG 880
CircularRNA  GTTTTATCAGCAGTCTCCAGCTTCTGTTATTGGAAAATTTGTTCCATATCCTTTTTGTGTTGTTTTGGGATTGAAATTAG 668

                       900                 920                 940                 960
Genomic      TTTGACTTTCTGGGTTTCCTCAGTAAAGCTCGAAAAGGAAATTGTGTTGTTAGTAGGCTGACATATTAACTTTCATAATT 960
CircularRNA  TTTGACTTTCTGGGTTTCCTCAGTAAAGCTCGAAAAGGAAATTGTGTTGTTAGTAGGCTGACATATTAACTTTCATAATT 748

                       980               1.000               1.020               1.040
Genomic      TGAACTAGAAAAGAGAACAAAATGGTAATTTGATGGTGTGACATGCCTAATTGTCATCATAGTAACAATGATTTTGCAAC 1040
CircularRNA  TGAACTAGAAAAGAGAACAAAATGGTAATTTGATGGTGTGACATGCCTAATTGTCATCATAGTAACAATGATTTTGCAAC 828

                     1.060               1.080               1.100               1.120
Genomic      ATTTACTGTTTAGTTTGTGCGACAGCTTAAAGTTGTAACCATTTGGATCTACTGTATCGAGCAGATAATGGAAAAGAGCA 1120
CircularRNA  ATTTACTGTTTAGTTTGTGCGACAGCTTAAAGTTGTAACCATTTGGATCTACTGTATCGAGCAGATAATGGAAAAGAGCA 908

                     1.140               1.160               1.180               1.200
Genomic      ACTTCAAGTTGTTATCTGATGAAGAGTATGATGTTGCGCAGTCTGGAAAATATCTTTTGAACCTTCCCATCAAAGTTGAT 1200
CircularRNA  ACTTCAAGTTGTTATCTGATGAAGAGTATGATGTTGCGCAGTCTGGAAAATATCTTTTGAACCTTCCCATCAAAGTTGAT 988

                     1.220               1.240               1.260               1.280
Genomic      GAATCTAAGGTTCAAAAAATTATAAGCTTCTCAAGTTAGCATTAGAAAAGTACATGTCCACTGCACTGAATTTTTCTCTT 1280
CircularRNA  GAATCTAAGGTTCAAAAAATTATAAGCTTCTCAAGTTAGCATTAGAAAAGTACATGTCCACTGCACTGAATTTTTCTCTT 1068

                     1.300               1.320               1.340               1.360
Genomic      CAAATGTAGCTGGACAAGAAGTTGTTGACAACATACTTTAAAGAACACCCACATGATAATCTGCCTACATTTGCTGATAA 1360
CircularRNA  CAAATGTAGCTGGACAAGAAGTTGTTGACAACATACTTTAAAGAACACCCACATGATAATCTGCCTACATTTGCTGATAA 1148

                     1.380               1.400               1.420               1.440
Genomic      GGTAATCTTCATGTCTTCCTAACTGACTCACCACTTTCCCTTGATCCATGTATTACGGTTTTAATTTGAGGAATTTATGA 1440
CircularRNA  G------------------------------------------------------------------------------- 1149

                     1.460               1.480               1.500               1.520
Genomic      TTACTAGTGTGCGATAAGGGTGTGTTCTCATTTTATGATTATGTATCTTCTTTCCTTACTTTTGCATATCTAATGAGGTT 1520
CircularRNA  -------------------------------------------------------------------------------- 1149

Genomic      A 1521
CircularRNA  - 1149
```

Unknown_circular RNA (ID: Ch5:6888869-6889034)

CATGAAGATAGTGATGAAATAACCATTATGAAGGAAATTCTTGAAGACGTAGTGCATGCGGAA
GAGCATGTTGTCAACTAACAAGTTTCAACACAACACCAACTATTCAATCCATAGAGGGTAGGG**G**
GCATTCATCTTCTAGGACAGCCAGTTTGCCCCCGGTGTC

The nucleotides of junction-region are underlined. The nucleotides of junction-region which are supported by the junction-spaning sequencing reads are shown in red. Introns are not shown if the absence is supported by sequencing reads. In the absence of supporting sequencing reads, the intronic nucletides are shown as N.

Structural relationship between the circular RNA and its parental gene

```
Genomic     GTTACTTTGAAGCAAGTACGATGGACTCAGACAAGGAGTACTTCTATGCCCATTTCATGGATTCGTCATGGTCGAACGAG 80
CircularRNA -------------------------------------------------------------------------------- -

Genomic     CATGAAGATAGTGATGAAATAACCATTATGAAGGAAATTCTTGAAGACGTAGTGCATGCGGAAGAGCATGTTGTCAACTA 160
CircularRNA CATGAAGATAGTGATGAAATAACCATTATGAAGGAAATTCTTGAAGACGTAGTGCATGCGGAAGAGCATGTTGTCAACTA 80

Genomic     ACAAGTTTCAACACAACACCAACTATTCAATCCATAGAGGGTAGGGGGCATTCATCTTCTAGGACAGCCAGTTTGCCCCC 240
CircularRNA ACAAGTTTCAACACAACACCAACTATTCAATCCATAGAGGGTAGGGGGCATTCATCTTCTAGGACAGCCAGTTTGCCCCC 160

Genomic     GGTGTCGTATTTGGACCATACTTTGCCATGGAGGAATTCATAAGCAACTTCAAAATTGTCTGGTTGCCCATCTATGGTCA 320
CircularRNA GGTGTC-------------------------------------------------------------------------- 166

Genomic     TTACATGGAGACAACTAGACAAAGTGTTAGTGTAAGTTGA 360
CircularRNA ---------------------------------------- 166
```

Probable long non-coding RNA_circular RNA (ID: Ch5:53926495-53926935)

<u>G</u>AGGAAGGCAGGAAGATGGCATTGTGTGTAGAAAGGCGTCATCCACCCTGTGCCTTTGCGTC
AGCCGCGATGGTGATCAGTTGTGGGCAGATCCAATTTCTGCACGTGGATCTACTACTCGGGGT
GGCATGACATGGTTGTTGTGGAGTGACGTGCCACAAATATTGTGTTGTTTGGGTGAGTTTGGAG
AGATAGAAGTTGTGTTGTGCTCTAGTCTACGCCTTCAACTGTACCAAGCGCAACTTATTCTGAGT
TTCTATGCTCTCAAGCTTAAGCCTTTCTCTGAACCAATCCTAAGCCCAAGAAACCGAGCCTAGCT
ATTAAAGTACTGATAACATGGACTTATATTGATGTGCTTAATTTACGGAATTATGAGATCATCGCT
TAAGAACC**AATAACTTTGCATGGAATAAAAGCCAACTTTAGATTGTGATTGGTTC<u>A</u>**

The nucleotides of junction-region are underlined. The nucleotides of junction-region which are supported by the junction-spaning sequencing reads are shown in red. Introns are not shown if the absence is supported by sequencing reads. In the absence of supporting sequencing reads, the intronic nucletides are shown as N.

Structural relationship between the circular RNA and its parental gene

```
Genomic      GAAAACGTTGTCGGGAGTGGACTACCACAACCATCAGCTTTTGAACATGGAGTTGTGTAGTGAGGGAATAATGACAGGAG  80
CircularRNA  --------------------------------------------------------------------------------  -

Genomic      AAGCGAGGAAGGCAGGAAGATGGCATTGTGTGTAGAAAGGCGTCATCCACCCTGTGCCTTTGCGTCAGCCGCGATGGTGA  160
CircularRNA  ----GAGGAAGGCAGGAAGATGGCATTGTGTGTAGAAAGGCGTCATCCACCCTGTGCCTTTGCGTCAGCCGCGATGGTGA  76

Genomic      TCAGTTGTGGGCAGATCCAATTTCTGCACGTGGATCTACTACTCGGGGTGGCATGACATGGTTGTTGTGGAGTGACGTGC  240
CircularRNA  TCAGTTGTGGGCAGATCCAATTTCTGCACGTGGATCTACTACTCGGGGTGGCATGACATGGTTGTTGTGGAGTGACGTGC  156

Genomic      CACAAATATTGTGTTGTTTGGGTGAGTTTGGAGAGATAGAAGTTGTGTTGTGCTCTAGTCTACGCCTTCAACTGTACCAA  320
CircularRNA  CACAAATATTGTGTTGTTTGGGTGAGTTTGGAGAGATAGAAGTTGTGTTGTGCTCTAGTCTACGCCTTCAACTGTACCAA  236

Genomic      GCGCAACTTATTCTGAGTTTCTATGCTCTCAAGCTTAAGCCTTTCTCTGAACCAATCCTAAGCCCAAGAAACCGAGCCTA  400
CircularRNA  GCGCAACTTATTCTGAGTTTCTATGCTCTCAAGCTTAAGCCTTTCTCTGAACCAATCCTAAGCCCAAGAAACCGAGCCTA  316

Genomic      GCTATTAAAGTACTGATAACATGGACTTATATTGATGTGCTTAATTTACGGAATTATGAGATCATCGCTTAAGAACCAAT  480
CircularRNA  GCTATTAAAGTACTGATAACATGGACTTATATTGATGTGCTTAATTTACGGAATTATGAGATCATCGCTTAAGAACCAAT  396

Genomic      AACTTTGCATGGAATAAAAGCCAACTTTAGATTGTGATTGTGTGTGTCAATCATGTTACATCCTTAAGCCCCAACAAGCA  560
CircularRNA  AACTTTGCATGGAATAAAAGCCAACTTTAGATTGTGATTGGTTCA-----------------------------------  441

Genomic      CCAAGTTGGCTGGTGCCATACGGAAGTTGTTTTGAGTAGTTATGATTGGAAGATGAGATAATCTGATGGTGATGTAGAAA  640
CircularRNA  --------------------------------------------------------------------------------  441
```

Probable long non-coding RNA_circular RNA (ID: Ch1:363958561-363959513)

CCTATGTACATAAATCAGGTCAGTGTCTCGTAGACGGTCTGTTTGTTTCATTTGTTATCGCCGC
CTCCGTGAGTTGGCTTGCAGGATGGATCTGGATCTGGTCTGGAAATGAAGCTCAGTGTGGTGTA
TGAACAGTATGTTGAAGTGGACTTGAGGCAGGTCAGTTTACCATGTTTGGTCACATCGATCTTGG
TGACGCCAGATTTGAGTGGATATACCTTAAGTTCTTTGATTTATTTTCGTCTGATGGCTCAACATG
AGTACCATTATCCCTCGGTACAAGGTGCATTCTCGTTGGTTCTCGTTGTTACAGCTGATCTCCGC
TTGTGGGGTATGTGTTTGTTCTCCTTCAGATGCCGGAGGGTGATGGTTGCTGAGTGATGAATCA
GAGGGTGGAGGTTTGTTTTAATGTTCATGGTTATTTGGATTGGTTATGGCTCAATGCCTTGCATG
ATATAAGATAGCATCTTGTGTGCTGAAGTTTTTGTTTTGGATTGAAGTCGGATTGGATCATTAAAC
ATGGAATGGAGATCAGAGAATTTGCGAACTGCGTTTGGATAATTGATCCTCAATTTAGTTTCTTT
GTTTGCACAGTCGAATTTAGCTTACAGCATCTCTCATATGGAACTGTTGGTTCTCCAGCTATTGG
ACTTATGGGGCTTTCTGGAAGGGCTTTGACTGTTGGATGGGGTTTCTTCTCTTGACTTCATCTTT
CCTGATTTGCTTTGAACATTGCACTTCCTAGACATTGTTTTCTATGGCCTTGGTAACTACATGTCC
TTGCAGATATTTACATGGACGAGAGATAATATAGTTTGCTATCTGGAACTGGTGTTTCCCTAACT
GATATCTCCCAGCCAAGCAGGCTTATCCTATCCCCTCAATGTTTGATGCATCATTTAGCTGTT**CA**
TAGGCAGCTTCGTTTCCCCAAAGCAGAAGTTGAAACGAAGCTG

The nucleotides of junction-region are underlined. The nucleotides of junction-region which are supported by the junction-spaning sequencing reads are shown in red. Introns are not shown if the absence is supported by sequencing reads. In the absence of supporting sequencing reads, the intronic nucletides are shown as N.

Structural relationship between the circular RNA and its parental gene

```
                    20                  40                  60                  80
cDNA(AK248174.1) GGCGCGCTGCCCTCCTCCCTCTCTCTTGGAGCTCTCGTTCGGACCGGCCGCGCCGCCGGTGTCGATCGCCTGAGTCGCCT 80
     CircularRNA -------------------------------------------------------------------------------- -
                   100                 120                 140                 160
cDNA(AK248174.1) CCTCGCGTCTTCGCGACTGTTCCCTTCCCCTTATCCATCAAATCTCGATTCGTTTCGAATCGCGTTTGATTTATCTCCTC 160
     CircularRNA -------------------------------------------------------------------------------- -
                   180                 200                 220                 240
cDNA(AK248174.1) GCGGCTTTGCTTCCTGTGCGCTCGCAGGGGATCCGGCCCATCTCCGCGCGACCTCGGGGAGCGAGCGATCGATCGATCGG 240
     CircularRNA -------------------------------------------------------------------------------- -
                   260                 280                 300                 320
cDNA(AK248174.1) GGGATTCCGTCGATTTTGCTCTGATTTCTGTGTGATGAGAGGGCCGAAGCAAGGTCAGTTTCCCGTGCTCGATCGGTCAG 320
     CircularRNA -------------------------------------------------------------------------------- -
                   340                 360                 380                 400
cDNA(AK248174.1) ATCGGTTCCTTGGTTCCGTCACCTGCCTGCCTTCCTCGGCGATTTCAGATTTGGATCCAGTTGGCCGGCTCTCGCCCGTG 400
     CircularRNA -------------------------------------------------------------------------------- -
                   420                 440                 460                 480
cDNA(AK248174.1) CGGCCTGCCCCCGATCCAGCCCTCTCGTCACGGGTTCCGTCGTCATCGGTTAATAATACGAGGTGGATTCGACGCCCTCG 480
     CircularRNA -------------------------------------------------------------------------------- -
                   500                 520                 540                 560
cDNA(AK248174.1) TGGATTCTAAATTTTACCGGCTGATCCCGCTTTTGGTATGTGTTTGTGCTCTCATCCTAGATGCAGCAGAAGCAGAGGCA 560
     CircularRNA -------------------------------------------------------------------------------- -
                   580                 600                 620                 640
cDNA(AK248174.1) AGGGACGCTCGGTAGTTCGCAAGAGATGATACATAAAAAGGGTGGAGGTTTGGTTTCTGTTATGGTTATTTTGATTCATT 640
     CircularRNA -------------------------------------------------------------------------------- -
                   660                 680                 700                 720
cDNA(AK248174.1) GGTCACGGTTCAGCGGTTACTCTGATTGATATGGACAGATGATCAAGCTTTGGATCTCACCAGTCTGCAATTGGATGATA 720
     CircularRNA -------------------------------------------------------------------------------- -
                   740                 760                 780                 800
cDNA(AK248174.1) ATAATAATTCAACCCCTCCCGATTTATTTATTTGGTTTGCACAGCACAGTTAATCTACAACTCATCTGATTTAGATTCGT 800
     CircularRNA -------------------------------------------------------------------------------- -
                   820                 840                 860                 880
cDNA(AK248174.1) TCCTTGATGGTTCTCCAGAAAATGGGATGACACGGCTTAGCGGGACCACCTATCTTCGGCTGATTTGTGTTTGGATGAAT 880
     CircularRNA -------------------------------------------------------------------------------- -
                   900                 920                 940                 960
cDNA(AK248174.1) ATCACTACTCCTATCTCTATTTAATCCCTTACTCTCCACCGTTTAATTTTAGCCTCCAACAATTCTAATTTGGATTTGGT 960
     CircularRNA -------------------------------------------------------------------------------- -
                   980               1.000               1.020               1.040
cDNA(AK248174.1) CTTCACTGTCCAGAAATTGGGATTACTTGGCTTAGTGGGAGCTGACTTATTATCTTCTTGTGTTTGGATGATCAACTCTC 1040
     CircularRNA -------------------------------------------------------------------------------- -
                 1.060               1.080               1.100               1.120
cDNA(AK248174.1) TCTCTCTCTCTCTCTCTCTCTCTTCAATTTGTTCCCCTCCACAGTTCAATTATTCTCTCAACAATTCTGATTTGGAATGG 1120
     CircularRNA -------------------------------------------------------------------------------- -
                 1.140               1.160               1.180               1.200
cDNA(AK248174.1) TTGCTGGTTGTCCAGAAACTGGAATTACACGGGCCTAGCGGGTGCTCCTATGTGGCCTCAGCATGACGCTTCTTCTTTCC 1200
     CircularRNA -------------------------------------------------------------------------------- -
                 1.220               1.240               1.260               1.280
cDNA(AK248174.1) TGGCTCGAACTTTCTGGAACTTTCTTACTCAATATTAATTAGAACTCAGCAGACATTAGTTTTCCCTGTCTTAGTCATTC 1280
     CircularRNA -------------------------------------------------------------------------------- -
                 1.300               1.320               1.340               1.360
cDNA(AK248174.1) TCAGCTCTTGCAGATAGTGTCTTGCAAAGGATATATAATTGGAACCGGTGTTGTCTACCTGACCTTCCCAGTCGAGACAG 1360
     CircularRNA -------------------------------------------------------------------------------- -
                 1.380               1.400               1.420               1.440
cDNA(AK248174.1) GCTTACTTATCCTACCTCCTCATCGTTTCATCCTAGTTTTCCCTAACTGAAGCATTTATGGAAATGTATCTTGCTTGTTG 1440
     CircularRNA -------------------------------------------------------------------------------- -
                 1.460               1.480               1.500               1.520
cDNA(AK248174.1) CAGGTCGTAGTTTACGAACTCAAGGCCACCAACCTGATGCTGACTTTGCAGTTCTTCTAAAACCACTATCTACTCAATTC 1520
     CircularRNA -------------------------------------------------------------------------------- -
                 1.540               1.560               1.580               1.600
cDNA(AK248174.1) CTTAACTGAGATAATGTTAATAAGATCTCTTACTAGGCAGTATTTTAAATTCAGATTTAGCTGCGAATGGTGCAAGAATG 1600
     CircularRNA -------------------------------------------------------------------------------- -
                 1.620               1.640               1.660               1.680
cDNA(AK248174.1) AAGCTTCAAACTAGTGGCATATCCTACAACCGGAAAAATAATAATCTCCTACCCTCCTATTTTTATTTCATTCAGCTACA 1680
     CircularRNA -------------------------------------------------------------------------------- -
                 1.700               1.720               1.740               1.760
cDNA(AK248174.1) TTTTCATGTCTTTGATTTGTGCTTATGCCCTCCATGTCGTTGCAGATCTGTTTTGCCTAAGACAATGCTTGTAGCGAGTC 1760
     CircularRNA -------------------------------------------------------------------------------- -
                 1.780               1.800               1.820               1.840
cDNA(AK248174.1) AAAAATCTTCTGGTTTGCTATCTATCTCTTTGTTGTTTGATTGGTTATATCTGTACTCTTGATCCCTGTGTTGGTCTTCC 1840
     CircularRNA -------------------------------------------------------------------------------- -
                 1.860               1.880               1.900               1.920
cDNA(AK248174.1) TATAAGTGTGTGGGTTGCACTGCAGGATTTATTTAGGAAGTCTGTGCCTACATATGCAAACCTTTGCTGTGTGTCTGTTG 1920
     CircularRNA -------------------------------------------------------------------------------- -
                 1.940               1.960               1.980               2.000
cDNA(AK248174.1) CGGTGTCAGGTCAGTAACTGTTGGTTCGTTTGTGTTCCCATGCCGGATGGGATCTGATATGTGGAGATCACTATGATGGT 2000
     CircularRNA -------------------------------------------------------------------------------- -
```

```
                                 2.020                2.040                2.060                2.080
cDNA(AK248174.1) GTATGTTCAAGGGGATTTTAGGTAGGTCAGATAACATGCCTATTGCCCGTGTGGTCTTCTAGTTCTCATCACGAGTTCCA 2080
     CircularRNA -------------------------------------------------------------------------------- -
                                 2.100                2.120                2.140                2.160
cDNA(AK248174.1) TCATAATCAATATAAGGTGGATTAGAGGTATTGGTGGATTCTTATTGTAGCAGTTGATCTCGCTTTTGGTTTGTGTTTGT 2160
     CircularRNA -------------------------------------------------------------------------------- -
                                 2.180                2.200                2.220                2.240
cDNA(AK248174.1) GCTCCTTCAGATGCAGCAGAAGCAGGAAGTGGTTCCGCATAGATGAACCGAAAGGATGGTGGTTTGGTTTCATATGGTCA 2240
     CircularRNA -------------------------------------------------------------------------------- -
                                 2.260                2.280                2.300                2.320
cDNA(AK248174.1) GAGGTCTCCAGGGATGATGTTTGGTGTTGGACTGAAGTTTTGGATCGCACATCAACCTGGAACTTGAGATCCGTGTTCAG 2320
     CircularRNA -------------------------------------------------------------------------------- -
                                 2.340                2.360                2.380                2.400
cDNA(AK248174.1) ATGATCCTCTGAATTTCACTTCATTTCATTTGGATTCGTTCAGTTCTAGCCTAACTTTAATTTGGATTCATTGCTCGTTG 2400
     CircularRNA -------------------------------------------------------------------------------- -
                                 2.420                2.440                2.460                2.480
cDNA(AK248174.1) TCACAAATTGAGATTACTCGGCTTAGTGGGAGCGGCGTACTATCTTCTTGCTGACCGACCTGTGTTTGGATGATCAAAAT 2480
     CircularRNA -------------------------------------------------------------------------------- -
                                 2.500                2.520                2.540                2.560
cDNA(AK248174.1) TCTGTTCAATTTGTTCTTCTCCACGGTTCAATTATTCTCCCAACAATTCTGATTTGGATTGTTTGCTGGTTATCCAGAAT 2560
     CircularRNA -------------------------------------------------------------------------------- -
                                 2.580                2.600                2.620                2.640
cDNA(AK248174.1) CTGGAATTGCACAGGCCTAGCGGGTGCTCTTATTTTGGCTCAGCATGGCGGTTCTTTTTCTGTGGCTAAACTTTTTGGAC 2640
     CircularRNA -------------------------------------------------------------------------------- -
                                 2.660                2.680                2.700                2.720
cDNA(AK248174.1) CTTCTTTATTCAATACAGTATTAATTAGAACTCAGCAGACATTAGTTTTCCCTGCCTTAGTCATTTTCAGCTCTTGCAGA 2720
     CircularRNA -------------------------------------------------------------------------------- -
                                 2.740                2.760                2.780                2.800
cDNA(AK248174.1) TAGTGTCTTGCAAGGGATATAATTGAAAGCCATGTGTCTACCTGACCTAGCTTCACAGTGGAGACAGACTTACTCATCCT 2800
     CircularRNA -------------------------------------------------------------------------------- -
                                 2.820                2.840                2.860                2.880
cDNA(AK248174.1) TTACCTCCTCATTCGTTTTTACCTACTTTCCTAATCCTAGTTTTCCCATGACTGAAATATTTATAGAAACGTATCTTACT 2880
     CircularRNA -------------------------------------------------------------------------------- -
                                 2.900                2.920                2.940                2.960
cDNA(AK248174.1) CGTTGCAGGTCGTAGTTTACGAACTCAAGTCCACCAACCTGATATTGAGTTTGCAGTTCTTCTAAATCTACTATCTTCTC 2960
     CircularRNA -------------------------------------------------------------------------------- -
                                 2.980                3.000                3.020                3.040
cDNA(AK248174.1) AATTCCTTGACTTAGATAATAATAATAAGATATCTTACTAGGTAATATCTTAAATTCAGATTTAACTGTAAAGGTGCAAT 3040
     CircularRNA -------------------------------------------------------------------------------- -
                                 3.060                3.080                3.100                3.120
cDNA(AK248174.1) AATGAAGCTTCAAAACTAGTGGCACATCCTACAACCGGAAAAAACCTAATCTCTTATCCTCTTATTTTTATTTCATTGAG 3120
     CircularRNA -------------------------------------------------------------------------------- -
                                 3.140                3.160                3.180                3.200
cDNA(AK248174.1) GTACATGTCTTCTCCGCCTTAGTCGGTAATTCTCAGCTCTTGTAGATACTGACTTGAAAATGATATAACTGCAGTTTGGA 3200
     CircularRNA -------------------------------------------------------------------------------- -
                                 3.220                3.240                3.260                3.280
cDNA(AK248174.1) ACCGGTGTTGTTGTCCTCCGTCAACCATAGCCGAACTTCCTAGCTGAGACAGGCTTTTACTTGTTCCCCGTCCATCTTTG 3280
     CircularRNA -------------------------------------------------------------------------------- -
                                 3.300                3.320                3.340                3.360
cDNA(AK248174.1) ATCCATGCTTTAGCTATTTAAAAGTAACGTTTTCCCCGAACTGAAAATTTATAGAAAGGTCTCTTGCTCGTTGCAGGTTG 3360
     CircularRNA -------------------------------------------------------------------------------- -
                                 3.380                3.400                3.420                3.440
cDNA(AK248174.1) TAGTTGATGACCTCGAGTCCAACCTGATGATGAATTTGTTTGCAGATCTTTGAAATCTACGAGTATTGTTTCAATTCATC 3440
     CircularRNA -------------------------------------------------------------------------------- -
                                 3.460                3.480                3.500                3.520
cDNA(AK248174.1) ACAAACTTATATAACCTACAAATTATCTTACTAGGCAATATTTATAATACAGATTCAAATGCAAAGGTGCATGAATCGAA 3520
     CircularRNA -------------------------------------------------------------------------------- -
                                 3.540                3.560                3.580                3.600
cDNA(AK248174.1) GGACAATCCGAAGCTTTCAACTAATGGCACATCCTACAACCGAAAGGCTCTAGCCCTCCTATTTTTTCATTTCGTTTAGC 3600
     CircularRNA -------------------------------------------------------------------------------- -
                                 3.620                3.640                3.660                3.680
cDNA(AK248174.1) TACTTGGCCATGTCTTTGATATAGGTTTTTGCTCCTGCCCTTCTTGTGGTTGCAGATTATTCTTTAGCCGGTAATGACAT 3680
     CircularRNA -------------------------------------------------------------------------------- -
                                 3.700                3.720                3.740                3.760
cDNA(AK248174.1) GGAATCTGTTAAATATTTTGGTTTGTGATCTTGCTCTCCATGATCCTACTAGTTATCCGTACTCTTGATCCCTGTGTTCT 3760
     CircularRNA -------------------------------------------------------------------------------- -
                                 3.780                3.800                3.820                3.840
cDNA(AK248174.1) TTTGGTCTTCTGCAAGTGTGGGTTGCAGGATATAACAGTTAGGATTTTATGCCCAAACCTATGTACATAAATCAGGTCAG 3840
     CircularRNA ---------------------------------------------------------CCTATGTACATAAATCAGGTCAG 23
                                 3.860                3.880                3.900                3.920
cDNA(AK248174.1) TGTCTCGTAGACGGTCTGTTTGTTTCATTTGTTATCGCCGCCTCCGTGAGTTGGCTTGCAGGATGGATCTGGATCTGGTC 3920
     CircularRNA TGTCTCGTAGACGGTCTGTTTGTTTCATTTGTTATCGCCGCCTCCGTGAGTTGGCTTGCAGGATGGATCTGGATCTGGTC 103
                                 3.940                3.960                3.980                4.000
cDNA(AK248174.1) TGGAAATGAAGCTCAGTGTGGTGTATGAACAGTATGTTGAAGTGGACTTGAGGCAGGTCAGTTTACCATGTTTGGTCACA 4000
     CircularRNA TGGAAATGAAGCTCAGTGTGGTGTATGAACAGTATGTTGAAGTGGACTTGAGGCAGGTCAGTTTACCATGTTTGGTCACA 183
```

```
                                4.020                          4.040                          4.060                          4.080
cDNA(AK248174.1)  TCGATCTTGGTGACGCCAGATTTGAGTGGATATACCTTAAGTTCTTTGATTTATTTTCGTCTGATGGCTCAACATGAGTA  4080
     CircularRNA  TCGATCTTGGTGACGCCAGATTTGAGTGGATATACCTTAAGTTCTTTGATTTATTTTCGTCTGATGGCTCAACATGAGTA  263
                                4.100                          4.120                          4.140                          4.160
cDNA(AK248174.1)  CCATTATCCCTCGGTACAAGGTGCATTCTCGTTGGTTCTCGTTGTTACAGCTGATCTCCGCTTGTGGGGTATGTGTTTGT  4160
     CircularRNA  CCATTATCCCTCGGTACAAGGTGCATTCTCGTTGGTTCTCGTTGTTACAGCTGATCTCCGCTTGTGGGGTATGTGTTTGT  343
                                4.180                          4.200                          4.220                          4.240
cDNA(AK248174.1)  TCTCCTTCAGATGCCGGAGGGTGATGGTTGCTGAGTGATGAATCAGAGGGTGGAGGTTTGTTTTAATGTTCATGGTTATT  4240
     CircularRNA  TCTCCTTCAGATGCCGGAGGGTGATGGTTGCTGAGTGATGAATCAGAGGGTGGAGGTTTGTTTTAATGTTCATGGTTATT  423
                                4.260                          4.280                          4.300                          4.320
cDNA(AK248174.1)  TGGATTGGTTATGGCTCAATGCCTTGCATGATATAAGATAGCATCTTGTGTGCTGAAGTTTTTGTTTTGGATTGAAGTCG  4320
     CircularRNA  TGGATTGGTTATGGCTCAATGCCTTGCATGATATAAGATAGCATCTTGTGTGCTGAAGTTTTTGTTTTGGATTGAAGTCG  503
                                4.340                          4.360                          4.380                          4.400
cDNA(AK248174.1)  GATTGGATCATTAAACATGGAATGGAGATCAGAGAATTTGCGAACTGCGTTTGGATAATTGATCCTCAATTTAGTTTCTT  4400
     CircularRNA  GATTGGATCATTAAACATGGAATGGAGATCAGAGAATTTGCGAACTGCGTTTGGATAATTGATCCTCAATTTAGTTTCTT  583
                                4.420                          4.440                          4.460                          4.480
cDNA(AK248174.1)  TGTTTGCACAGTCGAATTTAGCTTACAGCATCTCTCATATGGAACTGTTGGTTCTCCAGCTATTGGACTTATGGGGCTTT  4480
     CircularRNA  TGTTTGCACAGTCGAATTTAGCTTACAGCATCTCTCATATGGAACTGTTGGTTCTCCAGCTATTGGACTTATGGGGCTTT  663
                                4.500                          4.520                          4.540                          4.560
cDNA(AK248174.1)  CTGGAAGGGCTTTGACTGTTGGATGGGGTTTCTTCTCTTGACTTCATCTTTCCTGATTTGCTTTGAACATTGCACTTCCT  4560
     CircularRNA  CTGGAAGGGCTTTGACTGTTGGATGGGGTTTCTTCTCTTGACTTCATCTTTCCTGATTTGCTTTGAACATTGCACTTCCT  743
                                4.580                          4.600                          4.620                          4.640
cDNA(AK248174.1)  AGACATTGTTTTCTATGGCCTTGGTAACTACATGTCCTTGCAGATATTTACATGGACGAGAGATAATATAGTTTGCTATC  4640
     CircularRNA  AGACATTGTTTTCTATGGCCTTGGTAACTACATGTCCTTGCAGATATTTACATGGACGAGAGATAATATAGTTTGCTATC  823
                                4.660                          4.680                          4.700                          4.720
cDNA(AK248174.1)  TGGAACTGGTGTTTCCCTAACTGATATCTCCCAGCCAAGCAGGCTTATCCTATCCCCTCAATGTTTGATGCATCATTTAG  4720
     CircularRNA  TGGAACTGGTGTTTCCCTAACTGATATCTCCCAGCCAAGCAGGCTTATCCTATCCCCTCAATGTTTGATGCATCATTTAG  903
                                4.740                          4.760                          4.780                          4.800
cDNA(AK248174.1)  CTGTTCATAGGCAGCTTCGTTTCCCCAAAGCAGAAGTTGAAACGAAGGTTTCTTGGTCATTGCAGGTCCTAACTGACGAT  4800
     CircularRNA  CTGTTCATAGGCAGCTTCGTTTCCCCAAAGCAGAAGTTGAAACGAAGCTG------------------------------  953
                                4.820                          4.840                          4.860                          4.880
cDNA(AK248174.1)  TTAGGTCCAACCTGATGATGAGTATGTTCGAAGATATTCTAAACCCTGCACCTTTCCAATTCGTCGTAATTTTTTGAACA  4880
     CircularRNA  --------------------------------------------------------------------------------  953
                                4.900                          4.920                          4.940                          4.960
cDNA(AK248174.1)  TATAATTTATCTTATGGAGACAAAATTTAAACATTCAGATTGAATTGCTGGATGTAGAAGGTTTGAAGGTTGCTACAGGG  4960
     CircularRNA  --------------------------------------------------------------------------------  953
                                4.980                          5.000                          5.020                          5.040
cDNA(AK248174.1)  CATCAATCTAGACGGACCATCCTACAGCCGAAAAAACCCAGTGCTCTCTATTTGATCTCTTGAAGAAGCTTCTGTTGTCT  5040
     CircularRNA  --------------------------------------------------------------------------------  953
                                5.060                          5.080                          5.100                          5.120
cDNA(AK248174.1)  TAGCTGCCTTGTGCTTGTAGCATTGATTGTGTTTTTTTGCCTCCGCCTTCTTGTGGTTGCAGATTGTTTAGCCCAAGCTA  5120
     CircularRNA  --------------------------------------------------------------------------------  953
                                5.140                          5.160                          5.180                          5.200
cDNA(AK248174.1)  TGCTGGTAGCCATCAAAATATGTCGCATTTTTGGTTTCTGATCCTCTCCTGACTTCTATAGTTGGGTAATGTTGATGGCT  5200
     CircularRNA  --------------------------------------------------------------------------------  953
                                5.220                          5.240                          5.260                          5.280
cDNA(AK248174.1)  TGATACGCAGAAAGAGGTTAGGGATTATCGCTATATATACTAAAAACAATGGAGAGTTGAAGCTTCTCGACTCGAAGCTT  5280
     CircularRNA  --------------------------------------------------------------------------------  953
                                5.300                          5.320                          5.340                          5.360
cDNA(AK248174.1)  GAACAAACAGTTTGGCATGGACACTACTACTCATCATGTCTTTGATTAGTGTCATTTCCACTGAATATGATTCCGATGAT  5360
     CircularRNA  --------------------------------------------------------------------------------  953
                                5.380                          5.400                          5.420                          5.440
cDNA(AK248174.1)  CCCGACTTATTGGTATGGAATTGATTTTCAGAAGTGATGTTCTTACCATTGGTGGTTCTCCAGTGGGATCTGGATTACGA  5440
     CircularRNA  --------------------------------------------------------------------------------  953
                                5.460                          5.480                          5.500                          5.520
cDNA(AK248174.1)  ATCTTCAATGCATCTTCTTGGTTTGCTGGGGAACTTCCTATTTTAATTTGACAACTTTGTAAAATAGTTGGGCTAAGTTT  5520
     CircularRNA  --------------------------------------------------------------------------------  953
                                5.540                          5.560                          5.580                          5.600
cDNA(AK248174.1)  GATGGTTTTACTGCTGATTGTATACTTTCAACTCAAGTAGCTTGTACACATGTCAGTTTGTTTCGTTCTGCATTCGTCAT  5600
     CircularRNA  --------------------------------------------------------------------------------  953
                                5.620                          5.640
cDNA(AK248174.1)  GGATTTGAAGATACAAAGCCTCCACACAAAAAAAAAAAAAAAAATAC  5646
     CircularRNA  -----------------------------------------------  953
```

Part III: Identified nucleotide patterns

Vacuolar cation/proton exchanger Cax2_circular RNA

Start	End	ModelScore	PatternScore	Patternlength	Pattern
110	119	927	202	10	TAGATGCCAT
312	321	927	194	10	TGTTTGCCAT
395	404	927	226	10	TGCATGCCAT
193	212	915	371	20	AACATGCCACTGGCCTTTGT
236	255	915	371	20	AACATGCCACTGGCCTTTAT

Mitogen-activated serine/threonine-protein kinase (Ctr1-like)_circular RNA

No motif detected

MicroRNA1126_circular RNA

Start	End	ModelScore	PatternScore	Patternlength	Pattern
47	64	555	210	18	TTATTGCACATAAAAGTG
127	144	555	270	18	CTGTTGCAAAGAAAAGTG
318	335	555	247	18	CTGTTGCATGTAAATTGG

Aux1_circular RNA

Start	End	ModelScore	PatternScore	Patternlength	Pattern
289	307	654	337	19	CTGCCTCCGCGCCCTCGCC
420	438	654	337	19	CCGCCTCCCCGCCCTCGCC
219	226	653	135	8	TCGGCTTC
312	319	653	209	8	TCCCCATC
324	331	653	209	8	TCCCCATC
339	346	653	185	8	TCGCCATC
351	358	653	186	8	TCCCCTTC
363	370	653	174	8	GCCCCATC
465	472	653	183	8	TCCGCATC

ATP-binding cassette Abcl3_circular RNA

Start	End	ModelScore	PatternScore	Patternlength	Pattern
111	130	292	267	20	CTTTTTCTTCGCTCTTCCGG
151	170	292	267	20	CTTTTTCTACTTTGTTTACG

18S rRNA_circular RNA1

Start	End	ModelScore	PatternScore	Patternlength	Pattern
57	69	232	240	13	TGGGTGGTGGTGC
81	93	232	213	13	TAGTTGGTGGAGC

18S rRNA_circular RNA2

No motif detected

18S rRNA_circular RNA3

Start	End	ModelScore	PatternScore	Patternlength	Pattern
70	82	193	237	13	TGGGTGGTGGTGC
94	106	193	211	13	TAGTTGGTGGAGC

18S rRNA_circular RNA4

Start	End	ModelScore	PatternScore	Patternlength	Pattern
42	54	148	233	13	TGGGTGGTGGTGC
66	78	148	207	13	TAGTTGGTGGAGC

18S rRNA_circular RNA5

Start	End	ModelScore	PatternScore	Patternlength	Pattern
50	62	126	231	13	TGGGTGGTGGTGC
74	86	126	205	13	TAGTTGGTGGAGC

18S rRNA_circular RNA6

Start	End	ModelScore	PatternScore	Patternlength	Pattern
72	84	197	238	13	TGGGTGGTGGTGC
96	108	197	211	13	TAGTTGGTGGAGC

18S rRNA_circular RNA7

Start	End	ModelScore	PatternScore	Patternlength	Pattern
26	45	193	275	20	GGCCGGGTCGGCCGGTCCGC
106	125	193	275	20	GGCCGGGTCGTGTTTTCGGC

Internal transcribed spacer 2 of 18S,5.8S,26S rRNA_circular RNA1 and 2

No motif detected

NADH dehydrogenase Nad9_circular RNA

Start	End	ModelScore	PatternScore	Patternlength	Pattern
12	27	542	255	16	TATTTGCGGAGTGGAT
277	292	542	255	16	TTTCCTCTGAGTGGAT
149	168	538	291	20	TATTTCCATCAGCCGGCCGG
201	220	538	291	20	TTCTTCCATCAATCATCCGG

Apocytochrome b_circular RNA

Start	End	ModelScore	PatternScore	Patternlength	Pattern
139	159	876	275	21	TTTTGGGGAGCAACAGTAATT
467	487	876	275	21	TTTTGGGGCATCCCGACAATT
4	11	871	193	8	CATATTTT
250	257	871	155	8	CGTTTTTT
280	287	871	213	8	CTTATTTT
386	393	871	213	8	CTTATTTT
432	439	871	203	8	CTTTTTTT
448	455	871	147	8	ATTTTTTT
520	527	871	143	8	CATATTGT

Cytochrome c oxidase Cox1_circular RNA1

Start	End	ModelScore	PatternScore	Patternlength	Pattern
174	194	469	297	21	TTATGCCGGCGATGATAGGTG
210	230	469	297	21	TTGTTCCGATTCTGATAGGTG

Cytochrome c oxidase Cox1_circular RNA2

Start	End	ModelScore	PatternScore	Patternlength	Pattern
117	136	505	261	20	ATAATATATCATTCTGGTTG
227	246	505	261	20	ATTACCAGCCATTCTGGAGG
253	267	502	222	15	TGATTTAGCAATTTT
286	300	502	244	15	TATTTCATCAATTTT
302	316	502	244	15	GGTTCTATCAATTTT

Cytochrome c oxidase Cox1_circular RNA3

Start	End	ModelScore	PatternScore	Patternlength	Pattern
260	279	537	263	20	ATAATATATCATTCTGGTTG
370	389	537	263	20	ATTACCAGCCATTCTGGAGG
176	196	533	299	21	TTATGCCGGCGATGATAGGTG
212	232	533	299	21	TTGTTCCGATTCTGATAGGTG

Cytochrome c oxidase Cox1_circular RNA4

Start	End	ModelScore	PatternScore	Patternlength	Pattern
315	323	2342	181	9	CAAGGATAT
576	584	2342	150	9	AAATAATAT
905	913	2342	232	9	CAATGTTAT
968	976	2342	226	9	CAATATTAT
1292	1300	2342	219	9	CCATGTTAT
1603	1611	2342	190	9	CCAGATTAT
437	445	2330	203	9	ATCAACTTT
481	489	2330	178	9	ATCTTTTTT
720	728	2330	204	9	AGCAATTTT
753	761	2330	240	9	ATCAATTTT
769	777	2330	240	9	ATCAATTTT
828	836	2330	183	9	ACCACTTTT
983	991	2330	197	9	ATCTCTTTT
1516	1524	2330	207	9	ATCCATTTT
1528	1536	2330	224	9	ATCACTTTT

Cytochrome c oxidase Cox1_circular RNA5

Start	End	ModelScore	PatternScore	Patternlength	Pattern
295	304	2163	173	10	CAAGGATATT
885	894	2163	240	10	CAATGTTATT
948	957	2163	218	10	CAATATTATA
1272	1281	2163	215	10	CCATGTTATT
417	425	2150	201	9	ATCAACTTT
461	469	2150	177	9	ATCTTTTTT
700	708	2150	202	9	AGCAATTTT
733	741	2150	239	9	ATCAATTTT
749	757	2150	239	9	ATCAATTTT
808	816	2150	181	9	ACCACTTTT
963	971	2150	196	9	ATCTCTTTT
1496	1504	2150	206	9	ATCCATTTT
1508	1516	2150	222	9	ATCACTTTT

Cytochrome c oxidase Cox1_circular RNA6

Start	End	ModelScore	PatternScore	Patternlength	Pattern
296	304	2378	181	9	CAAGGATAT
557	565	2378	150	9	AAATAATAT
886	894	2378	233	9	CAATGTTAT
949	957	2378	226	9	CAATATTAT
1273	1281	2378	220	9	CCATGTTAT
1584	1592	2378	190	9	CCAGATTAT
418	426	2366	203	9	ATCAACTTT
462	470	2366	179	9	ATCTTTTTT
701	709	2366	204	9	AGCAATTTT
734	742	2366	241	9	ATCAATTTT
750	758	2366	241	9	ATCAATTTT
809	817	2366	183	9	ACCACTTTT
964	972	2366	198	9	ATCTCTTTT
1497	1505	2366	208	9	ATCCATTTT
1509	1517	2366	224	9	ATCACTTTT

Cystathionine beta-lyase_circular RNA

Start	End	ModelScore	PatternScore	Patternlength	Pattern
20	35	285	246	16	CTGAATTCTTAGCTTC
218	233	285	246	16	CGTAACAGTTAGCTTC

BZip11_circular RNA

Start	End	ModelScore	PatternScore	Patternlength	Pattern
40	51	644	255	12	AGAGAGACAGAG
59	70	644	270	12	AGAGAGAGAGAG
73	84	644	270	12	AGAGAGAGAGAG
5	16	633	256	12	CCCTCCTCCTCC
300	311	633	223	12	CCCACCTCGTCC
373	384	633	220	12	CACACCTCCTCC
391	402	633	256	12	CCCTCCTCCTCC
408	419	633	256	12	CCCTCCTCCTCC
427	438	633	198	12	CTCTCCTCGTCC

ADP-ribosylation factor 1_circular RNA

Start	End	ModelScore	PatternScore	Patternlength	Pattern
45	57	562	237	13	AGGGGCTTGACTG
128	140	562	239	13	AGGGGCTTCTATG
204	223	557	292	20	GACAGATAATGTGGGTGTTT
262	281	557	292	20	GATTGTTTTCGTGGGTGTTT

Sec23/Sec24 transport protein_circular RNA

Start	End	ModelScore	PatternScore	Patternlength	Pattern
21	36	660	260	16	TGATGTGGTAGAAGCA
355	370	660	260	16	TCCTGATATAGAAGCA
332	350	659	270	19	CCTACACTGAGTTCGTACT
435	453	659	270	19	CTTACACTGACCTTTTGGT

Fumarase 2 _circular RNA

No motif detected

Inositol transporter 2_circular RNA

Start	End	ModelScore	PatternScore	Patternlength	Pattern
34	43	610	199	10	ATCATTGGCG
250	259	610	199	10	ATCAGGGGCG
286	295	610	212	10	ATCACCGGCG
22	30	597	198	9	GTGGCCGGC
49	57	597	188	9	GTCGGCGGC
97	105	597	150	9	GTGGCCGAC
163	171	597	175	9	GTGGTCGGC
178	186	597	126	9	TTCGTCGGC
190	198	597	166	9	GGCGTCGGC
237	245	597	216	9	GTCGCCGGC
336	344	597	150	9	GGCGCCGGG
367	375	597	216	9	GTCGCCGGC

Formin-like protein 20_circular RNA1

Start	End	ModelScore	PatternScore	Patternlength	Pattern
189	203	484	254	15	TAGGTATTTGTTCTT
204	218	484	276	15	TAGGTGTTAGATCTT
43	61	484	263	19	GGAGTACTGTTAGGTTGGT
317	335	484	263	19	GTTCTACTGTTAGATGACT

Formin-like protein 20_circular RNA2

Start	End	ModelScore	PatternScore	Patternlength	Pattern
62	80	298	255	19	TGTTCTTTAGGTGTTAGAT
178	196	298	255	19	TTCTGTTCTACTGTTAGAT

Alpha-mannosidase 1_circular RNA

Start	End	ModelScore	PatternScore	Patternlength	Pattern
126	143	821	274	18	AGCTCACCCTTTGAGAAT
282	299	821	274	18	ACCTGCCTTGTTGAGAAT
364	383	819	298	20	TAGGCAACACAATTGTAGGT
495	514	819	298	20	TTTGCAACACATATGTAACT

Chromosome segregation protein_circular RNA

Start	End	ModelScore	PatternScore	Patternlength	Pattern
281	292	631	251	12	GAGGCAGGCGGT
383	394	631	242	12	GAAGCAGGCGAT

Laccase 12_circular RNA

Start	End	ModelScore	PatternScore	Patternlength	Pattern
56	70	317	228	15	ATCCCGCCGGCCTTC
74	88	317	200	15	ACCCTCCCGGCATTC
101	115	317	218	15	GCCGCCACGGCCTTC

ARID/BRIGHT DNA-binding protein_circular RNA

Start	End	ModelScore	PatternScore	Patternlength	Pattern
8	17	352	202	10	CATGCAAACT
38	47	352	196	10	CATTCAAACC
215	224	352	174	10	CATGCACAAC
119	138	348	269	20	ATTGTAATTGTGCAATGGTG
146	165	348	269	20	AATGTAATTGACATGTGCTG

Ubiquitin-conjugating enzyme 11_circular RNA

Start	End	ModelScore	PatternScore	Patternlength	Pattern
4	12	293	199	9	CTGCTTTCA
22	30	293	179	9	CTGCTTACC
168	176	293	195	9	CTGCTTGCA

Probable microtubule-stabilizing protein_circular RNA

Start	End	ModelScore	PatternScore	Patternlength	Pattern
204	224	452	281	21	TGGATTCGATCAGGGTGTACA
276	296	452	281	21	TGAACTGGAAGTTGGTGTACA
105	125	444	352	21	TCTTGACTTGGCTAATGAGTT
138	158	444	352	21	TCTTGACTTGGATGATGAGTT

RNA-binding (RRM/RBD/RNP motifs) protein_circular RNA (ID: Ch4:252670767-252671078)

Start	End	ModelScore	PatternScore	Patternlength	Pattern
202	219	425	249	18	ACTACAGTGGTAATTCGG
295	312	425	249	18	ATCTGTGGGGTAATTCAG
21	41	417	337	21	GGTGGTTATGGAATGAGGATG
96	116	417	337	21	GGTGGTTATGGAATCGGTATG

RNA-binding (RRM/RBD/RNP motifs) protein_circular RNA (ID: Ch6:308455217-308455370)

Start	End	ModelScore	PatternScore	Patternlength	Pattern
109	121	203	264	13	CGCATATACAACA
136	148	203	264	13	CGCATATACAACA

RNA-binding (RRM/RBD/RNP motifs) protein_circular RNA (ID: Ch6:260427394-260427518)

Start	End	ModelScore	PatternScore	Patternlength	Pattern
7	17	165	196	11	GAGGAGGTCGA
19	29	165	202	11	GAGGAGGGCCA
52	62	165	168	11	GATGTGGGCGA
34	49	162	255	16	GGGAGGAGGGGACGGC
89	104	162	269	16	GGGATGACGGGAGGGC

Ribosomal protein L30/L7_circular RNA

Start	End	ModelScore	PatternScore	Patternlength	Pattern
327	343	592	269	17	GCCTTGTGTTTGGAAGA
415	431	592	269	17	GATGCATGTTTGGAAGA
33	52	586	333	20	GGGTTATGGAAAGCTCAACA
93	112	586	333	20	GGGTCTTGGAAAGCACAACA

Transducin/WD40 repeat-like protein_circular RNA

Start	End	ModelScore	PatternScore	Patternlength	Pattern
175	187	493	226	13	TCTCACCATCCTT
339	351	493	253	13	TATCTCCATCCAT
148	166	488	291	19	CTGTTGCATCAGCTCTCCA
209	227	488	291	19	CTGTTGCAGCCGTATTCCA

Abscisic acid-responsive (TB2/DP1, HVA22) protein_circular RNA

Start	End	ModelScore	PatternScore	Patternlength	Pattern
424	443	769	218	20	CACCACCTCCACGTACTCGG
460	479	769	270	20	CACCACCTGTTCCTGCACCG
484	503	769	256	20	CACCACCTATGCCACCACAG

Far upstream element-binding protein 2 _circular RNA

Start	End	ModelScore	PatternScore	Patternlength	Pattern
61	69	626	170	9	ACACTGTAT
174	182	626	219	9	CCACTGTTT
276	284	626	197	9	CCTCTGTTT
286	294	626	197	9	CCATTGTTT
141	149	621	163	9	TCTTTCTCT
185	193	621	199	9	TATTTATCT
227	235	621	170	9	TTTTTGTAT
245	253	621	205	9	TTTTTGTCT
261	269	621	188	9	TATTTCTCT
365	373	621	209	9	TTTTTATCT

Sec-independent protein translocase _circular RNA

Start	End	ModelScore	PatternScore	Patternlength	Pattern
279	288	1435	198	10	TCATTTTTCC
536	545	1435	218	10	TCGTCTTTCC
646	655	1435	178	10	TCCTTGTTCC
918	927	1435	205	10	TGCTCTTTCC
958	967	1435	147	10	GCCCCTTTCC
357	368	1427	203	12	TCGGATATTGAT
460	471	1427	267	12	TCGTATTTTGTT
783	794	1427	267	12	TCGTATTTTGTT
885	896	1427	243	12	TCGTTTTTTGAT

Ubiquitin-specific protease 17_circular RNA

Start	End	ModelScore	PatternScore	Patternlength	Pattern
63	74	1736	243	12	ATGCTAATGCTA
546	557	1736	251	12	ATGCTGATGTTA
1026	1037	1736	213	12	ATGCAAATGTTG
943	953	1737	229	11	ATATGATGTTT
1047	1057	1737	240	11	GCTTGATGTTT
1124	1134	1737	233	11	GCATGGTGTTT

Probable KH domain-containing splicing factor_circular RNA

Start	End	ModelScore	PatternScore	Patternlength	Pattern
34	44	271	216	11	CAACTTTCTCC
148	158	271	182	11	CAAATATGTCC
1	9	269	169	9	CCCTTCAAA
68	76	269	202	9	ACCTTCACA
137	145	269	187	9	ACCTTCTCA

Ribosomal protein L6_circular RNA

Start	End	ModelScore	PatternScore	Patternlength	Pattern
40	55	379	287	16	TGCCTGACTTCAAAAA
106	121	379	210	16	TCCCTGACCTTAAGAA
66	81	375	291	16	AAGGCCATTGATGCTG
90	105	375	286	16	AAGGCTATCGATGCTG

Glycyl-tRNA synthetase 2_circular RNA1

Start	End	ModelScore	PatternScore	Patternlength	Pattern
141	150	968	206	10	TTTAATCAAT
573	582	968	238	10	ATCAATCAAT
619	628	968	223	10	ATCAATCCAT
68	75	961	188	8	TTAGTTTC
90	97	961	198	8	TCATTTTC
109	116	961	219	8	TTATTTTC
128	135	961	183	8	TTATATTC
211	218	961	140	8	TCCTTTTC
270	277	961	139	8	TCATTTTT
401	408	961	219	8	TTATTTTC
608	615	961	189	8	TGATTTTC

Glycyl-tRNA synthetase 2_circular RNA2

Start	End	ModelScore	PatternScore	Patternlength	Pattern
142	150	1061	207	9	TTAATCAAT
574	582	1061	229	9	TCAATCAAT
616	624	1061	226	9	CCAATCAAT
730	738	1061	168	9	CCATTCAAN
68	75	1054	189	8	TTAGTTTC
90	97	1054	200	8	TCATTTTC
109	116	1054	220	8	TTATTTTC
128	135	1054	185	8	TTATATTC
211	218	1054	142	8	TCCTTTTC
270	277	1054	141	8	TCATTTTT
401	408	1054	220	8	TTATTTTC
608	615	1054	191	8	TGATTTTC

Kinesin-related protein 11-like_circular RNA

Start	End	ModelScore	PatternScore	Patternlength	Pattern
720	734	1655	301	15	TGCTAGTCAGGAAGA
927	941	1655	308	15	TGTTAGTGAGGAAGA
629	636	1652	228	8	TTGATGAG
689	696	1652	228	8	TTGATGAG
697	704	1652	163	8	TTTAGGAG
764	771	1652	197	8	TGGAGGAG
797	804	1652	216	8	TTGAGGAG
826	833	1652	194	8	CTGATGAG
1037	1044	1652	210	8	TGGATGAG

SAD1/UNC-84 domain protein 2_circular RNA

Start	End	ModelScore	PatternScore	Patternlength	Pattern
134	144	484	225	11	AGGCAAAAAAA
198	208	484	190	11	ACACAGAAAAA
105	124	480	150	20	TGTTATCGACAAGCCAAAAT
284	303	480	343	20	TGTTATGGGGATGGCAAAAT

Probable beta-1-4-glucosyltransferase_circular RNA

Start	End	ModelScore	PatternScore	Patternlength	Pattern
67	88	1123	327	22	CACTACATTTCCTGTTGGAACA
564	585	1123	362	22	CACTACATGTTCCATTGGAACA
713	734	1123	362	22	CACTACATGTTCTACTGGAACA
17	38	1119	391	22	CATGAGATCTGAACCAATGAAT
514	535	1119	391	22	CATGAGATCTGAACCAATGAAT
655	676	1119	349	22	CACGAGACCTGATCCAATGAAT

Unknown_circular RNA (ID: Ch7:4237959-4238849)

Start	End	ModelScore	PatternScore	Patternlength	Pattern
159	171	1227	295	13	AGGGAGGCATGCG
792	804	1227	295	13	AGGGAGGCATGCG
88	106	1209	276	19	GAGAGGACGAGGGAGGCAC
222	240	1209	314	19	GGGAGCAAGGGGGAGGCGC
721	739	1209	324	19	GAGAGGACGGGGGAGGCGC
855	873	1209	293	19	GGAAGCAGGGGGGAGGCGC

Unknown_circular RNA (ID: Ch2:476367671-476368169)

Start	End	ModelScore	PatternScore	Patternlength	Pattern
5	13	687	152	9	AGGGATCAA
351	359	687	212	9	ATGAATCAA
436	444	687	212	9	ATGAATCAC
490	498	687	151	9	AAGACTCAC
32	41	683	221	10	ACTGCAGATC
230	239	683	227	10	ACTGAAGATT
335	344	683	239	10	ACTGAAGATC

Probable long non-coding RNA_circular RNA (ID: Ch2:605441109-605441443)

Start	End	ModelScore	PatternScore	Patternlength	Pattern
181	199	449	330	19	GTTCTGACCCTTTTAGCAA
239	257	449	330	19	GATCTGACCCTTTTCGCAA

Unknown_circular RNA (ID: Ch3:100501939-100502294)

Start	End	ModelScore	PatternScore	Patternlength	Pattern
77	96	484	275	20	GGCATGAGAGGAGTAGCTTG
225	244	484	275	20	GCTGTGAGAGGAGTTCATAG

Probable long non-coding RNA_circular RNA (ID: Ch4:247058951-247059280)

Start	End	ModelScore	PatternScore	Patternlength	Pattern
15	24	449	199	10	GATGTATGGA
108	117	449	163	10	TGTGGAGGGA
127	136	449	210	10	TATGGATGGA
285	294	449	178	10	TATTTATGGA
4	12	448	220	9	AGAGGAGAT
63	71	448	220	9	AGAGGAGAT
118	126	448	199	9	AGGGGAGAT
205	213	448	158	9	AGAAGAGGT

Probable aminopeptidase_circular RNA (ID: Ch5:286201637-286202872)

Start	End	ModelScore	PatternScore	Patternlength	Pattern
193	200	1584	230	8	TGACATAT
225	232	1584	207	8	TTACATAT
244	251	1584	202	8	TGACACAT
317	324	1584	202	8	TGAAATAT
630	637	1584	164	8	TTCCATAT
727	734	1584	230	8	TGACATAT
274	284	1580	247	11	TGTGTTGTTTT
554	564	1580	193	11	TGGCTTGTTTG
583	593	1580	179	11	TGTATGGTTTT
644	654	1580	247	11	TGTGTTGTTTT
711	721	1580	218	11	TGTGTTGTTAG
835	845	1580	177	11	TGTTTAGTTTG

Unknown_circular RNA (ID: Ch5:6888869-6889034)

No motif detected.

Probable long non-coding RNA_circular RNA (ID: Ch5:53926495-53926935)

Start	End	ModelScore	PatternScore	Patternlength	Pattern
87	97	604	191	11	GCAGATCCAAT
282	292	604	225	11	TCTGAACCAAT
386	396	604	225	11	TAAGAACCAAT
153	173	602	286	21	GTGCCACAAATATTGTGTTGT
187	207	602	286	21	GAGAGATAGAAGTTGTGTTGT

Probable long non-coding RNA_circular RNA (ID: Ch1:363958561-363959513)

Start	End	ModelScore	PatternScore	Patternlength	Pattern
91	99	1313	212	9	CTGGATCTG
102	110	1313	211	9	CTGGAAATG
621	629	1313	224	9	ATGGAACTG
822	830	1313	235	9	CTGGAACTG
41	48	1301	181	8	TTTGTTTC
50	57	1301	196	8	TTTGTTAT
227	234	1301	169	8	TTTGATTT
294	301	1301	145	8	TTGGTTCT
338	345	1301	191	8	TTTGTTCT
399	406	1301	222	8	TTTGTTTT
427	434	1301	149	8	TTGGTTAT
483	490	1301	222	8	TTTGTTTT
581	588	1301	179	8	TTTGTTTG
630	637	1301	145	8	TTGGTTCT
719	726	1301	189	8	TTTGCTTT
747	754	1301	177	8	ATTGTTTT
814	821	1301	163	8	TTTGCTAT

Part IV

Primer sequences

Vacuolar cation/proton exchanger Cax2_circular RNA

F:

GCCTTATGCTCATATTATGTTATCTCATAGT

R:

TGTTATCTCCTTTTCATCCTCCTCAT

Vacuolar cation/proton exchanger Cax2

F:

GCCTTATGCTCATATTATGTTATCTCATAGT

R:

CACAGTTTTGTTGGGAACTTGTCA

Ctr1-like_circular RNA

F:

CAAATGTAATATTGTTTCTTGGGGCAT

R:

TGAGATCCTGCTCCAGAAATACTTTGA

Ctr1-like

F:

AAATTTCTGAAACAAACAATAATTGTGAT

R:

TGAGATCCTGCTCCAGAAATACTTTGA

MicroRNA1126_circular RNA

F:

GAATAACCAAATCCCAATTCTGCTT

R:

GAATATGCTAATGGAAAGGAGAGCTT

MicroRNA1126

F :

TAGACTATATCCCACCCAGTCCTGTT

R:

AGTCATCATCCTCTCAAGTAGCATGA

Aux1_circular RNA

F:

CCTCACCTACCGATCCGCAT

R:

CCACATCGCGTGCATGATCT

Aux1

F:

TGCGTGTCCTCAGTGTTTGTGTT

R:

TTATAAGCCATGATGACCATACCAT

Abcl3_circular

F:

GGTTTTGGGGAAGGCCTATGT

R:

ATAGCTGTCGCGATATAAATAACTATACTCA

Abcl3

F:

GGTTTTGGGGAAGGCCTATGT

R:

AAGATGAATACAGAAGTTAAACGAGCA

18S rRNA_circular RNA1 & 2

F:

GCTAACTAGCTATGCGGAGCCA

R:

GCTCTCAGTCTGTCAATCCTTGCT

18S rRNA_circular RNA1, 3, 4, & 6

F:

CGATTTGTCTGGTTAATTCCGTTA

R:

TCAGTCTGTCAATCCTTGCTATGTCT

18S rRNA

F:

CGCAAGGCTGAAACTTAAAGGAAT

R:

TCAGTCTGTCAATCCTTGCTATGTCT

Nad9_circular RNA

F:

CGATTTTGCTAGTCCTTGGGAA

R:

GTGTTACTTCGTCTGCACTTGTTTGT

Nad9

F:

AAGTAGCTATGAAGCCCAATCTACTGT

R:

CTATAACGAACACTCATTCCTATCTATTGA

Apocytochrome b_circular

F:

GCACTAGTTTTTATATCTCTCTTGGCT

R:

TCCCTAGGACTGCTATAACTCGCA

Apocytochrome b

F:

TGTGAGTTCTAATTACATGGAGTTGGAA

R:

ATAAAAGTGAAAGTAATGCAGATTAGCAT

Cox1_circular RNA1

F:

CACTGGGTGGACAGTATTGGGA

R:

CCTATCAGAATCGGAACAAACCA

Cox1_circular RNA2

F:

GACCTTCTTTCCCATGCATTTCT

R:

GGAGCTTTCCGCCACTACGA

Cox1_circular RNA3

F:

AAGCAGTGGAAAGAACAAAAAATGT

R:

CTTCCCACCGGATCCTCTGTT

Cox1_circular RNA4

F:

GCCATTCTGGAGGAGCAGATATT

R:

TAAGGCTGAGCTTAATAGGAGCAA

Cox1_circular RNA5

F:

GCATAGATTACCAGGCTCACGCTT

R:

CTGAGCTTAATAGGAGCAAGAGACTT

Cox1

F:

TGTTGAACAGAATCCAACCACACT

R:

GAAACTTCCTTCATAGTTAGGAGAGGTT

Cystathionine beta-lyase_circular RNA

F:

CTCAGTTTTCTAACTGGTTCATTGTCT

R:

AATTCAGCAATCTTCTGGGCATTA

Cystathionine beta-lyase

F:

AGCATAGAGCATACAAAATCTGCACTT

R:

GAATCACCAATTCAAATCGCATCT

BZip11-circular RNA

F:

GCTTACTCTCTCGTCGTCGTCACT

R:

TCTCTCTCTAGACGGACTCTGTCTCTCT

ADP-ribosylation factor 1_circular RNA

F8:

CTGTCTTGGCATAGTTTGTCACTGT

R8:

CCCTCCCCAGAGGTAGCACAT

Fumarase 2_circular RNA

F:

GAAGCAAACCATAAGAGAATTTCACA

R:

CAGATGCATCCCCTAATAATCTCAA

Sec23/sec24_circular RNA

F:

CCTTTCCTCCTCCACGTGACT

R:

TAGCCTTGCTTCTACCACATCACTT

Alpha-mannosidase 1_circular RNA

F:

TTATTGTTTTGATTATATGATATATAAAAAGGGTT

R:

GAAGCTCTGCATCTTGTCGTCCT

Chromosome segregation protein_circular RNA

F:

ACTTGATTTGTGCTGCGACCAT
R:
CCCGCAACATGCCTAGATGA

Chromosome segregation protein

F:
GGGCAAGTTGACATGTGATGCTT
R:
GAGATATGCTCCTAAGCCCCTTACT

Inositol transporter 2_circular RNA

F:
ATGCTATTCCTCCCCGAATCA
R:
CGCCATGCTCACGATCATTT

Formin-like protein 20_circular RNA1

F:
ACCTATATGGTCCTGACTTAGGTATTTGT
R:
GAATCTAGGTGGAAAGCTGCTATTCT

E3 UFM1-protein ligase 1_circular RNA (false positive candidate)

F:
GAAAGACTTGAGCGATCAATGCT
R:
AGTCTCTGAAGAAACTTGTGCTATCA

Ref genes:

Vacuolar ATPase (MLOC_59475)

F:
GATGCTGGGTGGGGGTTGAT
R:
GCCAGACCCAACAGGCAAAT

Gadph (MLOC_18233)

F:

CCAAGGCTGTTGGTAAGGTT

R:

GACACATCCACAGTGGGAAC
